# Analysis of Tumor Suppressor Genes Based on Gene Ontology and the KEGG Pathway

**DOI:** 10.1371/journal.pone.0107202

**Published:** 2014-09-10

**Authors:** Jing Yang, Lei Chen, Xiangyin Kong, Tao Huang, Yu-Dong Cai

**Affiliations:** 1 The Key Laboratory of Stem Cell Biology, Institute of Health Sciences, Shanghai Jiao Tong University School of Medicine (SJTUSM) and Shanghai Institutes for Biological Sciences (SIBS), Chinese Academy of Sciences (CAS), Shanghai, People’s Republic of China; 2 College of Information Engineering, Shanghai Maritime University, Shanghai, People’s Republic of China; 3 Department of Genetics and Genomic Sciences, Mount Sinai School of Medicine, New York, New York, United States of America; 4 Institute of Systems Biology, Shanghai University, Shanghai, People’s Republic of China; University of North Carolina School of Medicine, United States of America

## Abstract

Cancer is a serious disease that causes many deaths every year. We urgently need to design effective treatments to cure this disease. Tumor suppressor genes (TSGs) are a type of gene that can protect cells from becoming cancerous. In view of this, correct identification of TSGs is an alternative method for identifying effective cancer therapies. In this study, we performed gene ontology (GO) and pathway enrichment analysis of the TSGs and non-TSGs. Some popular feature selection methods, including minimum redundancy maximum relevance (mRMR) and incremental feature selection (IFS), were employed to analyze the enrichment features. Accordingly, some GO terms and KEGG pathways, such as biological adhesion, cell cycle control, genomic stability maintenance and cell death regulation, were extracted, which are important factors for identifying TSGs. We hope these findings can help in building effective prediction methods for identifying TSGs and thereby, promoting the discovery of effective cancer treatments.

## Introduction

Currently, cancer is the second most common cause of death, following cardiovascular disease. Cancer that originates from the epithelial cells or mesenchymal cells is characterized by uncontrolled cell proliferation. In malignancy, cancer cells invade adjacent normal tissues and metastasize through blood circulation, lymphokinesis or body cavity transfer. In this process, proteins that are coded by tumor suppressor genes (TSGs) play vital roles in the mechanisms associated with cellular growth, DNA damage, apoptosis and metabolic regulation [1].

It has been reported that tumor suppressor inactivation and haploinsufficiency occur at several different levels in tumor patients. In the past decades, many classic TSGs have been widely identified, which are silenced by recurrent LOH (loss of heterozygosity) and physical deletion in the tumor genome. Increasing evidence has shown the abnormal DNA methylation or histone modifications, and non-coding RNA affect the expression of TSGs at the epigenetic level and post-transcriptional level, respectively [2], [3].

The first identified TSG was retinoblastoma protein (Rb), which was identified by studies of familial retinoblastoma in early childhood. Based on this, the “two-hit” hypothesis was introduced by Knudson in 1971 [4], [5]. As a guardian to the normal cell cycle, the Rb protein is responsible for the G1/S checkpoint and maintains regular cell growth. In addition to loss of heterozygosity, the high frequent mutations or partial deletions are mainly located in exon13∼exon17 of Rb and have been found in various cancer types, especially in lung cancer, breast cancer, osteosarcoma and bladder cancer, with a frequency ranging from 15% to 50% [6]–[10]. Like Rb, the p53 protein family as a key element of the tumor suppression network, exerts much of its growth arrest in the cell cycle and induces apoptosis. Changes to p53 are involved in various cancers. Genetic variation mainly missense mutations, in p53 are often regarded as the driver mutations that confer apoptosis evasion and abnormal cell growth of tumor cells, especially those that originate from the epithelial tissue. More than 86% of point mutations occur in the evolutionary conservative regions, especially four mutation hotspots [11], [12]. In addition, p53 is silenced via LOH in the genome and hypermethylation at the epigenetic level in cancer patients [13], [14].

Like Rb and p53, some tumor suppressor proteins control cell behaviors directly by arresting cell proliferation, disturbing the cell cycle and inducing apoptosis, and these are called the gate-keepers. The destiny of a cell is also affected indirectly by some tumor suppressor proteins that are associated with mutation accumulation and genome stability maintenance such as BRCA1 and BRCA2, which are also referred to as caretakers [15], [16].

Additionally inherited mutations of BRCA1 and BRCA2 (breast cancer 1/2) are associated with patients who have hereditary breast cancer, accounting for 5–10% of all breast cancer patients [17]. Loss function of their products causes abnormal homologous recombination and genome instability, which increases the susceptibility to breast and ovarian cancer [18].

Unlike the activated oncogene, suppression of TSGs occurs more frequently, providing evidence for understanding the initiation and progress of various cancers. The identification and subsequent activation of TSGs can facilitate controlling cell proliferation, restraining the biological activity of cancer. In this study, we attempted to investigate the characteristics of TSGs. The TSGs retrieved from the web-based database, TSGene (tumor suppressor gene database), facilitated our investigation of TSGs. These genes were called ‘positive genes’ and all of the remaining genes in the STRING were selected as ‘negative genes’. Gene Ontology (GO) is an acknowledged bioinformatics tool for representing gene product properties across all species by defined GO terms, the function of the genes and their products were represented by the GO terms and predicted by the GO annotation effectively [19], [20]. In contrast, the Kyoto Encyclopedia of Genes and Genomes (KEGG) is a comprehensive database based on known molecular interaction networks and is usually used to understand biological pathways and systems [21]. In view of this, the enrichment scores of the GO terms and KEGG pathways were used to encode all genes investigated in this study. Minimum redundancy maximum relevance (mRMR) and incremental feature selection (IFS) [22] combined with a prediction engine were employed to analyze these features. The analysis of the extracted GO terms and KEGG pathways suggests that they are related to TSGs. In addition, the extracted GO terms and KEGG pathways were used to predict the novel TSGs, indicating that they may help build effective computational methods for identifying TSGs.

## Materials and Methods

### Dataset

We compiled 716 human TSGs in the TSGene database (http://bioinfo.mc.vanderbilt.edu/TSGene/download.cgi), which were collected from two resources: public databases and literature reports. In detail, 187 (human) and 170 (human) known TSGs were retrieved from UniProtKB (28 January, 2012) and the TAG database (http://www.binfo.ncku.edu.tw/TAG/GeneDoc.php) (29 March, 2012), respectively, with only 41 overlapped genes by mapping to the Entrez gene symbols. By combining two exhaustive searches, PubMed and Gene Reference Into Function (GeneRIF) [23], [24], and after overlapping and synonymous genes with same the Entrez gene ID were filtered, 637 protein-coding TSGs and 79 non-coding TSGs were identified [25]. Because the encoding method described in Section “Encoding method” employed the neighbors of each investigated TSG in the STRING, we obtained 615 genes with their ensembl protein IDs in the STRING. These genes were termed ‘positive genes’ and are given in Table S1. The remaining 17,985 ensembl protein IDs in the STRING were considered ‘negative genes’.

The number of negative genes was much larger than that of the positive genes. This is an imbalanced dataset. Inspired by some studies dealing with this type of data [26], [27], we divided the 17,985 negative genes into six datasets, 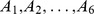, where 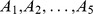 contained 3,075 negative genes and, 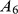 contained 2,610 negative genes. The 615 positive genes were put into each of these datasets, comprising six new datasets, 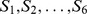, *i.e.*, *S_i_* (*i* = 1,2,3,4,5,6) consisting of genes in *A_i_* (*i* = 1,2,3,4,5,6) and 615 positive genes.

### Encoding method

To analyze the characteristics of the TSGs, it is very important to encode each gene with its essential properties. GO is an acknowledged bioinformatics tool for representing gene product properties across all species by defined GO terms, while KEGG is a comprehensive database based on known molecular interaction networks and usually includes the biological pathway and system information [21]. Therefore, we selected GO terms and KEGG pathways to code each gene. TSGs have a strong relationship with some GO terms and KEGG pathways. On the other hand, the enrichment method of GO can reflect the relationship between the genes and GO terms [28]. It is reasonable to use this method to encode genes and analyze the relationship of the TSGs and GO terms. Furthermore, this method can also be extended to KEGG pathways [29] to find the relationship between the genes and KEGG pathways.

#### GO enrichment

For one gene *g* and one GO term GO*_j_*, the GO enrichment score is defined as the −log_10_ of the hypergeometric test *P* value [28]−[30] of a gene set *G* containing *g*’s direct neighbors in the protein-protein interaction network of STRING and GO term GO*_j_*, which can be calculated by:
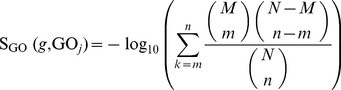
(1)where *N* is the number of overall proteins in human, *M* is the number of proteins annotated to the GO term GO*_j_*, *n* is the number of proteins in *G*, and *m* is the number of proteins in *G*, which are annotated to the GO term GO*_j_*. The high score for one gene and one GO term implies that the gene and GO term have a special relationship. The 12,877 GO terms induced 12,877 GO enrichment scores.

#### KEGG enrichment

For one gene *g* and one KEGG pathway P*_j_*, the KEGG enrichment score is defined as the –log_10_ of the hypergeometric test *P* value [29] of a gene set *G* containing *g*’s direct neighbors in the protein-protein interaction network of STRING and KEGG pathway P*_j_*, which can be computed as follows:
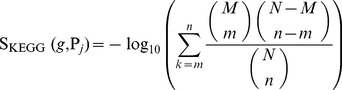
(2)where *N* is the number of overall proteins in human, *M* is the number of proteins in the KEGG pathway P*_j_*, *n* is the number of proteins in *G*, *m* is the number of proteins in both *G* and P*_j_*. Additionally, the higher the KEGG enrichment score for *g* and P*_j_*, the stronger the relationship between them. The 239 KEGG pathways induced 239 features of KEGG enrichment scores.

Each of the 12,877 GO enrichment scores or each of the 239 KEGG enrichment scores can be considered a dimension. Accordingly, each gene *g* can be represented by a vector in 12,877+239 = 13,116-D space, which is formulated as:
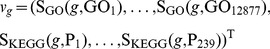
(3)


### Prediction method

Dagging is a well-known meta classifier. The main idea of this classifier is to integrate multiple classifiers derived from a single learning algorithm that is trained by disjoint samples of the original dataset [31]. The brief description of this method is as follows. For a training dataset 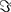 with samples 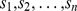, construct *k* disjoint subsets by randomly taking 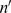 samples in 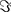
_,_ without replacement, such that 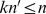. These subsets were used to train a basic classifier (*e.g.,* support vector machine) and derive *k* classification models, 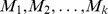. For a query sample, each of these models *M_i_* (1≤*i*≤ *k*) provides a predicted result. The predicted result of dagging integrated these results by majority voting.

In Weka 3.6.4 [32], the classifier “Dagging” implements the dagging classifier mentioned above. Here, it was adopted as the prediction engine. For convenience, it was run with its default parameters. In detail, the SMO (Sequential Minimal Optimization), which implements John Platt’s sequential minimal optimization algorithm for solving the optimization problem during the training of a support vector classifier using polynomial or Gaussian kernels [33], [34], is set as the basic classifier, and *k* is set to 10.

### Evaluation method

Ten-fold cross-validation is a widely used cross-validation method for evaluating the performance of different classification models [35]−[38]. Compared to the Jackknife test [39], [40], the 10-fold cross-validation test requires less computing time and provides similar results for a given dataset. Therefore, the current study adopted this cross-validation method to evaluate the performance of the prediction method.

To represent the predicted results of a two-class classification problem, a confusion matrix was often employed, which contained the following four entries: true positives (TP), true negative (TN), false positives (FP), and false negative (FN) [41], [42]. Based on these values, the prediction accuracy (ACC), specificity (SP), sensitivity (SN) [42] and Matthews’s correlation coefficient (MCC) [43] were often used to evaluate the predicted results, which can be computed by
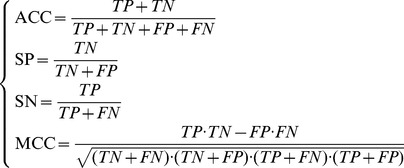
(4)


As mentioned in Section “Dataset”, five datasets were constructed in this study to reduce the size difference of the ‘positive genes’ and ‘negative genes’. However, each dataset still had very different class sizes. In detail, the number of ‘negative genes’ was at least 4 times as many as that of ‘positive genes’. Thus, the ACC is not appropriate for evaluating the predicted results on the whole. MCC, as a balanced measure even if the classes are of very different sizes, was employed as the key measurement.

### Feature selection method

As mentioned in Section “Encoding method”, each gene was represented by 13,116 features of the enrichment scores, which indicated the relationship between the genes and GO terms or KEGG pathways. TSGs are related to some GO terms and KEGG pathways. To identify key GO terms and KEGG pathways, some feature selection methods were employed in this study. The procedure of the feature selection method included two stages: (I) Cramer’s coefficient [44], [45], which used to discard non-essential features and (II) minimum redundancy maximum relevance (mRMR), incremental feature selection (IFS) [22] and Dagging [31] for further selection.

The Cramer’s coefficient [44], [45], derived from the Pearson Chi-square test [46], is a statistical measure of two variables. Its value is between 0 and 1. According to the fact that a high Cramer’s coefficient of two variables indicates a strong association of two variables, features with low Cramer’s coefficients to samples’ class labels were deemed non-essential features. Here, we used 0.1 as the threshold and features with Cramer’s coefficients lower than 0.1 were excluded.

The second stage of the feature selection involved the mRMR, IFS and Dagging. In detail, the mRMR method sorted the remaining features in two lists, while the IFS and Dagging were used to extract key features based on the feature lists obtained by the mRMR method. The mRMR method, proposed by *Peng et al.* [22], has two criteria: Max-Relevance and Min-Redundancy, producing the following two feature lists: (I) MaxRel feature list and (II) mRMR feature list. The MaxRel feature list sort features only based on the Max-Relevance criterion, while the mRMR feature list sort features based on both the Max-Relevance and Min-Redundancy. In this study, these two lists were formulated as follows:
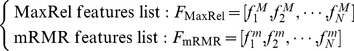
(5)where *N* is the total number of features. The mRMR method has been widely used in recent years to analyze complicated biological problems [36], . Since the mRMR feature list was built with both the Max-Relevance and Min-Redundancy criteria in mind, it was used to extract important features by combining the IFS and Dagging. This procedure was as follows:

(I) Construct *N* feature set from the mRMR features list   *F*_mRMR_, say 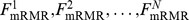, such that   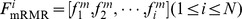, *i.e.* 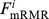 consisted   of the first *i* features in *F*_mRMR_.

(II) For each 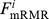, Dagging was conducted on samples   represented by features in 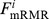, evaluated by 10-fold   cross-validation, thereby obtaining ACC, SP, SN and MCC   (cf. **Eq. 4**).

(III) The feature set that can produce the maximum MCC is the   optimal feature set. Additionally, an IFS-curve was plotted   with the MCC value as its Y-axis and the superscript *i* of   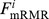 (the number of features that participate in the   classification) as its X-axis.

## Results and Discussion

### Results of the feature selection

As mentioned in Section “Dataset”, 6 datasets, 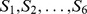, were constructed. For each, we calculated the Cramer’s coefficients of the features and the samples’ class labels. Then, the features with Cramer’s coefficients lower than 0.1 were excluded. The remaining features were kept for the further selection. The number of remaining features for each dataset is shown in **Table 1**.

**Table 1:** The number of remaining features after using Cramer’s coefficient to exclude non-essential features.

Dataset	Number of remaining features
*S*_1_	3,347
*S*_2_	3,837
*S*_3_	4,632
*S*_4_	4,270
*S*_5_	4,956
*S*_6_	6,661

The mRMR method, IFS method and Dagging were used to analyze the remaining features for each dataset *S_i_*. The mRMR program, downloaded from http://research.janelia.org/peng/proj/mRMR/, was executed on each dataset *S_i_*, in which each sample was represented by the remaining features. For convenience, the mRMR method was conducted with its default parameters. As mentioned in Section “Feature selection method”, the MaxRel features list and mRMR features list were obtained for each dataset *S_i_*. However, to reduce the computation time, we only obtained the first 500 features in each of the two feature lists, which are summarized in Table S2.

**Figure 1:**
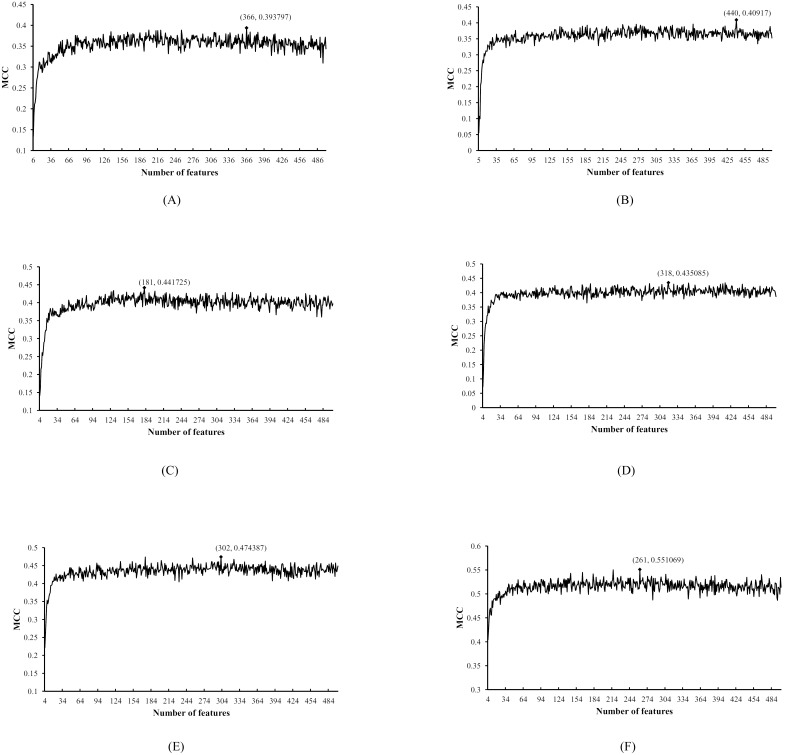
Six IFS-curves for six datasets. In detail, (A) shows the IFS-curve for the dataset *S*_1_; (B) shows the IFS-curve for the dataset *S*_2_; (C) shows the IFS-curve for the dataset *S*_3_; (D) shows the IFS-curve for the dataset S_4_; (E) shows the IFS-curve for the dataset *S*_5_; (F) shows the IFS-curve for the dataset *S*_6_. The Y-axis represents the Matthews’s correlation coefficient (MCC) and the X-axis represents the number of features participating in the classification model.

The IFS method and classifier Dagging were executed according to the mRMR features list for each dataset *S_i_*, which was evaluated by 10-fold cross-validation. The SNs, SPs, ACCs and MCCs obtained for each dataset *S_i_* are given in Table S3. For easy observation, we plotted an IFS-curve for each dataset *S_i_*. The six IFS-curves are shown in **Figure 1**; the maximum MCCs for datasets 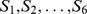 were 0.3938, 0.4092, 0.4417, 0.4351, 0.4744, and 0.5511, respectively. These values are listed in **Table 2**, in which the numbers of the features used to obtain these maximum MCCs are also listed. In detail, by using the first 366, 440, 181, 318, 302, and 261 features in the mRMR features lists of the six datasets (see Table S3), respectively, the MCCs calculated by **Eq. 4** were 0.3938, 0.4092, 0.4417, 0.4351, 0.4744, and 0.5511, respectively. Accordingly, six optimal feature sets, *OS*_1_, *OS*_2_, …, *OS*_6_ can be obtained by selecting the first 366, 440, 181, 318, 302, and 261 features in six mRMR feature lists of six datasets, respectively.

**Table 2:** The number of features in the optimal feature set for each dataset and the MCC values obtained by using these features.

Dataset	Number of features inthe optimal feature set	Maximum MCC value
*S*_1_	366	0.3938
*S*_2_	440	0.4092
*S*_3_	181	0.4417
*S*_4_	318	0.4351
*S*_5_	302	0.4744
*S*_6_	261	0.5511
Mean		0.4509

### Analysis of the GO terms in the total optimal feature set

As mentioned in Section “Results of the feature selection”, six optimal feature sets were obtained. We took the union operation of these sets and obtained a new dataset denoted by *OS* (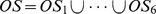) and termed the total optimal feature set, consisting of 708 enrichment features of the GO terms and 9 enrichment features of the KEGG pathways, which are available in Table S4. The analysis of 708 GO terms is described below.

**Figure 2:**
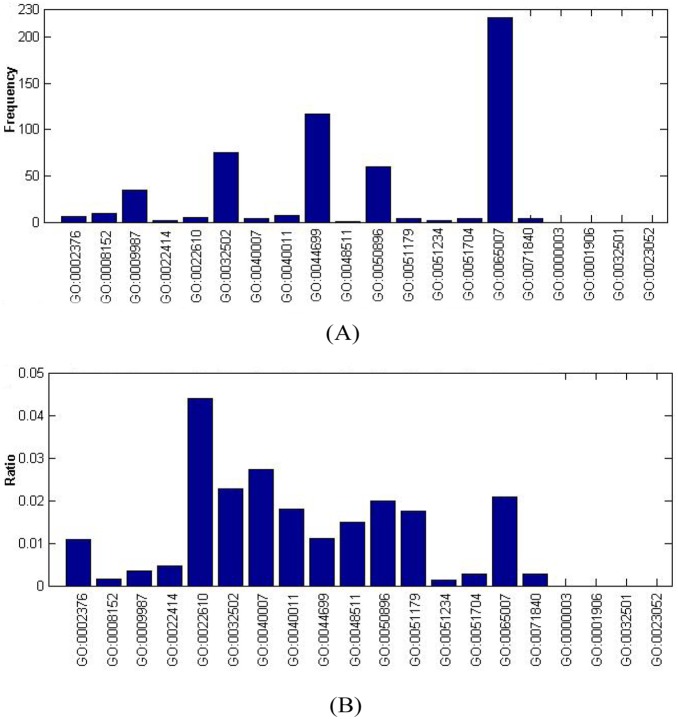
Frequency and ratio of GO terms of biological process in *OS*. (A) Frequency of GO terms of biological process in *OS*. (B) Ratio of GO terms of biological process in *OS*.

**Figure 3:**
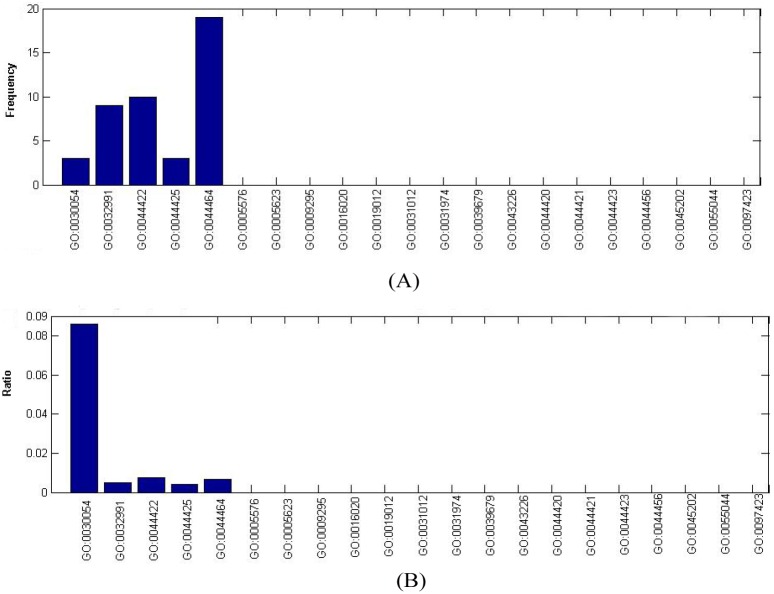
Frequency and ratio of GO terms of cellular component in *OS*. (A) Frequency of GO terms of cellular component in *OS*. (B) Ratio of GO terms of cellular component in *OS*.

**Figure 4:**
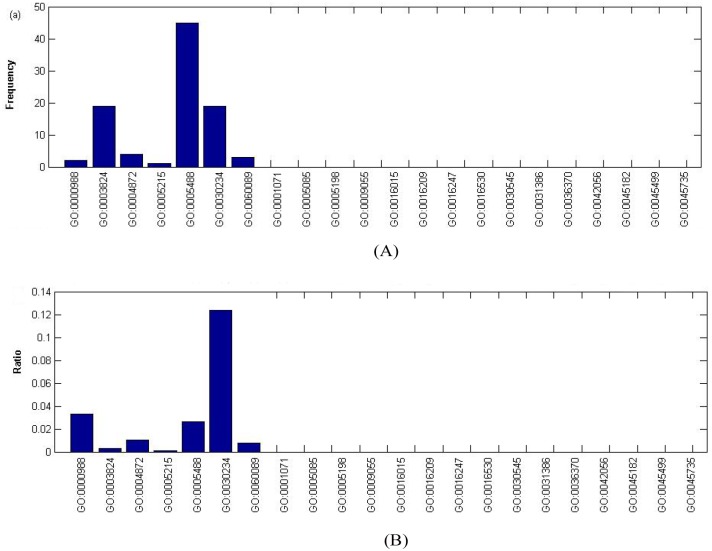
Frequency and ratio of GO terms of molecular function in *OS*. (A) Frequency of GO terms of molecular function in *OS*. (B) Ratio of GO terms of molecular function in *OS*.

Seven hundred and eight GO terms can be divided into the following three parts: (1) Biological Process (BF); (2) Cellular Component (CC); and (3) Molecular Function (MF). We mapped the 708 GO terms to the children terms of three GO domains. As we can see in **Figures 2**−**4**, the GO terms in the *OS* were significantly enriched in some specific children terms with a high frequency and high ratio, which is defined as “the number of each GO term”/“the scale of the number of its children terms”.

**Biological process GO terms.** The top five biological process GO terms of the frequency shown in **Figure 2(A)** are GO: 0065007: biological regulation (221), GO: 0044699: single-organism process (117), GO: 0032502: developmental process (75), GO: 0050896: response to stimulus (60) and GO: 0009987: cellular process (35). The top five biological process terms with large base numbers that perform fundamental functions in organisms and tumor suppressor proteins may be functional disturbance in health maintenance of cancer patients.

For the ratio of the biological process GO terms shown in **Figure 2(B)**, the top five are GO: 0022610: biological adhesion (4.39%, 5/114), GO: 0040007: growth (2.72%, 4/147), GO: 0032502: developmental process (2.28%, 75/3294), GO:0065007: biological regulation (2.09%, 221/10551) and GO:0050896: response to stimulus (2.0%, 60/3001). The GO terms biological adhesion and response to stimulus should be noted and relevant TS proteins act in the alarm reaction and have protective roles in tumorigenesis and the metastasis process. The GO term single-organism process involved in death and cell proliferation is highlighted too, although its percentage is not high. The destiny of an organism is critically regulated by the cell cycle and apoptosis in which TSGs play an important part. TSGs act like brakes on a car and are involved in maintenance of the cell cycle checkpoints and apoptosis induction [53].

Cells are under constant attack by various agents and oncogenic DNA variants form because of endogenous (normal cell metabolite) and exogenous agents (chemical species and physical mutagens). To maintain genome stability, TSGs participated in multiple mechanisms to repair DNA damage and arrest cell proliferation. In DNA double-strand break repair (DSBR), several TS genes, including ATM, NBS1, BRCA1 and BRCA2, are activated by DNA damage to induce cell cycle checkpoint arrest and DSB repair complex formation [54]. The highly conserved DNA mismatch repair (MMR) proteins composed of MSH2, MLH1, PMS1 and PMS2 tumor suppressor proteins in people, are required to correct base mismatches that are formed in response to exogenous or endogenous substances. If the expression of MLH1 or MSH2 is suppressed, cells lose the ability to perform mismatch repair and have resistance to alkylation mutagens that would normally activate G2/M checkpoint or apoptosis [55]. In nucleotide excision repair (NER), the DNA repair genes are regulated by p53 to remove bulky DNA adducts including pyrimidine dimmers induced by UV [56]. Normal, unrepaired DNA variants promote cells apoptosis.

Normally, cell proliferation is tightly regulated in different periods of the cell cycle. The pRb (retinoblastoma protein), known as the first TSG, maintains the G1/S checkpoint through its regulation of the E2F family. Inactivation of pRb, which caused by mutations, promoter methylation or interaction with oncoproteins, results in loss of control of the checkpoint R, allowing for uncontrolled cell proliferation [57], [58]. In addition, cancer cells inhibit the expression of many other tumor suppressor proteins to gain malignant proliferation ability. For example, with mutations or the low expression of TGF-βR II (transforming growth factor βreceptor II) and its downstream proteins Smad2/3/4 (SMAD family member 2/3/4), cancer cells will be insensitive to the proliferation inhibition of TGF [59], [60]. Similar to pRb, the INK4 (cyclin -dependent kinase inhibitor, *e.g.,* p16INK4A) family, which is regulated by TGF-β, can block CDK, causing cell growth arrest in a different period of the cell cycle. The dysfunction of INK4 or TGF-βR II will allow cells to pass through the checkpoint abnormally and accumulate variations [61], [62].

Apoptosis, known as programmed cell death, can be initiated by two distinct signaling pathways, BCL2 induced and death receptor induced, which ultimately converge in the caspase cascade. The most famous TSG, p53, is mutated in ∼50% of human cancers and related to some tumor suppression network [14]. p53 is a transcriptional regulator that can be activated by DNA damage, certain oncogenes and other cytotoxic stress signals, triggering cell cycle arrest (G1/S checkpoint), DNA repair and apoptosis. Dysfunction of p53 caused by mutations or methylation prevents the damage-induced cell cycle arrest and apoptosis [63], [64]. As a TSG, PTEN (phosphatase with tensin homology) negatively regulates the PI3K (the phosphatidylinositol 3-kinase) pathway, preventing inappropriate metabolism via effects on TOR and promoting cell proliferation via effects on proapoptotic proteins [65]. CYLD(cylindromatosis), first identified as a TSG in the familial cylindromatosis, is a DUB (deubiquitinase) of the USP subfamily. Multiple myeloma patients with dysfunction of CYLD have abnormal activation of NF-kB and cell cycle and apoptosis dysfunction [66], [67]. The insufficient activation of caspase 8 (apoptosis-related cysteine peptidase), a key TS gene in the caspase cascade, leads to the interruption of signal transduction from death receptors, inducing normal apoptosis [68], [69].

Many tumor cell types acquire the capacity to invade and metastasize though loss of cell-cell adhesion or cell-ECM (extracellular matrix) junctions. The silencing or suppression of E-cadherin, which is regulated by promoter methylation, histone methylation, transcriptional repression or frequent mutations cause EMT (epithelial-mesenchymal transition), disruption of cell contacts, tumor cell detachment and invasion [70], [71]. Integrins, a family of heterodimeric transmembrane proteins, mediate cell–ECM (extracellular matrix) interactions. Aberrant integrin can induce the activation of proteolytic enzymes and cause degradation of the extracellular matrix and basement membrane, promoting tumor cells metastasis [72]. MMPs (matrix metalloproteinase) are endopeptidases that are involved in the breakdown of the extracellular matrix; they are regulated by inhibitors, TIMPs (Tissue Inhibitor of Metalloproteinases). Loss of function of TIMPs, which are TSGs, may cause a MMP/TIMP equilibrium shift into a malignant status [73], [74].

Except the features above, which help us comprehend the relevance between tumor suppressors and specific GO terms or pathways, some rare investigated terms were highlighted such as metabolic process (GO:0008152), reproductive process (GO:0022414), locomotion (GO:0040011), localization (GO:0051179)/establishment of localization (GO:0051234) and multi-organism process (GO:0051704). These features remind us tumor suppressors participate in protein localization intracellular, various cells migration and locomotion intercellular, complex metabolic process and multi-organism process in the whole organism. Particularly, in some tumor types, tumor suppressors play key roles in reproductive process, usually related to hormone and hormone receptors. These features are not studies deeply as others, but need more attention to mine novel tumor suppressors.

**Cellular component GO terms.** It can be seen from **Figure 3(A)** that the top five CC GO terms with regard to frequency are GO:0044464: cell part (19), GO:0044422: organelle part (10), GO:0032991: macromolecular complex (9), GO:0030054: cell junction (3), and GO:0044425: membrane part (3), which also have a corresponding high percentage. Their ratios (cf. **Figure 3(B)**) are GO: 0030054: cell junction (8.57%, 3/35), GO: 0044422: organelle part (0.73%, 10/1361), GO: 0044464: cell part (0.67%, 19/2823), GO: 0032991: macromolecular complex (0.49%, 9/1824), and GO:0044425: membrane part (0.41%, 3/724). Cell junction is a cellular component that forms connections between two cells or between a cell and the extracellular matrix. As discussed above, TSGs such as E-cadherin and integrin play critical roles in tumor cell adhesion and metastasis. Additionally, organelles, including the mitochondria, ribosomes and UPS (ubiquitin-proteasome system), participate in the biological process involved in carcinogenesis. Many macromolecular complexes consist of tumor suppressor protein inside cells, such as TSgene SMAD2/3(SMAD family member 2/3) in the SMAD protein complex [75] and SMARCB1(SWI/SNF related, matrix associated, actin dependent regulator of chromatin, subfamily b, member 1) in the Swi/Snf complex [76].

**Molecular function the GO terms.** It can be observed from **Figure 4(A)** that the five highest frequency of MF GO terms are GO: 0005488: binding (45), GO: 0003824: catalytic activity (19), GO: 0030234: enzyme regulator activity (19), GO:0004872: receptor activity (4), and GO:0060089: molecular transducer activity (3). On one hand, these high frequency MF GO terms consist of a huge number of proteins that perform basic biological functions; on the other hand, the catalytic activity and enzyme regulator are involved in most vital biological processes, including cell proliferation, DNA damage repair and apoptosis. The cell junction requires protein binding and enzymes catalyze, which can involve biological processes such as phosphorylation, acetylation, the cell-extracellular matrix link and cell cycle control. The transcription factor Dp (DPDP-polypeptide) forms a complex with E2F1 to regulate its binding to DNA and the expression of certain genes (such as myc) catalyzed by enzymes [77]. Genomic instability is essential in almost all tumor factors, and mutations in ATM (ataxia telangiectasia mutated) which belongs to the PI3/PI4-kinase family, leave DSBs (DNA double-strand breaks) unrepaired [78]. The receptor proteins transduce extracellular or intracellular messenger to the biological effectors, triggering a serial biochemical reaction. The typical receptor protein and tumor suppressors in the TGF-β signaling pathway are TGF-βR II and BMPR2 (bone morphogenetic protein receptor, type II (serine/threonine kinase)) [79]. The five most common MF GO terms (cf. **Figure 4(B)**) are GO: 0030234: enzyme regulator (12.33%, 19/154), GO: 0000988: protein binding transcription factor activity (3.28%, 2/61), GO: 0005488: binding (2.64%, 45/1703), GO:0004872: receptor activity (1.02%, 4/391), and GO:0060089: molecular transducer activity (0.74%, 3/405). The corresponding percentages of the top five MF terms are similar to the top MF frequency, which are associated with the BP percentage and CC percentage and participate in tumorigenesis at different level.

**Directed acyclic graph (DAG) analysis of the GO children terms.** To further understand the function of the selected GO terms, we analyzed the directed acyclic graph of the GO children terms. We found that the GO children terms clustered in several particular modules under the primary GO terms discussed above. In addition to cell adhesion, the cellular response to UV-induced DNA damage and subsequent activated apoptotic signaling pathway and cell cycle regulation, phosphate metabolism, signal transduction and some molecular complex were highlighted in the biological modules.

The phosphorus utilization including phosphorylation and dephosphorylation catalyzed by kinases and phosphatases, respectively, is a key mechanism in a number of vital cellular pathways such as the cell cycle, cell proliferation and apoptosis. Mutations or low expression in certain TSGs, such as PTP (protein tyrosine phosphatase), should bring the phosphorylation/dephosphorylation ratio out of balance [80], [81].

Cancer is a disease of aberrant signal transduction. In the functioning biological system, tumor suppressors keep the signaling cascades in balance, such as for the TGF-βR II and Smad2/3/4 in TGFβ signaling pathways [59], [60] and ptch1 protein (patched 1) in hedgehog pathway [82].

In addition, some molecular complex and enzyme activity should be noticed. The SWI/SNF complex, which contains a subunit from the BAF family, mediated chromatin remodeling in cell differentiation, proliferation and DNA repair. Several components of the SWI/SNF complex, such as BAF47, function as tumor suppressors, and BRM and BRG1 act as putative tumor suppressors, which is evidenced by frequently loss of heterozygosity [83].

### Analysis of the KEGG pathways in the total optimal feature set

Nine KEGG pathway terms in the *OS*, were hsa04115 (p53 signaling pathway), hsa00100 (steroid biosynthesis), hsa05213 (endometrial cancer), hsa05216 (thyroid cancer), hsa05218 (melanoma), hsa05219 (bladder cancer), hsa05220 (chronic myeloid leukemia), hsa05221 (acute myeloid leukemia) and hsa05223 (non-small cell lung cancer). As discussed above, p53 participates in cell death regulation and cell cycle control as a key central element. Aberrant genetic inactivation or diminished expression of p53 was found in the most of KEGG cancers terms. In addition to Rb in bladder cancer and chronic myeloid leukemia [7], , abnormal PTEN was also found in thyroid cancer and endometrial cancer [7], . In melanoma, chronic myeloid leukemia and non-small cell lung cancer patients, there is reported silence or suppression of ink4a/arf leading to cell cycle disorder and sustained cellular proliferation [7], . Steroids and steroid metabolism have markedly influenced in some cancer types, such as breast cancer and prostate cancer, which may mediate the apoptosis network [87], [88].

Unlike oncogenes, TSGs act as guardians regulating the network of cell cycle and apoptosis factors involved in controlling cell fate. Furthermore, maintaining genomic stability and balanced cell adhesion demand that the TSGs perform normal physiological functions.

### Analysis of candidate tumor suppressors predicted based on optimal features

We try to predict the novel TSGs based on features in the total optimal feature set, *i.e.*, the key functions that defines tumor suppressor. For each ‘negative gene’, we counted the number of key tumor suppressor functions that it was annotated onto. The genes with great number of key tumor suppressor functions were considered as candidate tumor suppressors, since they shared similar functions with the known tumor suppressors. Since oncogene and tumor suppressor are two sides of a coin, their functions are difficult to distinguish. To better prioritize candidate tumor suppressor, we removed the 330 oncogenes from oncogene family of GSEA MSigDB (Molecular Signatures DATAbase, http://www.broadinstitute.org/gsea/msigdb/gene_families.jsp) and 251 oncogenes from HGNC (HUGO Gene Nomenclature Committee, http://www.genenames.org/) with the oncogene as the keyword. MSigDB is an online database, which collected annotated genes sets for GSEA analyze and categorize genes into gene family to provide a functional overview. HGNC is a collection of unique symbols and names for genes, ncRNA genes and pseudogenes. Subsequently, the overlap genes between these genes and the ‘negative genes’ were filtered out, 17,553 ensembl protein IDs remain in the end, which are available in Table S5.

Our study performs the gene enrichment and pathway enrichment analysis, providing a support to identify novel tumor suppressor in these features and pathways. In **Table 3**, we revealed a list of novel tumor suppressor genes, which shared at least 293 key annotations with known tumor suppressors. It has been demonstrated part of them play suppressive roles in tumorigenesis and more genes need verification by functional evidence and a larger clinical pathological characteristics data set. There are many tumor suppress genes proved partly, such as EP300 [89]−[91], GATA4 [92], ESR1 [93] and NFKBIA [94], [95], which still need a large clinic data validation and functional research.

**Table 3:** Top forty putative tumor suppressors based on features in the total optimal feature set.

Ensembl ID	Number of key tumorsuppressor functions^a^	Gene symbol
ENSP00000297261	353	SHH
ENSP00000324806	353	GSK3B
ENSP00000389184	345	MARK2
ENSP00000264657	338	STAT3
ENSP00000355069	338	PAX2
ENSP00000293549	337	WNT1
ENSP00000353483	331	MAPK8
ENSP00000263253	331	EP300
ENSP00000218894	327	SUPT20H
ENSP00000328181	327	NOG
ENSP00000228872	327	CDKN1B
ENSP00000338548	325	FGF1
ENSP00000250003	322	MYOD1
ENSP00000206249	322	ESR1
ENSP00000245451	321	BMP4
ENSP00000352514	317	RUNX2
ENSP00000348986	316	INS-IGF2
ENSP00000263025	315	MAPK3
ENSP00000354558	313	MTOR
ENSP00000363822	311	AR
ENSP00000361066	310	NCOA3
ENSP00000339004	309	FOXG1
ENSP00000320604	309	FAXDC2
ENSP00000338018	308	HIF1A
ENSP00000278385	308	CD44
ENSP00000216797	306	NFKBIA
ENSP00000222330	304	GSK3A
ENSP00000255465	304	CCNA1
ENSP00000222726	303	HOXA5
ENSP00000334458	303	GATA4
ENSP00000264498	303	FGF2
ENSP00000323588	302	SOX2
ENSP00000392858	299	TNF
ENSP00000302665	299	IGF1
ENSP00000338297	298	-
ENSP00000362649	297	HDAC1
ENSP00000318977	297	GEN1
ENSP00000343745	296	DICER1
ENSP00000265165	294	LEF1
ENSP00000415481	293	PROM1

a The value in this column is the number of features in the total optimal feature set whose values are greater than –log_10_(0.05).

Glycogen synthase kinase 3 beta (GSK3β) belongs to the glycogen synthase kinase subfamily. GSK2β regulated Wnt signaling and PI3K/Akt pathway negatively, which play key roles in cell cycle, anti-apoptosis and invasion [96], [97]. It has been identified suppression of GSK3β in many tumor types including, oral squamous cell carcinoma (OSCC), lung cancer, cutaneous squamous cell carcinoma and esophageal carcinoma [98]−[101]. Inhibition of constitutively active GSK3β leads to epithelial-mesenchymal transition (EMT) transition during tumorigenesis [102]. In vitro, GSK3β play a negative regulator of myeloid cell leukemia-1(Mcl-1), which has anti-apoptotic function and is correlated to the poor prognosis of breast cancer patients [98], [103], [104]. Although there are some controversial reports, GSK3β is a putative tumor suppressor and need more studies [105], [106].

Homeobox A5 (HOXA5) is belonging to a DNA-binding transcription factor family, homeobox genes cluster A, and regulates organism gene expression, adult differentiation and embryonic development in organism. It has been observed a frequently increased methylation of the HOXA5 promoter region in various tumor tissues [107]−[109] and is related to decreased expression [107], [110]. In addition, HOXA5 up-regulates p53 transcription through binding to a target element in its promoter [111]. These evidences document that HOXA5 is a putative tumor suppressor for tumorigenesis. But it still warrants further functional studies that how HOXA5 suppress tumorigenesis in animal model and in clinic.

Holliday Junction 5′ Flap Endonuclease, previous named gen endonuclease homolog 1 (GEN1) is an enzyme, evolved in Holliday junctions (HJs) formation during homologous recombination and DNA repair. The activity of Yen1, the ortholog of GEN1, is inhibited by phosphorylation events in the G1/S transition, keep inactive through S-phase and G2, and activated by dephosphorylation at the later stages of mitosis [112], [113]. Similarly, in the early stages of the cell cycle, GEN1 is excluded from the nucleus, and access chromatin and HJs [113]. GEN1 participates in some specific features: cell cycle, DNA repair and phosphorylation/dephosphorylation, which involved in many tumor suppressors. In Bloom’s syndrome cells, depletion of GEN1 results in severe chromosome abnormalities [114]. It has been identified rare recessive at-risk alleles of GEN1 in breast cancer by Ekatherina Sh [115]−[117], and two somatic frameshift mutations in breast cancer cell lines and primary tumors through exome sequencing [114]. Above all, GEN1 is a novel tumor suppressor akin to some other DNA repair genes, BRCA1 and BRCA2 in breast cancer, although there is rare study prove GEN1 make a high-appreciable contribution to breast cancer. In future study, it would be focus on the methylation or LOH level and anti-tumorigenesis mechanism to explore function of GEN1.

Besides these genes discussed above, our study reveals more novel candidate tumor suppressors including SHH, STAT3, SUPT20H and GSK3A, which are highlighted and need more focus and research in future cancer research.

## Conclusions

This study summarizes the enrichment analysis of TSGs. The features of the GO and KEGG pathway enrichment scores were used to encode the investigated genes and some feature selection methods were employed to analyze these features. The analysis of the 708 GO terms and 9 KEGG pathways implies that they are strongly related to the determination of TSGs. We hope that effective methods based on these GO terms and KEGG pathways can be built to identify TSGs.

## Supporting Information

Table S1 List of 615 tumor suppressor genes.
(PDF)

Table S2 List of the MaxRel features lists and mRMR features lists obtained by mRMR method for each dataset.
(PDF)

Table S3 List of the SNs, SPs, ACCs and MCCs obtained by IFS and Dagging for each dataset *S_i_*.
(PDF)

Table S4 List of 717 Features in the final optimal feature set.
(PDF)

Table S5 List of the novel tumor suppressors predicted based on features in the total optimal feature set.
(PDF)
