# Supplementary material for: Analysis of Tumor Suppressor Genes Based on Gene Ontology and the KEGG Pathway
Source: PLoS One. 2014 Sep 10;9(9):e107202. doi: 10.1371/journal.pone.0107202 (PMC4160198; doi:10.1371/journal.pone.0107202)
Supplement: Table S1 — List of 615 tumor suppressor genes. (PDF) [file pone.0107202.s001.pdf]

Table S1. 615 tumor suppressor genes

ENSP00000006724	ENSP00000013034	ENSP00000014914	ENSP00000020926
ENSP00000025008	ENSP00000028008	ENSP00000161559	ENSP00000172229
ENSP00000182290	ENSP00000211998	ENSP00000212355	ENSP00000215743
ENSP00000216101	ENSP00000216115	ENSP00000216218	ENSP00000216225
ENSP00000216832	ENSP00000219476	ENSP00000220562	ENSP00000220772
ENSP00000221132	ENSP00000221138	ENSP00000221265	ENSP00000221496
ENSP00000221930	ENSP00000222329	ENSP00000222374	ENSP00000222543
ENSP00000222574	ENSP00000222693	ENSP00000225655	ENSP00000226574
ENSP00000227155	ENSP00000228437	ENSP00000228865	ENSP00000231004
ENSP00000231749	ENSP00000231751	ENSP00000231790	ENSP00000232496
ENSP00000232501	ENSP00000232508	ENSP00000233057	ENSP00000233146
ENSP00000233607	ENSP00000233668	ENSP00000233813	ENSP00000235372
ENSP00000237289	ENSP00000238044	ENSP00000238699	ENSP00000238918
ENSP00000239938	ENSP00000240139	ENSP00000241463	ENSP00000242480
ENSP00000242728	ENSP00000243050	ENSP00000244741	ENSP00000245414
ENSP00000246802	ENSP00000247470	ENSP00000248444	ENSP00000248594
ENSP00000248673	ENSP00000248935	ENSP00000249373	ENSP00000249396
ENSP00000249750	ENSP00000249910	ENSP00000250457	ENSP00000251630
ENSP00000251822	ENSP00000251968	ENSP00000252506	ENSP00000252542
ENSP00000253332	ENSP00000253339	ENSP00000253754	ENSP00000254079
ENSP00000254227	ENSP00000254521	ENSP00000255688	ENSP00000255695
ENSP00000256474	ENSP00000256925	ENSP00000256951	ENSP00000257430
ENSP00000257497	ENSP00000257700	ENSP00000257831	ENSP00000257879
ENSP00000257940	ENSP00000258080	ENSP00000258439	ENSP00000258526
ENSP00000258613	ENSP00000259371	ENSP00000259748	ENSP00000260264
ENSP00000260283	ENSP00000260356	ENSP00000260526	ENSP00000260766
ENSP00000260947	ENSP00000260985	ENSP00000261017	ENSP00000261023
ENSP00000261584	ENSP00000261692	ENSP00000261729	ENSP00000261769
ENSP00000261908	ENSP00000262096	ENSP00000262102	ENSP00000262133
ENSP00000262160	ENSP00000262304	ENSP00000262320	ENSP00000262367
ENSP00000262445	ENSP00000262450	ENSP00000262613	ENSP00000262662
ENSP00000262752	ENSP00000262903	ENSP00000262992	ENSP00000263083
ENSP00000263121	ENSP00000263461	ENSP00000263867	ENSP00000263895
ENSP00000263934	ENSP00000264010	ENSP00000264265	ENSP00000264360
ENSP00000264414	ENSP00000264634	ENSP00000264677	ENSP00000264731
ENSP00000264775	ENSP00000264932	ENSP00000264951	ENSP00000265437
ENSP00000265773	ENSP00000265801	ENSP00000265849	ENSP00000266022
ENSP00000266066	ENSP00000266070	ENSP00000266085	ENSP00000266427
ENSP00000266659	ENSP00000266744	ENSP00000267163	ENSP00000267569
ENSP00000268058	ENSP00000268489	ENSP00000268603	ENSP00000268613
ENSP00000268638	ENSP00000268679	ENSP00000268720	ENSP00000268864
ENSP00000269305	ENSP00000269593	ENSP00000270162	ENSP00000271638

ENSP00000272102	ENSP00000272233	ENSP00000272771	ENSP00000273179
ENSP00000273317	ENSP00000273590	ENSP00000273739	ENSP00000274289
ENSP00000274565	ENSP00000275815	ENSP00000276282	ENSP00000276297
ENSP00000276420	ENSP00000276431	ENSP00000276893	ENSP00000276925
ENSP00000276927	ENSP00000277165	ENSP00000277508	ENSP00000277541
ENSP00000277632	ENSP00000278616	ENSP00000278903	ENSP00000278916
ENSP00000279101	ENSP00000279146	ENSP00000279230	ENSP00000279488
ENSP00000280154	ENSP00000281708	ENSP00000282026	ENSP00000282441
ENSP00000282561	ENSP00000282849	ENSP00000282884	ENSP00000282928
ENSP00000284240	ENSP00000284440	ENSP00000284694	ENSP00000285071
ENSP00000285407	ENSP00000285930	ENSP00000286574	ENSP00000287598
ENSP00000289749	ENSP00000290100	ENSP00000290295	ENSP00000290551
ENSP00000290921	ENSP00000292123	ENSP00000292641	ENSP00000292928
ENSP00000293230	ENSP00000293288	ENSP00000293379	ENSP00000294984
ENSP00000295379	ENSP00000295666	ENSP00000295797	ENSP00000296029
ENSP00000296318	ENSP00000296522	ENSP00000297439	ENSP00000297788
ENSP00000297954	ENSP00000298231	ENSP00000298552	ENSP00000298687
ENSP00000298743	ENSP00000298772	ENSP00000299259	ENSP00000299727
ENSP00000300408	ENSP00000301244	ENSP00000301264	ENSP00000301488
ENSP00000301838	ENSP00000301921	ENSP00000301924	ENSP00000302625
ENSP00000302630	ENSP00000302967	ENSP00000303507	ENSP00000303766
ENSP00000304229	ENSP00000304360	ENSP00000304956	ENSP00000305244
ENSP00000306522	ENSP00000306866	ENSP00000307132	ENSP00000307183
ENSP00000307292	ENSP00000307617	ENSP00000307859	ENSP00000308597
ENSP00000308928	ENSP00000308944	ENSP00000309148	ENSP00000309710
ENSP00000309913	ENSP00000310127	ENSP00000310260	ENSP00000310841
ENSP00000310880	ENSP00000311113	ENSP00000311313	ENSP00000311344
ENSP00000311469	ENSP00000311746	ENSP00000311747	ENSP00000311825
ENSP00000311857	ENSP00000313158	ENSP00000313391	ENSP00000313851
ENSP00000313983	ENSP00000314080	ENSP00000314129	ENSP00000314910
ENSP00000315130	ENSP00000315955	ENSP00000316203	ENSP00000316779
ENSP00000318902	ENSP00000319412	ENSP00000319678	ENSP00000319977
ENSP00000320337	ENSP00000320485	ENSP00000320557	ENSP00000320566
ENSP00000321674	ENSP00000321927	ENSP00000322142	ENSP00000322568
ENSP00000322804	ENSP00000323065	ENSP00000323580	ENSP00000323816
ENSP00000324191	ENSP00000324404	ENSP00000324422	ENSP00000324856
ENSP00000325526	ENSP00000325836	ENSP00000326031	ENSP00000326563
ENSP00000326581	ENSP00000326759	ENSP00000326804	ENSP00000327048
ENSP00000328088	ENSP00000328160	ENSP00000328352	ENSP00000328364
ENSP00000328777	ENSP00000329029	ENSP00000329097	ENSP00000329102
ENSP00000329117	ENSP00000329418	ENSP00000329794	ENSP00000329797
ENSP00000330054	ENSP00000330341	ENSP00000330862	ENSP00000331087
ENSP00000331152	ENSP00000331327	ENSP00000331504	ENSP00000331602
ENSP00000332171	ENSP00000332296	ENSP00000332353	ENSP00000332643

ENSP00000332737	ENSP00000332973	ENSP00000333122	ENSP00000334329
ENSP00000334611	ENSP00000335044	ENSP00000336701	ENSP00000336775
ENSP00000337056	ENSP00000337088	ENSP00000337127	ENSP00000337464
ENSP00000337946	ENSP00000338157	ENSP00000338785	ENSP00000338983
ENSP00000339191	ENSP00000339527	ENSP00000339587	ENSP00000339692
ENSP00000339906	ENSP00000340093	ENSP00000340118	ENSP00000340278
ENSP00000340507	ENSP00000340797	ENSP00000340989	ENSP00000341138
ENSP00000341208	ENSP00000341551	ENSP00000341730	ENSP00000341957
ENSP00000342087	ENSP00000342235	ENSP00000342656	ENSP00000342924
ENSP00000343001	ENSP00000343023	ENSP00000343054	ENSP00000343126
ENSP00000343477	ENSP00000343619	ENSP00000343741	ENSP00000343925
ENSP00000344215	ENSP00000344666	ENSP00000344668	ENSP00000345064
ENSP00000345281	ENSP00000345571	ENSP00000345731	ENSP00000345873
ENSP00000346440	ENSP00000346810	ENSP00000346927	ENSP00000347232
ENSP00000347443	ENSP00000348300	ENSP00000348611	ENSP00000348786
ENSP00000348812	ENSP00000348827	ENSP00000349156	ENSP00000349213
ENSP00000349259	ENSP00000349437	ENSP00000349486	ENSP00000349496
ENSP00000349547	ENSP00000349577	ENSP00000349727	ENSP00000350283
ENSP00000350720	ENSP00000350785	ENSP00000350844	ENSP00000350896
ENSP00000350937	ENSP00000351015	ENSP00000351273	ENSP00000351363
ENSP00000351410	ENSP00000351539	ENSP00000351885	ENSP00000351905
ENSP00000352270	ENSP00000352798	ENSP00000353059	ENSP00000353656
ENSP00000353731	ENSP00000353735	ENSP00000354033	ENSP00000354040
ENSP00000354376	ENSP00000354734	ENSP00000354778	ENSP00000354822
ENSP00000355153	ENSP00000355231	ENSP00000355518	ENSP00000355601
ENSP00000355865	ENSP00000356162	ENSP00000356278	ENSP00000356405
ENSP00000356437	ENSP00000356473	ENSP00000356530	ENSP00000356630
ENSP00000356737	ENSP00000356954	ENSP00000357106	ENSP00000357112
ENSP00000357122	ENSP00000357209	ENSP00000357392	ENSP00000357615
ENSP00000357697	ENSP00000357905	ENSP00000357980	ENSP00000358092
ENSP00000358120	ENSP00000358222	ENSP00000358283	ENSP00000358323
ENSP00000358404	ENSP00000358795	ENSP00000358918	ENSP00000359131
ENSP00000359424	ENSP00000359645	ENSP00000359799	ENSP00000359854
ENSP00000360020	ENSP00000360329	ENSP00000360365	ENSP00000360472
ENSP00000361021	ENSP00000361266	ENSP00000361759	ENSP00000361894
ENSP00000361895	ENSP00000362010	ENSP00000362410	ENSP00000362637
ENSP00000362762	ENSP00000362768	ENSP00000362817	ENSP00000363081
ENSP00000363128	ENSP00000363377	ENSP00000363398	ENSP00000363763
ENSP00000363804	ENSP00000363851	ENSP00000363880	ENSP00000363897
ENSP00000364013	ENSP00000364094	ENSP00000364252	ENSP00000364589
ENSP00000364649	ENSP00000364699	ENSP00000364839	ENSP00000364898
ENSP00000364929	ENSP00000365380	ENSP00000365439	ENSP00000366306
ENSP00000366307	ENSP00000366702	ENSP00000366800	ENSP00000366915
ENSP00000366923	ENSP00000367202	ENSP00000367446	ENSP00000367545

ENSP00000367794	ENSP00000367910	ENSP00000367972	ENSP00000368104
ENSP00000368314	ENSP00000368401	ENSP00000368684	ENSP00000368699
ENSP00000368880	ENSP00000368924	ENSP00000369038	ENSP00000369351
ENSP00000369427	ENSP00000369442	ENSP00000369497	ENSP00000369519
ENSP00000370003	ENSP00000370253	ENSP00000370256	ENSP00000370421
ENSP00000370473	ENSP00000370968	ENSP00000371070	ENSP00000371734
ENSP00000372023	ENSP00000372035	ENSP00000372191	ENSP00000372221
ENSP00000372295	ENSP00000373477	ENSP00000374135	ENSP00000376436
ENSP00000376504	ENSP00000376822	ENSP00000376903	ENSP00000377696
ENSP00000377823	ENSP00000377854	ENSP00000377862	ENSP00000378180
ENSP00000378191	ENSP00000378349	ENSP00000379183	ENSP00000379364
ENSP00000379823	ENSP00000379946	ENSP00000380024	ENSP00000380066
ENSP00000380378	ENSP00000380942	ENSP00000380969	ENSP00000381425
ENSP00000381821	ENSP00000382004	ENSP00000382562	ENSP00000382840
ENSP00000384442	ENSP00000385720	ENSP00000386050	ENSP00000386096
ENSP00000386161	ENSP00000386165	ENSP00000386227	ENSP00000386733
ENSP00000389140	ENSP00000391397	ENSP00000391592	ENSP00000392553
ENSP00000392985	ENSP00000395465	ENSP00000396052	ENSP00000397435
ENSP00000398410	ENSP00000400010	ENSP00000400175	ENSP00000401435
ENSP00000401632	ENSP00000403397	ENSP00000405176	ENSP00000405738
ENSP00000406022	ENSP00000406229	ENSP00000406490	ENSP00000408176
ENSP00000409016	ENSP00000413720	ENSP00000414598	ENSP00000416330
ENSP00000417132	ENSP00000417659	ENSP00000417864	ENSP00000418735
ENSP00000419000	ENSP00000419449	ENSP00000419923	
