# Supplementary material for: Analysis of Tumor Suppressor Genes Based on Gene Ontology and the KEGG Pathway
Source: PLoS One. 2014 Sep 10;9(9):e107202. doi: 10.1371/journal.pone.0107202 (PMC4160198; doi:10.1371/journal.pone.0107202)
Supplement: Table S2 — List of the MaxRel features lists and mRMR features lists obtained by mRMR method for each dataset. (PDF) [file pone.0107202.s002.pdf]

Table S2. The MaxRel features lists and mRMR features lists obtained by mRMR method for each dataset.

(1) Dataset S_1

a) MaxRel features list

Rank	Feature name
1	GO:0051717
2	GO:0033032
3	GO:0051800
4	GO:0090071
5	GO:0031658
6	GO:2000808
7	GO:0097107
8	GO:0032535
9	hsa05219
10	GO:0051146
11	hsa05213
12	hsa05216
13	GO:0016314
14	GO:0007050
15	GO:0033596
16	hsa05215
17	hsa05220
18	GO:0032286
19	GO:0044430
20	GO:0045792
21	GO:0090394
22	hsa05200
23	hsa04115
24	GO:0002902
25	GO:0030336
26	GO:0004861
27	GO:0060179
28	hsa05218
29	hsa05212
30	GO:0032794
31	hsa05223
32	GO:0050821
33	hsa05221
34	hsa05210
35	GO:0060074
36	GO:0007092
37	GO:0031571
38	GO:0035022

39	hsa05214
40	GO:0042058
41	GO:0090398
42	GO:0031575
43	GO:0060024
44	GO:0031647
45	GO:0071681
46	GO:0006407
47	GO:0008285
48	GO:0045475
49	GO:0033561
50	GO:0055105
51	GO:0070141
52	GO:2000008
53	GO:0010165
54	GO:0043066
55	GO:0071850
56	GO:0050680
57	GO:0055100
58	GO:0071158
59	GO:0043220
60	GO:0071479
61	GO:0071456
62	GO:0001952
63	GO:0001047
64	GO:0008629
65	GO:0042127
66	GO:0045667
67	GO:0033601
68	GO:0019002
69	GO:0006309
70	GO:0061002
71	GO:0043281
72	GO:0032880
73	GO:0042771
74	GO:0007265
75	GO:0051492
76	GO:0090403
77	GO:0043542
78	GO:0035802
79	GO:0003156
80	GO:0071930
81	GO:0031134
82	GO:0048546
83	GO:0016342

84	GO:0051726
85	GO:0090230
86	GO:0000279
87	GO:0006469
88	GO:0051894
89	GO:0019903
90	GO:0051895
91	hsa05222
92	GO:0001570
93	GO:0019912
94	GO:2000278
95	GO:0097105
96	GO:0048745
97	GO:0048262
98	GO:0030997
99	GO:0060479
100	GO:0035112
101	GO:0006921
102	GO:0042802
103	GO:0048714
104	GO:0010997
105	GO:0008630
106	GO:0005515
107	hsa05166
108	GO:0001836
109	GO:0048146
110	hsa04012
111	GO:0072079
112	GO:0045736
113	GO:0030858
114	GO:0032228
115	GO:0034750
116	GO:0050808
117	GO:0007420
118	GO:0042326
119	GO:0071364
120	GO:0007356
121	GO:2001076
122	GO:0030308
123	GO:0051257
124	GO:0007060
125	GO:0043060
126	GO:0070215
127	GO:0060749
128	GO:0002039

129	GO:0010975
130	GO:0014010
131	GO:0001701
132	GO:0008134
133	GO:0072166
134	GO:0008634
135	GO:0045892
136	GO:0034088
137	GO:0016310
138	GO:0051898
139	GO:0007406
140	GO:0033598
141	GO:0043550
142	GO:0072302
143	GO:0045930
144	GO:0047710
145	GO:0015964
146	GO:0000186
147	GO:0033673
148	GO:0035189
149	hsa04520
150	GO:0048147
151	GO:0006917
152	hsa04320
153	GO:0033088
154	GO:0045950
155	GO:0048015
156	GO:0008283
157	GO:0051893
158	GO:0060421
159	GO:0032390
160	GO:0005712
161	GO:0008013
162	GO:0043234
163	GO:0022408
164	GO:0019901
165	GO:0007417
166	GO:0005924
167	GO:0048011
168	GO:0044334
169	GO:0010909
170	GO:0070369
171	GO:0051097
172	GO:0043296
173	GO:0071285

174	GO:0060770
175	GO:0006978
176	hsa05211
177	GO:0031100
178	GO:0005737
179	GO:0001085
180	GO:0007569
181	GO:0019899
182	GO:0051412
183	GO:0060709
184	GO:2001047
185	GO:0045295
186	GO:0051668
187	GO:0048853
188	GO:0003690
189	GO:0043409
190	GO:0030097
191	GO:0051153
192	GO:0048742
193	GO:0043065
194	GO:0008633
195	GO:2000060
196	GO:0043508
197	GO:0019538
198	GO:0007281
199	GO:0002052
200	GO:0032355
201	GO:0043280
202	GO:0021542
203	GO:0001889
204	GO:0033326
205	GO:0033077
206	GO:0071922
207	GO:0043535
208	GO:0004438
209	GO:0010629
210	GO:0051318
211	GO:0009898
212	GO:0061032
213	GO:0010628
214	GO:0002053
215	GO:0035264
216	GO:0000122
217	GO:0004716
218	GO:0008543

219	GO:0034644
220	GO:0003714
221	GO:0036023
222	GO:0043497
223	GO:0032956
224	GO:0001934
225	GO:0060644
226	GO:0035033
227	GO:0048617
228	GO:0031069
229	GO:0016604
230	GO:0043433
231	GO:0045765
232	GO:0046777
233	GO:0031670
234	GO:0060916
235	GO:0007173
236	GO:0016328
237	GO:0030325
238	GO:0003908
239	GO:0055096
240	GO:0001933
241	GO:0010424
242	GO:0034747
243	GO:0061324
244	GO:0045768
245	GO:0072054
246	GO:0072053
247	GO:0001102
248	GO:0043524
249	GO:0060736
250	GO:0060662
251	GO:0072033
252	GO:0042153
253	GO:0031065
254	GO:0090399
255	GO:0006468
256	GO:0042551
257	GO:0045668
258	GO:0060789
259	GO:0048538
260	GO:0048565
261	GO:0008637
262	GO:0005072
263	GO:0045893

264	GO:0048145
265	GO:2000134
266	GO:2000653
267	GO:0044029
268	GO:0000790
269	GO:2000379
270	GO:2000054
271	GO:2001220
272	GO:0044346
273	GO:0090096
274	GO:0050679
275	GO:0000739
276	GO:0000733
277	GO:0001829
278	GO:0034613
279	GO:0022405
280	GO:0072332
281	GO:0051444
282	GO:0045931
283	GO:0030857
284	GO:2000195
285	GO:0007435
286	GO:0030335
287	GO:0070245
288	GO:0030879
289	GO:0016605
290	GO:0033235
291	GO:0030900
292	GO:0003682
293	GO:0035021
294	GO:0046022
295	GO:0033689
296	GO:0007346
297	GO:0032091
298	GO:0030856
299	GO:0007507
300	GO:0014067
301	GO:0007416
302	GO:0005913
303	GO:0006919
304	GO:0071459
305	GO:0046621
306	GO:0030511
307	GO:0046674
308	GO:0005757

309	GO:0032976
310	GO:0048733
311	GO:0001953
312	GO:0046825
313	GO:0008284
314	GO:0034742
315	GO:0060769
316	GO:2000270
317	GO:0071481
318	GO:0043923
319	GO:0032318
320	GO:0010332
321	GO:0045597
322	GO:0008286
323	GO:0045737
324	GO:0060997
325	GO:0031625
326	GO:0071157
327	GO:0043517
328	GO:0007369
329	GO:0046546
330	GO:0032204
331	GO:0043666
332	hsa04722
333	GO:0010942
334	GO:0004672
335	GO:0001776
336	GO:0043392
337	GO:0007169
338	GO:0010468
339	GO:0045685
340	GO:0021897
341	GO:0031668
342	GO:0000165
343	GO:0030307
344	GO:0009314
345	GO:0016772
346	GO:0090200
347	GO:0032993
348	GO:0001706
349	GO:0032461
350	GO:0048589
351	GO:0033138
352	GO:0021955
353	GO:0004713

354	GO:0000785
355	GO:0042493
356	GO:0038028
357	GO:0006306
358	GO:0000080
359	GO:0019904
360	GO:0035265
361	GO:0004714
362	GO:0030235
363	GO:0070557
364	GO:0038085
365	GO:0060923
366	GO:0045732
367	GO:0007422
368	GO:0002326
369	GO:0043627
370	GO:0060529
371	GO:0043154
372	GO:0051000
373	GO:0001954
374	GO:0030695
375	GO:0060177
376	GO:0030690
377	GO:0072112
378	GO:0010243
379	GO:0030521
380	GO:0000320
381	GO:0060439
382	GO:0000307
383	hsa04010
384	GO:0030030
385	GO:2000617
386	GO:2000620
387	GO:0070512
388	GO:2000271
389	GO:2000052
390	GO:0090246
391	GO:2000080
392	GO:0044345
393	GO:0090051
394	GO:0007398
395	GO:2000041
396	GO:0040008
397	GO:0051385
398	GO:0030850

399	GO:0050678
400	GO:0043523
401	GO:0031648
402	GO:0050681
403	GO:0016601
404	GO:0032007
405	GO:0045595
406	GO:0021747
407	GO:0048568
408	GO:0001841
409	GO:0070372
410	GO:0007270
411	GO:0005113
412	GO:0005938
413	GO:0031659
414	GO:0046983
415	GO:0033687
416	GO:0060484
417	GO:0007090
418	GO:0045445
419	GO:0060742
420	GO:0006974
421	GO:0072284
422	GO:0051573
423	GO:0072134
424	GO:0032848
425	GO:0048753
426	GO:0043375
427	GO:0048743
428	GO:2001234
429	GO:0022898
430	GO:0006808
431	GO:0014042
432	GO:0060433
433	GO:0042663
434	GO:0030177
435	GO:0031436
436	GO:0000792
437	GO:0005667
438	GO:0051902
439	GO:0042770
440	GO:0018105
441	GO:0061198
442	GO:0016477
443	GO:0046329

444	GO:0045180
445	GO:0031226
446	GO:0006346
447	GO:0043406
448	GO:0072001
449	GO:0071899
450	GO:0010719
451	GO:0043616
452	GO:0023014
453	GO:0051721
454	GO:0046889
455	GO:0010033
456	GO:0045786
457	GO:0060209
458	GO:0001042
459	GO:0072277
460	GO:0051171
461	GO:0042483
462	GO:0048103
463	GO:0035441
464	hsa04150
465	GO:0045600
466	GO:0060440
467	GO:0042992
468	GO:0071407
469	GO:0043525
470	GO:0009987
471	GO:0009887
472	GO:0007595
473	GO:0048320
474	GO:0001835
475	GO:0045945
476	GO:0043006
477	GO:0046851
478	GO:0046902
479	GO:0009880
480	GO:0033593
481	GO:0031619
482	GO:2000342
483	GO:0034349
484	GO:0019900
485	GO:0008624
486	GO:0030616
487	GO:0046982
488	GO:0006461

489	GO:0007403
490	GO:0072133
491	GO:0030334
492	GO:0045165
493	GO:0090343
494	GO:0046600
495	GO:0047485
496	GO:0071398
497	GO:0072182
498	GO:0043009
499	GO:0070602
500	GO:0070491

b) mRMR features list

Rank	Feature name
1	GO:0051717
2	GO:0010424
3	GO:0032794
4	GO:0007356
5	GO:0051721
6	GO:0047710
7	GO:0051894
8	GO:0033673
9	GO:0043508
10	GO:0035022
11	GO:0033596
12	GO:0090403
13	GO:0042058
14	GO:0035694
15	GO:0050808
16	GO:0051153
17	GO:0071364
18	GO:0006307
19	GO:0033235
20	GO:0048745
21	GO:0051492
22	GO:0006309
23	GO:0033601
24	GO:0042271
25	GO:0016601
26	GO:0043666
27	GO:0010165
28	GO:0021612
29	GO:0006921

30	GO:0042551
31	GO:0043281
32	GO:0045792
33	GO:0071481
34	GO:0000115
35	GO:0033326
36	GO:0051895
37	GO:0032300
38	GO:0006407
39	GO:0031670
40	GO:0016514
41	GO:0015964
42	GO:0060997
43	GO:0030336
44	GO:0051097
45	GO:2000500
46	GO:0035802
47	GO:0030695
48	GO:0019538
49	GO:0055100
50	GO:0038085
51	GO:0051893
52	GO:0031647
53	GO:0000792
54	GO:0045884
55	GO:0004861
56	GO:0044430
57	GO:0035117
58	GO:0032461
59	GO:0003908
60	GO:0051146
61	GO:0001825
62	GO:0043015
63	GO:0030852
64	GO:0031571
65	GO:0032321
66	GO:0060024
67	GO:0043535
68	GO:0050508
69	GO:0050821
70	GO:0008634
71	GO:0042766
72	GO:0016339
73	GO:0051271
74	GO:0017148

75	GO:0031575
76	GO:0045814
77	GO:0071157
78	GO:0008340
79	GO:0032956
80	GO:0048546
81	GO:0055105
82	GO:2000119
83	GO:0051385
84	GO:0033561
85	GO:0006627
86	GO:0001952
87	GO:0000739
88	GO:2001076
89	GO:0007270
90	GO:0007162
91	GO:0006306
92	GO:0016310
93	GO:0008209
94	GO:2000041
95	GO:0032211
96	GO:0007185
97	GO:0048742
98	GO:0051257
99	GO:0010975
100	GO:0048846
101	GO:0048147
102	GO:0043296
103	GO:0034124
104	GO:0048014
105	GO:0043550
106	GO:0021599
107	GO:0010761
108	GO:0031461
109	GO:0097105
110	GO:0007422
111	GO:0032516
112	GO:0048714
113	GO:0097190
114	GO:0046718
115	GO:0055096
116	GO:0030030
117	GO:0090398
118	GO:0090343
119	GO:0005913

120	GO:0035907
121	GO:0072166
122	GO:0071565
123	GO:0048102
124	GO:0042518
125	GO:0016151
126	hsa05213
127	GO:0006346
128	GO:0015938
129	GO:0035375
130	GO:0008432
131	hsa04115
132	GO:0009826
133	GO:0071901
134	GO:2000463
135	GO:0046546
136	GO:0019789
137	GO:0043542
138	GO:0051444
139	GO:0001841
140	GO:0042711
141	GO:0034088
142	GO:0071279
143	GO:0051668
144	GO:0072332
145	GO:0019898
146	GO:0003714
147	GO:0007060
148	GO:0005099
149	GO:0003156
150	GO:2000134
151	GO:0032090
152	GO:0004716
153	GO:0051276
154	GO:2000484
155	GO:2000836
156	GO:0030857
157	GO:0010506
158	GO:0000791
159	GO:0043220
160	GO:0032007
161	GO:0060465
162	GO:0043409
163	GO:0042771
164	GO:0035329

165	GO:0002762
166	GO:0035414
167	GO:0021569
168	GO:0008064
169	GO:0070215
170	GO:0005068
171	GO:0060074
172	GO:2000310
173	GO:0060546
174	GO:2000271
175	GO:0071930
176	GO:2001241
177	GO:0007182
178	GO:0072302
179	GO:0031401
180	GO:0001953
181	GO:0000733
182	GO:0045667
183	GO:0008330
184	GO:0001835
185	GO:0090200
186	GO:0090219
187	GO:0060770
188	GO:0031226
189	GO:0016918
190	GO:0046825
191	GO:0090136
192	GO:0060480
193	GO:0030506
194	GO:0016314
195	GO:0032204
196	GO:0097162
197	GO:0032390
198	GO:0010907
199	GO:0009132
200	GO:0071681
201	GO:0010637
202	GO:0019941
203	GO:0004515
204	GO:0007050
205	GO:0042328
206	GO:0035305
207	GO:0070026
208	GO:0007265
209	GO:0043152

210	GO:2000052
211	GO:0051606
212	GO:0017053
213	GO:0048853
214	GO:0043497
215	GO:0007634
216	GO:0005884
217	GO:0033327
218	GO:0071398
219	GO:0061002
220	GO:0000093
221	GO:0051057
222	hsa05216
223	GO:0070245
224	GO:0046426
225	GO:0060421
226	GO:0002070
227	GO:0008327
228	GO:0044319
229	GO:0007090
230	GO:0007416
231	GO:0046580
232	GO:0048733
233	GO:0061428
234	GO:0071564
235	GO:0060709
236	GO:0001776
237	GO:0045950
238	GO:0005100
239	GO:0070507
240	GO:0090175
241	GO:0051865
242	GO:0046022
243	GO:0010389
244	GO:0032228
245	GO:0045837
246	GO:0007092
247	GO:0030850
248	GO:0061364
249	GO:0035441
250	GO:0034332
251	GO:0031065
252	GO:0000819
253	GO:0031235
254	GO:2000008

255	GO:0000188
256	GO:0010957
257	GO:0060876
258	GO:0002309
259	GO:0043060
260	GO:0046356
261	GO:0001570
262	GO:0051497
263	GO:0004721
264	GO:0016605
265	GO:0090246
266	GO:2001020
267	GO:0009987
268	GO:0045184
269	GO:0009008
270	hsa05221
271	GO:0021877
272	GO:0005501
273	GO:0060662
274	GO:0071158
275	GO:0050509
276	GO:0042992
277	GO:0070664
278	GO:0030264
279	GO:0051048
280	GO:0090394
281	GO:0072284
282	GO:0045859
283	GO:0043517
284	GO:0060340
285	GO:0035189
286	GO:2001022
287	GO:0017124
288	GO:0010717
289	GO:0070534
290	GO:0016538
291	GO:0050678
292	GO:0030100
293	GO:0006983
294	GO:0071679
295	GO:0043005
296	GO:0040008
297	GO:0072112
298	GO:0015267
299	GO:2000080

300	GO:0072384
301	GO:0071850
302	GO:0005712
303	GO:0021542
304	GO:0034614
305	GO:0014010
306	GO:0032862
307	GO:0050847
308	GO:0097119
309	GO:0001047
310	GO:0006930
311	GO:0004438
312	GO:0042069
313	GO:0045579
314	GO:0010839
315	GO:0007417
316	GO:0090116
317	GO:0001032
318	GO:0046329
319	GO:0001726
320	GO:0045569
321	GO:0000783
322	GO:0016342
323	GO:0043653
324	GO:0014037
325	GO:0010522
326	GO:0046621
327	GO:0032407
328	GO:0031668
329	GO:0044345
330	GO:0045090
331	GO:0033692
332	GO:0035021
333	GO:0060923
334	GO:0002902
335	GO:0050910
336	GO:0035791
337	GO:0032515
338	GO:0051262
339	GO:0071479
340	GO:0097057
341	GO:0001829
342	GO:0035024
343	GO:0045606
344	GO:0035265

345	GO:0008630
346	GO:2001047
347	GO:0050878
348	GO:0043522
349	GO:0045842
350	GO:0005912
351	hsa05219
352	GO:0007158
353	GO:0006417
354	GO:0006473
355	GO:0021889
356	GO:0021955
357	GO:0006349
358	GO:0070372
359	GO:0034644
360	GO:0022601
361	GO:0008385
362	GO:0070830
363	GO:0045899
364	GO:0008629
365	GO:0051017
366	GO:0061032
367	GO:0045475
368	GO:0000795
369	GO:0042325
370	GO:0021754
371	GO:0051414
372	GO:0043523
373	GO:0046473
374	GO:0042059
375	GO:0033088
376	GO:0000239
377	GO:2000270
378	GO:0005815
379	GO:0034983
380	GO:0034405
381	GO:0045295
382	GO:0045947
383	GO:0045322
384	GO:0071559
385	GO:0010507
386	GO:0001547
387	GO:0060179
388	GO:0016818
389	GO:0042552

390	GO:0008635
391	GO:0050798
392	GO:0045656
393	GO:0042787
394	GO:0051898
395	GO:0035726
396	GO:0045216
397	GO:0021759
398	GO:0070557
399	GO:0042117
400	GO:0042993
401	GO:0035098
402	GO:0043616
403	GO:0050795
404	GO:0070141
405	GO:0060242
406	GO:0003886
407	GO:2000195
408	GO:0032680
409	GO:0002039
410	GO:0035385
411	GO:0072079
412	GO:0031265
413	GO:0031256
414	GO:0042406
415	GO:0033554
416	GO:0005019
417	GO:0005113
418	GO:0046855
419	GO:0044257
420	GO:0004860
421	GO:0001836
422	GO:2000054
423	GO:0007088
424	GO:0035195
425	GO:0010997
426	GO:0045647
427	GO:0061010
428	GO:0033593
429	GO:0032320
430	GO:0071456
431	GO:0045296
432	GO:0035731
433	GO:0045088
434	GO:0032880

435	GO:0007497
436	GO:0043923
437	GO:0006464
438	GO:0050920
439	GO:0046851
440	GO:0035522
441	GO:0033032
442	GO:0005575
443	GO:0035330
444	GO:0030858
445	GO:0016235
446	GO:0060023
447	GO:0016571
448	GO:0005979
449	GO:0019211
450	GO:0032137
451	GO:0045737
452	GO:2000378
453	GO:0060748
454	GO:0090141
455	GO:0005154
456	GO:0032835
457	GO:2000637
458	GO:0033629
459	GO:0070997
460	GO:0030027
461	GO:0031134
462	GO:0008384
463	GO:0008595
464	GO:0035749
465	GO:0035033
466	GO:0072091
467	GO:0010792
468	GO:0014704
469	GO:0051974
470	GO:0019002
471	GO:0035732
472	GO:0002089
473	GO:0033687
474	GO:0005678
475	GO:0051400
476	GO:0044325
477	GO:0051800
478	GO:2000564
479	GO:0048729

480	GO:0051683
481	GO:0035331
482	GO:0014034
483	GO:0032389
484	GO:0060385
485	GO:0030859
486	GO:0048070
487	GO:0003712
488	GO:0051205
489	GO:0032938
490	GO:0007420
491	GO:0034329
492	GO:0010225
493	GO:0060213
494	GO:0042696
495	GO:0033146
496	GO:0043045
497	GO:0016328
498	hsa05220
499	GO:0001031
500	GO:0006266

(2) Dataset S_2

a) MaxRel features list

Rank	Feature name
1	hsa05219
2	hsa05216
3	GO:0051146
4	GO:2000808
5	GO:0032535
6	GO:0097107
7	GO:0033032
8	GO:0051717
9	GO:0031658
10	GO:0090071
11	GO:0051800
12	GO:0007050
13	GO:0007092
14	GO:0090403
15	GO:0071850
16	GO:0016314
17	GO:0071158
18	hsa05213
19	GO:0042127
20	GO:0071479

21	hsa05215
22	GO:0050821
23	GO:0030336
24	GO:0032286
25	GO:0004861
26	hsa05220
27	GO:0002902
28	GO:0031571
29	GO:0008285
30	hsa05210
31	GO:0090394
32	GO:0060179
33	GO:0031575
34	GO:0048147
35	GO:0042771
36	hsa05212
37	hsa05200
38	GO:0055105
39	GO:0051726
40	GO:0010165
41	hsa04115
42	GO:0060074
43	GO:0071456
44	hsa05218
45	hsa05221
46	GO:0044430
47	GO:0043066
48	GO:0090398
49	hsa05223
50	GO:0033596
51	GO:0071681
52	GO:0001952
53	GO:0070215
54	GO:0045475
55	GO:0050680
56	GO:0031647
57	GO:0007406
58	GO:0070141
59	GO:0030308
60	GO:0071930
61	GO:0051097
62	GO:0045792
63	GO:0006978
64	GO:0001836
65	GO:0014010

66	GO:0045736
67	GO:0048546
68	GO:0010997
69	GO:0060479
70	GO:0030997
71	GO:0048262
72	GO:0035112
73	GO:0008629
74	GO:0033561
75	GO:0033601
76	GO:0032880
77	GO:0006469
78	GO:0001047
79	GO:0032794
80	GO:0001701
81	GO:0071364
82	GO:0008134
83	GO:0000122
84	GO:0006407
85	GO:0007569
86	GO:0001829
87	GO:0070245
88	GO:2000008
89	GO:2000278
90	GO:0019912
91	GO:0019901
92	GO:0045930
93	GO:0035189
94	GO:0072079
95	GO:0034750
96	GO:0034613
97	GO:0008283
98	GO:0005072
99	GO:0033598
100	GO:0000279
101	GO:0090230
102	GO:0043433
103	GO:0045892
104	GO:0006917
105	GO:0042326
106	GO:0048145
107	GO:0030858
108	GO:0031134
109	GO:0042802
110	GO:0035022

111	GO:0003156
112	GO:0035802
113	GO:0048146
114	hsa05214
115	GO:0060749
116	GO:0010629
117	hsa05222
118	GO:0060024
119	GO:0007281
120	GO:0071157
121	GO:0070412
122	GO:0030511
123	GO:0034644
124	GO:0010909
125	GO:0044334
126	GO:0070369
127	GO:0003714
128	GO:0043535
129	GO:0019002
130	GO:0072054
131	GO:0072053
132	GO:0043234
133	GO:0045667
134	GO:0072332
135	GO:0045668
136	GO:0043281
137	GO:0033673
138	GO:0008630
139	GO:0036023
140	GO:0090200
141	GO:0061324
142	GO:0002326
143	GO:0033088
144	GO:0051412
145	GO:0031625
146	GO:0010628
147	GO:0006309
148	GO:0060789
149	GO:0048714
150	GO:0032355
151	GO:0001570
152	GO:0032390
153	GO:0005712
154	hsa05166
155	GO:0008013

156	GO:0002039
157	GO:0016342
158	GO:0043550
159	GO:0043060
160	GO:0007060
161	GO:0051257
162	GO:0045893
163	GO:0042058
164	GO:0051444
165	GO:0005737
166	GO:0007265
167	GO:0005924
168	GO:0045295
169	GO:0055100
170	GO:0001934
171	GO:0048538
172	GO:0008634
173	GO:0043508
174	GO:0031670
175	GO:0007417
176	GO:0045685
177	GO:0021897
178	GO:0019899
179	GO:0050681
180	GO:0060770
181	GO:0000790
182	GO:0010332
183	GO:0048589
184	GO:0061002
185	GO:0051895
186	GO:0035033
187	GO:0009314
188	GO:0001933
189	GO:0001085
190	GO:0043542
191	GO:0002053
192	GO:0030097
193	GO:0001889
194	GO:0016604
195	GO:0000739
196	GO:0000733
197	GO:0033077
198	GO:0071285
199	GO:0060769
200	GO:0043280

201	GO:0016310
202	GO:0001776
203	GO:0032318
204	GO:0022408
205	GO:0043627
206	GO:0005515
207	GO:0035021
208	GO:0008633
209	GO:0002309
210	GO:0072033
211	GO:0051894
212	GO:0032461
213	GO:0006921
214	GO:0007507
215	GO:2001047
216	GO:0060709
217	GO:0045765
218	GO:0048102
219	GO:0051898
220	GO:0033235
221	GO:0005667
222	GO:0045597
223	GO:2001220
224	GO:0044346
225	GO:0090096
226	GO:0043065
227	GO:0060916
228	GO:0034088
229	GO:0048733
230	GO:0031100
231	GO:0048745
232	GO:0042551
233	GO:0043220
234	GO:0015964
235	GO:0047710
236	GO:0034747
237	GO:0048742
238	GO:0051153
239	GO:0072166
240	GO:0003690
241	GO:0035264
242	GO:0001102
243	GO:0060644
244	GO:0008637
245	GO:0090051

246	GO:0033326
247	GO:0050679
248	GO:0007346
249	GO:0048617
250	GO:2001076
251	GO:0007356
252	GO:0071407
253	GO:0030856
254	GO:0007090
255	GO:0045950
256	GO:0014067
257	GO:2000054
258	GO:0048565
259	GO:0045445
260	GO:0046825
261	GO:0032228
262	GO:0010975
263	GO:0043154
264	GO:0042992
265	GO:0060923
266	GO:0070512
267	GO:2000617
268	GO:2000620
269	GO:0045595
270	GO:0031648
271	GO:0004438
272	GO:0071922
273	GO:0060736
274	GO:0045931
275	GO:0045786
276	GO:0006915
277	GO:0002052
278	GO:0000307
279	GO:0070557
280	GO:0019538
281	GO:0061032
282	GO:0030879
283	GO:0045578
284	GO:0003908
285	GO:0030521
286	GO:0046983
287	GO:0072302
288	GO:0030325
289	GO:0072182
290	GO:0007420

291	GO:0043517
292	GO:0048320
293	GO:0000165
294	GO:2000060
295	GO:0031065
296	hsa04320
297	GO:0043392
298	GO:0010942
299	GO:0043409
300	GO:2000379
301	GO:0014070
302	GO:0010468
303	GO:0045600
304	GO:0031398
305	GO:0048015
306	GO:0060439
307	GO:0046600
308	GO:2000045
309	GO:0000785
310	GO:0051893
311	GO:0006974
312	GO:0000080
313	GO:0045732
314	GO:0046902
315	GO:0071459
316	GO:0004716
317	GO:0043923
318	GO:0030857
319	GO:0060527
320	GO:0031436
321	GO:0010243
322	GO:0032091
323	GO:0090343
324	GO:0022898
325	GO:0014042
326	GO:0006808
327	GO:2001234
328	GO:0048743
329	GO:0048753
330	GO:0032848
331	GO:0043375
332	GO:0034742
333	GO:0060687
334	GO:0000186
335	GO:0032204

336	GO:0033138
337	GO:0060662
338	GO:0060421
339	GO:0030235
340	GO:0072134
341	GO:0006919
342	GO:0007369
343	GO:0043497
344	GO:0034244
345	GO:0007435
346	GO:0048853
347	GO:0090399
348	GO:0031069
349	GO:0050808
350	GO:0061198
351	GO:0072133
352	GO:0030307
353	GO:0048705
354	GO:0006306
355	GO:2000342
356	GO:0001841
357	GO:0046022
358	GO:0035265
359	GO:2000134
360	GO:0051797
361	GO:0033689
362	GO:0070602
363	GO:0006468
364	GO:0001953
365	GO:0045444
366	GO:0043525
367	GO:0060177
368	GO:2000195
369	GO:0008284
370	GO:0048048
371	GO:0032993
372	GO:0032007
373	GO:0043524
374	GO:0051902
375	GO:0000987
376	GO:0070372
377	GO:0006461
378	GO:0001656
379	GO:0047485
380	GO:0003682

381	GO:0021747
382	GO:0045596
383	GO:0030900
384	GO:0019904
385	GO:0050678
386	GO:0005757
387	GO:0046674
388	GO:0032976
389	GO:0051318
390	GO:0022405
391	GO:0043296
392	GO:0019903
393	GO:0000320
394	GO:0071363
395	GO:0030616
396	GO:0060823
397	GO:0051248
398	GO:0001743
399	GO:0070932
400	GO:0071559
401	GO:0060529
402	GO:0038028
403	GO:0010248
404	GO:0016328
405	GO:0042981
406	GO:0043009
407	GO:0033600
408	GO:0051492
409	GO:0001042
410	GO:0060209
411	GO:0042803
412	GO:0051782
413	GO:0001658
414	GO:0009636
415	GO:0060484
416	GO:0051668
417	GO:0001077
418	GO:0060742
419	GO:0009898
420	GO:0001706
421	hsa05211
422	GO:0045787
423	GO:0030690
424	GO:0070491
425	GO:0032872

426	GO:0021881
427	GO:0045944
428	GO:0060441
429	GO:0010800
430	GO:0046329
431	GO:0006357
432	GO:0018205
433	GO:0046982
434	GO:0045165
435	GO:0046621
436	GO:0046929
437	GO:0051402
438	GO:0072001
439	GO:0044336
440	GO:0014031
441	GO:0048715
442	GO:0032436
443	GO:0042493
444	GO:0001707
445	GO:0030216
446	GO:0048103
447	GO:0044212
448	GO:0035441
449	GO:0043006
450	GO:0007403
451	hsa04012
452	GO:0048859
453	GO:0030512
454	GO:0043523
455	GO:0023014
456	GO:0055096
457	GO:0009887
458	GO:0048011
459	GO:0009411
460	GO:0042993
461	GO:0035026
462	GO:0009950
463	GO:0060433
464	GO:0051276
465	GO:2000653
466	GO:0044029
467	GO:0034349
468	GO:0045768
469	GO:0090400
470	GO:0000979

471	GO:0014858
472	GO:0070447
473	GO:0046534
474	GO:1900175
475	GO:2000062
476	GO:0007502
477	GO:2000063
478	GO:0061189
479	GO:2000358
480	GO:0060516
481	GO:0043369
482	GO:0080125
483	GO:0060459
484	GO:0060458
485	GO:2000357
486	GO:0060782
487	GO:0060783
488	GO:0048318
489	GO:0009792
490	GO:0090344
491	GO:0021542
492	GO:0022612
493	GO:0035726
494	hsa04010
495	GO:0071230
496	GO:0043518
497	GO:0002360
498	GO:0045665
499	GO:0060599
500	GO:0042153

b) mRMR features list

Rank	Feature name
1	hsa05219
2	GO:0005678
3	GO:0050808
4	GO:0003714
5	GO:0003908
6	GO:0007092
7	GO:0071364
8	GO:0015964
9	GO:0044430
10	GO:0048102
11	GO:0048147
12	GO:0072166

13	GO:0016918
14	GO:0019538
15	GO:0043508
16	GO:0001841
17	GO:0031575
18	GO:0051721
19	GO:0034613
20	GO:0033673
21	GO:0071157
22	GO:0033235
23	GO:0006407
24	GO:0001829
25	GO:0035694
26	GO:0090403
27	GO:0016310
28	GO:0031398
29	GO:0010424
30	GO:0043535
31	GO:0017017
32	GO:0033326
33	GO:0032390
34	GO:0009103
35	GO:0045578
36	GO:0032794
37	GO:0042518
38	GO:0070215
39	GO:0021612
40	GO:0001952
41	GO:0042551
42	GO:2000134
43	GO:0045295
44	GO:0090200
45	GO:0050508
46	GO:0047710
47	GO:0030695
48	GO:0010165
49	GO:0030336
50	GO:0055100
51	GO:0045884
52	GO:0048742
53	GO:0000819
54	GO:0050821
55	GO:0030852
56	GO:0097057
57	GO:0060769

58	GO:0071479
59	GO:0046580
60	GO:0003156
61	GO:0031647
62	GO:0045604
63	GO:0051893
64	GO:0031571
65	GO:0006306
66	GO:0071481
67	GO:0033596
68	GO:0051146
69	GO:0019841
70	GO:0042271
71	GO:0051097
72	GO:0060023
73	GO:0051865
74	GO:0000115
75	GO:0060340
76	GO:0035189
77	GO:0002902
78	GO:0007406
79	GO:0043666
80	GO:2000500
81	GO:0051444
82	GO:0033601
83	GO:0048714
84	GO:0022601
85	GO:0031665
86	GO:0032007
87	hsa05216
88	GO:0046718
89	GO:0006307
90	GO:0048048
91	GO:0032321
92	GO:0071279
93	GO:0007090
94	GO:0001776
95	GO:0070245
96	GO:0048733
97	GO:0046329
98	GO:0060547
99	GO:0043281
100	GO:0051894
101	GO:0035021
102	GO:0004727

103	GO:0071158
104	GO:0000791
105	GO:0035088
106	GO:0035802
107	GO:0005712
108	GO:0048546
109	GO:0072332
110	GO:0051492
111	GO:0060024
112	GO:0006309
113	GO:2001022
114	GO:0035265
115	GO:0010506
116	GO:0071850
117	GO:0007140
118	GO:0008327
119	GO:0042058
120	GO:0008330
121	GO:0014010
122	GO:0035731
123	GO:0045792
124	GO:0021754
125	GO:0042771
126	GO:0002070
127	GO:0033561
128	GO:0035037
129	GO:0035022
130	GO:0017148
131	GO:0055105
132	GO:0008138
133	GO:0019941
134	GO:0060770
135	GO:2000041
136	GO:0051276
137	GO:2000378
138	GO:0006930
139	GO:0016151
140	GO:0051895
141	GO:0042992
142	GO:0010637
143	GO:0004861
144	GO:0050509
145	GO:0000739
146	GO:2001076
147	GO:0035414

148	GO:0060789
149	GO:0060465
150	GO:0010997
151	GO:0071385
152	GO:0030833
153	GO:0032872
154	GO:0032461
155	GO:0051153
156	GO:0048745
157	GO:2000836
158	GO:0032300
159	GO:0043550
160	GO:0010975
161	GO:0021779
162	GO:0022612
163	GO:0051400
164	GO:0034614
165	GO:0009987
166	GO:2000054
167	GO:0045090
168	GO:0035730
169	GO:0090398
170	GO:0071901
171	GO:0090343
172	GO:0060997
173	GO:0031670
174	GO:0045837
175	GO:0043152
176	GO:0071681
177	GO:0007634
178	GO:0042766
179	GO:0060706
180	GO:0070534
181	GO:0032025
182	GO:0060923
183	GO:0001953
184	GO:0016538
185	GO:0008191
186	GO:0090051
187	GO:0060074
188	GO:0060662
189	GO:0030521
190	GO:0045569
191	GO:0043517
192	GO:0002309

193	GO:2001241
194	GO:0043060
195	GO:0008064
196	GO:0046851
197	GO:0060716
198	GO:0007050
199	GO:0016514
200	GO:0007356
201	GO:0035732
202	GO:0035329
203	GO:0042328
204	GO:0035330
205	GO:0005072
206	GO:0030030
207	GO:0051782
208	GO:0071679
209	GO:0071930
210	GO:0035907
211	GO:0045685
212	GO:0000188
213	GO:2000808
214	GO:0021546
215	GO:0019210
216	GO:0017053
217	GO:0043015
218	GO:0008595
219	GO:0009410
220	GO:0000733
221	GO:0071559
222	GO:0070141
223	GO:0010452
224	GO:0090175
225	GO:0034124
226	GO:0006914
227	GO:0031461
228	GO:0051365
229	GO:0034244
230	GO:2000310
231	GO:0008634
232	GO:0043296
233	GO:0006978
234	GO:0021599
235	GO:0070026
236	GO:0007281
237	GO:0005501

238	GO:0007060
239	GO:0055106
240	GO:0043542
241	GO:0034983
242	GO:0035375
243	GO:0035033
244	GO:0033146
245	GO:0042993
246	GO:0045647
247	GO:0032535
248	GO:0043045
249	GO:0071228
250	GO:0021897
251	GO:0014034
252	GO:0072302
253	GO:0097162
254	GO:0035117
255	GO:0070059
256	GO:0071168
257	GO:0010957
258	GO:0004716
259	GO:0033692
260	GO:0045793
261	GO:0070052
262	GO:0000239
263	GO:0033327
264	GO:0070936
265	GO:0006921
266	GO:0070664
267	GO:0051595
268	GO:0045475
269	GO:0032435
270	GO:0008426
271	GO:0033088
272	GO:0061428
273	GO:0001893
274	GO:0016571
275	GO:0060876
276	GO:0045296
277	GO:2000342
278	GO:2000117
279	GO:0047485
280	GO:0060571
281	GO:0002352
282	GO:2000195

283	GO:0051257
284	GO:2001020
285	GO:0016339
286	GO:0051797
287	GO:0097105
288	GO:0034644
289	GO:0006349
290	GO:0032880
291	GO:0000093
292	GO:0021569
293	GO:0019207
294	GO:0043923
295	GO:0031235
296	GO:0043220
297	GO:0007098
298	GO:0021877
299	GO:0036023
300	GO:0035583
301	GO:0050856
302	GO:0045736
303	GO:0046546
304	GO:0035441
305	GO:0035970
306	GO:0006266
307	GO:0030235
308	GO:0046825
309	GO:0010942
310	GO:0015014
311	GO:0097107
312	GO:0050847
313	GO:0001886
314	GO:0051443
315	GO:0009008
316	GO:0032318
317	GO:0000320
318	GO:0071565
319	GO:0055096
320	GO:0000422
321	GO:0021615
322	GO:0001836
323	GO:0046882
324	GO:0021571
325	GO:0060411
326	GO:0030520
327	GO:0032090

328	GO:0048320
329	GO:0021782
330	GO:2001047
331	GO:0000785
332	GO:0021772
333	GO:0045750
334	GO:0002762
335	GO:0046022
336	GO:0010389
337	GO:0033032
338	GO:2000080
339	GO:0009890
340	GO:0030857
341	GO:0031307
342	GO:0006464
343	GO:0007497
344	GO:0016342
345	GO:0031065
346	GO:0032835
347	GO:0051271
348	GO:0048608
349	GO:0046985
350	GO:0032228
351	GO:0006346
352	GO:0060546
353	GO:0007417
354	GO:0035767
355	GO:0070372
356	GO:0001047
357	GO:0048597
358	GO:0090136
359	GO:0030891
360	GO:0060996
361	GO:0071889
362	GO:0009950
363	GO:0043295
364	GO:0051717
365	GO:0000792
366	GO:2001244
367	GO:0007422
368	GO:0034088
369	GO:0001661
370	GO:2000052
371	GO:0009411
372	GO:0031226

373	GO:0032680
374	GO:0060421
375	GO:0007185
376	GO:0043028
377	GO:0004882
378	GO:0005912
379	GO:0043523
380	GO:0005741
381	GO:0001570
382	GO:0009048
383	GO:0031931
384	GO:0030511
385	GO:0090141
386	GO:0032389
387	GO:0072498
388	GO:0008432
389	GO:0031648
390	GO:0042552
391	GO:0097194
392	GO:0004721
393	GO:0048145
394	GO:0008270
395	GO:0031658
396	GO:0003712
397	GO:0045879
398	GO:0046930
399	GO:0009826
400	GO:0043409
401	GO:0010464
402	GO:0004712
403	GO:0048562
404	GO:0032204
405	GO:0006513
406	GO:0090246
407	GO:0030675
408	GO:0006469
409	GO:0030506
410	GO:0048712
411	GO:0060011
412	GO:0045950
413	GO:0060709
414	GO:0003886
415	GO:0030296
416	GO:0071456
417	GO:0035024

418	GO:0004438
419	GO:0070557
420	GO:0010719
421	GO:0004515
422	GO:0034742
423	GO:0006473
424	GO:0007530
425	GO:0007265
426	GO:0044319
427	GO:0046902
428	GO:0051102
429	GO:0033631
430	GO:0008630
431	GO:0048807
432	GO:0001835
433	GO:0051973
434	GO:0045880
435	GO:0035726
436	GO:0090071
437	GO:0016235
438	GO:0009792
439	GO:0097190
440	GO:0030858
441	GO:0031396
442	GO:0051668
443	GO:2000119
444	GO:0061198
445	GO:0051409
446	GO:0045842
447	GO:0019211
448	GO:0046499
449	GO:0033598
450	GO:0008053
451	GO:0051898
452	GO:0060539
453	GO:0061156
454	GO:0060806
455	GO:0004704
456	GO:2000271
457	GO:0001547
458	GO:0042325
459	GO:2000008
460	GO:0045204
461	GO:0043009
462	GO:0048619

463	GO:0002360
464	GO:0060823
465	GO:0065004
466	GO:0051800
467	GO:0000795
468	GO:0043495
469	GO:0008209
470	GO:0042054
471	GO:0045762
472	GO:0005884
473	GO:0061032
474	GO:0019887
475	GO:0042069
476	GO:0070932
477	GO:0030216
478	GO:0006417
479	GO:0014003
480	GO:0032480
481	GO:0008340
482	GO:0044345
483	GO:0071398
484	GO:0042641
485	GO:0030325
486	GO:0001825
487	GO:0045209
488	hsa05213
489	GO:0048729
490	GO:0010248
491	GO:0005099
492	GO:0043569
493	GO:0016314
494	GO:0032407
495	GO:0045814
496	GO:0005042
497	GO:0046983
498	GO:0021570
499	GO:0046426
500	GO:0005881

(3) Dataset S_3

a) MaxRel features list

Rank	Feature name
1	GO:0051146
2	hsa05219
3	hsa05216

4	hsa05200
5	GO:0007050
6	GO:0032535
7	GO:0090071
8	GO:0051717
9	GO:0031658
10	GO:0033032
11	GO:0097107
12	GO:0051800
13	GO:2000808
14	GO:0050680
15	hsa05213
16	hsa05220
17	GO:0008285
18	GO:0030336
19	GO:0045792
20	hsa05218
21	GO:0042127
22	hsa05210
23	GO:0014010
24	hsa05221
25	GO:0016314
26	GO:0090398
27	GO:0060074
28	GO:0033596
29	GO:0050821
30	GO:0032286
31	GO:0007092
32	GO:0071681
33	GO:0060479
34	GO:0035112
35	GO:0030997
36	GO:0048262
37	GO:0002902
38	hsa05215
39	GO:0090394
40	hsa05223
41	GO:0071158
42	GO:0048145
43	GO:0031575
44	GO:0016342
45	GO:0071850
46	hsa05212
47	GO:0044430
48	hsa05214

49	GO:0072079
50	GO:0032794
51	GO:0006407
52	GO:0043066
53	GO:0071479
54	GO:0060749
55	GO:0071456
56	GO:0033561
57	GO:0061324
58	GO:0034750
59	GO:0072033
60	GO:0060179
61	GO:0008013
62	GO:0071364
63	GO:0072054
64	GO:0072053
65	GO:0019912
66	GO:2000278
67	GO:0090403
68	GO:0010909
69	GO:0044334
70	GO:0070369
71	GO:0033077
72	GO:0048546
73	GO:0008283
74	GO:0030308
75	GO:2000008
76	hsa04115
77	GO:0001934
78	GO:0001570
79	GO:0022408
80	GO:0055105
81	GO:0045930
82	GO:0051726
83	GO:0045667
84	GO:0048147
85	GO:0009314
86	GO:0010629
87	GO:0032880
88	GO:0001952
89	GO:0042326
90	GO:0033598
91	GO:0061002
92	GO:0070141
93	GO:0019002

94	GO:0010997
95	GO:0007569
96	GO:2001047
97	GO:0060709
98	GO:0045475
99	GO:0070412
100	GO:0008629
101	GO:0010165
102	GO:0043433
103	GO:0048146
104	GO:0007265
105	GO:0043234
106	GO:0060024
107	GO:0036023
108	GO:0042771
109	GO:0004861
110	GO:0001836
111	GO:0005924
112	GO:0031571
113	GO:0001829
114	GO:0005737
115	GO:0031647
116	GO:0045668
117	GO:0032355
118	GO:0016310
119	GO:0007406
120	GO:0050679
121	GO:0010332
122	GO:0022405
123	GO:0043542
124	GO:0045893
125	GO:0035022
126	GO:0072182
127	GO:0044336
128	GO:0030858
129	GO:0001701
130	GO:0000279
131	GO:0090230
132	GO:0004438
133	GO:0031134
134	GO:0043524
135	GO:0007420
136	GO:0001889
137	GO:0030511
138	GO:0007281

139	GO:0033601
140	GO:0043281
141	GO:0043220
142	GO:0051895
143	GO:0042802
144	GO:0000186
145	GO:0042058
146	GO:0006978
147	GO:0000165
148	GO:0042493
149	GO:0045736
150	GO:0002053
151	GO:0005072
152	GO:0010628
153	GO:0007435
154	GO:0048538
155	GO:0060644
156	GO:0005515
157	GO:0045295
158	GO:0034742
159	GO:0030097
160	hsa04012
161	hsa05222
162	GO:0008284
163	GO:0051894
164	GO:0001707
165	GO:0034747
166	GO:0030325
167	GO:0045892
168	GO:0034613
169	GO:0097105
170	GO:2000379
171	GO:0035802
172	GO:0003156
173	GO:0006469
174	GO:0032228
175	GO:0043065
176	GO:0051412
177	GO:0048733
178	GO:0072134
179	GO:0071930
180	GO:0070215
181	GO:0046825
182	GO:0071407
183	GO:0019901

184	GO:0006917
185	GO:0060736
186	GO:0043627
187	GO:0060769
188	GO:0006921
189	GO:0033138
190	GO:0048745
191	GO:0000122
192	GO:0008134
193	GO:0010975
194	GO:0071285
195	GO:0031100
196	hsa05166
197	GO:0001658
198	GO:0060421
199	GO:0061047
200	GO:0038028
201	GO:0006468
202	GO:0032993
203	GO:0060770
204	GO:0045765
205	GO:0035264
206	GO:0048589
207	GO:0072133
208	GO:0055100
209	GO:0009987
210	GO:0071363
211	GO:0043154
212	GO:0043535
213	GO:0046777
214	hsa05211
215	GO:0031670
216	GO:0001933
217	GO:0090400
218	GO:0031069
219	GO:2000060
220	GO:0019903
221	GO:0019899
222	GO:0048320
223	GO:0016328
224	hsa04350
225	GO:0060492
226	GO:0060789
227	GO:0034088
228	GO:0031253

229	GO:0051492
230	GO:0038085
231	GO:0014070
232	GO:0009887
233	GO:0045445
234	GO:0007507
235	GO:0051444
236	GO:0019538
237	GO:0045578
238	GO:0010033
239	GO:0051893
240	GO:0070245
241	GO:0060916
242	GO:0008543
243	GO:0051318
244	GO:0060923
245	GO:0043280
246	hsa04520
247	GO:0017015
248	GO:0070602
249	GO:0045786
250	GO:0043508
251	GO:0060066
252	GO:0001776
253	GO:0042153
254	GO:0045732
255	GO:0007403
256	GO:0046022
257	GO:0034644
258	GO:0001938
259	GO:0042129
260	GO:0043550
261	GO:0060687
262	GO:0030879
263	GO:0030307
264	GO:2000134
265	GO:0001047
266	GO:0007417
267	GO:0051097
268	GO:0048859
269	GO:0045884
270	GO:0001657
271	GO:2001076
272	GO:0007356
273	GO:0043409

274	GO:0007179
275	GO:0045950
276	GO:0048617
277	GO:0060716
278	GO:0002326
279	GO:0002052
280	GO:0030335
281	GO:0001085
282	GO:2001220
283	GO:0090096
284	GO:0044346
285	hsa04320
286	GO:0071922
287	GO:0043392
288	GO:0045768
289	GO:0051797
290	GO:0071559
291	GO:0042803
292	GO:0043525
293	GO:0010468
294	GO:0001953
295	GO:0022898
296	GO:2001234
297	GO:0032848
298	GO:0006808
299	GO:0048743
300	GO:0043375
301	GO:0014042
302	GO:0048753
303	GO:0006309
304	GO:0043060
305	GO:0051257
306	GO:0007060
307	GO:0019904
308	GO:0070411
309	GO:0033088
310	GO:0001822
311	GO:0035414
312	GO:0048318
313	GO:0008633
314	GO:0043296
315	GO:0048853
316	GO:0021747
317	GO:0033673
318	GO:0060440

319	GO:0030224
320	GO:0000790
321	GO:0071230
322	GO:0070557
323	GO:0004713
324	GO:0051000
325	GO:0008630
326	GO:0009898
327	GO:0035189
328	GO:0010800
329	GO:0072277
330	GO:0051902
331	GO:0050808
332	GO:0003338
333	GO:0030910
334	GO:0043652
335	GO:0008634
336	GO:0004716
337	GO:0030616
338	GO:0045597
339	GO:0048011
340	GO:0042551
341	GO:0006582
342	GO:0048015
343	GO:0010942
344	GO:0005712
345	GO:0032390
346	GO:0007173
347	GO:0070372
348	GO:0008637
349	GO:0001503
350	GO:0030178
351	GO:0022601
352	GO:0001649
353	GO:0048714
354	GO:0007369
355	GO:2000617
356	GO:0070512
357	GO:2000620
358	GO:0031436
359	GO:0030235
360	GO:0030539
361	GO:0046982
362	GO:2000054
363	GO:0051668

364	GO:0070507
365	GO:0072001
366	GO:0001706
367	GO:0046600
368	GO:0007398
369	GO:0005913
370	GO:0033326
371	GO:0003690
372	GO:0061032
373	GO:0048565
374	GO:0045595
375	GO:0046983
376	GO:0030855
377	GO:0030900
378	GO:0035026
379	GO:0043009
380	GO:0033687
381	GO:0030856
382	GO:0008286
383	GO:0072112
384	GO:0006919
385	GO:0072166
386	GO:0044212
387	GO:0061198
388	GO:0030324
389	GO:0060529
390	GO:0030521
391	GO:0045944
392	GO:0010595
393	GO:0042060
394	GO:0001843
395	GO:0030513
396	GO:0050681
397	GO:0042993
398	GO:0072302
399	GO:0031625
400	GO:0001702
401	GO:0042475
402	GO:0031226
403	GO:0001837
404	GO:0000733
405	GO:0000739
406	GO:0014031
407	GO:0004672
408	GO:0035033

409	GO:0034333
410	GO:0010243
411	GO:0048705
412	GO:0003136
413	GO:0021542
414	GO:0030857
415	GO:0060197
416	GO:0051365
417	GO:0060441
418	GO:0051898
419	GO:0000904
420	GO:0042770
421	GO:0043006
422	GO:0002039
423	GO:0010718
424	GO:0034349
425	GO:0043536
426	GO:0047485
427	GO:0002320
428	GO:0045180
429	GO:0051591
430	GO:0035441
431	GO:2000017
432	hsa04722
433	GO:0055096
434	GO:0045596
435	GO:0090344
436	GO:0071157
437	GO:0014067
438	GO:0010839
439	GO:0045931
440	GO:0032872
441	GO:0033689
442	GO:0044029
443	GO:2000653
444	GO:0048660
445	GO:0001569
446	GO:0001102
447	GO:0014068
448	GO:0071901
449	GO:0042992
450	GO:0048754
451	GO:0051248
452	GO:0060823
453	GO:0001743

454	GO:2001241
455	GO:0021881
456	GO:2000195
457	GO:0031122
458	GO:0050678
459	GO:0072332
460	GO:0051153
461	GO:0048742
462	GO:0032204
463	hsa04010
464	GO:0045444
465	GO:0035988
466	GO:0001525
467	GO:0032091
468	GO:0010564
469	GO:0016539
470	GO:0046639
471	GO:0035021
472	GO:0001944
473	GO:0006461
474	GO:0072207
475	GO:0001077
476	GO:0032318
477	GO:0043923
478	GO:0048286
479	GO:0034504
480	GO:0046902
481	GO:0030695
482	GO:0060599
483	GO:0001656
484	GO:0000320
485	GO:0031065
486	GO:0030690
487	GO:0045600
488	GO:0000307
489	GO:0030111
490	GO:0046621
491	GO:0032570
492	GO:0030889
493	GO:0048102
494	GO:0035116
495	GO:0010801
496	GO:0031659
497	GO:0034244
498	GO:0007090

499	GO:0042524
500	GO:0060484

b) mRMR features list

Rank	Feature name
1	GO:0051146
2	GO:0033596
3	GO:0090403
4	GO:0060421
5	GO:0042271
6	GO:0043508
7	GO:0038085
8	GO:0009987
9	GO:0033235
10	GO:0051893
11	GO:0071364
12	GO:0047710
13	GO:0060074
14	GO:0050808
15	GO:0043281
16	GO:0033673
17	GO:0048147
18	GO:2001241
19	GO:0006407
20	GO:0048733
21	hsa00100
22	GO:0030336
23	GO:0010424
24	GO:0035694
25	GO:0090398
26	GO:0032300
27	GO:0045792
28	GO:0022601
29	GO:0035414
30	GO:0014003
31	GO:0010165
32	GO:0001829
33	GO:0001952
34	GO:0010506
35	GO:0031575
36	GO:0045604
37	GO:0032794
38	GO:0060716
39	GO:0043015
40	GO:0055100

41	GO:2001076
42	GO:0051895
43	GO:0030695
44	GO:0060023
45	GO:0014010
46	GO:0016918
47	GO:0033326
48	GO:0030224
49	GO:0044430
50	GO:0051444
51	GO:0006306
52	GO:0033601
53	GO:0040008
54	GO:0000733
55	GO:0001707
56	GO:0035022
57	GO:0097105
58	GO:0045606
59	GO:0048745
60	GO:0003908
61	GO:0016342
62	GO:0008634
63	GO:0009826
64	GO:0043666
65	GO:0045578
66	GO:0031670
67	GO:0051492
68	GO:0042551
69	GO:0015964
70	hsa05221
71	GO:0071901
72	GO:0070215
73	GO:0019208
74	GO:0035802
75	GO:0045884
76	GO:0070507
77	GO:0048102
78	GO:0016339
79	GO:0030852
80	GO:0070372
81	GO:0042771
82	GO:0051894
83	GO:2000134
84	GO:0017148
85	GO:2000378

86	GO:0030030
87	GO:0071850
88	GO:0010839
89	GO:2000119
90	GO:0001570
91	GO:0006921
92	GO:0071391
93	GO:0043045
94	GO:0050821
95	GO:0045950
96	GO:0070052
97	GO:0043295
98	GO:0005913
99	GO:0043550
100	GO:0048546
101	GO:0051721
102	GO:0031226
103	GO:0051153
104	GO:0010717
105	GO:0042058
106	GO:0007356
107	GO:0016310
108	GO:0051097
109	GO:0033561
110	GO:0051865
111	GO:0034333
112	GO:0001953
113	GO:0006473
114	GO:0007497
115	GO:0051400
116	GO:0046825
117	GO:0072133
118	GO:0016151
119	GO:0021754
120	GO:0032872
121	GO:0071157
122	GO:0009948
123	GO:0007092
124	GO:0051606
125	GO:0030100
126	GO:0045090
127	GO:0060770
128	GO:0045899
129	GO:0034614
130	GO:0034613

131	GO:0060749
132	GO:0002309
133	GO:0046851
134	GO:0060465
135	GO:0070318
136	GO:0048320
137	GO:0045569
138	GO:0043542
139	GO:0051057
140	GO:0007050
141	GO:0019538
142	GO:0002762
143	GO:0060575
144	GO:0042518
145	GO:0030325
146	GO:0002070
147	GO:0043060
148	GO:0010801
149	GO:0031647
150	GO:0000904
151	GO:0000791
152	GO:0005501
153	GO:0046022
154	GO:0003156
155	GO:0060024
156	GO:0017015
157	GO:0055105
158	GO:0047485
159	GO:0044319
160	GO:0022612
161	GO:0070245
162	GO:0021612
163	GO:0016601
164	GO:0045750
165	GO:0001776
166	GO:0010975
167	GO:0007435
168	GO:0034088
169	GO:0035732
170	GO:0034742
171	GO:0043366
172	GO:0042766
173	GO:0045793
174	GO:0050680
175	GO:2000310

176	GO:0008340
177	GO:0035441
178	GO:0035988
179	GO:0005884
180	hsa05219
181	GO:0060923
182	GO:0060242
183	GO:0090175
184	GO:0071479
185	GO:0042993
186	GO:0016514
187	GO:0051385
188	GO:0000739
189	GO:0030111
190	GO:0035021
191	GO:0019841
192	GO:0043220
193	GO:0097155
194	GO:0051271
195	GO:0046329
196	GO:0051257
197	GO:0072112
198	GO:0071158
199	GO:0006417
200	GO:0016327
201	GO:0051365
202	GO:0045814
203	GO:0010997
204	GO:0048014
205	GO:0043495
206	GO:0001843
207	GO:0032835
208	hsa05216
209	GO:0046718
210	GO:0006307
211	GO:0034124
212	GO:0071559
213	GO:0007090
214	GO:0014037
215	GO:0071481
216	GO:0042326
217	GO:0031398
218	GO:0071679
219	GO:0031253
220	GO:0072332

221	GO:0007185
222	GO:0045656
223	GO:0032956
224	GO:0035730
225	GO:0045295
226	GO:0071279
227	GO:0009605
228	GO:0048853
229	GO:0055096
230	GO:0032007
231	GO:0090191
232	GO:0072166
233	GO:0070059
234	GO:0038091
235	GO:0090343
236	GO:0034629
237	GO:0008330
238	GO:0001702
239	GO:0045930
240	GO:0019898
241	GO:0004438
242	GO:0031571
243	GO:0000115
244	GO:0007060
245	GO:0008595
246	GO:0046473
247	GO:0043409
248	GO:0048742
249	GO:0007605
250	GO:0035970
251	GO:0071681
252	GO:0019941
253	GO:0030889
254	GO:0021892
255	GO:0032228
256	GO:0002009
257	GO:0033598
258	GO:0071564
259	GO:0048859
260	GO:0046621
261	GO:0046790
262	GO:0007406
263	GO:0031619
264	GO:0043046
265	GO:0072277

266	GO:0045545
267	GO:0060997
268	GO:0060706
269	GO:0050699
270	GO:0044345
271	GO:0060546
272	GO:0072302
273	GO:0010957
274	GO:0000228
275	GO:0032880
276	GO:0097156
277	GO:0097057
278	GO:0048145
279	GO:0035583
280	GO:0070557
281	GO:0001931
282	GO:0060197
283	GO:0070534
284	GO:0034405
285	GO:0009314
286	GO:0045184
287	GO:0072134
288	GO:2000096
289	GO:0061002
290	GO:0005678
291	GO:0021800
292	GO:0043535
293	GO:0045837
294	GO:2000054
295	GO:0060571
296	GO:0010225
297	GO:2000008
298	GO:0050508
299	GO:0006309
300	GO:0050920
301	GO:0031235
302	GO:0030857
303	GO:0031256
304	GO:0006978
305	GO:0021569
306	GO:0005072
307	GO:0010592
308	GO:0048070
309	GO:0070664
310	GO:0019210

311	GO:0001774
312	GO:0021879
313	GO:0035791
314	GO:0051797
315	GO:0061428
316	GO:0001836
317	GO:2000052
318	GO:0043296
319	GO:0061032
320	GO:0051276
321	GO:0007158
322	GO:0006930
323	GO:0019002
324	GO:0001502
325	GO:0042415
326	GO:0035189
327	GO:0060736
328	GO:0002089
329	GO:0032137
330	GO:0031122
331	GO:0032211
332	GO:0050847
333	GO:0071456
334	GO:0007162
335	GO:2001020
336	GO:0042641
337	GO:0000186
338	GO:0090027
339	GO:0090394
340	GO:0004415
341	GO:0005815
342	GO:0043374
343	GO:0016328
344	GO:0032461
345	GO:0009048
346	GO:0030511
347	GO:0070026
348	GO:0001893
349	GO:2000271
350	GO:0048597
351	GO:0032204
352	GO:0061146
353	GO:0005712
354	GO:0010942
355	GO:0001825

356	GO:0045647
357	GO:0003334
358	GO:0060394
359	GO:0031931
360	GO:0051902
361	GO:0060512
362	GO:0003886
363	GO:2000060
364	GO:0033593
365	GO:0042524
366	GO:0008147
367	GO:0007265
368	GO:0042438
369	GO:0060011
370	GO:2000500
371	GO:0045842
372	GO:0032090
373	GO:0005737
374	GO:0097119
375	GO:0046882
376	GO:0032862
377	GO:0001835
378	GO:2000080
379	GO:0060769
380	GO:0032535
381	GO:0008191
382	GO:0010637
383	GO:0005019
384	GO:0032321
385	GO:0043433
386	GO:0060748
387	GO:0043152
388	GO:0035731
389	GO:0007184
390	GO:0060876
391	GO:0031668
392	GO:0032390
393	GO:0010761
394	GO:0007416
395	GO:0046499
396	GO:0032444
397	GO:2001047
398	GO:0030855
399	GO:0006907
400	GO:0046930

401	GO:0060806
402	GO:0004861
403	GO:0042117
404	GO:0035330
405	GO:0060789
406	GO:0035924
407	GO:0090246
408	GO:0010485
409	GO:0060547
410	GO:0034644
411	GO:0003417
412	GO:0016538
413	GO:0060492
414	GO:0097021
415	GO:0051020
416	GO:0090071
417	GO:0051595
418	GO:0043517
419	GO:0034244
420	GO:0051493
421	GO:0021759
422	GO:0022011
423	GO:0060687
424	GO:0035037
425	GO:0097162
426	GO:0008013
427	GO:0032407
428	GO:0035033
429	GO:0007634
430	GO:0060022
431	GO:0002352
432	GO:0072207
433	GO:0007098
434	GO:0010748
435	GO:0030891
436	GO:0060346
437	GO:0051318
438	GO:0060711
439	GO:0070853
440	GO:0045668
441	GO:0004704
442	GO:2001220
443	GO:0007270
444	GO:0035375
445	GO:0051248

446	GO:0017166
447	GO:0019912
448	GO:0031503
449	GO:0042129
450	GO:0002064
451	GO:0016235
452	GO:0002902
453	GO:0060385
454	GO:0030539
455	GO:1900273
456	GO:0010464
457	GO:0009890
458	GO:0035088
459	GO:0008629
460	GO:0072284
461	GO:0008601
462	GO:0070563
463	GO:0030947
464	GO:0035026
465	GO:0061418
466	GO:0045667
467	GO:0035024
468	GO:0007281
469	GO:0019215
470	GO:0018205
471	GO:0070016
472	GO:0001841
473	GO:0003407
474	GO:0051717
475	GO:2000041
476	GO:0007182
477	GO:0031462
478	GO:0022408
479	GO:0021599
480	GO:0035907
481	GO:0035264
482	GO:0090141
483	GO:0035176
484	GO:0000279
485	GO:0035117
486	GO:0008432
487	GO:0006776
488	GO:0030616
489	GO:0090116
490	GO:0045859

491	GO:0032139
492	GO:0032435
493	GO:0031065
494	GO:0035305
495	GO:0072091
496	GO:0090400
497	GO:0060429
498	GO:0046626
499	GO:0045621
500	GO:0050678

(4) Dataset S_4

a) MaxRel features list

Rank	Feature name
1	hsa05219
2	GO:0051146
3	hsa05200
4	GO:0007050
5	hsa05220
6	GO:0031658
7	GO:0090071
8	GO:0032535
9	GO:2000808
10	GO:0051800
11	GO:0097107
12	GO:0033032
13	GO:0051717
14	hsa05216
15	GO:0050680
16	hsa05218
17	GO:0071850
18	hsa05213
19	GO:0030336
20	GO:0007092
21	GO:0016314
22	GO:0051726
23	hsa04115
24	hsa05210
25	GO:0008285
26	GO:0042127
27	hsa05215
28	GO:0032286
29	GO:0004861
30	GO:0050821
31	hsa05223

32	hsa05221
33	GO:0002902
34	GO:0048546
35	hsa05212
36	GO:0030308
37	GO:0043066
38	GO:0001889
39	GO:0060179
40	GO:0008283
41	GO:0090394
42	GO:0044430
43	GO:0090398
44	GO:0071456
45	GO:0045475
46	GO:0042326
47	GO:0033596
48	GO:0055105
49	GO:0045736
50	GO:0071479
51	GO:0060074
52	GO:0045792
53	GO:0033601
54	GO:0010165
55	GO:0033561
56	GO:0045667
57	GO:0031575
58	GO:0014010
59	GO:0071681
60	GO:0060479
61	GO:0030997
62	GO:0035112
63	GO:0048262
64	GO:0090403
65	GO:0048147
66	GO:0001570
67	GO:0010997
68	GO:0070141
69	GO:0031100
70	GO:0043542
71	hsa05214
72	GO:0001934
73	GO:0071158
74	GO:0048146
75	GO:0034750
76	GO:0042058

77	GO:0031571
78	GO:0030511
79	GO:2000278
80	GO:0019912
81	GO:0043433
82	GO:0048145
83	GO:0019002
84	GO:0061324
85	GO:0003156
86	GO:0035802
87	GO:0010629
88	GO:0090230
89	GO:0000279
90	GO:0033077
91	GO:0031134
92	GO:0006407
93	GO:0032794
94	GO:0007569
95	GO:0008629
96	GO:0031069
97	GO:0032355
98	GO:0072079
99	GO:0033598
100	GO:2000008
101	GO:0071930
102	GO:0010628
103	GO:0002053
104	GO:0031647
105	GO:0006469
106	GO:0001701
107	GO:0070369
108	GO:0044334
109	GO:0010909
110	GO:0048538
111	GO:0060770
112	GO:0001952
113	hsa05222
114	hsa05166
115	GO:0071364
116	GO:0019901
117	GO:0050679
118	GO:0010332
119	GO:0007435
120	GO:0060024
121	GO:0072054

122	GO:0072053
123	GO:0072033
124	GO:0001836
125	GO:0042802
126	GO:0055100
127	GO:0032880
128	GO:0042771
129	GO:0036023
130	GO:0001829
131	GO:0045893
132	GO:0045930
133	GO:0016342
134	GO:2000379
135	GO:0045668
136	GO:0030097
137	GO:0000122
138	GO:0034613
139	GO:0030858
140	GO:0005072
141	GO:0043550
142	GO:0045295
143	GO:0060923
144	GO:0071285
145	GO:0008284
146	GO:0022408
147	GO:0071922
148	GO:0042493
149	GO:0007417
150	GO:0060749
151	GO:0006917
152	GO:0030879
153	GO:0043281
154	GO:0051412
155	GO:0009314
156	GO:0008013
157	GO:0007406
158	GO:0045595
159	GO:0051257
160	GO:0043060
161	GO:0007060
162	GO:0070215
163	GO:0032390
164	GO:0005712
165	GO:0043220
166	GO:0045892

167	GO:0033088
168	GO:0035022
169	GO:0033673
170	GO:0070245
171	GO:0001047
172	GO:0060644
173	GO:2000054
174	GO:0019903
175	GO:0048714
176	GO:0090096
177	GO:2001220
178	GO:0044346
179	GO:0019538
180	GO:0045786
181	GO:0008634
182	GO:0030856
183	GO:0007420
184	GO:0030325
185	GO:0060769
186	GO:0035189
187	GO:0008134
188	GO:0022405
189	GO:0034088
190	GO:0051318
191	GO:0005515
192	GO:0035264
193	GO:0072332
194	GO:0070412
195	GO:0005924
196	GO:0034644
197	GO:0045445
198	GO:0045765
199	GO:0030307
200	GO:0003690
201	GO:0045597
202	GO:0061002
203	GO:0007507
204	GO:0032228
205	GO:0071407
206	GO:0048015
207	GO:0048745
208	GO:0043065
209	GO:0000080
210	GO:0048565
211	GO:0043154

212	GO:0009887
213	GO:0045596
214	GO:0048103
215	GO:0043524
216	GO:0043627
217	GO:0033687
218	GO:0003140
219	GO:0031670
220	GO:0072001
221	GO:0045768
222	GO:0045950
223	GO:0045944
224	GO:0043535
225	GO:0060421
226	GO:0048589
227	GO:0042803
228	GO:0030111
229	GO:0030178
230	GO:0001656
231	GO:0032570
232	GO:0045578
233	GO:0014070
234	GO:0048102
235	GO:0043508
236	GO:0005113
237	GO:0046982
238	GO:0048853
239	GO:0090344
240	GO:0010468
241	GO:0090090
242	GO:0043280
243	GO:0007398
244	GO:0006978
245	GO:0007281
246	GO:0032993
247	GO:0045732
248	GO:0006919
249	GO:0072182
250	GO:0007403
251	GO:0010243
252	GO:0090200
253	GO:0051895
254	GO:0051097
255	GO:0007356
256	GO:2001076

257	GO:0021983
258	GO:0022601
259	GO:0051444
260	GO:0006921
261	GO:0048733
262	GO:0032204
263	GO:0044336
264	GO:0072166
265	GO:0006468
266	GO:0060709
267	GO:2001047
268	GO:0071157
269	GO:0031625
270	GO:0003908
271	GO:0000979
272	GO:0051894
273	GO:0002052
274	GO:0060439
275	GO:0001649
276	GO:0000307
277	GO:0060440
278	GO:0001707
279	GO:0006309
280	GO:0001933
281	GO:0060916
282	GO:0055096
283	GO:0048557
284	GO:2000041
285	GO:0008630
286	GO:0046825
287	GO:0040008
288	GO:0031668
289	GO:0007090
290	GO:0030857
291	GO:0016539
292	GO:0046639
293	GO:0030335
294	hsa04110
295	GO:0061032
296	GO:0048859
297	GO:0001077
298	GO:0001658
299	GO:0038028
300	GO:0042992
301	GO:0033138

302	GO:0000165
303	GO:0060441
304	GO:0007369
305	GO:0008633
306	GO:0019899
307	GO:0051898
308	GO:0072302
309	hsa04012
310	GO:0042475
311	GO:0030514
312	GO:0060492
313	GO:0010718
314	GO:0071363
315	GO:0031648
316	GO:0046983
317	GO:0008637
318	GO:0007265
319	GO:0046022
320	GO:0030900
321	GO:0008543
322	GO:0043234
323	GO:0043392
324	GO:0021542
325	GO:0030855
326	GO:0007179
327	GO:0043525
328	GO:0046851
329	GO:0060736
330	GO:0032461
331	GO:0001706
332	hsa04350
333	GO:0042551
334	GO:0001657
335	GO:0002326
336	GO:0005737
337	GO:0044029
338	GO:2000653
339	GO:0007346
340	GO:0009636
341	GO:0043517
342	GO:0061047
343	GO:0071230
344	GO:0070557
345	GO:0015964
346	GO:0047710

347	GO:0072112
348	GO:0072134
349	GO:0045737
350	GO:0010942
351	GO:0016604
352	GO:0060484
353	GO:0045931
354	GO:0014003
355	GO:0030275
356	GO:0072133
357	GO:0033326
358	GO:0034747
359	GO:0034097
360	GO:0030539
361	GO:0016328
362	GO:0035116
363	GO:0003406
364	GO:0043409
365	GO:0060662
366	GO:0046546
367	GO:0051668
368	GO:0051797
369	GO:0048705
370	GO:0043296
371	GO:0060433
372	GO:0001776
373	GO:2000270
374	GO:0070507
375	GO:0034742
376	GO:0022898
377	GO:0043375
378	GO:2001234
379	GO:0006808
380	GO:0014042
381	GO:0032848
382	GO:0048753
383	GO:0048743
384	GO:2000271
385	GO:2000080
386	GO:0090246
387	GO:2000052
388	GO:0044345
389	GO:0001944
390	GO:0072207
391	GO:0001822

392	GO:0030521
393	GO:0009898
394	GO:0050808
395	GO:0033689
396	GO:0042981
397	GO:0010033
398	GO:0000733
399	GO:0000739
400	GO:0048617
401	hsa05211
402	GO:0060783
403	GO:0007502
404	GO:0046534
405	GO:0043369
406	GO:0080125
407	GO:2000063
408	GO:2000062
409	GO:0070447
410	GO:2000358
411	GO:1900175
412	GO:0060516
413	GO:2000357
414	GO:0060458
415	GO:0014858
416	GO:0061189
417	GO:0060459
418	GO:0060782
419	GO:2000195
420	GO:0061053
421	GO:0045842
422	GO:0051782
423	GO:0000079
424	GO:0001702
425	GO:0048318
426	GO:0001953
427	GO:2000117
428	GO:0006915
429	GO:0021747
430	GO:0097105
431	GO:0048754
432	GO:0090399
433	GO:0042153
434	GO:0009790
435	GO:0046677
436	GO:0034504

437	GO:0030324
438	GO:0060021
439	GO:0071899
440	GO:0009411
441	GO:0008584
442	GO:0014068
443	GO:0051902
444	GO:0033235
445	GO:0005667
446	GO:0045740
447	GO:0014067
448	GO:0042129
449	GO:0090175
450	GO:0060066
451	GO:0001954
452	GO:0060687
453	GO:0048663
454	GO:0070888
455	GO:0005757
456	GO:0046674
457	GO:0032976
458	hsa04320
459	GO:0072284
460	GO:0035414
461	GO:0009880
462	GO:0001503
463	GO:0003338
464	GO:0071559
465	GO:0019210
466	GO:0045600
467	GO:0032091
468	GO:0004438
469	GO:0042733
470	GO:0007228
471	GO:0060070
472	GO:0048701
473	GO:0070372
474	GO:0048706
475	GO:0030616
476	GO:0060340
477	GO:0001841
478	GO:0030690
479	GO:0035021
480	GO:0045656
481	GO:2000134

482	GO:0010975
483	GO:0008286
484	GO:0070602
485	GO:0042518
486	GO:0001085
487	GO:0016310
488	GO:0004716
489	GO:0009950
490	GO:0043491
491	GO:2000729
492	GO:0048715
493	GO:0016538
494	GO:0051091
495	GO:0032007
496	GO:0021766
497	GO:0045165
498	GO:0048742
499	GO:0051153
500	GO:0060529

b) mRMR features list

Rank	Feature name
1	hsa05219
2	GO:0030695
3	GO:0022601
4	GO:0090403
5	GO:0003156
6	GO:0003908
7	GO:0033673
8	GO:0042058
9	GO:0044430
10	GO:0051146
11	GO:0015964
12	GO:0014003
13	GO:0007092
14	GO:0035694
15	GO:0019538
16	GO:0040008
17	GO:0071364
18	GO:0048147
19	GO:0050808
20	GO:0016918
21	GO:0048546
22	GO:0033235
23	GO:0010165

24	GO:0060465
25	GO:0006407
26	GO:0042518
27	GO:0001829
28	GO:0071850
29	GO:2000041
30	GO:0043550
31	GO:0017148
32	GO:0042271
33	GO:0001570
34	GO:0032390
35	GO:0048102
36	GO:0001952
37	GO:0045295
38	GO:0033601
39	GO:0010424
40	GO:0090200
41	GO:0034613
42	GO:0045792
43	GO:0046790
44	GO:0043508
45	GO:0055100
46	GO:0031575
47	GO:0000115
48	GO:0047710
49	GO:0050821
50	GO:0000819
51	GO:0060770
52	GO:0033596
53	GO:0035802
54	GO:0060346
55	GO:0055105
56	GO:0035414
57	GO:0051097
58	GO:0030336
59	GO:0061428
60	GO:0032794
61	GO:0072498
62	GO:0006776
63	GO:0031668
64	GO:0097105
65	GO:0019210
66	GO:0035022
67	GO:0006307
68	GO:0060340

69	GO:0072332
70	GO:0051782
71	GO:0042551
72	GO:0045750
73	GO:0043542
74	GO:0048742
75	GO:0043666
76	GO:0033687
77	GO:0090175
78	GO:0004861
79	GO:0060923
80	GO:0043152
81	GO:0007090
82	GO:2000304
83	GO:0060216
84	GO:0051721
85	GO:0007435
86	GO:0043281
87	GO:0019841
88	GO:0071479
89	GO:0030111
90	GO:0060709
91	GO:0032300
92	GO:0016538
93	GO:0042992
94	GO:0071279
95	GO:0033326
96	GO:0016339
97	GO:0090398
98	GO:0046851
99	GO:0051492
100	GO:0071157
101	GO:0007398
102	GO:0000733
103	GO:0042326
104	GO:0043374
105	GO:0045475
106	hsa00100
107	GO:0033561
108	GO:0042069
109	GO:0019207
110	GO:0033088
111	hsa05221
112	GO:0070507
113	GO:0043495

114	GO:0051257
115	GO:0030852
116	GO:0031670
117	GO:2000117
118	GO:0042771
119	GO:0046426
120	GO:0007356
121	GO:0031647
122	GO:0045667
123	GO:0046718
124	GO:0032461
125	GO:0030511
126	GO:0035375
127	GO:0032007
128	GO:0016151
129	GO:0046825
130	GO:0070245
131	GO:0048714
132	GO:0008544
133	GO:0045837
134	GO:0035189
135	GO:2000271
136	GO:0001953
137	GO:0048145
138	GO:0071385
139	GO:0055096
140	GO:0000979
141	GO:0045604
142	GO:0060179
143	GO:0021754
144	GO:0051893
145	GO:0045736
146	GO:0030100
147	GO:0033598
148	GO:2000119
149	GO:0005712
150	GO:0007162
151	GO:0006346
152	GO:0046882
153	GO:0070215
154	GO:0030030
155	GO:0072112
156	GO:0044342
157	GO:0033593
158	GO:0071481

159	GO:0048745
160	GO:2001241
161	GO:0008634
162	GO:2000134
163	GO:0005501
164	hsa05220
165	GO:0097162
166	GO:0002089
167	GO:0051444
168	GO:0051262
169	GO:0010839
170	GO:0008595
171	GO:0051276
172	GO:0022029
173	GO:0070141
174	GO:2000080
175	GO:0034088
176	GO:0051271
177	GO:0035730
178	GO:0005113
179	GO:0070534
180	GO:0032228
181	GO:0031571
182	GO:2001076
183	GO:0046022
184	GO:0043296
185	GO:0010637
186	GO:0060024
187	GO:0043517
188	GO:0045647
189	GO:0044319
190	GO:0048733
191	GO:0009826
192	GO:0043060
193	GO:0000739
194	GO:0001841
195	GO:0090246
196	GO:0030857
197	GO:0009410
198	GO:0001776
199	GO:0006921
200	GO:0008054
201	GO:0090096
202	GO:2000484
203	GO:0005072

204	GO:0004702
205	GO:0051153
206	GO:0002902
207	GO:0050508
208	GO:0043045
209	GO:2000378
210	GO:0014010
211	GO:0043295
212	GO:0030325
213	GO:0051726
214	GO:2000052
215	GO:0006097
216	GO:0090343
217	GO:0032956
218	GO:0021889
219	GO:2000836
220	GO:0007050
221	GO:0046580
222	GO:0007060
223	GO:0021571
224	GO:0045884
225	GO:0001656
226	GO:0016342
227	GO:0071930
228	GO:0051902
229	GO:0051865
230	GO:0072166
231	GO:0070372
232	GO:0019002
233	GO:0044345
234	GO:0060706
235	GO:0035583
236	GO:0010997
237	GO:0006306
238	GO:0001889
239	GO:2000310
240	GO:0031619
241	GO:0050680
242	GO:0051020
243	GO:0009411
244	GO:0043535
245	GO:0015938
246	GO:0048846
247	GO:0097057
248	GO:0002064

249	GO:0060411
250	hsa04115
251	GO:0005678
252	GO:0045545
253	hsa05216
254	GO:0004515
255	GO:0050920
256	GO:2000054
257	GO:0042993
258	GO:0015014
259	GO:0035330
260	GO:0034644
261	GO:0051668
262	GO:0021615
263	GO:0071168
264	GO:0051894
265	GO:0021612
266	GO:2000500
267	GO:0060074
268	GO:0010717
269	GO:0010957
270	GO:0071559
271	GO:0043015
272	GO:0035623
273	GO:0045793
274	GO:0045578
275	GO:0090141
276	GO:0043220
277	GO:0072302
278	GO:0031134
279	GO:0051385
280	GO:0021602
281	GO:0032862
282	GO:0032090
283	GO:0046546
284	GO:0003140
285	GO:0021782
286	GO:0045621
287	GO:0035732
288	GO:0035117
289	GO:0018205
290	GO:0060197
291	GO:0060023
292	GO:0060997
293	GO:0009008

294	GO:0045899
295	GO:0051895
296	GO:0002309
297	GO:0032204
298	GO:0008191
299	GO:0008585
300	GO:0031931
301	GO:0010506
302	GO:0043433
303	GO:0035907
304	GO:0016314
305	GO:0030891
306	GO:0000791
307	GO:0001893
308	GO:0009954
309	GO:0032137
310	GO:0007406
311	GO:0060421
312	GO:0043653
313	GO:0043409
314	GO:0042512
315	hsa05223
316	GO:0090179
317	GO:0034332
318	GO:0007417
319	GO:0006907
320	GO:0060547
321	GO:0002041
322	GO:2000270
323	GO:0006275
324	GO:0046983
325	GO:0004450
326	GO:0010485
327	GO:0001825
328	GO:0048702
329	GO:0046329
330	GO:0071391
331	GO:0071681
332	GO:0045950
333	GO:0061308
334	GO:0048935
335	GO:0030833
336	GO:0031658
337	GO:0051973
338	GO:0045842

339	GO:0001667
340	GO:0009132
341	GO:0030856
342	GO:0006309
343	GO:0051606
344	GO:0071773
345	GO:0071456
346	GO:0004704
347	GO:0051057
348	hsa05213
349	GO:0008480
350	GO:0070664
351	GO:0060429
352	GO:0048630
353	GO:0043276
354	GO:0060492
355	GO:0031226
356	GO:0032407
357	GO:0047485
358	GO:0007422
359	GO:0061032
360	GO:2001047
361	GO:0007141
362	GO:2001220
363	GO:0022612
364	GO:0071901
365	GO:0031461
366	GO:0010942
367	GO:0071922
368	GO:0051414
369	GO:0060876
370	GO:0090071
371	GO:0061156
372	GO:0008209
373	GO:0035988
374	GO:0034124
375	GO:0005913
376	GO:0046356
377	GO:0007569
378	GO:0004860
379	GO:0006469
380	GO:0002762
381	GO:0045732
382	GO:0045569
383	GO:0051797

384	GO:0070026
385	GO:0045606
386	GO:0010216
387	GO:0090344
388	GO:0060662
389	GO:0031648
390	GO:0010761
391	GO:0097066
392	GO:0003183
393	GO:0046930
394	GO:0008340
395	GO:0001047
396	GO:0042448
397	GO:0072001
398	GO:0010975
399	GO:0035329
400	GO:0042524
401	GO:0035385
402	GO:2000008
403	GO:0045069
404	GO:0050509
405	GO:2000278
406	GO:0046544
407	GO:0030514
408	GO:0042633
409	GO:0032535
410	GO:0051365
411	GO:0031462
412	GO:0071158
413	GO:0007634
414	GO:0008432
415	GO:2000647
416	GO:0006417
417	GO:0032835
418	GO:0006983
419	GO:0090230
420	GO:0010621
421	GO:0000904
422	GO:0035731
423	GO:0051220
424	GO:0032880
425	GO:0043523
426	GO:0070853
427	GO:0021983
428	GO:0019985

429	GO:0006464
430	GO:0045737
431	GO:0022011
432	GO:0030704
433	GO:0038028
434	GO:0003281
435	GO:0010484
436	GO:0044346
437	GO:0001031
438	GO:0060324
439	GO:0003886
440	GO:2000808
441	GO:1900103
442	GO:0021877
443	GO:0007403
444	GO:0034769
445	GO:0030855
446	GO:0030690
447	GO:0000080
448	GO:0035441
449	GO:0000712
450	GO:0042117
451	GO:0048859
452	GO:0007530
453	GO:0034614
454	GO:0042328
455	GO:0021599
456	GO:0048853
457	GO:0005068
458	GO:0048263
459	GO:2000564
460	GO:0042483
461	GO:0000279
462	GO:0048523
463	GO:0060318
464	GO:0008629
465	GO:0060575
466	GO:0042766
467	GO:0090394
468	GO:0070318
469	GO:0010389
470	GO:0071336
471	GO:0035921
472	hsa05200
473	GO:0032389

474	GO:2001022
475	GO:0002009
476	GO:0060711
477	GO:0060769
478	GO:0021546
479	GO:0009890
480	GO:0070052
481	GO:0035264
482	GO:0045814
483	GO:0060385
484	GO:0001836
485	GO:0090191
486	GO:0050847
487	GO:0043616
488	GO:0051800
489	GO:0051252
490	GO:0006278
491	GO:0019912
492	GO:0033692
493	GO:0021779
494	GO:0032321
495	GO:0033327
496	GO:0031265
497	GO:0060022
498	GO:0035021
499	GO:0048048
500	GO:0006627

(5) Dataset S_5

a) MaxRel features list

Rank	Feature name
1	hsa05219
2	GO:0051146
3	GO:0007050
4	GO:0050680
5	hsa05200
6	hsa05218
7	GO:0051717
8	GO:0051800
9	GO:0033032
10	GO:0097107
11	GO:0031658
12	GO:0090071
13	GO:0032535
14	GO:2000808

15	hsa05220
16	hsa05216
17	hsa05213
18	GO:0016314
19	hsa05210
20	GO:0050821
21	GO:0007092
22	GO:0032286
23	GO:0008285
24	hsa05215
25	GO:0071158
26	hsa05212
27	GO:0030336
28	GO:0071456
29	GO:0042127
30	hsa05223
31	GO:0033601
32	GO:0090403
33	GO:0060074
34	GO:0045792
35	GO:0060179
36	GO:0071681
37	GO:0002902
38	hsa05221
39	GO:0090398
40	GO:0043066
41	GO:0090394
42	GO:0033561
43	hsa04115
44	GO:0051726
45	hsa05214
46	GO:0014010
47	GO:0070141
48	GO:0045475
49	GO:0004861
50	GO:0030308
51	GO:0071479
52	GO:0032794
53	GO:0010997
54	GO:0033596
55	GO:0032355
56	GO:0042326
57	GO:0007569
58	GO:0071850
59	GO:0008283

60	GO:0001934
61	GO:0055105
62	GO:0048147
63	GO:0060749
64	GO:0006469
65	GO:0048546
66	GO:0019901
67	GO:2000379
68	GO:0043234
69	GO:0008284
70	GO:0031571
71	GO:0060024
72	GO:0042771
73	GO:0002053
74	GO:0007265
75	GO:0044430
76	GO:0001889
77	GO:0022408
78	GO:0008629
79	GO:0001829
80	GO:0001570
81	GO:0016342
82	hsa05166
83	GO:0010629
84	GO:0031575
85	GO:0031100
86	GO:2000008
87	GO:0043065
88	GO:0010165
89	GO:2000278
90	GO:0019912
91	GO:0031647
92	GO:0045893
93	GO:0033077
94	GO:0060709
95	GO:2001047
96	GO:0048146
97	GO:0006917
98	GO:0043433
99	GO:0033598
100	GO:0060479
101	GO:0048262
102	GO:0035112
103	GO:0030997
104	GO:0045736

105	GO:0007507
106	GO:0019002
107	GO:0001701
108	GO:0005072
109	GO:0042493
110	GO:0001836
111	GO:0045667
112	GO:0070245
113	GO:0010628
114	GO:0008543
115	GO:0070215
116	GO:0001952
117	GO:0043220
118	GO:0071364
119	GO:0045930
120	GO:0072079
121	GO:0019903
122	GO:0060644
123	GO:0060770
124	GO:0005737
125	GO:0008013
126	GO:0043627
127	GO:0050679
128	GO:0071285
129	GO:0043542
130	GO:0071407
131	GO:0042802
132	GO:0032880
133	GO:0042058
134	GO:0030335
135	GO:0001047
136	GO:0007281
137	GO:0045765
138	GO:0000122
139	GO:0048145
140	GO:0000165
141	GO:0019899
142	GO:0071930
143	GO:0045597
144	GO:0034750
145	GO:0046825
146	GO:0009314
147	GO:0044334
148	GO:0070369
149	GO:0010909

150	GO:0072033
151	GO:0072053
152	GO:0072054
153	hsa05222
154	GO:0007435
155	GO:0030097
156	GO:0051412
157	GO:0010243
158	GO:0008134
159	GO:0061324
160	GO:0019538
161	GO:0035264
162	GO:0045295
163	GO:0000186
164	GO:0007406
165	GO:0010332
166	GO:0005515
167	GO:0006978
168	hsa05211
169	GO:0033673
170	GO:0035802
171	GO:0003156
172	GO:0043524
173	GO:0045892
174	GO:0030858
175	GO:0072182
176	GO:0051898
177	GO:0043154
178	GO:0045786
179	GO:0000279
180	GO:0090230
181	GO:0007417
182	GO:0043280
183	GO:0048538
184	GO:0031134
185	GO:0045445
186	GO:0045732
187	GO:0030879
188	GO:0051000
189	GO:0051895
190	GO:0048745
191	GO:0043535
192	hsa04012
193	GO:0030325
194	GO:0030511

195	GO:0001085
196	GO:0055100
197	GO:0010975
198	GO:0034613
199	GO:0006407
200	GO:0007420
201	GO:0043434
202	GO:0045578
203	GO:0048589
204	GO:0031670
205	GO:0051668
206	GO:0070412
207	GO:0061002
208	GO:0036023
209	GO:0001077
210	GO:0004716
211	GO:0045944
212	GO:0046982
213	GO:0007369
214	GO:0022601
215	GO:0033138
216	GO:0014070
217	GO:0048565
218	GO:0030307
219	GO:0033088
220	GO:0030334
221	GO:0001933
222	GO:0032228
223	GO:0045768
224	GO:0008634
225	GO:0004438
226	GO:0060923
227	GO:0045668
228	GO:0072134
229	GO:0043281
230	GO:0030178
231	GO:0035189
232	GO:0043652
233	GO:0006915
234	GO:0042803
235	hsa04520
236	GO:0048011
237	GO:0014068
238	GO:0031648
239	GO:0051492

240	GO:0072332
241	GO:0072133
242	GO:0001707
243	GO:0001776
244	GO:0032993
245	GO:0051444
246	GO:0019904
247	GO:0006921
248	GO:0032869
249	GO:0005924
250	GO:0038028
251	GO:0016328
252	GO:0046022
253	GO:0007179
254	GO:0090096
255	GO:0044346
256	GO:2001220
257	GO:0032570
258	GO:0044212
259	GO:0034088
260	GO:0008633
261	GO:0071157
262	GO:0071922
263	GO:0031625
264	hsa04350
265	GO:0016310
266	GO:0031065
267	GO:0048705
268	GO:0043525
269	GO:0048733
270	GO:0010468
271	GO:0034644
272	hsa04010
273	GO:0001658
274	GO:0006919
275	GO:0002326
276	GO:0034742
277	GO:0070372
278	GO:0033687
279	GO:0002039
280	GO:0065004
281	GO:0008637
282	GO:0051097
283	GO:0045444
284	GO:0045595

285	hsa04320
286	GO:0048102
287	GO:0051894
288	GO:0035022
289	GO:0005667
290	GO:0090344
291	GO:0008630
292	GO:0060716
293	GO:0001656
294	GO:0043409
295	GO:0006468
296	GO:0010800
297	GO:0051153
298	GO:0045600
299	GO:0048742
300	GO:0001953
301	GO:0071363
302	GO:0034747
303	GO:0031069
304	GO:0007060
305	GO:0043060
306	GO:0051257
307	GO:0043296
308	GO:0003690
309	GO:0000320
310	GO:0090200
311	GO:0050681
312	GO:0048015
313	GO:0010033
314	GO:0043406
315	GO:0090400
316	GO:0043550
317	GO:0046777
318	GO:0014067
319	GO:0060433
320	GO:0009887
321	GO:0030856
322	GO:0023014
323	GO:0044336
324	GO:0045884
325	GO:0005712
326	GO:0032390
327	GO:2000134
328	GO:0045931
329	GO:0061031

330	GO:0035033
331	GO:0010718
332	GO:2000054
333	GO:0001525
334	GO:0030857
335	GO:0048859
336	GO:0046600
337	GO:0045596
338	GO:0007173
339	GO:0010595
340	GO:0048714
341	GO:0030900
342	GO:0001569
343	GO:0051318
344	GO:0007346
345	GO:0070512
346	GO:2000620
347	GO:2000617
348	GO:0045950
349	GO:0006461
350	GO:0001938
351	GO:0001102
352	GO:0090090
353	GO:0002309
354	hsa04110
355	GO:0048320
356	GO:0009612
357	GO:0045165
358	GO:0051893
359	GO:0060789
360	GO:0008286
361	GO:0003908
362	GO:0043508
363	GO:0046983
364	GO:0060441
365	GO:0043009
366	GO:0001954
367	GO:0071559
368	GO:0021983
369	GO:0032461
370	GO:0060769
371	GO:0060916
372	GO:0042060
373	GO:0030235
374	GO:0000739

375	GO:0000733
376	GO:0033689
377	GO:0043006
378	GO:0031668
379	GO:0006309
380	GO:0043491
381	GO:0009636
382	GO:0032204
383	GO:0001837
384	GO:0060440
385	GO:0042327
386	GO:0009790
387	GO:0003382
388	GO:0009898
389	GO:0055096
390	GO:0000307
391	GO:0033600
392	GO:0032318
393	GO:0060070
394	GO:0060177
395	GO:0002052
396	GO:0000790
397	GO:0018105
398	GO:0030275
399	GO:0042992
400	GO:0043392
401	GO:0070557
402	GO:2000653
403	GO:0044029
404	GO:0060421
405	GO:0016604
406	GO:0072112
407	GO:0001822
408	GO:2000195
409	GO:0030521
410	GO:0051797
411	GO:0042153
412	GO:0071230
413	GO:2000060
414	GO:0045787
415	GO:0032956
416	GO:0031226
417	GO:0060529
418	GO:0072277
419	GO:0051781

420	GO:0006916
421	GO:2001076
422	GO:0007356
423	GO:0035021
424	GO:0046621
425	GO:0001657
426	GO:0051091
427	GO:0048318
428	GO:0030324
429	GO:0070888
430	GO:0021847
431	GO:0021769
432	GO:0035607
433	GO:0045793
434	GO:0016477
435	GO:0030616
436	GO:0033326
437	GO:0031436
438	GO:0090343
439	GO:0038085
440	GO:0042551
441	GO:0001756
442	GO:0001649
443	GO:0016301
444	GO:0004713
445	GO:0045740
446	GO:0007403
447	GO:0042663
448	GO:0035026
449	GO:0035441
450	hsa04722
451	GO:0022405
452	GO:0010942
453	GO:0017015
454	GO:0001706
455	GO:0060736
456	GO:0003007
457	GO:0051365
458	GO:0071459
459	GO:0031122
460	GO:0042518
461	GO:0004714
462	GO:0003700
463	GO:0034097
464	GO:0061047

465	GO:0000080
466	GO:0060687
467	GO:0045685
468	GO:0021897
469	GO:0034504
470	GO:0060197
471	GO:0022898
472	GO:0048743
473	GO:0014042
474	GO:2001234
475	GO:0032848
476	GO:0048753
477	GO:0006808
478	GO:0043375
479	GO:0032091
480	GO:0007422
481	GO:0048870
482	GO:0043923
483	GO:0030224
484	GO:0010719
485	GO:0007398
486	GO:0048853
487	GO:0046677
488	GO:0043219
489	GO:0060439
490	GO:0060449
491	GO:0060021
492	GO:0042981
493	GO:0003406
494	GO:0046639
495	GO:0016539
496	hsa04310
497	GO:0051385
498	GO:0021747
499	GO:0072166
500	GO:0001042

b) mRMR features list

Rank	Feature name
1	hsa05219
2	GO:0030695
3	GO:0022601
4	GO:0090403
5	GO:0033673
6	GO:0045295

7	GO:0003908
8	GO:0016314
9	GO:0035802
10	GO:0071364
11	GO:0033596
12	GO:0051153
13	GO:0051146
14	GO:0016151
15	GO:0048147
16	GO:0051444
17	GO:0001829
18	GO:0042518
19	GO:0048745
20	GO:0032794
21	GO:0033601
22	GO:0035694
23	GO:0030215
24	GO:0050821
25	GO:0016918
26	GO:0014003
27	GO:0042771
28	GO:0070372
29	GO:0050808
30	GO:0050680
31	GO:0045792
32	GO:0070215
33	GO:0055100
34	GO:0048102
35	GO:0071481
36	GO:0035441
37	GO:0042271
38	GO:0001952
39	GO:0002070
40	GO:0010424
41	GO:0051895
42	GO:0019538
43	GO:0031575
44	GO:0090200
45	GO:0051893
46	GO:0046825
47	GO:0043666
48	GO:0010165
49	GO:0021569
50	GO:0071157
51	GO:0047710

52	GO:0060716
53	GO:0071681
54	GO:0071385
55	GO:0007050
56	GO:0006307
57	GO:0044430
58	GO:0030336
59	GO:0070245
60	GO:0042551
61	GO:0007092
62	GO:0030852
63	GO:0050508
64	GO:0030325
65	GO:0060770
66	GO:0031665
67	GO:0043508
68	GO:0090398
69	GO:0031462
70	GO:0001707
71	GO:0042058
72	GO:0043296
73	GO:0003156
74	GO:0051492
75	GO:0071158
76	GO:0048546
77	GO:0032090
78	GO:0045884
79	GO:0072332
80	GO:0008634
81	GO:0045606
82	GO:0004861
83	GO:0043652
84	GO:0009987
85	GO:0015964
86	GO:0045578
87	GO:0035189
88	GO:0007162
89	GO:0006407
90	GO:0060074
91	GO:0072498
92	GO:0007060
93	GO:0001570
94	GO:0045604
95	GO:0034613
96	GO:0090343

97	GO:0004716
98	GO:0033326
99	GO:0043535
100	GO:2000310
101	GO:0031670
102	GO:0048742
103	GO:0060749
104	GO:0035414
105	GO:0043220
106	GO:0000819
107	GO:0033235
108	GO:0033687
109	GO:0014010
110	GO:0046718
111	hsa05220
112	GO:0060023
113	GO:0032461
114	GO:0031226
115	GO:0010997
116	GO:0042663
117	GO:0010506
118	GO:0016538
119	GO:0048733
120	GO:2000041
121	GO:0016342
122	GO:0031571
123	GO:0060876
124	GO:0019210
125	GO:0032930
126	GO:0042326
127	GO:0010975
128	GO:0065004
129	GO:0051894
130	GO:2001241
131	GO:0017148
132	GO:0055105
133	GO:0043015
134	GO:0045732
135	GO:0007265
136	GO:0006776
137	GO:0060997
138	GO:0042406
139	GO:0033561
140	GO:0002309
141	GO:0048702

142	GO:0043060
143	GO:0051365
144	GO:0001953
145	GO:2000119
146	GO:2001076
147	GO:0071479
148	GO:0009826
149	GO:0007435
150	GO:0043550
151	GO:2000134
152	GO:0032956
153	GO:0001776
154	GO:0046851
155	GO:0046022
156	GO:0032228
157	GO:0010637
158	GO:0061031
159	GO:0050847
160	GO:0071456
161	GO:0015014
162	GO:0030030
163	GO:0007422
164	GO:0051782
165	GO:0060024
166	GO:0030178
167	GO:0031065
168	GO:0016339
169	GO:0035264
170	GO:0005501
171	GO:0035791
172	GO:0007090
173	GO:0072015
174	GO:0035330
175	GO:2000273
176	GO:0051721
177	GO:0021612
178	hsa04115
179	GO:0043281
180	GO:0051898
181	GO:0051171
182	GO:0016601
183	GO:0000739
184	GO:0060923
185	hsa05221
186	GO:0060206

187	GO:2000117
188	GO:0004438
189	GO:0045793
190	GO:0070483
191	GO:0051257
192	GO:0060465
193	GO:0046329
194	GO:0035022
195	GO:0001656
196	GO:0031647
197	GO:0070026
198	GO:0005737
199	GO:0038085
200	GO:0071279
201	GO:0034742
202	GO:0019841
203	GO:0005072
204	GO:0008330
205	GO:0051097
206	GO:0045444
207	GO:0021754
208	GO:0033692
209	GO:0043542
210	GO:2000008
211	GO:0070534
212	GO:0044345
213	GO:0033088
214	GO:0032007
215	GO:0040008
216	GO:0007356
217	GO:0045475
218	GO:0051385
219	GO:0031398
220	GO:0031668
221	GO:0071930
222	GO:0035088
223	GO:0030111
224	GO:0070141
225	GO:0006306
226	GO:0051973
227	GO:0043409
228	GO:0060346
229	GO:0001836
230	GO:0032862
231	GO:0060706

232	GO:2000379
233	GO:0016328
234	GO:0045569
235	GO:0043570
236	GO:0000733
237	hsa05218
238	GO:0045837
239	GO:0004415
240	GO:0032938
241	GO:0060512
242	GO:0010243
243	GO:0004721
244	GO:0036120
245	GO:0031648
246	GO:2000080
247	GO:0005712
248	GO:0005913
249	hsa05216
250	GO:0021599
251	GO:0042328
252	GO:0090394
253	hsa00100
254	GO:0071850
255	GO:0071559
256	GO:0051400
257	GO:0003214
258	GO:0033598
259	GO:0070664
260	GO:0060709
261	GO:0007417
262	GO:0043517
263	GO:0042473
264	GO:0051606
265	GO:0032870
266	GO:0060070
267	GO:0007569
268	GO:0034614
269	GO:0071391
270	GO:0055096
271	GO:0051726
272	GO:0030506
273	GO:0060711
274	GO:0060599
275	GO:0046580
276	GO:0051717

277	GO:0034244
278	GO:0032321
279	GO:0032869
280	GO:0032300
281	GO:0001047
282	GO:2000271
283	GO:0006469
284	GO:0050509
285	GO:0007634
286	GO:0072133
287	hsa05213
288	GO:0006513
289	GO:0030704
290	GO:0030578
291	GO:0005019
292	GO:0034088
293	GO:0048712
294	GO:0043280
295	GO:0046621
296	GO:2000195
297	GO:0007406
298	GO:0007098
299	GO:0051668
300	GO:0042117
301	GO:0061428
302	GO:0030858
303	GO:0035731
304	GO:0032204
305	GO:0001825
306	hsa05223
307	GO:2000052
308	GO:0009792
309	GO:0001835
310	GO:0051800
311	GO:0033085
312	GO:0034124
313	GO:0070052
314	GO:0010907
315	GO:0000188
316	GO:0070888
317	GO:0030833
318	GO:0042483
319	GO:0034644
320	GO:0008595
321	GO:0061003

322	GO:0032390
323	GO:0007346
324	GO:0002902
325	GO:0071901
326	GO:0030857
327	GO:0090246
328	GO:0072302
329	GO:0090141
330	GO:0042992
331	GO:0035117
332	GO:0008656
333	GO:0043045
334	GO:0019208
335	GO:0033600
336	GO:0042327
337	GO:0060840
338	GO:0070557
339	GO:0045296
340	GO:0007281
341	GO:0071679
342	GO:0007270
343	GO:0070318
344	GO:0008340
345	GO:2000035
346	GO:0010522
347	GO:0006978
348	GO:0048705
349	GO:0035730
350	GO:2001047
351	GO:0060420
352	GO:0033032
353	GO:0019215
354	GO:0021570
355	GO:0070059
356	GO:0072112
357	GO:0071670
358	GO:0009008
359	GO:0010464
360	GO:0017017
361	GO:0046546
362	GO:0006921
363	GO:0046983
364	GO:0007185
365	GO:0048074
366	GO:0010389

367	GO:0044342
368	GO:0060340
369	GO:0097107
370	GO:2000836
371	GO:0060644
372	GO:0031466
373	GO:0007398
374	GO:0000228
375	GO:0035021
376	GO:0048014
377	GO:0005006
378	GO:2001244
379	GO:0051271
380	GO:0097057
381	GO:0010717
382	GO:0060429
383	GO:0019002
384	GO:0016327
385	GO:2001022
386	GO:0048638
387	GO:0060216
388	GO:0033689
389	GO:0007416
390	GO:0047485
391	GO:0031658
392	GO:0002041
393	GO:0021892
394	GO:0034405
395	GO:0043518
396	GO:0030511
397	GO:0031307
398	GO:0035033
399	GO:0072166
400	GO:0022408
401	GO:0090175
402	GO:0050920
403	GO:0045950
404	GO:0005017
405	GO:0003714
406	GO:0023014
407	GO:0016015
408	GO:0009411
409	GO:0051000
410	GO:0061308
411	GO:0003382

412	GO:0002762
413	GO:0032286
414	GO:0035732
415	GO:0043433
416	GO:0031235
417	GO:0006309
418	GO:0031122
419	GO:0006417
420	GO:0060347
421	GO:0045842
422	GO:2000484
423	GO:0043219
424	GO:0035026
425	GO:2000054
426	GO:0032211
427	GO:0008064
428	GO:2000500
429	hsa05210
430	GO:0048160
431	GO:0001077
432	GO:0097105
433	GO:0045762
434	GO:0070507
435	GO:0001783
436	GO:0007091
437	GO:0035924
438	GO:0010719
439	GO:0001893
440	GO:0060547
441	GO:0090071
442	GO:0060197
443	GO:0072284
444	GO:0006917
445	GO:0060981
446	GO:0060220
447	GO:0050897
448	GO:0016310
449	GO:0016514
450	GO:0071773
451	GO:0000422
452	GO:0051412
453	GO:0030334
454	GO:0042054
455	GO:0097162
456	GO:0060437

457	GO:0008629
458	GO:0021602
459	GO:2000045
460	GO:0050431
461	GO:0032137
462	GO:0072134
463	GO:0097120
464	GO:0032535
465	GO:0055093
466	GO:0048858
467	GO:0043009
468	GO:0022029
469	GO:0009890
470	GO:0045736
471	GO:0042108
472	GO:0060807
473	GO:0045667
474	GO:0045656
475	GO:2000270
476	GO:0050772
477	GO:0006346
478	GO:0007497
479	GO:0043497
480	GO:0048714
481	GO:0043923
482	GO:0005979
483	GO:0048738
484	GO:0045184
485	GO:0035121
486	GO:2000808
487	GO:0006473
488	GO:0045726
489	GO:0043434
490	GO:0000239
491	GO:0010754
492	GO:0051020
493	GO:0050732
494	GO:0000785
495	GO:0048859
496	hsa05200
497	GO:0035907
498	GO:0072175
499	GO:0035329
500	GO:0038091

(6) Dataset S_6

a) MaxRel features list

Rank	Feature name
1	GO:0051146
2	hsa05219
3	hsa05218
4	hsa05216
5	GO:0051717
6	GO:0033032
7	GO:0031658
8	GO:0090071
9	GO:0032535
10	GO:0051800
11	GO:0097107
12	GO:2000808
13	GO:0014010
14	GO:0060179
15	GO:0032286
16	GO:0060074
17	GO:0050680
18	hsa05200
19	GO:0007092
20	GO:0016314
21	GO:0007050
22	GO:0010997
23	GO:0045792
24	hsa05220
25	hsa05213
26	GO:0008283
27	GO:0042127
28	GO:0002902
29	GO:0090394
30	GO:0060024
31	hsa05215
32	hsa05212
33	hsa05210
34	GO:0090398
35	GO:0071456
36	GO:0008285
37	GO:0070141
38	GO:0072182
39	GO:0050821
40	GO:0090403
41	GO:0043066
42	GO:2001047

43	GO:0060709
44	GO:0045475
45	hsa05214
46	hsa05223
47	GO:0071158
48	GO:0071930
49	GO:0035112
50	GO:0060479
51	GO:0048262
52	GO:0030997
53	GO:0030336
54	GO:0071681
55	GO:0061324
56	GO:0033561
57	GO:0001889
58	GO:0045884
59	GO:0001934
60	GO:0033601
61	hsa05221
62	GO:0061002
63	GO:0044334
64	GO:0070369
65	GO:0010909
66	GO:0032355
67	GO:0072079
68	GO:0030308
69	GO:0048745
70	GO:0048145
71	GO:0008284
72	GO:0043542
73	GO:0050679
74	GO:0034750
75	GO:0051412
76	GO:0007403
77	GO:0031575
78	GO:0001570
79	GO:0045930
80	GO:0036023
81	GO:0007281
82	GO:0072033
83	GO:0071850
84	GO:0019899
85	GO:0072053
86	GO:0072054
87	GO:0042129

88	GO:0043220
89	GO:0002053
90	GO:0048146
91	GO:0006469
92	GO:0045765
93	GO:0060769
94	GO:0061047
95	GO:0019912
96	GO:2000278
97	GO:0007569
98	GO:0004861
99	GO:0042493
100	GO:2000379
101	GO:0044336
102	GO:0016342
103	GO:0022408
104	GO:0043234
105	GO:0031999
106	GO:0004438
107	GO:0071479
108	GO:0022405
109	GO:0048546
110	GO:0070245
111	GO:0035189
112	GO:0010629
113	GO:0071364
114	GO:0043550
115	GO:0060749
116	GO:0031069
117	GO:0071407
118	GO:0051782
119	GO:0043281
120	GO:0055105
121	GO:0051726
122	GO:0005924
123	GO:0060644
124	GO:0043627
125	GO:0031100
126	GO:0014070
127	GO:0044430
128	GO:0060492
129	GO:0010628
130	GO:0001829
131	GO:0001701
132	GO:0010165

133	GO:0000279
134	GO:0019901
135	GO:0090230
136	GO:0005072
137	GO:0007507
138	GO:0031134
139	GO:0060440
140	GO:0006978
141	GO:0051385
142	GO:0032880
143	GO:0032993
144	GO:0048538
145	hsa05166
146	GO:0070557
147	GO:0048147
148	GO:0033596
149	GO:0008013
150	GO:0060923
151	GO:0022601
152	GO:0060066
153	GO:0030879
154	GO:0010332
155	GO:0042326
156	GO:0000165
157	GO:0035022
158	GO:0033088
159	GO:0031571
160	GO:2000008
161	GO:0033138
162	GO:0043433
163	GO:0030858
164	GO:0045445
165	GO:0045893
166	GO:0043535
167	hsa04115
168	GO:0045736
169	GO:0045768
170	GO:0007435
171	GO:0032570
172	GO:0003338
173	GO:0031647
174	GO:0003136
175	GO:0042771
176	GO:0008543
177	GO:0045667

178	GO:0001658
179	GO:0071157
180	GO:0048853
181	GO:0032228
182	GO:0071285
183	GO:0033598
184	GO:0008637
185	GO:0071922
186	GO:0032794
187	GO:0051894
188	GO:0033077
189	GO:0016310
190	GO:0042802
191	GO:0071363
192	GO:0046825
193	GO:0051097
194	GO:0048565
195	GO:0045578
196	hsa05222
197	GO:0019538
198	GO:0001656
199	hsa05211
200	GO:0043524
201	GO:0034088
202	GO:0030307
203	GO:2000134
204	GO:0048859
205	GO:0031670
206	GO:0043006
207	GO:0072112
208	GO:0043154
209	GO:0038085
210	GO:0001503
211	GO:0030856
212	GO:0007417
213	GO:0009314
214	GO:0042524
215	GO:0010975
216	GO:0010243
217	GO:0008629
218	GO:0030511
219	GO:0010033
220	GO:0019002
221	GO:0034613
222	GO:0030325

223	GO:0051492
224	GO:0043652
225	GO:0001707
226	GO:0008134
227	GO:0007406
228	GO:0051000
229	GO:0030539
230	GO:0043434
231	GO:0035802
232	GO:0003156
233	GO:0030097
234	GO:0090400
235	GO:2000017
236	GO:0009636
237	GO:0042992
238	GO:0019903
239	GO:0048589
240	GO:0003382
241	GO:0010468
242	GO:2000045
243	GO:0048015
244	GO:0032204
245	GO:0070602
246	GO:0060916
247	GO:0051318
248	GO:0090344
249	GO:0061198
250	GO:0030335
251	GO:0005515
252	GO:0046982
253	GO:0070215
254	GO:0043508
255	GO:0010564
256	GO:0045668
257	GO:0090096
258	GO:0044346
259	GO:2001220
260	GO:0001047
261	GO:0045786
262	GO:0043280
263	GO:0051898
264	GO:0071504
265	GO:0001938
266	GO:0043491
267	GO:0051668

268	GO:0045787
269	GO:0000904
270	GO:0003690
271	GO:0000122
272	GO:0001525
273	GO:0072133
274	GO:0072134
275	GO:0010942
276	GO:0030900
277	GO:0019211
278	GO:0051895
279	GO:0071559
280	GO:0065004
281	GO:0071459
282	GO:0034504
283	GO:0090343
284	GO:0034644
285	GO:0035749
286	GO:0045892
287	hsa04350
288	GO:0043065
289	GO:0060599
290	GO:0060770
291	GO:0051591
292	GO:0035117
293	GO:0043616
294	GO:0022009
295	GO:0001657
296	GO:0051444
297	GO:0042803
298	GO:0001952
299	GO:0044212
300	GO:0009887
301	GO:0060433
302	GO:0001837
303	GO:2000060
304	GO:0060687
305	GO:0030324
306	GO:0007595
307	GO:0045595
308	hsa04520
309	GO:0045656
310	GO:0035264
311	GO:0007265
312	GO:0070412

313	GO:0031253
314	GO:0042153
315	hsa04012
316	GO:0005006
317	GO:0034742
318	GO:0048660
319	GO:0071230
320	GO:0002052
321	GO:0045944
322	GO:0006917
323	GO:0030235
324	GO:0007420
325	GO:0005737
326	GO:0000186
327	GO:0007346
328	GO:0038028
329	GO:0007369
330	GO:0009612
331	GO:0042518
332	GO:0001569
333	GO:0042551
334	GO:0010718
335	GO:0001077
336	GO:0060197
337	GO:0031648
338	GO:0045732
339	GO:0048661
340	GO:0002904
341	GO:0060439
342	GO:0045726
343	GO:0070435
344	GO:0034244
345	GO:0010595
346	GO:0048617
347	GO:0072136
348	GO:0042475
349	GO:0048705
350	GO:0070507
351	GO:0010800
352	GO:0045740
353	GO:0060789
354	GO:0001706
355	GO:0048754
356	GO:0060571
357	GO:0045597

358	GO:0043406
359	GO:0072207
360	GO:0001085
361	GO:0045471
362	GO:0033673
363	GO:0045743
364	GO:0031077
365	GO:2000054
366	GO:0000320
367	GO:0009898
368	GO:0060512
369	GO:0060662
370	GO:0003266
371	GO:0000733
372	GO:0000739
373	GO:0043296
374	GO:0050920
375	GO:0043219
376	GO:0007173
377	GO:0007179
378	GO:0008584
379	GO:0072001
380	GO:0045842
381	GO:0032925
382	GO:0051897
383	GO:0060716
384	GO:0007090
385	GO:0007398
386	GO:0045165
387	GO:0001822
388	GO:0005667
389	GO:0060748
390	GO:0006921
391	GO:0034405
392	GO:0035021
393	GO:0043923
394	GO:0043666
395	GO:0001102
396	GO:0043009
397	GO:0048715
398	GO:0060021
399	GO:0009790
400	GO:0048702
401	GO:0045669
402	GO:0032869

403	GO:0097105
404	GO:0004716
405	GO:0001836
406	GO:0070372
407	GO:0045671
408	GO:0046621
409	GO:0007270
410	GO:0016477
411	GO:0045295
412	GO:0016328
413	GO:0031642
414	GO:0070491
415	GO:0032967
416	GO:0010719
417	GO:0051091
418	GO:0046983
419	GO:0048557
420	GO:0060742
421	GO:0051893
422	GO:0060449
423	GO:0001649
424	GO:0021955
425	GO:0048733
426	GO:0033235
427	GO:0070374
428	GO:0045600
429	GO:0008286
430	GO:0002309
431	GO:0032091
432	GO:0060529
433	GO:0033085
434	GO:0033600
435	GO:0001843
436	GO:0055096
437	GO:0000578
438	GO:0042058
439	GO:0014068
440	GO:0030513
441	GO:2000653
442	GO:0044029
443	GO:0002076
444	GO:0061030
445	GO:0034747
446	GO:0090090
447	GO:0000307

448	GO:0030855
449	GO:0033687
450	GO:0033688
451	GO:0030178
452	GO:0060441
453	GO:0001953
454	GO:0001702
455	GO:0035441
456	GO:0045444
457	GO:0048701
458	GO:0055100
459	GO:0060421
460	GO:0060290
461	GO:0003214
462	GO:0030334
463	GO:0060688
464	GO:0006407
465	GO:0030216
466	GO:0034097
467	GO:0060429
468	GO:0050681
469	GO:0031436
470	GO:0031226
471	GO:0032909
472	GO:0031659
473	hsa04010
474	GO:0050678
475	hsa04320
476	GO:0002039
477	GO:0046686
478	GO:2000620
479	GO:2000617
480	GO:0070512
481	GO:0048712
482	GO:0034333
483	GO:0023014
484	GO:0072277
485	GO:0032916
486	GO:0001042
487	GO:0060209
488	GO:0032318
489	GO:0001776
490	GO:0046639
491	GO:0016539
492	GO:0035035

493	GO:0051153
494	GO:0048742
495	GO:0046600
496	GO:0014034
497	GO:0048102
498	GO:0070301
499	GO:0045793
500	GO:0003682

b) mRMR features list

Rank	Feature name
1	GO:0051146
2	GO:0005678
3	GO:0022601
4	GO:0045792
5	GO:0051782
6	GO:0003908
7	GO:0014010
8	GO:0048745
9	GO:0033596
10	GO:0033235
11	GO:0035802
12	GO:0043508
13	GO:0060024
14	GO:0070141
15	GO:0071157
16	GO:0019211
17	GO:0031575
18	GO:0016918
19	GO:0051893
20	GO:0019538
21	GO:2000304
22	GO:0070245
23	GO:0001570
24	GO:0048102
25	GO:0042518
26	GO:0071364
27	GO:0071930
28	GO:0060346
29	GO:0033601
30	GO:0043666
31	GO:0038085
32	GO:0001656
33	GO:0030100
34	GO:0010997

35	GO:0090398
36	GO:0016342
37	GO:0071456
38	hsa00100
39	GO:0045884
40	GO:0010165
41	GO:0071305
42	GO:0051894
43	GO:0032930
44	GO:0042551
45	GO:0042992
46	GO:0043542
47	GO:0044430
48	GO:0000904
49	GO:0090403
50	GO:0035022
51	GO:0001829
52	GO:0042524
53	GO:0010424
54	GO:0071481
55	GO:0050680
56	GO:2000378
57	GO:0048853
58	GO:0051492
59	GO:0072112
60	GO:0048147
61	GO:0071559
62	GO:0007090
63	GO:0007281
64	GO:0003382
65	GO:0051412
66	GO:0060074
67	GO:0019841
68	GO:0032794
69	GO:0007050
70	GO:0050808
71	GO:0090191
72	GO:0006307
73	hsa05216
74	GO:0001503
75	GO:0090343
76	GO:0031670
77	GO:0055096
78	GO:0050821
79	GO:0043015

80	GO:0014034
81	GO:0050920
82	GO:0055105
83	GO:0000819
84	GO:0060179
85	GO:0003156
86	GO:0051385
87	GO:0043616
88	GO:0046718
89	GO:0043550
90	GO:0061198
91	GO:0060571
92	GO:0043281
93	GO:0033673
94	GO:0050431
95	GO:0032516
96	GO:0006407
97	GO:0070507
98	GO:0042771
99	GO:0045578
100	GO:0043535
101	GO:0016151
102	GO:0016310
103	GO:0030284
104	GO:0005913
105	GO:0051153
106	GO:0060429
107	hsa05218
108	GO:0008191
109	GO:0035189
110	GO:0032925
111	GO:2001047
112	GO:0032228
113	GO:0002309
114	GO:0003214
115	GO:0022405
116	GO:0046825
117	GO:0006346
118	GO:0043220
119	GO:0045765
120	GO:0034644
121	GO:0048702
122	GO:0055100
123	GO:0030414
124	GO:0001952

125	GO:0060923
126	GO:0007092
127	GO:0090175
128	GO:0048545
129	GO:2001022
130	GO:0002042
131	GO:0040008
132	GO:0005006
133	GO:0071850
134	GO:0002009
135	GO:0048859
136	GO:0046426
137	GO:0072498
138	hsa05219
139	GO:0030030
140	GO:0048712
141	GO:0007098
142	GO:0060440
143	GO:2000310
144	GO:2000045
145	GO:0090394
146	GO:0035441
147	GO:0031571
148	GO:0045726
149	GO:0046546
150	GO:0034613
151	GO:0001835
152	GO:0060716
153	GO:0033088
154	GO:0035694
155	GO:0030539
156	GO:0034088
157	GO:0070372
158	GO:0051097
159	GO:0007435
160	GO:0030332
161	GO:0047710
162	GO:0031999
163	GO:0046851
164	GO:0051717
165	GO:0045656
166	GO:0006266
167	GO:0030325
168	GO:0048733
169	GO:0048807

170	GO:0033688
171	GO:0060749
172	GO:0071681
173	GO:0046882
174	GO:0010506
175	GO:0004861
176	GO:0001843
177	GO:0019210
178	GO:0051895
179	GO:0070215
180	GO:0051205
181	GO:0019985
182	GO:0014003
183	GO:0045750
184	GO:0045930
185	GO:0002070
186	GO:0097105
187	GO:0060197
188	GO:0043652
189	GO:0017017
190	GO:0065004
191	GO:0005072
192	GO:2000017
193	GO:0044444
194	GO:0010564
195	GO:0032461
196	GO:0046329
197	GO:0051444
198	GO:0031931
199	GO:0033032
200	GO:0001707
201	GO:0032872
202	GO:0015964
203	GO:0071158
204	GO:2000041
205	GO:0007403
206	GO:0035021
207	GO:0000733
208	GO:0001502
209	GO:0033561
210	GO:0060709
211	GO:0048619
212	GO:0048742
213	GO:0018205
214	GO:0001957

215	GO:0032007
216	GO:0001841
217	GO:0060547
218	GO:0021955
219	GO:0070557
220	GO:0060056
221	hsa05220
222	GO:2000054
223	GO:0060347
224	GO:0031134
225	GO:0060324
226	GO:0010975
227	GO:0016538
228	GO:0042129
229	GO:0038091
230	GO:0006978
231	GO:0005113
232	GO:0010389
233	GO:0051668
234	GO:2000134
235	GO:0060135
236	GO:0072133
237	GO:0044344
238	GO:0045950
239	GO:0007422
240	GO:0009649
241	GO:0032286
242	GO:0030695
243	GO:0021895
244	GO:0010942
245	GO:0032956
246	GO:0007346
247	GO:0003136
248	GO:0007595
249	GO:2001076
250	GO:0000739
251	GO:0010719
252	GO:0031647
253	GO:0050847
254	GO:2000379
255	GO:0071504
256	GO:0045842
257	GO:0048844
258	GO:0070435
259	GO:0006306

260	GO:2001241
261	GO:0035414
262	GO:0031658
263	GO:0006633
264	GO:0033598
265	GO:0009925
266	GO:0019530
267	GO:0008634
268	GO:0048806
269	GO:0050795
270	GO:0060318
271	GO:0043296
272	GO:0033326
273	GO:0035988
274	GO:0051353
275	GO:0007270
276	GO:2000270
277	GO:0072332
278	GO:0035117
279	GO:0071922
280	GO:0001953
281	GO:0071889
282	GO:0060433
283	GO:0060748
284	GO:0090071
285	GO:0071385
286	GO:0034405
287	GO:0043374
288	GO:0007356
289	GO:0035924
290	GO:0060770
291	GO:0007569
292	GO:0048705
293	GO:0042058
294	GO:0004415
295	GO:0060513
296	GO:0035970
297	GO:0030855
298	GO:0032535
299	GO:0043045
300	GO:0042473
301	GO:0060789
302	GO:0051298
303	GO:0043006
304	GO:0090246

305	GO:0031648
306	GO:0032139
307	GO:0050867
308	GO:0048538
309	GO:0046621
310	GO:0032862
311	GO:0045880
312	GO:0030957
313	GO:0005979
314	GO:0060512
315	GO:0042993
316	GO:0007497
317	GO:0043923
318	GO:0072134
319	GO:0051800
320	GO:0016339
321	GO:0032204
322	GO:0032300
323	GO:0060599
324	GO:0000422
325	GO:0000279
326	GO:0050918
327	GO:0036023
328	GO:2000052
329	GO:0019912
330	GO:0030235
331	GO:0007141
332	GO:0006776
333	GO:0071901
334	GO:0072207
335	GO:0009267
336	GO:0097107
337	GO:0008330
338	GO:0060706
339	GO:0031668
340	GO:0048562
341	GO:0061428
342	GO:0060662
343	GO:0006469
344	GO:0007162
345	GO:0005652
346	GO:0045671
347	GO:0045120
348	GO:0030336
349	GO:0008637

350	GO:0004438
351	GO:0045793
352	GO:2000271
353	GO:0030858
354	GO:0002762
355	GO:0090230
356	GO:0031226
357	GO:0031730
358	GO:0060687
359	GO:0046498
360	GO:0043570
361	GO:0010757
362	GO:0060644
363	GO:0032318
364	GO:0002089
365	GO:2000808
366	GO:0045651
367	GO:0003334
368	GO:0051797
369	GO:0048333
370	GO:0070932
371	GO:0010243
372	GO:0044345
373	GO:0021569
374	GO:0060492
375	GO:0010637
376	GO:0010484
377	GO:0010595
378	hsa05223
379	GO:0033687
380	GO:0009636
381	GO:0030122
382	GO:0035326
383	GO:0045475
384	GO:0002360
385	GO:0060421
386	GO:2000080
387	GO:0030216
388	GO:0055008
389	GO:2000278
390	GO:2000060
391	GO:0001889
392	GO:0090102
393	GO:0008160
394	GO:0043653

395	GO:0060769
396	GO:0021860
397	GO:0042117
398	GO:0001893
399	GO:0003197
400	GO:0042069
401	GO:0009987
402	GO:0016314
403	GO:0042326
404	GO:0042271
405	GO:0022408
406	GO:0032390
407	GO:0030837
408	GO:0008595
409	GO:0019207
410	GO:0008283
411	GO:0010507
412	GO:0060413
413	GO:0045685
414	GO:0090096
415	GO:0048638
416	GO:0022009
417	GO:0004860
418	GO:0072166
419	GO:0002902
420	GO:0019898
421	GO:0048546
422	GO:0005720
423	GO:0007406
424	GO:0003094
425	GO:0071391
426	GO:0045617
427	GO:0001667
428	GO:0033158
429	GO:0033593
430	GO:0010811
431	hsa05212
432	GO:0007417
433	GO:0045814
434	GO:0045737
435	GO:0007379
436	GO:0043219
437	GO:0071141
438	GO:0061047
439	GO:0051262

440	GO:0061002
441	GO:0048608
442	GO:0032570
443	GO:0005712
444	GO:0045879
445	GO:0036120
446	GO:0016540
447	GO:0090136
448	GO:0021897
449	GO:0009008
450	GO:0017134
451	GO:0090050
452	hsa05213
453	GO:0060340
454	GO:0021798
455	GO:0071479
456	GO:0010717
457	GO:0032203
458	GO:0055062
459	GO:0030857
460	GO:0060434
461	GO:0051318
462	GO:0061324
463	GO:0010748
464	GO:0006473
465	GO:0051898
466	GO:0035330
467	GO:0044346
468	GO:0034244
469	GO:0008585
470	GO:0000115
471	GO:0042711
472	GO:0048386
473	GO:0034504
474	GO:0030891
475	GO:0031619
476	GO:0033144
477	GO:0034673
478	GO:0060022
479	GO:0032025
480	GO:0010804
481	GO:0051964
482	GO:0010839
483	GO:0005501
484	GO:0033085

485	GO:0009620
486	GO:0051573
487	GO:0045295
488	GO:0045184
489	GO:0061042
490	GO:0043434
491	GO:0043517
492	GO:0004882
493	GO:0051271
494	GO:0034742
495	GO:0071459
496	GO:0010907
497	GO:0043473
498	GO:0032444
499	GO:0050730
500	GO:0009792
