# Supplementary material for: Analysis of Tumor Suppressor Genes Based on Gene Ontology and the KEGG Pathway
Source: PLoS One. 2014 Sep 10;9(9):e107202. doi: 10.1371/journal.pone.0107202 (PMC4160198; doi:10.1371/journal.pone.0107202)
Supplement: Table S3 — List of the SNs, SPs, ACCs and MCCs obtained by IFS and Dagging for each dataset Si. (PDF) [file pone.0107202.s003.pdf]

Table S3. The SNs, SPs, ACCs and MCCs obtained by IFS and Dagging for each dataset S_i.

(1) Dataset S_1

Number of features	SN	SP	ACC	MCC
4	0	1	0.833333	N/A
5	0	1	0.833333	N/A
6	0.053659	0.991545	0.83523	0.134305
7	0.053659	0.998049	0.84065	0.18844
8	0.078049	0.995122	0.842276	0.210501
9	0.089431	0.992846	0.842276	0.214514
10	0.100813	0.991545	0.843089	0.22559
11	0.131707	0.988618	0.845799	0.256987
12	0.134959	0.990244	0.847696	0.270818
13	0.146341	0.988293	0.847967	0.276287
14	0.160976	0.986992	0.849322	0.28963
15	0.162602	0.987967	0.850407	0.296782
16	0.17561	0.987642	0.852304	0.312101
17	0.169106	0.988943	0.852304	0.310438
18	0.170732	0.986016	0.850136	0.297719
19	0.182114	0.982439	0.849051	0.295865
20	0.18374	0.984065	0.850678	0.305241
21	0.177236	0.981789	0.847696	0.286749
22	0.180488	0.982114	0.848509	0.292357
23	0.201626	0.982764	0.852575	0.321568
24	0.186992	0.984715	0.851762	0.31234
25	0.185366	0.982114	0.849322	0.29857
26	0.193496	0.981138	0.849864	0.304553
27	0.195122	0.982114	0.850949	0.310763
28	0.2	0.980813	0.850678	0.311189
29	0.203252	0.980813	0.85122	0.315149
30	0.193496	0.981138	0.849864	0.304553
31	0.219512	0.981138	0.854201	0.335872
32	0.22439	0.978537	0.852846	0.330918
33	0.21626	0.981789	0.854201	0.334815
34	0.209756	0.978537	0.850407	0.313655
35	0.20813	0.978537	0.850136	0.311703
36	0.219512	0.977561	0.85122	0.321373
37	0.204878	0.978862	0.849864	0.309083
38	0.227642	0.977561	0.852575	0.330841
39	0.221138	0.975285	0.849593	0.314571
40	0.237398	0.973659	0.850949	0.327356

41	0.22439	0.975285	0.850136	0.318373
42	0.227642	0.97561	0.850949	0.323369
43	0.239024	0.973659	0.85122	0.329201
44	0.22439	0.978537	0.852846	0.330918
45	0.226016	0.97561	0.850678	0.321484
46	0.234146	0.975935	0.852304	0.33207
47	0.235772	0.974309	0.85122	0.327876
48	0.24065	0.975285	0.852846	0.337012
49	0.242276	0.979187	0.856369	0.353891
50	0.237398	0.975285	0.852304	0.333332
51	0.247154	0.975935	0.854472	0.346734
52	0.258537	0.97626	0.85664	0.360492
53	0.24065	0.975285	0.852846	0.337012
54	0.24065	0.974309	0.852033	0.333409
55	0.265041	0.975285	0.856911	0.363905
56	0.239024	0.973333	0.850949	0.328027
57	0.235772	0.973659	0.850678	0.325505
58	0.24878	0.974309	0.853388	0.342517
59	0.245528	0.973008	0.851762	0.334187
60	0.252033	0.972683	0.852575	0.340268
61	0.263415	0.972683	0.854472	0.352738
62	0.239024	0.977561	0.854472	0.343833
63	0.268293	0.974959	0.857182	0.366206
64	0.268293	0.973333	0.855827	0.360319
65	0.255285	0.973008	0.853388	0.345014
66	0.266667	0.970732	0.853388	0.349458
67	0.271545	0.967805	0.851762	0.344894
68	0.266667	0.971057	0.853659	0.350576
69	0.273171	0.971382	0.855014	0.358685
70	0.260163	0.972358	0.853659	0.348051
71	0.255285	0.968455	0.849593	0.329352
72	0.271545	0.96878	0.852575	0.348116
73	0.261789	0.972358	0.85393	0.349823
74	0.265041	0.96748	0.850407	0.336811
75	0.263415	0.971707	0.853659	0.34931
76	0.261789	0.966829	0.849322	0.331158
77	0.286179	0.972358	0.857995	0.375818
78	0.271545	0.970407	0.85393	0.353594
79	0.286179	0.969431	0.855556	0.365799
80	0.294309	0.969756	0.857182	0.37535
81	0.269919	0.970081	0.853388	0.350742
82	0.278049	0.973008	0.857182	0.369563
83	0.287805	0.96748	0.854201	0.361058

84	0.289431	0.96748	0.854472	0.362756
85	0.281301	0.96878	0.854201	0.3585
86	0.279675	0.971057	0.855827	0.364473
87	0.273171	0.96813	0.852304	0.347705
88	0.284553	0.96813	0.854201	0.359776
89	0.261789	0.970407	0.852304	0.343052
90	0.291057	0.966504	0.85393	0.361298
91	0.276423	0.970407	0.854743	0.3588
92	0.289431	0.968455	0.855285	0.365954
93	0.291057	0.96813	0.855285	0.366577
94	0.279675	0.968455	0.853659	0.355702
95	0.295935	0.968455	0.856369	0.372703
96	0.273171	0.967154	0.851491	0.344514
97	0.282927	0.966829	0.852846	0.353826
98	0.274797	0.968455	0.852846	0.350517
99	0.284553	0.96878	0.854743	0.361925
100	0.279675	0.969106	0.854201	0.357863
101	0.291057	0.964553	0.852304	0.355119
102	0.274797	0.963577	0.84878	0.334929
103	0.269919	0.96878	0.852304	0.34637
104	0.268293	0.968455	0.851762	0.343538
105	0.281301	0.967805	0.853388	0.355282
106	0.274797	0.964878	0.849864	0.33898
107	0.292683	0.96813	0.855556	0.368267
108	0.287805	0.968455	0.855014	0.364256
109	0.281301	0.969431	0.854743	0.36067
110	0.289431	0.967154	0.854201	0.3617
111	0.304065	0.964228	0.854201	0.367535
112	0.302439	0.964553	0.854201	0.366883
113	0.291057	0.966179	0.853659	0.360257
114	0.291057	0.963252	0.85122	0.35109
115	0.282927	0.970732	0.856098	0.366787
116	0.307317	0.963902	0.854472	0.369846
117	0.289431	0.966829	0.85393	0.360649
118	0.284553	0.963577	0.850407	0.345277
119	0.300813	0.965528	0.854743	0.368279
120	0.292683	0.96748	0.855014	0.366141
121	0.305691	0.961301	0.852033	0.360278
122	0.287805	0.963252	0.850678	0.347691
123	0.281301	0.965854	0.851762	0.348981
124	0.313821	0.964553	0.856098	0.378454
125	0.292683	0.965528	0.853388	0.35988
126	0.289431	0.965203	0.852575	0.355464

127	0.291057	0.961301	0.849593	0.345176
128	0.291057	0.961626	0.849864	0.346151
129	0.295935	0.963577	0.852304	0.357158
130	0.291057	0.963252	0.85122	0.35109
131	0.292683	0.964553	0.852575	0.356812
132	0.299187	0.961626	0.85122	0.35458
133	0.297561	0.962276	0.851491	0.354864
134	0.286179	0.963902	0.850949	0.347992
135	0.308943	0.964878	0.855556	0.374534
136	0.284553	0.961626	0.84878	0.339335
137	0.321951	0.963902	0.856911	0.384594
138	0.307317	0.960976	0.852033	0.360967
139	0.325203	0.957073	0.851762	0.367642
140	0.295935	0.961951	0.850949	0.352199
141	0.307317	0.965203	0.855556	0.373902
142	0.304065	0.962602	0.852846	0.362538
143	0.312195	0.961626	0.853388	0.367865
144	0.308943	0.95935	0.850949	0.35783
145	0.287805	0.962276	0.849864	0.344715
146	0.295935	0.964878	0.853388	0.361203
147	0.297561	0.959675	0.849322	0.347113
148	0.279675	0.964878	0.850678	0.344173
149	0.312195	0.964228	0.855556	0.375802
150	0.300813	0.962927	0.852575	0.360194
151	0.308943	0.96065	0.852033	0.361656
152	0.320325	0.960976	0.854201	0.374105
153	0.313821	0.963577	0.855285	0.375435
154	0.305691	0.960976	0.851762	0.359308
155	0.308943	0.960976	0.852304	0.362622
156	0.318699	0.958699	0.852033	0.365798
157	0.304065	0.961626	0.852033	0.35959
158	0.284553	0.962276	0.849322	0.341299
159	0.315447	0.963902	0.855827	0.378076
160	0.312195	0.960976	0.852846	0.365921
161	0.313821	0.961626	0.853659	0.369508
162	0.295935	0.963252	0.852033	0.356158
163	0.318699	0.961301	0.854201	0.373444
164	0.291057	0.962602	0.850678	0.349102
165	0.318699	0.964553	0.856911	0.383357
166	0.317073	0.961626	0.854201	0.372785
167	0.325203	0.960325	0.854472	0.377047
168	0.320325	0.957724	0.851491	0.364623
169	0.299187	0.960325	0.850136	0.350705

170	0.312195	0.956748	0.849322	0.35366
171	0.334959	0.957073	0.853388	0.377295
172	0.302439	0.959675	0.850136	0.352136
173	0.307317	0.960976	0.852033	0.360967
174	0.334959	0.958049	0.854201	0.380077
175	0.320325	0.96	0.853388	0.371221
176	0.317073	0.963252	0.855556	0.377711
177	0.325203	0.954797	0.849864	0.361269
178	0.299187	0.95935	0.849322	0.347841
179	0.317073	0.956098	0.849593	0.356753
180	0.302439	0.963577	0.853388	0.363858
181	0.323577	0.957724	0.852033	0.367874
182	0.320325	0.958374	0.852033	0.36649
183	0.300813	0.962602	0.852304	0.359203
184	0.304065	0.958374	0.849322	0.350023
185	0.336585	0.955122	0.852033	0.373422
186	0.334959	0.958699	0.854743	0.381949
187	0.318699	0.958699	0.852033	0.365798
188	0.308943	0.962927	0.85393	0.368503
189	0.317073	0.958374	0.851491	0.363225
190	0.317073	0.960325	0.853117	0.368915
191	0.317073	0.958374	0.851491	0.363225
192	0.313821	0.956423	0.849322	0.354388
193	0.307317	0.952846	0.845257	0.337925
194	0.331707	0.956748	0.852575	0.37317
195	0.315447	0.959024	0.851762	0.363469
196	0.326829	0.961626	0.855827	0.382529
197	0.317073	0.959675	0.852575	0.367003
198	0.325203	0.958049	0.852575	0.370426
199	0.334959	0.955772	0.852304	0.373634
200	0.334959	0.955772	0.852304	0.373634
201	0.339837	0.95935	0.856098	0.388614
202	0.323577	0.956098	0.850678	0.36327
203	0.330081	0.955772	0.851491	0.368822
204	0.326829	0.959024	0.853659	0.374858
205	0.328455	0.955772	0.85122	0.367211
206	0.326829	0.956423	0.851491	0.367421
207	0.323577	0.958049	0.852304	0.368805
208	0.325203	0.953821	0.849051	0.358589
209	0.330081	0.958699	0.85393	0.377139
210	0.333333	0.956423	0.852575	0.373857
211	0.339837	0.953496	0.85122	0.372141
212	0.317073	0.958049	0.85122	0.36229

213	0.328455	0.962602	0.856911	0.38708
214	0.297561	0.959675	0.849322	0.347113
215	0.315447	0.956748	0.849864	0.356947
216	0.343089	0.957724	0.855285	0.387099
217	0.318699	0.958049	0.851491	0.363924
218	0.318699	0.955122	0.849051	0.355666
219	0.320325	0.957073	0.850949	0.36277
220	0.320325	0.956423	0.850407	0.360932
221	0.341463	0.958699	0.855827	0.388316
222	0.321951	0.958049	0.852033	0.367182
223	0.341463	0.953171	0.85122	0.372845
224	0.313821	0.955772	0.84878	0.352563
225	0.313821	0.956423	0.849322	0.354388
226	0.317073	0.954797	0.848509	0.353132
227	0.313821	0.956098	0.849051	0.353474
228	0.331707	0.954797	0.850949	0.367719
229	0.331707	0.959675	0.855014	0.381581
230	0.331707	0.955772	0.851762	0.370429
231	0.331707	0.954472	0.850678	0.366822
232	0.330081	0.956748	0.852304	0.371563
233	0.321951	0.958049	0.852033	0.367182
234	0.305691	0.95122	0.843631	0.331919
235	0.325203	0.954472	0.849593	0.360372
236	0.317073	0.960976	0.853659	0.370842
237	0.310569	0.956748	0.849051	0.352012
238	0.338211	0.955122	0.852304	0.375016
239	0.339837	0.953171	0.850949	0.371257
240	0.346341	0.949919	0.849322	0.368923
241	0.325203	0.952846	0.848238	0.355937
242	0.330081	0.955772	0.851491	0.368822
243	0.330081	0.955447	0.85122	0.367915
244	0.326829	0.955772	0.850949	0.365597
245	0.320325	0.953171	0.847696	0.351946
246	0.331707	0.954146	0.850407	0.365929
247	0.330081	0.951545	0.847967	0.357288
248	0.308943	0.957724	0.849593	0.353133
249	0.344715	0.953171	0.851762	0.376013
250	0.325203	0.957398	0.852033	0.368566
251	0.323577	0.947317	0.84336	0.33982
252	0.320325	0.95187	0.846612	0.348444
253	0.323577	0.949919	0.845528	0.346534
254	0.315447	0.953496	0.847154	0.347928
255	0.313821	0.948618	0.842818	0.33337

256	0.35935	0.95252	0.853659	0.388361
257	0.334959	0.956748	0.853117	0.376375
258	0.323577	0.95252	0.847696	0.353439
259	0.321951	0.954146	0.84878	0.356234
260	0.341463	0.950894	0.849322	0.366745
261	0.308943	0.952195	0.844986	0.33783
262	0.331707	0.948293	0.845528	0.350379
263	0.328455	0.947967	0.844715	0.346328
264	0.334959	0.95187	0.849051	0.362968
265	0.346341	0.950244	0.849593	0.369776
266	0.323577	0.954472	0.849322	0.358752
267	0.334959	0.953171	0.850136	0.366473
268	0.341463	0.949268	0.847967	0.362478
269	0.330081	0.954146	0.850136	0.364321
270	0.318699	0.95122	0.845799	0.345082
271	0.330081	0.951545	0.847967	0.357288
272	0.343089	0.949919	0.84878	0.365761
273	0.325203	0.957073	0.851762	0.367642
274	0.349593	0.951545	0.85122	0.376369
275	0.315447	0.959675	0.852304	0.365366
276	0.357724	0.948618	0.850136	0.376509
277	0.333333	0.954472	0.850949	0.368426
278	0.331707	0.954797	0.850949	0.367719
279	0.330081	0.957724	0.853117	0.374335
280	0.330081	0.956748	0.852304	0.371563
281	0.346341	0.951545	0.850678	0.37322
282	0.35122	0.950569	0.850678	0.375352
283	0.323577	0.955772	0.850407	0.362359
284	0.328455	0.956098	0.851491	0.368122
285	0.338211	0.949919	0.847967	0.360996
286	0.318699	0.953496	0.847696	0.351199
287	0.326829	0.948943	0.845257	0.34723
288	0.323577	0.950569	0.84607	0.348242
289	0.343089	0.95122	0.849864	0.369193
290	0.341463	0.949919	0.848509	0.364176
291	0.349593	0.946016	0.846612	0.36205
292	0.347967	0.953171	0.852304	0.379168
293	0.333333	0.952195	0.849051	0.362239
294	0.343089	0.949268	0.848238	0.364063
295	0.336585	0.949919	0.847696	0.359402
296	0.346341	0.948943	0.848509	0.366379
297	0.354472	0.946992	0.848238	0.369219
298	0.323577	0.95187	0.847154	0.351694

299	0.334959	0.946016	0.844173	0.347792
300	0.338211	0.947317	0.845799	0.354274
301	0.339837	0.953821	0.851491	0.373028
302	0.339837	0.946992	0.845799	0.355037
303	0.341463	0.947317	0.846341	0.357452
304	0.341463	0.95122	0.849593	0.367607
305	0.35935	0.945691	0.847967	0.370607
306	0.325203	0.949268	0.845257	0.346457
307	0.334959	0.95122	0.848509	0.361235
308	0.344715	0.949268	0.848509	0.365645
309	0.344715	0.947642	0.847154	0.361449
310	0.344715	0.947317	0.846883	0.360619
311	0.35935	0.950244	0.851762	0.382304
312	0.35935	0.950569	0.852033	0.383161
313	0.349593	0.950244	0.850136	0.372926
314	0.356098	0.95122	0.852033	0.381768
315	0.326829	0.949593	0.845799	0.34892
316	0.354472	0.942114	0.844173	0.357109
317	0.336585	0.947967	0.84607	0.354344
318	0.339837	0.949919	0.848238	0.362588
319	0.347967	0.946341	0.846612	0.361297
320	0.354472	0.949268	0.850136	0.375076
321	0.339837	0.949593	0.847967	0.361737
322	0.328455	0.947317	0.844173	0.344666
323	0.315447	0.953496	0.847154	0.347928
324	0.336585	0.95187	0.849322	0.364566
325	0.331707	0.948618	0.845799	0.351218
326	0.325203	0.953496	0.84878	0.357702
327	0.334959	0.947317	0.845257	0.351084
328	0.336585	0.950894	0.848509	0.361971
329	0.35122	0.945366	0.846341	0.361987
330	0.338211	0.948943	0.847154	0.358454
331	0.352846	0.951545	0.851762	0.379507
332	0.331707	0.948293	0.845528	0.350379
333	0.326829	0.949268	0.845528	0.348073
334	0.343089	0.948293	0.847425	0.361537
335	0.347967	0.95252	0.851762	0.37741
336	0.336585	0.945366	0.843902	0.347758
337	0.334959	0.953496	0.850407	0.367356
338	0.354472	0.95187	0.852304	0.38194
339	0.35935	0.946341	0.848509	0.372245
340	0.326829	0.947317	0.843902	0.343054
341	0.338211	0.947967	0.846341	0.355938

342	0.321951	0.949919	0.845257	0.344911
343	0.330081	0.949593	0.846341	0.352143
344	0.333333	0.95122	0.848238	0.359634
345	0.347967	0.943415	0.844173	0.354013
346	0.334959	0.954472	0.85122	0.370027
347	0.333333	0.950569	0.847696	0.357913
348	0.347967	0.95187	0.85122	0.375664
349	0.341463	0.946341	0.845528	0.354978
350	0.343089	0.950569	0.849322	0.367471
351	0.339837	0.947642	0.846341	0.356695
352	0.364228	0.946992	0.849864	0.378545
353	0.336585	0.95252	0.849864	0.366312
354	0.336585	0.953496	0.850678	0.368954
355	0.334959	0.939837	0.839024	0.33273
356	0.35935	0.943089	0.845799	0.364157
357	0.344715	0.946016	0.845799	0.357323
358	0.347967	0.946667	0.846883	0.36212
359	0.357724	0.949593	0.850949	0.379044
360	0.347967	0.944715	0.845257	0.357224
361	0.334959	0.950894	0.848238	0.360373
362	0.349593	0.945041	0.845799	0.359605
363	0.321951	0.950894	0.84607	0.347477
364	0.333333	0.945041	0.843089	0.343752
365	0.334959	0.948618	0.846341	0.354421
366	0.372358	0.949919	0.853659	0.393797
367	0.321951	0.949593	0.844986	0.344061
368	0.356098	0.946341	0.847967	0.369131
369	0.35122	0.95122	0.85122	0.377074
370	0.344715	0.944065	0.844173	0.352461
371	0.333333	0.950894	0.847967	0.358772
372	0.362602	0.949593	0.851762	0.383703
373	0.318699	0.952846	0.847154	0.349435
374	0.346341	0.94439	0.844715	0.354842
375	0.334959	0.945041	0.84336	0.345351
376	0.321951	0.95252	0.847425	0.351816
377	0.339837	0.948293	0.846883	0.358365
378	0.323577	0.952846	0.847967	0.354317
379	0.354472	0.947642	0.84878	0.370878
380	0.369106	0.94374	0.847967	0.375027
381	0.326829	0.945041	0.842005	0.337327
382	0.35935	0.945691	0.847967	0.370607
383	0.352846	0.950894	0.85122	0.377779
384	0.336585	0.948293	0.846341	0.35518

385	0.357724	0.95122	0.852304	0.383326
386	0.354472	0.945691	0.847154	0.365932
387	0.336585	0.951545	0.849051	0.363698
388	0.333333	0.946341	0.844173	0.347011
389	0.339837	0.946992	0.845799	0.355037
390	0.323577	0.950569	0.84607	0.348242
391	0.330081	0.948618	0.845528	0.349611
392	0.344715	0.948618	0.847967	0.363958
393	0.328455	0.947642	0.844444	0.345496
394	0.347967	0.950244	0.849864	0.371352
395	0.341463	0.945366	0.844715	0.352527
396	0.362602	0.945691	0.848509	0.373709
397	0.344715	0.946992	0.846612	0.359791
398	0.347967	0.946341	0.846612	0.361297
399	0.331707	0.948943	0.84607	0.352059
400	0.344715	0.95187	0.850678	0.372509
401	0.341463	0.952195	0.850407	0.370212
402	0.330081	0.948293	0.845257	0.348773
403	0.343089	0.946667	0.84607	0.357384
404	0.352846	0.946667	0.847696	0.366829
405	0.343089	0.949593	0.848509	0.364911
406	0.343089	0.948618	0.847696	0.362376
407	0.338211	0.95122	0.849051	0.364427
408	0.317073	0.945041	0.840379	0.327596
409	0.339837	0.945366	0.844444	0.35094
410	0.343089	0.945366	0.844986	0.354111
411	0.346341	0.949593	0.849051	0.368072
412	0.36748	0.945366	0.849051	0.377527
413	0.315447	0.949268	0.843631	0.336691
414	0.356098	0.950244	0.85122	0.379189
415	0.346341	0.945366	0.845528	0.35727
416	0.347967	0.946667	0.846883	0.36212
417	0.357724	0.942764	0.845257	0.361808
418	0.325203	0.947642	0.843902	0.342268
419	0.313821	0.947967	0.842276	0.3317
420	0.328455	0.946667	0.843631	0.343016
421	0.339837	0.95122	0.849322	0.366019
422	0.333333	0.949919	0.847154	0.356204
423	0.344715	0.946992	0.846612	0.359791
424	0.331707	0.945041	0.842818	0.342151
425	0.356098	0.945041	0.846883	0.365866
426	0.336585	0.946341	0.844715	0.350207
427	0.35122	0.946016	0.846883	0.363619

428	0.330081	0.947967	0.844986	0.347938
429	0.336585	0.945691	0.844173	0.348571
430	0.320325	0.944065	0.840108	0.328441
431	0.35935	0.945041	0.847425	0.368979
432	0.349593	0.947642	0.847967	0.366177
433	0.336585	0.947967	0.84607	0.354344
434	0.349593	0.942439	0.843631	0.353202
435	0.331707	0.949593	0.846612	0.35375
436	0.330081	0.949593	0.846341	0.352143
437	0.343089	0.950569	0.849322	0.367471
438	0.347967	0.947642	0.847696	0.364604
439	0.35122	0.946341	0.847154	0.364439
440	0.336585	0.946341	0.844715	0.350207
441	0.334959	0.949919	0.847425	0.357804
442	0.339837	0.946341	0.845257	0.35339
443	0.35122	0.943089	0.844444	0.356356
444	0.315447	0.948943	0.84336	0.335849
445	0.346341	0.945691	0.845799	0.358085
446	0.343089	0.949268	0.848238	0.364063
447	0.334959	0.949268	0.846883	0.356107
448	0.331707	0.945366	0.843089	0.342961
449	0.339837	0.949593	0.847967	0.361737
450	0.331707	0.944715	0.842547	0.341343
451	0.321951	0.943089	0.839566	0.327677
452	0.339837	0.944065	0.84336	0.347711
453	0.334959	0.949593	0.847154	0.356954
454	0.325203	0.947317	0.843631	0.341439
455	0.344715	0.943089	0.84336	0.350065
456	0.321951	0.94439	0.84065	0.330865
457	0.349593	0.949593	0.849593	0.371221
458	0.346341	0.946016	0.84607	0.358902
459	0.325203	0.943089	0.840108	0.330911
460	0.343089	0.948943	0.847967	0.363218
461	0.333333	0.948618	0.84607	0.352821
462	0.343089	0.948618	0.847696	0.362376
463	0.334959	0.942439	0.841192	0.338959
464	0.320325	0.944715	0.84065	0.330046
465	0.344715	0.942764	0.843089	0.349271
466	0.331707	0.948618	0.845799	0.351218
467	0.330081	0.946341	0.843631	0.343803
468	0.328455	0.947317	0.844173	0.344666
469	0.338211	0.943089	0.842276	0.343728
470	0.338211	0.94374	0.842818	0.345321

471	0.339837	0.948618	0.847154	0.359203
472	0.341463	0.948293	0.847154	0.359952
473	0.339837	0.946341	0.845257	0.35339
474	0.341463	0.944065	0.843631	0.349297
475	0.35122	0.950569	0.850678	0.375352
476	0.328455	0.948618	0.845257	0.348002
477	0.346341	0.949268	0.84878	0.367224
478	0.338211	0.945691	0.844444	0.350164
479	0.330081	0.946016	0.84336	0.342985
480	0.35122	0.944715	0.845799	0.360365
481	0.328455	0.949593	0.84607	0.350533
482	0.356098	0.942439	0.844715	0.359458
483	0.346341	0.943415	0.843902	0.352438
484	0.35122	0.946992	0.847696	0.366088
485	0.341463	0.945691	0.844986	0.353341
486	0.330081	0.945366	0.842818	0.341356
487	0.339837	0.945366	0.844444	0.35094
488	0.341463	0.941789	0.841734	0.343744
489	0.328455	0.945691	0.842818	0.340561
490	0.357724	0.946667	0.848509	0.371513
491	0.317073	0.943089	0.838753	0.322801
492	0.338211	0.947317	0.845799	0.354274
493	0.347967	0.945041	0.845528	0.358033
494	0.346341	0.945041	0.845257	0.356458
495	0.299187	0.945041	0.837398	0.309449
496	0.341463	0.945691	0.844986	0.353341
497	0.344715	0.943415	0.843631	0.350861
498	0.352846	0.944065	0.845528	0.36032
499	0.346341	0.950894	0.850136	0.371492
500	0.341463	0.941789	0.841734	0.343744

(2) Dataset S_2

Number of features	SN	SP	ACC	MCC
4	0	1	0.833333	N/A
5	0.009756	0.998374	0.833604	0.055577
6	0.02439	0.997724	0.835501	0.107053
7	0.029268	0.995447	0.834417	0.099342
8	0.082927	0.993171	0.841463	0.205036
9	0.089431	0.992846	0.842276	0.214514
10	0.130081	0.988293	0.845257	0.252819
11	0.125203	0.992846	0.848238	0.272268
12	0.146341	0.989593	0.849051	0.283334

13	0.141463	0.989268	0.847967	0.274673
14	0.156098	0.990894	0.851762	0.303985
15	0.15935	0.988618	0.850407	0.295841
16	0.16748	0.987967	0.85122	0.303225
17	0.182114	0.986016	0.852033	0.312352
18	0.177236	0.986992	0.852033	0.310921
19	0.178862	0.986667	0.852033	0.31139
20	0.19187	0.987317	0.854743	0.330861
21	0.198374	0.985041	0.85393	0.327903
22	0.204878	0.982764	0.853117	0.325498
23	0.19187	0.98439	0.852304	0.316915
24	0.226016	0.980813	0.855014	0.34206
25	0.209756	0.98374	0.854743	0.335653
26	0.226016	0.980488	0.854743	0.340712
27	0.204878	0.980488	0.85122	0.315756
28	0.234146	0.981463	0.856911	0.354063
29	0.219512	0.982764	0.855556	0.342822
30	0.221138	0.978862	0.852575	0.328429
31	0.21626	0.980813	0.853388	0.330695
32	0.227642	0.978862	0.853659	0.335978
33	0.235772	0.977561	0.85393	0.340151
34	0.239024	0.977561	0.854472	0.343833
35	0.255285	0.978211	0.857724	0.364419
36	0.24065	0.975935	0.853388	0.339448
37	0.247154	0.977561	0.855827	0.352935
38	0.247154	0.977236	0.855556	0.35168
39	0.247154	0.975285	0.85393	0.344302
40	0.237398	0.977236	0.85393	0.340735
41	0.24878	0.973984	0.853117	0.341333
42	0.250407	0.974634	0.85393	0.345513
43	0.265041	0.972683	0.854743	0.354499
44	0.256911	0.974309	0.854743	0.351489
45	0.24878	0.974634	0.853659	0.343709
46	0.247154	0.973659	0.852575	0.338343
47	0.245528	0.977886	0.855827	0.352389
48	0.243902	0.976585	0.854472	0.345564
49	0.260163	0.97561	0.856369	0.359828
50	0.250407	0.974634	0.85393	0.345513
51	0.239024	0.971707	0.849593	0.322252
52	0.266667	0.974634	0.85664	0.363268
53	0.258537	0.974959	0.855556	0.35565
54	0.265041	0.969431	0.852033	0.343279
55	0.24878	0.974959	0.85393	0.344907

56	0.269919	0.971057	0.854201	0.354079
57	0.253659	0.968455	0.849322	0.327556
58	0.256911	0.970407	0.851491	0.337715
59	0.260163	0.970732	0.852304	0.342392
60	0.269919	0.973333	0.856098	0.362064
61	0.284553	0.96878	0.854743	0.361925
62	0.268293	0.971057	0.85393	0.35233
63	0.260163	0.970732	0.852304	0.342392
64	0.282927	0.967154	0.853117	0.354878
65	0.289431	0.967805	0.854743	0.363817
66	0.278049	0.969106	0.85393	0.35614
67	0.291057	0.971057	0.857724	0.376394
68	0.261789	0.96878	0.850949	0.337565
69	0.271545	0.967805	0.851762	0.344894
70	0.276423	0.96748	0.852304	0.349045
71	0.263415	0.969431	0.851762	0.341514
72	0.271545	0.968455	0.852304	0.347037
73	0.278049	0.964553	0.850136	0.341427
74	0.278049	0.964553	0.850136	0.341427
75	0.261789	0.969756	0.851762	0.340841
76	0.276423	0.965203	0.850407	0.34174
77	0.278049	0.968455	0.853388	0.353978
78	0.269919	0.965203	0.849322	0.334772
79	0.278049	0.968455	0.853388	0.353978
80	0.289431	0.964553	0.852033	0.353422
81	0.284553	0.96878	0.854743	0.361925
82	0.263415	0.967805	0.850407	0.336109
83	0.284553	0.962602	0.849593	0.342287
84	0.289431	0.961626	0.849593	0.344453
85	0.299187	0.965528	0.854472	0.366607
86	0.282927	0.969106	0.854743	0.361297
87	0.300813	0.966504	0.855556	0.371384
88	0.281301	0.965528	0.851491	0.347948
89	0.297561	0.964878	0.853659	0.362883
90	0.294309	0.965854	0.85393	0.362599
91	0.294309	0.965528	0.853659	0.361568
92	0.289431	0.965528	0.852846	0.356492
93	0.295935	0.965528	0.85393	0.363252
94	0.297561	0.963577	0.852575	0.358839
95	0.307317	0.964228	0.854743	0.370853
96	0.302439	0.962602	0.852575	0.360872
97	0.302439	0.962927	0.852846	0.361863
98	0.302439	0.963252	0.853117	0.362859

99	0.273171	0.961951	0.847154	0.328225
100	0.302439	0.962602	0.852575	0.360872
101	0.292683	0.961626	0.850136	0.347845
102	0.308943	0.962602	0.853659	0.367513
103	0.304065	0.962276	0.852575	0.361551
104	0.302439	0.961951	0.852033	0.358903
105	0.302439	0.963902	0.853659	0.364862
106	0.294309	0.963577	0.852033	0.355473
107	0.284553	0.962276	0.849322	0.341299
108	0.304065	0.956423	0.847696	0.344465
109	0.294309	0.965528	0.853659	0.361568
110	0.307317	0.957073	0.84878	0.349625
111	0.305691	0.963252	0.853659	0.366186
112	0.297561	0.963577	0.852575	0.358839
113	0.300813	0.964228	0.853659	0.364201
114	0.305691	0.964228	0.854472	0.369196
115	0.300813	0.961626	0.851491	0.356254
116	0.292683	0.962927	0.85122	0.351787
117	0.304065	0.961301	0.851762	0.358616
118	0.305691	0.96065	0.851491	0.358341
119	0.299187	0.961951	0.851491	0.355559
120	0.313821	0.957073	0.849864	0.356226
121	0.313821	0.956748	0.849593	0.355305
122	0.305691	0.959675	0.850678	0.355466
123	0.308943	0.960976	0.852304	0.362622
124	0.308943	0.961626	0.852846	0.364566
125	0.307317	0.96065	0.851762	0.36
126	0.295935	0.96065	0.849864	0.348308
127	0.302439	0.962602	0.852575	0.360872
128	0.325203	0.958699	0.853117	0.372299
129	0.307317	0.959675	0.850949	0.357125
130	0.305691	0.956098	0.847696	0.345214
131	0.305691	0.962276	0.852846	0.363213
132	0.307317	0.962276	0.853117	0.364872
133	0.325203	0.960976	0.855014	0.378973
134	0.315447	0.963902	0.855827	0.378076
135	0.325203	0.96065	0.854743	0.378008
136	0.318699	0.957398	0.850949	0.362064
137	0.305691	0.956748	0.848238	0.347045
138	0.318699	0.957724	0.85122	0.362992
139	0.304065	0.958374	0.849322	0.350023
140	0.313821	0.957724	0.850407	0.358079
141	0.313821	0.961301	0.853388	0.368535

142	0.339837	0.95935	0.856098	0.388614
143	0.312195	0.956423	0.849051	0.352743
144	0.320325	0.953821	0.848238	0.353717
145	0.328455	0.962276	0.85664	0.386096
146	0.310569	0.963577	0.854743	0.372146
147	0.331707	0.958699	0.854201	0.378745
148	0.334959	0.953821	0.850678	0.368244
149	0.310569	0.959024	0.850949	0.358535
150	0.325203	0.956098	0.850949	0.36489
151	0.321951	0.960325	0.85393	0.373805
152	0.321951	0.958699	0.852575	0.369055
153	0.331707	0.953496	0.849864	0.364151
154	0.305691	0.959024	0.850136	0.353568
155	0.325203	0.953496	0.84878	0.357702
156	0.330081	0.956748	0.852304	0.371563
157	0.313821	0.954146	0.847425	0.348063
158	0.315447	0.957073	0.850136	0.357867
159	0.315447	0.955772	0.849051	0.354205
160	0.323577	0.95187	0.847154	0.351694
161	0.323577	0.956423	0.850949	0.364184
162	0.343089	0.955772	0.853659	0.381591
163	0.331707	0.953496	0.849864	0.364151
164	0.328455	0.956748	0.852033	0.369952
165	0.320325	0.953496	0.847967	0.35283
166	0.330081	0.958049	0.853388	0.375266
167	0.308943	0.953171	0.845799	0.34046
168	0.318699	0.958049	0.851491	0.363924
169	0.318699	0.955122	0.849051	0.355666
170	0.320325	0.954797	0.849051	0.356397
171	0.315447	0.953171	0.846883	0.347044
172	0.336585	0.954797	0.851762	0.372522
173	0.323577	0.956098	0.850678	0.36327
174	0.331707	0.954797	0.850949	0.367719
175	0.320325	0.952846	0.847425	0.351066
176	0.346341	0.955772	0.854201	0.384751
177	0.317073	0.955122	0.84878	0.354032
178	0.318699	0.957073	0.850678	0.36114
179	0.321951	0.954472	0.849051	0.357128
180	0.35122	0.955772	0.855014	0.389469
181	0.323577	0.954146	0.849051	0.357858
182	0.330081	0.953821	0.849864	0.363431
183	0.323577	0.957398	0.851762	0.366946
184	0.312195	0.959675	0.851762	0.362081

185	0.349593	0.955122	0.854201	0.386091
186	0.343089	0.95122	0.849864	0.369193
187	0.334959	0.950569	0.847967	0.359514
188	0.349593	0.957398	0.856098	0.392478
189	0.326829	0.953171	0.84878	0.358435
190	0.318699	0.954472	0.848509	0.353869
191	0.330081	0.948293	0.845257	0.348773
192	0.341463	0.95122	0.849593	0.367607
193	0.339837	0.957398	0.854472	0.383
194	0.323577	0.953821	0.84878	0.356968
195	0.331707	0.948943	0.84607	0.352059
196	0.323577	0.950569	0.84607	0.348242
197	0.338211	0.951545	0.849322	0.365293
198	0.331707	0.953496	0.849864	0.364151
199	0.325203	0.949268	0.845257	0.346457
200	0.328455	0.958049	0.853117	0.373656
201	0.341463	0.95252	0.850678	0.371087
202	0.339837	0.952846	0.850678	0.370376
203	0.354472	0.951545	0.852033	0.381071
204	0.344715	0.956423	0.854472	0.384995
205	0.336585	0.955772	0.852575	0.375232
206	0.338211	0.949919	0.847967	0.360996
207	0.313821	0.946667	0.841192	0.328394
208	0.326829	0.949593	0.845799	0.34892
209	0.336585	0.948943	0.846883	0.35686
210	0.352846	0.946341	0.847425	0.366006
211	0.341463	0.946992	0.84607	0.356625
212	0.331707	0.95122	0.847967	0.35803
213	0.344715	0.954797	0.853117	0.380463
214	0.334959	0.949593	0.847154	0.356954
215	0.347967	0.947967	0.847967	0.365438
216	0.330081	0.947967	0.844986	0.347938
217	0.347967	0.950894	0.850407	0.373068
218	0.326829	0.946341	0.843089	0.340583
219	0.334959	0.953171	0.850136	0.366473
220	0.360976	0.949919	0.851762	0.383003
221	0.35122	0.955447	0.854743	0.388563
222	0.330081	0.949268	0.84607	0.351296
223	0.343089	0.949593	0.848509	0.364911
224	0.346341	0.954472	0.853117	0.381146
225	0.344715	0.951545	0.850407	0.37164
226	0.339837	0.953821	0.851491	0.373028
227	0.341463	0.953821	0.851762	0.374616

228	0.333333	0.945691	0.843631	0.345376
229	0.349593	0.94439	0.845257	0.357989
230	0.338211	0.948618	0.846883	0.357612
231	0.341463	0.950569	0.849051	0.365886
232	0.343089	0.950244	0.849051	0.366615
233	0.339837	0.952195	0.850136	0.368624
234	0.341463	0.950569	0.849051	0.365886
235	0.35122	0.95122	0.85122	0.377074
236	0.343089	0.946992	0.846341	0.358209
237	0.35122	0.946992	0.847696	0.366088
238	0.346341	0.95252	0.851491	0.375834
239	0.331707	0.952195	0.84878	0.360635
240	0.347967	0.954797	0.853659	0.383618
241	0.339837	0.953496	0.85122	0.372141
242	0.331707	0.952846	0.849322	0.362387
243	0.344715	0.947967	0.847425	0.362283
244	0.336585	0.953171	0.850407	0.36807
245	0.360976	0.949593	0.851491	0.382153
246	0.336585	0.947967	0.84607	0.354344
247	0.347967	0.947642	0.847696	0.364604
248	0.372358	0.950244	0.85393	0.39465
249	0.354472	0.953171	0.853388	0.385443
250	0.35122	0.95252	0.852304	0.380554
251	0.341463	0.955772	0.853388	0.380006
252	0.338211	0.949268	0.847425	0.359299
253	0.343089	0.949268	0.848238	0.364063
254	0.330081	0.950244	0.846883	0.353846
255	0.331707	0.950244	0.847154	0.355453
256	0.364228	0.946667	0.849593	0.377719
257	0.35935	0.948293	0.850136	0.377225
258	0.369106	0.949593	0.852846	0.389876
259	0.357724	0.951545	0.852575	0.384192
260	0.334959	0.947317	0.845257	0.351084
261	0.344715	0.948293	0.847696	0.363119
262	0.364228	0.948943	0.851491	0.383557
263	0.349593	0.947967	0.848238	0.367011
264	0.360976	0.947317	0.849593	0.376276
265	0.356098	0.942439	0.844715	0.359458
266	0.35122	0.947967	0.848509	0.368581
267	0.344715	0.950894	0.849864	0.369913
268	0.35935	0.949268	0.850949	0.379752
269	0.364228	0.950569	0.852846	0.387811
270	0.323577	0.955122	0.849864	0.360549

271	0.365854	0.952846	0.855014	0.395431
272	0.346341	0.949268	0.84878	0.367224
273	0.344715	0.949268	0.848509	0.365645
274	0.370732	0.946016	0.850136	0.382237
275	0.357724	0.946016	0.847967	0.369869
276	0.356098	0.947642	0.849051	0.37244
277	0.364228	0.947642	0.850407	0.380205
278	0.377236	0.947642	0.852575	0.392487
279	0.380488	0.944065	0.850136	0.386528
280	0.336585	0.94439	0.843089	0.345333
281	0.352846	0.948943	0.849593	0.372666
282	0.362602	0.95187	0.853659	0.389718
283	0.344715	0.95187	0.850678	0.372509
284	0.364228	0.942114	0.845799	0.366427
285	0.364228	0.950569	0.852846	0.387811
286	0.341463	0.945691	0.844986	0.353341
287	0.364228	0.947967	0.850678	0.381039
288	0.349593	0.943089	0.844173	0.354787
289	0.362602	0.947317	0.849864	0.377826
290	0.341463	0.951545	0.849864	0.368473
291	0.352846	0.951545	0.851762	0.379507
292	0.357724	0.94439	0.846612	0.365806
293	0.354472	0.946667	0.847967	0.368393
294	0.364228	0.945041	0.848238	0.373628
295	0.341463	0.947317	0.846341	0.357452
296	0.370732	0.943089	0.847696	0.374964
297	0.339837	0.953496	0.85122	0.372141
298	0.334959	0.953171	0.850136	0.366473
299	0.343089	0.950569	0.849322	0.367471
300	0.354472	0.948293	0.849322	0.372549
301	0.341463	0.949919	0.848509	0.364176
302	0.357724	0.945041	0.847154	0.367424
303	0.334959	0.94374	0.842276	0.342134
304	0.343089	0.947967	0.847154	0.360701
305	0.356098	0.945691	0.847425	0.367493
306	0.35122	0.947317	0.847967	0.366916
307	0.343089	0.943415	0.84336	0.349281
308	0.360976	0.947642	0.849864	0.377107
309	0.373984	0.946667	0.85122	0.386946
310	0.370732	0.945041	0.849322	0.37979
311	0.346341	0.949593	0.849051	0.368072
312	0.373984	0.947967	0.852304	0.390266
313	0.35935	0.947642	0.849593	0.375554

314	0.346341	0.947642	0.847425	0.363028
315	0.352846	0.947642	0.848509	0.369314
316	0.338211	0.947317	0.845799	0.354274
317	0.360976	0.946992	0.849322	0.375447
318	0.354472	0.950569	0.85122	0.378484
319	0.364228	0.950244	0.852575	0.386955
320	0.364228	0.94439	0.847696	0.37201
321	0.369106	0.946992	0.850678	0.38317
322	0.390244	0.940813	0.849051	0.387673
323	0.346341	0.950244	0.849593	0.369776
324	0.364228	0.946667	0.849593	0.377719
325	0.356098	0.946667	0.848238	0.369954
326	0.352846	0.942764	0.844444	0.357127
327	0.328455	0.948293	0.844986	0.347164
328	0.35935	0.947317	0.849322	0.374723
329	0.373984	0.947642	0.852033	0.389432
330	0.357724	0.950244	0.851491	0.380748
331	0.362602	0.939187	0.843089	0.357877
332	0.37561	0.948293	0.852846	0.392631
333	0.360976	0.941463	0.844715	0.361759
334	0.336585	0.947317	0.845528	0.352681
335	0.336585	0.947642	0.845799	0.353511
336	0.35122	0.940813	0.842547	0.350849
337	0.356098	0.941463	0.843902	0.357097
338	0.364228	0.94374	0.847154	0.370402
339	0.357724	0.949919	0.85122	0.379895
340	0.373984	0.945366	0.850136	0.383668
341	0.369106	0.941789	0.846341	0.370261
342	0.344715	0.944065	0.844173	0.352461
343	0.364228	0.948618	0.85122	0.382715
344	0.354472	0.945041	0.846612	0.364305
345	0.365854	0.946992	0.850136	0.380089
346	0.334959	0.947642	0.845528	0.351914
347	0.370732	0.944715	0.849051	0.378979
348	0.370732	0.947967	0.851762	0.387201
349	0.35935	0.945691	0.847967	0.370607
350	0.36748	0.943089	0.847154	0.37189
351	0.360976	0.945691	0.848238	0.37216
352	0.369106	0.948943	0.852304	0.388183
353	0.364228	0.943415	0.846883	0.369602
354	0.354472	0.946341	0.847696	0.36757
355	0.354472	0.941138	0.84336	0.354755
356	0.373984	0.943089	0.848238	0.378028

357	0.338211	0.943089	0.842276	0.343728
358	0.339837	0.94439	0.843631	0.348514
359	0.338211	0.94374	0.842818	0.345321
360	0.369106	0.944065	0.848238	0.37583
361	0.349593	0.946016	0.846612	0.36205
362	0.362602	0.943089	0.846341	0.367258
363	0.349593	0.943415	0.844444	0.355584
364	0.373984	0.940488	0.84607	0.371731
365	0.357724	0.945366	0.847425	0.368236
366	0.357724	0.941789	0.844444	0.359438
367	0.395122	0.942439	0.85122	0.396108
368	0.333333	0.946341	0.844173	0.347011
369	0.380488	0.942439	0.84878	0.382536
370	0.357724	0.948943	0.850407	0.377351
371	0.341463	0.942114	0.842005	0.34453
372	0.35935	0.940813	0.843902	0.358645
373	0.360976	0.946992	0.849322	0.375447
374	0.372358	0.943089	0.847967	0.376498
375	0.349593	0.942439	0.843631	0.353202
376	0.35935	0.939512	0.842818	0.355548
377	0.370732	0.943089	0.847696	0.374964
378	0.360976	0.943415	0.846341	0.366506
379	0.357724	0.945366	0.847425	0.368236
380	0.369106	0.94374	0.847967	0.375027
381	0.35935	0.945366	0.847696	0.369792
382	0.352846	0.940813	0.842818	0.352413
383	0.338211	0.950244	0.848238	0.36185
384	0.336585	0.945366	0.843902	0.347758
385	0.352846	0.950244	0.850678	0.376063
386	0.37561	0.943415	0.84878	0.380355
387	0.349593	0.947317	0.847696	0.365346
388	0.357724	0.940488	0.84336	0.356313
389	0.35935	0.942764	0.845528	0.363362
390	0.349593	0.946341	0.846883	0.36287
391	0.338211	0.946992	0.845528	0.353447
392	0.360976	0.942114	0.845257	0.363331
393	0.360976	0.941789	0.844986	0.362544
394	0.354472	0.947642	0.84878	0.370878
395	0.365854	0.94374	0.847425	0.371947
396	0.360976	0.94439	0.847154	0.368914
397	0.372358	0.941789	0.846883	0.37333
398	0.364228	0.944715	0.847967	0.372818
399	0.346341	0.945041	0.845257	0.356458

400	0.372358	0.942439	0.847425	0.374909
401	0.35935	0.940488	0.843631	0.357867
402	0.364228	0.944715	0.847967	0.372818
403	0.35935	0.943415	0.84607	0.364954
404	0.356098	0.945041	0.846883	0.365866
405	0.369106	0.945691	0.849593	0.379882
406	0.356098	0.945366	0.847154	0.366678
407	0.349593	0.944065	0.844986	0.357185
408	0.372358	0.942764	0.847696	0.375702
409	0.362602	0.942439	0.845799	0.36567
410	0.352846	0.947967	0.84878	0.370148
411	0.35935	0.942439	0.845257	0.36257
412	0.360976	0.94439	0.847154	0.368914
413	0.362602	0.944065	0.847154	0.369658
414	0.364228	0.943089	0.846612	0.368805
415	0.344715	0.939837	0.84065	0.342241
416	0.360976	0.937236	0.841192	0.351767
417	0.378862	0.943089	0.849051	0.382605
418	0.36748	0.948943	0.852033	0.386644
419	0.369106	0.94374	0.847967	0.375027
420	0.347967	0.949593	0.849322	0.369648
421	0.378862	0.946016	0.851491	0.389879
422	0.341463	0.949268	0.847967	0.362478
423	0.365854	0.945041	0.848509	0.375173
424	0.35122	0.944065	0.845257	0.358754
425	0.35122	0.942114	0.843631	0.353981
426	0.36748	0.938537	0.84336	0.360973
427	0.37561	0.941463	0.847154	0.375602
428	0.341463	0.946016	0.845257	0.354158
429	0.346341	0.946016	0.84607	0.358902
430	0.362602	0.94439	0.847425	0.370463
431	0.364228	0.942114	0.845799	0.366427
432	0.347967	0.945691	0.84607	0.35966
433	0.35935	0.939837	0.843089	0.356318
434	0.360976	0.947642	0.849864	0.377107
435	0.357724	0.945041	0.847154	0.367424
436	0.357724	0.94374	0.84607	0.364199
437	0.344715	0.944065	0.844173	0.352461
438	0.382114	0.940813	0.847696	0.380122
439	0.364228	0.948618	0.85122	0.382715
440	0.403252	0.944715	0.854472	0.40917
441	0.36748	0.943415	0.847425	0.372688
442	0.338211	0.946667	0.845257	0.352622

443	0.369106	0.94374	0.847967	0.375027
444	0.35122	0.948618	0.849051	0.370257
445	0.364228	0.94374	0.847154	0.370402
446	0.378862	0.943089	0.849051	0.382605
447	0.373984	0.940163	0.845799	0.370954
448	0.377236	0.942114	0.847967	0.378702
449	0.35935	0.941138	0.844173	0.359425
450	0.357724	0.944065	0.846341	0.365001
451	0.369106	0.945366	0.849322	0.379066
452	0.356098	0.944715	0.846612	0.365056
453	0.341463	0.946341	0.845528	0.354978
454	0.347967	0.941789	0.842818	0.350056
455	0.370732	0.940813	0.845799	0.369447
456	0.344715	0.940813	0.841463	0.344562
457	0.354472	0.939837	0.842276	0.35165
458	0.354472	0.946992	0.848238	0.369219
459	0.360976	0.938862	0.842547	0.355563
460	0.35122	0.946016	0.846883	0.363619
461	0.360976	0.941138	0.844444	0.360976
462	0.37561	0.937886	0.844173	0.367109
463	0.365854	0.943089	0.846883	0.370349
464	0.349593	0.945366	0.84607	0.360417
465	0.364228	0.942114	0.845799	0.366427
466	0.352846	0.945041	0.846341	0.362741
467	0.357724	0.940163	0.843089	0.355538
468	0.360976	0.942764	0.845799	0.364914
469	0.369106	0.945691	0.849593	0.379882
470	0.356098	0.944715	0.846612	0.365056
471	0.364228	0.937561	0.842005	0.355612
472	0.369106	0.943089	0.847425	0.373428
473	0.36748	0.942439	0.846612	0.370302
474	0.349593	0.941138	0.842547	0.350061
475	0.365854	0.945041	0.848509	0.375173
476	0.331707	0.941789	0.840108	0.334191
477	0.360976	0.945366	0.847967	0.371344
478	0.347967	0.939837	0.841192	0.345389
479	0.362602	0.941789	0.845257	0.364093
480	0.378862	0.938211	0.844986	0.370915
481	0.343089	0.944065	0.843902	0.35088
482	0.35935	0.945041	0.847425	0.368979
483	0.354472	0.94374	0.845528	0.361081
484	0.369106	0.942114	0.846612	0.371049
485	0.354472	0.946341	0.847696	0.36757

486	0.349593	0.944715	0.845528	0.358796
487	0.349593	0.943415	0.844444	0.355584
488	0.362602	0.939512	0.84336	0.358646
489	0.354472	0.946992	0.848238	0.369219
490	0.35935	0.939187	0.842547	0.354779
491	0.369106	0.94374	0.847967	0.375027
492	0.36748	0.944065	0.847967	0.374291
493	0.36748	0.940488	0.844986	0.365595
494	0.341463	0.945366	0.844715	0.352527
495	0.36748	0.94439	0.848238	0.375096
496	0.380488	0.939837	0.846612	0.376274
497	0.385366	0.943089	0.850136	0.388672
498	0.360976	0.943415	0.846341	0.366506
499	0.370732	0.940488	0.845528	0.368668
500	0.35935	0.938211	0.841734	0.352488

(3) Dataset S_3

Number of features	SN	SP	ACC	MCC
4	0.066667	0.987967	0.834417	0.141548
5	0.068293	0.990569	0.836856	0.159687
6	0.102439	0.989268	0.841463	0.214704
7	0.108943	0.988618	0.842005	0.221774
8	0.144715	0.985691	0.845528	0.26073
9	0.144715	0.984065	0.844173	0.25295
10	0.154472	0.98439	0.84607	0.268129
11	0.18374	0.980488	0.847696	0.289532
12	0.180488	0.982114	0.848509	0.292357
13	0.203252	0.981138	0.851491	0.316523
14	0.206504	0.981789	0.852575	0.323221
15	0.214634	0.979512	0.852033	0.323406
16	0.250407	0.977561	0.856369	0.356536
17	0.245528	0.97626	0.854472	0.346147
18	0.237398	0.978537	0.855014	0.34582
19	0.274797	0.974309	0.857724	0.370777
20	0.263415	0.972358	0.854201	0.35159
21	0.284553	0.972358	0.857724	0.374118
22	0.286179	0.974634	0.859892	0.383925
23	0.282927	0.973333	0.858266	0.375856
24	0.276423	0.972358	0.856369	0.365547
25	0.274797	0.972033	0.855827	0.36268
26	0.287805	0.974309	0.859892	0.384441
27	0.284553	0.971382	0.856911	0.370728

28	0.281301	0.970081	0.855285	0.362862
29	0.289431	0.971707	0.857995	0.376944
30	0.278049	0.972033	0.856369	0.366132
31	0.292683	0.970407	0.857453	0.375863
32	0.295935	0.96878	0.85664	0.373777
33	0.287805	0.968455	0.855014	0.364256
34	0.289431	0.96878	0.855556	0.36703
35	0.282927	0.969106	0.854743	0.361297
36	0.287805	0.96748	0.854201	0.361058
37	0.282927	0.970732	0.856098	0.366787
38	0.281301	0.969431	0.854743	0.36067
39	0.304065	0.969431	0.858537	0.384277
40	0.304065	0.964228	0.854201	0.367535
41	0.308943	0.965528	0.856098	0.376579
42	0.323577	0.966829	0.859621	0.39541
43	0.300813	0.966829	0.855827	0.372429
44	0.308943	0.965528	0.856098	0.376579
45	0.312195	0.969431	0.859892	0.392514
46	0.312195	0.963577	0.855014	0.373792
47	0.317073	0.965528	0.857453	0.384783
48	0.325203	0.959675	0.85393	0.375137
49	0.302439	0.967805	0.856911	0.377255
50	0.315447	0.965203	0.856911	0.382126
51	0.320325	0.964228	0.856911	0.383975
52	0.312195	0.964553	0.855827	0.376813
53	0.330081	0.968455	0.86206	0.407096
54	0.330081	0.966179	0.860163	0.399773
55	0.323577	0.965203	0.858266	0.390258
56	0.321951	0.965203	0.857995	0.388639
57	0.326829	0.965854	0.85935	0.395532
58	0.318699	0.962602	0.855285	0.377361
59	0.325203	0.965203	0.858537	0.391874
60	0.312195	0.965528	0.85664	0.379872
61	0.328455	0.964878	0.858808	0.394078
62	0.315447	0.963902	0.855827	0.378076
63	0.299187	0.969106	0.857453	0.378204
64	0.326829	0.962276	0.856369	0.384485
65	0.325203	0.967154	0.860163	0.398067
66	0.310569	0.96748	0.857995	0.384467
67	0.330081	0.963902	0.858266	0.392661
68	0.349593	0.962927	0.860705	0.408701
69	0.323577	0.964553	0.857724	0.388228
70	0.344715	0.963902	0.860705	0.406966

71	0.352846	0.961301	0.859892	0.40695
72	0.321951	0.962276	0.855556	0.37963
73	0.336585	0.95935	0.855556	0.385431
74	0.325203	0.964878	0.858266	0.390857
75	0.331707	0.964553	0.859079	0.396274
76	0.346341	0.965203	0.86206	0.412563
77	0.318699	0.964553	0.856911	0.383357
78	0.331707	0.963252	0.857995	0.392269
79	0.338211	0.95935	0.855827	0.387024
80	0.343089	0.96065	0.857724	0.395597
81	0.344715	0.961301	0.858537	0.399104
82	0.347967	0.961951	0.859621	0.404193
83	0.35935	0.963577	0.862873	0.420017
84	0.331707	0.961951	0.856911	0.388329
85	0.336585	0.961951	0.857724	0.393123
86	0.334959	0.958699	0.854743	0.381949
87	0.336585	0.962927	0.858537	0.39607
88	0.344715	0.958049	0.855827	0.38961
89	0.338211	0.960976	0.857182	0.391802
90	0.341463	0.96	0.856911	0.3921
91	0.334959	0.957073	0.853388	0.377295
92	0.341463	0.959675	0.85664	0.391149
93	0.35935	0.956423	0.856911	0.399091
94	0.341463	0.96065	0.857453	0.394014
95	0.331707	0.958699	0.854201	0.378745
96	0.334959	0.959024	0.855014	0.38289
97	0.334959	0.960325	0.856098	0.386692
98	0.357724	0.95935	0.859079	0.405893
99	0.349593	0.959024	0.857453	0.397143
100	0.347967	0.96	0.857995	0.398413
101	0.364228	0.957398	0.858537	0.406489
102	0.36748	0.956098	0.857995	0.405908
103	0.352846	0.957724	0.856911	0.396539
104	0.343089	0.963902	0.860434	0.40539
105	0.370732	0.95935	0.861247	0.41823
106	0.354472	0.961301	0.860163	0.40851
107	0.369106	0.957724	0.859621	0.41203
108	0.369106	0.95935	0.860976	0.416698
109	0.347967	0.958374	0.85664	0.393695
110	0.352846	0.958049	0.857182	0.397468
111	0.349593	0.958699	0.857182	0.396203
112	0.360976	0.960325	0.860434	0.411841
113	0.343089	0.960325	0.857453	0.394638

114	0.362602	0.95935	0.859892	0.410542
115	0.377236	0.959024	0.86206	0.423391
116	0.35122	0.958699	0.857453	0.397771
117	0.352846	0.959675	0.858537	0.402164
118	0.354472	0.957724	0.857182	0.398101
119	0.370732	0.957073	0.85935	0.41172
120	0.352846	0.956423	0.855827	0.392856
121	0.372358	0.958699	0.860976	0.417883
122	0.356098	0.954472	0.854743	0.390556
123	0.369106	0.96	0.861518	0.418589
124	0.364228	0.95935	0.860163	0.412085
125	0.39187	0.956748	0.862602	0.430494
126	0.354472	0.958699	0.857995	0.400898
127	0.35935	0.956098	0.85664	0.39818
128	0.38374	0.958699	0.862873	0.428513
129	0.385366	0.96	0.864228	0.433777
130	0.360976	0.957073	0.857724	0.402475
131	0.370732	0.954146	0.856911	0.403583
132	0.378862	0.955122	0.859079	0.413899
133	0.377236	0.955122	0.858808	0.412378
134	0.373984	0.956098	0.859079	0.412039
135	0.365854	0.956748	0.858266	0.406193
136	0.388618	0.95122	0.857453	0.412414
137	0.377236	0.958699	0.861789	0.422455
138	0.369106	0.953496	0.856098	0.400273
139	0.382114	0.952846	0.857724	0.410721
140	0.356098	0.956423	0.856369	0.39598
141	0.364228	0.954146	0.855827	0.397425
142	0.372358	0.954146	0.857182	0.405115
143	0.38374	0.955772	0.860434	0.420248
144	0.370732	0.955772	0.858266	0.408072
145	0.385366	0.956098	0.860976	0.422663
146	0.373984	0.950244	0.854201	0.396181
147	0.35122	0.955447	0.854743	0.388563
148	0.362602	0.958374	0.859079	0.407728
149	0.382114	0.955122	0.859621	0.416933
150	0.380488	0.95122	0.856098	0.404854
151	0.393496	0.955772	0.86206	0.429268
152	0.373984	0.954146	0.857453	0.406645
153	0.378862	0.952846	0.857182	0.407684
154	0.388618	0.954146	0.859892	0.420291
155	0.37561	0.955122	0.858537	0.410855
156	0.386992	0.953496	0.859079	0.417014

157	0.386992	0.953171	0.858808	0.416133
158	0.380488	0.95187	0.85664	0.406585
159	0.37561	0.954797	0.858266	0.409957
160	0.388618	0.953821	0.859621	0.419404
161	0.388618	0.954472	0.860163	0.421181
162	0.388618	0.95122	0.857453	0.412414
163	0.398374	0.953496	0.860976	0.427502
164	0.39187	0.950244	0.857182	0.412847
165	0.369106	0.95187	0.854743	0.39589
166	0.364228	0.950894	0.853117	0.38867
167	0.378862	0.954472	0.858537	0.412109
168	0.369106	0.953821	0.856369	0.401159
169	0.373984	0.95122	0.855014	0.398758
170	0.38374	0.954146	0.859079	0.415766
171	0.38374	0.949268	0.855014	0.40276
172	0.393496	0.95187	0.858808	0.418649
173	0.388618	0.949268	0.855827	0.407291
174	0.398374	0.95252	0.860163	0.424871
175	0.396748	0.954797	0.861789	0.42956
176	0.382114	0.95187	0.856911	0.408102
177	0.413008	0.946992	0.857995	0.423762
178	0.373984	0.947317	0.851762	0.388601
179	0.388618	0.956423	0.861789	0.426583
180	0.4	0.952195	0.860163	0.425488
181	0.40813	0.955447	0.864228	0.441725
182	0.386992	0.953496	0.859079	0.417014
183	0.370732	0.949919	0.853388	0.392263
184	0.386992	0.951545	0.857453	0.411771
185	0.390244	0.948618	0.855556	0.40711
186	0.369106	0.951545	0.854472	0.395022
187	0.382114	0.952195	0.857182	0.408972
188	0.39187	0.946341	0.85393	0.402795
189	0.393496	0.955122	0.861518	0.427468
190	0.385366	0.947317	0.853659	0.399246
191	0.386992	0.954146	0.859621	0.418785
192	0.378862	0.949268	0.854201	0.398207
193	0.401626	0.947967	0.856911	0.415902
194	0.393496	0.949593	0.856911	0.412645
195	0.373984	0.950894	0.854743	0.397896
196	0.390244	0.949919	0.85664	0.410492
197	0.395122	0.948618	0.856369	0.41161
198	0.4	0.950569	0.858808	0.421175
199	0.413008	0.950244	0.860705	0.432151

200	0.372358	0.949593	0.853388	0.392946
201	0.396748	0.950569	0.858266	0.418193
202	0.398374	0.945366	0.854201	0.406328
203	0.388618	0.946667	0.853659	0.40061
204	0.401626	0.947967	0.856911	0.415902
205	0.395122	0.947642	0.855556	0.409102
206	0.390244	0.945041	0.852575	0.398024
207	0.398374	0.947642	0.856098	0.41209
208	0.390244	0.95187	0.858266	0.415649
209	0.385366	0.952195	0.857724	0.411998
210	0.39187	0.950569	0.857453	0.413702
211	0.373984	0.953821	0.857182	0.405757
212	0.38374	0.943415	0.850136	0.387957
213	0.403252	0.949268	0.858266	0.420746
214	0.39187	0.949268	0.856369	0.410298
215	0.370732	0.947317	0.85122	0.385536
216	0.411382	0.947967	0.858537	0.42478
217	0.395122	0.944715	0.853117	0.401715
218	0.426016	0.944065	0.857724	0.428125
219	0.395122	0.946016	0.854201	0.404973
220	0.380488	0.945041	0.850949	0.388953
221	0.377236	0.946667	0.851762	0.39
222	0.382114	0.945366	0.851491	0.391284
223	0.409756	0.943415	0.854472	0.411875
224	0.403252	0.950569	0.85935	0.424147
225	0.385366	0.950569	0.856369	0.407678
226	0.386992	0.946341	0.853117	0.398278
227	0.398374	0.94439	0.853388	0.403896
228	0.39187	0.941463	0.849864	0.390742
229	0.395122	0.947317	0.855285	0.408271
230	0.396748	0.949919	0.857724	0.416486
231	0.36748	0.948293	0.851491	0.384962
232	0.386992	0.95252	0.858266	0.41438
233	0.36748	0.946992	0.850407	0.381631
234	0.395122	0.948293	0.856098	0.410771
235	0.390244	0.949919	0.85664	0.410492
236	0.398374	0.946341	0.855014	0.408782
237	0.40813	0.945691	0.856098	0.416053
238	0.386992	0.938862	0.846883	0.380017
239	0.41626	0.944065	0.856098	0.419364
240	0.38374	0.94439	0.850949	0.390367
241	0.398374	0.947967	0.856369	0.412923
242	0.386992	0.947642	0.854201	0.401587

243	0.404878	0.946016	0.855827	0.413911
244	0.386992	0.947642	0.854201	0.401587
245	0.382114	0.945366	0.851491	0.391284
246	0.401626	0.946341	0.855556	0.411761
247	0.39187	0.94374	0.851762	0.396299
248	0.395122	0.946992	0.855014	0.407442
249	0.404878	0.945041	0.855014	0.411465
250	0.393496	0.94374	0.852033	0.397799
251	0.390244	0.948293	0.855285	0.406271
252	0.430894	0.941463	0.856369	0.426121
253	0.386992	0.946667	0.853388	0.399102
254	0.404878	0.947642	0.857182	0.418038
255	0.386992	0.945691	0.852575	0.396639
256	0.39187	0.947317	0.854743	0.405272
257	0.398374	0.944715	0.853659	0.404704
258	0.393496	0.943415	0.851762	0.396998
259	0.413008	0.942764	0.854472	0.413228
260	0.382114	0.942764	0.849322	0.384848
261	0.398374	0.946667	0.855285	0.409605
262	0.385366	0.944065	0.850949	0.391075
263	0.403252	0.937236	0.848238	0.391172
264	0.401626	0.946992	0.856098	0.41341
265	0.396748	0.943415	0.852304	0.399993
266	0.406504	0.94374	0.854201	0.409719
267	0.390244	0.940813	0.849051	0.387673
268	0.401626	0.946667	0.855827	0.412584
269	0.377236	0.938862	0.845257	0.370922
270	0.37561	0.945041	0.850136	0.384383
271	0.403252	0.94439	0.854201	0.408362
272	0.388618	0.94374	0.85122	0.39329
273	0.38374	0.948293	0.854201	0.400234
274	0.382114	0.946992	0.852846	0.395389
275	0.409756	0.944715	0.855556	0.415092
276	0.406504	0.949268	0.858808	0.423709
277	0.401626	0.943089	0.852846	0.403667
278	0.4	0.944715	0.85393	0.406195
279	0.413008	0.940813	0.852846	0.4085
280	0.414634	0.944065	0.855827	0.417896
281	0.39187	0.941463	0.849864	0.390742
282	0.396748	0.944715	0.853388	0.403211
283	0.373984	0.946016	0.850678	0.385302
284	0.403252	0.943415	0.853388	0.405952
285	0.419512	0.945691	0.857995	0.426343

286	0.38374	0.938862	0.846341	0.376995
287	0.422764	0.944065	0.857182	0.425213
288	0.388618	0.942764	0.850407	0.390894
289	0.414634	0.941789	0.85393	0.412324
290	0.426016	0.940163	0.854472	0.418645
291	0.413008	0.945691	0.856911	0.420477
292	0.395122	0.94439	0.852846	0.400907
293	0.411382	0.938537	0.850678	0.401613
294	0.37561	0.946992	0.851762	0.3893
295	0.413008	0.941138	0.853117	0.409282
296	0.365854	0.946016	0.849322	0.377619
297	0.398374	0.938862	0.84878	0.390518
298	0.39187	0.941789	0.850136	0.391529
299	0.395122	0.944065	0.852575	0.400101
300	0.406504	0.939837	0.850949	0.400262
301	0.4	0.940813	0.850678	0.396655
302	0.406504	0.944065	0.854472	0.410522
303	0.386992	0.94439	0.851491	0.393391
304	0.42439	0.94374	0.857182	0.425867
305	0.406504	0.944065	0.854472	0.410522
306	0.406504	0.945691	0.855827	0.414574
307	0.409756	0.939187	0.850949	0.401674
308	0.4	0.943089	0.852575	0.402179
309	0.378862	0.941789	0.847967	0.379436
310	0.414634	0.940813	0.853117	0.40997
311	0.411382	0.941138	0.852846	0.407809
312	0.396748	0.946992	0.855285	0.408938
313	0.4	0.940813	0.850678	0.396655
314	0.403252	0.949268	0.858266	0.420746
315	0.4	0.943415	0.852846	0.402977
316	0.390244	0.939187	0.847696	0.383797
317	0.411382	0.937236	0.849593	0.398568
318	0.42439	0.944715	0.857995	0.428282
319	0.403252	0.94374	0.853659	0.406753
320	0.393496	0.941463	0.850136	0.392243
321	0.403252	0.944715	0.854472	0.40917
322	0.390244	0.942114	0.850136	0.390815
323	0.393496	0.944065	0.852304	0.398603
324	0.406504	0.942439	0.853117	0.40653
325	0.411382	0.940163	0.852033	0.405469
326	0.386992	0.937236	0.845528	0.376209
327	0.413008	0.939187	0.851491	0.404622
328	0.414634	0.941138	0.853388	0.410753

329	0.406504	0.942764	0.853388	0.407323
330	0.437398	0.940163	0.856369	0.428772
331	0.388618	0.940813	0.84878	0.386168
332	0.406504	0.938862	0.850136	0.397949
333	0.409756	0.938537	0.850407	0.400138
334	0.39187	0.942764	0.850949	0.393903
335	0.421138	0.938211	0.852033	0.409652
336	0.4	0.939512	0.849593	0.393548
337	0.421138	0.94374	0.85664	0.422951
338	0.404878	0.939837	0.850678	0.39878
339	0.393496	0.937886	0.847154	0.383739
340	0.419512	0.944065	0.85664	0.422293
341	0.430894	0.936585	0.852304	0.414585
342	0.395122	0.939187	0.848509	0.388298
343	0.398374	0.941463	0.850949	0.39673
344	0.398374	0.938537	0.848509	0.389751
345	0.40813	0.938211	0.849864	0.397897
346	0.414634	0.942114	0.854201	0.413113
347	0.413008	0.940488	0.852575	0.40772
348	0.401626	0.942764	0.852575	0.402871
349	0.386992	0.944065	0.85122	0.392585
350	0.40813	0.944065	0.854743	0.412001
351	0.393496	0.937561	0.846883	0.38298
352	0.39187	0.941138	0.849593	0.389958
353	0.413008	0.93561	0.848509	0.39628
354	0.39187	0.940488	0.849051	0.388396
355	0.401626	0.940163	0.850407	0.396586
356	0.40813	0.939512	0.850949	0.400968
357	0.401626	0.94439	0.85393	0.406876
358	0.40813	0.940813	0.852033	0.404075
359	0.426016	0.939512	0.85393	0.417096
360	0.40813	0.945366	0.855827	0.415238
361	0.426016	0.94439	0.857995	0.428929
362	0.395122	0.942114	0.850949	0.395317
363	0.395122	0.945691	0.85393	0.404155
364	0.386992	0.941463	0.849051	0.386226
365	0.406504	0.940813	0.851762	0.402595
366	0.406504	0.945366	0.855556	0.413759
367	0.396748	0.944065	0.852846	0.401597
368	0.413008	0.941138	0.853117	0.409282
369	0.4	0.939512	0.849593	0.393548
370	0.404878	0.941789	0.852304	0.403467
371	0.429268	0.934959	0.850678	0.409394

372	0.4	0.942764	0.852304	0.401382
373	0.42439	0.940813	0.854743	0.418747
374	0.388618	0.937561	0.84607	0.378473
375	0.395122	0.941789	0.850678	0.394528
376	0.385366	0.937561	0.845528	0.375457
377	0.39187	0.941463	0.849864	0.390742
378	0.385366	0.941463	0.84878	0.384716
379	0.409756	0.942439	0.853659	0.409486
380	0.396748	0.944065	0.852846	0.401597
381	0.419512	0.940163	0.853388	0.412811
382	0.404878	0.944065	0.854201	0.40904
383	0.42439	0.937886	0.852304	0.411806
384	0.4	0.940813	0.850678	0.396655
385	0.401626	0.940163	0.850407	0.396586
386	0.422764	0.938537	0.852575	0.411876
387	0.435772	0.939512	0.855556	0.425783
388	0.406504	0.940813	0.851762	0.402595
389	0.411382	0.941138	0.852846	0.407809
390	0.421138	0.936585	0.850678	0.405859
391	0.409756	0.937561	0.849593	0.397851
392	0.380488	0.94374	0.849864	0.385725
393	0.37561	0.940163	0.84607	0.372481
394	0.41626	0.939837	0.852575	0.409105
395	0.403252	0.941463	0.851762	0.401196
396	0.414634	0.94439	0.856098	0.418701
397	0.396748	0.944715	0.853388	0.403211
398	0.398374	0.944065	0.853117	0.40309
399	0.403252	0.942764	0.852846	0.404358
400	0.411382	0.943089	0.854472	0.412551
401	0.4	0.940163	0.850136	0.395097
402	0.403252	0.940163	0.850678	0.398072
403	0.390244	0.946992	0.854201	0.402941
404	0.373984	0.938211	0.844173	0.366343
405	0.393496	0.943415	0.851762	0.396998
406	0.378862	0.94374	0.849593	0.384204
407	0.40813	0.937236	0.849051	0.395616
408	0.421138	0.940163	0.853659	0.414272
409	0.390244	0.939837	0.848238	0.385341
410	0.396748	0.940813	0.850136	0.39367
411	0.409756	0.940488	0.852033	0.404772
412	0.396748	0.941138	0.850407	0.394452
413	0.41626	0.942764	0.855014	0.416166
414	0.4	0.941138	0.850949	0.397437

415	0.404878	0.942439	0.852846	0.405048
416	0.403252	0.938862	0.849593	0.394983
417	0.421138	0.935935	0.850136	0.404357
418	0.4	0.941138	0.850949	0.397437
419	0.417886	0.941463	0.854201	0.414471
420	0.404878	0.941789	0.852304	0.403467
421	0.409756	0.93626	0.848509	0.394831
422	0.4	0.940488	0.850407	0.395875
423	0.404878	0.943415	0.853659	0.407436
424	0.393496	0.940813	0.849593	0.390676
425	0.409756	0.944065	0.855014	0.413478
426	0.403252	0.941138	0.851491	0.400412
427	0.427642	0.93561	0.850949	0.409434
428	0.390244	0.939187	0.847696	0.383797
429	0.390244	0.941138	0.849322	0.388455
430	0.403252	0.942114	0.852304	0.402772
431	0.382114	0.939187	0.846341	0.376248
432	0.385366	0.940813	0.848238	0.38315
433	0.398374	0.937561	0.847696	0.387465
434	0.406504	0.942114	0.852846	0.405738
435	0.403252	0.944715	0.854472	0.40917
436	0.409756	0.937236	0.849322	0.397093
437	0.4	0.940163	0.850136	0.395097
438	0.386992	0.943415	0.850678	0.390981
439	0.41626	0.942764	0.855014	0.416166
440	0.406504	0.942764	0.853388	0.407323
441	0.41626	0.939187	0.852033	0.407561
442	0.409756	0.945041	0.855827	0.415902
443	0.401626	0.940813	0.850949	0.398143
444	0.403252	0.939187	0.849864	0.395752
445	0.396748	0.939187	0.84878	0.389793
446	0.401626	0.939512	0.849864	0.395037
447	0.413008	0.940813	0.852846	0.4085
448	0.404878	0.940163	0.850949	0.399556
449	0.411382	0.940813	0.852575	0.407027
450	0.39187	0.940163	0.84878	0.387619
451	0.398374	0.937561	0.847696	0.387465
452	0.390244	0.939187	0.847696	0.383797
453	0.380488	0.940488	0.847154	0.377825
454	0.43252	0.940488	0.855827	0.425222
455	0.404878	0.945691	0.855556	0.413093
456	0.393496	0.941138	0.849864	0.391458
457	0.398374	0.938211	0.848238	0.388987

458	0.404878	0.937236	0.848509	0.392655
459	0.395122	0.942114	0.850949	0.395317
460	0.403252	0.939837	0.850407	0.397296
461	0.409756	0.946992	0.857453	0.420816
462	0.378862	0.941138	0.847425	0.377866
463	0.404878	0.94374	0.85393	0.408237
464	0.411382	0.942764	0.854201	0.411755
465	0.406504	0.941138	0.852033	0.403378
466	0.413008	0.935935	0.84878	0.397028
467	0.409756	0.940488	0.852033	0.404772
468	0.40813	0.934634	0.846883	0.389626
469	0.39187	0.940488	0.849051	0.388396
470	0.413008	0.937561	0.850136	0.400798
471	0.41626	0.944715	0.85664	0.420977
472	0.419512	0.942439	0.855285	0.418302
473	0.369106	0.937886	0.843089	0.360988
474	0.39187	0.941789	0.850136	0.391529
475	0.39187	0.937886	0.846883	0.382239
476	0.406504	0.938862	0.850136	0.397949
477	0.404878	0.937561	0.84878	0.393413
478	0.414634	0.940813	0.853117	0.40997
479	0.406504	0.942439	0.853117	0.40653
480	0.395122	0.93561	0.845528	0.379963
481	0.364228	0.939512	0.843631	0.360191
482	0.395122	0.941138	0.850136	0.392957
483	0.38374	0.945691	0.852033	0.393615
484	0.403252	0.945041	0.854743	0.409981
485	0.411382	0.94439	0.855556	0.415759
486	0.411382	0.940488	0.852304	0.406247
487	0.422764	0.939512	0.853388	0.414183
488	0.414634	0.938537	0.85122	0.404556
489	0.39187	0.941138	0.849593	0.389958
490	0.395122	0.941138	0.850136	0.392957
491	0.403252	0.94439	0.854201	0.408362
492	0.42439	0.940813	0.854743	0.418747
493	0.406504	0.940488	0.851491	0.401815
494	0.41626	0.934309	0.847967	0.396246
495	0.390244	0.938537	0.847154	0.382263
496	0.417886	0.934309	0.848238	0.397712
497	0.421138	0.93626	0.850407	0.405107
498	0.409756	0.938862	0.850678	0.400905
499	0.40813	0.935285	0.847425	0.391111
500	0.398374	0.941463	0.850949	0.39673

(4) Dataset S_4

Number of features	SN	SP	ACC	MCC
4	0.00813	0.999675	0.834417	0.072192
5	0.030894	0.997398	0.836314	0.123718
6	0.102439	0.989919	0.842005	0.218454
7	0.130081	0.988293	0.845257	0.252819
8	0.141463	0.990244	0.84878	0.280131
9	0.164228	0.986992	0.849864	0.293972
10	0.165854	0.986341	0.849593	0.292894
11	0.180488	0.987967	0.853388	0.319956
12	0.178862	0.988618	0.853659	0.32124
13	0.21626	0.986016	0.857724	0.353711
14	0.20813	0.982764	0.853659	0.329399
15	0.206504	0.98374	0.854201	0.331776
16	0.227642	0.982764	0.856911	0.352203
17	0.226016	0.982439	0.856369	0.348937
18	0.252033	0.981463	0.859892	0.373944
19	0.250407	0.980488	0.858808	0.368152
20	0.242276	0.981789	0.858537	0.364549
21	0.24065	0.982439	0.858808	0.36549
22	0.253659	0.977236	0.85664	0.358864
23	0.260163	0.977561	0.857995	0.367209
24	0.261789	0.979187	0.859621	0.37531
25	0.274797	0.979187	0.861789	0.389169
26	0.289431	0.976585	0.86206	0.394475
27	0.291057	0.975935	0.861789	0.393739
28	0.273171	0.979187	0.861518	0.387454
29	0.274797	0.977561	0.860434	0.382871
30	0.295935	0.974309	0.861247	0.392837
31	0.282927	0.975285	0.859892	0.382901
32	0.268293	0.977236	0.859079	0.374718
33	0.289431	0.97626	0.861789	0.393264
34	0.294309	0.974309	0.860976	0.391166
35	0.295935	0.976585	0.863144	0.401158
36	0.294309	0.975285	0.861789	0.394699
37	0.302439	0.974309	0.862331	0.399477
38	0.295935	0.973984	0.860976	0.391672
39	0.300813	0.97561	0.863144	0.402532
40	0.299187	0.970407	0.858537	0.382567
41	0.291057	0.97626	0.86206	0.394942
42	0.295935	0.974309	0.861247	0.392837
43	0.302439	0.969431	0.858266	0.382618

44	0.308943	0.972683	0.86206	0.400312
45	0.284553	0.973984	0.859079	0.379882
46	0.299187	0.972358	0.860163	0.389268
47	0.305691	0.971057	0.860163	0.391414
48	0.300813	0.972358	0.860434	0.39093
49	0.300813	0.973984	0.861789	0.39666
50	0.302439	0.971707	0.860163	0.390336
51	0.299187	0.971707	0.859621	0.387013
52	0.312195	0.970407	0.860705	0.395781
53	0.315447	0.969756	0.860705	0.396865
54	0.308943	0.96878	0.858808	0.387075
55	0.300813	0.970081	0.858537	0.383135
56	0.305691	0.972033	0.860976	0.394765
57	0.286179	0.973333	0.858808	0.379258
58	0.305691	0.968455	0.857995	0.382703
59	0.318699	0.971707	0.862873	0.406712
60	0.321951	0.96748	0.859892	0.395885
61	0.304065	0.96748	0.856911	0.377859
62	0.312195	0.96748	0.858266	0.386109
63	0.318699	0.96878	0.860434	0.396885
64	0.320325	0.96878	0.860705	0.398506
65	0.321951	0.967805	0.860163	0.396938
66	0.325203	0.966179	0.85935	0.394951
67	0.300813	0.966829	0.855827	0.372429
68	0.330081	0.969431	0.862873	0.410303
69	0.331707	0.96748	0.861518	0.40553
70	0.321951	0.967154	0.859621	0.394836
71	0.326829	0.96813	0.861247	0.402831
72	0.318699	0.965854	0.857995	0.38744
73	0.326829	0.966179	0.859621	0.396561
74	0.330081	0.96748	0.861247	0.403931
75	0.320325	0.967154	0.85935	0.393216
76	0.323577	0.966504	0.85935	0.394371
77	0.333333	0.96813	0.862331	0.409228
78	0.321951	0.964228	0.857182	0.385599
79	0.308943	0.968455	0.858537	0.386004
80	0.318699	0.964878	0.857182	0.384372
81	0.317073	0.967154	0.858808	0.389964
82	0.325203	0.965854	0.859079	0.393921
83	0.326829	0.967154	0.860434	0.399676
84	0.333333	0.962602	0.857724	0.391892
85	0.321951	0.966179	0.858808	0.391718
86	0.328455	0.961626	0.856098	0.38414

87	0.323577	0.966179	0.859079	0.393336
88	0.328455	0.96813	0.861518	0.404436
89	0.328455	0.96813	0.861518	0.404436
90	0.315447	0.96748	0.858808	0.389383
91	0.330081	0.967805	0.861518	0.404982
92	0.328455	0.966504	0.860163	0.399202
93	0.315447	0.964553	0.856369	0.380092
94	0.354472	0.963902	0.862331	0.416357
95	0.318699	0.964878	0.857182	0.384372
96	0.349593	0.963252	0.860976	0.409689
97	0.326829	0.960325	0.854743	0.378663
98	0.328455	0.964228	0.858266	0.392058
99	0.333333	0.965854	0.860434	0.401942
100	0.328455	0.963902	0.857995	0.391054
101	0.347967	0.961301	0.859079	0.402252
102	0.343089	0.963902	0.860434	0.40539
103	0.35122	0.965528	0.863144	0.418273
104	0.330081	0.961951	0.85664	0.386724
105	0.334959	0.961626	0.857182	0.390553
106	0.354472	0.963577	0.86206	0.415362
107	0.338211	0.960325	0.85664	0.38988
108	0.343089	0.963577	0.860163	0.404393
109	0.333333	0.962276	0.857453	0.390909
110	0.339837	0.96065	0.857182	0.392428
111	0.347967	0.960325	0.858266	0.399367
112	0.336585	0.962276	0.857995	0.394101
113	0.344715	0.960325	0.857724	0.396217
114	0.328455	0.961626	0.856098	0.38414
115	0.339837	0.961626	0.857995	0.395328
116	0.347967	0.96065	0.858537	0.400325
117	0.357724	0.962927	0.86206	0.416493
118	0.35122	0.964553	0.862331	0.415241
119	0.364228	0.957073	0.858266	0.405568
120	0.356098	0.959675	0.859079	0.405284
121	0.341463	0.961626	0.858266	0.396913
122	0.365854	0.96065	0.861518	0.417424
123	0.356098	0.962927	0.861789	0.414941
124	0.362602	0.958699	0.85935	0.408662
125	0.354472	0.96065	0.859621	0.406585
126	0.369106	0.962276	0.863415	0.425318
127	0.370732	0.953171	0.856098	0.400926
128	0.338211	0.961626	0.857724	0.39374
129	0.35122	0.962927	0.860976	0.410266

130	0.339837	0.960976	0.857453	0.393391
131	0.373984	0.955447	0.858537	0.410229
132	0.344715	0.96	0.857453	0.395263
133	0.352846	0.957398	0.85664	0.395613
134	0.35122	0.958049	0.856911	0.395902
135	0.344715	0.958699	0.856369	0.39148
136	0.352846	0.958374	0.857453	0.3984
137	0.35935	0.961301	0.860976	0.413171
138	0.35122	0.957724	0.85664	0.394973
139	0.360976	0.961626	0.861518	0.415685
140	0.352846	0.961301	0.859892	0.40695
141	0.349593	0.957724	0.856369	0.393404
142	0.346341	0.953496	0.852304	0.378476
143	0.372358	0.951545	0.855014	0.398092
144	0.344715	0.960325	0.857724	0.396217
145	0.372358	0.954472	0.857453	0.406007
146	0.364228	0.955122	0.85664	0.400111
147	0.339837	0.96	0.85664	0.390514
148	0.352846	0.962602	0.860976	0.410844
149	0.347967	0.953821	0.852846	0.380939
150	0.35935	0.955122	0.855827	0.395464
151	0.339837	0.957724	0.854743	0.383927
152	0.36748	0.96	0.861247	0.417055
153	0.378862	0.958374	0.861789	0.423042
154	0.385366	0.956098	0.860976	0.422663
155	0.357724	0.957073	0.857182	0.39937
156	0.35935	0.958374	0.858537	0.40463
157	0.349593	0.955772	0.854743	0.387899
158	0.354472	0.956748	0.856369	0.395335
159	0.343089	0.956748	0.854472	0.38433
160	0.372358	0.953496	0.85664	0.403342
161	0.362602	0.956098	0.857182	0.40128
162	0.364228	0.955772	0.857182	0.401917
163	0.343089	0.954146	0.852304	0.377091
164	0.356098	0.960325	0.859621	0.407186
165	0.365854	0.95252	0.854743	0.394555
166	0.35935	0.956423	0.856911	0.399091
167	0.365854	0.956098	0.857724	0.404368
168	0.349593	0.957398	0.856098	0.392478
169	0.354472	0.957073	0.85664	0.396254
170	0.347967	0.958374	0.85664	0.393695
171	0.356098	0.954472	0.854743	0.390556
172	0.38374	0.958374	0.862602	0.427582

173	0.385366	0.955447	0.860434	0.420856
174	0.35122	0.95122	0.85122	0.377074
175	0.369106	0.950569	0.853659	0.392436
176	0.372358	0.954797	0.857724	0.406901
177	0.362602	0.953171	0.854743	0.39322
178	0.370732	0.957724	0.859892	0.413564
179	0.37561	0.958049	0.860976	0.419072
180	0.328455	0.954797	0.850407	0.364501
181	0.36748	0.955772	0.857724	0.405
182	0.373984	0.957724	0.860434	0.416622
183	0.364228	0.958374	0.85935	0.409272
184	0.369106	0.952846	0.855556	0.398511
185	0.373984	0.955772	0.858808	0.411133
186	0.388618	0.958049	0.863144	0.43117
187	0.35122	0.954146	0.853659	0.384972
188	0.357724	0.948943	0.850407	0.377351
189	0.369106	0.95252	0.855285	0.397635
190	0.356098	0.95252	0.853117	0.385247
191	0.364228	0.954797	0.856369	0.399213
192	0.35935	0.950569	0.852033	0.383161
193	0.36748	0.95252	0.855014	0.396096
194	0.373984	0.949593	0.853659	0.394477
195	0.382114	0.955447	0.859892	0.417833
196	0.356098	0.951545	0.852304	0.382633
197	0.380488	0.954472	0.858808	0.413627
198	0.360976	0.95252	0.85393	0.389914
199	0.360976	0.950569	0.852304	0.384714
200	0.377236	0.948943	0.853659	0.395839
201	0.364228	0.955772	0.857182	0.401917
202	0.378862	0.952195	0.85664	0.405935
203	0.38374	0.954797	0.859621	0.41755
204	0.378862	0.949593	0.854472	0.399054
205	0.370732	0.95187	0.855014	0.397426
206	0.385366	0.949268	0.855285	0.404273
207	0.373984	0.95187	0.855556	0.40049
208	0.36748	0.952195	0.854743	0.395222
209	0.354472	0.95187	0.852304	0.38194
210	0.369106	0.954146	0.85664	0.402048
211	0.349593	0.953171	0.852575	0.380741
212	0.395122	0.950569	0.857995	0.416699
213	0.365854	0.95252	0.854743	0.394555
214	0.365854	0.950569	0.853117	0.389356
215	0.377236	0.948618	0.853388	0.394997

216	0.360976	0.95252	0.85393	0.389914
217	0.380488	0.948618	0.85393	0.39804
218	0.38374	0.948943	0.854743	0.401916
219	0.386992	0.946992	0.853659	0.399928
220	0.377236	0.949919	0.854472	0.398382
221	0.393496	0.951545	0.858537	0.417783
222	0.360976	0.95252	0.85393	0.389914
223	0.393496	0.953496	0.860163	0.423022
224	0.357724	0.955447	0.855827	0.394811
225	0.37561	0.949268	0.853659	0.395158
226	0.385366	0.955122	0.860163	0.419956
227	0.396748	0.953171	0.860434	0.425131
228	0.406504	0.948618	0.858266	0.422025
229	0.377236	0.947642	0.852575	0.392487
230	0.39187	0.95122	0.857995	0.41542
231	0.378862	0.95187	0.856369	0.405065
232	0.401626	0.95252	0.860705	0.427846
233	0.37561	0.953171	0.856911	0.405517
234	0.37561	0.950894	0.855014	0.399424
235	0.385366	0.95252	0.857995	0.412871
236	0.37561	0.94439	0.849593	0.382764
237	0.396748	0.949268	0.857182	0.41479
238	0.395122	0.95122	0.858537	0.418417
239	0.36748	0.95252	0.855014	0.396096
240	0.377236	0.948943	0.853659	0.395839
241	0.373984	0.949268	0.853388	0.39363
242	0.401626	0.950244	0.858808	0.421808
243	0.38374	0.942764	0.849593	0.386363
244	0.393496	0.949919	0.857182	0.413494
245	0.393496	0.946667	0.854472	0.405119
246	0.382114	0.950894	0.856098	0.40551
247	0.382114	0.954797	0.85935	0.416037
248	0.395122	0.946016	0.854201	0.404973
249	0.372358	0.948943	0.852846	0.391254
250	0.388618	0.949593	0.856098	0.408138
251	0.369106	0.953821	0.856369	0.401159
252	0.370732	0.948618	0.852304	0.388877
253	0.382114	0.947967	0.853659	0.397882
254	0.369106	0.949919	0.853117	0.390727
255	0.403252	0.948943	0.857995	0.419902
256	0.395122	0.946992	0.855014	0.407442
257	0.403252	0.950244	0.859079	0.423292
258	0.401626	0.950569	0.859079	0.422662

259	0.393496	0.948618	0.856098	0.410112
260	0.385366	0.954472	0.859621	0.418167
261	0.39187	0.950569	0.857453	0.413702
262	0.369106	0.948618	0.852033	0.387341
263	0.372358	0.951545	0.855014	0.398092
264	0.396748	0.947967	0.856098	0.41143
265	0.393496	0.947642	0.855285	0.407604
266	0.385366	0.947317	0.853659	0.399246
267	0.38374	0.949593	0.855285	0.403608
268	0.388618	0.943089	0.850678	0.391691
269	0.38374	0.944715	0.85122	0.391176
270	0.393496	0.949593	0.856911	0.412645
271	0.386992	0.950244	0.856369	0.408333
272	0.39187	0.947317	0.854743	0.405272
273	0.365854	0.944065	0.847696	0.372749
274	0.372358	0.948618	0.852575	0.390411
275	0.377236	0.945366	0.850678	0.386722
276	0.386992	0.948618	0.855014	0.404097
277	0.378862	0.948293	0.853388	0.39568
278	0.396748	0.947317	0.855556	0.409766
279	0.411382	0.947642	0.858266	0.423947
280	0.382114	0.945691	0.851762	0.3921
281	0.393496	0.94374	0.852033	0.397799
282	0.38374	0.948618	0.854472	0.401074
283	0.388618	0.945366	0.852575	0.397331
284	0.403252	0.943089	0.853117	0.405154
285	0.393496	0.945041	0.853117	0.401027
286	0.422764	0.946667	0.85935	0.43172
287	0.393496	0.946992	0.854743	0.405944
288	0.390244	0.943415	0.85122	0.393995
289	0.395122	0.948618	0.856369	0.41161
290	0.395122	0.945691	0.85393	0.404155
291	0.398374	0.948293	0.85664	0.413759
292	0.398374	0.948293	0.85664	0.413759
293	0.404878	0.947967	0.857453	0.418871
294	0.360976	0.947967	0.850136	0.377941
295	0.373984	0.947317	0.851762	0.388601
296	0.4	0.94374	0.853117	0.403778
297	0.398374	0.944715	0.853659	0.404704
298	0.382114	0.945041	0.85122	0.390471
299	0.406504	0.94439	0.854743	0.411328
300	0.401626	0.945691	0.855014	0.410123
301	0.393496	0.945366	0.853388	0.401841

302	0.398374	0.950894	0.858808	0.420542
303	0.377236	0.947967	0.852846	0.393321
304	0.388618	0.944065	0.851491	0.394093
305	0.378862	0.948618	0.853659	0.39652
306	0.404878	0.94439	0.854472	0.409846
307	0.413008	0.948943	0.859621	0.428764
308	0.395122	0.944065	0.852575	0.400101
309	0.380488	0.940488	0.847154	0.377825
310	0.38374	0.950244	0.855827	0.405311
311	0.396748	0.950569	0.858266	0.418193
312	0.401626	0.946992	0.856098	0.41341
313	0.395122	0.94439	0.852846	0.400907
314	0.385366	0.946016	0.852575	0.395947
315	0.409756	0.940488	0.852033	0.404772
316	0.396748	0.941463	0.850678	0.395237
317	0.39187	0.954797	0.860976	0.425076
318	0.41626	0.950244	0.861247	0.435085
319	0.409756	0.94439	0.855285	0.414284
320	0.398374	0.948618	0.856911	0.414598
321	0.403252	0.945366	0.855014	0.410794
322	0.396748	0.944065	0.852846	0.401597
323	0.4	0.944715	0.85393	0.406195
324	0.395122	0.946341	0.854472	0.405793
325	0.39187	0.946016	0.853659	0.401974
326	0.364228	0.949593	0.852033	0.38525
327	0.411382	0.949919	0.860163	0.42983
328	0.386992	0.944715	0.851762	0.394199
329	0.388618	0.946992	0.85393	0.401435
330	0.393496	0.947642	0.855285	0.407604
331	0.38374	0.94439	0.850949	0.390367
332	0.390244	0.947317	0.854472	0.403769
333	0.417886	0.946016	0.857995	0.425697
334	0.39187	0.950569	0.857453	0.413702
335	0.385366	0.948618	0.854743	0.402586
336	0.401626	0.939837	0.850136	0.39581
337	0.401626	0.948943	0.857724	0.418417
338	0.40813	0.942439	0.853388	0.408009
339	0.4	0.947967	0.85664	0.414414
340	0.40813	0.944065	0.854743	0.412001
341	0.422764	0.944065	0.857182	0.425213
342	0.403252	0.939837	0.850407	0.397296
343	0.409756	0.950244	0.860163	0.429208
344	0.372358	0.94374	0.848509	0.378096

345	0.395122	0.942439	0.85122	0.396108
346	0.395122	0.942114	0.850949	0.395317
347	0.409756	0.945366	0.856098	0.416715
348	0.39187	0.945366	0.853117	0.40034
349	0.37561	0.947642	0.852304	0.390961
350	0.403252	0.946667	0.856098	0.41407
351	0.390244	0.949919	0.85664	0.410492
352	0.41626	0.949593	0.860705	0.433387
353	0.4	0.948943	0.857453	0.416929
354	0.377236	0.946016	0.85122	0.388356
355	0.393496	0.947642	0.855285	0.407604
356	0.40813	0.942114	0.853117	0.407218
357	0.393496	0.942439	0.850949	0.39461
358	0.396748	0.940163	0.849593	0.392113
359	0.395122	0.940488	0.849593	0.391395
360	0.403252	0.946992	0.856369	0.414896
361	0.396748	0.947642	0.855827	0.410597
362	0.388618	0.942439	0.850136	0.390101
363	0.417886	0.946341	0.858266	0.426516
364	0.413008	0.94374	0.855285	0.415623
365	0.4	0.942764	0.852304	0.401382
366	0.393496	0.946667	0.854472	0.405119
367	0.393496	0.948618	0.856098	0.410112
368	0.403252	0.944715	0.854472	0.40917
369	0.393496	0.947967	0.855556	0.408437
370	0.401626	0.947317	0.856369	0.414238
371	0.395122	0.948943	0.85664	0.412451
372	0.398374	0.947317	0.855827	0.411259
373	0.385366	0.946016	0.852575	0.395947
374	0.4	0.945691	0.854743	0.408634
375	0.404878	0.941789	0.852304	0.403467
376	0.396748	0.942114	0.85122	0.396813
377	0.403252	0.940488	0.850949	0.39885
378	0.395122	0.947967	0.855827	0.409935
379	0.386992	0.945366	0.852304	0.395823
380	0.38374	0.947967	0.85393	0.399398
381	0.409756	0.946016	0.85664	0.418348
382	0.419512	0.939512	0.852846	0.411262
383	0.404878	0.943415	0.853659	0.407436
384	0.390244	0.946667	0.85393	0.402115
385	0.396748	0.943089	0.852033	0.399194
386	0.393496	0.945041	0.853117	0.401027
387	0.421138	0.94439	0.857182	0.424559

388	0.414634	0.943415	0.855285	0.416292
389	0.398374	0.942114	0.851491	0.398306
390	0.403252	0.946667	0.856098	0.41407
391	0.40813	0.940813	0.852033	0.404075
392	0.401626	0.94374	0.853388	0.405267
393	0.388618	0.940488	0.848509	0.385388
394	0.414634	0.945041	0.85664	0.420319
395	0.403252	0.947317	0.85664	0.415724
396	0.406504	0.941138	0.852033	0.403378
397	0.39187	0.941138	0.849593	0.389958
398	0.395122	0.943415	0.852033	0.398497
399	0.380488	0.942114	0.848509	0.381745
400	0.390244	0.944715	0.852304	0.397213
401	0.38374	0.956098	0.860705	0.421154
402	0.411382	0.943415	0.854743	0.413349
403	0.385366	0.94439	0.85122	0.39188
404	0.403252	0.947317	0.85664	0.415724
405	0.403252	0.941463	0.851762	0.401196
406	0.403252	0.95252	0.860976	0.429329
407	0.398374	0.945041	0.85393	0.405515
408	0.395122	0.944715	0.853117	0.401715
409	0.40813	0.94374	0.854472	0.411198
410	0.421138	0.946992	0.85935	0.431086
411	0.396748	0.946992	0.855285	0.408938
412	0.4	0.940163	0.850136	0.395097
413	0.395122	0.94374	0.852304	0.399298
414	0.404878	0.953821	0.862331	0.434321
415	0.404878	0.937886	0.849051	0.394174
416	0.41626	0.946992	0.858537	0.426698
417	0.422764	0.941138	0.854743	0.418072
418	0.40813	0.944065	0.854743	0.412001
419	0.411382	0.939837	0.851762	0.404694
420	0.409756	0.943415	0.854472	0.411875
421	0.406504	0.938862	0.850136	0.397949
422	0.413008	0.941789	0.853659	0.410853
423	0.395122	0.941789	0.850678	0.394528
424	0.40813	0.945041	0.855556	0.414425
425	0.413008	0.946992	0.857995	0.423762
426	0.419512	0.944065	0.85664	0.422293
427	0.396748	0.943089	0.852033	0.399194
428	0.40813	0.942114	0.853117	0.407218
429	0.388618	0.941463	0.849322	0.387734
430	0.396748	0.946341	0.854743	0.407289

431	0.411382	0.941138	0.852846	0.407809
432	0.395122	0.946992	0.855014	0.407442
433	0.396748	0.943089	0.852033	0.399194
434	0.395122	0.940488	0.849593	0.391395
435	0.404878	0.946341	0.856098	0.414731
436	0.39187	0.947642	0.855014	0.406103
437	0.403252	0.935935	0.847154	0.388161
438	0.393496	0.950894	0.857995	0.416059
439	0.39187	0.942114	0.850407	0.392318
440	0.396748	0.943089	0.852033	0.399194
441	0.4	0.945041	0.854201	0.407006
442	0.401626	0.941463	0.851491	0.39971
443	0.419512	0.946992	0.859079	0.429626
444	0.409756	0.940488	0.852033	0.404772
445	0.414634	0.941463	0.853659	0.411537
446	0.401626	0.934309	0.845528	0.382959
447	0.39187	0.947317	0.854743	0.405272
448	0.404878	0.947642	0.857182	0.418038
449	0.388618	0.942114	0.849864	0.389309
450	0.393496	0.942439	0.850949	0.39461
451	0.406504	0.944715	0.855014	0.412136
452	0.40813	0.944065	0.854743	0.412001
453	0.403252	0.945366	0.855014	0.410794
454	0.386992	0.946016	0.852846	0.397457
455	0.385366	0.939837	0.847425	0.380818
456	0.414634	0.939187	0.851762	0.406092
457	0.382114	0.942114	0.84878	0.383263
458	0.390244	0.94439	0.852033	0.396405
459	0.409756	0.942764	0.85393	0.41028
460	0.395122	0.940813	0.849864	0.392174
461	0.413008	0.942439	0.854201	0.412434
462	0.413008	0.945366	0.85664	0.419662
463	0.419512	0.939512	0.852846	0.411262
464	0.413008	0.943415	0.855014	0.414822
465	0.422764	0.940488	0.854201	0.41651
466	0.396748	0.937236	0.847154	0.385215
467	0.390244	0.947967	0.855014	0.405434
468	0.377236	0.944715	0.850136	0.385098
469	0.398374	0.946341	0.855014	0.408782
470	0.403252	0.942764	0.852846	0.404358
471	0.401626	0.939512	0.849864	0.395037
472	0.404878	0.940813	0.851491	0.401114
473	0.421138	0.94374	0.85664	0.422951

474	0.396748	0.940488	0.849864	0.39289
475	0.395122	0.943415	0.852033	0.398497
476	0.385366	0.945691	0.852304	0.395128
477	0.404878	0.939187	0.850136	0.397236
478	0.396748	0.945041	0.853659	0.404021
479	0.419512	0.940813	0.85393	0.414369
480	0.395122	0.942114	0.850949	0.395317
481	0.390244	0.944065	0.851762	0.395599
482	0.411382	0.943415	0.854743	0.413349
483	0.40813	0.938862	0.850407	0.399428
484	0.414634	0.945366	0.856911	0.421132
485	0.396748	0.945691	0.854201	0.40565
486	0.406504	0.94439	0.854743	0.411328
487	0.406504	0.94439	0.854743	0.411328
488	0.406504	0.940488	0.851491	0.401815
489	0.413008	0.94439	0.855827	0.417231
490	0.38374	0.942764	0.849593	0.386363
491	0.40813	0.939512	0.850949	0.400968
492	0.40813	0.940488	0.851762	0.403295
493	0.41626	0.944715	0.85664	0.420977
494	0.411382	0.944065	0.855285	0.414953
495	0.414634	0.940488	0.852846	0.40919
496	0.401626	0.945041	0.854472	0.408494
497	0.396748	0.946341	0.854743	0.407289
498	0.41626	0.938537	0.851491	0.406025
499	0.4	0.941463	0.85122	0.398221
500	0.38374	0.942764	0.849593	0.386363

(5) Dataset S_5

Number of features	SN	SP	ACC	MCC
4	0.089431	0.993821	0.843089	0.221322
5	0.133333	0.988293	0.845799	0.257626
6	0.160976	0.988293	0.850407	0.296306
7	0.198374	0.986667	0.855285	0.335607
8	0.222764	0.98439	0.857453	0.353773
9	0.217886	0.983415	0.855827	0.343776
10	0.234146	0.980488	0.856098	0.35001
11	0.243902	0.981138	0.858266	0.363643
12	0.255285	0.979837	0.859079	0.370855
13	0.261789	0.978862	0.85935	0.374028
14	0.289431	0.976585	0.86206	0.394475
15	0.286179	0.977561	0.862331	0.394783

16	0.287805	0.977561	0.862602	0.396466
17	0.292683	0.978862	0.864499	0.406466
18	0.297561	0.977236	0.863957	0.405249
19	0.305691	0.97561	0.863957	0.407471
20	0.323577	0.973659	0.865312	0.418316
21	0.304065	0.97626	0.864228	0.408215
22	0.315447	0.973659	0.863957	0.410256
23	0.318699	0.977236	0.86748	0.426431
24	0.318699	0.972683	0.863686	0.410078
25	0.308943	0.974309	0.863415	0.406051
26	0.317073	0.974309	0.86477	0.41418
27	0.323577	0.973659	0.865312	0.418316
28	0.326829	0.972358	0.86477	0.416989
29	0.325203	0.974634	0.866396	0.423369
30	0.313821	0.973333	0.863415	0.407487
31	0.326829	0.974309	0.866396	0.423809
32	0.325203	0.971707	0.863957	0.413156
33	0.320325	0.973984	0.865041	0.416251
34	0.330081	0.973008	0.865854	0.422428
35	0.321951	0.970407	0.862331	0.40553
36	0.317073	0.972683	0.863415	0.40846
37	0.344715	0.971707	0.867209	0.432144
38	0.343089	0.969106	0.86477	0.421901
39	0.333333	0.969431	0.863415	0.41349
40	0.326829	0.973984	0.866125	0.422659
41	0.331707	0.972683	0.865854	0.42289
42	0.347967	0.968455	0.865041	0.424476
43	0.336585	0.972683	0.866667	0.427635
44	0.343089	0.969756	0.865312	0.424043
45	0.354472	0.968455	0.866125	0.430697
46	0.35935	0.971057	0.869106	0.443884
47	0.343089	0.967154	0.863144	0.415582
48	0.364228	0.969756	0.868835	0.444168
49	0.360976	0.969756	0.868293	0.441107
50	0.349593	0.967805	0.86477	0.423935
51	0.356098	0.970407	0.868022	0.438641
52	0.354472	0.969756	0.867209	0.434947
53	0.331707	0.968455	0.862331	0.408693
54	0.349593	0.96748	0.864499	0.42289
55	0.35122	0.96748	0.86477	0.424448
56	0.347967	0.970081	0.866396	0.429811
57	0.35122	0.969431	0.866396	0.430777
58	0.352846	0.967805	0.865312	0.427047

59	0.347967	0.96813	0.86477	0.423423
60	0.349593	0.968455	0.865312	0.426036
61	0.349593	0.965528	0.862873	0.416712
62	0.382114	0.965203	0.868022	0.446355
63	0.364228	0.965203	0.865041	0.429641
64	0.370732	0.96748	0.868022	0.442907
65	0.372358	0.965854	0.866938	0.439303
66	0.357724	0.965203	0.863957	0.423474
67	0.344715	0.966179	0.862602	0.414054
68	0.369106	0.964553	0.865312	0.432226
69	0.338211	0.965854	0.861247	0.406714
70	0.347967	0.96813	0.86477	0.423423
71	0.373984	0.964228	0.865854	0.435799
72	0.357724	0.96813	0.866396	0.432738
73	0.35122	0.965528	0.863144	0.418273
74	0.382114	0.964878	0.867751	0.445353
75	0.377236	0.963252	0.865583	0.435867
76	0.352846	0.965528	0.863415	0.419831
77	0.37561	0.957724	0.860705	0.418146
78	0.35935	0.96748	0.866125	0.432193
79	0.362602	0.966504	0.865854	0.432174
80	0.385366	0.965528	0.868835	0.450362
81	0.36748	0.964553	0.865041	0.430697
82	0.369106	0.964228	0.865041	0.431228
83	0.35935	0.965528	0.864499	0.426033
84	0.356098	0.962602	0.861518	0.413959
85	0.377236	0.960976	0.863686	0.429076
86	0.377236	0.963902	0.866125	0.43784
87	0.357724	0.964228	0.863144	0.420459
88	0.362602	0.964228	0.863957	0.425092
89	0.372358	0.96065	0.862602	0.423553
90	0.36748	0.958374	0.859892	0.412352
91	0.37561	0.965203	0.866938	0.440317
92	0.4	0.963252	0.869377	0.456808
93	0.386992	0.959675	0.864228	0.434336
94	0.385366	0.965854	0.869106	0.45137
95	0.385366	0.963902	0.86748	0.445376
96	0.386992	0.960325	0.86477	0.436227
97	0.401626	0.960325	0.867209	0.449638
98	0.37561	0.96	0.862602	0.424698
99	0.393496	0.96065	0.866125	0.443163
100	0.369106	0.957398	0.85935	0.411107
101	0.382114	0.962276	0.865583	0.437471

102	0.38374	0.961626	0.865312	0.437044
103	0.370732	0.961301	0.862873	0.423943
104	0.385366	0.958374	0.862873	0.429089
105	0.377236	0.958049	0.861247	0.420594
106	0.38374	0.959675	0.863686	0.431326
107	0.372358	0.960325	0.862331	0.422599
108	0.37561	0.958374	0.861247	0.420002
109	0.364228	0.95935	0.860163	0.412085
110	0.390244	0.96065	0.865583	0.440176
111	0.393496	0.958374	0.864228	0.436589
112	0.386992	0.96	0.864499	0.43528
113	0.390244	0.959024	0.864228	0.43546
114	0.395122	0.96065	0.866396	0.444652
115	0.38374	0.96	0.863957	0.43227
116	0.373984	0.961626	0.863686	0.427959
117	0.386992	0.957724	0.862602	0.428743
118	0.398374	0.958699	0.865312	0.441985
119	0.406504	0.957398	0.865583	0.445691
120	0.386992	0.959675	0.864228	0.434336
121	0.388618	0.959024	0.863957	0.433961
122	0.370732	0.961301	0.862873	0.423943
123	0.396748	0.956748	0.863415	0.434974
124	0.390244	0.96065	0.865583	0.440176
125	0.380488	0.95935	0.862873	0.427363
126	0.390244	0.961626	0.866396	0.443045
127	0.380488	0.955772	0.859892	0.41722
128	0.38374	0.956423	0.860976	0.422063
129	0.398374	0.958049	0.86477	0.440132
130	0.4	0.956748	0.863957	0.437948
131	0.398374	0.959024	0.865583	0.442917
132	0.40813	0.962276	0.869919	0.461254
133	0.403252	0.954146	0.862331	0.433726
134	0.4	0.957724	0.86477	0.440694
135	0.401626	0.956098	0.863686	0.437616
136	0.404878	0.956423	0.864499	0.441482
137	0.403252	0.956423	0.864228	0.440003
138	0.393496	0.957724	0.863686	0.434739
139	0.385366	0.955122	0.860163	0.419956
140	0.393496	0.95187	0.858808	0.418649
141	0.385366	0.958049	0.862602	0.428162
142	0.404878	0.956748	0.86477	0.44239
143	0.388618	0.957398	0.862602	0.429326
144	0.404878	0.957073	0.865041	0.443302

145	0.393496	0.954472	0.860976	0.425681
146	0.404878	0.953821	0.862331	0.434321
147	0.40813	0.956423	0.865041	0.444431
148	0.413008	0.958374	0.86748	0.454325
149	0.409756	0.954472	0.863686	0.440519
150	0.40813	0.955447	0.864228	0.441725
151	0.404878	0.953496	0.86206	0.433439
152	0.419512	0.958699	0.868835	0.461082
153	0.413008	0.954146	0.863957	0.442571
154	0.409756	0.951545	0.861247	0.432638
155	0.40813	0.953496	0.862602	0.436393
156	0.417886	0.95252	0.863415	0.442574
157	0.403252	0.953171	0.861518	0.431079
158	0.427642	0.954472	0.866667	0.456563
159	0.390244	0.95187	0.858266	0.415649
160	0.403252	0.951545	0.860163	0.426726
161	0.413008	0.95252	0.862602	0.438181
162	0.413008	0.956748	0.866125	0.449744
163	0.406504	0.956748	0.865041	0.443866
164	0.417886	0.956098	0.866396	0.452319
165	0.422764	0.950894	0.862873	0.442633
166	0.42439	0.953821	0.865583	0.4519
167	0.393496	0.953496	0.860163	0.423022
168	0.398374	0.954472	0.861789	0.430159
169	0.409756	0.953496	0.862873	0.437867
170	0.409756	0.953171	0.862602	0.436988
171	0.421138	0.953171	0.864499	0.447237
172	0.41626	0.95252	0.863144	0.441112
173	0.409756	0.954146	0.863415	0.439632
174	0.442276	0.956098	0.870461	0.473935
175	0.396748	0.95122	0.858808	0.419911
176	0.411382	0.954146	0.863686	0.441103
177	0.426016	0.95252	0.86477	0.449851
178	0.38374	0.953821	0.858808	0.414879
179	0.411382	0.954146	0.863686	0.441103
180	0.404878	0.950244	0.85935	0.424775
181	0.426016	0.950569	0.863144	0.444684
182	0.422764	0.948943	0.861247	0.437542
183	0.413008	0.947642	0.858537	0.425419
184	0.427642	0.954472	0.866667	0.456563
185	0.411382	0.954797	0.864228	0.442879
186	0.419512	0.951545	0.862873	0.441436
187	0.422764	0.952195	0.863957	0.446079

188	0.422764	0.946667	0.85935	0.43172
189	0.413008	0.946992	0.857995	0.423762
190	0.42439	0.948618	0.861247	0.438158
191	0.419512	0.95122	0.862602	0.440576
192	0.406504	0.948618	0.858266	0.422025
193	0.422764	0.948618	0.860976	0.436703
194	0.417886	0.951545	0.862602	0.439976
195	0.435772	0.95252	0.866396	0.458508
196	0.411382	0.950894	0.860976	0.43239
197	0.406504	0.952195	0.861247	0.431419
198	0.411382	0.952846	0.862602	0.437584
199	0.417886	0.950244	0.861518	0.436549
200	0.421138	0.949919	0.861789	0.438619
201	0.447154	0.950894	0.866938	0.464212
202	0.43252	0.95187	0.865312	0.4539
203	0.419512	0.952846	0.863957	0.444906
204	0.413008	0.95252	0.862602	0.438181
205	0.406504	0.948618	0.858266	0.422025
206	0.413008	0.952195	0.862331	0.437311
207	0.430894	0.953171	0.866125	0.455932
208	0.417886	0.949919	0.861247	0.435698
209	0.439024	0.948618	0.863686	0.451157
210	0.429268	0.948293	0.861789	0.441675
211	0.41626	0.949919	0.860976	0.434235
212	0.403252	0.95122	0.859892	0.425863
213	0.422764	0.950894	0.862873	0.442633
214	0.426016	0.946992	0.860163	0.435454
215	0.421138	0.949268	0.861247	0.436927
216	0.421138	0.949919	0.861789	0.438619
217	0.417886	0.948618	0.860163	0.432324
218	0.429268	0.949268	0.862602	0.444191
219	0.411382	0.952195	0.86206	0.435842
220	0.430894	0.953821	0.866667	0.457685
221	0.411382	0.95187	0.861789	0.434975
222	0.404878	0.950894	0.859892	0.426486
223	0.421138	0.953496	0.86477	0.448114
224	0.401626	0.945041	0.854472	0.408494
225	0.426016	0.946016	0.85935	0.432989
226	0.417886	0.95252	0.863415	0.442574
227	0.42439	0.950244	0.862602	0.44238
228	0.437398	0.950569	0.865041	0.454786
229	0.43252	0.950244	0.863957	0.449619
230	0.43252	0.947967	0.86206	0.443732

231	0.398374	0.944715	0.853659	0.404704
232	0.44065	0.945366	0.861247	0.44435
233	0.426016	0.953496	0.865583	0.452471
234	0.421138	0.950244	0.86206	0.439469
235	0.437398	0.946341	0.861518	0.443925
236	0.413008	0.942439	0.854201	0.412434
237	0.42439	0.948618	0.861247	0.438158
238	0.421138	0.949593	0.861518	0.437772
239	0.429268	0.948618	0.86206	0.442511
240	0.422764	0.951545	0.863415	0.44435
241	0.422764	0.946341	0.859079	0.430898
242	0.422764	0.948618	0.860976	0.436703
243	0.43252	0.947967	0.86206	0.443732
244	0.442276	0.948293	0.863957	0.453187
245	0.427642	0.948293	0.861518	0.440226
246	0.395122	0.953821	0.860705	0.425401
247	0.422764	0.948293	0.860705	0.435866
248	0.41626	0.947317	0.858808	0.427525
249	0.414634	0.948943	0.859892	0.430233
250	0.442276	0.947317	0.863144	0.450697
251	0.44878	0.945041	0.862331	0.450684
252	0.44878	0.94374	0.861247	0.447466
253	0.422764	0.950569	0.862602	0.441778
254	0.460163	0.949268	0.867751	0.471303
255	0.44878	0.946016	0.863144	0.453121
256	0.430894	0.945691	0.859892	0.436522
257	0.429268	0.949593	0.862873	0.445034
258	0.445528	0.946341	0.862873	0.451088
259	0.44065	0.945691	0.861518	0.445163
260	0.422764	0.945041	0.857995	0.427635
261	0.430894	0.949268	0.862873	0.445637
262	0.43252	0.948293	0.862331	0.444566
263	0.434146	0.946667	0.861247	0.441867
264	0.439024	0.950244	0.865041	0.45537
265	0.427642	0.946341	0.859892	0.43526
266	0.44065	0.94374	0.859892	0.440318
267	0.42439	0.949268	0.861789	0.439839
268	0.443902	0.948293	0.864228	0.454616
269	0.422764	0.945366	0.858266	0.428447
270	0.443902	0.941789	0.858808	0.438422
271	0.422764	0.943089	0.856369	0.422812
272	0.430894	0.948618	0.862331	0.443957
273	0.439024	0.94374	0.859621	0.438883

274	0.437398	0.946016	0.861247	0.443107
275	0.447154	0.941463	0.859079	0.440494
276	0.430894	0.944065	0.858537	0.432476
277	0.44878	0.947967	0.86477	0.45806
278	0.442276	0.944065	0.860434	0.442553
279	0.426016	0.948618	0.861518	0.439611
280	0.426016	0.948618	0.861518	0.439611
281	0.429268	0.947642	0.861247	0.44001
282	0.447154	0.941789	0.85935	0.44128
283	0.426016	0.949268	0.86206	0.441292
284	0.43252	0.947317	0.861518	0.442073
285	0.439024	0.946341	0.861789	0.445362
286	0.452033	0.947967	0.865312	0.460901
287	0.44065	0.951545	0.866396	0.460218
288	0.44878	0.940813	0.858808	0.440355
289	0.437398	0.949593	0.864228	0.452242
290	0.445528	0.947642	0.863957	0.454382
291	0.453659	0.946341	0.864228	0.458199
292	0.450407	0.943089	0.860976	0.447294
293	0.435772	0.946667	0.861518	0.443307
294	0.427642	0.946992	0.860434	0.436906
295	0.439024	0.947642	0.862873	0.448659
296	0.453659	0.946992	0.86477	0.45984
297	0.447154	0.943415	0.860705	0.445241
298	0.460163	0.945041	0.864228	0.460602
299	0.42439	0.943089	0.85664	0.424269
300	0.447154	0.945366	0.862331	0.450069
301	0.437398	0.943089	0.858808	0.435848
302	0.478049	0.94439	0.866667	0.474387
303	0.43252	0.946341	0.860705	0.439602
304	0.421138	0.945366	0.857995	0.426989
305	0.435772	0.945366	0.860434	0.440038
306	0.437398	0.940813	0.856911	0.430329
307	0.453659	0.941789	0.860434	0.446972
308	0.447154	0.940488	0.858266	0.43815
309	0.443902	0.948943	0.86477	0.456288
310	0.435772	0.945691	0.860705	0.440852
311	0.445528	0.943415	0.860434	0.443814
312	0.447154	0.94439	0.861518	0.447645
313	0.460163	0.942764	0.862331	0.455
314	0.429268	0.947317	0.860976	0.439181
315	0.421138	0.942764	0.855827	0.420557
316	0.44065	0.940163	0.856911	0.431647

317	0.44065	0.940488	0.857182	0.432425
318	0.442276	0.942114	0.858808	0.437778
319	0.442276	0.948943	0.864499	0.454859
320	0.443902	0.942764	0.859621	0.440792
321	0.442276	0.94374	0.860163	0.441752
322	0.456911	0.944065	0.862873	0.455363
323	0.434146	0.950244	0.864228	0.45106
324	0.458537	0.948293	0.866667	0.467389
325	0.456911	0.94374	0.862602	0.454562
326	0.435772	0.946016	0.860976	0.441668
327	0.450407	0.938211	0.856911	0.435606
328	0.450407	0.945691	0.863144	0.453729
329	0.439024	0.942439	0.858537	0.435698
330	0.434146	0.944715	0.859621	0.436977
331	0.434146	0.943089	0.858266	0.432966
332	0.453659	0.940488	0.85935	0.443844
333	0.437398	0.944065	0.859621	0.438247
334	0.460163	0.942114	0.861789	0.45342
335	0.460163	0.942114	0.861789	0.45342
336	0.442276	0.940488	0.857453	0.433859
337	0.44065	0.941789	0.858266	0.435556
338	0.447154	0.946667	0.863415	0.453334
339	0.461789	0.937886	0.858537	0.444764
340	0.435772	0.94374	0.859079	0.436005
341	0.439024	0.937561	0.854472	0.424068
342	0.456911	0.946016	0.864499	0.460213
343	0.442276	0.938537	0.855827	0.429227
344	0.453659	0.941463	0.860163	0.446187
345	0.455285	0.943089	0.861789	0.451552
346	0.430894	0.939837	0.855014	0.422222
347	0.434146	0.943415	0.858537	0.433764
348	0.443902	0.94374	0.860434	0.443183
349	0.461789	0.940813	0.860976	0.451695
350	0.453659	0.940813	0.859621	0.444622
351	0.437398	0.943415	0.859079	0.436645
352	0.455285	0.940813	0.859892	0.446041
353	0.44878	0.942114	0.859892	0.443494
354	0.445528	0.94439	0.861247	0.446218
355	0.44878	0.941463	0.85935	0.44192
356	0.455285	0.943415	0.86206	0.452349
357	0.450407	0.945366	0.862873	0.452917
358	0.429268	0.940813	0.855556	0.423106
359	0.42439	0.945691	0.858808	0.430718

360	0.452033	0.941463	0.859892	0.444766
361	0.445528	0.943415	0.860434	0.443814
362	0.43252	0.941138	0.856369	0.426784
363	0.445528	0.94439	0.861247	0.446218
364	0.458537	0.940163	0.859892	0.447317
365	0.430894	0.943089	0.857724	0.430076
366	0.463415	0.940813	0.861247	0.453104
367	0.435772	0.942764	0.858266	0.433613
368	0.434146	0.948943	0.863144	0.447682
369	0.456911	0.942764	0.861789	0.452174
370	0.443902	0.945366	0.861789	0.447214
371	0.453659	0.945041	0.863144	0.454946
372	0.434146	0.941463	0.856911	0.429012
373	0.453659	0.942114	0.860705	0.447759
374	0.429268	0.94439	0.858537	0.431832
375	0.44878	0.945041	0.862331	0.450684
376	0.43252	0.941138	0.856369	0.426784
377	0.469919	0.942439	0.863686	0.462641
378	0.43252	0.94439	0.859079	0.434726
379	0.450407	0.941138	0.85935	0.442561
380	0.43252	0.940813	0.856098	0.426002
381	0.421138	0.945691	0.858266	0.427803
382	0.435772	0.941789	0.857453	0.431241
383	0.437398	0.940163	0.856369	0.428772
384	0.435772	0.94374	0.859079	0.436005
385	0.452033	0.94439	0.862331	0.451914
386	0.437398	0.941138	0.857182	0.431111
387	0.439024	0.942439	0.858537	0.435698
388	0.443902	0.945691	0.86206	0.448027
389	0.41626	0.939187	0.852033	0.407561
390	0.439024	0.946992	0.862331	0.447006
391	0.430894	0.947967	0.861789	0.442288
392	0.437398	0.939512	0.855827	0.427224
393	0.44065	0.94439	0.860434	0.441924
394	0.445528	0.945691	0.862331	0.449455
395	0.456911	0.941138	0.860434	0.448238
396	0.430894	0.940813	0.855827	0.424555
397	0.427642	0.946341	0.859892	0.43526
398	0.430894	0.947642	0.861518	0.441457
399	0.437398	0.937561	0.854201	0.42263
400	0.44878	0.939837	0.857995	0.438025
401	0.419512	0.945691	0.857995	0.426343
402	0.439024	0.942439	0.858537	0.435698

403	0.422764	0.939512	0.853388	0.414183
404	0.435772	0.943415	0.858808	0.435206
405	0.460163	0.942114	0.861789	0.45342
406	0.439024	0.950244	0.865041	0.45537
407	0.442276	0.948293	0.863957	0.453187
408	0.429268	0.945041	0.859079	0.433448
409	0.455285	0.939187	0.858537	0.442168
410	0.426016	0.940813	0.855014	0.420202
411	0.44878	0.942439	0.860163	0.444283
412	0.437398	0.943415	0.859079	0.436645
413	0.430894	0.938211	0.853659	0.418377
414	0.434146	0.944065	0.859079	0.435365
415	0.445528	0.938537	0.856369	0.432091
416	0.395122	0.946992	0.855014	0.407442
417	0.43252	0.944065	0.858808	0.433922
418	0.435772	0.940488	0.856369	0.428109
419	0.434146	0.937236	0.853388	0.418987
420	0.455285	0.945691	0.863957	0.457985
421	0.429268	0.938211	0.853388	0.416928
422	0.455285	0.941138	0.860163	0.446822
423	0.430894	0.942764	0.857453	0.42928
424	0.421138	0.941138	0.854472	0.416612
425	0.417886	0.941138	0.85393	0.413687
426	0.453659	0.943415	0.861789	0.450931
427	0.465041	0.942439	0.862873	0.458433
428	0.44065	0.939837	0.85664	0.430872
429	0.43252	0.939187	0.854743	0.422125
430	0.419512	0.945366	0.857724	0.425528
431	0.427642	0.938862	0.853659	0.417009
432	0.450407	0.941789	0.859892	0.44413
433	0.458537	0.939837	0.859621	0.446542
434	0.429268	0.94374	0.857995	0.430225
435	0.447154	0.942114	0.859621	0.442068
436	0.455285	0.937236	0.856911	0.437589
437	0.426016	0.939512	0.85393	0.417096
438	0.445528	0.944065	0.860976	0.445414
439	0.435772	0.939187	0.855285	0.425012
440	0.453659	0.939837	0.858808	0.442292
441	0.455285	0.938862	0.858266	0.4414
442	0.439024	0.94374	0.859621	0.438883
443	0.435772	0.942764	0.858266	0.433613
444	0.429268	0.945041	0.859079	0.433448
445	0.445528	0.939187	0.856911	0.433626

446	0.453659	0.941789	0.860434	0.446972
447	0.43252	0.937886	0.853659	0.419062
448	0.439024	0.942439	0.858537	0.435698
449	0.434146	0.942439	0.857724	0.431378
450	0.44878	0.942764	0.860434	0.445076
451	0.458537	0.942764	0.86206	0.453588
452	0.435772	0.938862	0.855014	0.424243
453	0.434146	0.937236	0.853388	0.418987
454	0.437398	0.941463	0.857453	0.431895
455	0.419512	0.943415	0.856098	0.42069
456	0.44878	0.942764	0.860434	0.445076
457	0.439024	0.941789	0.857995	0.434119
458	0.44065	0.946341	0.86206	0.446797
459	0.450407	0.944715	0.862331	0.451299
460	0.447154	0.941789	0.85935	0.44128
461	0.434146	0.943089	0.858266	0.432966
462	0.427642	0.936911	0.852033	0.412439
463	0.447154	0.945691	0.862602	0.450882
464	0.452033	0.946992	0.864499	0.458422
465	0.439024	0.937236	0.854201	0.42331
466	0.44065	0.940813	0.857453	0.433204
467	0.443902	0.940163	0.857453	0.434514
468	0.43252	0.937561	0.853388	0.418301
469	0.442276	0.942114	0.858808	0.437778
470	0.458537	0.941138	0.860705	0.449652
471	0.453659	0.945366	0.863415	0.455756
472	0.447154	0.939837	0.857724	0.436598
473	0.442276	0.942114	0.858808	0.437778
474	0.44878	0.940813	0.858808	0.440355
475	0.443902	0.941463	0.858537	0.437636
476	0.445528	0.940488	0.857995	0.436722
477	0.442276	0.939512	0.85664	0.431534
478	0.43252	0.942764	0.857724	0.430727
479	0.453659	0.937886	0.857182	0.437688
480	0.434146	0.943089	0.858266	0.432966
481	0.452033	0.941138	0.859621	0.443983
482	0.452033	0.94374	0.861789	0.45031
483	0.455285	0.940813	0.859892	0.446041
484	0.453659	0.935935	0.855556	0.433157
485	0.461789	0.941789	0.861789	0.454043
486	0.437398	0.945366	0.860705	0.441478
487	0.44878	0.93626	0.855014	0.429638
488	0.447154	0.942439	0.859892	0.442858

489	0.460163	0.94374	0.863144	0.457387
490	0.450407	0.941138	0.85935	0.442561
491	0.442276	0.938537	0.855827	0.429227
492	0.460163	0.941789	0.861518	0.452632
493	0.453659	0.939837	0.858808	0.442292
494	0.455285	0.938537	0.857995	0.440633
495	0.434146	0.937561	0.853659	0.419746
496	0.461789	0.943089	0.862873	0.457203
497	0.447154	0.939837	0.857724	0.436598
498	0.445528	0.938862	0.85664	0.432857
499	0.44065	0.94374	0.859892	0.440318
500	0.452033	0.943089	0.861247	0.448715

(6) Dataset S_6

Number of features	SN	SP	ACC	MCC
4	0.294309	0.976628	0.846512	0.40401
5	0.320325	0.975096	0.850233	0.424798
6	0.357724	0.971648	0.854574	0.449533
7	0.378862	0.970498	0.857674	0.465619
8	0.395122	0.96705	0.857984	0.470084
9	0.388618	0.964368	0.854574	0.456322
10	0.385366	0.965134	0.854574	0.455548
11	0.419512	0.964751	0.860775	0.485326
12	0.406504	0.963985	0.857674	0.4715
13	0.421138	0.964751	0.861085	0.48677
14	0.43252	0.962069	0.861085	0.489298
15	0.434146	0.961686	0.861085	0.489671
16	0.43252	0.962452	0.861395	0.49036
17	0.429268	0.962069	0.860465	0.486431
18	0.447154	0.96092	0.862946	0.498949
19	0.437398	0.959387	0.859845	0.486268
20	0.442276	0.959004	0.860465	0.489518
21	0.450407	0.960536	0.863256	0.500733
22	0.447154	0.958238	0.860775	0.491736
23	0.466667	0.957088	0.863566	0.505592
24	0.460163	0.958621	0.863566	0.504033
25	0.44065	0.955172	0.857054	0.477984
26	0.44878	0.958238	0.861085	0.493153
27	0.460163	0.955556	0.861085	0.496003
28	0.450407	0.957088	0.860465	0.491532
29	0.456911	0.956322	0.861085	0.495177
30	0.456911	0.957854	0.862326	0.499197

31	0.455285	0.955939	0.860465	0.492769
32	0.461789	0.956705	0.862326	0.500394
33	0.463415	0.955556	0.861705	0.498811
34	0.474797	0.954406	0.862946	0.505635
35	0.471545	0.954023	0.862016	0.501875
36	0.465041	0.954023	0.860775	0.496281
37	0.476423	0.956705	0.865116	0.512936
38	0.474797	0.954023	0.862636	0.50466
39	0.487805	0.955939	0.866667	0.52061
40	0.491057	0.95364	0.865426	0.517509
41	0.482927	0.950192	0.861085	0.502027
42	0.482927	0.953257	0.863566	0.509655
43	0.481301	0.954789	0.864496	0.512157
44	0.489431	0.951341	0.863256	0.510392
45	0.491057	0.954406	0.866047	0.519445
46	0.466667	0.95364	0.860775	0.496707
47	0.481301	0.955172	0.864806	0.513136
48	0.489431	0.95249	0.864186	0.51325
49	0.479675	0.955172	0.864496	0.511754
50	0.482927	0.952107	0.862636	0.506773
51	0.486179	0.954789	0.865426	0.516296
52	0.482927	0.955172	0.865116	0.514517
53	0.495935	0.95364	0.866357	0.521619
54	0.474797	0.952107	0.861085	0.499829
55	0.504065	0.954023	0.868217	0.529397
56	0.489431	0.952874	0.864496	0.514209
57	0.497561	0.951341	0.864806	0.517259
58	0.482927	0.954023	0.864186	0.511591
59	0.486179	0.954789	0.865426	0.516296
60	0.484553	0.950958	0.862016	0.505301
61	0.492683	0.952107	0.864496	0.515045
62	0.482927	0.950575	0.861395	0.50297
63	0.481301	0.951341	0.861705	0.50348
64	0.494309	0.954023	0.866357	0.521216
65	0.507317	0.95249	0.867597	0.528283
66	0.492683	0.950575	0.863256	0.511254
67	0.518699	0.945977	0.864496	0.522014
68	0.497561	0.950575	0.864186	0.515372
69	0.499187	0.949425	0.863566	0.513933
70	0.482927	0.954023	0.864186	0.511591
71	0.486179	0.947126	0.859225	0.497353
72	0.492683	0.952107	0.864496	0.515045
73	0.508943	0.945977	0.862636	0.513862

74	0.499187	0.947893	0.862326	0.510226
75	0.504065	0.950958	0.865736	0.521778
76	0.486179	0.950575	0.862016	0.505739
77	0.492683	0.94751	0.860775	0.503804
78	0.492683	0.948276	0.861395	0.50565
79	0.494309	0.948276	0.861705	0.507027
80	0.502439	0.949425	0.864186	0.516668
81	0.495935	0.948276	0.862016	0.508403
82	0.512195	0.948659	0.865426	0.522984
83	0.513821	0.949808	0.866667	0.527119
84	0.495935	0.949042	0.862636	0.510258
85	0.502439	0.95249	0.866667	0.524204
86	0.502439	0.950958	0.865426	0.520414
87	0.499187	0.948659	0.862946	0.512074
88	0.492683	0.947893	0.861085	0.504726
89	0.520325	0.944444	0.863566	0.519774
90	0.510569	0.949425	0.865736	0.523478
91	0.512195	0.945211	0.862636	0.514781
92	0.504065	0.950575	0.865426	0.520838
93	0.484553	0.949425	0.860775	0.501534
94	0.518699	0.949425	0.867287	0.530246
95	0.517073	0.949808	0.867287	0.529824
96	0.518699	0.946743	0.865116	0.523825
97	0.507317	0.947893	0.863876	0.517062
98	0.520325	0.948276	0.866667	0.528829
99	0.502439	0.948659	0.863566	0.514812
100	0.515447	0.948659	0.866047	0.525695
101	0.491057	0.949042	0.861705	0.506129
102	0.489431	0.948276	0.860775	0.502891
103	0.487805	0.949425	0.861395	0.504302
104	0.515447	0.942529	0.861085	0.511264
105	0.497561	0.951341	0.864806	0.517259
106	0.492683	0.945977	0.859535	0.500143
107	0.510569	0.949042	0.865426	0.522551
108	0.495935	0.947126	0.861085	0.50564
109	0.512195	0.945594	0.862946	0.515682
110	0.512195	0.95249	0.868527	0.532346
111	0.518699	0.948659	0.866667	0.528399
112	0.505691	0.945977	0.862016	0.511132
113	0.510569	0.944444	0.861705	0.511623
114	0.505691	0.950192	0.865426	0.521263
115	0.508943	0.94636	0.862946	0.51477
116	0.520325	0.944828	0.863876	0.520668

117	0.502439	0.945977	0.861395	0.508395
118	0.505691	0.948659	0.864186	0.517542
119	0.525203	0.943295	0.863566	0.521163
120	0.504065	0.944828	0.860775	0.507054
121	0.499187	0.94636	0.861085	0.506561
122	0.523577	0.94636	0.865736	0.526971
123	0.534959	0.944828	0.866667	0.532784
124	0.505691	0.947126	0.862946	0.513864
125	0.515447	0.945977	0.863876	0.519303
126	0.518699	0.943295	0.862326	0.515749
127	0.520325	0.945977	0.864806	0.523366
128	0.497561	0.943678	0.858605	0.498869
129	0.508943	0.949042	0.865116	0.521192
130	0.517073	0.945594	0.863876	0.519756
131	0.505691	0.946743	0.862636	0.512951
132	0.531707	0.944061	0.865426	0.528322
133	0.518699	0.949808	0.867597	0.531174
134	0.505691	0.947126	0.862946	0.513864
135	0.525203	0.946743	0.866357	0.529224
136	0.517073	0.942912	0.861705	0.513507
137	0.520325	0.948276	0.866667	0.528829
138	0.497561	0.945977	0.860465	0.504277
139	0.528455	0.946743	0.866977	0.531913
140	0.523577	0.944061	0.863876	0.521587
141	0.525203	0.947893	0.867287	0.531955
142	0.523577	0.944444	0.864186	0.522478
143	0.530081	0.945594	0.866357	0.530551
144	0.539837	0.94751	0.869767	0.543088
145	0.517073	0.943295	0.862016	0.514392
146	0.512195	0.940996	0.859225	0.505026
147	0.515447	0.94636	0.864186	0.520209
148	0.518699	0.946743	0.865116	0.523825
149	0.533333	0.938314	0.861085	0.51661
150	0.531707	0.943295	0.864806	0.526551
151	0.521951	0.943678	0.863256	0.519346
152	0.517073	0.94023	0.859535	0.507373
153	0.513821	0.941762	0.860155	0.508141
154	0.525203	0.941379	0.862016	0.516768
155	0.517073	0.945211	0.863566	0.518856
156	0.521951	0.943678	0.863256	0.519346
157	0.526829	0.945211	0.865426	0.526964
158	0.517073	0.945594	0.863876	0.519756
159	0.520325	0.941379	0.861085	0.512705

160	0.504065	0.941762	0.858295	0.499935
161	0.525203	0.944828	0.864806	0.524721
162	0.526829	0.942529	0.863256	0.520748
163	0.521951	0.944061	0.863566	0.520235
164	0.549593	0.944828	0.869457	0.544777
165	0.520325	0.941762	0.861395	0.51358
166	0.531707	0.942529	0.864186	0.524789
167	0.530081	0.941762	0.863256	0.52169
168	0.515447	0.945211	0.863256	0.517499
169	0.510569	0.946743	0.863566	0.517041
170	0.528455	0.944828	0.865426	0.527415
171	0.533333	0.944444	0.866047	0.530553
172	0.534959	0.940996	0.863566	0.523983
173	0.526829	0.942912	0.863566	0.521629
174	0.500813	0.942529	0.858295	0.498952
175	0.538211	0.94023	0.863566	0.524935
176	0.526829	0.943295	0.863876	0.522512
177	0.517073	0.942146	0.861085	0.511742
178	0.517073	0.941379	0.860465	0.509988
179	0.520325	0.942146	0.861705	0.514458
180	0.515447	0.942912	0.861395	0.512147
181	0.520325	0.941762	0.861395	0.51358
182	0.544715	0.945211	0.868837	0.541683
183	0.544715	0.939464	0.864186	0.528567
184	0.530081	0.938314	0.860465	0.513913
185	0.518699	0.939464	0.859225	0.507002
186	0.520325	0.939464	0.859535	0.508362
187	0.55122	0.939847	0.865736	0.534756
188	0.526829	0.941379	0.862326	0.518119
189	0.533333	0.941762	0.863876	0.524381
190	0.517073	0.93908	0.858605	0.504779
191	0.525203	0.939464	0.860465	0.51243
192	0.534959	0.940996	0.863566	0.523983
193	0.520325	0.943295	0.862636	0.517105
194	0.546341	0.93908	0.864186	0.529046
195	0.515447	0.944444	0.862636	0.515706
196	0.528455	0.938697	0.860465	0.513417
197	0.533333	0.940613	0.862946	0.521771
198	0.536585	0.937548	0.861085	0.5176
199	0.536585	0.941762	0.864496	0.527066
200	0.549593	0.940996	0.866357	0.536011
201	0.518699	0.940996	0.860465	0.510473
202	0.520325	0.937931	0.858295	0.504928

203	0.544715	0.937548	0.862636	0.524306
204	0.520325	0.943678	0.862946	0.517992
205	0.536585	0.942146	0.864806	0.52794
206	0.530081	0.940996	0.862636	0.519946
207	0.526829	0.94023	0.861395	0.515511
208	0.539837	0.938314	0.862326	0.521987
209	0.534959	0.94023	0.862946	0.52225
210	0.525203	0.940996	0.861705	0.515896
211	0.533333	0.937931	0.860775	0.515758
212	0.526829	0.944444	0.864806	0.525176
213	0.530081	0.937165	0.859535	0.51136
214	0.543089	0.939464	0.863876	0.52723
215	0.538211	0.941762	0.864806	0.528406
216	0.552846	0.945977	0.871008	0.550097
217	0.55122	0.935249	0.862016	0.524609
218	0.536585	0.936782	0.860465	0.515908
219	0.528455	0.940613	0.862016	0.517729
220	0.541463	0.938314	0.862636	0.523327
221	0.526829	0.941762	0.862636	0.518993
222	0.518699	0.939847	0.859535	0.507867
223	0.547967	0.940613	0.865736	0.533816
224	0.546341	0.94023	0.865116	0.531621
225	0.534959	0.939464	0.862326	0.520526
226	0.539837	0.93908	0.862946	0.523695
227	0.538211	0.93908	0.862636	0.522354
228	0.549593	0.938314	0.864186	0.530008
229	0.560976	0.937165	0.865426	0.536767
230	0.526829	0.939464	0.860775	0.513784
231	0.523577	0.937165	0.858295	0.505943
232	0.547967	0.937548	0.863256	0.526978
233	0.533333	0.938314	0.861085	0.51661
234	0.525203	0.939847	0.860775	0.513293
235	0.534959	0.936782	0.860155	0.514561
236	0.538211	0.9341	0.858605	0.511397
237	0.546341	0.937548	0.862946	0.525643
238	0.538211	0.938697	0.862326	0.521498
239	0.549593	0.935632	0.862016	0.524109
240	0.533333	0.939847	0.862326	0.520042
241	0.544715	0.941379	0.865736	0.532882
242	0.541463	0.937548	0.862016	0.521628
243	0.549593	0.939464	0.865116	0.532568
244	0.530081	0.935249	0.857984	0.507149
245	0.557724	0.936782	0.864496	0.533276

246	0.534959	0.93908	0.862016	0.519667
247	0.526829	0.9341	0.856434	0.501937
248	0.536585	0.934866	0.858915	0.511713
249	0.521951	0.935249	0.856434	0.500366
250	0.562602	0.933716	0.862946	0.530604
251	0.538211	0.94023	0.863566	0.524935
252	0.543089	0.937165	0.862016	0.522122
253	0.547967	0.936782	0.862636	0.52529
254	0.538211	0.935249	0.859535	0.513894
255	0.55122	0.934866	0.861705	0.523777
256	0.547967	0.937165	0.862946	0.526133
257	0.547967	0.935249	0.861395	0.521939
258	0.534959	0.934483	0.858295	0.509532
259	0.534959	0.936015	0.859535	0.512876
260	0.541463	0.932567	0.857984	0.510788
261	0.572358	0.939464	0.869457	0.551069
262	0.543089	0.936398	0.861395	0.520436
263	0.557724	0.933716	0.862016	0.526622
264	0.557724	0.9341	0.862326	0.527447
265	0.543089	0.939464	0.863876	0.52723
266	0.55122	0.939464	0.865426	0.533898
267	0.547967	0.934483	0.860775	0.520276
268	0.539837	0.938697	0.862636	0.52284
269	0.541463	0.9341	0.859225	0.514086
270	0.534959	0.935632	0.859225	0.512037
271	0.547967	0.936782	0.862636	0.52529
272	0.538211	0.931034	0.856124	0.504827
273	0.539837	0.934866	0.859535	0.514404
274	0.557724	0.938314	0.865736	0.536652
275	0.544715	0.938314	0.863256	0.526004
276	0.55122	0.937548	0.863876	0.529644
277	0.549593	0.937548	0.863566	0.528312
278	0.536585	0.939464	0.862636	0.52187
279	0.531707	0.938697	0.861085	0.516117
280	0.554472	0.937165	0.864186	0.531461
281	0.552846	0.94023	0.866357	0.536943
282	0.539837	0.935632	0.860155	0.516074
283	0.521951	0.929119	0.851473	0.487216
284	0.573984	0.930268	0.862326	0.53254
285	0.528455	0.936398	0.858605	0.508316
286	0.528455	0.934866	0.857364	0.504959
287	0.557724	0.936398	0.864186	0.532437
288	0.533333	0.94023	0.862636	0.520905

289	0.534959	0.939464	0.862326	0.520526
290	0.543089	0.938314	0.862946	0.524666
291	0.539837	0.937931	0.862016	0.521136
292	0.546341	0.937548	0.862946	0.525643
293	0.544715	0.931418	0.857674	0.511022
294	0.525203	0.935632	0.857364	0.503922
295	0.543089	0.931801	0.857674	0.510495
296	0.546341	0.938697	0.863876	0.528192
297	0.552846	0.939464	0.865736	0.535228
298	0.549593	0.936398	0.862636	0.525784
299	0.564228	0.931034	0.861085	0.526219
300	0.541463	0.936015	0.860775	0.518255
301	0.534959	0.939464	0.862326	0.520526
302	0.549593	0.9341	0.860775	0.520784
303	0.539837	0.936398	0.860775	0.517753
304	0.546341	0.936015	0.861705	0.522274
305	0.560976	0.93908	0.866977	0.541
306	0.544715	0.935632	0.861085	0.520098
307	0.556098	0.935249	0.862946	0.528604
308	0.531707	0.93295	0.856434	0.503518
309	0.570732	0.931034	0.862326	0.531512
310	0.547967	0.931801	0.858605	0.514519
311	0.531707	0.933716	0.857054	0.505171
312	0.546341	0.936015	0.861705	0.522274
313	0.55935	0.93295	0.861705	0.526308
314	0.536585	0.93295	0.857364	0.50757
315	0.562602	0.93295	0.862326	0.528962
316	0.55122	0.9341	0.861085	0.522119
317	0.538211	0.933333	0.857984	0.509742
318	0.556098	0.927586	0.856744	0.512358
319	0.546341	0.935249	0.861085	0.520602
320	0.549593	0.929119	0.856744	0.510198
321	0.55122	0.934483	0.861395	0.522947
322	0.549593	0.932184	0.859225	0.516673
323	0.538211	0.932567	0.857364	0.508096
324	0.538211	0.934866	0.859225	0.513059
325	0.547967	0.937931	0.863566	0.527825
326	0.538211	0.934866	0.859225	0.513059
327	0.556098	0.930268	0.858915	0.517956
328	0.55935	0.933333	0.862016	0.527128
329	0.531707	0.934483	0.857674	0.506831
330	0.541463	0.933716	0.858915	0.513258
331	0.556098	0.934483	0.862326	0.526944

332	0.55935	0.928352	0.857984	0.516614
333	0.547967	0.930268	0.857364	0.511272
334	0.536585	0.935632	0.859535	0.513384
335	0.534959	0.937165	0.860465	0.515407
336	0.569106	0.931418	0.862326	0.531
337	0.552846	0.931418	0.859225	0.517716
338	0.549593	0.933716	0.860465	0.519958
339	0.543089	0.934483	0.859845	0.516258
340	0.541463	0.933333	0.858605	0.512433
341	0.546341	0.931418	0.857984	0.512364
342	0.543089	0.9341	0.859535	0.515428
343	0.528455	0.927586	0.851473	0.489458
344	0.541463	0.929885	0.855814	0.505092
345	0.554472	0.933716	0.861395	0.523961
346	0.556098	0.935249	0.862946	0.528604
347	0.541463	0.931801	0.857364	0.50915
348	0.549593	0.932184	0.859225	0.516673
349	0.556098	0.934866	0.862636	0.527773
350	0.554472	0.928352	0.857054	0.512613
351	0.562602	0.931418	0.861085	0.525703
352	0.543089	0.93295	0.858605	0.512952
353	0.546341	0.932567	0.858915	0.514814
354	0.543089	0.930651	0.856744	0.508055
355	0.536585	0.941762	0.864496	0.527066
356	0.572358	0.93295	0.864186	0.536893
357	0.56748	0.931034	0.861705	0.528868
358	0.554472	0.932567	0.860465	0.521497
359	0.547967	0.9341	0.860465	0.519447
360	0.544715	0.933716	0.859535	0.515942
361	0.546341	0.935249	0.861085	0.520602
362	0.544715	0.930651	0.857054	0.509398
363	0.520325	0.931801	0.853333	0.491541
364	0.556098	0.93295	0.861085	0.523648
365	0.534959	0.9341	0.857984	0.508701
366	0.544715	0.934866	0.860465	0.51843
367	0.534959	0.931034	0.855504	0.502128
368	0.538211	0.936782	0.860775	0.517252
369	0.547967	0.928736	0.856124	0.508057
370	0.552846	0.929502	0.857674	0.513677
371	0.560976	0.925287	0.855814	0.511635
372	0.554472	0.936782	0.863876	0.53062
373	0.539837	0.932567	0.857674	0.509442
374	0.549593	0.933716	0.860465	0.519958

375	0.538211	0.926437	0.852403	0.495209
376	0.552846	0.932567	0.860155	0.520163
377	0.554472	0.930268	0.858605	0.516622
378	0.543089	0.929885	0.856124	0.506438
379	0.552846	0.932184	0.859845	0.519345
380	0.560976	0.933333	0.862326	0.528456
381	0.564228	0.92682	0.857674	0.517436
382	0.572358	0.93295	0.864186	0.536893
383	0.536585	0.931801	0.856434	0.505109
384	0.560976	0.930651	0.860155	0.522756
385	0.556098	0.934866	0.862636	0.527773
386	0.554472	0.933333	0.861085	0.523137
387	0.521951	0.9341	0.855504	0.49786
388	0.554472	0.94023	0.866667	0.538271
389	0.533333	0.929885	0.854264	0.498343
390	0.554472	0.931418	0.859535	0.519051
391	0.539837	0.930651	0.856124	0.505363
392	0.543089	0.936015	0.861085	0.519596
393	0.55122	0.931801	0.859225	0.517194
394	0.55122	0.934866	0.861705	0.523777
395	0.533333	0.932184	0.856124	0.503226
396	0.528455	0.936398	0.858605	0.508316
397	0.556098	0.931418	0.859845	0.520384
398	0.541463	0.9341	0.859225	0.514086
399	0.544715	0.931034	0.857364	0.510209
400	0.562602	0.932567	0.862016	0.528144
401	0.533333	0.936782	0.859845	0.513213
402	0.557724	0.931418	0.860155	0.521716
403	0.541463	0.930268	0.856124	0.5059
404	0.547967	0.934866	0.861085	0.521106
405	0.552846	0.935249	0.862326	0.525942
406	0.554472	0.928352	0.857054	0.512613
407	0.552846	0.937165	0.863876	0.530131
408	0.55935	0.934866	0.863256	0.53043
409	0.549593	0.932567	0.859535	0.517491
410	0.541463	0.933716	0.858915	0.513258
411	0.544715	0.932567	0.858605	0.513473
412	0.526829	0.933333	0.855814	0.500279
413	0.56748	0.928736	0.859845	0.524053
414	0.554472	0.932184	0.860155	0.520679
415	0.543089	0.930651	0.856744	0.508055
416	0.546341	0.934483	0.860465	0.518938
417	0.556098	0.929885	0.858605	0.517151

418	0.547967	0.92682	0.854574	0.50408
419	0.554472	0.930268	0.858605	0.516622
420	0.536585	0.932567	0.857054	0.506748
421	0.533333	0.933333	0.857054	0.505695
422	0.539837	0.934483	0.859225	0.513572
423	0.538211	0.936398	0.860465	0.516409
424	0.533333	0.931418	0.855504	0.501591
425	0.530081	0.929885	0.853643	0.495633
426	0.530081	0.933716	0.856744	0.503818
427	0.556098	0.931801	0.860155	0.521197
428	0.560976	0.924521	0.855194	0.510075
429	0.546341	0.935249	0.861085	0.520602
430	0.536585	0.935632	0.859535	0.513384
431	0.55935	0.928352	0.857984	0.516614
432	0.552846	0.927969	0.856434	0.510479
433	0.534959	0.932567	0.856744	0.505398
434	0.549593	0.93295	0.859845	0.518312
435	0.547967	0.933716	0.860155	0.518621
436	0.55122	0.929885	0.857674	0.513144
437	0.549593	0.927203	0.855194	0.506213
438	0.549593	0.928736	0.856434	0.509397
439	0.547967	0.936398	0.862326	0.524449
440	0.557724	0.928736	0.857984	0.516079
441	0.554472	0.931034	0.859225	0.518239
442	0.55935	0.936782	0.864806	0.534602
443	0.530081	0.931418	0.854884	0.498883
444	0.549593	0.930651	0.857984	0.51342
445	0.541463	0.93295	0.858295	0.511609
446	0.544715	0.9341	0.859845	0.51677
447	0.521951	0.935249	0.856434	0.500366
448	0.544715	0.9341	0.859845	0.51677
449	0.557724	0.931418	0.860155	0.521716
450	0.556098	0.934483	0.862326	0.526944
451	0.547967	0.929119	0.856434	0.508858
452	0.544715	0.929885	0.856434	0.507782
453	0.536585	0.934483	0.858605	0.51088
454	0.531707	0.928736	0.853023	0.494573
455	0.543089	0.931418	0.857364	0.509679
456	0.531707	0.936398	0.859225	0.51102
457	0.557724	0.934866	0.862946	0.529102
458	0.556098	0.93295	0.861085	0.523648
459	0.541463	0.930651	0.856434	0.50671
460	0.534959	0.936782	0.860155	0.514561

461	0.538211	0.934483	0.858915	0.512227
462	0.543089	0.934483	0.859845	0.516258
463	0.533333	0.927969	0.852713	0.494327
464	0.569106	0.929502	0.860775	0.526974
465	0.556098	0.934866	0.862636	0.527773
466	0.541463	0.932567	0.857984	0.510788
467	0.569106	0.935632	0.865736	0.540026
468	0.557724	0.933716	0.862016	0.526622
469	0.549593	0.932567	0.859535	0.517491
470	0.546341	0.927969	0.855194	0.505118
471	0.539837	0.932184	0.857364	0.508623
472	0.556098	0.931034	0.859535	0.519573
473	0.531707	0.931418	0.855194	0.500237
474	0.541463	0.932184	0.857674	0.509968
475	0.554472	0.930268	0.858605	0.516622
476	0.546341	0.9341	0.860155	0.518109
477	0.55122	0.930268	0.857984	0.51395
478	0.554472	0.932567	0.860465	0.521497
479	0.546341	0.932567	0.858915	0.514814
480	0.55935	0.935632	0.863876	0.532093
481	0.55122	0.934483	0.861395	0.522947
482	0.520325	0.937165	0.857674	0.503225
483	0.554472	0.928736	0.857364	0.513411
484	0.560976	0.929119	0.858915	0.519541
485	0.549593	0.929502	0.857054	0.511001
486	0.547967	0.930268	0.857364	0.511272
487	0.531707	0.931418	0.855194	0.500237
488	0.541463	0.931801	0.857364	0.50915
489	0.530081	0.931034	0.854574	0.498067
490	0.55122	0.934483	0.861395	0.522947
491	0.549593	0.934483	0.861085	0.521612
492	0.556098	0.931801	0.860155	0.521197
493	0.530081	0.928352	0.852403	0.492415
494	0.526829	0.92682	0.850543	0.486508
495	0.55122	0.930268	0.857984	0.51395
496	0.552846	0.929119	0.857364	0.512875
497	0.560976	0.924904	0.855504	0.510854
498	0.531707	0.931034	0.854884	0.499422
499	0.564228	0.934866	0.864186	0.534405
500	0.547967	0.931418	0.858295	0.513704
