# Supplementary material for: Analysis of Tumor Suppressor Genes Based on Gene Ontology and the KEGG Pathway
Source: PLoS One. 2014 Sep 10;9(9):e107202. doi: 10.1371/journal.pone.0107202 (PMC4160198; doi:10.1371/journal.pone.0107202)
Supplement: Table S4 — List of 717 Features in the final optimal feature set. (PDF) [file pone.0107202.s004.pdf]

Table S4. 717 Features in the final optimal feature set

(1) 708 Features of GO terms

GO:0000093	GO:0000115	GO:0000188	GO:0000239
GO:0000320	GO:0000422	GO:0000733	GO:0000739
GO:0000783	GO:0000785	GO:0000791	GO:0000792
GO:0000819	GO:0000904	GO:0000979	GO:0001032
GO:0001047	GO:0001502	GO:0001503	GO:0001570
GO:0001656	GO:0001661	GO:0001707	GO:0001726
GO:0001776	GO:0001825	GO:0001829	GO:0001835
GO:0001836	GO:0001841	GO:0001843	GO:0001886
GO:0001889	GO:0001893	GO:0001952	GO:0001953
GO:0001957	GO:0002009	GO:0002042	GO:0002064
GO:0002070	GO:0002089	GO:0002309	GO:0002352
GO:0002762	GO:0002902	GO:0003136	GO:0003140
GO:0003156	GO:0003214	GO:0003382	GO:0003712
GO:0003714	GO:0003886	GO:0003908	GO:0004415
GO:0004438	GO:0004515	GO:0004702	GO:0004712
GO:0004716	GO:0004721	GO:0004727	GO:0004861
GO:0004882	GO:0005006	GO:0005019	GO:0005068
GO:0005072	GO:0005099	GO:0005100	GO:0005113
GO:0005501	GO:0005678	GO:0005712	GO:0005737
GO:0005741	GO:0005884	GO:0005912	GO:0005913
GO:0006097	GO:0006266	GO:0006306	GO:0006307
GO:0006309	GO:0006346	GO:0006349	GO:0006407
GO:0006417	GO:0006464	GO:0006469	GO:0006473
GO:0006513	GO:0006627	GO:0006776	GO:0006914
GO:0006921	GO:0006930	GO:0006978	GO:0006983
GO:0007050	GO:0007060	GO:0007090	GO:0007092
GO:0007098	GO:0007140	GO:0007158	GO:0007162
GO:0007182	GO:0007185	GO:0007265	GO:0007270
GO:0007281	GO:0007346	GO:0007356	GO:0007398
GO:0007403	GO:0007406	GO:0007416	GO:0007417
GO:0007422	GO:0007435	GO:0007497	GO:0007530
GO:0007569	GO:0007595	GO:0007634	GO:0008054
GO:0008064	GO:0008138	GO:0008191	GO:0008209
GO:0008270	GO:0008327	GO:0008330	GO:0008340
GO:0008385	GO:0008426	GO:0008432	GO:0008544
GO:0008585	GO:0008595	GO:0008629	GO:0008630
GO:0008634	GO:0009008	GO:0009048	GO:0009103
GO:0009132	GO:0009410	GO:0009411	GO:0009649
GO:0009792	GO:0009826	GO:0009890	GO:0009948
GO:0009950	GO:0009954	GO:0009987	GO:0010165
GO:0010243	GO:0010389	GO:0010424	GO:0010452

GO:0010464	GO:0010506	GO:0010522	GO:0010564
GO:0010637	GO:0010717	GO:0010719	GO:0010761
GO:0010801	GO:0010839	GO:0010907	GO:0010942
GO:0010957	GO:0010975	GO:0010997	GO:0014003
GO:0014010	GO:0014034	GO:0014037	GO:0015014
GO:0015267	GO:0015938	GO:0015964	GO:0016151
GO:0016235	GO:0016310	GO:0016314	GO:0016328
GO:0016339	GO:0016342	GO:0016514	GO:0016538
GO:0016571	GO:0016601	GO:0016605	GO:0016918
GO:0017015	GO:0017017	GO:0017053	GO:0017124
GO:0017148	GO:0018205	GO:0019002	GO:0019207
GO:0019208	GO:0019210	GO:0019211	GO:0019538
GO:0019789	GO:0019841	GO:0019898	GO:0019941
GO:0019985	GO:0021542	GO:0021546	GO:0021569
GO:0021571	GO:0021599	GO:0021602	GO:0021612
GO:0021615	GO:0021754	GO:0021772	GO:0021779
GO:0021782	GO:0021877	GO:0021889	GO:0021895
GO:0021897	GO:0021955	GO:0022029	GO:0022405
GO:0022601	GO:0022612	GO:0030030	GO:0030100
GO:0030111	GO:0030178	GO:0030215	GO:0030224
GO:0030235	GO:0030264	GO:0030284	GO:0030296
GO:0030325	GO:0030332	GO:0030336	GO:0030414
GO:0030506	GO:0030511	GO:0030520	GO:0030521
GO:0030539	GO:0030578	GO:0030675	GO:0030695
GO:0030704	GO:0030833	GO:0030850	GO:0030852
GO:0030857	GO:0030858	GO:0030891	GO:0031065
GO:0031134	GO:0031226	GO:0031235	GO:0031307
GO:0031398	GO:0031401	GO:0031461	GO:0031462
GO:0031571	GO:0031575	GO:0031619	GO:0031647
GO:0031648	GO:0031658	GO:0031665	GO:0031668
GO:0031670	GO:0031931	GO:0031999	GO:0032007
GO:0032025	GO:0032090	GO:0032137	GO:0032204
GO:0032211	GO:0032228	GO:0032286	GO:0032300
GO:0032318	GO:0032321	GO:0032389	GO:0032390
GO:0032407	GO:0032435	GO:0032461	GO:0032515
GO:0032516	GO:0032535	GO:0032680	GO:0032794
GO:0032835	GO:0032862	GO:0032869	GO:0032870
GO:0032872	GO:0032880	GO:0032925	GO:0032930
GO:0032938	GO:0032956	GO:0033032	GO:0033088
GO:0033146	GO:0033235	GO:0033326	GO:0033327
GO:0033561	GO:0033593	GO:0033596	GO:0033598
GO:0033601	GO:0033631	GO:0033673	GO:0033687
GO:0033688	GO:0033692	GO:0034088	GO:0034124
GO:0034244	GO:0034332	GO:0034333	GO:0034613

GO:0034614	GO:0034644	GO:0034742	GO:0034983
GO:0035021	GO:0035022	GO:0035024	GO:0035033
GO:0035037	GO:0035088	GO:0035117	GO:0035189
GO:0035264	GO:0035265	GO:0035305	GO:0035329
GO:0035330	GO:0035375	GO:0035414	GO:0035441
GO:0035583	GO:0035623	GO:0035694	GO:0035726
GO:0035730	GO:0035731	GO:0035732	GO:0035767
GO:0035791	GO:0035802	GO:0035907	GO:0035970
GO:0035988	GO:0036023	GO:0036120	GO:0038085
GO:0038091	GO:0040008	GO:0042058	GO:0042069
GO:0042117	GO:0042129	GO:0042271	GO:0042326
GO:0042328	GO:0042406	GO:0042473	GO:0042512
GO:0042518	GO:0042524	GO:0042551	GO:0042552
GO:0042663	GO:0042711	GO:0042766	GO:0042771
GO:0042992	GO:0042993	GO:0043005	GO:0043015
GO:0043028	GO:0043045	GO:0043060	GO:0043152
GO:0043220	GO:0043280	GO:0043281	GO:0043295
GO:0043296	GO:0043366	GO:0043374	GO:0043409
GO:0043433	GO:0043495	GO:0043497	GO:0043508
GO:0043517	GO:0043522	GO:0043523	GO:0043535
GO:0043542	GO:0043550	GO:0043570	GO:0043616
GO:0043652	GO:0043653	GO:0043666	GO:0043923
GO:0044319	GO:0044342	GO:0044344	GO:0044345
GO:0044430	GO:0044444	GO:0045090	GO:0045184
GO:0045295	GO:0045296	GO:0045444	GO:0045475
GO:0045545	GO:0045569	GO:0045578	GO:0045579
GO:0045604	GO:0045606	GO:0045621	GO:0045647
GO:0045656	GO:0045667	GO:0045685	GO:0045726
GO:0045732	GO:0045736	GO:0045750	GO:0045765
GO:0045792	GO:0045793	GO:0045814	GO:0045837
GO:0045842	GO:0045859	GO:0045879	GO:0045880
GO:0045884	GO:0045899	GO:0045930	GO:0045950
GO:0046022	GO:0046329	GO:0046356	GO:0046426
GO:0046546	GO:0046580	GO:0046621	GO:0046718
GO:0046790	GO:0046825	GO:0046851	GO:0046882
GO:0046902	GO:0046930	GO:0046985	GO:0047485
GO:0047710	GO:0048014	GO:0048048	GO:0048102
GO:0048145	GO:0048147	GO:0048320	GO:0048545
GO:0048546	GO:0048562	GO:0048597	GO:0048608
GO:0048619	GO:0048702	GO:0048712	GO:0048714
GO:0048733	GO:0048742	GO:0048745	GO:0048807
GO:0048844	GO:0048846	GO:0048853	GO:0048859
GO:0050431	GO:0050508	GO:0050509	GO:0050678
GO:0050680	GO:0050808	GO:0050821	GO:0050847

GO:0050856	GO:0050878	GO:0050910	GO:0050920
GO:0051017	GO:0051020	GO:0051048	GO:0051057
GO:0051097	GO:0051102	GO:0051146	GO:0051153
GO:0051171	GO:0051205	GO:0051257	GO:0051262
GO:0051271	GO:0051276	GO:0051365	GO:0051385
GO:0051400	GO:0051412	GO:0051443	GO:0051444
GO:0051492	GO:0051497	GO:0051595	GO:0051606
GO:0051668	GO:0051717	GO:0051721	GO:0051726
GO:0051782	GO:0051797	GO:0051865	GO:0051893
GO:0051894	GO:0051895	GO:0051898	GO:0051902
GO:0051973	GO:0055096	GO:0055100	GO:0055105
GO:0055106	GO:0060011	GO:0060023	GO:0060024
GO:0060056	GO:0060070	GO:0060074	GO:0060135
GO:0060179	GO:0060197	GO:0060206	GO:0060216
GO:0060324	GO:0060340	GO:0060346	GO:0060347
GO:0060411	GO:0060421	GO:0060429	GO:0060440
GO:0060465	GO:0060480	GO:0060512	GO:0060546
GO:0060547	GO:0060571	GO:0060575	GO:0060599
GO:0060662	GO:0060706	GO:0060709	GO:0060711
GO:0060716	GO:0060749	GO:0060769	GO:0060770
GO:0060789	GO:0060876	GO:0060923	GO:0060996
GO:0060997	GO:0061002	GO:0061031	GO:0061032
GO:0061198	GO:0061364	GO:0061428	GO:0065004
GO:0070026	GO:0070052	GO:0070059	GO:0070141
GO:0070215	GO:0070245	GO:0070318	GO:0070372
GO:0070435	GO:0070483	GO:0070507	GO:0070534
GO:0070557	GO:0070664	GO:0070830	GO:0070936
GO:0071157	GO:0071158	GO:0071168	GO:0071228
GO:0071279	GO:0071305	GO:0071364	GO:0071385
GO:0071391	GO:0071398	GO:0071456	GO:0071479
GO:0071481	GO:0071504	GO:0071559	GO:0071564
GO:0071565	GO:0071679	GO:0071681	GO:0071850
GO:0071889	GO:0071901	GO:0071930	GO:0072015
GO:0072112	GO:0072133	GO:0072166	GO:0072284
GO:0072302	GO:0072332	GO:0072384	GO:0072498
GO:0090051	GO:0090071	GO:0090096	GO:0090116
GO:0090136	GO:0090141	GO:0090175	GO:0090179
GO:0090191	GO:0090200	GO:0090219	GO:0090246
GO:0090343	GO:0090394	GO:0090398	GO:0090403
GO:0097057	GO:0097105	GO:0097107	GO:0097119
GO:0097162	GO:0097190	GO:0097194	GO:2000008
GO:2000017	GO:2000041	GO:2000045	GO:2000052
GO:2000054	GO:2000080	GO:2000117	GO:2000119
GO:2000134	GO:2000195	GO:2000271	GO:2000273

GO:2000304	GO:2000310	GO:2000342	GO:2000378
GO:2000379	GO:2000463	GO:2000484	GO:2000500
GO:2000808	GO:2000836	GO:2001020	GO:2001022
GO:2001047	GO:2001076	GO:2001241	GO:2001244

(2) 9 Features of KEGG pathways

hsa00100	hsa04115	hsa05213	hsa05216
hsa05218	hsa05219	hsa05220	hsa05221
hsa05223			
