# Supplementary material for: Analysis of Tumor Suppressor Genes Based on Gene Ontology and the KEGG Pathway
Source: PLoS One. 2014 Sep 10;9(9):e107202. doi: 10.1371/journal.pone.0107202 (PMC4160198; doi:10.1371/journal.pone.0107202)
Supplement: Table S5 — List of the novel tumor suppressors predicted based on features in the total optimal feature set. (PDF) [file pone.0107202.s005.pdf]

Table S5. The novel tumor suppressors predicted based on features in the total optimal feature set. 'Number of key tumor suppressor functions' is defined as the number of features in the total optimal feature set whose values are greater than $-\log_{10}(0.05)$, *i.e.* , enrichment p value is smaller than 0.05.

Ensembl ID	Number of key tumor suppressor functions	Gene symbol
ENSP00000297261	353	SHH
ENSP00000324806	353	GSK3B
ENSP00000389184	345	MARK2
ENSP00000264657	338	STAT3
ENSP00000355069	338	PAX2
ENSP00000293549	337	WNT1
ENSP00000353483	331	MAPK8
ENSP00000263253	331	EP300
ENSP00000218894	327	SUPT20H
ENSP00000328181	327	NOG
ENSP00000228872	327	CDKN1B
ENSP00000338548	325	FGF1
ENSP00000250003	322	MYOD1
ENSP00000206249	322	ESR1
ENSP00000245451	321	BMP4
ENSP00000352514	317	RUNX2
ENSP00000348986	316	INS-IGF2
ENSP00000263025	315	MAPK3
ENSP00000354558	313	MTOR
ENSP00000363822	311	AR
ENSP00000361066	310	NCOA3
ENSP00000339004	309	FOXG1
ENSP00000320604	309	FAXDC2
ENSP00000338018	308	HIF1A
ENSP00000278385	308	CD44
ENSP00000216797	306	NFKBIA
ENSP00000222330	304	GSK3A
ENSP00000255465	304	CCNA1
ENSP00000222726	303	HOXA5
ENSP00000334458	303	GATA4
ENSP00000264498	303	FGF2
ENSP00000323588	302	SOX2
ENSP00000392858	299	TNF
ENSP00000302665	299	IGF1
ENSP00000338297	298	-
ENSP00000362649	297	HDAC1
ENSP00000318977	297	GEN1
ENSP00000343745	296	DICER1
ENSP00000265165	294	LEF1
ENSP00000415481	293	PROM1

ENSP00000321797	293	FGF8
ENSP00000284523	292	WNT3A
ENSP00000265171	292	EGF
ENSP00000274026	292	CCNA2
ENSP00000301633	291	BIRC5
ENSP00000020945	290	SNAI2
ENSP00000302486	290	MAP2K1
ENSP00000379204	290	BMP7
ENSP00000215832	289	MAPK1
ENSP00000360025	288	GADD45A
ENSP00000361125	287	VEGFA
ENSP00000345206	287	RBPJ
ENSP00000268035	287	IGF1R
ENSP00000370938	287	CDK8
ENSP00000346294	286	S100A4
ENSP00000368438	286	PCNA
ENSP00000229794	286	MAPK14
ENSP00000298902	286	IFI27
ENSP00000319248	285	ZEB1
ENSP00000262158	285	SMAD7
ENSP00000231509	285	NR3C1
ENSP00000372170	285	MSX1
ENSP00000332164	283	SLIT3
ENSP00000269141	283	CDH2
ENSP00000337451	282	EPHA3
ENSP00000310036	282	CD34
ENSP00000329357	281	SP1
ENSP00000312987	281	HNF4A
ENSP00000320147	281	EZH2
ENSP00000311032	281	CASP3
ENSP00000315112	280	RNF31
ENSP00000348461	280	RAC1
ENSP00000305769	278	SMAD1
ENSP00000408617	278	HDAC9
ENSP00000346879	277	NKX2-1
ENSP00000258743	277	IL6
ENSP00000229135	277	IFNG
ENSP00000321410	276	MAPK9
ENSP00000295731	276	IHH
ENSP00000234091	276	ID2
ENSP00000225893	276	HNF1B
ENSP00000245479	275	SOX9
ENSP00000364265	275	FOXE1
ENSP00000290167	274	WNT4
ENSP00000241261	274	TNFSF10
ENSP00000354394	272	STAT1

ENSP00000361405	272	MMP9
ENSP00000266646	272	INHBE
ENSP00000358525	271	NGF
ENSP00000228280	271	KITLG
ENSP00000232424	271	HES1
ENSP00000363591	271	BAK1
ENSP00000303830	270	INSR
ENSP00000282397	270	FLT1
ENSP00000027335	270	CDH17
ENSP00000355896	269	TGFB2
ENSP00000294008	269	SLX4
ENSP00000419194	269	MSMP
ENSP00000302564	269	BCL2L1
ENSP00000324897	268	UBE2I
ENSP00000348551	268	NCOR2
ENSP00000305422	268	CEBPB
ENSP00000257863	268	AMHR2
ENSP00000244050	267	SNAI1
ENSP00000356694	267	FASLG
ENSP00000301727	267	E4F1
ENSP00000349017	266	ABCC11
ENSP00000262120	265	TWSG1
ENSP00000328928	265	HOXB4
ENSP00000280193	263	VEGFC
ENSP00000302111	263	MGMT
ENSP00000317145	263	GDNF
ENSP00000297268	263	COL1A2
ENSP00000239849	262	TNFSF11
ENSP00000402515	262	SMG1
ENSP00000352121	262	PIK3CG
ENSP00000368632	261	GATA3
ENSP00000011653	261	CD4
ENSP00000224764	260	-
ENSP00000346508	259	PDGFA
ENSP00000254958	259	JAG1
ENSP00000222390	259	HGF
ENSP00000263551	259	-
ENSP00000339007	258	GRB2
ENSP00000346839	258	FN1
ENSP00000347169	257	NUMB
ENSP00000310263	256	TNFRSF10D
ENSP00000356480	256	RNF2
ENSP00000241651	256	MYOG
ENSP00000368169	256	DVL1
ENSP00000351901	256	CARD8
ENSP00000262765	255	QRICH2

ENSP00000341189	255	PTK2
ENSP00000382423	255	MAP3K1
ENSP00000252242	255	KRT5
ENSP00000219454	254	WFDC1
ENSP00000242261	254	TWIST1
ENSP00000325120	253	PGR
ENSP00000314709	252	ZHX2
ENSP00000362690	252	NR5A1
ENSP00000340330	252	KAT5
ENSP00000204961	252	EFNB1
ENSP00000262643	252	CCNE1
ENSP00000309572	251	TERT
ENSP00000299293	251	FRS2
ENSP00000266970	251	CDK2
ENSP00000343785	250	SPRY1
ENSP00000244745	250	SOX4
ENSP00000401303	250	SHC1
ENSP00000410715	250	SFRP4
ENSP00000274335	250	PIK3R1
ENSP00000342392	250	MESP2
ENSP00000339151	250	IKBKB
ENSP00000302501	249	ZEB2
ENSP00000363571	249	MUSK
ENSP00000283635	249	CD8A
ENSP00000295349	249	-
ENSP00000240328	248	TBX2
ENSP00000229307	248	NANOG
ENSP00000358022	248	MCL1
ENSP00000419692	247	RXRA
ENSP00000351490	247	MAX
ENSP00000309103	247	BAD
ENSP00000252486	247	APOE
ENSP00000278968	246	TAGLN
ENSP00000249636	246	PIAS1
ENSP00000239243	246	MSX2
ENSP00000338272	246	HEY1
ENSP00000254122	246	FSHB
ENSP00000300145	245	XRCC6BP1
ENSP00000212015	245	SIRT1
ENSP00000327758	245	NKX2-5
ENSP00000341835	245	MYOCD
ENSP00000219070	245	MMP2
ENSP00000328169	245	JAG2
ENSP00000309503	244	YWHAZ
ENSP00000388107	244	UBA52
ENSP00000363089	244	TLR4

ENSP00000295400	244	TGFA
ENSP00000386200	244	FOXP2
ENSP00000368686	244	E2F4
ENSP00000339845	244	DROSHA
ENSP00000367830	243	PRKCZ
ENSP00000264867	243	PPARGC1A
ENSP00000297494	243	NOS3
ENSP00000326371	243	FOXC2
ENSP00000352516	243	DNMT1
ENSP00000264110	243	ATF2
ENSP00000224237	242	VIM
ENSP00000328547	242	DNMT3B
ENSP00000359206	242	BTRC
ENSP00000344818	241	UBC
ENSP00000200453	241	PPP1R15A
ENSP00000289153	241	PIK3CB
ENSP00000284981	241	APP
ENSP00000298700	241	-
ENSP00000307356	241	-
ENSP00000306682	240	PPM1D
ENSP00000268712	240	NCOR1
ENSP00000344352	240	ATF3
ENSP00000316840	240	-
ENSP00000304895	239	IRS1
ENSP00000381607	239	GSTP1
ENSP00000340347	238	TCF7
ENSP00000356438	238	PTGS2
ENSP00000261349	238	LRP6
ENSP00000347979	238	FAS
ENSP00000242057	238	AHR
ENSP00000363998	237	ITCH
ENSP00000262904	237	E2F3
ENSP00000302812	236	SP7
ENSP00000268459	236	NKD1
ENSP00000327850	236	NFATC1
ENSP00000369554	236	IFNA2
ENSP00000357429	236	FABP7
ENSP00000340691	236	EIF4EBP1
ENSP00000264709	236	DNMT3A
ENSP00000257963	236	ACVR1B
ENSP00000234420	235	MSH6
ENSP00000314458	235	CDC42
ENSP00000303706	235	CDC25A
ENSP00000384803	235	-
ENSP00000343535	234	USP7
ENSP00000383089	234	TPTE2

ENSP00000195419	234	-
ENSP00000411532	233	TOP2A
ENSP00000274376	233	RASA1
ENSP00000217185	233	PTK6
ENSP00000311005	233	MAPK7
ENSP00000262426	233	FOXF1
ENSP00000263734	233	EPAS1
ENSP00000295743	233	EOMES
ENSP00000358903	233	CYP17A1
ENSP00000356623	233	CITED2
ENSP00000379255	233	-
ENSP00000232219	232	RBP1
ENSP00000252809	232	GDF15
ENSP00000350311	232	-
ENSP00000304169	231	PITX2
ENSP00000348634	231	MYH6
ENSP00000225983	231	HDAC5
ENSP00000287934	231	FZD1
ENSP00000342307	231	FOXM1
ENSP00000385705	230	TOX3
ENSP00000167586	230	KRT14
ENSP00000220592	230	AGO2
ENSP00000263583	230	-
ENSP00000274255	229	SKP2
ENSP00000228307	229	PXN
ENSP00000360683	229	PTPN1
ENSP00000263754	229	KAT2B
ENSP00000274625	229	FGF18
ENSP00000368238	229	-
ENSP00000369972	229	-
ENSP00000370857	229	-
ENSP00000390675	229	-
ENSP00000411229	229	-
ENSP00000281537	228	TJP1
ENSP00000326366	228	PSEN1
ENSP00000327255	228	PPM1A
ENSP00000359212	228	LBX1
ENSP00000261937	228	FLT4
ENSP00000386884	228	CXCR4
ENSP00000256442	228	CCNB1
ENSP00000265354	227	SRF
ENSP00000313420	227	PRKDC
ENSP00000355304	227	MOK
ENSP00000264664	227	FGF10
ENSP00000332118	227	EPHB3
ENSP00000357255	227	BGLAP

ENSP00000376076	226	SUMO1
ENSP00000265563	226	PRKAR2A
ENSP00000312652	226	LEP
ENSP00000283921	226	HOXA10
ENSP00000253799	226	AOC2
ENSP00000349960	226	ACTB
ENSP00000353864	225	PAK3
ENSP00000368759	225	NEDD9
ENSP00000215631	225	GADD45B
ENSP00000005340	225	DVL2
ENSP00000336790	225	ATF4
ENSP00000344220	224	PDPK1
ENSP00000222598	224	DLX5
ENSP00000392762	224	DCT
ENSP00000321656	224	CDC25C
ENSP00000314132	224	BOK
ENSP00000241416	224	ACVR2A
ENSP00000356999	223	USF1
ENSP00000280612	223	SLC7A11
ENSP00000416293	223	SLC2A1
ENSP00000344173	223	GRM8
ENSP00000250448	223	FOXA1
ENSP00000310170	223	FOSL1
ENSP00000316054	223	DVL3
ENSP00000314897	223	ANGPT2
ENSP00000265164	222	CASP6
ENSP00000262187	221	RHEB
ENSP00000282091	221	PTH
ENSP00000366563	221	PIK3CD
ENSP00000263341	221	IL1B
ENSP00000295113	221	FRZB
ENSP00000335376	221	-
ENSP00000341032	220	WNT7B
ENSP00000364133	220	TGFBR1
ENSP00000238682	220	TGFB3
ENSP00000307046	220	SDC2
ENSP00000257555	220	HNF1A
ENSP00000264606	220	HDAC4
ENSP00000256759	220	FST
ENSP00000309181	220	CCNE2
ENSP00000288207	220	CCNB2
ENSP00000414303	220	BDNF
ENSP00000355583	220	-
ENSP00000360215	219	ZBP1
ENSP00000229022	219	VDR
ENSP00000314946	219	RPRM

ENSP00000394794	219	PTPN13
ENSP00000351049	219	PAK4
ENSP00000403447	219	NOTCH4
ENSP00000384625	219	NLK
ENSP00000396219	219	MEF2C
ENSP00000359573	219	LMO4
ENSP00000323901	219	FZD2
ENSP00000364895	218	ZBTB17
ENSP00000381045	218	UBE3A
ENSP00000255612	218	PRAM1
ENSP00000262971	218	PIAS4
ENSP00000330875	218	FANCF
ENSP00000355718	218	DLL1
ENSP00000268053	218	CYP11A1
ENSP00000265922	218	BRINP1
ENSP00000265441	217	WNT2
ENSP00000301061	217	WNT10B
ENSP00000171887	217	TNS1
ENSP00000323050	217	RBBP8
ENSP00000300177	217	GREM1
ENSP00000252723	217	EPO
ENSP00000398124	217	E2F5
ENSP00000369050	217	CYP1A1
ENSP00000363548	217	CXCL12
ENSP00000283147	217	BMP6
ENSP00000308887	216	WNT5B
ENSP00000205143	216	DLL3
ENSP00000304669	216	CTNNA1
ENSP00000256495	216	BHLHE40
ENSP00000247970	215	PIN1
ENSP00000346151	215	MAGI2
ENSP00000254231	215	LIN28A
ENSP00000315147	215	ISYNA1
ENSP00000364310	215	H2AFX
ENSP00000262493	215	GNAO1
ENSP00000247843	214	YEATS4
ENSP00000265131	214	TNC
ENSP00000262948	214	MAP2K2
ENSP00000302100	214	LGALS4
ENSP00000222725	214	LFNG
ENSP00000364389	214	CDC14B
ENSP00000309968	214	ADAM17
ENSP00000306043	214	-
ENSP00000317199	214	-
ENSP00000304308	213	ZNF217
ENSP00000347858	213	XIAP

ENSP00000247668	213	TRAF2
ENSP00000264708	213	POMC
ENSP00000348020	213	MLH3
ENSP00000216117	213	HMOX1
ENSP00000306920	213	GLB1
ENSP00000417257	213	FNDC3A
ENSP00000217244	213	CSNK2A1
ENSP00000299529	213	CRABP1
ENSP00000349508	213	CHD4
ENSP00000330237	213	CASP9
ENSP00000264487	213	AREG
ENSP00000234313	212	PLEK
ENSP00000333685	212	MAPK11
ENSP00000337439	212	DACT1
ENSP00000355759	211	PARP1
ENSP00000351908	211	MAP3K5
ENSP00000345083	211	MAP2K3
ENSP00000380605	211	LYPD1
ENSP00000253408	211	GFAP
ENSP00000263209	211	DGCR8
ENSP00000354621	210	SMURF1
ENSP00000311816	210	REST
ENSP00000260227	210	MMP7
ENSP00000395535	210	MECP2
ENSP00000293308	210	KRT8
ENSP00000349049	210	KDM1A
ENSP00000363689	210	ID3
ENSP00000342105	210	DYRK2
ENSP00000331103	209	IP6K2
ENSP00000345785	209	FZD9
ENSP00000357625	209	BNIP3
ENSP00000220781	209	-
ENSP00000282111	208	TCF7L1
ENSP00000302150	208	PRL
ENSP00000312988	208	NFKBIB
ENSP00000345530	208	NEDD4
ENSP00000349393	208	LIG4
ENSP00000387662	208	GCG
ENSP00000295206	208	EN1
ENSP00000245323	208	EFNB2
ENSP00000307305	208	DDIT4
ENSP00000355370	208	CNTF
ENSP00000265709	208	ANK1
ENSP00000257566	207	TBX3
ENSP00000262539	207	PTPN3
ENSP00000302189	207	NHLH1

ENSP00000412237	207	IL10
ENSP00000080059	207	HDAC7
ENSP00000180166	207	FGF20
ENSP00000358596	207	DUSP5
ENSP00000249749	207	DLL4
ENSP00000220003	207	CSK
ENSP00000259008	207	BRIP1
ENSP00000333920	206	TTF1
ENSP00000265970	206	PIK3C2A
ENSP00000219255	206	PARD6A
ENSP00000265081	206	MSH3
ENSP00000308208	206	MMP14
ENSP00000233809	206	IGFBP2
ENSP00000377372	206	GAP43
ENSP00000260433	206	CYP19A1
ENSP00000310491	206	ARHGAP1
ENSP00000352257	205	XRCC6
ENSP00000291527	205	TFF1
ENSP00000225577	205	RPS6KB1
ENSP00000382697	205	ROCK1
ENSP00000256429	205	MBD2
ENSP00000340820	205	MAPT
ENSP00000299421	205	ILK
ENSP00000351209	205	EPHA2
ENSP00000368683	205	EDN1
ENSP00000327513	205	CSF1
ENSP00000305873	205	CRTC2
ENSP00000364893	205	ARHGEF7
ENSP00000297450	205	ANGPT1
ENSP00000364519	204	TFDP1
ENSP00000274063	204	SFRP2
ENSP00000308620	204	RAG2
ENSP00000268182	204	IQGAP1
ENSP00000296575	204	HHIP
ENSP00000269216	204	GATA6
ENSP00000371790	204	FGF9
ENSP00000302530	204	BUB1
ENSP00000250160	203	WISP1
ENSP00000217086	203	SALL4
ENSP00000352157	203	MAPK10
ENSP00000304604	203	MAGI3
ENSP00000263851	203	IL7
ENSP00000355249	203	E2F2
ENSP00000343412	203	BRE
ENSP00000227758	203	BIRC2
ENSP00000226359	203	AFP

ENSP00000263640	203	ACVR1
ENSP00000376688	203	-
ENSP00000385485	203	-
ENSP00000340677	202	WNT8B
ENSP00000177694	202	TBX21
ENSP00000228644	202	MYF5
ENSP00000278823	202	MTA2
ENSP00000333633	202	MTA1
ENSP00000306157	202	IL7R
ENSP00000323880	202	FOXJ1
ENSP00000292408	202	FGFR4
ENSP00000258443	202	EDAR
ENSP00000290015	201	WNT9B
ENSP00000296504	201	SAP30
ENSP00000284384	201	PRKCA
ENSP00000301843	201	CTTN
ENSP00000352011	201	CACNA1G
ENSP00000231061	200	SPARC
ENSP00000381331	200	HDAC2
ENSP00000261532	200	ESRRB
ENSP00000261590	200	DSG2
ENSP00000312262	200	ADRBK1
ENSP00000305372	200	ADRB2
ENSP00000353863	199	ZNF148
ENSP00000358698	199	WNT2B
ENSP00000355747	199	PSEN2
ENSP00000261454	199	PROX1
ENSP00000398824	199	PHF21A
ENSP00000311697	199	FGF5
ENSP00000404179	199	DOCK4
ENSP00000361359	199	CD40
ENSP00000325690	199	CARM1
ENSP00000162330	199	BCAR1
ENSP00000340429	199	-
ENSP00000352262	199	-
ENSP00000376765	198	PIAS3
ENSP00000325863	198	MRE11A
ENSP00000231121	198	HAND1
ENSP00000381793	198	GRB10
ENSP00000363708	198	BMPR2
ENSP00000263084	198	-
ENSP00000311581	198	-
ENSP00000410211	198	-
ENSP00000269485	197	TNFRSF11A
ENSP00000365877	197	SUV39H1
ENSP00000297316	197	SOX17

ENSP00000352900	197	RXRG
ENSP00000381565	197	DIAPH1
ENSP00000404503	197	BBC3
ENSP00000224784	197	ACTA2
ENSP00000355645	197	ACTA1
ENSP00000267008	197	-
ENSP00000332698	197	-
ENSP00000262768	196	TIMP2
ENSP00000354130	196	SOX10
ENSP00000345826	196	PMEPA1
ENSP00000362413	196	PGK1
ENSP00000272641	196	NXPH2
ENSP00000306070	196	NUDT6
ENSP00000174618	196	MNT
ENSP00000299213	196	LARP6
ENSP00000354519	196	INPP5F
ENSP00000273430	196	AGTR1
ENSP00000233139	196	-
ENSP00000222462	195	WNT16
ENSP00000244007	195	PLCG1
ENSP00000263388	195	NOTCH3
ENSP00000417303	195	KLF8
ENSP00000386896	195	ITGA6
ENSP00000419564	195	IGHD6-13
ENSP00000360216	195	DNTT
ENSP00000354916	195	CDC14A
ENSP00000363019	194	UBE2D1
ENSP00000326119	194	PMAIP1
ENSP00000343325	194	PKN1
ENSP00000349041	194	NRARP
ENSP00000359240	194	LZTS2
ENSP00000354607	194	FZD5
ENSP00000246166	194	FNTB
ENSP00000228837	194	FGF6
ENSP00000342626	194	EYA1
ENSP00000307109	194	-
ENSP00000265077	193	VCAN
ENSP00000296145	193	TDGF1
ENSP00000377446	193	SUCLG1
ENSP00000293328	193	STAT5B
ENSP00000304283	193	RAC3
ENSP00000317580	193	NEUROG1
ENSP00000322788	193	MMP1
ENSP00000335311	193	EDNRB
ENSP00000297518	193	CDK5
ENSP00000225698	193	C1QBP

ENSP00000295897	193	ALB
ENSP00000391490	193	AGR2
ENSP00000355330	192	TGM2
ENSP00000353622	192	SIN3A
ENSP00000345752	192	MTMR2
ENSP00000294304	192	LRP5
ENSP00000263339	192	IL1A
ENSP00000239151	192	HOXB5
ENSP00000417404	192	HFE
ENSP00000362299	192	ENG
ENSP00000280892	192	EIF4E
ENSP00000246657	192	CCR7
ENSP00000320510	192	-
ENSP00000201031	191	TFAP2C
ENSP00000305355	191	PRKCB
ENSP00000296440	191	PLXNB1
ENSP00000321746	191	PDLIM5
ENSP00000347684	191	LMX1B
ENSP00000343246	191	HOXA1
ENSP00000286201	191	FZD7
ENSP00000351605	191	FZD6
ENSP00000294312	191	FGF19
ENSP00000262238	190	YY1
ENSP00000247182	190	SIX1
ENSP00000348944	190	RSPO1
ENSP00000369071	190	POSTN
ENSP00000343847	190	NEK10
ENSP00000272369	190	MEIS1
ENSP00000242208	190	INHBA
ENSP00000264832	190	ICAM1
ENSP00000360058	190	FRAT2
ENSP00000385269	190	ELAVL1
ENSP00000307134	190	CTNND2
ENSP00000216911	190	AURKA
ENSP00000381992	190	7-Sep
ENSP00000418684	190	-
ENSP00000343392	189	XRCC3
ENSP00000310928	189	PPARD
ENSP00000318472	189	NCAM1
ENSP00000249075	189	LIF
ENSP00000256999	189	FOLH1
ENSP00000239223	189	DUSP1
ENSP00000257600	189	DTX1
ENSP00000365643	189	DOCK9
ENSP00000279593	189	-
ENSP00000323670	188	ZBTB7A

ENSP00000342011	188	XRCC4
ENSP00000201586	188	SULT2B1
ENSP00000356346	188	PTPRC
ENSP00000357206	188	NES
ENSP00000410926	188	LMLN
ENSP00000263087	188	ITGAE
ENSP00000219700	188	HMOX2
ENSP00000184956	188	HEATR6
ENSP00000381098	188	GRIP1
ENSP00000338934	188	EZR
ENSP00000242577	188	DYNLL1
ENSP00000376849	188	CASP5
ENSP00000219865	188	-
ENSP00000405965	187	SUMO2
ENSP00000223095	187	SERPINE1
ENSP00000276659	187	RSPO2
ENSP00000368884	187	RPS6KA3
ENSP00000344479	187	NR4A2
ENSP00000362674	187	HDAC8
ENSP00000216271	187	HDAC10
ENSP00000403536	187	GAMT
ENSP00000256996	187	DDB2
ENSP00000225474	187	CSF3
ENSP00000384179	186	ZFPM2
ENSP00000330218	186	SOX1
ENSP00000360181	186	SH2D1A
ENSP00000361850	186	PLAU
ENSP00000397297	186	NTF3
ENSP00000260950	186	MSTN
ENSP00000357348	186	HEY2
ENSP00000326272	186	FOXL1
ENSP00000369889	186	COL2A1
ENSP00000315743	186	CCNG2
ENSP00000286186	186	CASP10
ENSP00000368678	186	AGRN
ENSP00000340361	186	ACVR2B
ENSP00000293813	186	-
ENSP00000312150	185	TRIB1
ENSP00000262241	185	RCOR1
ENSP00000300093	185	PLK1
ENSP00000363387	185	PDIK1L
ENSP00000253727	185	NR1H2
ENSP00000362057	185	NOX1
ENSP00000260302	185	MMP13
ENSP00000297904	185	FIGF
ENSP00000267843	185	FGF7

ENSP00000322898	185	EBF1
ENSP00000305714	185	BMP1
ENSP00000222567	184	TWISTNB
ENSP00000374205	184	TBR1
ENSP00000338235	184	MTDH
ENSP00000356070	184	MAPKAPK2
ENSP00000299163	184	HIF1AN
ENSP00000322909	184	FHL2
ENSP00000335320	184	BCL9L
ENSP00000250405	184	BCL2L2
ENSP00000365682	183	TLE1
ENSP00000368664	183	RGCC
ENSP00000324804	183	PPP2R1A
ENSP00000343819	183	OTX2
ENSP00000173229	183	NTN1
ENSP00000295108	183	NEUROD1
ENSP00000379625	183	MYD88
ENSP00000351997	183	MAP2K6
ENSP00000355124	183	KRT19
ENSP00000200181	183	ITGB4
ENSP00000333950	183	FMN1
ENSP00000318057	183	EGR3
ENSP00000352295	183	CGB2
ENSP00000319914	183	C8orf4
ENSP00000332744	183	ALX4
ENSP00000298139	182	WRN
ENSP00000260010	182	TLR2
ENSP00000393286	182	RXRB
ENSP00000393870	182	RXRB
ENSP00000402590	182	RXRB
ENSP00000272190	182	REN
ENSP00000368959	182	REEP5
ENSP00000321999	182	PTH1R
ENSP00000381499	182	PAX1
ENSP00000365775	182	MTHFR
ENSP00000271555	182	MEF2D
ENSP00000346389	182	MEF2A
ENSP00000294816	182	LMX1A
ENSP00000304791	182	KLK7
ENSP00000243786	182	INHA
ENSP00000334061	182	HDAC6
ENSP00000238558	182	GSC
ENSP00000240093	182	FZD3
ENSP00000289081	182	FANCC
ENSP00000337445	182	ELAC2
ENSP00000301764	182	DDB1

ENSP00000307297	182	CHST14
ENSP00000347582	182	CGB8
ENSP00000348545	182	CGB7
ENSP00000301408	182	CGB5
ENSP00000318486	182	CDK5R1
ENSP00000327336	182	BGN
ENSP00000351575	182	BDP1
ENSP00000246789	182	-
ENSP00000357040	181	VANGL2
ENSP00000218388	181	TIMP1
ENSP00000301691	181	SOST
ENSP00000386759	181	SETD2
ENSP00000259808	181	RIPK1
ENSP00000360290	181	PRKAA2
ENSP00000282077	181	PDK1
ENSP00000254457	181	LHX1
ENSP00000278187	181	GAS2
ENSP00000220812	181	DKK4
ENSP00000285311	181	DKK2
ENSP00000357204	181	CRABP2
ENSP00000325660	181	CNTN1
ENSP00000301407	181	CGB1
ENSP00000312455	181	CFLAR
ENSP00000245960	181	CDC25B
ENSP00000302216	181	ATOH1
ENSP00000360689	180	TNKS2
ENSP00000392423	180	RELN
ENSP00000344936	180	PTTG1
ENSP00000363921	180	PARD3
ENSP00000262811	180	MAST3
ENSP00000354671	180	MAST2
ENSP00000298910	180	LRRK2
ENSP00000272134	180	LEFTY1
ENSP00000406037	180	KAT8
ENSP00000267082	180	ITGB7
ENSP00000230658	180	ISL1
ENSP00000306512	180	IL8
ENSP00000294702	180	GFI1
ENSP00000262995	180	GAB1
ENSP00000243222	180	COL10A1
ENSP00000358595	180	CGA
ENSP00000338673	180	CDK10
ENSP00000367608	180	CA9
ENSP00000287295	180	AIFM1
ENSP00000258411	179	WNT10A
ENSP00000332468	179	TRAF3

ENSP00000231487	179	SKP1
ENSP00000327025	179	RORC
ENSP00000364860	179	ROR2
ENSP00000271411	179	POU2F1
ENSP00000325312	179	METAP2
ENSP00000360060	179	FRAT1
ENSP00000301607	179	EVPL
ENSP00000398736	179	CYR61
ENSP00000333873	179	CIB1
ENSP00000406288	179	CHD8
ENSP00000344635	179	CCNG1
ENSP00000320461	179	ASCC1
ENSP00000314414	179	AP2B1
ENSP00000329908	179	-
ENSP00000297350	178	TNFRSF11B
ENSP00000262418	178	SLC4A1
ENSP00000309591	178	PRKACA
ENSP00000319788	178	NQO1
ENSP00000296849	178	NKD2
ENSP00000254301	178	LGALS3
ENSP00000248071	178	KLF2
ENSP00000290341	178	IGF2BP1
ENSP00000340292	178	DLK1
ENSP00000264568	178	BMPR1B
ENSP00000275248	178	ARID1B
ENSP00000361027	178	AMOT
ENSP00000308226	178	-
ENSP00000315768	177	STAT2
ENSP00000348069	177	SREBF1
ENSP00000372703	177	RXRB
ENSP00000411397	177	MARK3
ENSP00000268171	177	FURIN
ENSP00000349467	177	CALM1
ENSP00000310800	176	VANGL1
ENSP00000222305	176	USF2
ENSP00000311579	176	TNKS
ENSP00000308927	176	SOX5
ENSP00000400104	176	RXRB
ENSP00000333568	176	PRKD1
ENSP00000263556	176	P4HA1
ENSP00000309336	176	HOXC5
ENSP00000308252	176	HOXB3
ENSP00000283179	176	HNRNPU
ENSP00000302251	176	GBX2
ENSP00000363826	176	FZD8
ENSP00000234198	176	DLX2

ENSP00000318351	176	BCKDHB
ENSP00000285021	175	XPC
ENSP00000364270	175	XPA
ENSP00000376350	175	TPCN1
ENSP00000308937	175	PMP22
ENSP00000397157	175	PERP
ENSP00000282493	175	PDZD2
ENSP00000265371	175	NRP1
ENSP00000317333	175	NEUROG2
ENSP00000329411	175	IRF7
ENSP00000262626	175	HPN
ENSP00000000442	175	ESRRA
ENSP00000312697	175	DMAP1
ENSP00000266991	175	DHH
ENSP00000344259	174	UBE2L3
ENSP00000371475	174	TP53BP1
ENSP00000268989	174	SGSM2
ENSP00000324729	174	SAV1
ENSP00000308413	174	RPS6KB2
ENSP00000281171	174	PTPRO
ENSP00000261207	174	PPP1R12A
ENSP00000381648	174	PIAS2
ENSP00000375986	174	MAP3K4
ENSP00000239165	174	HOXB7
ENSP00000259456	174	HEMGN
ENSP00000295757	174	HDAC11
ENSP00000352414	174	FGF17
ENSP00000287647	174	FANCD2
ENSP00000262375	174	DNAJA3
ENSP00000231656	174	CDX1
ENSP00000258682	174	CAMK2B
ENSP00000262017	174	-
ENSP00000389817	174	-
ENSP00000260128	173	SULF1
ENSP00000295736	173	SLC4A7
ENSP00000332695	173	RARG
ENSP00000418447	173	PPP2CA
ENSP00000238607	173	PGF
ENSP00000348099	173	PDLIM7
ENSP00000373487	173	KRT18
ENSP00000369581	173	IFNB1
ENSP00000248553	173	HSPB1
ENSP00000352565	173	HAND2
ENSP00000261858	173	GLCE
ENSP00000378735	173	DNAJC6
ENSP00000292535	173	CUX1

ENSP00000414187	173	CPEB1
ENSP00000253968	173	BARX1
ENSP00000374357	173	ARNTL
ENSP00000262999	172	UCP1
ENSP00000370543	172	SLC5A3
ENSP00000340463	172	S100A14
ENSP00000370113	172	PPP2R2A
ENSP00000261537	172	MIB1
ENSP00000300651	172	MED1
ENSP00000362717	172	LHX2
ENSP00000281821	172	EPHA4
ENSP00000263991	172	EHBP1
ENSP00000007660	172	DLX6
ENSP00000296871	172	CSF2
ENSP00000346697	172	BMF
ENSP00000288266	172	APPL1
ENSP00000237612	172	ABCG2
ENSP00000336946	172	-
ENSP00000233948	171	WNT6
ENSP00000371693	171	TNFRSF19
ENSP00000296084	171	RYK
ENSP00000230419	171	PTK7
ENSP00000367817	171	PODXL
ENSP00000364802	171	HSPA1A
ENSP00000332194	171	HIST2H2AC
ENSP00000249598	171	GDF2
ENSP00000296839	171	FOXQ1
ENSP00000369857	171	FAM175A
ENSP00000354923	171	DMD
ENSP00000052754	171	DCN
ENSP00000334130	171	C11orf30
ENSP00000329246	171	-
ENSP00000355751	170	THBS2
ENSP00000341815	170	SOX18
ENSP00000265335	170	RAD50
ENSP00000368253	170	NR0B1
ENSP00000331775	170	LIMS1
ENSP00000377492	170	HMMR
ENSP00000339250	170	DPPA3
ENSP00000224356	170	CYP26A1
ENSP00000381998	170	BRCC3
ENSP00000348455	169	UVRAG
ENSP00000263321	169	TYR
ENSP00000262435	169	SMURF2
ENSP00000261707	169	SLC6A4
ENSP00000396439	169	RING1

ENSP00000306561	169	OGG1
ENSP00000407879	169	MYH14
ENSP00000281923	169	MGAT5
ENSP00000363489	169	GDF5
ENSP00000228862	169	DUSP16
ENSP00000254322	169	DNAJB1
ENSP00000409346	169	CISH
ENSP00000313419	169	CD19
ENSP00000313046	169	BAI1
ENSP00000290378	169	ACTC1
ENSP00000216612	169	-
ENSP00000358202	169	-
ENSP00000324203	168	WRAP53
ENSP00000272164	168	WNT9A
ENSP00000230354	168	TBP
ENSP00000345512	168	SEMA6A
ENSP00000222254	168	PIK3R2
ENSP00000299855	168	MMP3
ENSP00000265986	168	IDE
ENSP00000308533	168	GEMIN2
ENSP00000312789	168	DYRK1B
ENSP00000295522	168	CLDN1
ENSP00000355731	168	CDC42BPA
ENSP00000286827	167	TIAM1
ENSP00000332995	167	SETD8
ENSP00000261509	167	PALLD
ENSP00000343479	167	NBR1
ENSP00000406861	167	MUC6
ENSP00000307235	167	EIF2AK3
ENSP00000304408	167	COL3A1
ENSP00000313854	167	CLPTM1L
ENSP00000287322	167	BAG4
ENSP00000362300	167	AGO1
ENSP00000264972	166	ZAP70
ENSP00000303208	166	PCSK9
ENSP00000265433	166	NBN
ENSP00000352852	166	MDK
ENSP00000257745	166	KMT2E
ENSP00000216223	166	IL2RB
ENSP00000252997	166	GATA5
ENSP00000358391	166	FCGR1B
ENSP00000263735	166	EPCAM
ENSP00000306356	166	ENC1
ENSP00000312664	166	CASP2
ENSP00000308774	166	BMX
ENSP00000262713	166	AJUBA

ENSP00000406437	166	-
ENSP00000190983	165	WISP2
ENSP00000266025	165	TMEM115
ENSP00000276072	165	TAF1
ENSP00000355924	165	SMYD2
ENSP00000288840	165	SMAD6
ENSP00000362592	165	RBBP4
ENSP00000342931	165	POU1F1
ENSP00000364204	165	PINK1
ENSP00000243924	165	PI3
ENSP00000215781	165	OSM
ENSP00000327213	165	NRIP1
ENSP00000337773	165	NQO2
ENSP00000297689	165	NFIL3
ENSP00000358622	165	IKBKG
ENSP00000317904	165	GYS1
ENSP00000415615	165	CSNK2B
ENSP00000349954	165	CGB
ENSP00000249364	165	CALU
ENSP00000342059	165	-
ENSP00000264316	164	TXK
ENSP00000162749	164	TNFRSF1A
ENSP00000386341	164	TICAM2
ENSP00000237316	164	TCF21
ENSP00000332816	164	PTK2B
ENSP00000371432	164	PRLR
ENSP00000339328	164	PLAUR
ENSP00000349465	164	PICK1
ENSP00000234111	164	ODC1
ENSP00000283249	164	ITGB6
ENSP00000412283	164	IER3
ENSP00000343040	164	HMGB1
ENSP00000282728	164	HHEX
ENSP00000337014	164	HFE2
ENSP00000261195	164	GYS2
ENSP00000342445	164	CLDN4
ENSP00000229335	164	AICDA
ENSP00000287396	164	-
ENSP00000379884	164	-
ENSP00000381450	164	-
ENSP00000322845	163	ZMYM3
ENSP00000377265	163	TFAP2B
ENSP00000217964	163	TBL1X
ENSP00000329967	163	TBK1
ENSP00000360120	163	ROR1
ENSP00000346148	163	PRKAA1

ENSP00000378323	163	PPP3CA
ENSP00000385450	163	MAGI1
ENSP00000330393	163	LEPR
ENSP00000295228	163	INHBB
ENSP00000296545	163	IL15
ENSP00000371634	163	IGF2BP2
ENSP00000367276	163	CKAP2
ENSP00000265381	163	APBA1
ENSP00000300086	162	TERF2IP
ENSP00000293897	162	SSTR5
ENSP00000301904	162	SCARA3
ENSP00000363812	162	RXRB
ENSP00000379213	162	PTHLH
ENSP00000000412	162	M6PR
ENSP00000359942	162	KLHL31
ENSP00000301464	162	IGFBP6
ENSP00000324173	162	HSPA5
ENSP00000229030	162	FZD10
ENSP00000360157	162	FOXD3
ENSP00000229277	162	ENO2
ENSP00000347596	162	EFEMP1
ENSP00000225387	162	CRYBA1
ENSP00000370297	162	CER1
ENSP00000243349	162	ACVR1C
ENSP00000408005	161	SLC9A3R2
ENSP00000306734	161	SESN1
ENSP00000221486	161	RNASEH2A
ENSP00000314151	161	KLK3
ENSP00000304915	161	IL13
ENSP00000357113	161	IFI16
ENSP00000276414	161	GNRH1
ENSP00000265094	161	FBXW11
ENSP00000378577	161	CLDN3
ENSP00000234626	161	CDC7
ENSP00000316845	161	ARHGEF4
ENSP00000265724	161	ABCB1
ENSP00000243347	160	TNFAIP6
ENSP00000383977	160	TEK
ENSP00000217188	160	SRMS
ENSP00000217254	160	SLC52A3
ENSP00000356832	160	SGK1
ENSP00000252785	160	SCO2
ENSP00000285735	160	RHOC
ENSP00000228918	160	LTBR
ENSP00000231228	160	IL12B
ENSP00000337477	160	FAM60A

ENSP00000261798	160	CSNK1A1
ENSP00000354511	160	COMT
ENSP00000310670	160	CCAR2
ENSP00000219611	160	CAPN15
ENSP00000365906	159	ZNF615
ENSP00000317686	159	ZFP42
ENSP00000276603	159	TERF1
ENSP00000286332	159	TAB2
ENSP00000349274	159	PPP1R26
ENSP00000202556	159	PPP1R13B
ENSP00000329170	159	POU3F2
ENSP00000301396	159	PELP1
ENSP00000278568	159	PAK1
ENSP00000347379	159	OCLN
ENSP00000277120	159	NTRK2
ENSP00000242462	159	NEUROG3
ENSP00000269468	159	MBD1
ENSP00000381066	159	MAP2K7
ENSP00000304592	159	FASN
ENSP00000215368	159	EFNA2
ENSP00000312995	159	CLSPN
ENSP00000379065	158	TRPS1
ENSP00000341268	158	TRADD
ENSP00000260810	158	TOPBP1
ENSP00000353007	158	SULF2
ENSP00000371527	158	SPATA13
ENSP00000339467	158	RHOG
ENSP00000264025	158	PVRL1
ENSP00000260404	158	PAK6
ENSP00000308383	158	LSP1
ENSP00000266674	158	LGR5
ENSP00000256594	158	GSTM3
ENSP00000363071	158	DES
ENSP00000228606	158	CYP27B1
ENSP00000287641	157	SST
ENSP00000304930	157	SOSTDC1
ENSP00000363277	157	RPS6KA1
ENSP00000366413	157	POU4F1
ENSP00000253255	157	PKDREJ
ENSP00000253055	157	MAP3K10
ENSP00000297293	157	LMTK2
ENSP00000365016	157	IRS2
ENSP00000362924	157	GSN
ENSP00000247005	157	GDF1
ENSP00000264716	157	FOSL2
ENSP00000222139	157	EPOR

ENSP00000370936	157	E2F6
ENSP00000261038	157	DIRC2
ENSP00000364979	157	COL4A1
ENSP00000344115	157	CDH5
ENSP00000340842	157	C19orf67
ENSP00000322049	157	-
ENSP00000377422	157	-
ENSP00000216923	156	ZFP64
ENSP00000217381	156	SNTA1
ENSP00000369154	156	SMAD9
ENSP00000322457	156	SIAH2
ENSP00000291700	156	S100B
ENSP00000261424	156	RFC1
ENSP00000306124	156	PRKCE
ENSP00000364902	156	POFUT1
ENSP00000359552	156	PKN2
ENSP00000262741	156	PIK3R3
ENSP00000368190	156	NPHS1
ENSP00000268605	156	NOL3
ENSP00000322408	156	KDM5D
ENSP00000007722	156	ITGA3
ENSP00000358151	156	HIST2H2BE
ENSP00000419945	156	ERVW-1
ENSP00000222005	156	CDC37
ENSP00000357668	156	ADAM12
ENSP00000378517	155	SPP1
ENSP00000299759	155	RRAD
ENSP00000350052	155	POTEF
ENSP00000377934	155	KIF7
ENSP00000368020	155	KIF3A
ENSP00000227378	155	HSPA8
ENSP00000204637	155	FLT3LG
ENSP00000332049	155	CD86
ENSP00000370989	155	CD274
ENSP00000303909	155	ABR
ENSP00000262795	155	-
ENSP00000296325	155	-
ENSP00000329528	155	-
ENSP00000265440	154	TFEC
ENSP00000263073	154	SMG6
ENSP00000345464	154	NHLRC1
ENSP00000344192	154	IL17A
ENSP00000296875	154	GDF9
ENSP00000258106	154	EMX1
ENSP00000397552	154	ACTL6A
ENSP00000310908	154	-

ENSP00000377867	154	-
ENSP00000309992	153	ZMYND11
ENSP00000370571	153	TH
ENSP00000367923	153	SUCLA2
ENSP00000374455	153	SQSTM1
ENSP00000344967	153	SPRY4
ENSP00000360310	153	SPO11
ENSP00000305847	153	SPACA5
ENSP00000262188	153	SMARCD3
ENSP00000220478	153	SCG3
ENSP00000251020	153	SALL1
ENSP00000323191	153	SAGE1
ENSP00000365152	153	MYLK2
ENSP00000257552	153	MSI1
ENSP00000358335	153	MAP3K7
ENSP00000231454	153	IL5
ENSP00000290573	153	HK2
ENSP00000393725	153	GFRA1
ENSP00000354859	153	DRD2
ENSP00000405798	153	CDK16
ENSP00000341940	153	CAV3
ENSP00000381739	152	WNT8A
ENSP00000366434	152	UIMC1
ENSP00000256592	152	TSHB
ENSP00000233143	152	TMSB10
ENSP00000366453	152	TJP2
ENSP00000254480	152	SMARCC1
ENSP00000227918	152	SCGB2A2
ENSP00000362103	152	POU3F1
ENSP00000288986	152	NCK1
ENSP00000354499	152	MT-CO1
ENSP00000321684	152	MEOX1
ENSP00000215659	152	MAPK12
ENSP00000264741	152	ITGA9
ENSP00000357583	152	INPP5A
ENSP00000258729	152	IGF2BP3
ENSP00000340935	152	DMP1
ENSP00000352712	152	DACH1
ENSP00000384554	152	CLDN5
ENSP00000361189	152	CDHR1
ENSP00000380256	152	CCNF
ENSP00000220659	152	BRF2
ENSP00000224112	152	-
ENSP00000417884	152	-
ENSP00000253024	151	TRIM28
ENSP00000371341	151	TNK2

ENSP00000270142	151	SOD1
ENSP00000320935	151	SLC2A4
ENSP00000304447	151	PTCRA
ENSP00000339495	151	NELFB
ENSP00000327465	151	MYT1
ENSP00000156825	151	MBD3
ENSP00000274364	151	IQGAP2
ENSP00000242159	151	HOXA7
ENSP00000369600	151	FRY
ENSP00000265348	151	CUL7
ENSP00000332293	151	ASCL2
ENSP00000261980	150	VSX2
ENSP00000264818	150	TYK2
ENSP00000326261	150	SRRM1
ENSP00000361400	150	SFTPA2
ENSP00000387176	150	POTEJ
ENSP00000392718	150	POTEI
ENSP00000354681	150	OPA1
ENSP00000388910	150	NFATC4
ENSP00000372793	150	LTA
ENSP00000357283	150	LMNA
ENSP00000370719	150	ITSN1
ENSP00000264563	150	IL11
ENSP00000379228	150	HECW1
ENSP00000370376	150	DUT
ENSP00000371711	150	DMRT1
ENSP00000354478	150	DLX1
ENSP00000356024	150	CR2
ENSP00000299335	150	COX11
ENSP00000388548	150	CITED1
ENSP00000261211	150	CDK17
ENSP00000269385	150	CBX8
ENSP00000360797	150	CARD9
ENSP00000178638	150	CA12
ENSP00000327214	150	ATP13A2
ENSP00000333037	150	ACTRT3
ENSP00000416706	150	ACTBL2
ENSP00000342589	150	-
ENSP00000304697	149	UBB
ENSP00000363463	149	TMEM57
ENSP00000262953	149	TLE2
ENSP00000291525	149	TFF3
ENSP00000245222	149	SPHK2
ENSP00000339428	149	SOCS2
ENSP00000274031	149	SETD7
ENSP00000356574	149	QSOX1

ENSP00000311290	149	PROP1
ENSP00000254657	149	PER2
ENSP00000375855	149	PDC
ENSP00000379330	149	NFATC2
ENSP00000352834	149	MYO1C
ENSP00000378400	149	LIMA1
ENSP00000256707	149	KIDINS220
ENSP00000264748	149	FGFRL1
ENSP00000325527	149	FBN1
ENSP00000363480	149	DLG3
ENSP00000410076	149	CASP1
ENSP00000282701	149	BMP3
ENSP00000356919	149	ATF6
ENSP00000160382	149	ACTL6B
ENSP00000268134	149	-
ENSP00000358677	148	TBX18
ENSP00000307684	148	TADA3
ENSP00000225688	148	RASD1
ENSP00000355923	148	PTPN14
ENSP00000344524	148	PPP1R9A
ENSP00000379003	148	NUPR1
ENSP00000263317	148	NOX4
ENSP00000316950	148	MYEF2
ENSP00000375863	148	HNRNPUL1
ENSP00000367714	148	HES5
ENSP00000281092	148	FER
ENSP00000256104	148	FABP4
ENSP00000401445	148	ERN1
ENSP00000013807	148	ERCC1
ENSP00000356489	148	EPM2A
ENSP00000352721	148	DNM2
ENSP00000316670	148	DHRS9
ENSP00000264246	148	CD80
ENSP00000310697	148	BRSK2
ENSP00000267953	148	BCL2A1
ENSP00000250111	148	ATP1B2
ENSP00000343313	148	ATG5
ENSP00000349796	147	ZNF23
ENSP00000363390	147	TRIM63
ENSP00000299084	147	SPRED1
ENSP00000356591	147	SOAT1
ENSP00000307369	147	SHE
ENSP00000319610	147	NPAS3
ENSP00000326296	147	MEIS2
ENSP00000252444	147	LDLR
ENSP00000269576	147	KRT10

ENSP00000365280	147	ID1
ENSP00000322542	147	GTF2I
ENSP00000381097	147	EPHB1
ENSP00000307197	147	DNAJB7
ENSP00000260630	147	CYP1B1
ENSP00000349147	147	CNNM1
ENSP00000308450	147	CDC20
ENSP00000359663	147	CD40LG
ENSP00000349722	146	UBE2D3
ENSP00000283916	146	TMPRSS11D
ENSP00000353874	146	TLR9
ENSP00000257915	146	TFCP2
ENSP00000367802	146	TAF1C
ENSP00000336655	146	STRADA
ENSP00000278412	146	SSRP1
ENSP00000338345	146	SNCA
ENSP00000265195	146	SIL1
ENSP00000301522	146	PRDX2
ENSP00000252115	146	POLDIP3
ENSP00000355245	146	PAX9
ENSP00000396843	146	NFIC
ENSP00000221421	146	LHB
ENSP00000370812	146	ESM1
ENSP00000323246	146	E2F7
ENSP00000258534	146	DRAM1
ENSP00000370503	146	CCM2
ENSP00000274459	146	ATG12
ENSP00000338160	146	-
ENSP00000284273	145	UBASH3B
ENSP00000225396	145	TADA2A
ENSP00000257895	145	RDH5
ENSP00000296122	145	PPP1CB
ENSP00000220809	145	PLAT
ENSP00000359019	145	PITX3
ENSP00000356155	145	PIK3C2B
ENSP00000327251	145	NOS2
ENSP00000353679	145	MME
ENSP00000235329	145	MFN2
ENSP00000211287	145	MAPK13
ENSP00000302961	145	HSPA4
ENSP00000259806	145	FOXF2
ENSP00000304250	145	CDK5R2
ENSP00000009180	145	CD9
ENSP00000295137	145	ACTG2
ENSP00000276110	145	-
ENSP00000294728	144	VCAM1

ENSP00000355652	144	RHOU
ENSP00000249071	144	RAC2
ENSP00000342385	144	PTGES
ENSP00000298510	144	PRDX3
ENSP00000247271	144	OMG
ENSP00000228641	144	MYF6
ENSP00000378733	144	LBH
ENSP00000366534	144	FOXH1
ENSP00000265071	144	CDH6
ENSP00000256897	144	CCNH
ENSP00000263845	144	-
ENSP00000386239	143	TONSL
ENSP00000216160	143	TAB1
ENSP00000216297	143	SUPT16H
ENSP00000360938	143	SARDH
ENSP00000305416	143	S1PR1
ENSP00000418112	143	PTPRG
ENSP00000314949	143	POLR2A
ENSP00000242248	143	POLM
ENSP00000274276	143	OSMR
ENSP00000257572	143	HRK
ENSP00000078429	143	GNA11
ENSP00000345997	143	DMPK
ENSP00000366237	143	DFFA
ENSP00000302485	143	CSDC2
ENSP00000251071	143	CAPRIN2
ENSP00000289968	143	ARHGAP17
ENSP00000360905	143	AGBL4
ENSP00000395625	143	-
ENSP00000289373	142	TMSB15A
ENSP00000372547	142	SRY
ENSP00000254351	142	SDC1
ENSP00000356452	142	PRG4
ENSP00000415183	142	MUC2
ENSP00000307288	142	MCM7
ENSP00000264036	142	MCAM
ENSP00000265960	142	MAPKAP1
ENSP00000313644	142	MAP4K4
ENSP00000243077	142	LRP1
ENSP00000329243	142	KRT7
ENSP00000308716	142	INHBC
ENSP00000382791	142	GRIK1
ENSP00000407375	142	GPX1
ENSP00000221856	142	FSD1
ENSP00000247170	142	DAAM1
ENSP00000322343	142	CDK20

ENSP00000324890	142	CD28
ENSP00000259631	142	CCL27
ENSP00000388566	142	CASP4
ENSP00000334424	142	AMACR
ENSP00000334300	142	ACTL7A
ENSP00000331514	142	ACTG1
ENSP00000410312	142	-
ENSP00000262394	141	WSB1
ENSP00000316357	141	USP9X
ENSP00000376445	141	TIRAP
ENSP00000261313	141	PEBP1
ENSP00000347507	141	MYH7
ENSP00000337889	141	MXD4
ENSP00000246635	141	KRT13
ENSP00000298229	141	INPPL1
ENSP00000331358	141	GAST
ENSP00000297375	141	EN2
ENSP00000362014	141	DNM1
ENSP00000263196	141	DGCR2
ENSP00000272193	141	-
ENSP00000319371	141	-
ENSP00000268674	140	TIGD7
ENSP00000378332	140	TGFB1I1
ENSP00000319192	140	STK17A
ENSP00000300134	140	STAT6
ENSP00000306461	140	SPRR1B
ENSP00000304133	140	SCG2
ENSP00000295902	140	PRICKLE2
ENSP00000226382	140	PHOX2B
ENSP00000259526	140	NOV
ENSP00000371875	140	NKX3-2
ENSP00000264444	140	MXD1
ENSP00000378856	140	LGALS9
ENSP00000254043	140	KRT15
ENSP00000360200	140	INADL
ENSP00000228534	140	IL23A
ENSP00000319118	140	GSX2
ENSP00000364709	140	F10
ENSP00000166244	140	EPHA8
ENSP00000265741	140	CDK14
ENSP00000263201	140	CDC45
ENSP00000259607	140	CCL21
ENSP00000241052	140	CAT
ENSP00000292401	140	AZGP1
ENSP00000225973	140	-
ENSP00000271015	140	-

ENSP00000302452	140	-
ENSP00000320295	140	-
ENSP00000348031	140	-
ENSP00000349391	140	-
ENSP00000381977	140	-
ENSP00000399255	140	-
ENSP00000226218	139	VTN|SEBOX
ENSP00000311127	139	VEGFB
ENSP00000302640	139	UBTF
ENSP00000309532	139	TP53I11
ENSP00000358089	139	TIAL1
ENSP00000295987	139	SYN1
ENSP00000254722	139	SERPINF1
ENSP00000310572	139	PSMC5
ENSP00000355719	139	PRSS38
ENSP00000282549	139	OTX1
ENSP00000303398	139	MTBP
ENSP00000301263	139	LY6D
ENSP00000294954	139	LHCGR
ENSP00000258886	139	IREB2
ENSP00000275525	139	IGFBP1
ENSP00000381932	139	DYRK1A
ENSP00000362849	139	CTNNA3
ENSP00000311083	139	CKS1B
ENSP00000204604	139	CHRD
ENSP00000368244	139	CHGB
ENSP00000306340	139	CDKL2
ENSP00000398880	139	CDK12
ENSP00000299402	139	APBB1
ENSP00000264335	138	YWHAE
ENSP00000262887	138	XRCC1
ENSP00000361458	138	TSC22D3
ENSP00000300403	138	TPX2
ENSP00000262316	138	RHBDF1
ENSP00000332274	138	PTP4A3
ENSP00000261461	138	PPP2R5A
ENSP00000336591	138	PPP2R2B
ENSP00000352336	138	PLCG2
ENSP00000279022	138	MYL9
ENSP00000261681	138	MPP5
ENSP00000360811	138	LHX3
ENSP00000374309	138	LAMA1
ENSP00000226319	138	JADE1
ENSP00000303242	138	ITGB2
ENSP00000218006	138	GUCY2F
ENSP00000259803	138	GCM1

ENSP00000304286	138	FOXI1
ENSP00000342805	138	FAIM
ENSP00000281172	138	EPS8
ENSP00000263621	138	ELANE
ENSP00000236671	138	CTSD
ENSP00000262506	138	CSNK2A2
ENSP00000262738	138	CELSR1
ENSP00000341698	138	AKT1S1
ENSP00000263776	138	ACCS
ENSP00000232892	138	AADAC
ENSP00000383360	138	-
ENSP00000252029	137	TYMP
ENSP00000240335	137	TBX4
ENSP00000302139	137	SYNPO
ENSP00000313681	137	SPHK1
ENSP00000329213	137	SIVA1
ENSP00000343418	137	SEMA4D
ENSP00000361818	137	SDC4
ENSP00000043402	137	RTN4R
ENSP00000359685	137	PTP4A1
ENSP00000237596	137	PKD2
ENSP00000365651	137	NPPB
ENSP00000264834	137	KLF1
ENSP00000264735	137	HRASLS
ENSP00000371420	137	GPNMB
ENSP00000366013	137	GNB2L1
ENSP00000363680	137	EDA
ENSP00000355760	137	DACT2
ENSP00000215574	137	CDC34
ENSP00000301178	137	AXL
ENSP00000005198	137	-
ENSP00000320664	137	-
ENSP00000352271	136	XRCC2
ENSP00000309622	136	TFDP2
ENSP00000279386	136	TBX6
ENSP00000263233	136	SYP
ENSP00000216373	136	SOS2
ENSP00000380921	136	SH3KBP1
ENSP00000320768	136	RCAN1
ENSP00000298942	136	PTER
ENSP00000217800	136	POLI
ENSP00000274071	136	PDGFC
ENSP00000349275	136	NRG1
ENSP00000359423	136	MTM1
ENSP00000395102	136	LTB
ENSP00000348384	136	LAMB3

ENSP00000369293	136	IL2RA
ENSP00000375028	136	IGHV4-39
ENSP00000413496	136	FGF12
ENSP00000264638	136	CNTNAP1
ENSP00000221169	136	-
ENSP00000295566	135	YY1AP1
ENSP00000365576	135	UBQLN1
ENSP00000328973	135	TSPO
ENSP00000370007	135	TMSB4X
ENSP00000363392	135	SNX12
ENSP00000262288	135	SCPEP1
ENSP00000299440	135	RAG1
ENSP00000308938	135	PLG
ENSP00000342793	135	PLD1
ENSP00000367316	135	ITGA8
ENSP00000290219	135	IFNGR2
ENSP00000358421	135	HSD3B1
ENSP00000338082	135	HBG2
ENSP00000322421	135	HBA1
ENSP00000359998	135	GSTA4
ENSP00000332549	135	GRIN2A
ENSP00000345217	135	GOLGA8J
ENSP00000340698	135	GIPC1
ENSP00000384708	135	FSHR
ENSP00000362344	135	FPGS
ENSP00000334472	135	FOXE3
ENSP00000352929	135	CSNK1E
ENSP00000363500	135	CLIC4
ENSP00000318799	135	BHLHE22
ENSP00000358062	135	AIM1
ENSP00000385345	135	-
ENSP00000392148	135	-
ENSP00000402945	135	-
ENSP00000223023	134	WASL
ENSP00000229201	134	TIMELESS
ENSP00000348215	134	SH3PXD2A
ENSP00000296257	134	SENP2
ENSP00000362937	134	RCC1
ENSP00000245457	134	PTGER2
ENSP00000249269	134	PMPCB
ENSP00000269300	134	PIK3R5
ENSP00000246672	134	NR1D1
ENSP00000300131	134	NAB2
ENSP00000269095	134	MPP2
ENSP00000292599	134	MAML1
ENSP00000274520	134	IL9

ENSP00000304004	134	FOXA3
ENSP00000413196	134	FAM160A1
ENSP00000358140	134	EIF3A
ENSP00000358716	134	DDX20
ENSP00000284031	134	DDAH1
ENSP00000337697	134	DCX
ENSP00000301141	134	CYP2A6
ENSP00000379158	134	CAST
ENSP00000331746	134	CALCA
ENSP00000216099	134	APOBEC3D
ENSP00000342623	134	-
ENSP00000368186	134	-
ENSP00000372291	134	-
ENSP00000366509	133	ZKSCAN4
ENSP00000307858	133	ZBTB4
ENSP00000373570	133	TYRP1
ENSP00000321058	133	TWF1
ENSP00000215567	133	TECR
ENSP00000302327	133	PYGO1
ENSP00000375557	133	POU5F1B
ENSP00000257118	133	PHC2
ENSP00000415793	133	MUC5B
ENSP00000225275	133	MPO
ENSP00000354341	133	MEPE
ENSP00000353098	133	MCMBP
ENSP00000226284	133	IBSP
ENSP00000249501	133	HOXD10
ENSP00000040584	133	HOXC8
ENSP00000374265	133	GPX2
ENSP00000226247	133	FOXN1
ENSP00000323355	133	FAM187B
ENSP00000355596	133	DISC1
ENSP00000255448	133	DCLK1
ENSP00000355398	133	CTNNA2
ENSP00000227251	133	CRYAB
ENSP00000209875	133	CBX5
ENSP00000377380	133	ARHGAP9
ENSP00000261733	133	ALDH2
ENSP00000367658	133	ACTRT2
ENSP00000363799	133	ACTL7B
ENSP00000378430	133	-
ENSP00000216037	132	XBP1
ENSP00000314077	132	TSPY2
ENSP00000295924	132	TIPARP
ENSP00000359567	132	SOX3
ENSP00000351593	132	SMTN

ENSP00000369981	132	SH3GL2
ENSP00000253063	132	SESN2
ENSP00000342121	132	RNF6
ENSP00000281321	132	POU4F2
ENSP00000346127	132	PLEKHB1
ENSP00000262041	132	MEOX2
ENSP00000250894	132	MAPK8IP3
ENSP00000309597	132	MAP3K11
ENSP00000354560	132	KIFAP3
ENSP00000353151	132	HOXA4
ENSP00000400717	132	GNA13
ENSP00000229239	132	GAPDH
ENSP00000340742	132	DOCK7
ENSP00000276440	132	DOCK5
ENSP00000261891	132	DAPK2
ENSP00000337915	132	CYP3A4
ENSP00000350512	132	COPS5
ENSP00000383986	132	CDCA7L
ENSP00000348107	132	C1D
ENSP00000318585	132	BACE1
ENSP00000362441	132	ATRX
ENSP00000337261	132	ARHGEF1
ENSP00000363868	132	ABCA1
ENSP00000343140	131	XIRP1
ENSP00000347794	131	TRIM17
ENSP00000250018	131	TPH1
ENSP00000252996	131	TAF4
ENSP00000384675	131	SOS1
ENSP00000317985	131	ROCK2
ENSP00000341564	131	PAQR9
ENSP00000275015	131	NFKBIE
ENSP00000261597	131	NDC80
ENSP00000279441	131	MMP10
ENSP00000263205	131	MED15
ENSP00000261845	131	MAPK6
ENSP00000222304	131	HAMP
ENSP00000359506	131	FMR1
ENSP00000352182	131	FAM86C1
ENSP00000356705	131	DNM3
ENSP00000320434	131	CTNNAL1
ENSP00000360882	131	COL5A1
ENSP00000367748	131	CLLU1OS
ENSP00000316114	131	CCDC101
ENSP00000310275	131	BANF1
ENSP00000352324	131	ATXN3
ENSP00000320709	131	ADIPOQ

ENSP00000306606	131	ADH1B
ENSP00000330302	131	-
ENSP00000248975	130	YWHAH
ENSP00000216211	130	UPK3A
ENSP00000376611	130	TDG
ENSP00000261368	130	SNCAIP
ENSP00000304502	130	SIX2
ENSP00000356248	130	PTPN7
ENSP00000265340	130	PITX1
ENSP00000330271	130	NRBP2
ENSP00000320447	130	NR2C2
ENSP00000384523	130	NFIA
ENSP00000313437	130	MMP19
ENSP00000330572	130	MAPK8IP2
ENSP00000263056	130	MAP3K8
ENSP00000178640	130	MAP2K5
ENSP00000364847	130	MAGED1
ENSP00000261203	130	LIN7A
ENSP00000359074	130	L1CAM
ENSP00000222573	130	ITGB8
ENSP00000358997	130	IRAK1
ENSP00000318687	130	HSPH1
ENSP00000394842	130	GCA
ENSP00000310686	130	FBXW8
ENSP00000335076	130	EDARADD
ENSP00000264012	130	CDH3
ENSP00000242839	130	ATP7B
ENSP00000346437	130	ATG7
ENSP00000346032	130	ANXA2
ENSP00000374957	130	-
ENSP00000394135	130	-
ENSP00000415477	130	-
ENSP00000332325	129	ZNF703
ENSP00000265428	129	WWP1
ENSP00000245932	129	VASP
ENSP00000237014	129	TTR
ENSP00000419604	129	TRGJP1
ENSP00000366863	129	TBC1D4
ENSP00000265368	129	SYNE1
ENSP00000336750	129	SUPT7L
ENSP00000330343	129	SUMO3
ENSP00000219548	129	STUB1
ENSP00000217961	129	STS
ENSP00000378414	129	SMARCD1
ENSP00000321424	129	SLC34A1
ENSP00000363021	129	RPA2

ENSP00000349959	129	RICTOR
ENSP00000342830	129	RDX
ENSP00000338330	129	PAQR6
ENSP00000341911	129	PALM
ENSP00000385142	129	NRXN1
ENSP00000357982	129	NR2E1
ENSP00000307218	129	NAT1
ENSP00000306772	129	LDB2
ENSP00000251645	129	KRT31
ENSP00000328813	129	KCNH8
ENSP00000296464	129	HSPA4L
ENSP00000278715	129	HMBS
ENSP00000274341	129	HAPLN1
ENSP00000297146	129	GPR85
ENSP00000271348	129	GJA5
ENSP00000365295	129	FAM181B
ENSP00000310520	129	ERCC4
ENSP00000285398	129	ERCC3
ENSP00000258484	129	EPC2
ENSP00000226091	129	EFNB3
ENSP00000394352	129	DIXDC1
ENSP00000221954	129	CEACAM4
ENSP00000347602	129	ARID4A
ENSP00000250617	129	ARHGEF6
ENSP00000269321	129	ARHGDIA
ENSP00000358921	129	ACTR1A
ENSP00000262383	128	ZNF423
ENSP00000371532	128	VLDLR
ENSP00000354476	128	SREBF2
ENSP00000296589	128	SLC45A2
ENSP00000302324	128	RNASE3
ENSP00000332052	128	PCSK6
ENSP00000342828	128	NKX2-3
ENSP00000318195	128	NCL
ENSP00000223215	128	MEST
ENSP00000219782	128	MAZ
ENSP00000354927	128	MAP3K3
ENSP00000343463	128	MAP3K2
ENSP00000265713	128	KAT6A
ENSP00000373854	128	ITGAD
ENSP00000362115	128	INPP5B
ENSP00000225648	128	HOXB6
ENSP00000230882	128	GHR
ENSP00000347978	128	ERCC5
ENSP00000307786	128	CYCS
ENSP00000292301	128	CCR2

ENSP00000237654	128	CCNI
ENSP00000245157	128	BBS2
ENSP00000229595	128	ASF1A
ENSP00000350199	128	AP1B1
ENSP00000194097	128	-
ENSP00000327652	128	-
ENSP00000287727	127	ZFYVE9
ENSP00000328596	127	SIX6
ENSP00000221413	127	RUVBL2
ENSP00000238738	127	RHOQ
ENSP00000306335	127	RFX2
ENSP00000271331	127	PROK1
ENSP00000363092	127	PRKG1
ENSP00000264977	127	PPP2R3A
ENSP00000262735	127	PPARA
ENSP00000360158	127	PKHD1
ENSP00000343877	127	PAQR5
ENSP00000356331	127	NR5A2
ENSP00000347134	127	NET1
ENSP00000215057	127	MZF1
ENSP00000296474	127	MST1R
ENSP00000344223	127	MBTPS1
ENSP00000355884	127	MARK1
ENSP00000355785	127	LEFTY2
ENSP00000293330	127	HCRT
ENSP00000216465	127	GSTZ1
ENSP00000331831	127	GAS6
ENSP00000390590	127	DDN
ENSP00000325822	127	CYP11B2
ENSP00000271651	127	CTSK
ENSP00000198765	127	CPNE3
ENSP00000353654	127	COL4A2
ENSP00000369716	127	CHD3
ENSP00000256443	127	CDK7
ENSP00000351671	127	CCL20
ENSP00000307479	127	ARNT2
ENSP00000298316	127	ARF6
ENSP00000369530	127	ALOX15B
ENSP00000373490	127	-
ENSP00000381295	127	-
ENSP00000284856	126	TMSB4Y
ENSP00000316029	126	TLN1
ENSP00000394791	126	SENP1
ENSP00000296370	126	S100P
ENSP00000296271	126	RHO
ENSP00000359665	126	PI4K2A

ENSP00000420295	126	PDE6B
ENSP00000321133	126	OR52B1P
ENSP00000219066	126	NTHL1
ENSP00000333800	126	MRGPRX2
ENSP00000336740	126	LIMK1
ENSP00000380227	126	ITGA4
ENSP00000376609	126	GRK5
ENSP00000365301	126	FGF14
ENSP00000375809	126	ERCC2
ENSP00000216336	126	CTSG
ENSP00000271636	126	CGN
ENSP00000299345	126	CDH8
ENSP00000296129	126	CDCP1
ENSP00000225603	126	CBX1
ENSP00000366843	126	ATXN2
ENSP00000295598	126	ATP1A1
ENSP00000221561	126	AES
ENSP00000188312	126	ACTR6
ENSP00000381107	126	-
ENSP00000386092	126	-
ENSP00000321636	125	TOP3A
ENSP00000337513	125	SRA1
ENSP00000364995	125	SHC3
ENSP00000307272	125	RPTOR
ENSP00000261523	125	RORA
ENSP00000374013	125	PRB2
ENSP00000297565	125	OSR2
ENSP00000345702	125	NFYA
ENSP00000287239	125	KAT6B
ENSP00000361943	125	HEYL
ENSP00000222308	125	FKBP8
ENSP00000374213	125	EP400
ENSP00000369129	125	DSP
ENSP00000305913	125	COL8A2
ENSP00000331902	125	COL4A5
ENSP00000297265	125	CHMP4C
ENSP00000402697	125	CERS1
ENSP00000274695	125	CDKAL1
ENSP00000216133	125	CBX7
ENSP00000246785	125	BCL2L12
ENSP00000379290	125	BCAS1
ENSP00000263620	125	ARID3A
ENSP00000327968	124	SNAI3
ENSP00000392617	124	SMARCD2
ENSP00000266077	124	SLC2A4RG
ENSP00000365687	124	PTF1A

ENSP00000353582	124	NRP2
ENSP00000356587	124	NPHS2
ENSP00000264051	124	NGEF
ENSP00000297142	124	NEUROD6
ENSP00000373060	124	MDC1
ENSP00000241014	124	MAPK8IP1
ENSP00000327290	124	ITGA11
ENSP00000233946	124	IL1R1
ENSP00000310219	124	HSPA6
ENSP00000359531	124	GTF2B
ENSP00000306449	124	GPR37
ENSP00000354900	124	GJB1
ENSP00000357637	124	EBF3
ENSP00000355866	124	DUSP10
ENSP00000404623	124	DAXX
ENSP00000218507	124	CENPI
ENSP00000301019	124	CDT1
ENSP00000216378	124	CDKL1
ENSP00000362361	124	CDK9
ENSP00000349320	124	CACNA2D1
ENSP00000308176	124	BTK
ENSP00000259089	124	BLK
ENSP00000257749	124	BACH2
ENSP00000336923	124	ARHGAP10
ENSP00000388902	124	-
ENSP00000333595	123	ZNF285
ENSP00000409872	123	ZNF274
ENSP00000312029	123	UCP2
ENSP00000338413	123	UBA1
ENSP00000320239	123	TRPM7
ENSP00000296736	123	TIGD6
ENSP00000362332	123	TFAP2E
ENSP00000391372	123	SH3GL3
ENSP00000350878	123	S1PR3
ENSP00000360687	123	PTGDS
ENSP00000417963	123	PPP2R5D
ENSP00000354218	123	PPP1R13L
ENSP00000265192	123	PAIP2
ENSP00000380252	123	NFE2L2
ENSP00000286614	123	MMP16
ENSP00000323527	123	MKLN1
ENSP00000357731	123	LOR
ENSP00000296181	123	ITGB5
ENSP00000262457	123	INVS
ENSP00000385586	123	HOXD12
ENSP00000309439	123	HOXB9

ENSP00000221419	123	HNRNPL
ENSP00000370316	123	GPR143
ENSP00000372901	123	DDAH2
ENSP00000375067	123	BCAS3
ENSP00000369647	123	AVP
ENSP00000269260	123	ARRB2
ENSP00000305988	123	ALCAM
ENSP00000359489	123	AFF2
ENSP00000323036	123	ACPP
ENSP00000312246	123	-
ENSP00000240499	122	ZNF141
ENSP00000352802	122	WIPF1
ENSP00000315644	122	TYMS
ENSP00000328203	122	TRAIP
ENSP00000262395	122	TRAF4
ENSP00000301634	122	TK1
ENSP00000327315	122	TIAM2
ENSP00000319756	122	TENC1
ENSP00000261531	122	SNW1
ENSP00000337332	122	SIRT6
ENSP00000383256	122	SIAH3
ENSP00000254719	122	RPA1
ENSP00000311360	122	RAD9A
ENSP00000267868	122	RAD51
ENSP00000370151	122	RAD17
ENSP00000362283	122	PTPRT
ENSP00000371419	122	PARP4
ENSP00000252891	122	NUMBL
ENSP00000300659	122	NFATC3
ENSP00000346689	122	LMO3
ENSP00000167588	122	KRT20
ENSP00000337432	122	IL17F
ENSP00000420168	122	GSTA2
ENSP00000237837	122	FGF23
ENSP00000333640	122	EYA2
ENSP00000253861	122	EXOC4
ENSP00000386171	122	ESRRG
ENSP00000309953	122	EFEMP2
ENSP00000353475	122	CLDN7
ENSP00000362613	122	CDX4
ENSP00000209728	122	CDC6
ENSP00000276014	122	CCNB3
ENSP00000279247	122	CAPN1
ENSP00000368748	122	-
ENSP00000306983	121	YEATS2
ENSP00000402084	121	WEE1

ENSP00000322802	121	TSPYL5
ENSP00000258449	121	TGFBRAP1
ENSP00000385461	121	TFDP3
ENSP00000263663	121	TAF1B
ENSP00000222812	121	STX1A
ENSP00000265361	121	SEMA3C
ENSP00000377793	121	PRC1
ENSP00000303500	121	PAH
ENSP00000290705	121	MT1A
ENSP00000353634	121	MPZ
ENSP00000261366	121	LMNB1
ENSP00000400365	121	LAMA2
ENSP00000327890	121	IL3RA
ENSP00000356713	121	IFNGR1
ENSP00000371138	121	FKBP1A
ENSP00000229758	121	FBXO5
ENSP00000355809	121	ENAH
ENSP00000266481	121	DNM1L
ENSP00000321348	121	DIAPH2
ENSP00000286234	121	DEPTOR
ENSP00000280527	121	CRIM1
ENSP00000346340	121	COPS8
ENSP00000309132	121	BCL2L14
ENSP00000262095	121	-
ENSP00000373698	121	-
ENSP00000314153	120	ZBTB33
ENSP00000351446	120	WDR5
ENSP00000329093	120	TPH2
ENSP00000321703	120	TACC1
ENSP00000265087	120	STC2
ENSP00000310301	120	SP3
ENSP00000370083	120	SMN1
ENSP00000254325	120	RFX1
ENSP00000405963	120	RASGRF1
ENSP00000357442	120	PYGO2
ENSP00000349541	120	PTRF
ENSP00000276594	120	PRDM14
ENSP00000291906	120	PKN3
ENSP00000355014	120	PJA1
ENSP00000384515	120	PARVB
ENSP00000325819	120	NR2F1
ENSP00000366347	120	NKX2-2
ENSP00000364595	120	NINJ1
ENSP00000361718	120	NGFRAP1
ENSP00000349977	120	MVP
ENSP00000310596	120	LSM1

ENSP00000309757	120	LPL
ENSP00000230538	120	LAMA4
ENSP00000420419	120	JAM2
ENSP00000287497	120	ITGAM
ENSP00000355988	120	IRF6
ENSP00000318085	120	HINFP
ENSP00000331201	120	HGS
ENSP00000230990	120	HBEGF
ENSP00000310771	120	GRB7
ENSP00000343676	120	GJA4
ENSP00000383558	120	GCGR
ENSP00000348089	120	ERCC6
ENSP00000315011	120	EDNRA
ENSP00000288943	120	DUSP2
ENSP00000216862	120	CYP24A1
ENSP00000390948	120	CYFIP2
ENSP00000299543	120	CTDP1
ENSP00000356399	120	CFH
ENSP00000296154	120	CASR
ENSP00000244769	120	ATXN1
ENSP00000351926	120	AP2A1
ENSP00000356774	120	AHI1
ENSP00000336630	120	ADORA2A
ENSP00000278175	120	ADM
ENSP00000377941	120	ACTN1
ENSP00000212369	120	-
ENSP00000263528	120	-
ENSP00000312050	120	-
ENSP00000396920	120	-
ENSP00000347733	119	TRRAP
ENSP00000217233	119	TRIB3
ENSP00000361892	119	STK4
ENSP00000414750	119	SKOR2
ENSP00000316152	119	SFTPC
ENSP00000262039	119	PIK3C3
ENSP00000263246	119	PACSIN2
ENSP00000338283	119	NPAS2
ENSP00000300762	119	MMP26
ENSP00000300057	119	MESP1
ENSP00000363046	119	IPMK
ENSP00000347184	119	HTT
ENSP00000302548	119	HOXD4
ENSP00000333994	119	HBB
ENSP00000254810	119	H3F3B
ENSP00000350928	119	GAD1
ENSP00000310453	119	FNIP1

ENSP00000259988	119	FGFBP1
ENSP00000322390	119	FGF13
ENSP00000263062	119	EPC1
ENSP00000256261	119	DUSP26
ENSP00000292303	119	CCR5
ENSP00000296435	119	CAMP
ENSP00000307041	119	BNC1
ENSP00000356789	119	ATP1B1
ENSP00000349076	119	ATN1
ENSP00000331944	119	AEN
ENSP00000209668	119	ADH1A
ENSP00000235290	119	-
ENSP00000404976	119	-
ENSP00000337386	118	ZFP36L1
ENSP00000396211	118	WASF2
ENSP00000361852	118	TMCO2
ENSP00000379612	118	TAX1BP1
ENSP00000390500	118	STK3
ENSP00000262866	118	SLA2
ENSP00000304051	118	RNF139
ENSP00000365946	118	RBM3
ENSP00000418070	118	PVRL3
ENSP00000349932	118	PTPRS
ENSP00000335084	118	PPP1CC
ENSP00000355001	118	POU3F3
ENSP00000299206	118	POLL
ENSP00000320171	118	PKM
ENSP00000319814	118	PCK1
ENSP00000244458	118	PACSIN1
ENSP00000295886	118	NKX6-1
ENSP00000363970	118	MAP1LC3A
ENSP00000301067	118	KMT2D
ENSP00000366330	118	KLF9
ENSP00000405934	118	ITPR1
ENSP00000358310	118	ITGA10
ENSP00000331608	118	IRX3
ENSP00000280357	118	IL18
ENSP00000363827	118	HSPG2
ENSP00000347178	118	HSPB9
ENSP00000263915	118	GRB14
ENSP00000314499	118	GAK
ENSP00000359353	118	GABRE
ENSP00000346886	118	GABPA
ENSP00000358309	118	EPHA7
ENSP00000347717	118	DHCR7
ENSP00000357103	118	DARC

ENSP00000400088	118	CDK3
ENSP00000250092	118	CD68
ENSP00000357858	118	BUB3
ENSP00000357927	118	BNIPL
ENSP00000359866	118	BMP5
ENSP00000280772	118	ANK3
ENSP00000367432	118	AJAP1
ENSP00000316109	118	AGGF1
ENSP00000323172	118	-
ENSP00000348816	118	-
ENSP00000396679	118	-
ENSP00000302855	117	ZNF280A
ENSP00000307491	117	WDR48
ENSP00000412045	117	TXNRD1
ENSP00000362994	117	TRAF1
ENSP00000357306	117	RIT1
ENSP00000404306	117	PRNT
ENSP00000234310	117	PPP3R1
ENSP00000246151	117	PITHD1
ENSP00000270474	117	PDE4A
ENSP00000343376	117	OSGIN1
ENSP00000268533	117	NUDT7
ENSP00000307706	117	MT1E
ENSP00000349085	117	MAGEA1
ENSP00000356143	117	LRRN2
ENSP00000366271	117	KDM2B
ENSP00000264538	117	IFT57
ENSP00000360891	117	IFIT2
ENSP00000343552	117	ICMT
ENSP00000355870	117	HLX
ENSP00000357189	117	HDGF
ENSP00000299267	117	GABRB3
ENSP00000303423	117	FNTA
ENSP00000270221	117	EMP3
ENSP00000304290	117	CHRNB1
ENSP00000283006	117	CENPH
ENSP00000289746	117	CDH15
ENSP00000304236	117	CD14
ENSP00000231668	117	BNIP1
ENSP00000296511	117	ANXA5
ENSP00000349957	117	ANAPC11
ENSP00000341785	117	ADIPOR1
ENSP00000324892	117	-
ENSP00000403918	117	-
ENSP00000335434	116	WDR20
ENSP00000340913	116	TRPC6

ENSP00000365735	116	TLE4
ENSP00000262715	116	TEP1
ENSP00000290894	116	SHF
ENSP00000278070	116	PPRC1
ENSP00000216780	116	PCK2
ENSP00000365663	116	NPPA
ENSP00000262244	116	MOB3B
ENSP00000289893	116	MACF1
ENSP00000337354	116	LIPA
ENSP00000215539	116	IGFALS
ENSP00000390158	116	HERC1
ENSP00000339912	116	FLRT3
ENSP00000215530	116	FGF22
ENSP00000404658	116	EZH1
ENSP00000332604	116	EIF3C
ENSP00000369127	116	DNAJA1
ENSP00000376776	116	DBH
ENSP00000396876	116	DAXX
ENSP00000216492	116	CHGA
ENSP00000266557	116	CD27
ENSP00000280200	116	CD226
ENSP00000354826	116	CALD1
ENSP00000299694	116	BEAN1
ENSP00000329212	116	ATF7
ENSP00000252699	116	ACTN4
ENSP00000228217	116	-
ENSP00000219593	115	ZP2
ENSP00000353847	115	WWTR1
ENSP00000352425	115	WASF1
ENSP00000206765	115	TGM1
ENSP00000308022	115	TADA2B
ENSP00000276449	115	STAR
ENSP00000355930	115	SLC22A1
ENSP00000265113	115	SLC1A3
ENSP00000301068	115	RHEBL1
ENSP00000055077	115	RFC2
ENSP00000338629	115	RERE
ENSP00000310244	115	RASGRP1
ENSP00000306900	115	PRND
ENSP00000362824	115	OGT
ENSP00000062104	115	NNAT
ENSP00000363163	115	NLGN3
ENSP00000353219	115	NFIX
ENSP00000258341	115	LAMC1
ENSP00000324532	115	LAMA3
ENSP00000251643	115	KRT12

ENSP00000256458	115	IRAK2
ENSP00000296870	115	IL3
ENSP00000254963	115	HSPA12B
ENSP00000249499	115	HOXD9
ENSP00000222718	115	HOXA2
ENSP00000320176	115	HCLS1
ENSP00000306991	115	HAS2
ENSP00000261386	115	GDE1
ENSP00000240100	115	DUSP4
ENSP00000358081	115	BAG3
ENSP00000227667	115	APOC3
ENSP00000301945	115	ANTXR1
ENSP00000363965	115	ALPL
ENSP00000272065	115	ACP1
ENSP00000225402	115	AATF
ENSP00000323929	115	A2M
ENSP00000365514	114	ZIC2
ENSP00000318222	114	ZDHHC22
ENSP00000265333	114	VDAC1
ENSP00000367030	114	TRDMT1
ENSP00000281834	114	TNFSF4
ENSP00000270560	114	TM4SF5
ENSP00000075503	114	STYK1
ENSP00000346537	114	SMOC2
ENSP00000260653	114	SIX3
ENSP00000341024	114	SATB1
ENSP00000353030	114	PTPRF
ENSP00000246794	114	PRRG2
ENSP00000262746	114	PRDX1
ENSP00000238994	114	PPP1R3C
ENSP00000419425	114	PPIA
ENSP00000299492	114	PPFIBP2
ENSP00000310006	114	NR1D2
ENSP00000312436	114	NFE2
ENSP00000010404	114	MGST1
ENSP00000235310	114	MAD2L2
ENSP00000264144	114	LAMC2
ENSP00000262186	114	KCNH2
ENSP00000357753	114	IVL
ENSP00000321724	114	INSL3
ENSP00000298556	114	HPRT1
ENSP00000365012	114	HCK
ENSP00000263269	114	GRIN2D
ENSP00000264039	114	GPC1
ENSP00000309539	114	DPYSL2
ENSP00000381657	114	DOT1L

ENSP00000001146	114	CYP26B1
ENSP00000255641	114	CSNK1G2
ENSP00000321260	114	COX8A
ENSP00000379866	114	COL4A4
ENSP00000357013	114	CD244
ENSP00000216029	114	CBY1
ENSP00000353362	114	CACNA1A
ENSP00000290390	114	C2orf81
ENSP00000260502	114	BCAR3
ENSP00000312457	114	BAALC
ENSP00000396771	114	ANKRD6
ENSP00000356975	114	ADAMTS4
ENSP00000380436	114	-
ENSP00000200457	113	TRIP6
ENSP00000343505	113	TNFSF13
ENSP00000359013	113	TNFRSF6B
ENSP00000296795	113	TLR3
ENSP00000350877	113	SRSF2
ENSP00000280098	113	SPOPL
ENSP00000240123	113	SORBS3
ENSP00000296215	113	SNIP1
ENSP00000258052	113	SMPD2
ENSP00000221566	113	SGTA
ENSP00000356953	113	SDHC
ENSP00000332706	113	PURA
ENSP00000234179	113	PRKD3
ENSP00000263431	113	PRKCG
ENSP00000335333	113	PIP5K1C
ENSP00000365462	113	PCCA
ENSP00000356959	113	NR1I3
ENSP00000307549	113	NPTX1
ENSP00000351894	113	NCOA6
ENSP00000362127	113	MTF1
ENSP00000216122	113	MCM5
ENSP00000367787	113	LIG3
ENSP00000300035	113	KIAA0101
ENSP00000286627	113	KCNMA1
ENSP00000341108	113	HMX2
ENSP00000257868	113	GDF11
ENSP00000357393	113	EFNA3
ENSP00000267169	113	DIABLO
ENSP00000324464	113	CSNK1D
ENSP00000078445	113	CREB3L3
ENSP00000369075	113	CENPB
ENSP00000225831	113	CCL2
ENSP00000381590	113	ATXN7

ENSP00000233242	113	APOB
ENSP00000361626	112	YBX1
ENSP00000357804	112	THEM4
ENSP00000357362	112	THBS3
ENSP00000405574	112	TBL1XR1
ENSP00000348685	112	SH3BP2
ENSP00000369757	112	RPS6
ENSP00000317872	112	RBBP6
ENSP00000231572	112	RARS
ENSP00000298838	112	PACSIN3
ENSP00000350815	112	NR3C2
ENSP00000233154	112	NCK2
ENSP00000367024	112	NAA40
ENSP00000337816	112	MMP25
ENSP00000361014	112	MKNK1
ENSP00000333552	112	MEIS3
ENSP00000300231	112	MAP1A
ENSP00000303634	112	LRP8
ENSP00000284262	112	JPH3
ENSP00000352575	112	INPP5D
ENSP00000358727	112	GSTO1
ENSP00000343027	112	GNAI1
ENSP00000310447	112	GLS2
ENSP00000333193	112	GJC1
ENSP00000271450	112	FCGR2A
ENSP00000244869	112	EREG
ENSP00000353608	112	DSC3
ENSP00000361705	112	DNTTIP1
ENSP00000225719	112	CPD
ENSP00000348695	112	COL13A1
ENSP00000363596	112	CNR2
ENSP00000358511	112	CNR1
ENSP00000164024	112	CELSR3
ENSP00000354566	112	CD3E
ENSP00000320866	112	CALR
ENSP00000263119	112	CABIN1
ENSP00000263610	112	BARHL1
ENSP00000286800	112	BACH1
ENSP00000417764	112	ALG2
ENSP00000332256	112	ALDH1A3
ENSP00000360277	112	-
ENSP00000283943	111	TRIP12
ENSP00000262213	111	TRAM1
ENSP00000342098	111	SPINT1
ENSP00000267064	111	SMARCC2
ENSP00000224140	111	SETX

ENSP00000350633	111	SERTAD1
ENSP00000260926	111	SATB2
ENSP00000232217	111	RBP2
ENSP00000264399	111	PRKG2
ENSP00000375080	111	PPP2R3B
ENSP00000257905	111	PPP1R1A
ENSP00000373992	111	POU2F2
ENSP00000333142	111	PLA2G6
ENSP00000365757	111	PIP4K2A
ENSP00000275034	111	PHIP
ENSP00000242210	111	NT5C3A
ENSP00000408020	111	NEK1
ENSP00000333982	111	NDEL1
ENSP00000292431	111	NACC1
ENSP00000349640	111	MCRS1
ENSP00000326604	111	LMBR1
ENSP00000282588	111	ITGA1
ENSP00000377218	111	IRF2
ENSP00000302836	111	HOXC9
ENSP00000334382	111	HOMER1
ENSP00000362314	111	EIF4EBP2
ENSP00000296218	111	DNALI1
ENSP00000384109	111	CUL4B
ENSP00000331381	111	ANKS1B
ENSP00000301455	111	ANGPTL4
ENSP00000273628	111	-
ENSP00000297345	111	-
ENSP00000418294	111	-
ENSP00000238081	110	YWHAQ
ENSP00000365891	110	WAS
ENSP00000342381	110	VRK2
ENSP00000267996	110	TPM1
ENSP00000339730	110	THBS4
ENSP00000327959	110	TGIF1
ENSP00000354588	110	TEAD1
ENSP00000348753	110	SPRED2
ENSP00000391735	110	SLC39A7
ENSP00000284637	110	SH3RF1
ENSP00000366607	110	SF1
ENSP00000352608	110	RYR1
ENSP00000355927	110	RPS6KC1
ENSP00000359345	110	RPL5
ENSP00000363676	110	RPL11
ENSP00000283195	110	RANBP2
ENSP00000263125	110	PRKCQ
ENSP00000303057	110	PPIC

ENSP00000219479	110	NME4
ENSP00000383199	110	NEDD4L
ENSP00000345270	110	MGAT3
ENSP00000251871	110	MED17
ENSP00000299601	110	LEO1
ENSP00000261438	110	KLF3
ENSP00000380073	110	IRF9
ENSP00000375035	110	IGHV5-51
ENSP00000352980	110	HIST1H4A
ENSP00000244573	110	HIST1H1A
ENSP00000355778	110	H3F3A
ENSP00000327417	110	GPR39
ENSP00000320180	110	GHRHR
ENSP00000273920	110	ENOPH1
ENSP00000352162	110	ELAVL3
ENSP00000365272	110	DLG2
ENSP00000351665	110	CLIP1
ENSP00000228434	110	CD69
ENSP00000374529	110	CCNK
ENSP00000377141	110	ARRB1
ENSP00000260408	110	ADAM10
ENSP00000342470	110	-
ENSP00000334186	109	VWCE
ENSP00000277575	109	USP6NL
ENSP00000365048	109	TNFSF13B
ENSP00000398698	109	TNF
ENSP00000262306	109	TCEB2
ENSP00000331791	109	TBX1
ENSP00000261439	109	TBC1D1
ENSP00000403636	109	SYNGAP1
ENSP00000366746	109	STAM
ENSP00000363149	109	SPDEF
ENSP00000345161	109	SFTPB
ENSP00000370115	109	SERPINB1
ENSP00000359910	109	PSMA7
ENSP00000355145	109	PRMT6
ENSP00000347906	109	PRMT2
ENSP00000407181	109	PPP1R10
ENSP00000262300	109	PKMYT1
ENSP00000007708	109	PDK2
ENSP00000326519	109	PAGR1
ENSP00000303147	109	MAT2A
ENSP00000255681	109	MACROD1
ENSP00000252244	109	KRT1
ENSP00000379441	109	JMY
ENSP00000299106	109	JAM3

ENSP00000216341	109	GZMB
ENSP00000226413	109	GNRHR
ENSP00000273854	109	EPHA5
ENSP00000419687	109	EGR4
ENSP00000406463	109	DNAJC7
ENSP00000396308	109	DHFR
ENSP00000314299	109	CFHR1
ENSP00000363836	109	CCNY
ENSP00000300900	109	CA4
ENSP00000254900	109	BRD8
ENSP00000359371	109	BHLHE23
ENSP00000343002	109	B3GALTL
ENSP00000297785	109	ALDH1A1
ENSP00000202788	109	-
ENSP00000341409	109	-
ENSP00000348838	108	UBE2C
ENSP00000301071	108	TUBA1A
ENSP00000404817	108	TRGC1
ENSP00000370034	108	TLR7
ENSP00000275764	108	STRA8
ENSP00000300061	108	SCNN1G
ENSP00000300738	108	RRM1
ENSP00000310406	108	RIN1
ENSP00000286364	108	RASA2
ENSP00000334928	108	PTPRB
ENSP00000369756	108	PTPRA
ENSP00000356000	108	PLXNA2
ENSP00000332116	108	PDE4B
ENSP00000356191	108	OPTC
ENSP00000331066	108	OLIG1
ENSP00000315442	108	NR1H4
ENSP00000303580	108	NKIRAS2
ENSP00000222553	108	NAMPT
ENSP00000228938	108	MGP
ENSP00000348273	108	MBP
ENSP00000369349	108	KRT81
ENSP00000349477	108	KLRG1
ENSP00000260386	108	ITPKA
ENSP00000378295	108	INCENP
ENSP00000263379	108	IL27RA
ENSP00000302935	108	IL16
ENSP00000256216	108	HSD17B4
ENSP00000330074	108	HIST1H1B
ENSP00000296417	108	H2AFZ
ENSP00000353132	108	DMBX1
ENSP00000319635	108	CXCR2

ENSP00000366466	108	CTNNBIP1
ENSP00000305731	108	CST1
ENSP00000008527	108	CRY1
ENSP00000409275	108	COQ2
ENSP00000265689	108	CHKA
ENSP00000242872	108	CENPK
ENSP00000317214	108	CAPN6
ENSP00000296742	108	CAGE1
ENSP00000393596	108	BIRC6
ENSP00000318822	108	BID
ENSP00000316338	108	BAIAP2
ENSP00000251535	108	ALOX12
ENSP00000304501	108	ADORA2B
ENSP00000262211	108	-
ENSP00000337023	108	-
ENSP00000342447	108	-
ENSP00000381100	108	-
ENSP00000311221	107	ZMAT3
ENSP00000228251	107	YBX3
ENSP00000358470	107	VTCN1
ENSP00000302120	107	TRIM8
ENSP00000361554	107	TIE1
ENSP00000263707	107	TFCP2L1
ENSP00000321106	107	TAC1
ENSP00000330812	107	SDR42E2
ENSP00000350708	107	RAD23B
ENSP00000377047	107	PTPRZ1
ENSP00000335083	107	PPP2R2C
ENSP00000355031	107	PER3
ENSP00000261402	107	NUAK1
ENSP00000301411	107	NTF4
ENSP00000242152	107	NPY
ENSP00000304207	107	MUC4
ENSP00000331310	107	MORF4L1
ENSP00000236826	107	MMP8
ENSP00000282892	107	MED21
ENSP00000273261	107	LRIG1
ENSP00000356357	107	LHX9
ENSP00000392204	107	KLLN
ENSP00000361202	107	IRS4
ENSP00000357440	107	HSF2
ENSP00000243108	107	HOXC6
ENSP00000262441	107	GLP2R
ENSP00000287020	107	GDF6
ENSP00000252318	107	GALNT8
ENSP00000358866	107	FLNA

ENSP00000326819	107	FANCB
ENSP00000263026	107	EEF2K
ENSP00000240306	107	DLX4
ENSP00000382373	107	DAZL
ENSP00000381876	107	DAAM2
ENSP00000327647	107	CRADD
ENSP00000377914	107	CIAPIN1
ENSP00000358262	107	CHD1L
ENSP00000336687	107	CBX3
ENSP00000367359	107	C6orf62
ENSP00000267859	107	BNIP2
ENSP00000371897	107	ARHGAP5
ENSP00000295463	107	ALPI
ENSP00000172196	107	-
ENSP00000346253	107	-
ENSP00000337159	106	ZFYVE16
ENSP00000354219	106	TPM2
ENSP00000340477	106	TMEM179
ENSP00000261600	106	THOC1
ENSP00000264998	106	TF
ENSP00000386170	106	TBX20
ENSP00000269142	106	TAF4B
ENSP00000282223	106	SPOCK1
ENSP00000355136	106	SORBS1
ENSP00000354107	106	SLC6A6
ENSP00000351352	106	SIPA1L1
ENSP00000263593	106	SIAE
ENSP00000354045	106	RRBP1
ENSP00000341483	106	RANBP3
ENSP00000265717	106	PRKAR2B
ENSP00000285848	106	OXA1L
ENSP00000360530	106	NSMF
ENSP00000250495	106	NEDD8
ENSP00000274813	106	MUT
ENSP00000265056	106	MCM2
ENSP00000370710	106	KDM4C
ENSP00000225916	106	KAT2A
ENSP00000418357	106	IGKV1-17
ENSP00000249440	106	HOXD3
ENSP00000272937	106	HES6
ENSP00000225614	106	GALK1
ENSP00000366482	106	FXN
ENSP00000262177	106	DNAJB6
ENSP00000380033	106	DDX17
ENSP00000354609	106	CNKSR1
ENSP00000303727	106	CHRNA7

ENSP00000340896	106	ASH2L
ENSP00000262942	106	ARPC1A
ENSP00000352248	106	ANXA10
ENSP00000306163	106	ANKRD2
ENSP00000367263	106	AHNAK
ENSP00000360973	106	AGTR2
ENSP00000355627	106	AGT
ENSP00000356905	105	VNN1
ENSP00000399511	105	TNIK
ENSP00000307432	105	SUCLG2
ENSP00000410452	105	STMN1
ENSP00000364912	105	SPEN
ENSP00000263980	105	SLC9A1
ENSP00000391521	105	SLC25A36
ENSP00000365686	105	SLC15A1
ENSP00000357708	105	S100A6
ENSP00000361120	105	RALGDS
ENSP00000306390	105	PRSS27
ENSP00000196061	105	PLOD1
ENSP00000312070	105	PCDHGC3
ENSP00000349313	105	NHEJ1
ENSP00000294785	105	NCSTN
ENSP00000286317	105	MED7
ENSP00000263816	105	LRP2
ENSP00000373783	105	LOXL2
ENSP00000294638	105	LHX8
ENSP00000298480	105	KCNT1
ENSP00000264497	105	IL21
ENSP00000005558	105	IFRD1
ENSP00000360869	105	IFIT1
ENSP00000012134	105	HIVEP2
ENSP00000326579	105	HCCS
ENSP00000257934	105	ESPL1
ENSP00000296420	105	EMCN
ENSP00000369460	105	ELAVL2
ENSP00000280904	105	DSC2
ENSP00000176183	105	DRD4
ENSP00000338019	105	DNAJB2
ENSP00000349204	105	CRB3
ENSP00000334084	105	CCDC18
ENSP00000370047	105	BNC2
ENSP00000296794	105	ARHGEF28
ENSP00000302476	105	-
ENSP00000385360	105	-
ENSP00000335055	104	WASF3
ENSP00000367013	104	TNFRSF25

ENSP00000287814	104	TIMP4
ENSP00000401371	104	TIA1
ENSP00000347792	104	SYNJ2
ENSP00000358071	104	SEC23IP
ENSP00000357721	104	S100A8
ENSP00000357726	104	S100A12
ENSP00000308461	104	RND1
ENSP00000356429	104	RGS1
ENSP00000321365	104	RAD23A
ENSP00000341170	104	PTN
ENSP00000301258	104	PSCA
ENSP00000309509	104	PLAC8
ENSP00000320758	104	NOS1
ENSP00000263437	104	NLRP2
ENSP00000333539	104	MIPOL1
ENSP00000357047	104	MED23
ENSP00000349770	104	IRF5
ENSP00000355140	104	HOXB1
ENSP00000304226	104	HIPK3
ENSP00000294973	104	HAAO
ENSP00000341848	104	GOLGB1
ENSP00000331745	104	GDF3
ENSP00000371798	104	FSCN1
ENSP00000162391	104	FOXJ2
ENSP00000272167	104	EPHX1
ENSP00000352312	104	DNAH8
ENSP00000316377	104	DLGAP1
ENSP00000296414	104	DAPP1
ENSP00000222271	104	COMP
ENSP00000223398	104	CLIP2
ENSP00000349971	104	CEACAM3
ENSP00000261900	104	CCNT1
ENSP00000297156	104	CAMLG
ENSP00000312741	104	CAMKK2
ENSP00000356545	104	CACNA1E
ENSP00000319281	104	BASP1
ENSP00000345179	104	APOD
ENSP00000368766	104	ADRA1D
ENSP00000205557	104	ABCC6
ENSP00000248272	104	-
ENSP00000287968	104	-
ENSP00000397575	104	-
ENSP00000334854	103	ZACN
ENSP00000072644	103	YIPF1
ENSP00000339299	103	TRIO
ENSP00000263932	103	TNFRSF8

ENSP00000348813	103	TBC1D7
ENSP00000376024	103	SNX9
ENSP00000261024	103	SLC40A1
ENSP00000366093	103	RORB
ENSP00000373772	103	RNF20
ENSP00000260402	103	PLCB2
ENSP00000335062	103	PDCD1
ENSP00000300954	103	PCSK4
ENSP00000329295	103	NXPH3
ENSP00000302648	103	NRTN
ENSP00000261245	103	MNAT1
ENSP00000233121	103	MAPRE3
ENSP00000296509	103	MAD2L1
ENSP00000171111	103	KEAP1
ENSP00000259021	103	KAT7
ENSP00000341280	103	JARID2
ENSP00000221980	103	ICAM5
ENSP00000324884	103	HOXA3
ENSP00000261917	103	HCN4
ENSP00000248114	103	GFER
ENSP00000364277	103	FGD1
ENSP00000370330	103	ERBB2IP
ENSP00000359211	103	DPYD
ENSP00000266000	103	DAXX
ENSP00000345344	103	CTSL
ENSP00000279804	103	CTF1
ENSP00000365025	103	CSNK2B
ENSP00000260058	103	CREBZF
ENSP00000386104	103	CPE
ENSP00000308741	103	CLOCK
ENSP00000328674	103	CLDN6
ENSP00000003084	103	CFTR
ENSP00000357153	103	CD1D
ENSP00000375844	103	CAPN10
ENSP00000217169	103	BIRC7
ENSP00000357748	103	BCCIP
ENSP00000263207	103	ARVCF
ENSP00000221891	103	APLP1
ENSP00000362524	103	ANGPTL2
ENSP00000359204	103	-
ENSP00000390877	103	-
ENSP00000397964	103	-
ENSP00000347910	102	ZNF44
ENSP00000351777	102	VCP
ENSP00000355208	102	TPTE
ENSP00000368350	102	TPT1

ENSP00000370395	102	SGTB
ENSP00000298854	102	RAPSN
ENSP00000324248	102	PENK
ENSP00000309124	102	OLR1
ENSP00000262101	102	MSR1
ENSP00000298894	102	MOAP1
ENSP00000391664	102	MFRP
ENSP00000298048	102	MELK
ENSP00000383234	102	MAPK4
ENSP00000262407	102	ITGA2B
ENSP00000229134	102	IL26
ENSP00000372224	102	HGFAC
ENSP00000265755	102	GTF2IRD1
ENSP00000343633	102	FOXN2
ENSP00000306129	102	DRD5
ENSP00000357900	102	CUZD1
ENSP00000255409	102	CHI3L1
ENSP00000271332	102	CELSR2
ENSP00000383611	102	CCNL2
ENSP00000364260	102	C9orf156
ENSP00000363512	102	ALOX5
ENSP00000251757	102	-
ENSP00000382267	102	-
ENSP00000246957	101	TRAP1
ENSP00000396704	101	TOP2B
ENSP00000325748	101	STK25
ENSP00000240327	101	SPOP
ENSP00000270632	101	SPIB
ENSP00000332931	101	SLC8A1
ENSP00000374240	101	SHOX2
ENSP00000292169	101	S100A1
ENSP00000259939	101	RNF144B
ENSP00000408395	101	RBFOX3
ENSP00000264515	101	RBBP5
ENSP00000357883	101	PIP5K1A
ENSP00000228841	101	MYL2
ENSP00000339086	101	MKL2
ENSP00000353375	101	MAP4
ENSP00000345708	101	KCNJ11
ENSP00000358414	101	HMGCS2
ENSP00000322706	101	HMGCS1
ENSP00000352627	101	HIST1H2AM
ENSP00000341214	101	HIST1H1T
ENSP00000358571	101	HIPK1
ENSP00000241337	101	GSTM2
ENSP00000237527	101	GHRH

ENSP00000312673	101	GH1
ENSP00000321706	101	GEMIN4
ENSP00000359356	101	EVI5
ENSP00000365103	101	DAOA
ENSP00000303939	101	CTLA4
ENSP00000330633	101	CNTN2
ENSP00000353199	101	CMTM3
ENSP00000299752	101	CDH16
ENSP00000313172	101	BVES
ENSP00000253354	101	BPIFB1
ENSP00000361813	101	BEX1
ENSP00000358105	101	APH1A
ENSP00000263200	101	-
ENSP00000397527	101	-
ENSP00000297857	100	ZHX1
ENSP00000356602	100	VTA1
ENSP00000238721	100	TP53I3
ENSP00000220616	100	TG
ENSP00000338127	100	TESK1
ENSP00000352926	100	TEAD4
ENSP00000299328	100	TAZ
ENSP00000278499	100	SESN3
ENSP00000369318	100	RHOBTB3
ENSP00000369823	100	RGL3
ENSP00000263088	100	PLD2
ENSP00000221480	100	PEX11G
ENSP00000159060	100	NOX3
ENSP00000311997	100	NEFH
ENSP00000385045	100	MTA3
ENSP00000358547	100	MPP1
ENSP00000347427	100	MINK1
ENSP00000265026	100	MAP3K13
ENSP00000252999	100	LAMA5
ENSP00000381216	100	KHSRP
ENSP00000307006	100	IRX2
ENSP00000258774	100	HUS1
ENSP00000367034	100	HIST1H4C
ENSP00000303408	100	HIST1H2BO
ENSP00000342886	100	HIST1H2BJ
ENSP00000274764	100	HIST1H2BA
ENSP00000297012	100	HIST1H2AA
ENSP00000307705	100	HIST1H1E
ENSP00000224605	100	GDF10
ENSP00000347041	100	FMOD
ENSP00000305480	100	FEN1
ENSP00000379042	100	ERO1L

ENSP00000290246	100	ELMO2
ENSP00000332148	100	DZIP1L
ENSP00000360316	100	DHCR24
ENSP00000354830	100	DDIT4L
ENSP00000359151	100	DBT
ENSP00000301645	100	CYP7A1
ENSP00000353706	100	CDC42SE2
ENSP00000242786	100	CD97
ENSP00000229304	100	APOBEC1
ENSP00000216714	100	APEX1
ENSP00000261981	100	-
ENSP00000339344	100	-
ENSP00000390617	100	-
ENSP00000303252	99	ZNF804A
ENSP00000306330	99	YWHAG
ENSP00000283628	99	UBP1
ENSP00000332326	99	TNFAIP2
ENSP00000322234	99	SYNJ1
ENSP00000330659	99	SOCS7
ENSP00000270349	99	SLC6A3
ENSP00000331736	99	SELE
ENSP00000228916	99	SCNN1A
ENSP00000315212	99	RNF4
ENSP00000388287	99	REPIN1
ENSP00000334941	99	PTPRU
ENSP00000330276	99	PTPN21
ENSP00000319169	99	PRMT5
ENSP00000361009	99	PREX1
ENSP00000333905	99	PPP2R5C
ENSP00000361275	99	PLK3
ENSP00000323856	99	PLEC
ENSP00000367301	99	NDP
ENSP00000356505	99	NCF2
ENSP00000357643	99	MKI67
ENSP00000337691	99	MAPK15
ENSP00000353508	99	MAP2
ENSP00000374990	99	IGHG1
ENSP00000374989	99	IGHA1
ENSP00000358963	99	HTR1B
ENSP00000355656	99	HIST3H2A
ENSP00000358158	99	HIST2H2AA3
ENSP00000328773	99	HEXIM1
ENSP00000345023	99	GSTO2
ENSP00000397026	99	GRIK2
ENSP00000354313	99	GRAMD4
ENSP00000344782	99	GFI1B

ENSP00000379457	99	FAF1
ENSP00000247270	99	EVI2A
ENSP00000361725	99	ENDOG
ENSP00000397323	99	EHMT2
ENSP00000417052	99	EBP
ENSP00000322180	99	DSCC1
ENSP00000361467	99	DLG5
ENSP00000324549	99	CYFIP1
ENSP00000332602	99	CTAG1B
ENSP00000336571	99	CLDN2
ENSP00000245907	99	C3
ENSP00000330813	99	BRAP
ENSP00000412957	99	BIRC8
ENSP00000324510	99	BAIAP3
ENSP00000359727	99	BAG2
ENSP00000261879	99	APH1B
ENSP00000340261	99	-
ENSP00000402094	99	-
ENSP00000322915	98	ZMYM4
ENSP00000249330	98	VGF
ENSP00000332668	98	TSKU
ENSP00000326737	98	TNFRSF12A
ENSP00000246080	98	TCF15
ENSP00000350198	98	SSTR2
ENSP00000315713	98	SSH1
ENSP00000262901	98	SIM1
ENSP00000333537	98	SALL2
ENSP00000221975	98	RPS19
ENSP00000354951	98	QKI
ENSP00000358925	98	PSRC1
ENSP00000404676	98	PGGT1B
ENSP00000216727	98	PABPN1
ENSP00000272907	98	NYAP2
ENSP00000396538	98	NFAT5
ENSP00000205890	98	MYO15A
ENSP00000233545	98	MPV17
ENSP00000376827	98	MKS1
ENSP00000366477	98	MALRD1
ENSP00000218439	98	MAGED2
ENSP00000252252	98	KRT6B
ENSP00000385722	98	INTS1
ENSP00000270139	98	IFNAR1
ENSP00000396486	98	HSPA1L
ENSP00000328598	98	HOXD1
ENSP00000341826	98	HNRNPA1
ENSP00000353074	98	HIST1H2BF

ENSP00000373730	98	H2AFJ
ENSP00000359795	98	GIPC2
ENSP00000291744	98	FCN2
ENSP00000312185	98	ELMO1
ENSP00000257312	98	DZIP1
ENSP00000371451	98	DNAJC21
ENSP00000006053	98	CX3CL1
ENSP00000281282	98	CGNL1
ENSP00000284000	98	CEBPG
ENSP00000219789	98	CDIPT
ENSP00000329482	98	CCSER1
ENSP00000295926	98	CCNL1
ENSP00000293272	98	CCL5
ENSP00000368767	98	ASPH
ENSP00000315325	98	ARHGEF2
ENSP00000293761	98	ALOX15
ENSP00000284987	98	ADAMTS5
ENSP00000290866	98	ACE
ENSP00000301886	98	-
ENSP00000391797	98	-
ENSP00000380039	97	ZSCAN12
ENSP00000300161	97	YWHAB
ENSP00000356515	97	UTRN
ENSP00000265339	97	UBE2B
ENSP00000362013	97	TRERF1
ENSP00000293826	97	TNFSF12-TNFSF13
ENSP00000406888	97	TCOF1
ENSP00000314491	97	SRRT
ENSP00000222584	97	SP4
ENSP00000317382	97	SLC36A4
ENSP00000396523	97	SLC35G6
ENSP00000002829	97	SEMA3F
ENSP00000321239	97	RCHY1
ENSP00000252483	97	PVRL2
ENSP00000295927	97	PTX3
ENSP00000312286	97	PLA2G1B
ENSP00000352572	97	PCNT
ENSP00000314067	97	PAK2
ENSP00000244766	97	NRN1
ENSP00000295440	97	NPPC
ENSP00000355133	97	NOS1AP
ENSP00000269243	97	MYH10
ENSP00000226207	97	MYH1
ENSP00000229854	97	MCM3
ENSP00000392466	97	LDB1
ENSP00000347088	97	LARGE

ENSP00000307156	97	LAMB2
ENSP00000290200	97	IL10RB
ENSP00000244661	97	HIST1H3B
ENSP00000321389	97	HIST1H2AC
ENSP00000215780	97	GSTT2
ENSP00000230056	97	GMNN
ENSP00000246949	97	DNASE1
ENSP00000305777	97	CSNK1G1
ENSP00000389951	97	CELF2
ENSP00000303788	97	CDKN2AIP
ENSP00000324101	97	CD151
ENSP00000381412	97	CAMK2A
ENSP00000265838	97	ACAT1
ENSP00000305538	97	-
ENSP00000336543	97	-
ENSP00000338190	97	-
ENSP00000369582	97	-
ENSP00000393405	97	-
ENSP00000409572	97	-
ENSP00000363641	96	TXN
ENSP00000251413	96	TUBG1
ENSP00000261464	96	TRAF5
ENSP00000075120	96	SLC2A3
ENSP00000272542	96	SLC20A1
ENSP00000366936	96	SHB
ENSP00000261991	96	RPS6KA5
ENSP00000280362	96	PTS
ENSP00000363350	96	PTPN20B
ENSP00000271308	96	PSMA5
ENSP00000164133	96	PPP2R5B
ENSP00000312649	96	PPARGC1B
ENSP00000318165	96	OS9
ENSP00000278612	96	NPAT
ENSP00000380450	96	NOXO1
ENSP00000358994	96	MYO6
ENSP00000356859	96	MGST3
ENSP00000285879	96	MAGEC1
ENSP00000375857	96	LRRFIP1
ENSP00000290158	96	KPNB1
ENSP00000170630	96	IL4R
ENSP00000247207	96	HSPA2
ENSP00000358211	96	HSPA12A
ENSP00000350549	96	HMX3
ENSP00000348258	96	HIST1H4L
ENSP00000348924	96	HIST1H2BE
ENSP00000244534	96	HIST1H1D

ENSP00000339566	96	HIST1H1C
ENSP00000314774	96	HES7
ENSP00000264350	96	HERC5
ENSP00000264345	96	HERC3
ENSP00000327431	96	HBG1
ENSP00000346450	96	H2AFB1
ENSP00000367872	96	GNB1
ENSP00000272224	96	GDF7
ENSP00000359258	96	GCLM
ENSP00000331544	96	FBLN1
ENSP00000340660	96	ELSPBP1
ENSP00000404672	96	DCDC1
ENSP00000312435	96	DAG1
ENSP00000392028	96	CHD7
ENSP00000319060	96	CAMK2G
ENSP00000187397	96	ARPP21
ENSP00000262215	96	ARFGEF1
ENSP00000342952	96	ADCY2
ENSP00000221204	96	-
ENSP00000411560	96	-
ENSP00000311778	95	ZMIZ2
ENSP00000316176	95	UBE2N
ENSP00000410071	95	TUBB
ENSP00000312672	95	TMEM136
ENSP00000356795	95	TBX19
ENSP00000335191	95	TBX10
ENSP00000296946	95	T
ENSP00000302978	95	SPRY3
ENSP00000309945	95	SPNS1
ENSP00000408792	95	SP8
ENSP00000366988	95	SNX5
ENSP00000369638	95	SNX1
ENSP00000370119	95	SMN2
ENSP00000323967	95	SMARCE1
ENSP00000222248	95	SLC5A5
ENSP00000357692	95	S100A16
ENSP00000342755	95	RNF41
ENSP00000221515	95	RETN
ENSP00000266671	95	PHLDA1
ENSP00000302814	95	OTP
ENSP00000361214	95	NRG3
ENSP00000269280	95	NLRP1
ENSP00000350467	95	NAT9
ENSP00000381599	95	MX1
ENSP00000245615	95	MBOAT7
ENSP00000367309	95	MAOB

ENSP00000354912	95	MAGEE1
ENSP00000366418	95	LZIC
ENSP00000339916	95	LIMK2
ENSP00000367891	95	LGALS7
ENSP00000222399	95	LAMB1
ENSP00000364864	95	KIF3B
ENSP00000312631	95	INSM1
ENSP00000303231	95	IL12A
ENSP00000367959	95	HTR2A
ENSP00000352252	95	HIST1H3J
ENSP00000053867	95	GRN
ENSP00000308137	95	FOLR1
ENSP00000356946	95	FCGR3A
ENSP00000262464	95	FBN2
ENSP00000256383	95	EIF2S1
ENSP00000305651	95	CXCL10
ENSP00000265372	95	CREM
ENSP00000271889	95	CREB3L4
ENSP00000355180	95	COL6A1
ENSP00000181839	95	CDK13
ENSP00000355361	95	CD47
ENSP00000292144	95	CD3G
ENSP00000380349	95	CAPN3
ENSP00000261714	95	BLMH
ENSP00000310561	95	ARHGAP32
ENSP00000312370	95	AIFM2
ENSP00000404416	95	ADTRP
ENSP00000299351	95	-
ENSP00000334474	94	ZMIZ1
ENSP00000354877	94	ULK2
ENSP00000358674	94	UBL4A
ENSP00000254942	94	TERF2
ENSP00000259075	94	TANK
ENSP00000217893	94	TAF9
ENSP00000220876	94	STMN2
ENSP00000278742	94	ST14
ENSP00000335628	94	SPDYA
ENSP00000340409	94	SMPD1
ENSP00000323421	94	SMC1A
ENSP00000233202	94	SLC11A1
ENSP00000357701	94	S100A3
ENSP00000333948	94	RPS19BP1
ENSP00000220507	94	RHOV
ENSP00000417895	94	RASA4B
ENSP00000284957	94	RABGEF1
ENSP00000394863	94	PPT1

ENSP00000404845	94	PIK3C2G
ENSP00000291442	94	NR2F6
ENSP00000264187	94	NID1
ENSP00000363205	94	NFS1
ENSP00000383210	94	NEK3
ENSP00000350639	94	MAPKAPK3
ENSP00000300417	94	LRSAM1
ENSP00000321537	94	LLGL1
ENSP00000354753	94	LDLRAD4
ENSP00000252250	94	KRT6C
ENSP00000272117	94	ITPKB
ENSP00000352119	94	HIST1H2AG
ENSP00000290765	94	GSTT2B
ENSP00000360616	94	GRIN1
ENSP00000344254	94	FXYD5
ENSP00000295633	94	FSTL1
ENSP00000309629	94	CFL1
ENSP00000351409	94	CELF1
ENSP00000296777	94	CARTPT
ENSP00000323099	94	ATRIP
ENSP00000257527	94	ADAM19
ENSP00000368351	94	-
ENSP00000406904	94	-
ENSP00000416993	94	-
ENSP00000356213	93	VIP
ENSP00000343129	93	TRIM29
ENSP00000361186	93	TP53RK
ENSP00000304277	93	TM4SF1
ENSP00000360269	93	TACSTD2
ENSP00000370299	93	SHROOM2
ENSP00000216274	93	RIPK3
ENSP00000341874	93	RIOK3
ENSP00000255006	93	RIN2
ENSP00000302846	93	PTGER4
ENSP00000349428	93	PTBP1
ENSP00000305152	93	PLP1
ENSP00000338185	93	PLCB1
ENSP00000380855	93	PDCD1LG2
ENSP00000344314	93	OFD1
ENSP00000240055	93	NFYB
ENSP00000372313	93	MSLN
ENSP00000363193	93	MED12
ENSP00000352835	93	MB
ENSP00000249363	93	LRRC4
ENSP00000263274	93	LIG1
ENSP00000268763	93	KSR1

ENSP00000297814	93	KIF27
ENSP00000344741	93	INSIG1
ENSP00000356549	93	IER5
ENSP00000292433	93	IER2
ENSP00000409151	93	HSPA1L
ENSP00000393087	93	HSPA1B
ENSP00000329554	93	HIST1H3I
ENSP00000358160	93	HIST1H3H
ENSP00000350275	93	HIST1H3A
ENSP00000380177	93	HIST1H2BN
ENSP00000348706	93	HIST1H2BH
ENSP00000244601	93	HIST1H2BG
ENSP00000334805	93	H1FNT
ENSP00000398131	93	GSPT1
ENSP00000350005	93	GIP
ENSP00000337168	93	EPB41L1
ENSP00000259486	93	ENPP2
ENSP00000317955	93	EEA1
ENSP00000328405	93	DGKA
ENSP00000221996	93	CRX
ENSP00000258807	93	CIDEB
ENSP00000250151	93	CCL4
ENSP00000340297	93	ARHGEF10
ENSP00000237283	93	ADAT2
ENSP00000349436	93	ADAM15
ENSP00000225941	93	ABI3
ENSP00000258962	92	SRSF1
ENSP00000304783	92	SLC26A5
ENSP00000357022	92	SLAMF7
ENSP00000369131	92	SIN3B
ENSP00000337163	92	SH2B1
ENSP00000385899	92	SDK1
ENSP00000019103	92	SCTR
ENSP00000357799	92	S100A10
ENSP00000262850	92	RRAGB
ENSP00000361010	92	REXO4
ENSP00000343656	92	RAPGEF5
ENSP00000310129	92	PSMD2
ENSP00000329127	92	PRKCH
ENSP00000287008	92	PCDH1
ENSP00000301905	92	PBK
ENSP00000354929	92	NOTCH2NL
ENSP00000377995	92	MT1X
ENSP00000205386	92	LAMB4
ENSP00000262419	92	KANSL1
ENSP00000384534	92	INPP5J

ENSP00000360777	92	INPP5E
ENSP00000261465	92	HSD11B1
ENSP00000375736	92	HIST3H2BB
ENSP00000358164	92	HIST2H2BF
ENSP00000332790	92	HIST2H2AB
ENSP00000358155	92	HIST2H2AA4
ENSP00000350159	92	HIST1H4K
ENSP00000347168	92	HIST1H4J
ENSP00000244537	92	HIST1H4G
ENSP00000366974	92	HIST1H4F
ENSP00000353624	92	HIST1H4E
ENSP00000343282	92	HIST1H4D
ENSP00000366581	92	HIST1H4B
ENSP00000353581	92	HIST1H3E
ENSP00000366962	92	HIST1H2BI
ENSP00000321744	92	HIST1H2BC
ENSP00000350580	92	HIST1H2BB
ENSP00000349873	92	HIST1H2AL
ENSP00000330307	92	HIST1H2AK
ENSP00000328484	92	HIST1H2AJ
ENSP00000366679	92	HIST1H2AH
ENSP00000341094	92	HIST1H2AD
ENSP00000259791	92	HIST1H2AB
ENSP00000308405	92	H2AFV
ENSP00000358452	92	H2AFB3
ENSP00000346509	92	H2AFB2
ENSP00000352047	92	GULP1
ENSP00000275364	92	GNA12
ENSP00000305692	92	GAA
ENSP00000361672	92	FOXO6
ENSP00000293829	92	FGF11
ENSP00000384169	92	FBLN2
ENSP00000305138	92	FAM195A
ENSP00000419266	92	EVX1
ENSP00000376652	92	EVL
ENSP00000239882	92	ELF1
ENSP00000266037	92	DOCK3
ENSP00000286809	92	CLDN8
ENSP00000221476	92	CKM
ENSP00000296140	92	CCR1
ENSP00000307208	92	BPTF
ENSP00000252677	92	BMP15
ENSP00000301030	92	ANKRD11
ENSP00000290649	92	AMFR
ENSP00000367086	92	ACOT7
ENSP00000230495	92	-

ENSP00000259737	92	-
ENSP00000262266	92	-
ENSP00000307051	92	-
ENSP00000313026	92	-
ENSP00000319632	92	-
ENSP00000353783	92	-
ENSP00000358175	92	-
ENSP00000354680	91	ZNF266
ENSP00000357887	91	VPS72
ENSP00000339804	91	TSLP
ENSP00000320493	91	TRIP10
ENSP00000371376	91	TLR6
ENSP00000223051	91	TFR2
ENSP00000357307	91	SYT11
ENSP00000414259	91	SYNGAP1
ENSP00000367208	91	SUGT1
ENSP00000344742	91	STAMBP
ENSP00000285013	91	SLFN13
ENSP00000281456	91	SLC25A4
ENSP00000275300	91	SLC22A3
ENSP00000344822	91	S100A13
ENSP00000272430	91	RTKN
ENSP00000339521	91	RSU1
ENSP00000349131	91	RSPO3
ENSP00000259406	91	RGS3
ENSP00000362588	91	PBX3
ENSP00000263650	91	OSBPL5
ENSP00000342848	91	NOXA1
ENSP00000387310	91	NOTUM
ENSP00000294064	91	NEU3
ENSP00000362702	91	NEK6
ENSP00000386259	91	NEB
ENSP00000334872	91	MT1F
ENSP00000355775	91	MIXL1
ENSP00000264156	91	MCM6
ENSP00000384665	91	LPAR2
ENSP00000308452	91	KRT17
ENSP00000414982	91	KLC1
ENSP00000356087	91	IKBKE
ENSP00000375004	91	IGHV1-3
ENSP00000297185	91	HSPA9
ENSP00000289352	91	HIST1H4H
ENSP00000303373	91	HIST1H2AE
ENSP00000344504	91	H1F0
ENSP00000353695	91	GSTCD
ENSP00000256857	91	GRP

ENSP00000229416	91	GCLC
ENSP00000259271	91	GAD2
ENSP00000023897	91	GABRA1
ENSP00000222157	91	FGF21
ENSP00000361669	91	ESX1
ENSP00000344235	91	DUSP28
ENSP00000252137	91	DGCR14
ENSP00000264474	91	CSTA
ENSP00000344683	91	CDK5RAP3
ENSP00000226279	91	CD38
ENSP00000386378	91	CD207
ENSP00000300213	91	CCNDBP1
ENSP00000293889	91	CCDC78
ENSP00000369855	91	ASB9
ENSP00000360762	91	ANKRD1
ENSP00000286744	91	ADAMTSL3
ENSP00000325395	91	ACADVL
ENSP00000344789	91	ACACA
ENSP00000297905	91	-
ENSP00000375533	91	-
ENSP00000381655	91	-
ENSP00000287156	90	UBE2L6
ENSP00000381717	90	UBE2D2
ENSP00000362424	90	TRAF3IP1
ENSP00000349705	90	TOP3B
ENSP00000268957	90	TOB1
ENSP00000321203	90	TNIP2
ENSP00000340089	90	TLR5
ENSP00000319233	90	TLE3
ENSP00000362979	90	TGIF2
ENSP00000263688	90	SUCO
ENSP00000365530	90	STXBP4
ENSP00000282908	90	STK32B
ENSP00000365730	90	STK24
ENSP00000341550	90	SLC24A5
ENSP00000202967	90	SIRT4
ENSP00000264027	90	SC5D
ENSP00000309163	90	RCE1
ENSP00000348632	90	RBCK1
ENSP00000011619	90	RANBP9
ENSP00000351284	90	RAD52
ENSP00000354612	90	PTGS1
ENSP00000217958	90	PSMD10
ENSP00000271657	90	PI4KB
ENSP00000367365	90	PCDHA7
ENSP00000325421	90	PARL

ENSP00000289422	90	NRG2
ENSP00000356988	90	NIT1
ENSP00000306754	90	NEUROD2
ENSP00000261797	90	NDST1
ENSP00000345789	90	MUM1
ENSP00000371888	90	MLST8
ENSP00000327054	90	LMNB2
ENSP00000367198	90	LECT1
ENSP00000356234	90	KDM5B
ENSP00000364805	90	HSPA1L
ENSP00000408347	90	HSPA1L
ENSP00000364801	90	HSPA1B
ENSP00000375391	90	HSPA1B
ENSP00000375399	90	HSPA1B
ENSP00000403530	90	HSPA1B
ENSP00000385479	90	HIST2H3A
ENSP00000366999	90	HIST1H3D
ENSP00000349430	90	HIST1H2BK
ENSP00000289316	90	HIST1H2BD
ENSP00000261458	90	HHAT
ENSP00000283871	90	HGD
ENSP00000264108	90	HAT1
ENSP00000362352	90	H2AFY2
ENSP00000370105	90	GTF2H2C
ENSP00000334952	90	GPR125
ENSP00000340200	90	GLYAT
ENSP00000382165	90	GAREM
ENSP00000359329	90	GABRQ
ENSP00000254090	90	FMO5
ENSP00000323822	90	FBXO11
ENSP00000353321	90	FAM118B
ENSP00000297258	90	FABP5
ENSP00000311873	90	EXO1
ENSP00000307843	90	EGFL7
ENSP00000255030	90	CRP
ENSP00000263645	90	CD81
ENSP00000339393	90	CCR6
ENSP00000265431	90	CALB1
ENSP00000391998	90	ARTN
ENSP00000377233	90	ARAP1
ENSP00000258530	90	APPL2
ENSP00000298861	90	AGBL2
ENSP00000225760	90	-
ENSP00000278934	90	-
ENSP00000396157	90	-
ENSP00000337724	89	ZNF202

ENSP00000233055	89	WDFY1
ENSP00000231461	89	ST8SIA4
ENSP00000352936	89	SPINK5
ENSP00000261622	89	SLC7A5
ENSP00000357038	89	SLAMF6
ENSP00000362298	89	SGPL1
ENSP00000356071	89	SERAC1
ENSP00000314029	89	SENP3
ENSP00000299333	89	SCN3B
ENSP00000369270	89	RREB1
ENSP00000393159	89	RFXANK
ENSP00000297338	89	RAD21
ENSP00000344547	89	PTMA
ENSP00000293362	89	PSME3
ENSP00000261712	89	PSMD11
ENSP00000388631	89	PLCD4
ENSP00000374409	89	PKP4
ENSP00000252603	89	PGLS
ENSP00000215904	89	PDXP
ENSP00000166534	89	P4HA2
ENSP00000347710	89	OPHN1
ENSP00000284292	89	NRGN
ENSP00000381105	89	NPS
ENSP00000319377	89	NLRP12
ENSP00000291890	89	NCR1
ENSP00000351790	89	MYPN
ENSP00000311905	89	LTBP4
ENSP00000351755	89	LPAR1
ENSP00000354851	89	KIF21A
ENSP00000313829	89	KHDRBS1
ENSP00000323659	89	KDM3A
ENSP00000260570	89	IFT172
ENSP00000253083	89	HIP1R
ENSP00000329002	89	HAX1
ENSP00000271357	89	GPR161
ENSP00000371398	89	GLIS3
ENSP00000275428	89	GGCT
ENSP00000355996	89	G0S2
ENSP00000264895	89	FRAS1
ENSP00000373952	89	FANCA
ENSP00000367992	89	ESD
ENSP00000199448	89	EPDR1
ENSP00000367851	89	CYBB
ENSP00000261726	89	CUX2
ENSP00000249923	89	COPB1
ENSP00000307508	89	CALB2

ENSP00000338814	89	BAG5
ENSP00000313950	89	AURKB
ENSP00000264245	89	ARHGAP31
ENSP00000379709	89	ARHGAP21
ENSP00000355537	89	ACTN2
ENSP00000263100	89	A1BG
ENSP00000307722	89	-
ENSP00000332740	89	-
ENSP00000337122	88	ZNF354A
ENSP00000305426	88	TUB
ENSP00000230510	88	TTK
ENSP00000295314	88	TMOD4
ENSP00000338624	88	TASP1
ENSP00000327072	88	TAF1A
ENSP00000256637	88	SORT1
ENSP00000286398	88	SMC2
ENSP00000266058	88	SLIT1
ENSP00000369816	88	SHBG
ENSP00000278282	88	SCGB1A1
ENSP00000317895	88	RPTN
ENSP00000220751	88	RIPK2
ENSP00000234038	88	PPP1R7
ENSP00000070846	88	PKP2
ENSP00000337797	88	PIDD
ENSP00000313007	88	PABPC1
ENSP00000223190	88	NRF1
ENSP00000218652	88	NDFIP2
ENSP00000252575	88	NCAN
ENSP00000381007	88	NAV3
ENSP00000299766	88	MC4R
ENSP00000354360	88	LAMC3
ENSP00000332455	88	KPNA2
ENSP00000302456	88	KLF13
ENSP00000307023	88	KLF11
ENSP00000257408	88	KLB
ENSP00000352138	88	KIRREL
ENSP00000363435	88	ITPR3
ENSP00000388526	88	HLA-A
ENSP00000298251	88	HEPACAM
ENSP00000351524	88	HELZ
ENSP00000239027	88	HELLS
ENSP00000292896	88	HBE1
ENSP00000335620	88	GSTA1
ENSP00000339186	88	GRAP2
ENSP00000406367	88	GPR124
ENSP00000378338	88	GIT1

ENSP00000263281	88	GIPR
ENSP00000281623	88	FBXO4
ENSP00000316879	88	EIF4G1
ENSP00000250498	88	DAD1
ENSP00000262982	88	CSE1L
ENSP00000381333	88	CRHR1
ENSP00000261833	88	CIT
ENSP00000306490	88	CHRM1
ENSP00000262717	88	CDH20
ENSP00000302234	88	CCL11
ENSP00000258418	88	CAB39
ENSP00000297991	88	AQP3
ENSP00000311165	88	AQP1
ENSP00000388613	88	-
ENSP00000392676	88	-
ENSP00000304985	87	ZFX
ENSP00000334490	87	ZDHHC8
ENSP00000372635	87	ZBTB38
ENSP00000234392	87	VAX2
ENSP00000302239	87	USP8
ENSP00000363095	87	TRIM32
ENSP00000368391	87	TPD52
ENSP00000331305	87	TOB2
ENSP00000363157	87	TNFSF15
ENSP00000296861	87	TNFRSF21
ENSP00000330138	87	SSTR3
ENSP00000274192	87	SRD5A1
ENSP00000221448	87	SNRNP70
ENSP00000361087	87	SNCG
ENSP00000377401	87	SLC35B2
ENSP00000009589	87	RPS20
ENSP00000242719	87	RNF11
ENSP00000262940	87	RASA4
ENSP00000356436	87	PLA2G4A
ENSP00000316809	87	PITPNA
ENSP00000388001	87	OAS1
ENSP00000263273	87	NUCB1
ENSP00000326777	87	MTHFSD
ENSP00000334003	87	INTU
ENSP00000408907	87	HSPA1A
ENSP00000225929	87	HSD17B1
ENSP00000364398	87	HABP4
ENSP00000248996	87	GNAZ
ENSP00000359385	87	GLMN
ENSP00000317385	87	GLIPR1L2
ENSP00000320828	87	EPS8L2

ENSP00000293831	87	EIF4A1
ENSP00000307080	87	EGLN2
ENSP00000335306	87	DPPA4
ENSP00000352137	87	DNTTIP2
ENSP00000221498	87	DKKL1
ENSP00000352308	87	DDX42
ENSP00000347087	87	DDX4
ENSP00000323155	87	DAND5
ENSP00000297848	87	COL14A1
ENSP00000326830	87	CLK1
ENSP00000396163	87	CLEC2A
ENSP00000341828	87	CHIA
ENSP00000283882	87	CFDP1
ENSP00000384040	87	CCDC85A
ENSP00000221740	87	CASP14
ENSP00000271971	87	CAPN9
ENSP00000295006	87	CAPN2
ENSP00000179259	87	C12orf5
ENSP00000342434	87	BAZ1B
ENSP00000005260	87	BAIAP2L1
ENSP00000260364	87	-
ENSP00000315719	87	-
ENSP00000345328	87	-
ENSP00000351268	87	-
ENSP00000351432	87	-
ENSP00000320924	86	WIPF2
ENSP00000329548	86	TUSC5
ENSP00000366729	86	TNFRSF9
ENSP00000367227	86	TMEM8B
ENSP00000326550	86	TACC3
ENSP00000351255	86	STAT4
ENSP00000273183	86	STAC
ENSP00000330221	86	SPSB1
ENSP00000295367	86	SPRR3
ENSP00000353238	86	SPN
ENSP00000283131	86	SMARCA5
ENSP00000323568	86	SLC2A2
ENSP00000394049	86	SLA
ENSP00000290399	86	SIM2
ENSP00000352148	86	S100A5
ENSP00000395708	86	RAPGEF3
ENSP00000386541	86	PSMD14
ENSP00000234071	86	PROC
ENSP00000288368	86	PREX2
ENSP00000263681	86	POLD3
ENSP00000398644	86	NUB1

ENSP00000322087	86	NHLH2
ENSP00000284503	86	NEIL2
ENSP00000286479	86	NAT2
ENSP00000346291	86	MYO18A
ENSP00000355536	86	MTR
ENSP00000296755	86	MAP1B
ENSP00000351669	86	MANEA
ENSP00000316891	86	LIMCH1
ENSP00000263598	86	LCN1
ENSP00000296233	86	KLF15
ENSP00000250377	86	KIAA0391
ENSP00000378132	86	IRX5
ENSP00000233954	86	IL1RL1
ENSP00000342513	86	IFI6
ENSP00000299767	86	HSP90B1
ENSP00000344844	86	HAVCR1
ENSP00000324494	86	GRHL1
ENSP00000234142	86	GREB1
ENSP00000362353	86	GLP1R
ENSP00000362460	86	GJB3
ENSP00000347464	86	GIT2
ENSP00000389770	86	FXYD3
ENSP00000323377	86	EXOC3
ENSP00000341680	86	DTNBP1
ENSP00000292782	86	DCUN1D1
ENSP00000252945	86	CYP2E1
ENSP00000293778	86	CXCL16
ENSP00000387006	86	CWC22
ENSP00000366248	86	CORT
ENSP00000242285	86	CLTA
ENSP00000335357	86	CDKN3
ENSP00000360532	86	CDC5L
ENSP00000352561	86	CALCR
ENSP00000301419	86	C19orf48
ENSP00000364683	86	BAMBI
ENSP00000264183	86	ARID4B
ENSP00000324277	86	APOLD1
ENSP00000344055	86	AP3D1
ENSP00000354193	86	AOC1
ENSP00000284984	86	ADAMTS1
ENSP00000317912	86	-
ENSP00000353806	86	-
ENSP00000347999	85	ZC3H7A
ENSP00000303709	85	UBE2E1
ENSP00000217133	85	TUBB1
ENSP00000301072	85	TUBA1C

ENSP00000252015	85	TRPC4AP
ENSP00000304467	85	THOP1
ENSP00000334280	85	TACC2
ENSP00000350153	85	SPOCK3
ENSP00000338606	85	SMUG1
ENSP00000417686	85	SLBP
ENSP00000329466	85	SIRT7
ENSP00000262519	85	SETD1A
ENSP00000419494	85	RYBP
ENSP00000337838	85	RTN4
ENSP00000251810	85	RRM2B
ENSP00000344909	85	PTP4A2
ENSP00000326018	85	PRX
ENSP00000375907	85	PID1
ENSP00000384408	85	PARG
ENSP00000382834	85	NPRL3
ENSP00000355966	85	NEK2
ENSP00000320886	85	MLXIPL
ENSP00000320936	85	IL17RA
ENSP00000369962	85	IGSF5
ENSP00000404524	85	HSPA1A
ENSP00000406359	85	HSPA1A
ENSP00000199936	85	HSD17B2
ENSP00000315949	85	HOXD8
ENSP00000331741	85	HOXB2
ENSP00000358154	85	HIST2H3C
ENSP00000309555	85	HCFC1
ENSP00000245206	85	GOT2
ENSP00000319254	85	GIPC3
ENSP00000418823	85	FTO
ENSP00000328720	85	FOXK1
ENSP00000221665	85	FIZ1
ENSP00000337128	85	EDN3
ENSP00000382213	85	DSPP
ENSP00000373169	85	DRD3
ENSP00000331681	85	DGCR6
ENSP00000324633	85	DEFB103B
ENSP00000369213	85	DDX58
ENSP00000354791	85	DCTN1
ENSP00000367527	85	DCLRE1C
ENSP00000261623	85	CYBA
ENSP00000310901	85	CSRP2
ENSP00000215939	85	CRYBB1
ENSP00000356370	85	CRB1
ENSP00000264613	85	CP
ENSP00000308236	85	COMMD1

ENSP00000309338	85	CLCF1
ENSP00000300692	85	CD3D
ENSP00000331636	85	CAPN12
ENSP00000269980	85	BCKDHA
ENSP00000083182	85	APPBP2
ENSP00000357459	85	ADAR
ENSP00000411096	85	ABCA13
ENSP00000215861	85	-
ENSP00000347280	85	-
ENSP00000364115	85	-
ENSP00000403005	85	-
ENSP00000414605	85	-
ENSP00000411051	84	ZNF2
ENSP00000248948	84	VPREB3
ENSP00000359070	84	STMN3
ENSP00000284776	84	SORBS2
ENSP00000302400	84	SMR3B
ENSP00000342082	84	SLPI
ENSP00000348307	84	SIRPA
ENSP00000302579	84	SCEL
ENSP00000311513	84	RSF1
ENSP00000380903	84	ROBO3
ENSP00000316729	84	RHOJ
ENSP00000064780	84	RELT
ENSP00000054950	84	RCN1
ENSP00000367894	84	PRRG1
ENSP00000266505	84	PLCZ1
ENSP00000327801	84	P4HB
ENSP00000272223	84	OSR1
ENSP00000356125	84	NUAK2
ENSP00000219302	84	NME3
ENSP00000354159	84	NLRC4
ENSP00000215754	84	MIF
ENSP00000354660	84	MAGEC2
ENSP00000263800	84	LTK
ENSP00000369317	84	KRT6A
ENSP00000307078	84	KIF5B
ENSP00000260363	84	KIF23
ENSP00000240874	84	KALRN
ENSP00000263642	84	IFIH1
ENSP00000348815	84	HYLS1
ENSP00000264126	84	GPSM2
ENSP00000274721	84	GFRA3
ENSP00000223366	84	GCK
ENSP00000416387	84	FBLIM1
ENSP00000261800	84	FAT2

ENSP00000302578	84	FAM84B
ENSP00000296677	84	F2RL1
ENSP00000336702	84	EIF5A
ENSP00000220764	84	DECR1
ENSP00000356817	84	DCAF6
ENSP00000312506	84	CSPG4
ENSP00000276571	84	CRH
ENSP00000206513	84	CEBPE
ENSP00000295324	84	CDC42EP3
ENSP00000353462	84	CASS4
ENSP00000362777	84	ATOH7
ENSP00000279873	84	ARID5B
ENSP00000293599	84	AQP5
ENSP00000300060	84	ANPEP
ENSP00000225737	84	AKAP10
ENSP00000300482	83	TRPM2
ENSP00000381567	83	TMF1
ENSP00000318635	83	SUMO4
ENSP00000329668	83	SHC4
ENSP00000326933	83	SERTAD2
ENSP00000299502	83	SERPINB2
ENSP00000342935	83	SDCBP2
ENSP00000357711	83	S100A7
ENSP00000217260	83	RSPO4
ENSP00000417164	83	ROBO2
ENSP00000351132	83	RHOT1
ENSP00000216455	83	PSMA3
ENSP00000287878	83	PRKAG2
ENSP00000302886	83	PA2G4
ENSP00000339503	83	NUDT1
ENSP00000366410	83	NMNAT1
ENSP00000276062	83	NDUFB11
ENSP00000354251	83	NCKAP1
ENSP00000349892	83	MYCBP2
ENSP00000246062	83	MKKS
ENSP00000262105	83	MCM4
ENSP00000294066	83	MAP4K2
ENSP00000292596	83	LTC4S
ENSP00000229214	83	KRR1
ENSP00000349154	83	KIAA1841
ENSP00000266719	83	KERA
ENSP00000297792	83	KDM1B
ENSP00000367130	83	HES3
ENSP00000369654	83	HBD
ENSP00000211122	83	GSTA3
ENSP00000237858	83	GLRX

ENSP00000348578	83	G3BP1
ENSP00000358510	83	FUNDC2
ENSP00000273550	83	FTH1
ENSP00000357789	83	FLG
ENSP00000216330	83	FKBP3
ENSP00000306361	83	FGA
ENSP00000230449	83	EXOC2
ENSP00000264956	83	EVC
ENSP00000216733	83	EFS
ENSP00000345853	83	DUSP9
ENSP00000376623	83	CSH2
ENSP00000351163	83	COL11A1
ENSP00000355922	83	CENPF
ENSP00000264463	83	CDH10
ENSP00000245903	83	CD70
ENSP00000269397	83	CBX4
ENSP00000263168	83	CAPZA1
ENSP00000255608	83	BTBD2
ENSP00000376204	83	ASH1L
ENSP00000253401	83	ARHGEF9
ENSP00000249066	83	APOL2
ENSP00000284268	83	ANKH
ENSP00000264377	83	ADAM23
ENSP00000354995	83	ABCG1
ENSP00000338141	83	-
ENSP00000383457	83	-
ENSP00000397424	83	-
ENSP00000253699	82	ZFYVE20
ENSP00000226760	82	WFS1
ENSP00000385361	82	VPREB1
ENSP00000219473	82	USP10
ENSP00000253023	82	UBE2M
ENSP00000335144	82	TPGS2
ENSP00000254051	82	TNS4
ENSP00000328879	82	TBL1Y
ENSP00000265404	82	STAP1
ENSP00000376423	82	SPRR2A
ENSP00000332488	82	SPPL2C
ENSP00000360163	82	SMARCA1
ENSP00000302077	82	SLFN12
ENSP00000337053	82	SEL1L
ENSP00000176195	82	SCT
ENSP00000348577	82	RANGAP1
ENSP00000303424	82	PTGDR
ENSP00000222266	82	PSENEN
ENSP00000313731	82	PLCD3

ENSP00000334105	82	PLCB4
ENSP00000245255	82	PIWIL1
ENSP00000368682	82	PHEX
ENSP00000294489	82	PDPN
ENSP00000354676	82	OSTC
ENSP00000420267	82	NR6A1
ENSP00000287139	82	NODAL
ENSP00000341992	82	NANOS3
ENSP00000037502	82	MYOC
ENSP00000330587	82	MT1H
ENSP00000265023	82	KNG1
ENSP00000160827	82	KIF22
ENSP00000357470	82	IL6R
ENSP00000343957	82	IFNAR2
ENSP00000313199	82	HNRNPD
ENSP00000318650	82	GREM2
ENSP00000241125	82	GJA3
ENSP00000306654	82	GFRA2
ENSP00000333097	82	FIGLA
ENSP00000320130	82	DYNC1I1
ENSP00000356791	82	DPT
ENSP00000265968	82	CSRP3
ENSP00000219599	82	CRYM
ENSP00000279463	82	CNTN5
ENSP00000322887	82	CIRBP
ENSP00000316121	82	CDCA8
ENSP00000265872	82	CCAR1
ENSP00000282356	82	CAMK4
ENSP00000263674	82	ARHGEF17
ENSP00000326884	82	ARFRP1
ENSP00000264276	82	ALS2
ENSP00000354991	82	ALPK2
ENSP00000269701	82	AKAP8
ENSP00000360761	82	AGPAT2
ENSP00000358730	82	ADORA3
ENSP00000258738	82	ABCB5
ENSP00000267837	82	-
ENSP00000289779	82	-
ENSP00000299466	82	-
ENSP00000304565	82	-
ENSP00000315334	82	-
ENSP00000387188	82	-
ENSP00000391188	82	-
ENSP00000262178	81	VIPR2
ENSP00000320401	81	UGT2B17
ENSP00000306015	81	SYNPO2

ENSP00000364430	81	SP5
ENSP00000275230	81	SLC2A12
ENSP00000306190	81	SLAMF1
ENSP00000368552	81	SIRT5
ENSP00000348068	81	SERPINA1
ENSP00000379760	81	RSL1D1
ENSP00000325677	81	RNF40
ENSP00000308576	81	RHOD
ENSP00000397181	81	RGS4
ENSP00000378394	81	PSAP
ENSP00000366488	81	PRKACG
ENSP00000378306	81	PPP3CB
ENSP00000301242	81	PPP1R14A
ENSP00000230732	81	POU4F3
ENSP00000345494	81	PLSCR1
ENSP00000264380	81	PIKFYVE
ENSP00000282096	81	PDE3B
ENSP00000261826	81	P2RX7
ENSP00000359121	81	OLFM3
ENSP00000336894	81	NAB1
ENSP00000401867	81	MXD3
ENSP00000370880	81	MLANA
ENSP00000328410	81	MACC1
ENSP00000346467	81	LTBP1
ENSP00000342112	81	LMO7
ENSP00000263726	81	LHX4
ENSP00000339952	81	KSR2
ENSP00000363524	81	KIF4A
ENSP00000278200	81	IMMP1L
ENSP00000349365	81	IL27
ENSP00000304440	81	HAS3
ENSP00000369643	81	GRPR
ENSP00000329292	81	GKN2
ENSP00000267430	81	FANCM
ENSP00000261172	81	EPYC
ENSP00000374323	81	EPHA6
ENSP00000408910	81	DCTN2
ENSP00000246891	81	CSN1S1
ENSP00000307713	81	BDKRB2
ENSP00000261168	81	ATF7IP
ENSP00000236850	81	APOA1
ENSP00000369960	81	ADRA1A
ENSP00000342905	81	ADNP
ENSP00000272286	81	ABCG8
ENSP00000372449	81	-
ENSP00000339767	80	ZNF433

ENSP00000300811	80	ZNF428
ENSP00000302770	80	ZNF296
ENSP00000366902	80	ZBTB48
ENSP00000361875	80	WFDC5
ENSP00000262376	80	UBN1
ENSP00000345984	80	TRAF3IP2
ENSP00000291232	80	TNFRSF13C
ENSP00000263904	80	STAM2
ENSP00000367124	80	SLC3A2
ENSP00000370990	80	SHOX
ENSP00000263071	80	SCARF1
ENSP00000265080	80	RASGRF2
ENSP00000237696	80	RARRES1
ENSP00000243501	80	PLA2G12A
ENSP00000391901	80	PHF1
ENSP00000337862	80	OVOL1
ENSP00000279147	80	ORAOV1
ENSP00000313169	80	NPHP1
ENSP00000217652	80	MYL12A
ENSP00000267079	80	MAP3K12
ENSP00000357020	80	LY9
ENSP00000347451	80	LINGO1
ENSP00000262776	80	LGALS3BP
ENSP00000395337	80	LDHA
ENSP00000355580	80	KCNK1
ENSP00000268296	80	ITGAX
ENSP00000072516	80	IL1RAP
ENSP00000382915	80	HSPA1A
ENSP00000328269	80	HMG20B
ENSP00000358162	80	HIST2H4A
ENSP00000264426	80	GRIA2
ENSP00000297469	80	GPER1
ENSP00000293195	80	FDXR
ENSP00000289902	80	FCER1G
ENSP00000333779	80	EVI2B
ENSP00000266517	80	ETNK1
ENSP00000317997	80	DUOX1
ENSP00000264192	80	CYTIP
ENSP00000242338	80	CNTFR
ENSP00000252456	80	CNN1
ENSP00000263671	80	CHRDL2
ENSP00000293636	80	CELA1
ENSP00000246006	80	CD93
ENSP00000342681	80	CD5
ENSP00000354947	80	CAPZA2
ENSP00000407188	80	C6orf47

ENSP00000354032	80	BSCL2
ENSP00000375248	80	BHLHA9
ENSP00000031135	80	BCLAF1
ENSP00000175506	80	ASNS
ENSP00000360985	80	ARFGEF2
ENSP00000265723	80	ABCB4
ENSP00000353157	80	2-Sep
ENSP00000294390	80	-
ENSP00000357513	80	-
ENSP00000395926	80	-
ENSP00000300619	79	ZNF91
ENSP00000351141	79	WTAP
ENSP00000254950	79	VPS4A
ENSP00000247829	79	TSPAN8
ENSP00000391561	79	TRGV9
ENSP00000295756	79	TRAT1
ENSP00000223795	79	TNFSF8
ENSP00000408581	79	TMEM132D
ENSP00000360222	79	TM2D1
ENSP00000386130	79	TIAF1
ENSP00000284811	79	TCEB1
ENSP00000312624	79	TCAP
ENSP00000357075	79	TAGLN2
ENSP00000263373	79	SPTBN4
ENSP00000353542	79	SPRR2D
ENSP00000366641	79	SLC2A5
ENSP00000357965	79	SETDB1
ENSP00000310658	79	SCUBE2
ENSP00000261693	79	SCARB1
ENSP00000318297	79	RUVBL1
ENSP00000256585	79	REG4
ENSP00000349003	79	PTGER3
ENSP00000343966	79	PSPC1
ENSP00000372155	79	PSME1
ENSP00000234347	79	PRTN3
ENSP00000299824	79	PPP1R16B
ENSP00000355260	79	PPP1R14C
ENSP00000373399	79	PIP5KL1
ENSP00000396445	79	OSGIN2
ENSP00000234296	79	ORC2
ENSP00000340998	79	NTN4
ENSP00000275857	79	NLGN4X
ENSP00000263969	79	MFN1
ENSP00000401980	79	MAVS
ENSP00000301873	79	LTBP3
ENSP00000256720	79	LPIN1

ENSP00000397140	79	KNTC1
ENSP00000234371	79	KISS1R
ENSP00000307321	79	HOXC10
ENSP00000325136	79	HADHB
ENSP00000256593	79	GSTM5
ENSP00000284562	79	GSTA5
ENSP00000355316	79	GRM3
ENSP00000285900	79	GRIA1
ENSP00000402457	79	GRAMD1B
ENSP00000295500	79	GPR155
ENSP00000368805	79	GCM2
ENSP00000359693	79	FKBP1C
ENSP00000310332	79	FBXO45
ENSP00000295834	79	FABP1
ENSP00000257192	79	DSG1
ENSP00000363023	79	DLGAP4
ENSP00000297581	79	DCSTAMP
ENSP00000369126	79	CSNK1A1L
ENSP00000384053	79	CSF2RB
ENSP00000238892	79	CRIPT
ENSP00000342136	79	CREB3
ENSP00000364976	79	CKS2
ENSP00000352264	79	CD2AP
ENSP00000239374	79	CCDC170
ENSP00000367408	79	CASK
ENSP00000251973	79	CARD10
ENSP00000296238	79	CAMK2N2
ENSP00000371497	79	C1QTNF3
ENSP00000224337	79	BLNK
ENSP00000301887	79	BATF2
ENSP00000357177	79	ARHGEF11
ENSP00000348602	79	AMPH
ENSP00000263177	79	-
ENSP00000278593	79	-
ENSP00000304875	79	-
ENSP00000313515	79	-
ENSP00000342993	79	-
ENSP00000393876	78	ZNF655
ENSP00000261332	78	ZNF24
ENSP00000309640	78	XXYLT1
ENSP00000385143	78	UNC5D
ENSP00000358478	78	TTF2
ENSP00000261884	78	TRIP4
ENSP00000369299	78	TRIM22
ENSP00000253934	78	TMEM204
ENSP00000326531	78	THOC6

ENSP00000348128	78	SVIL
ENSP00000278379	78	SLC1A2
ENSP00000321735	78	SLC16A8
ENSP00000236147	78	SELL
ENSP00000355499	78	SDCCAG8
ENSP00000260130	78	SDCBP
ENSP00000364235	78	RNF5
ENSP00000333194	78	RGS19
ENSP00000295755	78	RETNLB
ENSP00000267396	78	REM2
ENSP00000378090	78	RAD51D
ENSP00000216200	78	PVALB
ENSP00000331418	78	PTPRM
ENSP00000261479	78	PSMA6
ENSP00000412064	78	PRR13
ENSP00000393860	78	PLEKHA2
ENSP00000364249	78	PLA2G5
ENSP00000330918	78	PGP
ENSP00000376865	78	PDGFD
ENSP00000333900	78	OIT3
ENSP00000403383	78	NREP
ENSP00000300589	78	NOD2
ENSP00000327763	78	NLRP10
ENSP00000339958	78	NISCH
ENSP00000298352	78	NGB
ENSP00000268766	78	NEK8
ENSP00000289473	78	NCF1
ENSP00000265498	78	MGST2
ENSP00000367888	78	LRP4
ENSP00000356186	78	LAX1
ENSP00000246662	78	KRT9
ENSP00000309570	78	KLF7
ENSP00000302562	78	HSPBAP1
ENSP00000317659	78	GUK1
ENSP00000329380	78	GP1BA
ENSP00000333157	78	GH2
ENSP00000378368	78	FLOT2
ENSP00000265018	78	FAM184B
ENSP00000321326	78	F2R
ENSP00000300215	78	EPB42
ENSP00000234590	78	ENO1
ENSP00000356898	78	DDR2
ENSP00000310721	78	CYP7B1
ENSP00000246012	78	CST8
ENSP00000291554	78	CRYAA
ENSP00000370588	78	CD99

ENSP00000313601	78	CC2D1A
ENSP00000261448	78	CASQ2
ENSP00000254691	78	CARD6
ENSP00000346251	78	BPIFA1
ENSP00000340858	78	B2M
ENSP00000258417	78	ARMC9
ENSP00000239440	78	ARAP3
ENSP00000338260	78	APOL4
ENSP00000317674	78	APOL1
ENSP00000295974	78	APBB2
ENSP00000337736	78	AKAP1
ENSP00000260645	78	ABCG5
ENSP00000253050	78	-
ENSP00000292964	78	-
ENSP00000345090	78	-
ENSP00000412394	78	-
ENSP00000218348	77	USP11
ENSP00000298832	77	TTLL5
ENSP00000291526	77	TFF2
ENSP00000348234	77	TAT
ENSP00000374372	77	SPTB
ENSP00000315554	77	SPEM1
ENSP00000318900	77	SOBP
ENSP00000278426	77	SLC43A1
ENSP00000296444	77	SHISA5
ENSP00000264554	77	SHC2
ENSP00000350894	77	SERPINH1
ENSP00000370074	77	SERPINB9
ENSP00000265362	77	SEMA3A
ENSP00000350261	77	SAMD4A
ENSP00000320430	77	S100Z
ENSP00000367766	77	RPGR
ENSP00000360217	77	RHAG
ENSP00000348538	77	RBM38
ENSP00000309871	77	RACGAP1
ENSP00000261303	77	PSMC1
ENSP00000340510	77	PPL
ENSP00000216259	77	PMM1
ENSP00000357260	77	PMF1
ENSP00000352842	77	PFKM
ENSP00000337383	77	NLRP3
ENSP00000258829	77	NKX2-8
ENSP00000312678	77	MID1
ENSP00000263369	77	MIA
ENSP00000362638	77	MARCKSL1
ENSP00000274507	77	LECT2

ENSP00000164247	77	KCNAB2
ENSP00000327786	77	IZUMO1
ENSP00000374813	77	IGKV1D-42
ENSP00000257696	77	HILPDA
ENSP00000261416	77	HEXB
ENSP00000370023	77	HADHA
ENSP00000334448	77	GNG2
ENSP00000312999	77	GNAI2
ENSP00000335074	77	GHRL
ENSP00000268695	77	GALNS
ENSP00000329219	77	FMNL1
ENSP00000264870	77	F13A1
ENSP00000306999	77	ESCO2
ENSP00000292385	77	DBN1
ENSP00000354398	77	CYTH1
ENSP00000354901	77	CXCL9
ENSP00000341030	77	CSN2
ENSP00000364140	77	COL15A1
ENSP00000344609	77	BTG3
ENSP00000326391	77	BHLHA15
ENSP00000282185	77	ATG10
ENSP00000393887	77	AHSG
ENSP00000295771	77	-
ENSP00000339836	77	-
ENSP00000384168	77	-
ENSP00000397094	77	-
ENSP00000387462	76	ZBTB10
ENSP00000410083	76	VEZT
ENSP00000264031	76	UPK2
ENSP00000368538	76	TNFRSF4
ENSP00000370912	76	TEC
ENSP00000361824	76	SPTAN1
ENSP00000293894	76	SOX8
ENSP00000358297	76	RNF115
ENSP00000228843	76	RAD51AP1
ENSP00000265462	76	PRDX5
ENSP00000363284	76	POLE3
ENSP00000352288	76	PLXNB2
ENSP00000369100	76	PFKFB3
ENSP00000337405	76	PCID2
ENSP00000340328	76	NYX
ENSP00000333275	76	NR2C1
ENSP00000320324	76	NPEPPS
ENSP00000264668	76	MTRR
ENSP00000384690	76	MMD2
ENSP00000235332	76	MIIP

ENSP00000347514	76	MIER1
ENSP00000231449	76	IL4
ENSP00000282466	76	IGSF10
ENSP00000382714	76	IFITM2
ENSP00000360876	76	IFIT3
ENSP00000258400	76	HTR2B
ENSP00000340019	76	HSPD1
ENSP00000316786	76	HSD11B2
ENSP00000287936	76	HMGCR
ENSP00000251595	76	HBA2
ENSP00000275732	76	GIGYF1
ENSP00000001008	76	FKBP4
ENSP00000310842	76	FANCI
ENSP00000364028	76	ECE1
ENSP00000226004	76	DUSP3
ENSP00000324648	76	CYP2B6
ENSP00000381148	76	CTDSP2
ENSP00000395546	76	CSNK2B
ENSP00000406751	76	CRY2
ENSP00000362029	76	CIZ1
ENSP00000293780	76	CHRNE
ENSP00000296733	76	CDC20B
ENSP00000200307	76	CCL7
ENSP00000308815	76	CCL19
ENSP00000381758	76	CAPN11
ENSP00000341674	76	ANXA8
ENSP00000365255	76	ANKRD26
ENSP00000310071	76	ANAPC10
ENSP00000393381	76	-
ENSP00000397982	76	-
ENSP00000405289	75	ZNF778
ENSP00000301547	75	ZNF443
ENSP00000308921	75	ZCCHC12
ENSP00000262139	75	WIPI1
ENSP00000333266	75	UBA7
ENSP00000155926	75	TRIB2
ENSP00000303476	75	TLN2
ENSP00000246112	75	TLE6
ENSP00000262518	75	SRCAP
ENSP00000321108	75	SPOCK2
ENSP00000303212	75	SEMA3E
ENSP00000358423	75	RRAGD
ENSP00000259605	75	RNF38
ENSP00000301336	75	RILP
ENSP00000369411	75	RFC3
ENSP00000164139	75	PYGM

ENSP00000402060	75	PVR
ENSP00000287143	75	PRG3
ENSP00000377055	75	PRCP
ENSP00000386359	75	PPP1R1C
ENSP00000362296	75	POU3F4
ENSP00000312735	75	POLR2B
ENSP00000368349	75	POLA1
ENSP00000378461	75	PCBP4
ENSP00000220597	75	PAG1
ENSP00000304767	75	P2RY1
ENSP00000263036	75	OPTN
ENSP00000360154	75	OCRL
ENSP00000383295	75	NBEA
ENSP00000341021	75	NANOS2
ENSP00000352208	75	MYOF
ENSP00000357123	75	MNDA
ENSP00000230321	75	MDFI
ENSP00000354119	75	LAT
ENSP00000301653	75	KRT16
ENSP00000366894	75	KLF12
ENSP00000353331	75	KIRREL2
ENSP00000254654	75	ILKAP
ENSP00000358424	75	HSD3B2
ENSP00000379701	75	HNF4G
ENSP00000296503	75	HMGB2
ENSP00000291823	75	HIPK4
ENSP00000371927	75	GPR78
ENSP00000262366	75	GLIS2
ENSP00000325775	75	GJC3
ENSP00000359497	75	GBP2
ENSP00000327145	75	FLNC
ENSP00000318674	75	FBXL16
ENSP00000367059	75	ESPN
ENSP00000298937	75	ELP4
ENSP00000363559	75	EIF6
ENSP00000263791	75	EIF2AK4
ENSP00000270172	75	DNMT3L
ENSP00000367422	75	DLEU1
ENSP00000308344	75	DCAF7
ENSP00000297044	75	CYTH3
ENSP00000265136	75	COBL
ENSP00000281141	75	CDC123
ENSP00000307870	75	CCS
ENSP00000301774	75	BEST1
ENSP00000370227	75	AREGB
ENSP00000263574	75	APLP2

ENSP00000264908	75	ANXA3
ENSP00000349616	75	ADIPOR2
ENSP00000355739	75	ADCK3
ENSP00000225840	75	-
ENSP00000349096	75	-
ENSP00000372408	75	-
ENSP00000418778	75	-
ENSP00000251038	74	ZC3H14
ENSP00000262629	74	TYROBP
ENSP00000371206	74	TMEM138
ENSP00000389277	74	TMBIM6
ENSP00000265097	74	THOC3
ENSP00000275198	74	TAAR6
ENSP00000290271	74	STC1
ENSP00000371388	74	SRXN1
ENSP00000268704	74	SPG7
ENSP00000347314	74	SPG20
ENSP00000354720	74	SMC3
ENSP00000344468	74	SDC3
ENSP00000264431	74	RAPGEF2
ENSP00000258098	74	RAB11FIP5
ENSP00000354498	74	PHLDB1
ENSP00000362643	74	PHKA1
ENSP00000167218	74	PDCD2
ENSP00000381486	74	NOTO
ENSP00000353452	74	MYLK
ENSP00000200691	74	MT3
ENSP00000369146	74	MT1M
ENSP00000219271	74	MMP15
ENSP00000219542	74	METRN
ENSP00000266718	74	LUM
ENSP00000261978	74	LTBP2
ENSP00000215909	74	LGALS1
ENSP00000304422	74	KREMEN2
ENSP00000226725	74	KLHL2
ENSP00000193391	74	IMPG2
ENSP00000415900	74	IL31RA
ENSP00000329312	74	IGLL1
ENSP00000262854	74	HUWE1
ENSP00000232003	74	HRG
ENSP00000358851	74	GSTM4
ENSP00000241124	74	GJB6
ENSP00000295956	74	FLNB
ENSP00000276326	74	FBXO25
ENSP00000314829	74	FAM89B
ENSP00000338481	74	EPB41L2

ENSP00000292427	74	CYP11B1
ENSP00000262607	74	CECR1
ENSP00000357033	74	CD84
ENSP00000264157	74	CCNT2
ENSP00000372654	74	AQP4
ENSP00000369927	74	AKR1C3
ENSP00000380557	74	AKAP8L
ENSP00000410689	74	AFAP1
ENSP00000281455	74	ACSL1
ENSP00000284322	74	ABI3BP
ENSP00000160874	74	-
ENSP00000352377	74	-
ENSP00000357130	74	-
ENSP00000366871	74	-
ENSP00000358694	73	XPNPEP1
ENSP00000408534	73	TNFAIP8
ENSP00000008391	73	TFAP2D
ENSP00000420588	73	TFAM
ENSP00000362390	73	TBX22
ENSP00000348496	73	SUN5
ENSP00000295709	73	STK36
ENSP00000337446	73	STARD3
ENSP00000218089	73	STAG2
ENSP00000242465	73	SRGN
ENSP00000325785	73	SPECC1L
ENSP00000412309	73	SLC14A1
ENSP00000361640	73	SH3GLB2
ENSP00000304701	73	SH2B2
ENSP00000371936	73	SEMA5A
ENSP00000345751	73	SCNN1B
ENSP00000228284	73	SART3
ENSP00000329008	73	S100A7A
ENSP00000320898	73	RNF168
ENSP00000235382	73	RGS2
ENSP00000360519	73	RBP4
ENSP00000344829	73	QPCT
ENSP00000345341	73	PTGES2
ENSP00000245810	73	PSPN
ENSP00000261817	73	PSMD9
ENSP00000308720	73	PRSS1
ENSP00000296028	73	PPBP
ENSP00000347883	73	PLEKHA7
ENSP00000291009	73	PIP
ENSP00000373411	73	NKIRAS1
ENSP00000257724	73	MDFIC
ENSP00000362870	73	MATN1

ENSP00000221973	73	LIM2
ENSP00000260731	73	KIF11
ENSP00000356468	73	IVNS1ABP
ENSP00000355568	73	IRF2BP2
ENSP00000329553	73	IMMP2L
ENSP00000347754	73	HTR3A
ENSP00000370826	73	HR
ENSP00000368698	73	HIVEP1
ENSP00000263208	73	HIRA
ENSP00000260983	73	HECW2
ENSP00000347251	73	GPS1
ENSP00000220940	73	GML
ENSP00000278927	73	ESAM
ENSP00000369843	73	EPHX2
ENSP00000386881	73	DYSF
ENSP00000320340	73	DGKZ
ENSP00000408236	73	CYTH2
ENSP00000273153	73	CSRNP1
ENSP00000339155	73	CRISP2
ENSP00000336868	73	CENPA
ENSP00000261944	73	CDHR2
ENSP00000321753	73	C7orf63
ENSP00000393583	73	AZI1
ENSP00000337722	73	ARL6
ENSP00000295381	73	ARHGAP25
ENSP00000358807	73	ALX3
ENSP00000222388	73	ABCF2
ENSP00000382342	73	ABCC1
ENSP00000278752	73	-
ENSP00000367415	73	-
ENSP00000394422	73	-
ENSP00000338770	72	ZNF420
ENSP00000370303	72	ZDHHC21
ENSP00000358207	72	VAX1
ENSP00000254181	72	USP29
ENSP00000222275	72	UPK1A
ENSP00000216185	72	TXN2
ENSP00000257910	72	TSPAN31
ENSP00000354932	72	TLR1
ENSP00000322977	72	SSBP2
ENSP00000371554	72	SOD3
ENSP00000219334	72	SMPD3
ENSP00000316842	72	SIX5
ENSP00000362092	72	RRAGC
ENSP00000267205	72	RHOF
ENSP00000264926	72	RAD18

ENSP00000251772	72	PLXNA1
ENSP00000349208	72	PIWIL2
ENSP00000350263	72	PIGN
ENSP00000322579	72	PHF23
ENSP00000352717	72	OR13G1
ENSP00000250896	72	MKNK2
ENSP00000291688	72	MCM3AP
ENSP00000333821	72	MC2R
ENSP00000300843	72	MARK4
ENSP00000310933	72	MADD
ENSP00000359643	72	LPAR3
ENSP00000265598	72	LAMP3
ENSP00000375829	72	LAD1
ENSP00000261667	72	KPNA3
ENSP00000349252	72	ITGAL
ENSP00000324742	72	IL32
ENSP00000348784	72	IGBP1
ENSP00000160262	72	ICAM3
ENSP00000240652	72	IAPP
ENSP00000260843	72	GPR87
ENSP00000263634	72	GPR75-ASB3
ENSP00000245983	72	GNRH2
ENSP00000366525	72	FTL
ENSP00000288670	72	FMNL2
ENSP00000236938	72	FCRLA
ENSP00000267803	72	DUOXA1
ENSP00000284476	72	DISP1
ENSP00000304414	72	CXCR6
ENSP00000410400	72	CT47A11
ENSP00000328521	72	CRIP2
ENSP00000262315	72	CHTF18
ENSP00000234170	72	CEBPZ
ENSP00000222902	72	CCL24
ENSP00000253693	72	CAPN7
ENSP00000229922	72	CAP2
ENSP00000339740	72	CAMK2D
ENSP00000340761	72	BRI3BP
ENSP00000351642	72	BCAS4
ENSP00000336762	72	ANG
ENSP00000216489	72	ALKBH1
ENSP00000290953	72	AGRP
ENSP00000223357	72	AEBP1
ENSP00000295833	72	-
ENSP00000341734	72	-
ENSP00000384548	72	-
ENSP00000396664	72	-

ENSP00000408030	72	-
ENSP00000416259	72	-
ENSP00000287538	71	ZIC3
ENSP00000265293	71	WWC1
ENSP00000355317	71	WDTC1
ENSP00000350854	71	VPS13D
ENSP00000400312	71	ULK3
ENSP00000273610	71	UCN2
ENSP00000311219	71	TRIM59
ENSP00000312309	71	TNK1
ENSP00000361993	71	TIMM8A
ENSP00000320236	71	TIMM22
ENSP00000216513	71	SIX4
ENSP00000333896	71	RPS6KA4
ENSP00000321971	71	RHOT2
ENSP00000290524	71	RFX5
ENSP00000317636	71	RECQL5
ENSP00000416463	71	RASGRP4
ENSP00000019317	71	RALBP1
ENSP00000340879	71	RAD1
ENSP00000292513	71	PTGER1
ENSP00000157812	71	PSMC4
ENSP00000396813	71	PSMB9
ENSP00000359719	71	PRKACB
ENSP00000368646	71	PRDX4
ENSP00000356239	71	PPP1R12B
ENSP00000276914	71	PLIN2
ENSP00000297431	71	ORC5
ENSP00000233557	71	NRBP1
ENSP00000355190	71	NFE2L1
ENSP00000357341	71	NCOA7
ENSP00000300119	71	MYO1A
ENSP00000340900	71	MIA3
ENSP00000256178	71	LYVE1
ENSP00000244333	71	LYPD3
ENSP00000354848	71	KIAA1279
ENSP00000074304	71	INPP4A
ENSP00000339801	71	IDS
ENSP00000305973	71	HOXC4
ENSP00000377665	71	GAPVD1
ENSP00000370259	71	GABPB1
ENSP00000370373	71	FKBP1B
ENSP00000223136	71	FIS1
ENSP00000258201	71	FHOD1
ENSP00000359724	71	FHL1
ENSP00000327116	71	EHD3

ENSP00000355958	71	DTL
ENSP00000350616	71	DDC
ENSP00000323663	71	DBF4B
ENSP00000382166	71	CX3CR1
ENSP00000252050	71	CUL9
ENSP00000377470	71	CNP
ENSP00000408094	71	CLIC1
ENSP00000273853	71	CENPC
ENSP00000308165	71	CD36
ENSP00000308750	71	CBX2
ENSP00000244513	71	BTN1A1
ENSP00000205948	71	APOH
ENSP00000376705	71	ANO4
ENSP00000244533	71	ABCC10
ENSP00000247523	71	-
ENSP00000344343	71	-
ENSP00000390324	71	-
ENSP00000398943	71	-
ENSP00000348283	70	WWP2
ENSP00000258963	70	VEZF1
ENSP00000314480	70	UNCX
ENSP00000304845	70	UGT1A1
ENSP00000341289	70	TUBB4B
ENSP00000258821	70	TTC5
ENSP00000329869	70	TPO
ENSP00000357387	70	TPD52L1
ENSP00000312143	70	TNS3
ENSP00000350990	70	TNKS1BP1
ENSP00000344537	70	TAF6
ENSP00000266743	70	SYCP3
ENSP00000381164	70	SLC7A2
ENSP00000296043	70	SHROOM3
ENSP00000357182	70	SH2D2A
ENSP00000261918	70	SEMA7A
ENSP00000353332	70	RTEL1
ENSP00000273064	70	RQCD1
ENSP00000258349	70	RC3H1
ENSP00000372484	70	RBMY1B
ENSP00000352833	70	PTPN22
ENSP00000337641	70	PPP2R5E
ENSP00000348163	70	PLS3
ENSP00000357986	70	PLEKHA1
ENSP00000283243	70	PLA2R1
ENSP00000367177	70	PCDH8
ENSP00000250416	70	PARP2
ENSP00000193322	70	OSTM1

ENSP00000265634	70	NPTX2
ENSP00000222823	70	NOD1
ENSP00000364578	70	NELFE
ENSP00000359321	70	MTF2
ENSP00000334998	70	MT1B
ENSP00000367637	70	MRS2
ENSP00000264605	70	MLPH
ENSP00000365588	70	MDC1
ENSP00000254846	70	KDM6B
ENSP00000266031	70	HYAL1
ENSP00000239144	70	HOXB8
ENSP00000252951	70	HBZ
ENSP00000282020	70	GRID2
ENSP00000311528	70	GPR162
ENSP00000366246	70	GPC6
ENSP00000265070	70	GOLPH3
ENSP00000354486	70	GOLGA4
ENSP00000261302	70	FOXN3
ENSP00000318437	70	FDCSP
ENSP00000358820	70	EPS8L3
ENSP00000262056	70	EIF4B
ENSP00000257189	70	DSG3
ENSP00000408994	70	DET1
ENSP00000342070	70	CTSB
ENSP00000243914	70	CTCFL
ENSP00000375220	70	CTAGE1
ENSP00000393912	70	COPG2
ENSP00000369614	70	CNTROB
ENSP00000064724	70	CLDN11
ENSP00000418287	70	CIAO1
ENSP00000341206	70	CHST4
ENSP00000308021	70	CEP290
ENSP00000231021	70	CDH9
ENSP00000274170	70	CDH18
ENSP00000348554	70	CDC16
ENSP00000004921	70	CCL18
ENSP00000246533	70	CAPNS1
ENSP00000356263	70	C6orf211
ENSP00000402389	70	C1QTNF5
ENSP00000278174	70	BTBD10
ENSP00000385057	70	APOBEC3G
ENSP00000278359	70	APIP
ENSP00000296456	70	APEH
ENSP00000262219	70	ANXA13
ENSP00000317684	70	ACRV1
ENSP00000236130	70	-

ENSP00000299604	70	-
ENSP00000390237	70	-
ENSP00000403688	70	-
ENSP00000412047	70	-
ENSP00000254806	69	WBP2
ENSP00000324560	69	ULK1
ENSP00000323740	69	UCP3
ENSP00000231238	69	TTC1
ENSP00000011898	69	TSPAN9
ENSP00000261652	69	TNFRSF13B
ENSP00000349296	69	TMEM71
ENSP00000340505	69	SENP8
ENSP00000269298	69	SAT2
ENSP00000373884	69	RYR3
ENSP00000339381	69	RGS12
ENSP00000255476	69	RFXAP
ENSP00000279387	69	PPP4C
ENSP00000312411	69	PPM1E
ENSP00000345988	69	PLCH1
ENSP00000246229	69	PLAGL2
ENSP00000263946	69	PKP1
ENSP00000264864	69	PI4K2B
ENSP00000239940	69	PFN2
ENSP00000331574	69	PDE1A
ENSP00000254908	69	PCBD2
ENSP00000316454	69	PACS1
ENSP00000309771	69	P2RY6
ENSP00000324527	69	MYO1D
ENSP00000223167	69	MYL10
ENSP00000307280	69	MYL1
ENSP00000302177	69	MBOAT2
ENSP00000357624	69	MARCKS
ENSP00000340684	69	MAOA
ENSP00000368516	69	LGR4
ENSP00000318212	69	KCNH6
ENSP00000261233	69	IRAK3
ENSP00000374983	69	IGHE
ENSP00000319476	69	ICOS
ENSP00000316244	69	HTR1A
ENSP00000349270	69	HBM
ENSP00000274400	69	GTF2H2
ENSP00000334876	69	GRK1
ENSP00000364512	69	G6PC2
ENSP00000378529	69	FZR1
ENSP00000316460	69	FYB
ENSP00000348550	69	FRMD6

ENSP00000360635	69	DPP7
ENSP00000221114	69	DCTN6
ENSP00000366124	69	CST3
ENSP00000321606	69	CRMP1
ENSP00000256509	69	CHL1
ENSP00000325485	69	CERS5
ENSP00000325708	69	CD37
ENSP00000354782	69	CD247
ENSP00000357058	69	CASQ1
ENSP00000288139	69	CACNA1D
ENSP00000335229	69	C17orf82
ENSP00000261192	69	BCAT1
ENSP00000368237	69	ATL2
ENSP00000287394	69	ATAD2
ENSP00000322628	69	ARL4D
ENSP00000256737	69	ANO3
ENSP00000370254	69	AKR1C1
ENSP00000272928	69	ACKR3
ENSP00000333926	69	ABCC5
ENSP00000344155	69	ABCA2
ENSP00000352675	69	-
ENSP00000359334	69	-
ENSP00000403834	69	-
ENSP00000408478	69	-
ENSP00000383301	68	ZNF74
ENSP00000371715	68	ZG16B
ENSP00000313059	68	WNK1
ENSP00000286448	68	VAMP7
ENSP00000261497	68	USP22
ENSP00000420270	68	UHMK1
ENSP00000304811	68	UGT2B7
ENSP00000303148	68	TMEM37
ENSP00000307340	68	SPRR1A
ENSP00000329287	68	SNN
ENSP00000246515	68	SLURP1
ENSP00000326305	68	SLC25A20
ENSP00000262052	68	SLC11A2
ENSP00000338171	68	SKAP1
ENSP00000298386	68	RXFP2
ENSP00000302955	68	RRM2
ENSP00000331871	68	RHD
ENSP00000356641	68	RFWD2
ENSP00000338864	68	RASGRP2
ENSP00000244296	68	PSG1
ENSP00000363939	68	PPP3R2
ENSP00000225174	68	PPIF

ENSP00000362329	68	PPA1
ENSP00000222381	68	PON1
ENSP00000358696	68	PLXNA3
ENSP00000393847	68	PLA2G10
ENSP00000291547	68	PKNOX1
ENSP00000269554	68	PIP4K2B
ENSP00000232375	68	PFKFB4
ENSP00000303427	68	PDS5A
ENSP00000308024	68	PCSK1
ENSP00000223364	68	MYL7
ENSP00000354554	68	MT-CYB
ENSP00000314620	68	MS4A1
ENSP00000257979	68	MIP
ENSP00000292035	68	MED27
ENSP00000343706	68	MAGEH1
ENSP00000293745	68	KRT72
ENSP00000262189	68	KMT2C
ENSP00000361298	68	KIF2C
ENSP00000379712	68	IQSEC2
ENSP00000233957	68	IL18R1
ENSP00000417637	68	IGKV2D-29
ENSP00000382707	68	IFITM3
ENSP00000348150	68	HOMER3
ENSP00000397331	68	HLA-G
ENSP00000378408	68	GPT
ENSP00000230036	68	GPLD1
ENSP00000387170	68	GIGYF2
ENSP00000355632	68	GALNT2
ENSP00000274547	68	GABRB2
ENSP00000302756	68	FOXD4L1
ENSP00000318884	68	FMN2
ENSP00000368547	68	FEZ2
ENSP00000221466	68	FCGRT
ENSP00000353393	68	F8
ENSP00000418748	68	ESRP2
ENSP00000354238	68	ENPP1
ENSP00000386178	68	EBF2
ENSP00000381526	68	DPYSL3
ENSP00000386565	68	DPP10
ENSP00000258390	68	DOCK10
ENSP00000219240	68	DHODH
ENSP00000380352	68	DDHD2
ENSP00000355185	68	DCLRE1A
ENSP00000302543	68	CXXC5
ENSP00000320949	68	CNOT1
ENSP00000358460	68	CLIC2

ENSP00000338642	68	CIDEC
ENSP00000265148	68	CENPE
ENSP00000272298	68	CALM2
ENSP00000377840	68	CACNB1
ENSP00000297623	68	C9orf24
ENSP00000333769	68	BSG
ENSP00000262126	68	ANKRD12
ENSP00000261819	68	ANAPC5
ENSP00000296412	68	ADH5
ENSP00000232744	68	ABTB1
ENSP00000324196	68	AATK
ENSP00000228858	68	-
ENSP00000287364	68	-
ENSP00000367369	68	-
ENSP00000216639	67	VRK1
ENSP00000363506	67	UQCC1
ENSP00000303174	67	UGT1A6
ENSP00000246548	67	UBA2
ENSP00000369003	67	TRPC4
ENSP00000389998	67	TMEM67
ENSP00000278550	67	TENM4
ENSP00000370201	67	TAF9
ENSP00000260637	67	SULT6B1
ENSP00000222002	67	SULT2A1
ENSP00000009041	67	STARD3NL
ENSP00000355217	67	SNX6
ENSP00000264938	67	SLC9A3
ENSP00000265715	67	SLC26A4
ENSP00000336801	67	SLC25A26
ENSP00000296694	67	SCGB3A2
ENSP00000369547	67	S100G
ENSP00000344106	67	RTN3
ENSP00000403175	67	RPS18
ENSP00000354361	67	RFWD3
ENSP00000308699	67	REC8
ENSP00000365528	67	PTPRH
ENSP00000229328	67	PRKAB1
ENSP00000269582	67	PNMT
ENSP00000265382	67	PIP5K1B
ENSP00000384806	67	PDE4D
ENSP00000403948	67	PBX2
ENSP00000218068	67	PAGE4
ENSP00000396620	67	NFYC
ENSP00000253814	67	NDFIP1
ENSP00000245503	67	MYH2
ENSP00000276344	67	MAGEA4

ENSP00000215637	67	MADCAM1
ENSP00000263636	67	LY75
ENSP00000278193	67	LIN7C
ENSP00000261731	67	LHX5
ENSP00000046794	67	LCP2
ENSP00000352035	67	KCNQ2
ENSP00000370744	67	ITPR2
ENSP00000290759	67	ISL2
ENSP00000369312	67	IL15RA
ENSP00000363779	67	IKBKAP
ENSP00000417892	67	IGHD5-5
ENSP00000260118	67	GGH
ENSP00000220584	67	FDFT1
ENSP00000256797	67	ERN2
ENSP00000276461	67	ERLIN2
ENSP00000296754	67	ERAP1
ENSP00000340281	67	EIF4G2
ENSP00000269349	67	EIF4A3
ENSP00000296591	67	EDIL3
ENSP00000329539	67	DUSP8
ENSP00000378324	67	DNAJA4
ENSP00000368667	67	DDX53
ENSP00000305964	67	COX7B2
ENSP00000354856	67	CLK2
ENSP00000222792	67	CHN2
ENSP00000337103	67	CHAT
ENSP00000249014	67	CDC42EP1
ENSP00000370658	67	CCDC91
ENSP00000368102	67	CCDC3
ENSP00000277549	67	CACNA1B
ENSP00000377545	67	C1QTNF2
ENSP00000262947	67	C19orf10
ENSP00000247178	67	ATG14
ENSP00000228945	67	ARHGDIB
ENSP00000338967	67	ARHGAP6
ENSP00000371347	67	ANGPT4
ENSP00000332369	67	ALAS2
ENSP00000315615	67	AKAP5
ENSP00000356825	67	ADCY10
ENSP00000252173	67	-
ENSP00000294241	67	-
ENSP00000337605	67	-
ENSP00000413780	67	-
ENSP00000415836	66	ZNF276
ENSP00000003302	66	USP28
ENSP00000305941	66	USH2A

ENSP00000220959	66	UBR5
ENSP00000364403	66	UBR4
ENSP00000347836	66	UBE2H
ENSP00000374332	66	TSN
ENSP00000361162	66	TOE1
ENSP00000308753	66	TMOD3
ENSP00000267415	66	TINF2
ENSP00000343223	66	TCL1B
ENSP00000282470	66	SPARCL1
ENSP00000369237	66	SLC6A2
ENSP00000345492	66	SH2B3
ENSP00000356671	66	SERPINC1
ENSP00000359380	66	SCD
ENSP00000369899	66	RRAGA
ENSP00000324956	66	RNF14
ENSP00000361857	66	RLF
ENSP00000364476	66	RIBC1
ENSP00000246194	66	RALY
ENSP00000302647	66	RALGAPA1
ENSP00000254667	66	PTPRE
ENSP00000265562	66	PTPN23
ENSP00000312134	66	PRG2
ENSP00000261475	66	PPP2R3C
ENSP00000414138	66	PLCL1
ENSP00000359991	66	PGAM1
ENSP00000294338	66	PDZK1IP1
ENSP00000005178	66	PDK4
ENSP00000347689	66	PDE4C
ENSP00000307387	66	PDCD6IP
ENSP00000406157	66	PAPSS2
ENSP00000221166	66	NEFM
ENSP00000306997	66	MYOZ2
ENSP00000207437	66	MYL6B
ENSP00000350833	66	MPPED2
ENSP00000261326	66	MOCOS
ENSP00000352163	66	LYPD2
ENSP00000263925	66	LNX1
ENSP00000267119	66	KRT71
ENSP00000356319	66	KIF14
ENSP00000327611	66	KCNK12
ENSP00000357178	66	INSRR
ENSP00000411940	66	IFNA17
ENSP00000350387	66	HYAL2
ENSP00000319690	66	HNRNPC
ENSP00000216951	66	GSS
ENSP00000345282	66	GPT2

ENSP00000374390	66	GPR149
ENSP00000204726	66	GOLGA3
ENSP00000335196	66	GLDN
ENSP00000368226	66	GK
ENSP00000241256	66	GHSR
ENSP00000265294	66	GABRP
ENSP00000253801	66	G6PC
ENSP00000324573	66	FLII
ENSP00000274024	66	FABP2
ENSP00000296641	66	F2RL2
ENSP00000302936	66	DYNLRB2
ENSP00000401397	66	DDR1
ENSP00000273062	66	CTDSP1
ENSP00000216420	66	CGRRF1
ENSP00000361336	66	CDH22
ENSP00000335544	66	CCKBR
ENSP00000353295	66	CBWD3
ENSP00000084798	66	CA11
ENSP00000270233	66	BCAM
ENSP00000338072	66	AVPR2
ENSP00000349595	66	ATP2A1
ENSP00000364694	66	ASPN
ENSP00000000233	66	ARF5
ENSP00000339109	66	ANAPC1
ENSP00000225740	66	ALDH3A1
ENSP00000359245	66	ABCA4
ENSP00000301732	66	ABCA3
ENSP00000382541	66	-
ENSP00000382599	66	-
ENSP00000402956	66	-
ENSP00000291187	65	ZNF229
ENSP00000389381	65	YY2
ENSP00000346829	65	WLS
ENSP00000365899	65	VSX1
ENSP00000363397	65	UGCG
ENSP00000354340	65	UBA3
ENSP00000268483	65	TXNL4B
ENSP00000413437	65	TRGC2
ENSP00000353165	65	TPK1
ENSP00000317891	65	TNIP1
ENSP00000261713	65	TMEM98
ENSP00000300108	65	TAC3
ENSP00000366395	65	SYVN1
ENSP00000380695	65	SUDS3
ENSP00000248566	65	SHFM1
ENSP00000386306	65	SCN9A

ENSP00000346342	65	RFPL1
ENSP00000216127	65	RASD2
ENSP00000376848	65	PRIMA1
ENSP00000383402	65	PPP4R1
ENSP00000292614	65	POLR2J
ENSP00000367747	65	PLCH2
ENSP00000310585	65	PCP2
ENSP00000355419	65	PCDH11Y
ENSP00000330658	65	PAPPA
ENSP00000243346	65	NMI
ENSP00000369915	65	NAP1L4
ENSP00000393275	65	NANOS1
ENSP00000261507	65	MSMO1
ENSP00000420026	65	LYRM4
ENSP00000159111	65	KDM4B
ENSP00000296585	65	ITGA2
ENSP00000354451	65	IQGAP3
ENSP00000326500	65	IL11RA
ENSP00000265983	65	HPX
ENSP00000335060	65	HPDL
ENSP00000365402	65	HLA-C
ENSP00000362810	65	HCRTR1
ENSP00000395497	65	GTF2H4
ENSP00000396251	65	GTF2H4
ENSP00000265963	65	GTF2H1
ENSP00000377204	65	GRK6
ENSP00000344012	65	GAS2L1
ENSP00000335677	65	FOXK2
ENSP00000328103	65	EIF4ENIF1
ENSP00000307940	65	EEF2
ENSP00000216024	65	DMC1
ENSP00000261835	65	CYP46A1
ENSP00000337065	65	CXCL14
ENSP00000234701	65	CLCA1
ENSP00000357907	65	CDK19
ENSP00000358501	65	CD58
ENSP00000357149	65	CD1E
ENSP00000289429	65	CD1A
ENSP00000254262	65	C19orf40
ENSP00000341178	65	C15orf62
ENSP00000375082	65	BORA
ENSP00000361915	65	BMP8B
ENSP00000352219	65	BCS1L
ENSP00000298032	65	ARMC3
ENSP00000346550	65	ANXA6
ENSP00000362287	65	AGO3

ENSP00000348381	65	ADD3
ENSP00000262624	65	-
ENSP00000319125	65	-
ENSP00000363457	65	-
ENSP00000371928	65	-
ENSP00000374965	65	-
ENSP00000362446	64	ZNF79
ENSP00000338927	64	ZNF385A
ENSP00000341045	64	UGT2B15
ENSP00000369703	64	TUBB2A
ENSP00000254436	64	TRIM21
ENSP00000413697	64	TOM1
ENSP00000266732	64	TMPO
ENSP00000297784	64	TMC1
ENSP00000296702	64	TCERG1
ENSP00000255613	64	SUV420H2
ENSP00000319104	64	SUPT6H
ENSP00000305810	64	STX18
ENSP00000326085	64	STRA6
ENSP00000379353	64	ST8SIA1
ENSP00000216294	64	SNAPC1
ENSP00000372612	64	SLC25A52
ENSP00000373301	64	SH3BP5
ENSP00000246868	64	SBDS
ENSP00000267484	64	RTN1
ENSP00000342667	64	RNF19A
ENSP00000223862	64	RLN1
ENSP00000309117	64	RBFOX1
ENSP00000380271	64	RAPGEF4
ENSP00000348472	64	PXK
ENSP00000263708	64	PTPN4
ENSP00000298852	64	PSMC3
ENSP00000257836	64	PRRG4
ENSP00000262848	64	PRKX
ENSP00000318176	64	PRKRA
ENSP00000269844	64	PRDM15
ENSP00000225992	64	PPY
ENSP00000311677	64	PPP1R8
ENSP00000364243	64	PLA2G2F
ENSP00000346227	64	PKIB
ENSP00000256404	64	PEBP4
ENSP00000416193	64	P2RX6
ENSP00000219097	64	ORC6
ENSP00000328216	64	ORAI1
ENSP00000359532	64	NTSR1
ENSP00000258960	64	NMT1

ENSP00000313572	64	NIM1K
ENSP00000278836	64	MYRF
ENSP00000386331	64	MYO7A
ENSP00000347055	64	MYL4
ENSP00000387278	64	MPP4
ENSP00000325612	64	MED16
ENSP00000314901	64	LRRN1
ENSP00000264005	64	LCAT
ENSP00000397598	64	KY
ENSP00000336769	64	KIR2DL1
ENSP00000272748	64	KIAA1715
ENSP00000364550	64	KDM5C
ENSP00000155840	64	KCNQ1
ENSP00000240662	64	KCNJ8
ENSP00000321427	64	KCNH5
ENSP00000264661	64	KCNH4
ENSP00000365198	64	KAZN
ENSP00000262648	64	KAL1
ENSP00000231357	64	IRX4
ENSP00000323780	64	IP6K1
ENSP00000314425	64	IL12RB1
ENSP00000227752	64	IL10RA
ENSP00000364794	64	IARS
ENSP00000380785	64	HOOK2
ENSP00000351108	64	HNRNPAB
ENSP00000393198	64	HES4
ENSP00000297977	64	HDX
ENSP00000325290	64	GSG2
ENSP00000232461	64	GNAT1
ENSP00000393154	64	FBXO15
ENSP00000364486	64	FBP2
ENSP00000334145	64	F3
ENSP00000357175	64	ETV3
ENSP00000304736	64	ELOVL6
ENSP00000326342	64	ELMOD2
ENSP00000356076	64	DYRK3
ENSP00000348965	64	DYNC1H1
ENSP00000276202	64	DOCK11
ENSP00000383178	64	DIAPH3
ENSP00000260184	64	DDX60L
ENSP00000263239	64	DDX18
ENSP00000389792	64	DCDC1
ENSP00000362217	64	DACH2
ENSP00000319141	64	CYBRD1
ENSP00000376188	64	CRLF1
ENSP00000340211	64	CORO1B

ENSP00000299339	64	CLDN10
ENSP00000361721	64	CITED4
ENSP00000369325	64	CDKL5
ENSP00000225844	64	CCL13
ENSP00000225842	64	CCL1
ENSP00000416797	64	CAMSAP3
ENSP00000384193	64	CADM2
ENSP00000331625	64	C3orf35
ENSP00000350297	64	ASAP1
ENSP00000256682	64	ARF3
ENSP00000355340	64	APOBEC3C
ENSP00000244669	64	APOBEC2
ENSP00000319713	64	AGTRAP
ENSP00000303042	64	AFAP1L2
ENSP00000374071	64	ADAMTS20
ENSP00000265302	64	-
ENSP00000265870	64	-
ENSP00000269200	64	-
ENSP00000379035	64	-
ENSP00000394850	64	-
ENSP00000410636	64	-
ENSP00000273320	63	ZKSCAN7
ENSP00000364798	63	ZBTB40
ENSP00000248437	63	TUBA4A
ENSP00000324304	63	TSPAN4
ENSP00000373272	63	TRIM71
ENSP00000254250	63	THAP1
ENSP00000215838	63	TCN2
ENSP00000261205	63	SYT1
ENSP00000358635	63	SYNCRIP
ENSP00000360515	63	SUPT3H
ENSP00000350734	63	ST7L
ENSP00000347198	63	SRGAP1
ENSP00000264659	63	SRCIN1
ENSP00000352216	63	SLC2A10
ENSP00000289292	63	SHROOM4
ENSP00000245908	63	SH2D3A
ENSP00000306844	63	SEMA4C
ENSP00000375097	63	SBF1
ENSP00000355533	63	RYR2
ENSP00000349824	63	REPS2
ENSP00000336606	63	RAD54B
ENSP00000368666	63	PTCHD1
ENSP00000295901	63	PSMD6
ENSP00000310515	63	PRM1
ENSP00000319231	63	PHLDA2

ENSP00000337265	63	OSBPL9
ENSP00000234961	63	OPRD1
ENSP00000336528	63	NR1I2
ENSP00000263881	63	MAP4K3
ENSP00000404121	63	ILF3
ENSP00000356063	63	IL20
ENSP00000262345	63	IL12RB2
ENSP00000327824	63	IFNLR1
ENSP00000267845	63	HDC
ENSP00000356771	63	F5
ENSP00000358857	63	EMD
ENSP00000228741	63	ELK3
ENSP00000273668	63	EAF2
ENSP00000359790	63	DST
ENSP00000257198	63	DSC1
ENSP00000359170	63	DPCD
ENSP00000354643	63	DIO1
ENSP00000305725	63	CHST11
ENSP00000409378	63	CHRM4
ENSP00000315700	63	CHAF1B
ENSP00000382508	63	CDIP1
ENSP00000351118	63	CDHR5
ENSP00000293275	63	CCL16
ENSP00000266376	63	CACNA1C
ENSP00000349114	63	C7orf76
ENSP00000363298	63	BSPRY
ENSP00000297161	63	BMPER
ENSP00000263368	63	BLVRB
ENSP00000341562	63	B4GALNT1
ENSP00000281419	63	ASAP2
ENSP00000365411	63	APBB1IP
ENSP00000324074	63	ANP32E
ENSP00000313363	63	-
ENSP00000334101	63	-
ENSP00000354945	63	-
ENSP00000406470	63	-
ENSP00000383503	62	ZNRD1
ENSP00000363064	62	ZNF76
ENSP00000322872	62	ZKSCAN5
ENSP00000323183	62	ZBTB2
ENSP00000255198	62	ZBED3
ENSP00000411004	62	XG
ENSP00000361686	62	VDAC2
ENSP00000261427	62	UBE2K
ENSP00000222747	62	TSPAN12
ENSP00000226230	62	TMEM97

ENSP00000257637	62	TMEM243
ENSP00000266987	62	TARBP2
ENSP00000350162	62	SYCP2
ENSP00000311489	62	SPTBN2
ENSP00000290330	62	SNF8
ENSP00000252595	62	SLC27A1
ENSP00000321853	62	SERPINF2
ENSP00000270176	62	SCYL1
ENSP00000220676	62	RP1
ENSP00000217740	62	RNF125
ENSP00000361043	62	RAD54L
ENSP00000308926	62	PPM1J
ENSP00000222572	62	PON2
ENSP00000300055	62	PLIN1
ENSP00000336552	62	PKIA
ENSP00000334738	62	PITPNB
ENSP00000348888	62	PIGR
ENSP00000377502	62	PHLDB2
ENSP00000365943	62	PCSK5
ENSP00000351618	62	PARD3B
ENSP00000225328	62	P2RX5
ENSP00000272371	62	OTOF
ENSP00000219022	62	OLFM4
ENSP00000359128	62	NPM3
ENSP00000258317	62	NPL
ENSP00000417763	62	NAA10
ENSP00000265517	62	MTTP
ENSP00000364754	62	MCF2L
ENSP00000384313	62	MAST4
ENSP00000278198	62	LRRC4C
ENSP00000305653	62	LETM1
ENSP00000333298	62	LAMP1
ENSP00000295746	62	KIAA1524
ENSP00000243457	62	KCNJ2
ENSP00000419952	62	KCNAB1
ENSP00000263370	62	ITPKC
ENSP00000255078	62	IGHMBP2
ENSP00000276198	62	HTR2C
ENSP00000370445	62	HTN3
ENSP00000310111	62	HSPB7
ENSP00000382025	62	HLA-DQB1
ENSP00000348510	62	GTF3C1
ENSP00000086933	62	GSC2
ENSP00000301149	62	GPD1
ENSP00000416097	62	GOLGA2
ENSP00000332879	62	FLRT2

ENSP00000188790	62	FAP
ENSP00000312385	62	EVX2
ENSP00000292147	62	ETHE1
ENSP00000320543	62	EPN2
ENSP00000375073	62	EID2
ENSP00000354294	62	CTRB1
ENSP00000332371	62	COL7A1
ENSP00000295550	62	COL6A3
ENSP00000364000	62	COL5A2
ENSP00000221232	62	CNOT3
ENSP00000259216	62	CFC1
ENSP00000221992	62	CEACAM5
ENSP00000301200	62	CDC42EP5
ENSP00000271324	62	CD53
ENSP00000005180	62	CCL26
ENSP00000290354	62	CBR3
ENSP00000400401	62	CADPS2
ENSP00000216629	62	BDKRB1
ENSP00000345728	62	ATP7A
ENSP00000400806	62	APTX
ENSP00000377833	62	ANXA4
ENSP00000347454	62	ANO1
ENSP00000306620	62	ADCYAP1R1
ENSP00000271643	62	ADAMTSL4
ENSP00000362174	62	-
ENSP00000370439	62	-
ENSP00000382368	62	-
ENSP00000252211	61	ZKSCAN3
ENSP00000356320	61	ULBP2
ENSP00000298596	61	STOX1
ENSP00000369419	61	STEAP4
ENSP00000312946	61	STAB1
ENSP00000348984	61	SON
ENSP00000390084	61	SHISA6
ENSP00000338343	61	SGCD
ENSP00000361366	61	SFTPD
ENSP00000357861	61	SELENBP1
ENSP00000328968	61	SCN5A
ENSP00000319308	61	RGS5
ENSP00000224600	61	RBP3
ENSP00000384192	61	RASGRP3
ENSP00000175756	61	PTPN18
ENSP00000368914	61	PSTPIP1
ENSP00000324122	61	PRPF31
ENSP00000342026	61	PRDX6
ENSP00000340125	61	PPP1R17

ENSP00000368645	61	POLR2J3
ENSP00000240617	61	PLBD1
ENSP00000352173	61	PLAC1
ENSP00000215885	61	PLA2G3
ENSP00000349016	61	PEX14
ENSP00000362186	61	PCDH11X
ENSP00000306100	61	PAM
ENSP00000264169	61	ORC4
ENSP00000373196	61	OR5H6
ENSP00000249389	61	OPN1SW
ENSP00000256010	61	NTS
ENSP00000289820	61	NPM2
ENSP00000345147	61	NKX2-4
ENSP00000361475	61	NCS1
ENSP00000390131	61	NCR3
ENSP00000293422	61	MYL6
ENSP00000356214	61	MYCT1
ENSP00000307853	61	MUS81
ENSP00000359417	61	MTMR1
ENSP00000383333	61	MORC3
ENSP00000351664	61	MICAL1
ENSP00000280082	61	MIA2
ENSP00000391227	61	MGAT5B
ENSP00000356257	61	LMOD1
ENSP00000363458	61	LDLRAP1
ENSP00000361373	61	KLF17
ENSP00000264712	61	KIF3C
ENSP00000261244	61	KIAA0586
ENSP00000328150	61	KCNJ12
ENSP00000271002	61	ITGB3BP
ENSP00000376410	61	INF2
ENSP00000330959	61	IL1R2
ENSP00000382034	61	HLA-DQB1
ENSP00000245541	61	GGA3
ENSP00000369538	61	GDI2
ENSP00000264717	61	GCKR
ENSP00000265000	61	GALNT7
ENSP00000261304	61	GALC
ENSP00000351602	61	FUT4
ENSP00000362107	61	FHL3
ENSP00000385021	61	FANCL
ENSP00000353564	61	EXOC8
ENSP00000334100	61	EXOC7
ENSP00000247191	61	DLGAP5
ENSP00000227256	61	DBX1
ENSP00000367064	61	CUBN

ENSP00000332449	61	CRIP1
ENSP00000350359	61	CREB5
ENSP00000219150	61	CORO1A
ENSP00000341128	61	CLUL1
ENSP00000309415	61	CLTB
ENSP00000262622	61	CHST8
ENSP00000289957	61	CHRNB3
ENSP00000361151	61	CEL
ENSP00000199764	61	CEACAM6
ENSP00000259633	61	CD72
ENSP00000085219	61	CD22
ENSP00000338728	61	CCDC88A
ENSP00000373340	61	BRPF1
ENSP00000265523	61	BLVRA
ENSP00000258168	61	BCMO1
ENSP00000264381	61	BCHE
ENSP00000281437	61	BARX2
ENSP00000284629	61	ASZ1
ENSP00000273258	61	ARL6IP5
ENSP00000203786	61	ARHGAP4
ENSP00000386280	61	APOL6
ENSP00000364126	61	APEX2
ENSP00000265447	61	ANXA11
ENSP00000309259	61	ALAS1
ENSP00000355377	61	AKR7A3
ENSP00000351327	61	AKAP4
ENSP00000284308	61	-
ENSP00000309297	61	-
ENSP00000329736	61	-
ENSP00000330930	61	-
ENSP00000402065	61	-
ENSP00000420819	61	-
ENSP00000360817	60	ZNFX1
ENSP00000358590	60	ZNF292
ENSP00000265351	60	XPO5
ENSP00000305906	60	UTF1
ENSP00000258123	60	USP15
ENSP00000268379	60	UQCRC2
ENSP00000242576	60	UNG
ENSP00000370055	60	UBL3
ENSP00000313454	60	UBA6
ENSP00000364591	60	TSPYL2
ENSP00000291532	60	TMPRSS3
ENSP00000303992	60	TMEM43
ENSP00000361998	60	TAF7L
ENSP00000286713	60	STOM

ENSP00000366827	60	ST8SIA6
ENSP00000377262	60	SRPK2
ENSP00000366144	60	SOX21
ENSP00000244520	60	SNRPC
ENSP00000298532	60	SNAPC4
ENSP00000383698	60	SLC2A7
ENSP00000301761	60	SDHAF2
ENSP00000265565	60	SCAP
ENSP00000383411	60	SAMSN1
ENSP00000312439	60	RNF26
ENSP00000268125	60	RLBP1
ENSP00000344226	60	RASSF7
ENSP00000262879	60	RALGAPB
ENSP00000202677	60	RALGAPA2
ENSP00000319730	60	PRSS8
ENSP00000299443	60	POTED
ENSP00000355378	60	PLXNB3
ENSP00000300658	60	PGAP3
ENSP00000227868	60	PDHX
ENSP00000257789	60	ORC3
ENSP00000356708	60	OLIG3
ENSP00000314813	60	OAZ1
ENSP00000389071	60	NCR3
ENSP00000371221	60	MTMR6
ENSP00000379156	60	MPRIP
ENSP00000362166	60	MEAF6
ENSP00000262290	60	LPO
ENSP00000309576	60	LGALS8
ENSP00000347649	60	LEPREL4
ENSP00000348298	60	LCOR
ENSP00000382021	60	LCMT1
ENSP00000331242	60	KREMEN1
ENSP00000349168	60	HNRNPH1
ENSP00000265276	60	GPAM
ENSP00000317379	60	GLS
ENSP00000350170	60	FXR1
ENSP00000335493	60	FOXD2
ENSP00000310935	60	FKBP2
ENSP00000364742	60	FBXO42
ENSP00000363694	60	EPB41L4B
ENSP00000263360	60	EED
ENSP00000358563	60	DKC1
ENSP00000328690	60	DDX24
ENSP00000362795	60	CXCR3
ENSP00000231948	60	CRBN
ENSP00000273857	60	CORIN

ENSP00000221455	60	CLASRP
ENSP00000374145	60	CHRNG
ENSP00000240132	60	CHRNA2
ENSP00000356395	60	CFHR3
ENSP00000262262	60	CD33
ENSP00000280614	60	CCRN4L
ENSP00000337209	60	CCDC68
ENSP00000158166	60	CAMKK1
ENSP00000373509	60	C11orf31
ENSP00000349143	60	BTN2A2
ENSP00000261173	60	ATP2B1
ENSP00000264992	60	ASTE1
ENSP00000263382	60	ASF1B
ENSP00000377769	60	ARL13B
ENSP00000335560	60	ARHGEF25
ENSP00000353518	60	ANKS1A
ENSP00000072869	60	ADCK2
ENSP00000251582	60	ADAMTS2
ENSP00000311436	60	ABRA
ENSP00000225928	60	-
ENSP00000263115	60	-
ENSP00000264492	60	-
ENSP00000324617	60	-
ENSP00000342095	60	-
ENSP00000348562	60	-
ENSP00000381049	60	-
ENSP00000383330	60	-
ENSP00000413419	60	-
ENSP00000419072	60	-
ENSP00000294740	59	ZNF281
ENSP00000303820	59	ZIK1
ENSP00000377944	59	UTP14A
ENSP00000323889	59	TRIM25
ENSP00000358765	59	TPBG
ENSP00000397379	59	TNPO2
ENSP00000248244	59	TICAM1
ENSP00000204517	59	TFAP4
ENSP00000346065	59	TDRD6
ENSP00000362068	59	TAF8
ENSP00000267377	59	SSTR1
ENSP00000359484	59	SMAP1
ENSP00000320246	59	SLC9A9
ENSP00000381399	59	SLC2A11
ENSP00000238508	59	SERPINB10
ENSP00000396320	59	SCN4A
ENSP00000364554	59	SCN1A

ENSP00000354622	59	RUNDC1
ENSP00000304321	59	RNF150
ENSP00000288199	59	RNF111
ENSP00000340176	59	RBPMS
ENSP00000366829	59	RBM10
ENSP00000359793	59	PTGFR
ENSP00000384211	59	PSME4
ENSP00000230381	59	PRPH2
ENSP00000264714	59	PPM1G
ENSP00000263212	59	PPM1F
ENSP00000410626	59	PHRF1
ENSP00000384048	59	PAXIP1
ENSP00000322957	59	PAK7
ENSP00000282516	59	NIPBL
ENSP00000325239	59	MYLPF
ENSP00000323184	59	MYCBPAP
ENSP00000354876	59	MT-CO2
ENSP00000304096	59	MPHOSPH9
ENSP00000246186	59	MMP24
ENSP00000348308	59	MMP23B
ENSP00000357798	59	MMP21
ENSP00000297153	59	MDGA1
ENSP00000251472	59	MAST1
ENSP00000343298	59	LRRC41
ENSP00000344401	59	LIN28B
ENSP00000308369	59	LEMD3
ENSP00000343701	59	KPNA1
ENSP00000317141	59	KCTD12
ENSP00000358784	59	KCNA3
ENSP00000415769	59	ITIH3
ENSP00000244174	59	IL9R
ENSP00000295981	59	IL17RC
ENSP00000288167	59	IL17RB
ENSP00000004982	59	HSPB6
ENSP00000308107	59	HPSE
ENSP00000397705	59	HLA-F
ENSP00000295934	59	HESX1
ENSP00000378492	59	GRM2
ENSP00000366267	59	GPC5
ENSP00000297537	59	GBX1
ENSP00000353910	59	FUT8
ENSP00000313691	59	FTMT
ENSP00000373370	59	FLG2
ENSP00000365944	59	FBXO6
ENSP00000345008	59	FBLN5
ENSP00000248070	59	EPS15L1

ENSP00000261486	59	EPB41L4A
ENSP00000253039	59	EIF2S3
ENSP00000356056	59	DYNLT1
ENSP00000315659	59	DNMBP
ENSP00000382133	59	DNA2
ENSP00000267889	59	DISP2
ENSP00000295683	59	CXCR1
ENSP00000309524	59	CSHL1
ENSP00000267085	59	CSAD
ENSP00000362734	59	CRB2
ENSP00000367265	59	CKAP4
ENSP00000301280	59	CHAF1A
ENSP00000332139	59	CFD
ENSP00000267383	59	CDH24
ENSP00000350003	59	CCR3
ENSP00000293280	59	CCL23
ENSP00000335657	59	CCK
ENSP00000264645	59	CASC3
ENSP00000262498	59	C16orf80
ENSP00000329106	59	BPY2
ENSP00000290299	59	ATP5O
ENSP00000253004	59	ASS1
ENSP00000364092	59	ASIP
ENSP00000261783	59	ARG2
ENSP00000357066	59	ARG1
ENSP00000363988	59	ALDOB
ENSP00000310459	59	AKIP1
ENSP00000343782	59	ADRB3
ENSP00000348912	59	ADAM33
ENSP00000303211	59	ACHE
ENSP00000250615	59	AANAT
ENSP00000222836	59	-
ENSP00000275189	59	-
ENSP00000295982	59	-
ENSP00000299671	59	-
ENSP00000319771	59	-
ENSP00000343563	59	-
ENSP00000368675	59	-
ENSP00000393742	59	-
ENSP00000340494	58	ZNF395
ENSP00000320347	58	ZFP3
ENSP00000354453	58	ZFP2
ENSP00000349503	58	ZBTB14
ENSP00000356562	58	XPR1
ENSP00000376792	58	VTI1A
ENSP00000220509	58	VPS18

ENSP00000054666	58	VAMP3
ENSP00000360613	58	UBE2A
ENSP00000336799	58	TUBA1B
ENSP00000267890	58	TTBK2
ENSP00000362111	58	TSPAN6
ENSP00000261862	58	THSD4
ENSP00000233156	58	TFPI
ENSP00000334042	58	TAC4
ENSP00000246071	58	SNRPB2
ENSP00000308057	58	SNCB
ENSP00000298472	58	SLC18A2
ENSP00000358464	58	SHOC2
ENSP00000345193	58	SHANK2
ENSP00000415786	58	SERPINE2
ENSP00000284136	58	SEMA3D
ENSP00000357555	58	RPS27
ENSP00000303192	58	RGL1
ENSP00000262031	58	RBMS2
ENSP00000312837	58	RAB11FIP4
ENSP00000338173	58	PUM2
ENSP00000294484	58	PTCHD2
ENSP00000210313	58	PSMD5
ENSP00000351314	58	PSMB10
ENSP00000287600	58	PDE6D
ENSP00000221784	58	PDCD5
ENSP00000396995	58	PAPD5
ENSP00000324270	58	OXTR
ENSP00000156084	58	OTUD5
ENSP00000304858	58	ORMDL3
ENSP00000255262	58	NMUR2
ENSP00000216121	58	NIPSNAP1
ENSP00000386134	58	NGDN
ENSP00000353154	58	NFASC
ENSP00000299641	58	NDST2
ENSP00000384971	58	LRRC72
ENSP00000231368	58	LNPEP
ENSP00000363944	58	LAS1L
ENSP00000293774	58	KRT4
ENSP00000314783	58	KLK15
ENSP00000347076	58	KIF16B
ENSP00000306565	58	ISG20
ENSP00000256452	58	IL5RA
ENSP00000366234	58	IL31
ENSP00000360730	58	IL13RA1
ENSP00000271588	58	HMCN1
ENSP00000284154	58	GRAP

ENSP00000258456	58	GPR45
ENSP00000308782	58	GP6
ENSP00000395772	58	GNL3
ENSP00000410732	58	GABRG2
ENSP00000399588	58	GAB3
ENSP00000403067	58	FRMD5
ENSP00000314806	58	FOXR1
ENSP00000391438	58	FLOT1
ENSP00000264211	58	EIF4G3
ENSP00000276682	58	EIF3H
ENSP00000358043	58	ECM1
ENSP00000286692	58	DRAM2
ENSP00000371846	58	DEAF1
ENSP00000292174	58	CXCR5
ENSP00000370213	58	CLCN4
ENSP00000276410	58	CHRNA6
ENSP00000219235	58	CCL22
ENSP00000256460	58	CAMK1
ENSP00000390329	58	CACNA2D2
ENSP00000372815	58	C4A
ENSP00000341787	58	BTNL3
ENSP00000349351	58	BICD2
ENSP00000369055	58	B4GALT1
ENSP00000340672	58	ARMCX3
ENSP00000255040	58	APCS
ENSP00000306185	58	ANTXR2
ENSP00000300245	58	AKTIP
ENSP00000317087	58	8-Mar
ENSP00000306939	58	-
ENSP00000329343	58	-
ENSP00000379943	58	-
ENSP00000337034	57	ZMYM5
ENSP00000262630	57	ZBTB32
ENSP00000329803	57	ZAR1
ENSP00000354919	57	VEPH1
ENSP00000258499	57	USP44
ENSP00000343526	57	USP1
ENSP00000290650	57	UBR1
ENSP00000263228	57	UBE2R2
ENSP00000174621	57	TRPV1
ENSP00000381672	57	TP53TG3
ENSP00000347930	57	TMEM30B
ENSP00000262319	57	TELO2
ENSP00000352463	57	TCF20
ENSP00000207157	57	TBX15
ENSP00000310094	57	TAOK2

ENSP00000275216	57	TAAR1
ENSP00000317714	57	STX4
ENSP00000311609	57	SPSB4
ENSP00000341327	57	SOCS4
ENSP00000270066	57	SMG9
ENSP00000280871	57	SLC2A13
ENSP00000305976	57	SIK2
ENSP00000381698	57	SHARPIN
ENSP00000301293	57	SEMA6B
ENSP00000390600	57	SCN10A
ENSP00000358374	57	SCAF11
ENSP00000346067	57	RPSA
ENSP00000262340	57	RPE65
ENSP00000413035	57	RBFOX2
ENSP00000362751	57	RABGAP1
ENSP00000270631	57	PTH2
ENSP00000291294	57	PTGIR
ENSP00000412788	57	PTBP2
ENSP00000327704	57	PSMF1
ENSP00000264639	57	PSMD3
ENSP00000350249	57	POT1
ENSP00000322570	57	POLE
ENSP00000346809	57	PGAP1
ENSP00000346725	57	PES1
ENSP00000242994	57	NEUROD4
ENSP00000267023	57	NABP2
ENSP00000296543	57	NAA15
ENSP00000245185	57	MT2A
ENSP00000278888	57	MS4A2
ENSP00000239761	57	MRC1
ENSP00000359818	57	MMS19
ENSP00000417235	57	MED12L
ENSP00000292494	57	LY6E
ENSP00000348762	57	LSS
ENSP00000354805	57	LRMP
ENSP00000314837	57	KLC2
ENSP00000296343	57	KIAA0226
ENSP00000347504	57	ITGB1BP1
ENSP00000358956	57	IRAK1BP1
ENSP00000345096	57	IMPDH1
ENSP00000259206	57	IL1RN
ENSP00000302924	57	IL17D
ENSP00000418649	57	IGKV3-20
ENSP00000281938	57	HSPB8
ENSP00000367065	57	HES2
ENSP00000359864	57	GPC4

ENSP00000290374	57	GJD2
ENSP00000351473	57	GIMAP5
ENSP00000260282	57	FXYD6
ENSP00000372873	57	FLOT1
ENSP00000353025	57	FAM3C
ENSP00000334285	57	FAM170A
ENSP00000406209	57	EPN1
ENSP00000221730	57	EPHX3
ENSP00000311010	57	ELF5
ENSP00000349594	57	ELAVL4
ENSP00000276651	57	DPYS
ENSP00000392066	57	DIP2A
ENSP00000356985	57	DEDD
ENSP00000311095	57	CYP4A11
ENSP00000342007	57	CYP1A2
ENSP00000350911	57	CTSE
ENSP00000355013	57	CTR9
ENSP00000370935	57	CSF2RA
ENSP00000323696	57	CRTAP
ENSP00000011292	57	CPA1
ENSP00000362776	57	COL16A1
ENSP00000298159	57	CFL2
ENSP00000306579	57	CERS6
ENSP00000244336	57	CEACAM8
ENSP00000340210	57	CD59
ENSP00000219244	57	CCL17
ENSP00000303525	57	CBR4
ENSP00000356037	57	C4BPA
ENSP00000215376	57	C19orf26
ENSP00000261318	57	C12orf49
ENSP00000261658	57	BFAR
ENSP00000318883	57	BCL2L13
ENSP00000268057	57	BBS4
ENSP00000322159	57	ATG4C
ENSP00000347433	57	APCDD1
ENSP00000253109	57	ANGPTL6
ENSP00000234816	57	ANGPTL1
ENSP00000312606	57	AKR1A1
ENSP00000291582	57	AIRE
ENSP00000310688	57	ABCD2
ENSP00000234923	57	-
ENSP00000262499	57	-
ENSP00000273481	57	-
ENSP00000299218	57	-
ENSP00000299633	57	-
ENSP00000375413	57	-

ENSP00000391210	57	-
ENSP00000397117	57	-
ENSP00000399122	57	-
ENSP00000408089	57	-
ENSP00000413007	57	-
ENSP00000222033	56	ZNRF4
ENSP00000340171	56	ZNF573
ENSP00000243643	56	ZNF415
ENSP00000299667	56	ZNF3
ENSP00000296099	56	UCN
ENSP00000252936	56	TUBGCP2
ENSP00000354006	56	TRPM6
ENSP00000361811	56	TP53TG5
ENSP00000245817	56	TNFSF9
ENSP00000347948	56	TNFRSF14
ENSP00000303145	56	TMED10
ENSP00000364336	56	TBXA2R
ENSP00000355560	56	TBCE
ENSP00000225525	56	TAX1BP3
ENSP00000248600	56	STYXL1
ENSP00000359988	56	SRSF11
ENSP00000301740	56	SRRM2
ENSP00000352097	56	SPTSSB
ENSP00000305343	56	SOX14
ENSP00000370128	56	SLC7A1
ENSP00000264784	56	SLC2A9
ENSP00000262502	56	SLC12A3
ENSP00000273905	56	SLC10A6
ENSP00000354829	56	SGMS1
ENSP00000304945	56	ROBO4
ENSP00000289013	56	RHPN1
ENSP00000233735	56	REG1A
ENSP00000335029	56	RASA3
ENSP00000252804	56	PXDN
ENSP00000325958	56	PTPMT1
ENSP00000368144	56	PRR5L
ENSP00000328178	56	PPP1R2
ENSP00000312769	56	PPIE
ENSP00000219252	56	POLR2C
ENSP00000353646	56	PLEKHG4
ENSP00000364246	56	PLA2G2D
ENSP00000349205	56	PIK3R4
ENSP00000329933	56	PHF12
ENSP00000397669	56	PHACTR1
ENSP00000202017	56	PDRG1
ENSP00000351957	56	PDE3A

ENSP00000262545	56	PCSK2
ENSP00000332823	56	NR2C2AP
ENSP00000305288	56	NLGN2
ENSP00000333938	56	NEXN
ENSP00000380444	56	MYO9B
ENSP00000384651	56	MTMR3
ENSP00000250144	56	MMP28
ENSP00000219905	56	MGA
ENSP00000302037	56	MFF
ENSP00000323720	56	MED14
ENSP00000243911	56	MC3R
ENSP00000374448	56	LRCH1
ENSP00000353826	56	LONP1
ENSP00000351933	56	KLHL9
ENSP00000368976	56	KIFC3
ENSP00000254712	56	INPP5K
ENSP00000419598	56	IGKV1-9
ENSP00000386187	56	IFITM1
ENSP00000259241	56	HS6ST1
ENSP00000355167	56	HORMAD1
ENSP00000306138	56	GRM5
ENSP00000370779	56	GPR139
ENSP00000331313	56	GNB1L
ENSP00000394071	56	GDI1
ENSP00000264072	56	FCER2
ENSP00000364475	56	FBP1
ENSP00000347294	56	EYA4
ENSP00000334675	56	ERCC6L
ENSP00000346412	56	ELP5
ENSP00000266126	56	EIF2B2
ENSP00000417980	56	EHMT1
ENSP00000383303	56	DSCAM
ENSP00000368523	56	DNAJC15
ENSP00000335304	56	DLST
ENSP00000235180	56	DLGAP3
ENSP00000003100	56	CYP51A1
ENSP00000360247	56	CYP2J2
ENSP00000259470	56	CTSV
ENSP00000330523	56	CTHRC1
ENSP00000345079	56	CSRP1
ENSP00000333268	56	CSH1
ENSP00000261951	56	CNOT6
ENSP00000326699	56	CLGN
ENSP00000300283	56	CKMT1B
ENSP00000306968	56	CDCA7
ENSP00000409197	56	CCL14

ENSP00000309681	56	CCDC106
ENSP00000346017	56	CALCB
ENSP00000226021	56	CACNG1
ENSP00000327440	56	BMP8A
ENSP00000359474	56	BARHL2
ENSP00000375872	56	ATG16L1
ENSP00000254584	56	ARFIP2
ENSP00000360170	56	ANGPTL3
ENSP00000377083	56	ALDH1L1
ENSP00000362306	56	AGO4
ENSP00000381840	56	AEBP2
ENSP00000158762	56	ACAP1
ENSP00000204279	56	-
ENSP00000273612	56	-
ENSP00000313859	56	-
ENSP00000350511	56	-
ENSP00000370656	56	-
ENSP00000381076	56	-
ENSP00000382366	56	-
ENSP00000382842	56	-
ENSP00000408580	56	-
ENSP00000306279	55	UMOD
ENSP00000240587	55	TSHZ3
ENSP00000398677	55	TRIM31
ENSP00000338989	55	TRIM16
ENSP00000339405	55	TP53TG3B
ENSP00000301057	55	TP53I13
ENSP00000328207	55	TNFRSF18
ENSP00000410668	55	TNF
ENSP00000366266	55	TMEM2
ENSP00000366603	55	TGOLN2
ENSP00000346206	55	TAP1
ENSP00000299424	55	TAF10
ENSP00000282999	55	SRP19
ENSP00000361666	55	SLC22A7
ENSP00000386444	55	SAG
ENSP00000202773	55	RPL6
ENSP00000254260	55	RHPN2
ENSP00000305071	55	RFXANK
ENSP00000383093	55	RFPL3S
ENSP00000258062	55	REPS1
ENSP00000354963	55	RASGEF1C
ENSP00000360286	55	RAE1
ENSP00000012443	55	PPP5C
ENSP00000336931	55	PNRC1
ENSP00000336831	55	PLS1

ENSP00000270861	55	PLK4
ENSP00000331678	55	PKP3
ENSP00000362116	55	PGC
ENSP00000342356	55	OSTN
ENSP00000225388	55	NUFIP2
ENSP00000359024	55	NOLC1
ENSP00000357581	55	NKX6-2
ENSP00000360576	55	NDOR1
ENSP00000290231	55	NCOA5
ENSP00000306382	55	MYO1B
ENSP00000380702	55	MYCBP
ENSP00000310375	55	MLC1
ENSP00000314543	55	MAGEB18
ENSP00000324701	55	MAB21L2
ENSP00000273286	55	LRTM1
ENSP00000334373	55	KPNA4
ENSP00000240618	55	KLRK1
ENSP00000311505	55	IQCB1
ENSP00000239316	55	INSL4
ENSP00000370842	55	IL33
ENSP00000328133	55	IL20RB
ENSP00000316664	55	IGSF8
ENSP00000276943	55	IFNK
ENSP00000337425	55	HYAL3
ENSP00000353915	55	HTR4
ENSP00000326649	55	HPS1
ENSP00000381237	55	HM13
ENSP00000410645	55	HLA-A
ENSP00000264553	55	GZMM
ENSP00000231009	55	GZMK
ENSP00000356842	55	GPA33
ENSP00000314508	55	GBA
ENSP00000376465	55	GALNT3
ENSP00000398597	55	EXOSC6
ENSP00000269214	55	ESCO1
ENSP00000361746	55	EPPIN
ENSP00000361668	55	EDN2
ENSP00000250024	55	E2F8
ENSP00000303550	55	CYTL1
ENSP00000301391	55	CYB5D2
ENSP00000286758	55	CXCL13
ENSP00000353904	55	CSNK1G3
ENSP00000339399	55	CRYZ
ENSP00000343746	55	CORO2A
ENSP00000250340	55	CLEC11A
ENSP00000381522	55	CHD9

ENSP00000264705	55	CAD
ENSP00000267012	55	BIN2
ENSP00000364883	55	AUH
ENSP00000313171	55	ATAD5
ENSP00000295685	55	ARPC2
ENSP00000332788	55	ANO9
ENSP00000352584	55	AKR1B10
ENSP00000357873	55	ACADSB
ENSP00000259050	55	7-Mar
ENSP00000348444	55	-
ENSP00000390424	55	-
ENSP00000407321	55	-
ENSP00000325634	54	ZNF639
ENSP00000354964	54	ZNF318
ENSP00000230122	54	ZBTB24
ENSP00000361761	54	WFDC2
ENSP00000233615	54	WBP1
ENSP00000167106	54	VASH1
ENSP00000314214	54	VAMP2
ENSP00000337393	54	UXT
ENSP00000229268	54	USP5
ENSP00000342222	54	TRPV2
ENSP00000335371	54	TRAPPC11
ENSP00000406482	54	THSD7A
ENSP00000345772	54	TEAD3
ENSP00000310701	54	TEAD2
ENSP00000307260	54	TBL2
ENSP00000314971	54	TAF1D
ENSP00000356236	54	SYT2
ENSP00000176763	54	STK10
ENSP00000300737	54	STIM1
ENSP00000290902	54	SPON2
ENSP00000300917	54	SMG8
ENSP00000293441	54	SHANK1
ENSP00000358703	54	RIPPLY2
ENSP00000383953	54	RGS6
ENSP00000356529	54	RGS16
ENSP00000226193	54	RCVRN
ENSP00000264400	54	RASGEF1B
ENSP00000223271	54	RARRES2
ENSP00000374069	54	PTPRN2
ENSP00000303554	54	PTPN9
ENSP00000375717	54	PTOV1
ENSP00000244043	54	PTGIS
ENSP00000216802	54	PSME2
ENSP00000377384	54	PSMC3IP

ENSP00000361547	54	PRRX2
ENSP00000331879	54	PRMT3
ENSP00000279575	54	PRB4
ENSP00000414202	54	PPP6R1
ENSP00000339933	54	PKLR
ENSP00000347032	54	PIP4K2C
ENSP00000281382	54	PIGF
ENSP00000367570	54	PHF11
ENSP00000417587	54	PEG10
ENSP00000300289	54	PDIA3
ENSP00000340836	54	OSTF1
ENSP00000261183	54	OSBPL8
ENSP00000394624	54	OPRM1
ENSP00000056233	54	NFE2L3
ENSP00000233840	54	NEU2
ENSP00000298925	54	NELL1
ENSP00000264596	54	NEIL3
ENSP00000383392	54	NCAM2
ENSP00000288235	54	MYO1E
ENSP00000391898	54	MOG
ENSP00000361064	54	MINPP1
ENSP00000352657	54	ME3
ENSP00000262858	54	MAMLD1
ENSP00000369251	54	MAB21L1
ENSP00000349629	54	LRBA
ENSP00000244289	54	LIPE
ENSP00000260753	54	KIF20B
ENSP00000362935	54	ITGB1BP2
ENSP00000341490	54	ISM2
ENSP00000243213	54	IL13RA2
ENSP00000369402	54	IFT74
ENSP00000370748	54	IDI1
ENSP00000264346	54	HERC6
ENSP00000341952	54	HAGHL
ENSP00000298173	54	GTF2A1
ENSP00000392398	54	GPX5
ENSP00000322731	54	GPR35
ENSP00000328818	54	GPR132
ENSP00000361483	54	GPR107
ENSP00000282541	54	GPD1L
ENSP00000370194	54	GMDS
ENSP00000329684	54	GALR2
ENSP00000380453	54	ERMN
ENSP00000355480	54	EFCAB2
ENSP00000357087	54	DUSP23
ENSP00000339850	54	DPYSL4

ENSP00000344277	54	DOK4
ENSP00000400258	54	DLGAP2
ENSP00000341351	54	DCAF5
ENSP00000338276	54	CRISP1
ENSP00000330060	54	CNOT10
ENSP00000263097	54	CNN2
ENSP00000258930	54	CIB2
ENSP00000260595	54	CGREF1
ENSP00000360540	54	CEP55
ENSP00000319166	54	CDH7
ENSP00000309376	54	CD164
ENSP00000364858	54	CARD16
ENSP00000288197	54	CACNA2D3
ENSP00000260442	54	BCL2L10
ENSP00000257861	54	AVIL
ENSP00000252725	54	ARPC1B
ENSP00000296557	54	ARFIP1
ENSP00000280155	54	ADRA2A
ENSP00000216254	54	ACO2
ENSP00000316746	54	-
ENSP00000328747	54	-
ENSP00000337113	54	-
ENSP00000342152	54	-
ENSP00000350459	54	-
ENSP00000369649	54	-
ENSP00000386047	54	-
ENSP00000308548	53	ZNHIT2
ENSP00000315664	53	ZNF18
ENSP00000292176	53	ZBTB7B
ENSP00000386840	53	XIRP2
ENSP00000263381	53	WIZ
ENSP00000369798	53	UCN3
ENSP00000360688	53	TXNDC12
ENSP00000356064	53	TULP4
ENSP00000355599	53	TSNAX
ENSP00000328835	53	TOP1MT
ENSP00000408958	53	TOM1L1
ENSP00000341838	53	TNNI3
ENSP00000296125	53	TGM4
ENSP00000360560	53	TCTE1
ENSP00000409932	53	TCEB3CL
ENSP00000288422	53	TAB3
ENSP00000323300	53	SPAG5
ENSP00000215730	53	SNAP29
ENSP00000233969	53	SLC9A2
ENSP00000357389	53	SLC50A1

ENSP00000248933	53	SEZ6L
ENSP00000377655	53	SENP7
ENSP00000350547	53	SEMA4F
ENSP00000332204	53	SEMA4B
ENSP00000322396	53	RRS1
ENSP00000346027	53	RPL21
ENSP00000332530	53	RNLS
ENSP00000358497	53	RNGTT
ENSP00000237455	53	RNF103
ENSP00000308219	53	RGMB
ENSP00000379154	53	RASGEF1A
ENSP00000350934	53	RABEP2
ENSP00000283228	53	PTPRR
ENSP00000387261	53	PSTPIP2
ENSP00000359286	53	PNMA3
ENSP00000011684	53	PLEKHG6
ENSP00000216446	53	PLEK2
ENSP00000321503	53	PGAM5
ENSP00000360305	53	PDLIM1
ENSP00000329403	53	PCP4
ENSP00000367362	53	PCDHA9
ENSP00000334008	53	PARVA
ENSP00000356634	53	PAPPA2
ENSP00000238633	53	NPC2
ENSP00000263257	53	NOVA2
ENSP00000304803	53	NKRF
ENSP00000416341	53	NELL2
ENSP00000373600	53	LRRK1
ENSP00000221459	53	LIN7B
ENSP00000337733	53	KLK5
ENSP00000360626	53	KCNG1
ENSP00000347276	53	KANK2
ENSP00000266041	53	ITIH4
ENSP00000371054	53	INSL6
ENSP00000325423	53	INPP1
ENSP00000313661	53	HTR1D
ENSP00000366898	53	HIF3A
ENSP00000246551	53	HCST
ENSP00000354003	53	GYPA
ENSP00000307900	53	GLUL
ENSP00000356410	53	GLRX2
ENSP00000355675	53	GJC2
ENSP00000276533	53	GINS4
ENSP00000352738	53	G3BP2
ENSP00000309823	53	FOXP4
ENSP00000299162	53	FOXN4

ENSP00000346240	53	FBXO2
ENSP00000356058	53	FAIM3
ENSP00000379383	53	ENAM
ENSP00000295822	53	EIF5A2
ENSP00000336741	53	DHX15
ENSP00000233078	53	DAZAP1
ENSP00000264723	53	CSPG5
ENSP00000354255	53	CPZ
ENSP00000261514	53	CLCN3
ENSP00000328336	53	CEND1
ENSP00000256447	53	CD180
ENSP00000225805	53	C17orf75
ENSP00000367705	53	BCOR
ENSP00000349325	53	ATP10A
ENSP00000246747	53	ARL2
ENSP00000314615	53	ARFGAP1
ENSP00000252490	53	APOC2
ENSP00000346921	53	AK2
ENSP00000306662	53	ADRA1B
ENSP00000264758	53	ADD1
ENSP00000252519	53	ACE2
ENSP00000393511	53	ABLIM2
ENSP00000319766	53	-
ENSP00000323148	52	ZKSCAN1
ENSP00000267294	52	ZIC5
ENSP00000353793	52	WDHD1
ENSP00000312981	52	USP39
ENSP00000261740	52	TRPV4
ENSP00000334962	52	TMPRSS6
ENSP00000381102	52	TMEM55B
ENSP00000262968	52	TJP3
ENSP00000266304	52	TEF
ENSP00000263974	52	TAF12
ENSP00000358531	52	SYCP1
ENSP00000302951	52	STRA13
ENSP00000377892	52	SRSF5
ENSP00000358770	52	SLK
ENSP00000266509	52	SLCO1C1
ENSP00000266088	52	SLC5A1
ENSP00000366797	52	SLC22A12
ENSP00000259608	52	SIT1
ENSP00000355141	52	SEPN1
ENSP00000357727	52	S100A9
ENSP00000377640	52	RPL24
ENSP00000380876	52	RGS11
ENSP00000258428	52	REV1

ENSP00000350491	52	PRIM1
ENSP00000365570	52	PRAF2
ENSP00000282406	52	PLEKHH2
ENSP00000369274	52	PHKA2
ENSP00000364145	52	PFKFB1
ENSP00000263666	52	PDZRN3
ENSP00000364597	52	PADI4
ENSP00000332170	52	P4HA3
ENSP00000307259	52	P2RY12
ENSP00000278780	52	OVOL2
ENSP00000318723	52	OSBPL6
ENSP00000357669	52	NPR1
ENSP00000367407	52	NMT2
ENSP00000261182	52	NAP1L1
ENSP00000226209	52	MYH3
ENSP00000262027	52	MARS
ENSP00000374443	52	LYST
ENSP00000321334	52	LPA
ENSP00000316881	52	LEPREL1
ENSP00000359557	52	LDOC1
ENSP00000357840	52	KIAA1919
ENSP00000221444	52	KCNA7
ENSP00000321345	52	IL23R
ENSP00000270800	52	IL22RA1
ENSP00000377197	52	HYDIN
ENSP00000364412	52	HSD17B3
ENSP00000346103	52	GPX4
ENSP00000265417	52	GPR116
ENSP00000341344	52	GGA1
ENSP00000281950	52	GEMIN6
ENSP00000309487	52	GCSAM
ENSP00000378890	52	GCH1
ENSP00000037243	52	GABARAPL2
ENSP00000252037	52	FKBP6
ENSP00000360262	52	FGGY
ENSP00000307298	52	FEM1B
ENSP00000268933	52	EPN3
ENSP00000352673	52	ELF3
ENSP00000265495	52	ELF2
ENSP00000401653	52	DRGX
ENSP00000307850	52	DRAP1
ENSP00000262593	52	DOK5
ENSP00000414402	52	DNAH14
ENSP00000337450	52	CYP3A7
ENSP00000296027	52	CXCL5
ENSP00000217131	52	CTSZ

ENSP00000356016	52	CR1
ENSP00000292907	52	COX7A1
ENSP00000261861	52	CORO2B
ENSP00000388340	52	CLINT1
ENSP00000284049	52	CHD1
ENSP00000359300	52	CETN2
ENSP00000337226	52	CDCA4
ENSP00000357156	52	CD5L
ENSP00000355192	52	CACNA1S
ENSP00000304065	52	C2orf27B
ENSP00000376992	52	BCL2L15
ENSP00000369858	52	ALOX5AP
ENSP00000380413	52	AGAP3
ENSP00000358301	52	ADRB1
ENSP00000273142	52	-
ENSP00000281525	52	-
ENSP00000300809	52	-
ENSP00000330180	52	-
ENSP00000333484	52	-
ENSP00000346850	52	-
ENSP00000379148	52	-
ENSP00000393457	52	-
ENSP00000403588	52	-
ENSP00000409842	52	-
ENSP00000355529	51	ZP4
ENSP00000227322	51	ZNF259
ENSP00000321406	51	ZNF135
ENSP00000257177	51	ZCCHC11
ENSP00000308759	51	ZBTB21
ENSP00000235090	51	WDR77
ENSP00000298767	51	WAPAL
ENSP00000295156	51	VSNL1
ENSP00000319501	51	UGDH
ENSP00000366006	51	UBIAD1
ENSP00000355343	51	UBAP2L
ENSP00000263525	51	TNR
ENSP00000245912	51	TNFSF14
ENSP00000285419	51	TMEM55A
ENSP00000362221	51	STK40
ENSP00000417583	51	ST6GALNAC5
ENSP00000361803	51	SMAP2
ENSP00000264047	51	SLC5A7
ENSP00000308895	51	SLC19A1
ENSP00000276373	51	SLC18A1
ENSP00000340237	51	SH3BP4
ENSP00000361867	51	SEMG1

ENSP00000297029	51	SCIN
ENSP00000362335	51	SAR1A
ENSP00000310356	51	RMI2
ENSP00000253571	51	RLIM
ENSP00000296273	51	RFC4
ENSP00000419944	51	PXT1
ENSP00000355308	51	PTPLA
ENSP00000262033	51	PTGES3
ENSP00000219313	51	PSMD7
ENSP00000262193	51	PSMB1
ENSP00000216968	51	PROCR
ENSP00000301788	51	POLR2G
ENSP00000317128	51	PLXND1
ENSP00000346228	51	PLA2G4C
ENSP00000286091	51	PDIA4
ENSP00000311713	51	OXSR1
ENSP00000263847	51	OSBP
ENSP00000260359	51	NUSAP1
ENSP00000378367	51	NRG4
ENSP00000367398	51	NPHP4
ENSP00000264790	51	MMRN1
ENSP00000370557	51	MIS12
ENSP00000282486	51	MBNL1
ENSP00000254898	51	MATN2
ENSP00000325313	51	MAP1S
ENSP00000344424	51	KIAA0247
ENSP00000369553	51	IFNA8
ENSP00000384432	51	ICOSLG
ENSP00000285667	51	HSPA13
ENSP00000280097	51	HNMT
ENSP00000355657	51	HIST3H3
ENSP00000261609	51	HERC2
ENSP00000282499	51	GRIA4
ENSP00000251808	51	GRHL2
ENSP00000333744	51	GPR19
ENSP00000365596	51	GPR183
ENSP00000343314	51	FXYD1
ENSP00000280481	51	FREM2
ENSP00000394487	51	FGD4
ENSP00000360538	51	FFAR4
ENSP00000217289	51	FERMT1
ENSP00000265299	51	FAM188B
ENSP00000377549	51	FABP6
ENSP00000376684	51	EPHB6
ENSP00000265162	51	ENPEP
ENSP00000306640	51	ELOVL5

ENSP00000384223	51	ECE2
ENSP00000303532	51	DEFB4A
ENSP00000321573	51	DCBLD2
ENSP00000221700	51	CYP4F2
ENSP00000340937	51	COL17A1
ENSP00000266546	51	CLSTN3
ENSP00000259938	51	CLPS
ENSP00000362330	51	CHD6
ENSP00000357025	51	CD48
ENSP00000357152	51	CD1C
ENSP00000287097	51	CD109
ENSP00000374507	51	CCDC88C
ENSP00000262653	51	CBFA2T2
ENSP00000285381	51	CA3
ENSP00000293805	51	BCL6B
ENSP00000392330	51	BCAP31
ENSP00000344087	51	AUTS2
ENSP00000356379	51	ASPM
ENSP00000368332	51	ARX
ENSP00000352918	51	ARPC5
ENSP00000270747	51	ARHGEF19
ENSP00000252491	51	APOC1
ENSP00000306678	51	ANKK1
ENSP00000318775	51	ANAPC4
ENSP00000278723	51	-
ENSP00000317618	51	-
ENSP00000376704	51	-
ENSP00000404231	51	-
ENSP00000386878	50	WIPF3
ENSP00000357651	50	TUBE1
ENSP00000260116	50	TTPA
ENSP00000259750	50	TTBK1
ENSP00000057513	50	TNIP3
ENSP00000365435	50	TNFRSF1B
ENSP00000308925	50	TLR10
ENSP00000261716	50	TAOK1
ENSP00000315630	50	SWAP70
ENSP00000295770	50	STT3B
ENSP00000263955	50	STK17B
ENSP00000379964	50	STK16
ENSP00000230085	50	SNX3
ENSP00000233575	50	SNX17
ENSP00000195649	50	SNAP91
ENSP00000254976	50	SNAP25
ENSP00000341382	50	SMC4
ENSP00000352028	50	SH3RF2

ENSP00000350447	50	SGOL2
ENSP00000246115	50	S1PR4
ENSP00000369257	50	RPGRIP1L
ENSP00000301459	50	RCOR2
ENSP00000309166	50	RBM4
ENSP00000365773	50	PSAT1
ENSP00000303775	50	PROKR1
ENSP00000361310	50	POLH
ENSP00000216367	50	POLE2
ENSP00000286063	50	PDE11A
ENSP00000381740	50	PARP3
ENSP00000363643	50	P2RY4
ENSP00000342278	50	OAS2
ENSP00000262077	50	NUP153
ENSP00000290401	50	NPTN
ENSP00000352839	50	NPSR1
ENSP00000299964	50	NNMT
ENSP00000372857	50	NEK11
ENSP00000347170	50	NEIL1
ENSP00000263354	50	NAPA
ENSP00000391106	50	MYO10
ENSP00000371577	50	MTMR12
ENSP00000364894	50	MSH5
ENSP00000352513	50	MPZL1
ENSP00000388137	50	MPPED1
ENSP00000255977	50	MKRN1
ENSP00000262843	50	MID2
ENSP00000268607	50	MAP1LC3B
ENSP00000307445	50	LTB4R
ENSP00000338727	50	LRRFIP2
ENSP00000309521	50	LIME1
ENSP00000353893	50	LGALS14
ENSP00000324020	50	KIF15
ENSP00000348108	50	KHDRBS3
ENSP00000271915	50	KCNN3
ENSP00000328511	50	KCNA4
ENSP00000305200	50	IL1RAPL1
ENSP00000398971	50	IFRD2
ENSP00000414109	50	HJURP
ENSP00000268097	50	HEXA
ENSP00000254799	50	GRSF1
ENSP00000284674	50	GPR26
ENSP00000303942	50	GP9
ENSP00000218516	50	GLA
ENSP00000265857	50	GET4
ENSP00000254466	50	GAS2L2

ENSP00000363317	50	FRMPD2
ENSP00000379369	50	FOXB1
ENSP00000266087	50	FBXO7
ENSP00000374218	50	ESYT3
ENSP00000417229	50	EIF2A
ENSP00000320516	50	EHD1
ENSP00000361787	50	DUSP13
ENSP00000272444	50	DUSP11
ENSP00000359396	50	DPPA5
ENSP00000337572	50	DGKH
ENSP00000263576	50	DDX25
ENSP00000349768	50	DCHS2
ENSP00000349790	50	COL9A1
ENSP00000298527	50	CLEC1B
ENSP00000346566	50	CKAP5
ENSP00000315477	50	CD209
ENSP00000297979	50	C9orf3
ENSP00000345824	50	BZRAP1
ENSP00000265016	50	BST1
ENSP00000368754	50	BAZ2A
ENSP00000286639	50	BATF
ENSP00000224652	50	ATE1
ENSP00000389003	50	ASPG
ENSP00000228825	50	ARPC3
ENSP00000353210	50	ARL9
ENSP00000375057	50	ARL4C
ENSP00000366121	50	ARHGAP27
ENSP00000305464	50	APLN
ENSP00000370713	50	ADARB2
ENSP00000344847	50	ADAMTS12
ENSP00000309477	50	ACO1
ENSP00000311496	50	10-Mar
ENSP00000294419	50	-
ENSP00000301453	50	-
ENSP00000302994	50	-
ENSP00000341308	50	-
ENSP00000364361	50	-
ENSP00000384903	50	-
ENSP00000390786	50	-
ENSP00000305804	49	ZNF131
ENSP00000267199	49	VPS33A
ENSP00000345195	49	UBQLN2
ENSP00000263798	49	TYRO3
ENSP00000357842	49	TUFT1
ENSP00000284551	49	TRIM11
ENSP00000299697	49	TK2

ENSP00000401548	49	TCF19
ENSP00000244625	49	TBCC
ENSP00000365962	49	TBC1D25
ENSP00000305899	49	SUV420H1
ENSP00000360992	49	STAMBPL1
ENSP00000317790	49	SPTBN5
ENSP00000355354	49	SOX15
ENSP00000222990	49	SNX8
ENSP00000249647	49	SNAP23
ENSP00000253122	49	SLC6A8
ENSP00000360398	49	SLC25A27
ENSP00000221742	49	SLC1A6
ENSP00000346112	49	SLC12A6
ENSP00000302913	49	SH3D19
ENSP00000398930	49	SGCE
ENSP00000337632	49	SARNP
ENSP00000369218	49	RBM17
ENSP00000304336	49	PYDC1
ENSP00000318602	49	PRTFDC1
ENSP00000317836	49	PRR15
ENSP00000254101	49	PRKAB2
ENSP00000260970	49	PPIG
ENSP00000357051	49	PEX19
ENSP00000360672	49	PARD6B
ENSP00000370019	49	PAQR3
ENSP00000310305	49	P2RY2
ENSP00000336607	49	P2RX4
ENSP00000337452	49	NIPA1
ENSP00000325285	49	MTMR4
ENSP00000222644	49	MPP6
ENSP00000304553	49	MPLKIP
ENSP00000354850	49	MGEA5
ENSP00000256379	49	MED6
ENSP00000348610	49	MED24
ENSP00000363079	49	MBL2
ENSP00000350195	49	MAP3K6
ENSP00000272163	49	LBR
ENSP00000310861	49	KRT2
ENSP00000229402	49	KLRB1
ENSP00000375608	49	KIR3DL1
ENSP00000378356	49	KIF20A
ENSP00000245787	49	INSIG2
ENSP00000314976	49	IL20RA
ENSP00000374779	49	IGKV5-2
ENSP00000374985	49	IGHG4
ENSP00000374998	49	IGHD

ENSP00000253680	49	HSH2D
ENSP00000366620	49	H6PD
ENSP00000274306	49	GZMA
ENSP00000221130	49	GSR
ENSP00000293662	49	GRASP
ENSP00000275169	49	GPR6
ENSP00000366157	49	GPR180
ENSP00000356581	49	GPR126
ENSP00000334940	49	GGN
ENSP00000367848	49	GABRD
ENSP00000359804	49	FUBP1
ENSP00000336914	49	FGD3
ENSP00000349078	49	FDPS
ENSP00000403396	49	FAM126A
ENSP00000362978	49	EYA3
ENSP00000260762	49	EXOC6
ENSP00000389934	49	EXOC5
ENSP00000201647	49	EPS8L1
ENSP00000377566	49	ELMO3
ENSP00000357535	49	ECHS1
ENSP00000382064	49	DTNA
ENSP00000345229	49	DNER
ENSP00000316938	49	DNASE1L2
ENSP00000014935	49	DNASE1L1
ENSP00000230895	49	DAP
ENSP00000298248	49	CRYL1
ENSP00000261401	49	CORO1C
ENSP00000359285	49	CHRNA4
ENSP00000311847	49	CARHSP1
ENSP00000376177	49	CALCRL
ENSP00000314407	49	CA8
ENSP00000278601	49	C11orf52
ENSP00000380598	49	BLOC1S5
ENSP00000215115	49	BCL7C
ENSP00000264649	49	ATP6V0A1
ENSP00000374424	49	ARHGAP8
ENSP00000249601	49	ARHGAP22
ENSP00000295095	49	ARHGAP15
ENSP00000399341	49	API5
ENSP00000315931	49	AHCYL2
ENSP00000209665	49	ADH7
ENSP00000265727	49	ADAM22
ENSP00000273145	49	ACKR2
ENSP00000246911	49	-
ENSP00000368347	49	-
ENSP00000369144	49	-

ENSP00000375049	49	-
ENSP00000311596	48	ZNF699
ENSP00000354206	48	ZNF219
ENSP00000361845	48	ZMPSTE24
ENSP00000372553	48	ZIC4
ENSP00000349803	48	ZBTB20
ENSP00000351137	48	XAB2
ENSP00000346986	48	WAC
ENSP00000262709	48	TOX4
ENSP00000354842	48	TOX
ENSP00000314733	48	TOLLIP
ENSP00000316021	48	TMEM256-PLSCR3
ENSP00000272643	48	THSD7B
ENSP00000260129	48	TGS1
ENSP00000376317	48	TAOK3
ENSP00000348278	48	STK39
ENSP00000372689	48	STAG1
ENSP00000354774	48	SPRYD7
ENSP00000365931	48	SP2
ENSP00000296088	48	SNRK
ENSP00000307252	48	SMOX
ENSP00000218197	48	SLC25A14
ENSP00000278222	48	SAA4
ENSP00000328472	48	S1PR5
ENSP00000302896	48	RPS9
ENSP00000321805	48	RIT2
ENSP00000264839	48	RIMS1
ENSP00000345144	48	RCBTB2
ENSP00000261336	48	PZP
ENSP00000233944	48	PRKAG3
ENSP00000260643	48	PREB
ENSP00000284601	48	PPP1R3A
ENSP00000303754	48	PPID
ENSP00000217971	48	PGRMC1
ENSP00000252854	48	OLFM1
ENSP00000338766	48	NPHP3
ENSP00000269228	48	NPC1
ENSP00000199447	48	NME8
ENSP00000361596	48	NEURL2
ENSP00000251496	48	NCAPG
ENSP00000325017	48	NCAPD2
ENSP00000292327	48	MYL3
ENSP00000381008	48	MUC16
ENSP00000355046	48	MT-ND2
ENSP00000255087	48	MTL5
ENSP00000219431	48	MPG

ENSP00000228506	48	MLEC
ENSP00000368798	48	MBTPS2
ENSP00000302830	48	MAEA
ENSP00000363752	48	LUZP1
ENSP00000299022	48	LIPC
ENSP00000362227	48	L3MBTL1
ENSP00000362728	48	KPNA6
ENSP00000394085	48	JMJD6
ENSP00000273221	48	IQSEC1
ENSP00000318641	48	INTS3
ENSP00000013222	48	INMT
ENSP00000260049	48	IL18BP
ENSP00000374851	48	IGLV3-9
ENSP00000342560	48	HRH3
ENSP00000308610	48	GPD2
ENSP00000220822	48	GDAP1
ENSP00000359504	48	GBP1
ENSP00000388035	48	GABBR1
ENSP00000261942	48	FAF2
ENSP00000315476	48	EXOSC4
ENSP00000263713	48	EPB41L5
ENSP00000250454	48	EAPP
ENSP00000304802	48	DTYMK
ENSP00000365397	48	DHRS3
ENSP00000227266	48	CTSC
ENSP00000311733	48	CMKLR1
ENSP00000316228	48	CLEC4M
ENSP00000365256	48	CLCN5
ENSP00000265593	48	CLCN2
ENSP00000357461	48	CHRNB2
ENSP00000217402	48	CHMP4B
ENSP00000247153	48	CFP
ENSP00000217372	48	CDK5RAP1
ENSP00000283285	48	CD96
ENSP00000331172	48	CD8B
ENSP00000415822	48	ATXN1L
ENSP00000316854	48	ATOX1
ENSP00000345808	48	ARHGAP12
ENSP00000318770	48	AQP11
ENSP00000354432	48	ANKRD30A
ENSP00000308549	48	ADORA1
ENSP00000216139	48	ACR
ENSP00000218758	48	ACP5
ENSP00000217455	48	ACOT8
ENSP00000374467	48	ABCC8
ENSP00000285238	48	ABCC3

ENSP00000268509	48	-
ENSP00000380364	48	-
ENSP00000388732	48	-
ENSP00000389863	48	-
ENSP00000325393	47	ZNF559
ENSP00000262291	47	VMP1
ENSP00000381577	47	USP34
ENSP00000285679	47	USP25
ENSP00000348708	47	UPF2
ENSP00000340305	47	UBE2V1
ENSP00000333326	47	TUBA8
ENSP00000326042	47	TUBA3D
ENSP00000367743	47	TSPAN7
ENSP00000362387	47	TSPAN15
ENSP00000166345	47	TRIP13
ENSP00000291574	47	TRAPPC10
ENSP00000368818	47	TOM1L2
ENSP00000256062	47	TMTC1
ENSP00000280734	47	TMEM86A
ENSP00000367406	47	TAF2
ENSP00000342538	47	STMN4
ENSP00000305958	47	STIP1
ENSP00000378418	47	SP9
ENSP00000346762	47	SND1
ENSP00000302851	47	SLC23A1
ENSP00000262461	47	SLC12A2
ENSP00000298841	47	SERPINA4
ENSP00000402527	47	SENP6
ENSP00000268099	47	SCAMP2
ENSP00000262173	47	RNMT
ENSP00000376585	47	RFX4
ENSP00000351697	47	REV3L
ENSP00000267568	47	PTGR2
ENSP00000362334	47	PSMB2
ENSP00000344413	47	PSG5
ENSP00000271620	47	PRUNE
ENSP00000295619	47	PROK2
ENSP00000323867	47	PRKAG1
ENSP00000371264	47	PRB3
ENSP00000268261	47	PMM2
ENSP00000338707	47	PLSCR2
ENSP00000305556	47	PCBP1
ENSP00000325618	47	PARP10
ENSP00000341422	47	P4HTM
ENSP00000265572	47	OPRK1
ENSP00000239690	47	NUDCD1

ENSP00000320168	47	NUCB2
ENSP00000223140	47	NOBOX
ENSP00000264218	47	NMU
ENSP00000295624	47	NAV1
ENSP00000382193	47	MYBPC3
ENSP00000360498	47	MRPL41
ENSP00000198801	47	MOGAT2
ENSP00000219596	47	MEFV
ENSP00000389128	47	LRRC15
ENSP00000301420	47	KLK1
ENSP00000387875	47	KIF4B
ENSP00000283936	47	KCNJ16
ENSP00000374777	47	IGKC
ENSP00000253513	47	IDO1
ENSP00000307766	47	HTR1E
ENSP00000338477	47	HNRNPF
ENSP00000359454	47	HFM1
ENSP00000312002	47	HAVCR2
ENSP00000348168	47	GTF2E2
ENSP00000364180	47	GPSM3
ENSP00000300098	47	GPR182
ENSP00000308622	47	GLE1
ENSP00000285873	47	GEMIN5
ENSP00000192314	47	GAL3ST2
ENSP00000241502	47	FYTTD1
ENSP00000342858	47	FERMT2
ENSP00000246069	47	DSTN
ENSP00000273814	47	DGKQ
ENSP00000367454	47	DFFB
ENSP00000404968	47	DAZAP2
ENSP00000375783	47	DACT3
ENSP00000359629	47	CRTAC1
ENSP00000317780	47	COX5A
ENSP00000361290	47	COL4A6
ENSP00000380602	47	CNTN4
ENSP00000317842	47	CES2
ENSP00000368450	47	CD83
ENSP00000375086	47	CCL25
ENSP00000253079	47	CCDC62
ENSP00000382708	47	C5orf49
ENSP00000350914	47	BDH1
ENSP00000218147	47	BCORL1
ENSP00000259407	47	BAAT
ENSP00000350078	47	ARID5A
ENSP00000349022	47	ARC
ENSP00000312442	47	AP1G2

ENSP00000394394	47	ANAPC7
ENSP00000315167	47	ALOX12B
ENSP00000339787	47	ACSL4
ENSP00000409612	47	ACADM
ENSP00000215555	47	2-Mar
ENSP00000023165	47	-
ENSP00000222270	47	-
ENSP00000292253	47	-
ENSP00000296977	47	-
ENSP00000328808	47	-
ENSP00000348697	47	-
ENSP00000374992	47	-
ENSP00000351530	46	ZXDA
ENSP00000394757	46	ZNF516
ENSP00000341497	46	ZNF177
ENSP00000282869	46	ZNF117
ENSP00000412932	46	ZFP57
ENSP00000270288	46	WTIP
ENSP00000327650	46	VPS33B
ENSP00000357556	46	VENTX
ENSP00000236192	46	VAMP4
ENSP00000215794	46	USP18
ENSP00000411698	46	USO1
ENSP00000330032	46	UPP1
ENSP00000264552	46	UBE2S
ENSP00000264071	46	TUBB4A
ENSP00000265310	46	TRPV5
ENSP00000266712	46	TMTC3
ENSP00000344166	46	TMEM189-UBE2V1
ENSP00000359449	46	TMEM185A
ENSP00000360171	46	TENM1
ENSP00000380779	46	TCTN1
ENSP00000265686	46	TCIRG1
ENSP00000194530	46	STRADB
ENSP00000311493	46	STON1-GTF2A1L
ENSP00000320754	46	STK33
ENSP00000340279	46	SSX2IP
ENSP00000248701	46	SPINK2
ENSP00000204566	46	SPG21
ENSP00000260197	46	SORL1
ENSP00000305133	46	SOCS5
ENSP00000373637	46	SMYD3
ENSP00000236877	46	SLC8A2
ENSP00000289932	46	SLC5A11
ENSP00000286298	46	SLC26A2
ENSP00000337335	46	SLC22A8

ENSP00000370839	46	SGCB
ENSP00000282030	46	SETBP1
ENSP00000337212	46	SERPINB7
ENSP00000324857	46	SEMA6D
ENSP00000264896	46	SCARB2
ENSP00000317224	46	SAMD4B
ENSP00000385328	46	RNF43
ENSP00000328287	46	RNF123
ENSP00000296859	46	RAPGEF6
ENSP00000281243	46	QDPR
ENSP00000328325	46	PTTG1IP
ENSP00000351342	46	PTPN5
ENSP00000270586	46	PSMB6
ENSP00000355325	46	PSMB5
ENSP00000303300	46	PRY2
ENSP00000165524	46	PRLH
ENSP00000343924	46	PRELP
ENSP00000300026	46	PPIB
ENSP00000258679	46	PLEKHA8
ENSP00000244137	46	PEPD
ENSP00000219406	46	PDIA2
ENSP00000231484	46	PCDH12
ENSP00000234040	46	PASK
ENSP00000409493	46	OBSCN
ENSP00000384551	46	NXPH1
ENSP00000337443	46	NXN
ENSP00000310205	46	NRIP3
ENSP00000375063	46	NLRP5
ENSP00000313881	46	MKRN3
ENSP00000170447	46	MKRN2
ENSP00000377778	46	MIEN1
ENSP00000334219	46	MEGF8
ENSP00000326767	46	MED25
ENSP00000414634	46	LSM2
ENSP00000353538	46	LRRCC1
ENSP00000035383	46	LRRC7
ENSP00000382204	46	JMJD1C
ENSP00000293756	46	IP6K3
ENSP00000368872	46	INSC
ENSP00000329384	46	IL22
ENSP00000264009	46	HSF4
ENSP00000318631	46	HSD17B6
ENSP00000252825	46	HRC
ENSP00000362566	46	HPCA
ENSP00000355541	46	HEATR1
ENSP00000349493	46	GUCA2A

ENSP00000264720	46	GTF3C2
ENSP00000360302	46	GRIA3
ENSP00000292377	46	GPC2
ENSP00000258145	46	GNS
ENSP00000349687	46	GM2A
ENSP00000340811	46	GJB5
ENSP00000340191	46	FPR2
ENSP00000341961	46	FOXD4L3
ENSP00000257515	46	FAM189A2
ENSP00000320503	46	EDC3
ENSP00000221418	46	ECH1
ENSP00000401566	46	ECD
ENSP00000294485	46	DRAXIN
ENSP00000344432	46	DOK7
ENSP00000353104	46	DHDDS
ENSP00000265022	46	DGKG
ENSP00000319831	46	DEF6
ENSP00000371234	46	CYS1
ENSP00000306884	46	CXCL11
ENSP00000357981	46	CTSS
ENSP00000359664	46	CTBS
ENSP00000418348	46	CMC1
ENSP00000338258	46	CDC42EP4
ENSP00000378350	46	CDC23
ENSP00000363330	46	CD52
ENSP00000290349	46	CBR1
ENSP00000344549	46	CARD14
ENSP00000247461	46	CANX
ENSP00000354536	46	ASTN1
ENSP00000321388	46	ASB4
ENSP00000327459	46	APOBEC3B
ENSP00000321679	46	ANKRD23
ENSP00000262374	46	ALG1
ENSP00000346827	46	ALDH9A1
ENSP00000362249	46	AK1
ENSP00000247291	46	AIF1L
ENSP00000378359	46	ADH6
ENSP00000262710	46	ACIN1
ENSP00000281189	46	-
ENSP00000299233	46	-
ENSP00000341187	46	-
ENSP00000342804	46	-
ENSP00000349140	46	-
ENSP00000368046	46	-
ENSP00000397121	46	-
ENSP00000402672	46	-

ENSP00000407950	46	-
ENSP00000357565	45	ZUFSP
ENSP00000326630	45	ZFPM1
ENSP00000282388	45	ZFP36L2
ENSP00000246914	45	WNK4
ENSP00000356425	45	UCHL5
ENSP00000361990	45	UBR2
ENSP00000217515	45	TXNL1
ENSP00000320797	45	TUBD1
ENSP00000401317	45	TUBB
ENSP00000382982	45	TUBA3C
ENSP00000325518	45	TSTD1
ENSP00000262839	45	TRPC5
ENSP00000368966	45	TRPC3
ENSP00000234798	45	TPSG1
ENSP00000252487	45	TOMM40
ENSP00000370867	45	TGM3
ENSP00000340995	45	TEX11
ENSP00000334294	45	TCFL5
ENSP00000256366	45	SYNJ2BP
ENSP00000355649	45	SPHAR
ENSP00000267814	45	SORD
ENSP00000265909	45	SNX19
ENSP00000378965	45	SNTB1
ENSP00000357754	45	SMCP
ENSP00000325827	45	SEC16A
ENSP00000300175	45	SCG5
ENSP00000371040	45	RLN2
ENSP00000330005	45	RGMA
ENSP00000395449	45	RECQL
ENSP00000258646	45	RCBTB1
ENSP00000275423	45	RBAK
ENSP00000376899	45	PTGFRN
ENSP00000309474	45	PSMD1
ENSP00000295984	45	PRRT3
ENSP00000257860	45	PRPH
ENSP00000282412	45	PPM1B
ENSP00000360782	45	PMPCA
ENSP00000332225	45	PMCH
ENSP00000323194	45	PLXNA4
ENSP00000282903	45	PLOD2
ENSP00000320017	45	PHYHIP
ENSP00000217305	45	PDYN
ENSP00000297598	45	PDP1
ENSP00000394282	45	OR4F3
ENSP00000356110	45	NUCKS1

ENSP00000338349	45	NRXN3
ENSP00000240423	45	NCAPH
ENSP00000371607	45	MIPEP
ENSP00000327875	45	LPAR5
ENSP00000341947	45	LIN54
ENSP00000315997	45	LILRB1
ENSP00000251390	45	LILRA3
ENSP00000338130	45	KLRD1
ENSP00000310878	45	KLF14
ENSP00000346453	45	IPO4
ENSP00000374987	45	IGHG2
ENSP00000305632	45	HOMER2
ENSP00000372975	45	HLA-C
ENSP00000343464	45	HELT
ENSP00000354677	45	GPX7
ENSP00000355146	45	GPRASP1
ENSP00000316861	45	GPM6B
ENSP00000261837	45	GNB5
ENSP00000203556	45	GMIP
ENSP00000248923	45	GGT1
ENSP00000268699	45	GAS8
ENSP00000266458	45	GABARAPL1
ENSP00000279227	45	FERMT3
ENSP00000347714	45	FCAR
ENSP00000313034	45	FBXW5
ENSP00000387040	45	FAT3
ENSP00000329499	45	FAM187A
ENSP00000225371	45	EPX
ENSP00000409382	45	EFCAB4B
ENSP00000352785	45	DSG4
ENSP00000336725	45	DDX3Y
ENSP00000360317	45	CYP2C8
ENSP00000338461	45	CYB5R3
ENSP00000370966	45	CRYZL1
ENSP00000357779	45	CRCT1
ENSP00000268717	45	COPS3
ENSP00000250378	45	CMA1
ENSP00000353500	45	CLEC4C
ENSP00000255380	45	CHRM3
ENSP00000303058	45	CEP120
ENSP00000388794	45	CD177
ENSP00000332504	45	CCR10
ENSP00000225245	45	CCL3
ENSP00000278559	45	CAPN5
ENSP00000256119	45	CA1
ENSP00000360210	45	C20orf85

ENSP00000265990	45	BTAF1
ENSP00000376946	45	BBS10
ENSP00000360776	45	B4GALT5
ENSP00000256496	45	ARL8B
ENSP00000320247	45	AQP6
ENSP00000315136	45	APBA3
ENSP00000382379	45	ANKRD28
ENSP00000393996	45	AIF1
ENSP00000356067	45	-
ENSP00000398263	45	-
ENSP00000411633	45	-
ENSP00000411943	45	-
ENSP00000352676	44	ZRANB1
ENSP00000314619	44	ZFAND2A
ENSP00000272321	44	WDPCP
ENSP00000261405	44	VWF
ENSP00000285279	44	VOPP1
ENSP00000337040	44	UNC119
ENSP00000263202	44	UFD1L
ENSP00000321987	44	TSPEAR
ENSP00000296098	44	TRIM54
ENSP00000319992	44	TMEM11
ENSP00000337353	44	TDP1
ENSP00000357794	44	TCHH
ENSP00000312709	44	TAF7
ENSP00000316464	44	SYTL1
ENSP00000363391	44	SPAG4
ENSP00000350024	44	SLC29A2
ENSP00000306473	44	SHCBP1
ENSP00000262018	44	SGCA
ENSP00000360034	44	SERBP1
ENSP00000347329	44	SEC31A
ENSP00000407460	44	SCRN1
ENSP00000359221	44	RWDD3
ENSP00000371471	44	RSAD2
ENSP00000319210	44	RPH3AL
ENSP00000354571	44	RNF121
ENSP00000261655	44	RIMBP2
ENSP00000304311	44	REG3A
ENSP00000341243	44	RBPJL
ENSP00000294904	44	RBMS1
ENSP00000386520	44	RAD54L2
ENSP00000356991	44	PVRL4
ENSP00000403852	44	POLR2F
ENSP00000358242	44	PNISR
ENSP00000303511	44	PEX6

ENSP00000379485	44	PDE1C
ENSP00000217386	44	OXT
ENSP00000360621	44	ORC1
ENSP00000346659	44	OCA2
ENSP00000245552	44	NT5C
ENSP00000398028	44	NPR3
ENSP00000366061	44	NMS
ENSP00000238616	44	NEK9
ENSP00000237500	44	MYL12B
ENSP00000262873	44	MYH7B
ENSP00000261758	44	MESDC2
ENSP00000354346	44	MATR3
ENSP00000320043	44	LYPLA1
ENSP00000209884	44	KLHL20
ENSP00000385545	44	KIF26B
ENSP00000394033	44	KCNK2
ENSP00000331698	44	KCNJ15
ENSP00000295225	44	KCNIP3
ENSP00000354742	44	IPO9
ENSP00000381064	44	INTS10
ENSP00000264260	44	IL18RAP
ENSP00000375025	44	IGHV4-34
ENSP00000304331	44	GSX1
ENSP00000299092	44	GPR176
ENSP00000370844	44	GPR12
ENSP00000313869	44	GORASP1
ENSP00000248150	44	GNG13
ENSP00000381339	44	GNAT3
ENSP00000250113	44	FXR2
ENSP00000298542	44	FRMD7
ENSP00000354620	44	FOXJ3
ENSP00000403293	44	FBXO43
ENSP00000337510	44	FBXO40
ENSP00000321951	44	FAIM2
ENSP00000339897	44	EME1
ENSP00000353575	44	EIF2B3
ENSP00000380308	44	DYNC1I2
ENSP00000294618	44	DOCK6
ENSP00000264093	44	DGUOK
ENSP00000265728	44	DBF4
ENSP00000341692	44	DAP3
ENSP00000308032	44	CYP2S1
ENSP00000348380	44	CYP20A1
ENSP00000284878	44	CXADR
ENSP00000282141	44	CRYGC
ENSP00000315013	44	CRAT

ENSP00000300527	44	COL6A2
ENSP00000325146	44	COL12A1
ENSP00000272133	44	CNIH3
ENSP00000278935	44	CEP164
ENSP00000328228	44	CDCA2
ENSP00000358490	44	CD2
ENSP00000320025	44	CACNB2
ENSP00000263413	44	C6
ENSP00000322582	44	C4orf17
ENSP00000361577	44	C10orf11
ENSP00000298295	44	C10orf10
ENSP00000331210	44	BCAN
ENSP00000332979	44	BACE2
ENSP00000339016	44	ATAD1
ENSP00000350331	44	ASB13
ENSP00000297988	44	AQP7
ENSP00000217456	44	APMAP
ENSP00000250244	44	AP1M2
ENSP00000349588	44	ANK2
ENSP00000280979	44	AKAP6
ENSP00000353920	44	ADARB1
ENSP00000310309	44	ABLIM3
ENSP00000303844	44	-
ENSP00000309431	44	-
ENSP00000323777	44	-
ENSP00000329964	44	-
ENSP00000351411	44	-
ENSP00000374949	44	-
ENSP00000381748	44	-
ENSP00000409384	44	-
ENSP00000412971	44	-
ENSP00000278853	43	ZP1
ENSP00000260187	43	USP2
ENSP00000294119	43	UBXN1
ENSP00000307863	43	U2AF2
ENSP00000318197	43	TUBA3E
ENSP00000395598	43	TRIM10
ENSP00000244709	43	TREM1
ENSP00000271583	43	TOR1AIP1
ENSP00000362122	43	TNMD
ENSP00000339917	43	TAF9B
ENSP00000263918	43	STRN
ENSP00000234454	43	SPR
ENSP00000282074	43	SPC25
ENSP00000227135	43	SPA17
ENSP00000369210	43	SOHLH2

ENSP00000300413	43	SNRPD1
ENSP00000369887	43	SLTM
ENSP00000349174	43	SLC39A8
ENSP00000248929	43	SGSM3
ENSP00000307599	43	SCN11A
ENSP00000266214	43	SCARF2
ENSP00000368572	43	SAT1
ENSP00000303248	43	RXFP1
ENSP00000346050	43	RPS3A
ENSP00000328340	43	RNF135
ENSP00000361942	43	RIMS4
ENSP00000386229	43	RGS14
ENSP00000340578	43	RASSF6
ENSP00000401802	43	PSMC6
ENSP00000225426	43	PSMB3
ENSP00000291281	43	PRKD2
ENSP00000252455	43	PRKCSH
ENSP00000353415	43	PRKAR1B
ENSP00000356156	43	PPP1R15B
ENSP00000253925	43	PPFIA1
ENSP00000252590	43	PLVAP
ENSP00000350132	43	PLN
ENSP00000362123	43	PLA2G12B
ENSP00000298282	43	PKNOX2
ENSP00000325296	43	PKD2L1
ENSP00000341805	43	PHF10
ENSP00000297283	43	PGAM2
ENSP00000356047	43	PFKFB2
ENSP00000315680	43	PEX7
ENSP00000223061	43	PCOLCE
ENSP00000252087	43	PCDHGC5
ENSP00000352438	43	PCBP2
ENSP00000367462	43	OLAH
ENSP00000325663	43	NFKBIZ
ENSP00000363985	43	MTMR8
ENSP00000310631	43	MRGPRD
ENSP00000216075	43	MIOX
ENSP00000268150	43	MFGE8
ENSP00000383894	43	MATN3
ENSP00000318604	43	MAF1
ENSP00000390120	43	LILRA6
ENSP00000355296	43	LDB3
ENSP00000317566	43	LCORL
ENSP00000367203	43	KDM6A
ENSP00000311202	43	KCTD13
ENSP00000355011	43	ILF2

ENSP00000374844	43	IGLV3-19
ENSP00000370125	43	HMGN1
ENSP00000330601	43	HHIPL1
ENSP00000384597	43	GTF2A1L
ENSP00000363296	43	GRM4
ENSP00000282753	43	GRM1
ENSP00000377192	43	G6PD
ENSP00000269097	43	G6PC3
ENSP00000371940	43	FOXD4
ENSP00000312017	43	FAM57A
ENSP00000356481	43	FAM129A
ENSP00000358456	43	F8A1
ENSP00000347213	43	ENTPD2
ENSP00000341658	43	DUSP15
ENSP00000288699	43	DPYSL5
ENSP00000252268	43	DPF2
ENSP00000220496	43	DNAJC17
ENSP00000221307	43	CYP4F3
ENSP00000363840	43	COL11A2
ENSP00000263665	43	CNTN3
ENSP00000273986	43	CISD2
ENSP00000350386	43	CHM
ENSP00000357311	43	CENPW
ENSP00000215980	43	CENPM
ENSP00000357150	43	CD1B
ENSP00000362704	43	CCDC28B
ENSP00000365401	43	CCDC22
ENSP00000356652	43	CACYBP
ENSP00000360281	43	C8B
ENSP00000376823	43	C7orf49
ENSP00000281474	43	BICD1
ENSP00000360899	43	BEND5
ENSP00000242067	43	BBS9
ENSP00000307875	43	B3GAT1
ENSP00000353072	43	ATP2A3
ENSP00000354708	43	ARMCX6
ENSP00000272217	43	ARL8A
ENSP00000367629	43	ARHGEF16
ENSP00000199280	43	AQP2
ENSP00000314004	43	ANAPC2
ENSP00000375881	43	ALPP
ENSP00000336927	43	ALDOA
ENSP00000235835	43	AKR7A2
ENSP00000265846	43	ADAP1
ENSP00000175238	43	ADAM7
ENSP00000361965	43	ADA

ENSP00000228245	43	-
ENSP00000303623	43	-
ENSP00000311232	43	-
ENSP00000348401	43	-
ENSP00000383670	43	-
ENSP00000391386	43	-
ENSP00000400953	43	-
ENSP00000404553	43	-
ENSP00000408457	43	-
ENSP00000420820	43	-
ENSP00000363055	42	ZWINT
ENSP00000362763	42	ZBTB6
ENSP00000351346	42	VPS13B
ENSP00000264234	42	UPK1B
ENSP00000229708	42	ULBP1
ENSP00000362525	42	UGT1A7
ENSP00000356243	42	UBE2T
ENSP00000317327	42	UBASH3A
ENSP00000260383	42	TUBGCP4
ENSP00000352358	42	TRPV6
ENSP00000362205	42	TREM2
ENSP00000232975	42	TNNC1
ENSP00000350965	42	TMBIM4
ENSP00000281030	42	THRSP
ENSP00000202625	42	TGM6
ENSP00000331815	42	TBL3
ENSP00000255224	42	SYT4
ENSP00000319622	42	SULT1C2
ENSP00000356918	42	STX7
ENSP00000156626	42	ST6GALNAC1
ENSP00000362095	42	SRPX2
ENSP00000216484	42	SPTLC2
ENSP00000301463	42	SPRYD3
ENSP00000398789	42	SNX13
ENSP00000415998	42	SLC48A1
ENSP00000258538	42	SLC41A2
ENSP00000352456	42	SLC33A1
ENSP00000360773	42	SLC29A1
ENSP00000344322	42	SLC23A2
ENSP00000194130	42	SLC13A1
ENSP00000343445	42	SERPINB4
ENSP00000263686	42	SELP
ENSP00000321845	42	SEC24C
ENSP00000345599	42	SCXA
ENSP00000206262	42	RGS17
ENSP00000371434	42	RFX3

ENSP00000316589	42	RANBP10
ENSP00000356607	42	RALGPS2
ENSP00000295718	42	PTPRN
ENSP00000241436	42	POLK
ENSP00000268124	42	POLG
ENSP00000265421	42	POLB
ENSP00000294964	42	PKDCC
ENSP00000317721	42	PIPOX
ENSP00000347046	42	PDE5A
ENSP00000367372	42	PCDHA2
ENSP00000007414	42	OSBPL7
ENSP00000316649	42	OSBPL2
ENSP00000262551	42	OGN
ENSP00000317992	42	NOC2L
ENSP00000180173	42	MTMR7
ENSP00000219162	42	MT4
ENSP00000263187	42	MSH4
ENSP00000305682	42	MRPL39
ENSP00000237380	42	MED28
ENSP00000329199	42	MAGEA6
ENSP00000352601	42	LRP10
ENSP00000384979	42	LINGO3
ENSP00000330720	42	KRTAP11-1
ENSP00000352064	42	KLRC1
ENSP00000262888	42	KCNN4
ENSP00000274629	42	KCNMB1
ENSP00000326981	42	IMP3
ENSP00000328111	42	IL25
ENSP00000261796	42	IL17B
ENSP00000374855	42	IGLC1
ENSP00000375015	42	IGHV3-20
ENSP00000419223	42	IGHJ6
ENSP00000418639	42	IGHD7-27
ENSP00000420442	42	IGHD3-3
ENSP00000296266	42	IFT122
ENSP00000360252	42	HOOK1
ENSP00000313699	42	HEPHL1
ENSP00000259895	42	GTF2H4
ENSP00000377969	42	GTF2F1
ENSP00000276708	42	GSDMC
ENSP00000369198	42	GPR64
ENSP00000295454	42	GABRB1
ENSP00000230124	42	FIG4
ENSP00000306099	42	FGB
ENSP00000260257	42	FDXACB1
ENSP00000237281	42	FBXO30

ENSP00000355890	42	EPRS
ENSP00000352540	42	EMP2
ENSP00000356920	42	DUSP12
ENSP00000320951	42	DEFB103A
ENSP00000219481	42	DECR2
ENSP00000412800	42	DDAH2
ENSP00000222122	42	DBP
ENSP00000222982	42	CYP3A5
ENSP00000355279	42	CNOT7
ENSP00000264734	42	CLDN16
ENSP00000299198	42	CKB
ENSP00000251363	42	CERS4
ENSP00000216264	42	CERK
ENSP00000262608	42	CECR2
ENSP00000250535	42	CDO1
ENSP00000229265	42	CDCA3
ENSP00000368124	42	CAMK1D
ENSP00000329926	42	CALHM1
ENSP00000313967	42	C1QB
ENSP00000320509	42	BANK1
ENSP00000261499	42	B9D1
ENSP00000299575	42	ATMIN
ENSP00000216124	42	ARSA
ENSP00000246174	42	ARMCX5
ENSP00000404304	42	ARMCX4
ENSP00000351221	42	ARMC8
ENSP00000366487	42	ARL5B
ENSP00000348828	42	ARHGEF26
ENSP00000391417	42	ANO6
ENSP00000215479	42	AMELY
ENSP00000357880	42	AMD1
ENSP00000313809	42	AMBN
ENSP00000380793	42	ALG3
ENSP00000362888	42	ADO
ENSP00000373472	42	ADAMTS7
ENSP00000249887	42	ACKR4
ENSP00000367079	42	ACACB
ENSP00000281527	42	-
ENSP00000307040	42	-
ENSP00000346984	42	-
ENSP00000375569	42	-
ENSP00000384994	42	-
ENSP00000415847	42	-
ENSP00000345809	41	ZNF197
ENSP00000155093	41	ZFY
ENSP00000324463	41	YLPM1

ENSP00000339122	41	VIPAS39
ENSP00000251412	41	TUBG2
ENSP00000369784	41	TUBAL3
ENSP00000410943	41	TSPYL4
ENSP00000231524	41	TRIM23
ENSP00000239462	41	TNN
ENSP00000233638	41	TLX2
ENSP00000218032	41	TLR8
ENSP00000271064	41	TINAGL1
ENSP00000264037	41	TECTA
ENSP00000317334	41	TCP1
ENSP00000263552	41	TBXAS1
ENSP00000362399	41	STXBP1
ENSP00000229812	41	STK38
ENSP00000360922	41	STAU1
ENSP00000269033	41	SSH2
ENSP00000340982	41	SPAG8
ENSP00000385746	41	SMS
ENSP00000354957	41	SMC5
ENSP00000393557	41	SLC4A4
ENSP00000362469	41	SLC2A8
ENSP00000370808	41	SLC25A6
ENSP00000216540	41	SLC10A1
ENSP00000264852	41	SIDT1
ENSP00000317817	41	SH2D3C
ENSP00000361382	41	SFTPA1
ENSP00000335321	41	SF3B1
ENSP00000316329	41	SCD5
ENSP00000349955	41	RPRD1A
ENSP00000346402	41	RNH1
ENSP00000262482	41	RNF167
ENSP00000334153	41	RFTN1
ENSP00000220062	41	RASL12
ENSP00000264158	41	RAB3GAP1
ENSP00000215071	41	PSMD8
ENSP00000326598	41	PRSS50
ENSP00000364697	41	PROZ
ENSP00000264233	41	POLQ
ENSP00000309542	41	PGA5
ENSP00000255266	41	PDE6A
ENSP00000361949	41	PABPC4
ENSP00000246081	41	OTOR
ENSP00000352671	41	OSCAR
ENSP00000360214	41	OPALIN
ENSP00000220514	41	OIP5
ENSP00000257570	41	OASL

ENSP00000228928	41	OAS3
ENSP00000265459	41	NRXN2
ENSP00000346890	41	NRD1
ENSP00000331487	41	NPLOC4
ENSP00000275820	41	NOM1
ENSP00000354541	41	NLGN1
ENSP00000345892	41	NDE1
ENSP00000262113	41	MYOM2
ENSP00000348821	41	MYOM1
ENSP00000265944	41	MYO3A
ENSP00000308330	41	MYEOV
ENSP00000255416	41	MYBPH
ENSP00000362718	41	MTCH1
ENSP00000314441	41	METTL1
ENSP00000323264	41	MARVELD2
ENSP00000362244	41	MAP7D1
ENSP00000355396	41	LRIG2
ENSP00000226522	41	LAMTOR3
ENSP00000342215	41	KIR2DL3
ENSP00000388241	41	KIF26A
ENSP00000290310	41	KCNE2
ENSP00000262487	41	ISM1
ENSP00000369042	41	IPO7
ENSP00000329991	41	IFNL1
ENSP00000337949	41	HTR7
ENSP00000359583	41	HPSE2
ENSP00000053469	41	GUCA1A
ENSP00000340823	41	GTF2F2
ENSP00000350757	41	GMFB
ENSP00000313423	41	GFRA4
ENSP00000354347	41	GFPT1
ENSP00000335592	41	GABRA5
ENSP00000312021	41	FUT1
ENSP00000298223	41	FOLR2
ENSP00000264658	41	FBXL20
ENSP00000264042	41	FARP2
ENSP00000229769	41	FANCE
ENSP00000419088	41	FAM162A
ENSP00000354461	41	FAM109A
ENSP00000261735	41	ERP29
ENSP00000307860	41	DIRC1
ENSP00000265036	41	DEPDC1B
ENSP00000373715	41	DCP2
ENSP00000222382	41	CYP3A43
ENSP00000346805	41	CRYBA4
ENSP00000317257	41	CPNE1

ENSP00000348385	41	COL27A1
ENSP00000337731	41	CIDEB
ENSP00000345133	41	CDC42BPG
ENSP00000348564	41	CDAN1
ENSP00000316333	41	CD55
ENSP00000257267	41	C5AR2
ENSP00000341610	41	C15orf48
ENSP00000351155	41	ATL1
ENSP00000361917	41	ARMCX1
ENSP00000327309	41	ARFGAP2
ENSP00000306010	41	ARF4
ENSP00000350425	41	APOA4
ENSP00000379086	41	ADM2
ENSP00000263238	41	ACTR3
ENSP00000311224	41	ACOT1
ENSP00000261200	41	ABCC9
ENSP00000276681	41	-
ENSP00000310831	41	-
ENSP00000322629	41	-
ENSP00000398312	41	-
ENSP00000399878	41	-
ENSP00000401223	41	-
ENSP00000412071	41	-
ENSP00000416304	41	-
ENSP00000289528	40	ZFAND2B
ENSP00000364448	40	UPF3A
ENSP00000293218	40	UNK
ENSP00000318697	40	TUBB6
ENSP00000362546	40	TTI1
ENSP00000243344	40	TTC21B
ENSP00000418089	40	TRGJP
ENSP00000318944	40	TRAF7
ENSP00000355566	40	TOMM20
ENSP00000336712	40	TNPO1
ENSP00000231198	40	THG1L
ENSP00000346691	40	TBC1D3
ENSP00000258301	40	STX6
ENSP00000366549	40	ST3GAL5
ENSP00000325905	40	SRSF7
ENSP00000334538	40	SREK1
ENSP00000345849	40	SPAM1
ENSP00000206020	40	SPAG7
ENSP00000366288	40	SLITRK1
ENSP00000300456	40	SLC27A4
ENSP00000264930	40	SLC12A7
ENSP00000373654	40	SKOR1

ENSP00000292055	40	SIK3
ENSP00000309714	40	SH3PXD2B
ENSP00000373614	40	SELPLG
ENSP00000251900	40	SCML2
ENSP00000256190	40	SBF2
ENSP00000371973	40	SAP18
ENSP00000243326	40	RIF1
ENSP00000325941	40	RIC8A
ENSP00000356430	40	RGS18
ENSP00000294435	40	RBP7
ENSP00000253363	40	RBM39
ENSP00000352956	40	RBM23
ENSP00000392147	40	PPP6C
ENSP00000308318	40	PPP1R3B
ENSP00000322304	40	PORCN
ENSP00000301015	40	PIEZO1
ENSP00000164640	40	PDZD4
ENSP00000263563	40	PALD1
ENSP00000357480	40	NUS1
ENSP00000287437	40	NSMCE2
ENSP00000354652	40	NPY1R
ENSP00000327545	40	NPTXR
ENSP00000368856	40	NFX1
ENSP00000306726	40	NETO2
ENSP00000350364	40	NEGR1
ENSP00000261435	40	N4BP2
ENSP00000349298	40	MYLIP
ENSP00000307513	40	MRC2
ENSP00000337907	40	MPP7
ENSP00000260229	40	MMP27
ENSP00000269202	40	MEP1B
ENSP00000260061	40	LRRC32
ENSP00000337224	40	LRAT
ENSP00000275635	40	LAT2
ENSP00000264501	40	KIAA1109
ENSP00000305824	40	KCNS3
ENSP00000252321	40	KCNA5
ENSP00000314520	40	KCNA2
ENSP00000330408	40	IST1
ENSP00000398208	40	INTS9
ENSP00000327889	40	INTS5
ENSP00000262644	40	IMPAD1
ENSP00000278353	40	HSD17B12
ENSP00000372746	40	HLA-DRA
ENSP00000380514	40	HAGH
ENSP00000341247	40	GSPT2

ENSP00000293190	40	GRIN2C
ENSP00000233838	40	GGCX
ENSP00000242209	40	FKBP9
ENSP00000257209	40	FHOD3
ENSP00000372326	40	FECH
ENSP00000270879	40	FCN3
ENSP00000381177	40	FAM153C
ENSP00000364324	40	FAM120C
ENSP00000261755	40	FAH
ENSP00000251527	40	ESYT2
ENSP00000261868	40	EIF3J
ENSP00000231887	40	EHHADH
ENSP00000296483	40	DUSP7
ENSP00000230431	40	DNPH1
ENSP00000365991	40	DNAJC3
ENSP00000358576	40	DCLRE1B
ENSP00000331470	40	DBX2
ENSP00000360991	40	CYP4B1
ENSP00000359540	40	CREG1
ENSP00000265641	40	CPT1A
ENSP00000317110	40	CORT
ENSP00000304327	40	COPRS
ENSP00000264903	40	CNOT6L
ENSP00000263856	40	CHMP3
ENSP00000364212	40	CDA
ENSP00000312027	40	CD7
ENSP00000257857	40	CD63
ENSP00000256652	40	CD101
ENSP00000357244	40	CCT3
ENSP00000206423	40	CCDC80
ENSP00000349562	40	CCDC180
ENSP00000252939	40	CALY
ENSP00000258947	40	CALCOCO2
ENSP00000274353	40	BHMT
ENSP00000319062	40	BBS12
ENSP00000305595	40	B3GNT2
ENSP00000419371	40	AZI2
ENSP00000329757	40	ATP6V0C
ENSP00000358777	40	ATP6AP1
ENSP00000302397	40	ATP1A3
ENSP00000354490	40	ATP1A2
ENSP00000363108	40	ASTN2
ENSP00000306788	40	ARL6IP1
ENSP00000257254	40	APLNR
ENSP00000413234	40	AP2A2
ENSP00000388996	40	AP1M1

ENSP00000386069	40	ADRA2C
ENSP00000379865	40	ADHFE1
ENSP00000264436	40	ADD2
ENSP00000254235	40	ADCY7
ENSP00000294517	40	ADC
ENSP00000286657	40	ADAMTS3
ENSP00000248151	40	-
ENSP00000280354	40	-
ENSP00000362398	40	-
ENSP00000375099	40	-
ENSP00000325326	39	ZNF335
ENSP00000312222	39	ZHX3
ENSP00000396292	39	ZBTB22
ENSP00000335038	39	VSTM2B
ENSP00000406988	39	VPS52
ENSP00000379931	39	UBE2E2
ENSP00000283645	39	TUBGCP5
ENSP00000173898	39	TRO
ENSP00000300747	39	TRIM68
ENSP00000365233	39	TPP2
ENSP00000216034	39	TOMM22
ENSP00000240185	39	TARDBP
ENSP00000321988	39	SULT1A1
ENSP00000229390	39	SRSF9
ENSP00000265729	39	SRI
ENSP00000345405	39	SPRED3
ENSP00000412566	39	SNRPB
ENSP00000370669	39	SLC8A3
ENSP00000298681	39	SLC39A2
ENSP00000247138	39	SLC35A2
ENSP00000361681	39	SLC25A53
ENSP00000319991	39	SLC16A6
ENSP00000318868	39	SHMT1
ENSP00000313025	39	SH3TC2
ENSP00000344148	39	SEMA6C
ENSP00000244926	39	SCGB1D2
ENSP00000303070	39	SCGB1D1
ENSP00000287482	39	SASS6
ENSP00000259235	39	SAP130
ENSP00000260385	39	RMDN3
ENSP00000391723	39	PUM1
ENSP00000348442	39	PSMD12
ENSP00000265636	39	PPP6R3
ENSP00000412297	39	PPP1R11
ENSP00000334840	39	PNRC2
ENSP00000364257	39	PLA2G2E

ENSP00000255882	39	PI4KA
ENSP00000260113	39	PI15
ENSP00000269848	39	PFKL
ENSP00000291565	39	PDXK
ENSP00000358033	39	PDSS2
ENSP00000331719	39	PCYT2
ENSP00000367366	39	PCDHA5
ENSP00000367096	39	PCDH9
ENSP00000363414	39	PAQR7
ENSP00000296783	39	PAPD4
ENSP00000281701	39	NVL
ENSP00000229179	39	NUP107
ENSP00000339377	39	NPY5R
ENSP00000292199	39	NLRX1
ENSP00000360492	39	NDUFA1
ENSP00000261745	39	NAA25
ENSP00000382177	39	MYO5A
ENSP00000313921	39	MSRA
ENSP00000355388	39	MPHOSPH8
ENSP00000353767	39	MMP17
ENSP00000267812	39	MFAP1
ENSP00000358719	39	ME1
ENSP00000328118	39	MCF2L2
ENSP00000364721	39	MAPRE1
ENSP00000340467	39	LRRC16B
ENSP00000359925	39	LRRC1
ENSP00000253193	39	LRP3
ENSP00000344353	39	LPAR6
ENSP00000251047	39	LMAN1
ENSP00000353346	39	LHFPL5
ENSP00000229319	39	LDHB
ENSP00000357769	39	LCE2B
ENSP00000217407	39	LBP
ENSP00000419901	39	L1TD1
ENSP00000375237	39	KRTAP2-3
ENSP00000402240	39	KIAA1432
ENSP00000306497	39	KCNJ4
ENSP00000288098	39	IL34
ENSP00000417344	39	IGKV1-16
ENSP00000247584	39	HIVEP3
ENSP00000216338	39	GZMH
ENSP00000355155	39	GRIN3A
ENSP00000383382	39	GP1BB
ENSP00000315263	39	GLTP
ENSP00000262460	39	GINS1
ENSP00000255945	39	GIMAP4

ENSP00000302833	39	GIMAP1
ENSP00000257248	39	GIF
ENSP00000311962	39	GGA2
ENSP00000253778	39	GFPT2
ENSP00000274545	39	GABRA6
ENSP00000318142	39	FUT7
ENSP00000368207	39	FTHL17
ENSP00000344866	39	FBXL5
ENSP00000350673	39	FAM3B
ENSP00000308541	39	F2
ENSP00000369371	39	EQTN
ENSP00000334314	39	EML1
ENSP00000273783	39	EIF2B5
ENSP00000381167	39	DYNC2H1
ENSP00000310565	39	DSEL
ENSP00000311399	39	DSCR3
ENSP00000346142	39	DPAGT1
ENSP00000222219	39	DNASE2
ENSP00000260818	39	DNAJC13
ENSP00000280346	39	DLAT
ENSP00000318867	39	CYP8B1
ENSP00000358695	39	CYB5R4
ENSP00000220166	39	CTSH
ENSP00000307741	39	CMTM8
ENSP00000381120	39	CMIP
ENSP00000185206	39	CLIC5
ENSP00000302569	39	CLEC7A
ENSP00000359596	39	CLCA2
ENSP00000216756	39	CINP
ENSP00000223500	39	CHMP5
ENSP00000358763	39	CHI3L2
ENSP00000378130	39	CFI
ENSP00000268383	39	CDR2
ENSP00000363322	39	CDC26
ENSP00000299300	39	CCT2
ENSP00000314099	39	CA5B
ENSP00000330753	39	BRWD1
ENSP00000354568	39	BRDT
ENSP00000255192	39	BHMT2
ENSP00000354675	39	BHLHB9
ENSP00000349887	39	ASNA1
ENSP00000276211	39	ARHGAP36
ENSP00000312326	39	AOC3
ENSP00000370088	39	AMELX
ENSP00000200557	39	ADAM11
ENSP00000254286	39	ACTR10

ENSP00000315309	39	-
ENSP00000377509	39	-
ENSP00000392703	39	-
ENSP00000404772	39	-
ENSP00000414032	39	-
ENSP00000200135	38	ZW10
ENSP00000228289	38	ZNF268
ENSP00000370150	38	WRNIP1
ENSP00000376097	38	URI1
ENSP00000239878	38	UFM1
ENSP00000366249	38	UBD
ENSP00000348565	38	UBA5
ENSP00000221399	38	TULP2
ENSP00000303452	38	TRH
ENSP00000379933	38	TPI1
ENSP00000320757	38	TMEM150B
ENSP00000304689	38	THAP11
ENSP00000306574	38	TEFM
ENSP00000386921	38	TBC1D14
ENSP00000387593	38	TANC2
ENSP00000258281	38	TAF5L
ENSP00000363727	38	STARD8
ENSP00000225276	38	ST6GALNAC2
ENSP00000227495	38	ST3GAL4
ENSP00000298396	38	SSX3
ENSP00000374274	38	SSC5D
ENSP00000244020	38	SRSF6
ENSP00000320885	38	SPAST
ENSP00000368831	38	SNX2
ENSP00000323439	38	SMC6
ENSP00000266682	38	SLC6A15
ENSP00000379326	38	SLC5A12
ENSP00000381089	38	SLC35E3
ENSP00000281154	38	SLC25A31
ENSP00000215882	38	SLC25A1
ENSP00000298877	38	SLC24A4
ENSP00000312550	38	SEZ6L2
ENSP00000348918	38	SAA1
ENSP00000410862	38	RUNDC3A
ENSP00000346644	38	RPS6KL1
ENSP00000262406	38	RGS9
ENSP00000408295	38	RFC5
ENSP00000306817	38	RBKS
ENSP00000379051	38	PURB
ENSP00000290541	38	PSMB4
ENSP00000241808	38	PRM2

ENSP00000365747	38	PQBP1
ENSP00000265465	38	POLA2
ENSP00000247992	38	PLA2G2C
ENSP00000363124	38	PHF20
ENSP00000322170	38	PFN4
ENSP00000370517	38	PFKP
ENSP00000334642	38	PDZD8
ENSP00000259467	38	PDCL
ENSP00000402918	38	OXR1
ENSP00000296220	38	OSBPL11
ENSP00000337196	38	OGFOD1
ENSP00000361843	38	NXF2
ENSP00000368459	38	NUFIP1
ENSP00000359297	38	NSDHL
ENSP00000383142	38	NFKBIL1
ENSP00000366326	38	NEBL
ENSP00000272425	38	NAT8
ENSP00000407952	38	NAMPTL
ENSP00000274054	38	NAF1
ENSP00000378288	38	MYLK3
ENSP00000377617	38	MTHFD2
ENSP00000278937	38	MPZL2
ENSP00000301159	38	LIN37
ENSP00000314311	38	LARP7
ENSP00000264690	38	KLKB1
ENSP00000326159	38	KLK4
ENSP00000383645	38	KIR2DL2
ENSP00000359219	38	KAZALD1
ENSP00000375042	38	IGHV1-69
ENSP00000270112	38	HUNK
ENSP00000216044	38	GTPBP1
ENSP00000369018	38	GPR56
ENSP00000251337	38	GNAT2
ENSP00000371236	38	GART
ENSP00000302707	38	FPR1
ENSP00000223528	38	FKTN
ENSP00000274963	38	FGD2
ENSP00000265651	38	FBXO3
ENSP00000346693	38	ELOVL2
ENSP00000199389	38	EIF2AK1
ENSP00000302051	38	ECEL1
ENSP00000372160	38	DOK6
ENSP00000332258	38	DGAT1
ENSP00000362401	38	CYSLTR1
ENSP00000332679	38	CYP2A13
ENSP00000226317	38	CXCL6

ENSP00000312099	38	CRYGS
ENSP00000296658	38	CMBL
ENSP00000296130	38	CLEC3B
ENSP00000264249	38	CHST10
ENSP00000279249	38	CDC42EP2
ENSP00000280326	38	CCT5
ENSP00000316224	38	CARF
ENSP00000009105	38	CAMK1G
ENSP00000248420	38	CACTIN
ENSP00000334267	38	C5orf38
ENSP00000302079	38	C3AR1
ENSP00000373657	38	C17orf85
ENSP00000341762	38	BSPH1
ENSP00000396976	38	BAHD1
ENSP00000306822	38	AVEN
ENSP00000297512	38	ASIC3
ENSP00000320675	38	ASB2
ENSP00000264607	38	ASB1
ENSP00000320038	38	ARHGAP33
ENSP00000302895	38	ARAP2
ENSP00000309749	38	APOBEC3F
ENSP00000335255	38	ANGPTL5
ENSP00000387739	38	AMOTL1
ENSP00000265132	38	AMBP
ENSP00000223029	38	AIMP2
ENSP00000286355	38	ADCY8
ENSP00000261880	38	AAGAB
ENSP00000221167	38	-
ENSP00000345280	38	-
ENSP00000347165	38	-
ENSP00000374581	38	-
ENSP00000388516	38	-
ENSP00000394981	38	-
ENSP00000301096	37	ZNF83
ENSP00000334853	37	ZNF555
ENSP00000332861	37	ZNF530
ENSP00000338572	37	ZNF212
ENSP00000240731	37	ZNF211
ENSP00000261381	37	XYLT1
ENSP00000302790	37	XPO6
ENSP00000346667	37	WNK3
ENSP00000235521	37	WARS2
ENSP00000263864	37	VAMP8
ENSP00000261776	37	VAC14
ENSP00000262803	37	UPF1
ENSP00000346768	37	UGT1A9

ENSP00000229771	37	TULP1
ENSP00000325738	37	TNP2
ENSP00000252898	37	TNNI2
ENSP00000363714	37	TMEM245
ENSP00000313408	37	TMC6
ENSP00000310796	37	THAP2
ENSP00000341803	37	TCTEX1D4
ENSP00000371932	37	TAS2R1
ENSP00000303522	37	TACR1
ENSP00000246032	37	STK35
ENSP00000265896	37	SQLE
ENSP00000251809	37	SPAG1
ENSP00000375899	37	SP140
ENSP00000295951	37	SLMAP
ENSP00000379008	37	SLC5A10
ENSP00000371483	37	SLC34A2
ENSP00000347778	37	SLC26A8
ENSP00000200652	37	SLC22A4
ENSP00000363229	37	SLC18A3
ENSP00000318557	37	SLC12A4
ENSP00000269724	37	SAMD1
ENSP00000362578	37	RNF8
ENSP00000222145	37	RASIP1
ENSP00000242249	37	RAMP3
ENSP00000373022	37	PSORS1C2
ENSP00000357879	37	PSMD4
ENSP00000396937	37	PSMD13
ENSP00000406797	37	PSMB8
ENSP00000304350	37	PRPF8
ENSP00000239032	37	PRLHR
ENSP00000215912	37	PIK3IP1
ENSP00000351379	37	PFN3
ENSP00000342143	37	PDZK1
ENSP00000387536	37	PDK3
ENSP00000278060	37	PAOX
ENSP00000365320	37	PAGE1
ENSP00000303686	37	NTSR2
ENSP00000416320	37	NTM
ENSP00000373674	37	NT5M
ENSP00000329568	37	NLRP7
ENSP00000296499	37	NDST3
ENSP00000362181	37	NCR2
ENSP00000348657	37	NCAPG2
ENSP00000312235	37	MUC13
ENSP00000257068	37	MTNR1B
ENSP00000354687	37	MT-ND1

ENSP00000296473	37	MON1A
ENSP00000380888	37	MED13
ENSP00000361287	37	MAT1A
ENSP00000359301	37	MAGEA3
ENSP00000209718	37	KRT23
ENSP00000316136	37	KCNJ1
ENSP00000385478	37	ISPD
ENSP00000414237	37	INTS2
ENSP00000374981	37	IGHA2
ENSP00000295256	37	HPGDS
ENSP00000383516	37	HMX1
ENSP00000372582	37	HLA-DPB1
ENSP00000334002	37	HAP1
ENSP00000243673	37	GPR83
ENSP00000253462	37	GINS2
ENSP00000222803	37	FKBP14
ENSP00000383746	37	FCAMR
ENSP00000267950	37	ETFA
ENSP00000332151	37	DSE
ENSP00000259512	37	DERL1
ENSP00000293371	37	DCD
ENSP00000355050	37	CTNNBL1
ENSP00000215855	37	CRYBB3
ENSP00000240316	37	COIL
ENSP00000341882	37	CNTN6
ENSP00000359225	37	CNN3
ENSP00000226951	37	CLNK
ENSP00000287916	37	CLDN12
ENSP00000352581	37	CLASP2
ENSP00000220913	37	CHRAC1
ENSP00000368080	37	CDKL4
ENSP00000313875	37	CD46
ENSP00000376250	37	CCDC50
ENSP00000335615	37	BTBD7
ENSP00000252593	37	BST2
ENSP00000369996	37	ATXN3L
ENSP00000390941	37	ATCAY
ENSP00000260746	37	ARL3
ENSP00000341071	37	ARHGEF3
ENSP00000355060	37	ARHGEF10L
ENSP00000219919	37	AQP9
ENSP00000263126	37	AKR1C4
ENSP00000365227	37	AIF1
ENSP00000367220	37	ACTR2
ENSP00000293217	37	ACOX1
ENSP00000377496	37	ACD

ENSP00000218104	37	ABCD1
ENSP00000299322	37	-
ENSP00000312758	37	-
ENSP00000351541	37	-
ENSP00000351716	37	-
ENSP00000354569	37	-
ENSP00000372489	37	-
ENSP00000413394	37	-
ENSP00000046087	36	ZPBP
ENSP00000334170	36	ZNF410
ENSP00000318480	36	YME1L1
ENSP00000367299	36	VPS36
ENSP00000396918	36	URGCP
ENSP00000380178	36	UBE2G1
ENSP00000346791	36	TTC3
ENSP00000333441	36	TREX2
ENSP00000381950	36	TMEM216
ENSP00000186436	36	TMEM131
ENSP00000351732	36	TMC2
ENSP00000354911	36	TIFA
ENSP00000374584	36	TEX14
ENSP00000332565	36	SULT4A1
ENSP00000343645	36	SULT1A3
ENSP00000346599	36	STYX
ENSP00000275560	36	SRCRB4D
ENSP00000289431	36	SPATA2
ENSP00000230671	36	SLC6A7
ENSP00000346298	36	SLC6A20
ENSP00000269187	36	SLC39A6
ENSP00000357650	36	SLC27A3
ENSP00000270792	36	SH3BGRL3
ENSP00000340608	36	SGK2
ENSP00000218867	36	SGCG
ENSP00000331692	36	SCRT1
ENSP00000283256	36	SCN2A
ENSP00000373713	36	SACM1L
ENSP00000255499	36	RNF128
ENSP00000297313	36	RGS20
ENSP00000253303	36	RGN
ENSP00000355969	36	RD3
ENSP00000266560	36	RBP5
ENSP00000334813	36	RAX
ENSP00000336616	36	RASSF3
ENSP00000323074	36	RAI1
ENSP00000387477	36	PRRC2A
ENSP00000227524	36	PRPF19

ENSP00000405950	36	PNCK
ENSP00000370377	36	PITRM1
ENSP00000382595	36	PAICS
ENSP00000320376	36	P2RY13
ENSP00000358747	36	OVGP1
ENSP00000289547	36	NPC1L1
ENSP00000352021	36	NOL12
ENSP00000293373	36	NCKAP1L
ENSP00000310998	36	NAGPA
ENSP00000333657	36	MX2
ENSP00000228510	36	MVK
ENSP00000354813	36	MT-ND5
ENSP00000216605	36	MTHFD1
ENSP00000356552	36	MR1
ENSP00000370410	36	MPDZ
ENSP00000248248	36	MON1B
ENSP00000260228	36	MMP20
ENSP00000217246	36	MACROD2
ENSP00000302297	36	LGI3
ENSP00000215886	36	LGALS2
ENSP00000408411	36	LAMP2
ENSP00000365469	36	KLHL35
ENSP00000250916	36	KLF16
ENSP00000322791	36	KIF1A
ENSP00000310338	36	KIAA1551
ENSP00000367828	36	ITM2B
ENSP00000395219	36	IGFL2
ENSP00000287907	36	HTR5A
ENSP00000216106	36	HMGXB4
ENSP00000364114	36	HLA-DRB5
ENSP00000364493	36	HIATL1
ENSP00000342118	36	HHIPL2
ENSP00000375066	36	HCAR2
ENSP00000282058	36	HAUS1
ENSP00000259254	36	GYPC
ENSP00000228955	36	GTF2H3
ENSP00000280187	36	GPM6A
ENSP00000365807	36	GNA14
ENSP00000379895	36	GATM
ENSP00000331912	36	GABRG3
ENSP00000397530	36	FKBPL
ENSP00000278919	36	FEZ1
ENSP00000295133	36	FBXO41
ENSP00000297532	36	FASTK
ENSP00000360437	36	FAM210B
ENSP00000359939	36	EXOSC1

ENSP00000237853	36	ELL2
ENSP00000270517	36	ECSIT
ENSP00000360644	36	DPM1
ENSP00000205402	36	DLD
ENSP00000384573	36	DAZ1
ENSP00000260682	36	CYP2C9
ENSP00000360372	36	CYP2C19
ENSP00000403721	36	CYP21A2
ENSP00000390475	36	CXXC1
ENSP00000247306	36	CTAG2
ENSP00000370021	36	CNTLN
ENSP00000360939	36	CMPK1
ENSP00000327009	36	CLIP4
ENSP00000183605	36	CLDN18
ENSP00000339867	36	CLCN1
ENSP00000258969	36	CHAD
ENSP00000262150	36	CDH19
ENSP00000323280	36	CD6
ENSP00000352071	36	CD163
ENSP00000350256	36	CCR9
ENSP00000405533	36	CCL4L1
ENSP00000304643	36	CALML6
ENSP00000347197	36	C5AR1
ENSP00000372853	36	C2
ENSP00000338769	36	ASAP3
ENSP00000352995	36	ARHGEF18
ENSP00000416094	36	APOBR
ENSP00000215941	36	ANKRD54
ENSP00000296785	36	ANKRA2
ENSP00000226253	36	ALDOC
ENSP00000419361	36	ADCY5
ENSP00000230914	36	-
ENSP00000346913	36	-
ENSP00000349289	36	-
ENSP00000354684	36	-
ENSP00000377404	36	-
ENSP00000386217	36	-
ENSP00000397905	36	-
ENSP00000408618	36	-
ENSP00000265069	35	ZFR
ENSP00000294258	35	ZFPL1
ENSP00000337313	35	ZCCHC8
ENSP00000378286	35	ZBTB3
ENSP00000366098	35	YIF1A
ENSP00000238497	35	VPS4B
ENSP00000362549	35	UGT1A8

ENSP00000261965	35	TUBGCP3
ENSP00000358529	35	TSPAN2
ENSP00000312675	35	TRIM72
ENSP00000369373	35	TRIM5
ENSP00000302413	35	TPST1
ENSP00000278317	35	TNNT3
ENSP00000367440	35	TDP2
ENSP00000257264	35	TCN1
ENSP00000411197	35	TBC1D9
ENSP00000384917	35	SVEP1
ENSP00000361032	35	SURF2
ENSP00000294179	35	STX5
ENSP00000360183	35	STX16
ENSP00000373539	35	STAB2
ENSP00000169298	35	ST6GAL1
ENSP00000360371	35	SSBP3
ENSP00000260956	35	SSB
ENSP00000403310	35	SNRNP35
ENSP00000351947	35	SMARCAD1
ENSP00000219343	35	SLC7A6
ENSP00000298085	35	SLC7A3
ENSP00000310208	35	SLC5A6
ENSP00000363852	35	SLC44A1
ENSP00000319574	35	SLC25A19
ENSP00000329033	35	SLC25A18
ENSP00000390722	35	SLC25A17
ENSP00000261187	35	SLC16A7
ENSP00000305675	35	SDPR
ENSP00000221431	35	SARS2
ENSP00000262878	35	SAMHD1
ENSP00000382895	35	RPGRIP1
ENSP00000223129	35	RPA3
ENSP00000317039	35	RMI1
ENSP00000382239	35	RBM12B
ENSP00000262305	35	RAB11FIP3
ENSP00000020673	35	PSD
ENSP00000337194	35	PRPF4B
ENSP00000263433	35	PPP1R12C
ENSP00000339529	35	POP1
ENSP00000293860	35	POLR3K
ENSP00000339613	35	POFUT2
ENSP00000366460	35	PLXDC2
ENSP00000323927	35	PLXDC1
ENSP00000380460	35	PLAA
ENSP00000339826	35	PIK3AP1
ENSP00000362942	35	PHACTR4

ENSP00000292357	35	PEAR1
ENSP00000218230	35	PCSK1N
ENSP00000194155	35	PCDHB2
ENSP00000393559	35	PACS2
ENSP00000361141	35	OPN4
ENSP00000294172	35	NXF1
ENSP00000376921	35	NTNG2
ENSP00000311364	35	MUCL1
ENSP00000324834	35	MUC3A
ENSP00000365473	35	MUC21
ENSP00000302811	35	MTNR1A
ENSP00000318318	35	MPI
ENSP00000203630	35	MLF2
ENSP00000349490	35	MFNG
ENSP00000392666	35	MEGF9
ENSP00000368174	35	MCM8
ENSP00000346581	35	MAP7
ENSP00000261921	35	LOXL1
ENSP00000334052	35	LGMN
ENSP00000360753	35	LCN8
ENSP00000203629	35	LAG3
ENSP00000261893	35	LACTB
ENSP00000355517	35	KMO
ENSP00000382770	35	KCNU1
ENSP00000334650	35	KCNK18
ENSP00000357068	35	KCNJ10
ENSP00000249760	35	IVD
ENSP00000343000	35	IL19
ENSP00000254801	35	IGJ
ENSP00000362121	35	IFT52
ENSP00000359581	35	HS2ST1
ENSP00000359393	35	HMGB3
ENSP00000222115	35	HAS1
ENSP00000277903	35	HABP2
ENSP00000283875	35	GTF2E1
ENSP00000384582	35	GPR98
ENSP00000279168	35	GPHA2
ENSP00000318690	35	GCFC2
ENSP00000359000	35	GBF1
ENSP00000317232	35	FKBP10
ENSP00000322323	35	FAM20C
ENSP00000218099	35	F9
ENSP00000250263	35	ERI1
ENSP00000265038	35	ERCC8
ENSP00000348842	35	EML6
ENSP00000334037	35	EMC10

ENSP00000350336	35	DSCR8
ENSP00000315465	35	DSCAML1
ENSP00000359290	35	DR1
ENSP00000242317	35	DNAI1
ENSP00000316476	35	DEGS1
ENSP00000366031	35	CST7
ENSP00000323978	35	CRIPAK
ENSP00000229251	35	COPS7A
ENSP00000325506	35	CLVS1
ENSP00000388872	35	CLUH
ENSP00000319984	35	CHRM2
ENSP00000299847	35	CHRFAM7A
ENSP00000384400	35	CHKB
ENSP00000356579	35	CEP350
ENSP00000371308	35	CENPJ
ENSP00000365465	35	CDSN
ENSP00000420298	35	CD200
ENSP00000377958	35	CCT4
ENSP00000398391	35	CC2D2A
ENSP00000369689	35	CALML5
ENSP00000317310	35	CABP1
ENSP00000263408	35	C9
ENSP00000342032	35	BPGM
ENSP00000311766	35	ATAD3B
ENSP00000225823	35	ASIC2
ENSP00000378897	35	ASAH2
ENSP00000264028	35	ARCN1
ENSP00000263270	35	AP2S1
ENSP00000358878	35	AMIGO1
ENSP00000318313	35	AMBRA1
ENSP00000265512	35	ADH4
ENSP00000250974	35	ABHD17A
ENSP00000366084	35	ABCC4
ENSP00000205214	35	AASDH
ENSP00000221852	35	-
ENSP00000300793	35	-
ENSP00000329626	35	-
ENSP00000341966	35	-
ENSP00000395190	35	-
ENSP00000415837	35	-
ENSP00000309606	34	ZNF408
ENSP00000384434	34	ZGLP1
ENSP00000286428	34	VBP1
ENSP00000248846	34	TUBGCP6
ENSP00000259818	34	TUBB2B
ENSP00000380897	34	TRPM1

ENSP00000332284	34	TRIM69
ENSP00000299413	34	TRIM44
ENSP00000411949	34	TRIM39
ENSP00000309818	34	TRHR
ENSP00000010338	34	TRAF3IP3
ENSP00000279263	34	TM7SF2
ENSP00000358854	34	TAF5
ENSP00000226444	34	SULT1E1
ENSP00000338742	34	SULT1A2
ENSP00000339435	34	SRR
ENSP00000341324	34	SRFBP1
ENSP00000340799	34	SP6
ENSP00000258381	34	SP110
ENSP00000355110	34	SMOC1
ENSP00000352702	34	SLC6A12
ENSP00000311402	34	SLC4A2
ENSP00000376794	34	SLC35G2
ENSP00000217420	34	SLC32A1
ENSP00000355968	34	SLC30A1
ENSP00000342267	34	SLC25A15
ENSP00000355920	34	SLC22A2
ENSP00000310241	34	SLC22A13
ENSP00000276033	34	SLC16A2
ENSP00000355979	34	SERTAD4
ENSP00000342109	34	SERPINA12
ENSP00000247020	34	SDF2
ENSP00000362650	34	SCAI
ENSP00000330188	34	RSRC2
ENSP00000198767	34	RRN3
ENSP00000309365	34	RNF146
ENSP00000355520	34	RGS7
ENSP00000273968	34	PYURF
ENSP00000044462	34	PSMA4
ENSP00000260045	34	PRKRIR
ENSP00000299853	34	POLR3E
ENSP00000274793	34	PLA2G7
ENSP00000262483	34	PITPNM3
ENSP00000358417	34	PHGDH
ENSP00000366876	34	PHF13
ENSP00000263174	34	PALMD
ENSP00000311196	34	NPAS4
ENSP00000278886	34	NINL
ENSP00000345292	34	NDRG3
ENSP00000255381	34	MYH4
ENSP00000260453	34	MNS1
ENSP00000240488	34	MND1

ENSP00000312834	34	MLXIP
ENSP00000246912	34	MLX
ENSP00000266003	34	MLN
ENSP00000296350	34	MFI2
ENSP00000255764	34	MED10
ENSP00000354575	34	LSR
ENSP00000260665	34	LRPPRC
ENSP00000264094	34	LOXL3
ENSP00000264162	34	LCT
ENSP00000375682	34	KLK8
ENSP00000337255	34	KCNE1
ENSP00000321184	34	IGSF3
ENSP00000375009	34	IGHV3-9
ENSP00000319623	34	IGDCC4
ENSP00000310749	34	HPCAL1
ENSP00000366005	34	HLA-A
ENSP00000312042	34	HDLBP
ENSP00000351430	34	GYPE
ENSP00000370532	34	GTF3A
ENSP00000236255	34	GRHL3
ENSP00000308908	34	GPR148
ENSP00000263863	34	GNLY
ENSP00000218075	34	GLRA2
ENSP00000223293	34	GIMAP2
ENSP00000369943	34	GCOM1
ENSP00000340396	34	GBP5
ENSP00000340466	34	GANAB
ENSP00000249041	34	GALR3
ENSP00000341821	34	FPR3
ENSP00000271234	34	FNBP1L
ENSP00000283268	34	FEZF2
ENSP00000361345	34	FAM78A
ENSP00000363753	34	FAM206A
ENSP00000264059	34	EFHD1
ENSP00000381921	34	DSCR4
ENSP00000256935	34	DOCK2
ENSP00000359699	34	DNASE2B
ENSP00000389455	34	DIRC3
ENSP00000354623	34	DFNB31
ENSP00000275016	34	CYP39A1
ENSP00000354028	34	CYB561
ENSP00000271835	34	CRNN
ENSP00000263045	34	CRISP3
ENSP00000312189	34	CPT1B
ENSP00000304102	34	COPS6
ENSP00000272602	34	CNGA3

ENSP00000315602	34	CHRNA3
ENSP00000335632	34	CHP1
ENSP00000271688	34	CERS2
ENSP00000281453	34	CENPU
ENSP00000381822	34	CDH23
ENSP00000308117	34	CD248
ENSP00000349967	34	CCRL2
ENSP00000315299	34	CALML3
ENSP00000301050	34	CACNB3
ENSP00000365441	34	CACNA1F
ENSP00000407200	34	C8orf59
ENSP00000331784	34	C7orf13
ENSP00000349358	34	C12orf29
ENSP00000350267	34	BRPF3
ENSP00000362993	34	BICC1
ENSP00000223368	34	BCL7B
ENSP00000264499	34	BBS7
ENSP00000353458	34	BAZ1A
ENSP00000243440	34	BATF3
ENSP00000352665	34	ATP2C1
ENSP00000185907	34	ASAH2B
ENSP00000371152	34	ASAH1
ENSP00000249044	34	APOL5
ENSP00000227665	34	APOA5
ENSP00000297183	34	ANKHD1
ENSP00000370129	34	AKR1C2
ENSP00000217426	34	AHCY
ENSP00000346478	34	ADAMTSL2
ENSP00000270328	34	ADAMTS10
ENSP00000348429	34	ACSL5
ENSP00000285093	34	ACAA2
ENSP00000268129	34	ABHD2
ENSP00000359478	34	ABCC2
ENSP00000379704	34	3-Sep
ENSP00000244316	34	-
ENSP00000257452	34	-
ENSP00000326473	34	-
ENSP00000344453	34	-
ENSP00000363875	34	-
ENSP00000385448	34	-
ENSP00000401040	34	-
ENSP00000402620	34	-
ENSP00000350869	33	ZNF346
ENSP00000333253	33	ZC3H11A
ENSP00000384792	33	WDR62
ENSP00000353422	33	VPS13A

ENSP00000283713	33	VILL
ENSP00000314556	33	UACA
ENSP00000362634	33	TSSK3
ENSP00000312304	33	TPMT
ENSP00000345719	33	TOR1A
ENSP00000379144	33	TNRC6A
ENSP00000359928	33	TNNI3K
ENSP00000319009	33	TMEM45A
ENSP00000300258	33	TCP10L
ENSP00000341489	33	SYDE1
ENSP00000319208	33	SUV39H2
ENSP00000350071	33	STRN3
ENSP00000319318	33	STAG3
ENSP00000243563	33	SNRPA
ENSP00000311291	33	SLCO2A1
ENSP00000282587	33	SLC30A6
ENSP00000360966	33	SLC2A6
ENSP00000236137	33	SLC19A2
ENSP00000341141	33	SIGLEC1
ENSP00000350018	33	SH3BP1
ENSP00000263753	33	SGOL1
ENSP00000374280	33	RTF1
ENSP00000369320	33	RS1
ENSP00000311028	33	RPS14
ENSP00000286070	33	RBM45
ENSP00000261741	33	RBM19
ENSP00000336866	33	RBBP9
ENSP00000327583	33	RANBP1
ENSP00000382492	33	RADIL
ENSP00000335610	33	QRFPR
ENSP00000216392	33	PYGL
ENSP00000377783	33	PROS1
ENSP00000286719	33	PPEF2
ENSP00000216180	33	PNPLA3
ENSP00000407401	33	PEX5
ENSP00000248633	33	PEX1
ENSP00000358812	33	PDCD11
ENSP00000367151	33	PCDH17
ENSP00000354950	33	PCDH15
ENSP00000251203	33	PBX4
ENSP00000242104	33	OCM
ENSP00000366934	33	NOL9
ENSP00000297990	33	NOL6
ENSP00000258042	33	NMBR
ENSP00000216286	33	NID2
ENSP00000396103	33	MYT1L

ENSP00000323047	33	MTG1
ENSP00000318077	33	MC5R
ENSP00000296280	33	MASP1
ENSP00000267978	33	MAN2C1
ENSP00000347358	33	MAGEA11
ENSP00000405165	33	LIMS3
ENSP00000343490	33	LCTL
ENSP00000364764	33	LAYN
ENSP00000256644	33	LAMTOR5
ENSP00000328236	33	KNTC1
ENSP00000257765	33	KHDC1
ENSP00000339960	33	KCNJ5
ENSP00000249842	33	ISLR
ENSP00000269159	33	IMPA2
ENSP00000222728	33	HOXA6
ENSP00000376309	33	HNRNPA3
ENSP00000379839	33	GNE
ENSP00000358867	33	GNAI3
ENSP00000264318	33	GABRA4
ENSP00000318177	33	FUBP3
ENSP00000209929	33	FMO2
ENSP00000346901	33	FMO1
ENSP00000354691	33	FCRL5
ENSP00000377280	33	FBXO8
ENSP00000338532	33	FAM53B
ENSP00000264691	33	F11
ENSP00000374354	33	EXOSC8
ENSP00000317564	33	EID2B
ENSP00000349679	33	DYNLRB1
ENSP00000356520	33	DHX9
ENSP00000284690	33	DHX32
ENSP00000395405	33	DDR1
ENSP00000333534	33	CYP2F1
ENSP00000353373	33	CUEDC1
ENSP00000217109	33	CSTF1
ENSP00000319343	33	CPT1C
ENSP00000272852	33	CPO
ENSP00000181383	33	CPB2
ENSP00000417656	33	COX7B
ENSP00000357048	33	COPA
ENSP00000296387	33	CLDN19
ENSP00000254190	33	CHSY1
ENSP00000307911	33	CHST2
ENSP00000261007	33	CHRNA1
ENSP00000317902	33	CEP57
ENSP00000299736	33	CENPV

ENSP00000290583	33	CELF3
ENSP00000359563	33	CDR1
ENSP00000329507	33	CD300C
ENSP00000344460	33	CBS
ENSP00000297273	33	CASD1
ENSP00000340329	33	CAPRIN1
ENSP00000361878	33	CAP1
ENSP00000411851	33	C17orf49
ENSP00000401498	33	C11orf94
ENSP00000350310	33	ATP2B4
ENSP00000404725	33	ATF6B
ENSP00000228468	33	ASIC1
ENSP00000417914	33	ASB11
ENSP00000370522	33	ARSH
ENSP00000369217	33	ARIH1
ENSP00000315371	33	ANO5
ENSP00000343021	33	ALKBH2
ENSP00000371230	33	AK3
ENSP00000358814	33	AHCYL1
ENSP00000265769	33	ADAM28
ENSP00000242475	33	-
ENSP00000253335	33	-
ENSP00000301647	33	-
ENSP00000303983	33	-
ENSP00000304507	33	-
ENSP00000390041	33	-
ENSP00000393335	33	-
ENSP00000420824	33	-
ENSP00000319053	32	ZNF77
ENSP00000315173	32	ZNF41
ENSP00000337368	32	ZNF224
ENSP00000278319	32	ZNF215
ENSP00000278314	32	ZNF214
ENSP00000359291	32	ZNF185
ENSP00000418210	32	ZMYND8
ENSP00000347872	32	VAT1
ENSP00000328939	32	UTY
ENSP00000390759	32	USP17L13
ENSP00000357775	32	UROS
ENSP00000291901	32	TNNT1
ENSP00000270538	32	TIMM44
ENSP00000300584	32	TBC1D2B
ENSP00000354633	32	TAF11
ENSP00000338562	32	STX3
ENSP00000368952	32	STOML3
ENSP00000312081	32	SSH3

ENSP00000262554	32	SPTLC1
ENSP00000296695	32	SPINK1
ENSP00000311684	32	SPEG
ENSP00000265007	32	SOX30
ENSP00000317332	32	SNX18
ENSP00000287766	32	SLC6A1
ENSP00000256689	32	SLC38A2
ENSP00000363394	32	SLC30A2
ENSP00000367102	32	SLC22A6
ENSP00000244527	32	SLC17A1
ENSP00000255226	32	SLC14A2
ENSP00000358541	32	SIKE1
ENSP00000310448	32	SART1
ENSP00000261441	32	RSBN1
ENSP00000315950	32	RNF32
ENSP00000313350	32	RNASEH1
ENSP00000234195	32	RMDN2
ENSP00000370750	32	RDH11
ENSP00000300069	32	RBPMS2
ENSP00000248706	32	RASL11B
ENSP00000417658	32	PTCHD3
ENSP00000292644	32	PSMC2
ENSP00000223321	32	PSMA2
ENSP00000300835	32	PRR14
ENSP00000217270	32	PROKR2
ENSP00000325638	32	PRM3
ENSP00000360035	32	PPP1R3D
ENSP00000361052	32	POMGNT1
ENSP00000272645	32	POLR2D
ENSP00000382362	32	PHB2
ENSP00000356563	32	PEX3
ENSP00000386653	32	PCDH20
ENSP00000302768	32	PAIP1
ENSP00000290291	32	OGFR
ENSP00000271452	32	NUF2
ENSP00000411012	32	NSMAF
ENSP00000220931	32	NCALD
ENSP00000326240	32	MYNN
ENSP00000368755	32	MUC12
ENSP00000350028	32	MOV10
ENSP00000223054	32	MOSPD3
ENSP00000225728	32	MED31
ENSP00000324444	32	MBIP
ENSP00000313021	32	MAK
ENSP00000273390	32	MAATS1
ENSP00000219168	32	LYRM1

ENSP00000331983	32	LRRC16A
ENSP00000298119	32	LRFN5
ENSP00000296252	32	LIPH
ENSP00000348704	32	KPNA5
ENSP00000407885	32	KIFC1
ENSP00000264773	32	KCNN2
ENSP00000344488	32	JPH1
ENSP00000267615	32	ITPK1
ENSP00000351190	32	ITIH2
ENSP00000386992	32	IPO11
ENSP00000355961	32	INTS7
ENSP00000289004	32	HPD
ENSP00000350348	32	GRM7
ENSP00000231188	32	GRM6
ENSP00000381129	32	GRK4
ENSP00000229264	32	GNB3
ENSP00000305260	32	GNB2
ENSP00000329632	32	FBXL7
ENSP00000259698	32	FAM65B
ENSP00000318298	32	FAM46A
ENSP00000288221	32	ERC2
ENSP00000217182	32	EEF1A2
ENSP00000264065	32	DNAJC10
ENSP00000328036	32	DGAT2L6
ENSP00000359361	32	DDX43
ENSP00000300087	32	DCTN5
ENSP00000285979	32	CYP2C18
ENSP00000295728	32	CRYBA2
ENSP00000339157	32	CPM
ENSP00000329419	32	COPB2
ENSP00000339292	32	CLDN14
ENSP00000263710	32	CLASP1
ENSP00000261751	32	CHRNB4
ENSP00000263780	32	CHMP2B
ENSP00000381428	32	CGGBP1
ENSP00000353720	32	CES1
ENSP00000380718	32	CDYL
ENSP00000286788	32	CCT8
ENSP00000291295	32	CALM3
ENSP00000415941	32	C4B
ENSP00000314792	32	BOLL
ENSP00000350314	32	BCO2
ENSP00000319974	32	BBX
ENSP00000376534	32	BAZ2B
ENSP00000283684	32	ATP8B1
ENSP00000367697	32	ATP6AP2

ENSP00000262623	32	ATP4A
ENSP00000263080	32	ASPA
ENSP00000399947	32	ASAH2C
ENSP00000348064	32	ART3
ENSP00000311538	32	ARR3
ENSP00000261636	32	ARL1
ENSP00000219409	32	ARHGDIG
ENSP00000351333	32	ARHGAP19
ENSP00000219660	32	AQP8
ENSP00000303769	32	AKNA
ENSP00000363649	32	AKAP2
ENSP00000387281	32	ADRA2B
ENSP00000349396	32	ABCD4
ENSP00000367991	32	8-Sep
ENSP00000334416	32	-
ENSP00000345532	32	-
ENSP00000357449	32	-
ENSP00000364287	32	-
ENSP00000377817	32	-
ENSP00000382881	32	-
ENSP00000391518	32	-
ENSP00000335091	31	ZNRF1
ENSP00000380293	31	ZNF99
ENSP00000366899	31	ZNF133
ENSP00000007699	31	YBX2
ENSP00000362576	31	YARS
ENSP00000324636	31	VRK3
ENSP00000327246	31	VIPR1
ENSP00000387019	31	UXS1
ENSP00000310590	31	USP36
ENSP00000359869	31	USP26
ENSP00000388330	31	TSFM
ENSP00000323926	31	TRPM8
ENSP00000274773	31	TRIM7
ENSP00000236979	31	TNP1
ENSP00000318409	31	TMEM176B
ENSP00000405279	31	TEN1
ENSP00000258770	31	TBRG4
ENSP00000356033	31	TAGAP
ENSP00000294168	31	TAF6L
ENSP00000377520	31	SYT12
ENSP00000216064	31	SUN2
ENSP00000308770	31	SULT1B1
ENSP00000335247	31	STARD10
ENSP00000168148	31	SPP2
ENSP00000256958	31	SLCO1B1

ENSP00000366299	31	SLC25A39
ENSP00000340465	31	SLC20A2
ENSP00000356906	31	SH2D1B
ENSP00000247225	31	SGPP1
ENSP00000355109	31	SFMBT2
ENSP00000322460	31	SCN4B
ENSP00000354733	31	SBNO2
ENSP00000256733	31	SAA2
ENSP00000255674	31	RTTN
ENSP00000314992	31	RRH
ENSP00000346037	31	RPLP1
ENSP00000255324	31	RNF17
ENSP00000283632	31	RMND5A
ENSP00000338671	31	RHOBTB1
ENSP00000352668	31	RBM12
ENSP00000265109	31	RAI14
ENSP00000216962	31	PYGB
ENSP00000332812	31	PTGDR2
ENSP00000296498	31	PRSS12
ENSP00000296666	31	PRRC1
ENSP00000390861	31	PPP1R37
ENSP00000356849	31	POGK
ENSP00000353940	31	PMEL
ENSP00000305995	31	PGK2
ENSP00000265810	31	PDLIM2
ENSP00000255531	31	PCDH19
ENSP00000230859	31	PAPD7
ENSP00000225538	31	P2RX1
ENSP00000361658	31	NUP188
ENSP00000257770	31	NT5E
ENSP00000332591	31	NPY2R
ENSP00000341083	31	NPR2
ENSP00000313088	31	NETO1
ENSP00000407964	31	NEDD1
ENSP00000358203	31	NBPF20
ENSP00000320488	31	NAP1L5
ENSP00000225927	31	NAGLU
ENSP00000301012	31	MVD
ENSP00000410910	31	MUSTN1
ENSP00000341171	31	MTSS1L
ENSP00000258874	31	MTHFS
ENSP00000303222	31	MTCH2
ENSP00000300952	31	MIDN
ENSP00000352455	31	MFAP5
ENSP00000337950	31	LYNX1
ENSP00000322270	31	LPHN2

ENSP00000270238	31	LMTK3
ENSP00000375622	31	LAIR1
ENSP00000242810	31	KLHL24
ENSP00000373648	31	KCNQ3
ENSP00000333496	31	KCND2
ENSP00000343274	31	INTS8
ENSP00000360860	31	IFIT5
ENSP00000348170	31	HP
ENSP00000386143	31	HEPN1
ENSP00000229330	31	HCFC2
ENSP00000312288	31	HADH
ENSP00000225567	31	GOSR2
ENSP00000408132	31	GOLGA6B
ENSP00000362656	31	GOLGA1
ENSP00000248564	31	GNG11
ENSP00000294409	31	GMEB1
ENSP00000300079	31	GLYATL1
ENSP00000277865	31	GLUD1
ENSP00000360829	31	GLT6D1
ENSP00000370737	31	GLDC
ENSP00000222214	31	GCDH
ENSP00000256680	31	FKBP11
ENSP00000246549	31	FFAR2
ENSP00000358165	31	FCGR1A
ENSP00000221347	31	FCGBP
ENSP00000347834	31	FBXL3
ENSP00000354497	31	FAN1
ENSP00000368362	31	FABP9
ENSP00000366135	31	EXOSC10
ENSP00000324105	31	ENO3
ENSP00000369677	31	EMILIN1
ENSP00000265840	31	ELMOD1
ENSP00000310040	31	EIF3F
ENSP00000313084	31	EGFLAM
ENSP00000256497	31	EDEM1
ENSP00000343244	31	DUSP21
ENSP00000333917	31	DUSP18
ENSP00000314030	31	DNAJA2
ENSP00000406091	31	DDR1
ENSP00000341625	31	CYB5A
ENSP00000280083	31	CTAGE5
ENSP00000274368	31	CRHBP
ENSP00000264193	31	CPOX
ENSP00000325548	31	CNDP2
ENSP00000353732	31	CLIP3
ENSP00000228438	31	CLEC2B

ENSP00000328983	31	CHST6
ENSP00000360918	31	CH25H
ENSP00000320084	31	CD276
ENSP00000258091	31	CCT7
ENSP00000282572	31	CCNO
ENSP00000285379	31	CA2
ENSP00000296452	31	BSN
ENSP00000327541	31	BLZF1
ENSP00000356094	31	AVPR1B
ENSP00000324172	31	ATP2B2
ENSP00000328631	31	ARMCX2
ENSP00000367615	31	APRT
ENSP00000336666	31	AP1S1
ENSP00000357943	31	ANXA9
ENSP00000274979	31	ANO7
ENSP00000297837	31	ANKS6
ENSP00000273588	31	AMT
ENSP00000314879	31	ALOXE3
ENSP00000360268	31	ALDH18A1
ENSP00000335369	31	AIFM3
ENSP00000339161	31	AIDA
ENSP00000308606	31	AGR3
ENSP00000252071	31	ACTR3C
ENSP00000355777	31	ACBD3
ENSP00000324287	31	ACAP2
ENSP00000301233	31	-
ENSP00000309482	31	-
ENSP00000349375	31	-
ENSP00000358546	31	-
ENSP00000380133	31	-
ENSP00000403092	31	-
ENSP00000405829	31	-
ENSP00000407526	31	-
ENSP00000311429	30	ZWILCH
ENSP00000359606	30	ZNHIT6
ENSP00000292579	30	ZNF250
ENSP00000332013	30	ZGPAT
ENSP00000409107	30	ZBTB42
ENSP00000320658	30	YARS2
ENSP00000347495	30	WARS
ENSP00000341819	30	VWC2
ENSP00000343348	30	VMAC
ENSP00000323516	30	UTS2R
ENSP00000342812	30	USP9Y
ENSP00000289865	30	USP21
ENSP00000362508	30	UGT1A4

ENSP00000259253	30	UGGT1
ENSP00000249042	30	TST
ENSP00000355299	30	TSEN15
ENSP00000366314	30	TRPM3
ENSP00000412124	30	TRIM67
ENSP00000262294	30	TRIM37
ENSP00000312599	30	TMEM70
ENSP00000372982	30	TMEM200C
ENSP00000281017	30	TMEM18
ENSP00000346883	30	TMEM116
ENSP00000237264	30	TBPL1
ENSP00000272902	30	SUMF1
ENSP00000359025	30	STXBP3
ENSP00000322652	30	SNX16
ENSP00000362285	30	SLC29A3
ENSP00000358640	30	SLC16A1
ENSP00000245312	30	SLC10A2
ENSP00000267197	30	SETD1B
ENSP00000295777	30	SERPINI1
ENSP00000354061	30	SCYL2
ENSP00000318094	30	SCMH1
ENSP00000286835	30	SCAF4
ENSP00000216036	30	RTDR1
ENSP00000401450	30	RPL28
ENSP00000377865	30	RPL23
ENSP00000332454	30	RIPK4
ENSP00000363516	30	RCAN3
ENSP00000373277	30	RBMS3
ENSP00000291576	30	PWP2
ENSP00000259457	30	PSMB7
ENSP00000266079	30	PRPF6
ENSP00000396732	30	PRDM7
ENSP00000310117	30	PPP1R14B
ENSP00000261534	30	POMT2
ENSP00000308315	30	PODN
ENSP00000301908	30	PNOC
ENSP00000348901	30	PLD3
ENSP00000387911	30	PARN
ENSP00000227638	30	PANX1
ENSP00000302108	30	PANK1
ENSP00000344996	30	PALM3
ENSP00000359085	30	NTNG1
ENSP00000318921	30	NOSTRIN
ENSP00000287667	30	NOMO1
ENSP00000340523	30	NMNAT3
ENSP00000365572	30	NME2

ENSP00000379680	30	NAGA
ENSP00000416753	30	MUC15
ENSP00000354632	30	MT-ATP6
ENSP00000311827	30	MSL2
ENSP00000321445	30	MSC
ENSP00000397900	30	MRO
ENSP00000356940	30	MOXD1
ENSP00000348081	30	MIB2
ENSP00000290663	30	MED8
ENSP00000258648	30	MED4
ENSP00000321070	30	ME2
ENSP00000318916	30	MARCO
ENSP00000300249	30	MAPRE2
ENSP00000272462	30	MALL
ENSP00000284818	30	LY96
ENSP00000326888	30	LIMS2
ENSP00000261434	30	LIAS
ENSP00000340011	30	KIR2DS4
ENSP00000368881	30	KIN
ENSP00000341466	30	KIF18B
ENSP00000306275	30	KCNK3
ENSP00000323587	30	ITLN1
ENSP00000341794	30	IL1F10
ENSP00000410447	30	IKZF2
ENSP00000369578	30	IFNW1
ENSP00000369571	30	IFNA14
ENSP00000351035	30	HSD17B11
ENSP00000325376	30	HNRNPM
ENSP00000199708	30	HBQ1
ENSP00000254854	30	GUCY2D
ENSP00000397351	30	GRID2IP
ENSP00000356251	30	GPR37L1
ENSP00000240986	30	GJA8
ENSP00000384214	30	GAGE12D
ENSP00000375748	30	FUT2
ENSP00000264433	30	FNIP2
ENSP00000386722	30	FCHSD2
ENSP00000243167	30	FAAH
ENSP00000350265	30	ENPP3
ENSP00000286523	30	ELMSAN1
ENSP00000320346	30	ELL3
ENSP00000262726	30	EFCAB6
ENSP00000221847	30	EBI3
ENSP00000307143	30	DONSON
ENSP00000316240	30	DNAJC14
ENSP00000265800	30	DMTN

ENSP00000384744	30	DERL3
ENSP00000257215	30	DAGLA
ENSP00000282018	30	CYSLTR2
ENSP00000368079	30	CYP4V2
ENSP00000353820	30	CYP2D6
ENSP00000338613	30	CRYGN
ENSP00000260988	30	CRYGB
ENSP00000378736	30	CRCP
ENSP00000285896	30	CNOT8
ENSP00000253475	30	CHMP1A
ENSP00000296449	30	CCDC36
ENSP00000328173	30	C1S
ENSP00000295297	30	C1QTNF7
ENSP00000307765	30	C17orf62
ENSP00000329930	30	C11orf82
ENSP00000228136	30	C11orf58
ENSP00000359682	30	BRS3
ENSP00000358554	30	BCAS2
ENSP00000361306	30	ATG4A
ENSP00000305502	30	ASCC2
ENSP00000277458	30	ASB6
ENSP00000349250	30	ARL4A
ENSP00000249116	30	APOBEC3A
ENSP00000345848	30	ANP32B
ENSP00000304292	30	ANKRD27
ENSP00000366015	30	ANGPTL7
ENSP00000264448	30	ALMS1
ENSP00000025301	30	AKAP11
ENSP00000311405	30	ADCY6
ENSP00000329468	30	ADAP2
ENSP00000268070	30	ADAMTS17
ENSP00000265150	30	-
ENSP00000265193	30	-
ENSP00000335272	30	-
ENSP00000339048	30	-
ENSP00000340166	30	-
ENSP00000355987	30	-
ENSP00000357358	30	-
ENSP00000364905	30	-
ENSP00000380148	30	-
ENSP00000381227	30	-
ENSP00000385395	30	-
ENSP00000390728	30	-
ENSP00000399750	30	-
ENSP00000408311	30	-
ENSP00000415575	30	-

ENSP00000420811	30	-
ENSP00000325018	29	ZNF569
ENSP00000196489	29	ZNF416
ENSP00000307604	29	ZBTB5
ENSP00000370616	29	ZBED1
ENSP00000339245	29	YTHDC1
ENSP00000417573	29	YIPF6
ENSP00000368727	29	XDH
ENSP00000324763	29	WFIKKN1
ENSP00000267973	29	WDR61
ENSP00000295024	29	WDR26
ENSP00000381436	29	VGLL3
ENSP00000264689	29	UFSP2
ENSP00000301365	29	TRPV3
ENSP00000398224	29	TRIM15
ENSP00000416959	29	TRA2B
ENSP00000265388	29	TNPO3
ENSP00000361636	29	TNNC2
ENSP00000275954	29	TMEM47
ENSP00000363189	29	TMEM222
ENSP00000324651	29	TMEM108
ENSP00000220420	29	TGM5
ENSP00000380929	29	TCP10
ENSP00000347719	29	TBCD
ENSP00000303325	29	TACR3
ENSP00000263033	29	SYTL4
ENSP00000243918	29	SYS1
ENSP00000350719	29	SYNE2
ENSP00000384015	29	SUN1
ENSP00000348132	29	STK31
ENSP00000309831	29	SNUPN
ENSP00000349823	29	SMARCAL1
ENSP00000289952	29	SLC39A14
ENSP00000266579	29	SLC38A4
ENSP00000261892	29	SLC24A1
ENSP00000279027	29	SLC13A3
ENSP00000345243	29	SIGLEC10
ENSP00000342962	29	SERINC1
ENSP00000347117	29	SEMA4A
ENSP00000297109	29	SAP30L
ENSP00000270225	29	SAE1
ENSP00000260563	29	RTCA
ENSP00000321330	29	RNF144A
ENSP00000363787	29	RING1
ENSP00000253796	29	RAMP2
ENSP00000310088	29	PTMS

ENSP00000301974	29	PTAFR
ENSP00000291041	29	PSKH1
ENSP00000324127	29	PSD3
ENSP00000317445	29	PSAPL1
ENSP00000382485	29	PROL1
ENSP00000215587	29	POLR2E
ENSP00000337701	29	PNPLA2
ENSP00000323511	29	PNKP
ENSP00000361508	29	PLTP
ENSP00000365505	29	PLP2
ENSP00000266095	29	PISD
ENSP00000279036	29	PIGT
ENSP00000008938	29	PGLYRP1
ENSP00000356350	29	PCMT1
ENSP00000353512	29	PARP9
ENSP00000300291	29	NUDT21
ENSP00000382392	29	NOP2
ENSP00000233027	29	NEK4
ENSP00000387128	29	NCKAP5
ENSP00000348349	29	MYO9A
ENSP00000297908	29	MRRF
ENSP00000349575	29	MOB3A
ENSP00000371811	29	MICU2
ENSP00000215957	29	MICALL1
ENSP00000256194	29	MICAL2
ENSP00000354920	29	METTL13
ENSP00000384115	29	MEI1
ENSP00000263390	29	MED26
ENSP00000362948	29	MED18
ENSP00000290429	29	MCAT
ENSP00000383690	29	MASP2
ENSP00000296135	29	LZTFL1
ENSP00000276654	29	LRP12
ENSP00000260702	29	LOXL4
ENSP00000360104	29	LEPROT
ENSP00000367075	29	KLHL1
ENSP00000297625	29	KIAA1161
ENSP00000321584	29	IMPDH2
ENSP00000363745	29	HNRNPR
ENSP00000336856	29	HMG20A
ENSP00000398064	29	HIGD1A
ENSP00000247815	29	HELB
ENSP00000361662	29	GUCA2B
ENSP00000230361	29	GUCA1B
ENSP00000379372	29	GTF2A2
ENSP00000301659	29	GSDMA

ENSP00000264954	29	GRPEL1
ENSP00000218316	29	GPR50
ENSP00000370767	29	GP2
ENSP00000205061	29	GLG1
ENSP00000351706	29	GK2
ENSP00000348668	29	GALNT6
ENSP00000351113	29	FRYL
ENSP00000381549	29	FRMD4B
ENSP00000365145	29	FOXS1
ENSP00000275461	29	FERD3L
ENSP00000248076	29	F2RL3
ENSP00000287675	29	EXOG
ENSP00000341743	29	ENSA
ENSP00000337146	29	ENOX2
ENSP00000263277	29	EHD2
ENSP00000316193	29	DNASE1L3
ENSP00000262415	29	DHX8
ENSP00000221403	29	DHDH
ENSP00000350266	29	DENND2D
ENSP00000296550	29	DCLK2
ENSP00000228476	29	DAO
ENSP00000304822	29	CSN3
ENSP00000363590	29	CSGALNACT2
ENSP00000302105	29	CRYGA
ENSP00000352544	29	CPLX2
ENSP00000264389	29	COPS4
ENSP00000361834	29	COL9A2
ENSP00000229195	29	CNOT2
ENSP00000229329	29	CMAS
ENSP00000353073	29	CLN3
ENSP00000254868	29	CLEC10A
ENSP00000264001	29	CKLF
ENSP00000317337	29	CD300LB
ENSP00000311035	29	CD200R1
ENSP00000235933	29	CD160
ENSP00000349238	29	CCDC88B
ENSP00000219197	29	CBLN1
ENSP00000399436	29	C6orf15
ENSP00000333737	29	C1QTNF9
ENSP00000340864	29	C1QTNF1
ENSP00000333283	29	C17orf70
ENSP00000378409	29	C10orf54
ENSP00000333919	29	BTLA
ENSP00000367471	29	BTBD3
ENSP00000410994	29	BRD2
ENSP00000304987	29	BFSP2

ENSP00000295240	29	BBS5
ENSP00000252934	29	ATXN10
ENSP00000282050	29	ATP5A1
ENSP00000321195	29	ATP11B
ENSP00000310321	29	ATG13
ENSP00000368030	29	ATAD3A
ENSP00000269299	29	ASGR1
ENSP00000320893	29	ASB8
ENSP00000316520	29	AMPD1
ENSP00000314649	29	ALDH5A1
ENSP00000253792	29	ACLY
ENSP00000252889	29	-
ENSP00000297163	29	-
ENSP00000304843	29	-
ENSP00000314528	29	-
ENSP00000318024	29	-
ENSP00000343089	29	-
ENSP00000344667	29	-
ENSP00000348197	29	-
ENSP00000391266	29	-
ENSP00000392496	29	-
ENSP00000392573	29	-
ENSP00000399011	29	-
ENSP00000403004	29	-
ENSP00000319829	28	ZNF687
ENSP00000420418	28	ZNF398
ENSP00000342019	28	ZNF189
ENSP00000333192	28	ZFP1
ENSP00000299138	28	VPS35
ENSP00000370479	28	VCX3A
ENSP00000313811	28	USP20
ENSP00000261601	28	USP14
ENSP00000005226	28	USH1C
ENSP00000367756	28	UNC13B
ENSP00000232607	28	UMPS
ENSP00000311648	28	UGT8
ENSP00000363338	28	UBXN11
ENSP00000013070	28	UBR7
ENSP00000338348	28	UBE2G2
ENSP00000264255	28	TXNDC9
ENSP00000307701	28	TSPAN5
ENSP00000391879	28	TRIM26
ENSP00000357906	28	TNFAIP8L2
ENSP00000287773	28	TMEM171
ENSP00000341657	28	THYN1
ENSP00000352678	28	SUPV3L1

ENSP00000392270	28	STRAP
ENSP00000348026	28	STAU2
ENSP00000246895	28	STATH
ENSP00000382518	28	SRL
ENSP00000397911	28	SP140L
ENSP00000338191	28	SNTB2
ENSP00000369135	28	SLCO6A1
ENSP00000261196	28	SLCO1B3
ENSP00000305974	28	SLCO1A2
ENSP00000267842	28	SLC27A2
ENSP00000352497	28	SLC25A44
ENSP00000352167	28	SLC25A29
ENSP00000279178	28	SLC22A9
ENSP00000263160	28	SLC17A6
ENSP00000277010	28	SIGMAR1
ENSP00000265175	28	SEC24B
ENSP00000360569	28	SCP2
ENSP00000346534	28	SCN8A
ENSP00000346990	28	SCARA5
ENSP00000307275	28	SCAMP3
ENSP00000353769	28	SCAF1
ENSP00000240285	28	RDH10
ENSP00000329454	28	RCAN2
ENSP00000216840	28	RABGGTA
ENSP00000358042	28	QRSL1
ENSP00000303088	28	POLR3D
ENSP00000283977	28	PGM3
ENSP00000322192	28	PGA3
ENSP00000226524	28	PF4V1
ENSP00000351789	28	PELI1
ENSP00000360327	28	PARS2
ENSP00000216277	28	PAPOLA
ENSP00000245544	28	NUP85
ENSP00000251074	28	NUP37
ENSP00000381293	28	NSF
ENSP00000277942	28	NPFFR1
ENSP00000343497	28	NOSIP
ENSP00000331857	28	NLRP9
ENSP00000360464	28	NKAP
ENSP00000369176	28	NDUFB6
ENSP00000239926	28	MYOT
ENSP00000354335	28	MPEG1
ENSP00000315702	28	MOB4
ENSP00000301920	28	MMADHC
ENSP00000339881	28	MIOS
ENSP00000407561	28	MICB

ENSP00000354583	28	MFAP3L
ENSP00000265350	28	MED20
ENSP00000294507	28	LAPTM5
ENSP00000339634	28	KIR2DL4
ENSP00000286452	28	KIF5A
ENSP00000263181	28	KIF18A
ENSP00000359404	28	KIAA1107
ENSP00000257981	28	KCNH3
ENSP00000319918	28	ITFG1
ENSP00000311492	28	ISX
ENSP00000262032	28	IKZF4
ENSP00000260191	28	HTR3B
ENSP00000255631	28	HSPBP1
ENSP00000363794	28	HSD17B8
ENSP00000307951	28	HIGD2B
ENSP00000378624	28	HERC4
ENSP00000361245	28	HECTD3
ENSP00000264424	28	GUCY1B3
ENSP00000314223	28	GPR88
ENSP00000359539	28	GOT1
ENSP00000401018	28	GINS3
ENSP00000343636	28	GFRAL
ENSP00000373572	28	FOXI2
ENSP00000344446	28	FGD6
ENSP00000220849	28	EIF3E
ENSP00000364119	28	EIF2S2
ENSP00000391944	28	EEF1D
ENSP00000351811	28	EDC4
ENSP00000354737	28	ECI2
ENSP00000315265	28	DYNAP
ENSP00000190165	28	DMRT3
ENSP00000305785	28	DMRT2
ENSP00000228027	28	DGAT2
ENSP00000158771	28	DERL2
ENSP00000320813	28	DEFB104A
ENSP00000360958	28	CYP4A22
ENSP00000361562	28	CTSA
ENSP00000402608	28	CPS1
ENSP00000305613	28	CPLX1
ENSP00000368824	28	CNKSR2
ENSP00000339723	28	CIR1
ENSP00000276055	28	CHST7
ENSP00000362207	28	CHST3
ENSP00000327075	28	CD300LF
ENSP00000332659	28	CCR4
ENSP00000293276	28	CCL15

ENSP00000262059	28	CALCOCO1
ENSP00000251143	28	C16orf62
ENSP00000256319	28	C14orf1
ENSP00000309595	28	C10orf2
ENSP00000394316	28	BZW1
ENSP00000216267	28	BRD1
ENSP00000414880	28	B3GALT4
ENSP00000299178	28	AVPR1A
ENSP00000302898	28	AURKC
ENSP00000332247	28	ATP6V0A2
ENSP00000262030	28	ATP5B
ENSP00000358896	28	AS3MT
ENSP00000228936	28	ART4
ENSP00000265748	28	ANLN
ENSP00000302232	28	ALKBH3
ENSP00000267584	28	AK7
ENSP00000302620	28	AGXT
ENSP00000328938	28	AFMID
ENSP00000269143	28	AFG3L2
ENSP00000251648	28	-
ENSP00000274345	28	-
ENSP00000344182	28	-
ENSP00000388822	28	-
ENSP00000388913	28	-
ENSP00000391434	28	-
ENSP00000374359	27	ZXDC
ENSP00000335384	27	ZPBP2
ENSP00000387091	27	ZNF365
ENSP00000419728	27	ZNF322
ENSP00000352821	27	ZDHHC14
ENSP00000337839	27	ZCCHC7
ENSP00000384290	27	WDR60
ENSP00000358126	27	VPS45
ENSP00000334808	27	USP16
ENSP00000320076	27	USH1G
ENSP00000203407	27	UQCRC1
ENSP00000311760	27	TTC9B
ENSP00000332152	27	TRIM52
ENSP00000254816	27	TRIM47
ENSP00000182527	27	TRAM2
ENSP00000217121	27	TPD52L2
ENSP00000331288	27	TMEM173
ENSP00000315387	27	TMEM102
ENSP00000327349	27	TMCC1
ENSP00000330433	27	TM2D3
ENSP00000061240	27	TLL1

ENSP00000259782	27	TINAG
ENSP00000356256	27	TIMM17A
ENSP00000370741	27	THUMPD1
ENSP00000273368	27	TAGLN3
ENSP00000245934	27	SYMPK
ENSP00000358142	27	SV2A
ENSP00000351075	27	SUSD2
ENSP00000265073	27	SUB1
ENSP00000305255	27	STX8
ENSP00000363054	27	STX12
ENSP00000267540	27	STON2
ENSP00000255008	27	SSTR4
ENSP00000420195	27	SRSF10
ENSP00000295213	27	SPATA18
ENSP00000024061	27	SLC45A4
ENSP00000233535	27	SLC30A3
ENSP00000263093	27	SLC27A5
ENSP00000341682	27	SLC26A9
ENSP00000357844	27	SLC16A10
ENSP00000333656	27	SIGIRR
ENSP00000309186	27	SH3RF3
ENSP00000271628	27	SF3B4
ENSP00000278407	27	SERPING1
ENSP00000261994	27	SERPINA10
ENSP00000306881	27	SEC23A
ENSP00000293502	27	SDR9C7
ENSP00000296506	27	SCRG1
ENSP00000278947	27	SCN2B
ENSP00000339145	27	RRP1B
ENSP00000420465	27	RNF182
ENSP00000244061	27	RNF114
ENSP00000272324	27	REG3G
ENSP00000353198	27	PYY
ENSP00000322016	27	PUF60
ENSP00000325589	27	PTPRCAP
ENSP00000272847	27	PTH2R
ENSP00000211413	27	PRRT1
ENSP00000343103	27	PRMT7
ENSP00000381461	27	PPME1
ENSP00000361302	27	POMT1
ENSP00000414208	27	POM121C
ENSP00000257622	27	POM121
ENSP00000302478	27	POLR1D
ENSP00000357452	27	PMVK
ENSP00000297540	27	PHAX
ENSP00000270776	27	PGD

ENSP00000349543	27	PEX2
ENSP00000265322	27	PECR
ENSP00000284288	27	PANX3
ENSP00000361104	27	OBP2B
ENSP00000329448	27	NUTM1
ENSP00000261396	27	NUP133
ENSP00000222674	27	NPVF
ENSP00000360660	27	NPDC1
ENSP00000378089	27	NMB
ENSP00000232978	27	NKTR
ENSP00000299166	27	NDUFB8
ENSP00000290810	27	NAE1
ENSP00000404705	27	NAALADL2
ENSP00000286733	27	NAAA
ENSP00000418734	27	MYSM1
ENSP00000217939	27	MXRA5
ENSP00000328251	27	MINA
ENSP00000367486	27	MEIG1
ENSP00000274473	27	MEGF10
ENSP00000325425	27	MAT2B
ENSP00000228740	27	LTA4H
ENSP00000371220	27	LOH12CR2
ENSP00000321546	27	LOH12CR1
ENSP00000233714	27	LANCL1
ENSP00000377582	27	KRTAP4-12
ENSP00000284669	27	KLHL41
ENSP00000393379	27	KIF5C
ENSP00000361473	27	KDM4A
ENSP00000310557	27	KCNE3
ENSP00000360195	27	KANK4
ENSP00000374852	27	IGLV2-8
ENSP00000350570	27	IFT20
ENSP00000247933	27	IDUA
ENSP00000392896	27	HSBP1
ENSP00000301790	27	HRASLS5
ENSP00000353099	27	HLA-DRB1
ENSP00000350767	27	HIST4H4
ENSP00000408681	27	HEATR5A
ENSP00000225724	27	GOSR1
ENSP00000294117	27	GNG3
ENSP00000274093	27	GLRA3
ENSP00000282841	27	GGPS1
ENSP00000368589	27	GFOD1
ENSP00000313050	27	GBAS
ENSP00000357644	27	GATAD2B
ENSP00000002165	27	FUCA2

ENSP00000356723	27	FMO4
ENSP00000269856	27	FEM1A
ENSP00000294800	27	FCGR3B
ENSP00000292853	27	FBXO27
ENSP00000369335	27	FBXO18
ENSP00000354958	27	FAM189B
ENSP00000328347	27	FAM110C
ENSP00000323275	27	DYX1C1
ENSP00000354111	27	DNAJC5
ENSP00000263579	27	DCPS
ENSP00000285949	27	CYP26C1
ENSP00000406145	27	CTXN2
ENSP00000368140	27	CRLS1
ENSP00000318804	27	CRLF3
ENSP00000265997	27	CPEB3
ENSP00000304544	27	COMMD5
ENSP00000351310	27	COL6A6
ENSP00000264828	27	COL5A3
ENSP00000297668	27	CNTNAP3
ENSP00000337512	27	CMTR2
ENSP00000316948	27	CLK4
ENSP00000381290	27	CHTF8
ENSP00000371278	27	CDC37L1
ENSP00000379566	27	CCHCR1
ENSP00000373586	27	C2orf54
ENSP00000334229	27	C2CD5
ENSP00000266542	27	C1RL
ENSP00000298943	27	C1QL3
ENSP00000416700	27	C11orf72
ENSP00000310649	27	BRSK1
ENSP00000220531	27	BLOC1S6
ENSP00000253413	27	ATP6V1E1
ENSP00000216442	27	ATP6V1D
ENSP00000370639	27	ASMT
ENSP00000347140	27	ASGR2
ENSP00000320252	27	ASCC3
ENSP00000370526	27	ARSE
ENSP00000380431	27	ARPC4
ENSP00000356969	27	APOA2
ENSP00000292807	27	AP2M1
ENSP00000359096	27	AMY1C
ENSP00000345774	27	ALDH3A2
ENSP00000375705	27	AHCTF1
ENSP00000401287	27	AGPAT1
ENSP00000233710	27	ACADL
ENSP00000324511	27	1-Sep

ENSP00000222279	27	-
ENSP00000370135	27	-
ENSP00000382303	27	-
ENSP00000392711	27	-
ENSP00000401170	27	-
ENSP00000415376	27	-
ENSP00000237937	26	ZFAND5
ENSP00000362918	26	YTHDF2
ENSP00000319303	26	XRRA1
ENSP00000255305	26	XPO4
ENSP00000311184	26	WFIKKN2
ENSP00000298125	26	WDFY2
ENSP00000320957	26	VGLL2
ENSP00000397453	26	UBE2W
ENSP00000369465	26	TXLNG
ENSP00000415328	26	TRIM26
ENSP00000352708	26	TRAPPC2
ENSP00000343577	26	TPSAB1
ENSP00000272424	26	TPRKB
ENSP00000226225	26	TNFAIP1
ENSP00000301272	26	TMIGD2
ENSP00000362562	26	TMEM54
ENSP00000405926	26	TMED7
ENSP00000325561	26	TMC8
ENSP00000358931	26	TKTL1
ENSP00000356807	26	TIPRL
ENSP00000245838	26	THOC2
ENSP00000385995	26	THADA
ENSP00000295201	26	TEKT4
ENSP00000356536	26	TEDDM1
ENSP00000361707	26	TCEAL1
ENSP00000321826	26	STXBP5
ENSP00000375777	26	STRN4
ENSP00000373684	26	STK38L
ENSP00000372817	26	STK19
ENSP00000244763	26	SSR1
ENSP00000369611	26	SPZ1
ENSP00000261866	26	SPG11
ENSP00000365811	26	SPAG6
ENSP00000251775	26	SNX4
ENSP00000400591	26	SNRPE
ENSP00000371872	26	SLC35G5
ENSP00000259392	26	SLC31A2
ENSP00000265631	26	SLC25A13
ENSP00000245407	26	SLC22A5
ENSP00000005587	26	SKAP2

ENSP00000404143	26	SH3YL1
ENSP00000333203	26	SERPINA5
ENSP00000357998	26	SEC63
ENSP00000262544	26	SEC23B
ENSP00000353094	26	SDF4
ENSP00000234677	26	SARS
ENSP00000278572	26	RPS3
ENSP00000237530	26	RPN2
ENSP00000378160	26	RPL34
ENSP00000218340	26	RP2
ENSP00000362555	26	RNF19B
ENSP00000265981	26	RNF141
ENSP00000360441	26	RHOXF2
ENSP00000418667	26	RGAG4
ENSP00000303206	26	REG1B
ENSP00000222008	26	RABAC1
ENSP00000418661	26	PTRH1
ENSP00000365908	26	PRUNE2
ENSP00000354280	26	PRSS3
ENSP00000375928	26	PRRC2C
ENSP00000358106	26	PREP
ENSP00000261308	26	PPWD1
ENSP00000228425	26	PPFIBP1
ENSP00000272198	26	PPFIA4
ENSP00000221957	26	PLIN3
ENSP00000354775	26	PJA2
ENSP00000369820	26	PIGA
ENSP00000333024	26	PHF7
ENSP00000262043	26	PHF3
ENSP00000368439	26	PCYT1B
ENSP00000394936	26	ORM2
ENSP00000348752	26	ORAI2
ENSP00000336764	26	OPRL1
ENSP00000376886	26	NME1-NME2
ENSP00000273598	26	NICN1
ENSP00000348394	26	NCDN
ENSP00000342137	26	NCCRP1
ENSP00000251588	26	NARFL
ENSP00000262384	26	N4BP1
ENSP00000324810	26	MVB12A
ENSP00000302021	26	MUC7
ENSP00000309782	26	MRGPRF
ENSP00000307423	26	MGAT2
ENSP00000364685	26	MFAP2
ENSP00000298717	26	METTL3
ENSP00000387429	26	MDC1

ENSP00000324175	26	MAMSTR
ENSP00000312001	26	LRRN3
ENSP00000373853	26	LRRC4B
ENSP00000340519	26	LRRC37B
ENSP00000340688	26	LPHN1
ENSP00000238983	26	LIPF
ENSP00000334869	26	LCE1A
ENSP00000216237	26	L3MBTL2
ENSP00000382592	26	KRTAP5-3
ENSP00000293670	26	KRT83
ENSP00000259154	26	KCTD3
ENSP00000319591	26	KCND3
ENSP00000361418	26	IPO13
ENSP00000355205	26	INO80
ENSP00000355010	26	IGSF1
ENSP00000375003	26	IGHV1-2
ENSP00000299238	26	HPS6
ENSP00000359680	26	HOGA1
ENSP00000316042	26	HNRNPA0
ENSP00000350848	26	HMGN5
ENSP00000356811	26	HBS1L
ENSP00000314380	26	GRINA
ENSP00000261654	26	GPR133
ENSP00000373363	26	GOLM1
ENSP00000355556	26	GNG4
ENSP00000334051	26	GNAL
ENSP00000345868	26	GJB4
ENSP00000351170	26	GDA
ENSP00000410833	26	GBE1
ENSP00000343234	26	GAL3ST1
ENSP00000335655	26	FMNL3
ENSP00000248598	26	FGL2
ENSP00000229971	26	FBXL4
ENSP00000261488	26	ENOX1
ENSP00000273130	26	DYNC1LI1
ENSP00000384084	26	DTNB
ENSP00000261615	26	DPEP1
ENSP00000416583	26	DERA
ENSP00000241051	26	DEPDC7
ENSP00000372126	26	DEFA1
ENSP00000330349	26	DDX41
ENSP00000280665	26	DCP1B
ENSP00000333212	26	CYP2U1
ENSP00000388119	26	CT45A6
ENSP00000383115	26	COLEC12
ENSP00000338783	26	CHST5

ENSP00000258385	26	CHRND
ENSP00000200676	26	CETP
ENSP00000259053	26	CD302
ENSP00000325681	26	CCZ1
ENSP00000347005	26	C7orf55-LUC7L2
ENSP00000301246	26	C19orf33
ENSP00000256969	26	C12orf39
ENSP00000267103	26	C12orf10
ENSP00000297290	26	BRI3
ENSP00000304676	26	ATOH8
ENSP00000249822	26	ARPP19
ENSP00000407333	26	ARHGAP23
ENSP00000346378	26	APBB3
ENSP00000352603	26	AP4M1
ENSP00000351416	26	ANKRD17
ENSP00000330484	26	AMY1B
ENSP00000359100	26	AMY1A
ENSP00000342564	26	ALDH6A1
ENSP00000255084	26	ALDH3B2
ENSP00000264409	26	AGPAT9
ENSP00000416834	26	ADIG
ENSP00000333666	26	ADI1
ENSP00000316924	26	ACSS1
ENSP00000348775	26	ACOX3
ENSP00000359233	26	ABCD3
ENSP00000265316	26	ABCB6
ENSP00000263817	26	ABCB11
ENSP00000007633	26	-
ENSP00000251275	26	-
ENSP00000261364	26	-
ENSP00000296472	26	-
ENSP00000304997	26	-
ENSP00000315139	26	-
ENSP00000326966	26	-
ENSP00000352498	26	-
ENSP00000370488	26	-
ENSP00000384279	26	-
ENSP00000387358	26	-
ENSP00000400971	26	-
ENSP00000415200	26	-
ENSP00000417383	26	-
ENSP00000304593	25	ZNHIT1
ENSP00000217130	25	ZNF512B
ENSP00000277225	25	ZNF462
ENSP00000353515	25	ZNF461
ENSP00000363747	25	ZNF33A

ENSP00000268655	25	ZNF174
ENSP00000366273	25	ZNF157
ENSP00000290974	25	ZFYVE28
ENSP00000367069	25	ZFAT
ENSP00000296326	25	ZDHHC19
ENSP00000326200	25	ZBTB11
ENSP00000223369	25	YKT6
ENSP00000207870	25	XYLB
ENSP00000356792	25	XCL1
ENSP00000242796	25	WDR83
ENSP00000295888	25	WDFY3
ENSP00000417175	25	VAPB
ENSP00000054668	25	UTS2
ENSP00000350009	25	USP33
ENSP00000257548	25	USP30
ENSP00000263897	25	USE1
ENSP00000305221	25	UGT2B4
ENSP00000418532	25	UGT1A3
ENSP00000222402	25	UBE2D4
ENSP00000303908	25	TWF2
ENSP00000356411	25	TROVE2
ENSP00000257909	25	TROAP
ENSP00000334657	25	TRIM46
ENSP00000398355	25	TRIM39
ENSP00000339659	25	TRIM2
ENSP00000328998	25	TRAK1
ENSP00000299427	25	TPP1
ENSP00000363942	25	TP53INP2
ENSP00000259365	25	TMOD1
ENSP00000363031	25	THEMIS2
ENSP00000351614	25	SYN3
ENSP00000312054	25	SUGCT
ENSP00000287908	25	STEAP2
ENSP00000312415	25	SSX5
ENSP00000265295	25	SPDL1
ENSP00000334879	25	SOWAHB
ENSP00000289575	25	SLCO2B1
ENSP00000354966	25	SLC9A8
ENSP00000298923	25	SLC6A5
ENSP00000369396	25	SLC4A11
ENSP00000359892	25	SLC44A5
ENSP00000261867	25	SLC30A4
ENSP00000286749	25	SLC28A1
ENSP00000378920	25	SLC26A6
ENSP00000344831	25	SLC25A40
ENSP00000258403	25	SLC19A3

ENSP00000221485	25	SLC17A7
ENSP00000333667	25	SHMT2
ENSP00000224807	25	SFXN3
ENSP00000318861	25	SF3B2
ENSP00000373354	25	SETMAR
ENSP00000326477	25	SETDB2
ENSP00000255175	25	SERINC3
ENSP00000327197	25	SENP5
ENSP00000364965	25	SECISBP2
ENSP00000295930	25	RSRC1
ENSP00000393393	25	RPL35A
ENSP00000363018	25	RPL10A
ENSP00000216487	25	RIN3
ENSP00000344779	25	RHBDD1
ENSP00000256953	25	RERG
ENSP00000377303	25	RENBP
ENSP00000317473	25	RABGGTB
ENSP00000354871	25	PPEF1
ENSP00000221249	25	PNPLA6
ENSP00000413897	25	PLA2G4E
ENSP00000396045	25	PLA2G4B
ENSP00000299001	25	PIWIL4
ENSP00000371393	25	PGM2
ENSP00000255389	25	PEMT
ENSP00000365388	25	PDSS1
ENSP00000334910	25	PDE2A
ENSP00000243052	25	PDE1B
ENSP00000253807	25	PCDHAC1
ENSP00000368887	25	PAK1IP1
ENSP00000281589	25	PABPC3
ENSP00000308361	25	P2RY14
ENSP00000196371	25	OXCT1
ENSP00000259396	25	ORM1
ENSP00000322249	25	ORAI3
ENSP00000307447	25	OR2AG1
ENSP00000358951	25	OPN1MW
ENSP00000254508	25	NUP210
ENSP00000281081	25	NUBPL
ENSP00000277746	25	NRBF2
ENSP00000305877	25	NMUR1
ENSP00000265191	25	NME5
ENSP00000342300	25	NELFCD
ENSP00000344871	25	MYO1F
ENSP00000311888	25	MGAT1
ENSP00000384398	25	MEX3D
ENSP00000264079	25	MCOLN1

ENSP00000304198	25	MBD3L1
ENSP00000309689	25	LRCH4
ENSP00000303366	25	LMAN2
ENSP00000339621	25	LCN6
ENSP00000358861	25	LCA5
ENSP00000333887	25	KRTAP22-1
ENSP00000328062	25	KIAA1033
ENSP00000295101	25	KCNJ3
ENSP00000331727	25	KCNH7
ENSP00000271751	25	KCNH1
ENSP00000265969	25	KCNC1
ENSP00000386738	25	KCMF1
ENSP00000379908	25	ICA1
ENSP00000361927	25	HNRNPH2
ENSP00000409127	25	HLA-DQA1
ENSP00000359899	25	HCRTR2
ENSP00000261208	25	HAL
ENSP00000234389	25	GRIN3B
ENSP00000362183	25	GRIK3
ENSP00000363136	25	GPR3
ENSP00000264718	25	GPN1
ENSP00000262958	25	GNA15
ENSP00000336832	25	GJD3
ENSP00000315474	25	GIMAP7
ENSP00000361207	25	GHITM
ENSP00000338964	25	GGT7
ENSP00000370962	25	GGT6
ENSP00000385721	25	GGT2
ENSP00000265643	25	GAL
ENSP00000336829	25	FGG
ENSP00000246553	25	FFAR1
ENSP00000278840	25	FADS2
ENSP00000267113	25	ESYT1
ENSP00000361592	25	ERMAP
ENSP00000406027	25	EPM2AIP1
ENSP00000332656	25	ENPP7
ENSP00000245046	25	EMC3
ENSP00000256398	25	ELP3
ENSP00000319910	25	EIF3M
ENSP00000368927	25	EIF1AX
ENSP00000365147	25	EFHD2
ENSP00000404381	25	DNAJB14
ENSP00000367815	25	DNAAF1
ENSP00000382260	25	DGKB
ENSP00000350698	25	DDX47
ENSP00000364188	25	DDOST

ENSP00000303356	25	DCXR
ENSP00000299441	25	DCHS1
ENSP00000408860	25	CYP21A2
ENSP00000310832	25	CTSF
ENSP00000288400	25	CREB3L1
ENSP00000369979	25	CPXM1
ENSP00000297135	25	COG5
ENSP00000300107	25	CLPX
ENSP00000387122	25	CLEC16A
ENSP00000261883	25	CILP
ENSP00000378812	25	CHCHD2
ENSP00000257287	25	CEP135
ENSP00000343818	25	CDK5RAP2
ENSP00000292574	25	CCDC105
ENSP00000345436	25	CASKIN1
ENSP00000300105	25	CACNG2
ENSP00000334198	25	CACNA1H
ENSP00000332162	25	C1orf64
ENSP00000400718	25	BCAP29
ENSP00000381844	25	ATL3
ENSP00000311318	25	ATG4D
ENSP00000251089	25	ANGEL1
ENSP00000331817	25	ALYREF
ENSP00000216194	25	ADSL
ENSP00000257359	25	ADAMTS8
ENSP00000370443	25	ADAMTS6
ENSP00000281182	25	ACAD8
ENSP00000269080	25	ABCA8
ENSP00000264893	25	11-Sep
ENSP00000219827	25	-
ENSP00000306535	25	-
ENSP00000322498	25	-
ENSP00000327557	25	-
ENSP00000350710	25	-
ENSP00000373555	25	-
ENSP00000387708	25	-
ENSP00000390683	25	-
ENSP00000342002	24	ZNF93
ENSP00000264447	24	ZNF638
ENSP00000269973	24	ZNF45
ENSP00000378792	24	ZNF169
ENSP00000291900	24	ZER1
ENSP00000383599	24	WDR47
ENSP00000328671	24	WDR4
ENSP00000320563	24	WDFY4
ENSP00000263559	24	VPS26A

ENSP00000339957	24	USP47
ENSP00000343838	24	UGT1A10
ENSP00000361289	24	UCK1
ENSP00000366819	24	UCHL3
ENSP00000296328	24	UBXN7
ENSP00000291552	24	U2AF1
ENSP00000370031	24	TTC8
ENSP00000382544	24	TSSK2
ENSP00000298355	24	TRIM9
ENSP00000328875	24	TRAK2
ENSP00000353785	24	TPPP
ENSP00000337022	24	TNNI1
ENSP00000385470	24	TNFSF18
ENSP00000275780	24	TLK2
ENSP00000261881	24	TIPIN
ENSP00000355736	24	TCTE3
ENSP00000011473	24	SYPL1
ENSP00000268164	24	ST8SIA2
ENSP00000295702	24	SSR2
ENSP00000328023	24	SRPR
ENSP00000260324	24	SQRDL
ENSP00000358605	24	SMNDC1
ENSP00000326603	24	SMCHD1
ENSP00000335557	24	SLX4IP
ENSP00000361381	24	SLC6A9
ENSP00000225665	24	SLC25A11
ENSP00000370381	24	SLC12A1
ENSP00000215727	24	SERPIND1
ENSP00000331368	24	SERPINB8
ENSP00000337688	24	SEC62
ENSP00000356146	24	SCAF8
ENSP00000349359	24	SASH3
ENSP00000358059	24	RTN4IP1
ENSP00000229554	24	RSPH4A
ENSP00000295704	24	RNF25
ENSP00000261593	24	RNF138
ENSP00000341361	24	RNF13
ENSP00000356272	24	RMND1
ENSP00000376583	24	RIC8B
ENSP00000217999	24	RHOXF1
ENSP00000358099	24	RGS10
ENSP00000265881	24	REXO2
ENSP00000368295	24	REXO1L1P
ENSP00000364582	24	RCC2
ENSP00000254661	24	RAMP1
ENSP00000005995	24	PRSS21

ENSP00000412436	24	PRH1
ENSP00000256103	24	PMP2
ENSP00000366977	24	PLEKHG5
ENSP00000338067	24	PKIG
ENSP00000369785	24	PIR
ENSP00000343144	24	PARD6G
ENSP00000364609	24	PADI3
ENSP00000323872	24	P2RY11
ENSP00000255174	24	OSER1
ENSP00000365451	24	OR4D1
ENSP00000408697	24	NUGGC
ENSP00000303575	24	NUDT9
ENSP00000319664	24	NUDC
ENSP00000369323	24	NPNT
ENSP00000370372	24	NLN
ENSP00000221978	24	NKG7
ENSP00000369400	24	NHS
ENSP00000363557	24	MYOM3
ENSP00000312244	24	MSL3
ENSP00000262065	24	MMD
ENSP00000337340	24	MED19
ENSP00000358400	24	MDN1
ENSP00000343212	24	MAP3K7CL
ENSP00000355895	24	LYPLAL1
ENSP00000354977	24	LTN1
ENSP00000156499	24	KLK14
ENSP00000367459	24	KIAA0319
ENSP00000295082	24	KCNF1
ENSP00000358802	24	KCNC4
ENSP00000360806	24	KCNB1
ENSP00000371886	24	JMJD7-PLA2G4B
ENSP00000374778	24	IGKV4-1
ENSP00000354895	24	IFT140
ENSP00000342114	24	ICAM4
ENSP00000390282	24	HLA-C
ENSP00000359049	24	HENMT1
ENSP00000263956	24	GTF3C3
ENSP00000348877	24	GPI
ENSP00000363411	24	GNG10
ENSP00000232564	24	GNB4
ENSP00000313933	24	GLTSCR1L
ENSP00000223145	24	GLCCI1
ENSP00000305107	24	GIMAP8
ENSP00000260270	24	FDX1
ENSP00000357086	24	FCRL6
ENSP00000355157	24	FCRL2

ENSP00000298097	24	FBXO33
ENSP00000296486	24	ETNPPL
ENSP00000308472	24	EIF4EBP3
ENSP00000224073	24	EDF1
ENSP00000315098	24	EDDM3A
ENSP00000225171	24	DNAJC12
ENSP00000262442	24	DNAH9
ENSP00000265104	24	DNAH5
ENSP00000251642	24	DHX58
ENSP00000344674	24	DHRS2
ENSP00000384703	24	DDX11
ENSP00000246554	24	COX6B1
ENSP00000229379	24	COX6A1
ENSP00000253452	24	COX4I1
ENSP00000361616	24	COMTD1
ENSP00000341640	24	COL9A3
ENSP00000358491	24	CMC4
ENSP00000245816	24	CLPP
ENSP00000299663	24	CLEC4E
ENSP00000254035	24	CKMT2
ENSP00000419879	24	CDS2
ENSP00000275517	24	CDCA5
ENSP00000295589	24	CCKAR
ENSP00000227520	24	CCDC86
ENSP00000402278	24	C2
ENSP00000294889	24	C1orf115
ENSP00000344285	24	BSX
ENSP00000334836	24	BMP2K
ENSP00000233997	24	AZU1
ENSP00000234396	24	ATP6V1B1
ENSP00000363832	24	AOX1
ENSP00000314048	24	ANO2
ENSP00000265708	24	ADAM2
ENSP00000256001	24	ACTR3B
ENSP00000258873	24	ACSBG1
ENSP00000312618	24	ACAD9
ENSP00000268231	24	12-Sep
ENSP00000309141	24	3-Mar
ENSP00000246538	24	-
ENSP00000256183	24	-
ENSP00000304656	24	-
ENSP00000317255	24	-
ENSP00000328986	24	-
ENSP00000329250	24	-
ENSP00000387668	24	-
ENSP00000387763	24	-

ENSP00000398476	24	-
ENSP00000402043	24	-
ENSP00000407969	24	-
ENSP00000354686	23	ZNF652
ENSP00000288466	23	ZNF618
ENSP00000282308	23	ZNF256
ENSP00000321049	23	ZNF227
ENSP00000254166	23	ZNF132
ENSP00000251119	23	ZFYVE26
ENSP00000363344	23	ZFP37
ENSP00000349689	23	ZDHHC9
ENSP00000378201	23	ZBTB1
ENSP00000254029	23	WDR44
ENSP00000368801	23	WBP4
ENSP00000333376	23	USP45
ENSP00000265403	23	UGT2B10
ENSP00000269601	23	TXNL4A
ENSP00000350716	23	TUSC1
ENSP00000309036	23	TSPAN17
ENSP00000384312	23	TRIOBP
ENSP00000283351	23	TRAPPC8
ENSP00000294309	23	TPCN2
ENSP00000359067	23	TMEM30A
ENSP00000365567	23	TM9SF2
ENSP00000263346	23	TCF25
ENSP00000247219	23	TBPL2
ENSP00000273037	23	TAMM41
ENSP00000355051	23	TAF13
ENSP00000263846	23	SYT7
ENSP00000376472	23	STT3A
ENSP00000321347	23	STRBP
ENSP00000298630	23	STK32C
ENSP00000404812	23	STIM2
ENSP00000381968	23	SPTLC3
ENSP00000368808	23	SMPX
ENSP00000363001	23	SMPDL3B
ENSP00000260126	23	SLCO5A1
ENSP00000217159	23	SLCO4A1
ENSP00000273063	23	SLC4A3
ENSP00000372700	23	SLC39A7
ENSP00000340450	23	SLC2A14
ENSP00000360671	23	SLC25A5
ENSP00000388658	23	SLC25A12
ENSP00000358515	23	SLC22A15
ENSP00000327569	23	SLC22A10
ENSP00000387694	23	SLC12A5

ENSP00000215793	23	SF3A1
ENSP00000325414	23	SERTAD3
ENSP00000362131	23	RPA4
ENSP00000344193	23	RNASE1
ENSP00000165698	23	REEP1
ENSP00000381206	23	RDH16
ENSP00000310471	23	RBM4B
ENSP00000262633	23	RBM42
ENSP00000358532	23	RBM20
ENSP00000220966	23	PYCRL
ENSP00000358714	23	PRSS35
ENSP00000365494	23	PRICKLE3
ENSP00000258083	23	PRADC1
ENSP00000318914	23	PNMA1
ENSP00000263038	23	PHYH
ENSP00000247665	23	PHPT1
ENSP00000379678	23	PGM5
ENSP00000367345	23	PCDHGA1
ENSP00000349344	23	PCDHA4
ENSP00000280701	23	OXSM
ENSP00000206542	23	OSGEP
ENSP00000364627	23	NXPE4
ENSP00000253247	23	NOL11
ENSP00000168977	23	NMRK2
ENSP00000356355	23	NEK7
ENSP00000273512	23	NCEH1
ENSP00000364289	23	NCBP1
ENSP00000379394	23	NAV2
ENSP00000262370	23	MGRN1
ENSP00000314343	23	MED29
ENSP00000244711	23	MEA1
ENSP00000365113	23	MASTL
ENSP00000381298	23	LONRF1
ENSP00000157600	23	LMCD1
ENSP00000359609	23	LMBRD1
ENSP00000296388	23	LEPRE1
ENSP00000357412	23	LENEP
ENSP00000357778	23	LCE3E
ENSP00000357776	23	LCE3D
ENSP00000334644	23	LCE3C
ENSP00000417116	23	LBX2
ENSP00000357288	23	LAMTOR2
ENSP00000247194	23	L3HYPDH
ENSP00000328270	23	KRTAP4-6
ENSP00000413479	23	KRT3
ENSP00000320917	23	KRI1

ENSP00000324414	23	KLK11
ENSP00000365637	23	KIAA1217
ENSP00000281156	23	KHDRBS2
ENSP00000329471	23	KDELR1
ENSP00000221200	23	KCTD9
ENSP00000301738	23	KCTD5
ENSP00000394390	23	KCTD15
ENSP00000366682	23	IRG1
ENSP00000259212	23	IL36RN
ENSP00000244241	23	IL17C
ENSP00000312778	23	IFT80
ENSP00000194214	23	HSPB11
ENSP00000338457	23	HPS4
ENSP00000330721	23	HDDC3
ENSP00000340736	23	GYG1
ENSP00000262580	23	GSDMD
ENSP00000267549	23	GPR65
ENSP00000375893	23	GPR55
ENSP00000272644	23	GPR17
ENSP00000295448	23	GNPDA2
ENSP00000360021	23	GNG12
ENSP00000362463	23	GLO1
ENSP00000330374	23	GIMAP6
ENSP00000381340	23	GGT5
ENSP00000363582	23	GGNBP1
ENSP00000319531	23	GCSH
ENSP00000351552	23	GATAD2A
ENSP00000348897	23	GABRA2
ENSP00000259455	23	GABBR2
ENSP00000363603	23	FUCA1
ENSP00000368039	23	FRG2
ENSP00000297267	23	FNDC1
ENSP00000244426	23	FBXO9
ENSP00000328187	23	FBXO21
ENSP00000403802	23	FBXO10
ENSP00000324292	23	FBF1
ENSP00000351632	23	FAM3D
ENSP00000264344	23	FAM13A
ENSP00000322229	23	FADS1
ENSP00000391594	23	ERV3-1
ENSP00000350967	23	ELP2
ENSP00000289371	23	EIF5B
ENSP00000254624	23	EFR3A
ENSP00000236051	23	EBNA1BP2
ENSP00000367841	23	DYNLT3
ENSP00000362850	23	DSN1

ENSP00000308312	23	DNAI2
ENSP00000352273	23	DIO3
ENSP00000233084	23	DDX1
ENSP00000372812	23	CYP21A2
ENSP00000394942	23	CYP21A2
ENSP00000160373	23	CTTNBP2
ENSP00000365116	23	CTRC
ENSP00000342056	23	CS
ENSP00000391774	23	CPSF6
ENSP00000262812	23	COPE
ENSP00000366513	23	CLSTN1
ENSP00000328182	23	CLN8
ENSP00000283122	23	CETN3
ENSP00000367229	23	CEP68
ENSP00000299415	23	CCDC182
ENSP00000384490	23	CBX6
ENSP00000064571	23	CBLN4
ENSP00000372169	23	CACNA2D4
ENSP00000338812	23	C1QTNF6
ENSP00000312158	23	BTN2A1
ENSP00000360312	23	BSND
ENSP00000347301	23	BEGAIN
ENSP00000263182	23	BBOX1
ENSP00000352408	23	BABAM1
ENSP00000306459	23	B4GALT6
ENSP00000300404	23	B4GALNT2
ENSP00000265471	23	B3GAT3
ENSP00000290949	23	ATP6V0D1
ENSP00000253856	23	ATP6V0A4
ENSP00000349877	23	ATP13A1
ENSP00000307188	23	ASL
ENSP00000296525	23	ASB5
ENSP00000293414	23	ASB16
ENSP00000250693	23	ART1
ENSP00000288065	23	ARMC12
ENSP00000313422	23	ARL6IP4
ENSP00000368994	23	ARHGAP44
ENSP00000411913	23	AKR7L
ENSP00000357105	23	AKAP7
ENSP00000353804	23	ACSS2
ENSP00000307697	23	ACOX2
ENSP00000301452	23	ACER1
ENSP00000207771	23	-
ENSP00000295943	23	-
ENSP00000308470	23	-
ENSP00000364304	23	-

ENSP00000380754	23	-
ENSP00000380795	23	-
ENSP00000385883	23	-
ENSP00000393902	23	-
ENSP00000395046	23	-
ENSP00000398885	23	-
ENSP00000406898	23	-
ENSP00000409702	23	-
ENSP00000410748	23	-
ENSP00000323879	22	ZNRF2
ENSP00000300870	22	ZNF267
ENSP00000262894	22	ZNF225
ENSP00000262259	22	ZNF175
ENSP00000348593	22	ZFYVE27
ENSP00000339030	22	ZFP91
ENSP00000343557	22	ZCCHC17
ENSP00000374592	22	ZC3H12D
ENSP00000344616	22	ZAR1L
ENSP00000253031	22	YIPF2
ENSP00000287387	22	WDYHV1
ENSP00000254442	22	WDR7
ENSP00000314444	22	WDR35
ENSP00000253794	22	VPS25
ENSP00000306864	22	VASN
ENSP00000407487	22	UNC45A
ENSP00000400409	22	UNC13A
ENSP00000272638	22	UBXN4
ENSP00000217173	22	UBOX5
ENSP00000354201	22	UBE2Z
ENSP00000360116	22	UBE2U
ENSP00000383719	22	UBE2J2
ENSP00000322439	22	TUFM
ENSP00000353971	22	TTI2
ENSP00000300181	22	TSC22D4
ENSP00000155858	22	TRPM5
ENSP00000252136	22	TRMT2A
ENSP00000373979	22	TRAPPC9
ENSP00000379531	22	TMC5
ENSP00000280358	22	TEX12
ENSP00000376328	22	TESC
ENSP00000334308	22	SYNE3
ENSP00000297205	22	STEAP1
ENSP00000291386	22	SSU72
ENSP00000305230	22	SRP9
ENSP00000006658	22	SPATA20
ENSP00000337804	22	SPAG17

ENSP00000357425	22	SMPDL3A
ENSP00000266086	22	SLC5A4
ENSP00000289877	22	SLC45A1
ENSP00000313740	22	SLC38A5
ENSP00000333519	22	SLC24A3
ENSP00000376150	22	SLC16A3
ENSP00000265807	22	SH2D4A
ENSP00000329374	22	SERPINA7
ENSP00000312122	22	SEC13
ENSP00000396915	22	SCN1B
ENSP00000367197	22	SCGN
ENSP00000267176	22	SBNO1
ENSP00000272317	22	RPS27A
ENSP00000360497	22	RNF113A
ENSP00000268122	22	RHCG
ENSP00000255013	22	RHBG
ENSP00000219551	22	RHBDL1
ENSP00000267502	22	RDH12
ENSP00000364639	22	RBM7
ENSP00000261973	22	RBM25
ENSP00000328858	22	PYCR1
ENSP00000420176	22	POLE4
ENSP00000219207	22	PLLP
ENSP00000389913	22	PLEKHM1
ENSP00000290472	22	PLA2G4D
ENSP00000317144	22	PIBF1
ENSP00000345968	22	PGLYRP2
ENSP00000319817	22	OPA3
ENSP00000360841	22	OBP2A
ENSP00000306670	22	NUDT16L1
ENSP00000364163	22	NOTCH4
ENSP00000301295	22	NLRP4
ENSP00000400476	22	NFRKB
ENSP00000333680	22	NFAM1
ENSP00000337998	22	NCKAP5L
ENSP00000324628	22	NAPG
ENSP00000366225	22	NAPB
ENSP00000341679	22	NADK
ENSP00000240922	22	NAA50
ENSP00000301972	22	MYRIP
ENSP00000252172	22	MYH13
ENSP00000354665	22	MT-ND6
ENSP00000402537	22	MTMR10
ENSP00000266263	22	MTFP1
ENSP00000310785	22	MRPS22
ENSP00000318352	22	MRFAP1

ENSP00000356846	22	MAEL
ENSP00000230568	22	LY86
ENSP00000295057	22	LRRTM1
ENSP00000248668	22	LRFN1
ENSP00000254770	22	LANCL2
ENSP00000356230	22	KLHL12
ENSP00000313995	22	KLHDC3
ENSP00000374566	22	KIF19
ENSP00000280020	22	KIAA1328
ENSP00000356189	22	IPCEF1
ENSP00000408526	22	IMPA1
ENSP00000374810	22	IGKV1D-13
ENSP00000246532	22	IGFLR1
ENSP00000233893	22	HSPE1
ENSP00000261374	22	HS3ST2
ENSP00000355228	22	HMGN2
ENSP00000255039	22	HAPLN2
ENSP00000302728	22	GUSB
ENSP00000392828	22	GPSM1
ENSP00000309493	22	GPRC6A
ENSP00000319744	22	GPR4
ENSP00000329266	22	GPIHBP1
ENSP00000319286	22	GP5
ENSP00000234160	22	GORASP2
ENSP00000355607	22	GNPAT
ENSP00000371594	22	GNG7
ENSP00000253054	22	GMFG
ENSP00000330836	22	GLRX3
ENSP00000274576	22	GLRA1
ENSP00000297596	22	GEM
ENSP00000307939	22	GCC2
ENSP00000248924	22	GCAT
ENSP00000273951	22	GC
ENSP00000367343	22	GBA2
ENSP00000349884	22	GALP
ENSP00000412673	22	GABRR1
ENSP00000263773	22	FNBP4
ENSP00000246841	22	FLRT1
ENSP00000400223	22	FJX1
ENSP00000260008	22	FHDC1
ENSP00000356925	22	FCRLB
ENSP00000357167	22	FCRL3
ENSP00000261844	22	FAM214A
ENSP00000418575	22	FAM19A1
ENSP00000033079	22	FAM13B
ENSP00000258416	22	EIF4E2

ENSP00000357278	22	ECHDC1
ENSP00000362041	22	DNAJC9
ENSP00000368565	22	DNAJC2
ENSP00000298292	22	DNAAF2
ENSP00000216500	22	DHRS7
ENSP00000288111	22	DHRS1
ENSP00000306528	22	DESI2
ENSP00000248901	22	CYTH4
ENSP00000248041	22	CYP4F11
ENSP00000334592	22	CYP2R1
ENSP00000264376	22	CRYGD
ENSP00000360541	22	CPT2
ENSP00000222482	22	CPA4
ENSP00000373298	22	COLQ
ENSP00000355629	22	COG2
ENSP00000358894	22	CNNM2
ENSP00000304782	22	CES3
ENSP00000377007	22	CENPN
ENSP00000352639	22	CELA2A
ENSP00000302968	22	CDY1
ENSP00000355237	22	CDC42BPB
ENSP00000338013	22	CCDC183
ENSP00000368931	22	CCDC112
ENSP00000257347	22	CARS2
ENSP00000272520	22	C1QL2
ENSP00000368623	22	C1orf159
ENSP00000216807	22	BRMS1L
ENSP00000262865	22	BPI
ENSP00000367104	22	BFSP1
ENSP00000372210	22	BET1L
ENSP00000263519	22	ATP2B3
ENSP00000318846	22	ASCL3
ENSP00000266503	22	ARNTL2
ENSP00000295087	22	ARL5A
ENSP00000308496	22	ARL10
ENSP00000348769	22	ARIH2
ENSP00000255194	22	AP3B1
ENSP00000397009	22	AKR1B15
ENSP00000347581	22	AGK
ENSP00000260600	22	ADCY3
ENSP00000289228	22	ACTR1B
ENSP00000256997	22	ACP2
ENSP00000342609	22	ACER2
ENSP00000373627	22	14-Sep
ENSP00000196548	22	-
ENSP00000222249	22	-

ENSP00000256972	22	-
ENSP00000278207	22	-
ENSP00000308352	22	-
ENSP00000383007	22	-
ENSP00000405410	22	-
ENSP00000412935	22	-
ENSP00000346729	21	ZNF589
ENSP00000298299	21	ZNF22
ENSP00000263805	21	ZNF106
ENSP00000261749	21	ZFAND6
ENSP00000330485	21	ZDHHC23
ENSP00000261353	21	YPEL5
ENSP00000017003	21	XYLT2
ENSP00000319474	21	WSB2
ENSP00000261015	21	WDR12
ENSP00000404251	21	VGLL4
ENSP00000359668	21	VGLL1
ENSP00000306397	21	UQCRFS1
ENSP00000267938	21	UBE2Q2
ENSP00000333114	21	TSHZ2
ENSP00000257604	21	TRAFD1
ENSP00000393316	21	TMX1
ENSP00000249700	21	TMOD2
ENSP00000005286	21	TMEM132A
ENSP00000261226	21	TMCC3
ENSP00000268138	21	TICRR
ENSP00000375093	21	TAS2R31
ENSP00000362403	21	TACR2
ENSP00000332287	21	SYNGR1
ENSP00000356540	21	STX11
ENSP00000358810	21	STRIP1
ENSP00000264228	21	SRD5A3
ENSP00000298130	21	SPTSSA
ENSP00000397131	21	SPC24
ENSP00000301466	21	SOAT2
ENSP00000369446	21	SMAGP
ENSP00000359521	21	SLITRK2
ENSP00000381634	21	SLC38A1
ENSP00000297195	21	SLC29A4
ENSP00000357915	21	SLC22A16
ENSP00000316909	21	SLC17A8
ENSP00000285116	21	SKA1
ENSP00000362308	21	SH3BGRL
ENSP00000341584	21	SERPINB13
ENSP00000367893	21	SEPHS1
ENSP00000356744	21	SCYL3

ENSP00000343248	21	SBK1
ENSP00000361772	21	SAMD8
ENSP00000377094	21	RUFY1
ENSP00000296142	21	RTP3
ENSP00000252655	21	RSPH3
ENSP00000274242	21	RPL37
ENSP00000293842	21	RPL26
ENSP00000316628	21	RNF152
ENSP00000261947	21	RNF130
ENSP00000292363	21	RNF126
ENSP00000361768	21	RIMS3
ENSP00000216085	21	RHBDD3
ENSP00000296292	21	RFT1
ENSP00000201979	21	REM1
ENSP00000356828	21	RCSD1
ENSP00000325919	21	PSMG2
ENSP00000384770	21	PSG4
ENSP00000332215	21	PSG3
ENSP00000280258	21	PRSS23
ENSP00000361642	21	PPCS
ENSP00000358320	21	POLR3GL
ENSP00000324124	21	POLR2L
ENSP00000223127	21	PLOD3
ENSP00000322373	21	PLEKHF2
ENSP00000371833	21	PLA2G4F
ENSP00000268043	21	PIF1
ENSP00000278243	21	PGAP2
ENSP00000274311	21	PELO
ENSP00000267460	21	PELI2
ENSP00000394382	21	PDHA1
ENSP00000264917	21	PDE8B
ENSP00000264254	21	PDCL3
ENSP00000367373	21	PCDHA1
ENSP00000299299	21	PCBD1
ENSP00000282391	21	PAN3
ENSP00000343339	21	P2RX2
ENSP00000222673	21	OGDH
ENSP00000318029	21	ODF3L2
ENSP00000325868	21	ODF3
ENSP00000285402	21	ODF1
ENSP00000219169	21	NUTF2
ENSP00000305503	21	NUP62
ENSP00000231498	21	NUP155
ENSP00000419628	21	NUDT5
ENSP00000283027	21	NUBP1
ENSP00000307822	21	NPFFR2

ENSP00000268802	21	NOB1
ENSP00000414415	21	NLRC3
ENSP00000407978	21	NKX1-1
ENSP00000326806	21	NCBP2
ENSP00000256854	21	NARS
ENSP00000267068	21	N4BP2L2
ENSP00000264198	21	MUL1
ENSP00000302716	21	MUC17
ENSP00000354580	21	MRPL21
ENSP00000354223	21	MPC1
ENSP00000233616	21	MOGS
ENSP00000312870	21	MCM9
ENSP00000281806	21	MCHR2
ENSP00000314196	21	MBOAT4
ENSP00000267287	21	MBNL2
ENSP00000368972	21	MAP7D2
ENSP00000334696	21	KRTAP19-7
ENSP00000259711	21	KIF13A
ENSP00000371514	21	KCNV2
ENSP00000359425	21	KCNQ5
ENSP00000304127	21	KCNG3
ENSP00000238647	21	IRF2BPL
ENSP00000302724	21	INSL5
ENSP00000374856	21	IGLC2
ENSP00000349181	21	HVCN1
ENSP00000322924	21	HTR1F
ENSP00000303394	21	HSPB3
ENSP00000373091	21	HLA-E
ENSP00000372713	21	HLA-DOA
ENSP00000307342	21	HCN1
ENSP00000319799	21	H1FOO
ENSP00000296518	21	GUCY1A3
ENSP00000394954	21	GSG1L
ENSP00000365624	21	GRIPAP1
ENSP00000300571	21	GPRC5B
ENSP00000327589	21	GLUD2
ENSP00000338523	21	FNDC3B
ENSP00000251547	21	FBXO44
ENSP00000236980	21	FASTKD2
ENSP00000364731	21	F7
ENSP00000342144	21	EVC2
ENSP00000356170	21	ETNK2
ENSP00000300682	21	ENGASE
ENSP00000265753	21	EIF4H
ENSP00000336727	21	EDRF1
ENSP00000309690	21	DMXL1

ENSP00000392601	21	DHX30
ENSP00000264057	21	DGKD
ENSP00000412292	21	DEPDC1
ENSP00000336972	21	DEDD2
ENSP00000310723	21	DDX23
ENSP00000346120	21	DDX21
ENSP00000258415	21	CYP27A1
ENSP00000361699	21	CTPS1
ENSP00000315791	21	CSTF3
ENSP00000291568	21	CSTB
ENSP00000292476	21	CPSF4
ENSP00000359603	21	COL24A1
ENSP00000375069	21	COL23A1
ENSP00000316030	21	COL19A1
ENSP00000216416	21	CNIH1
ENSP00000366673	21	CLN5
ENSP00000348074	21	CLEC9A
ENSP00000326407	21	CLEC1A
ENSP00000344563	21	CLEC12B
ENSP00000398105	21	CHAC1
ENSP00000264982	21	CEP70
ENSP00000256343	21	CATSPERB
ENSP00000269881	21	CALR3
ENSP00000317121	21	CACFD1
ENSP00000366662	21	CA6
ENSP00000358107	21	CA14
ENSP00000357507	21	C1orf43
ENSP00000029410	21	B4GALT7
ENSP00000364349	21	ATF6B
ENSP00000356992	21	ARHGAP30
ENSP00000335147	21	ANKRD37
ENSP00000301190	21	ANKRD33
ENSP00000348635	21	AMICA1
ENSP00000315417	21	ALX1
ENSP00000313670	21	ALS2CL
ENSP00000374219	21	ALKBH8
ENSP00000359224	21	ALG14
ENSP00000290597	21	ALDH4A1
ENSP00000386284	21	ALAD
ENSP00000324827	21	AKAP17A
ENSP00000298545	21	AK8
ENSP00000294724	21	AGL
ENSP00000307634	21	AGAP1
ENSP00000226355	21	AFM
ENSP00000301956	21	ACSM1
ENSP00000242592	21	ACADS

ENSP00000304111	21	ABCG4
ENSP00000284425	21	ABCA6
ENSP00000278618	21	AASDHPPT
ENSP00000190839	21	-
ENSP00000216520	21	-
ENSP00000232907	21	-
ENSP00000238522	21	-
ENSP00000240343	21	-
ENSP00000263498	21	-
ENSP00000302965	21	-
ENSP00000309032	21	-
ENSP00000314768	21	-
ENSP00000347299	21	-
ENSP00000360678	21	-
ENSP00000363036	21	-
ENSP00000381897	21	-
ENSP00000385877	21	-
ENSP00000389690	21	-
ENSP00000393180	21	-
ENSP00000409569	21	-
ENSP00000410971	21	-
ENSP00000411517	21	-
ENSP00000416995	21	-
ENSP00000320627	20	ZNF7
ENSP00000320050	20	ZNF580
ENSP00000306351	20	ZNF182
ENSP00000340749	20	ZNF124
ENSP00000310543	20	ZFYVE21
ENSP00000416707	20	XKR6
ENSP00000261708	20	UTP6
ENSP00000268876	20	UNC45B
ENSP00000251566	20	UGT2A3
ENSP00000252108	20	UBE4A
ENSP00000351596	20	TTC37
ENSP00000246801	20	TSKS
ENSP00000333934	20	TRMT11
ENSP00000356134	20	TFB1M
ENSP00000207457	20	TEKT2
ENSP00000343515	20	TCEA2
ENSP00000369736	20	TBCA
ENSP00000248121	20	SYNGR3
ENSP00000332818	20	SV2B
ENSP00000266971	20	SUOX
ENSP00000318445	20	ST3GAL1
ENSP00000317331	20	SSR4
ENSP00000388731	20	SSFA2

ENSP00000391069	20	SRPK1
ENSP00000301717	20	SPSB3
ENSP00000299272	20	SPIC
ENSP00000326841	20	SPATS2
ENSP00000360913	20	SPATA6
ENSP00000380336	20	SMU1
ENSP00000337125	20	SMEK1
ENSP00000299798	20	SLC9A5
ENSP00000023064	20	SLC7A9
ENSP00000253188	20	SLC7A10
ENSP00000395797	20	SLC4A10
ENSP00000387152	20	SLC35E1
ENSP00000355353	20	SLC34A3
ENSP00000363329	20	SLC31A1
ENSP00000315006	20	SLC28A2
ENSP00000417085	20	SLC15A2
ENSP00000275730	20	SLC12A9
ENSP00000273861	20	SLC10A4
ENSP00000231721	20	SEMA3G
ENSP00000386796	20	SCN7A
ENSP00000283254	20	SCN3A
ENSP00000381824	20	SAC3D1
ENSP00000245923	20	RTN2
ENSP00000361236	20	RSPH9
ENSP00000347271	20	RPS10
ENSP00000370606	20	RPAIN
ENSP00000297157	20	RP9
ENSP00000273480	20	RNF7
ENSP00000334958	20	RLTPR
ENSP00000293677	20	RAVER1
ENSP00000270077	20	PSG9
ENSP00000302114	20	PRELID1
ENSP00000416126	20	PRAP1
ENSP00000379093	20	PPP6R2
ENSP00000390427	20	PPIL2
ENSP00000306614	20	PPIH
ENSP00000371272	20	PLIN5
ENSP00000026218	20	PIGQ
ENSP00000363003	20	PHF19
ENSP00000363734	20	PFDN6
ENSP00000300056	20	PEX11A
ENSP00000322532	20	PELI3
ENSP00000284770	20	PDLIM3
ENSP00000307241	20	PDHB
ENSP00000233630	20	PCGF1
ENSP00000252085	20	PCDHGA12

ENSP00000332915	20	PARPBP
ENSP00000357838	20	OAT
ENSP00000225696	20	NUP88
ENSP00000405674	20	NOL7
ENSP00000408957	20	NEU1
ENSP00000322450	20	NDUFV1
ENSP00000330694	20	NDNL2
ENSP00000309899	20	NARF
ENSP00000362170	20	NAIF1
ENSP00000354961	20	MT-ND4
ENSP00000394649	20	MSH5
ENSP00000406868	20	MSH5
ENSP00000407047	20	MSH5
ENSP00000306697	20	MRAP
ENSP00000362282	20	MOCS1
ENSP00000288709	20	MMEL1
ENSP00000281317	20	MMAA
ENSP00000297508	20	MICALL2
ENSP00000281928	20	MED13L
ENSP00000263702	20	MECR
ENSP00000379638	20	MANSC1
ENSP00000215739	20	LZTR1
ENSP00000399441	20	LRRC70
ENSP00000302621	20	LRG1
ENSP00000291759	20	LILRA4
ENSP00000313571	20	LGALS7B
ENSP00000221797	20	LGALS13
ENSP00000301046	20	LALBA
ENSP00000342710	20	KRT77
ENSP00000341342	20	KLHL6
ENSP00000262820	20	KLHL13
ENSP00000385000	20	KIF2A
ENSP00000247986	20	KIF17
ENSP00000380982	20	KIAA0020
ENSP00000351068	20	KCNMB2
ENSP00000341479	20	KCNJ14
ENSP00000302586	20	KBTBD2
ENSP00000335106	20	KATNA1
ENSP00000347244	20	ITSN2
ENSP00000261574	20	IPO5
ENSP00000358865	20	INA
ENSP00000333639	20	IFNL2
ENSP00000384886	20	IFI30
ENSP00000303279	20	IARS2
ENSP00000246896	20	HTN1
ENSP00000296051	20	HPS3

ENSP00000400842	20	HLA-B
ENSP00000251287	20	HCN2
ENSP00000281543	20	GUF1
ENSP00000361180	20	GTF3C5
ENSP00000262895	20	GRIK5
ENSP00000340595	20	GPRC5C
ENSP00000300873	20	GNG8
ENSP00000264428	20	GLRB
ENSP00000315070	20	GJD4
ENSP00000350415	20	GJA9
ENSP00000358358	20	GJA10
ENSP00000292180	20	FLAD1
ENSP00000326570	20	FKRP
ENSP00000344855	20	FBXL14
ENSP00000353741	20	ETF1
ENSP00000202816	20	ESF1
ENSP00000248342	20	EIF3K
ENSP00000254730	20	EEFSEC
ENSP00000346160	20	DUSP19
ENSP00000366672	20	DTD1
ENSP00000361667	20	DOLK
ENSP00000261383	20	DNAH3
ENSP00000251241	20	DHX40
ENSP00000417078	20	DHX36
ENSP00000280557	20	DENR
ENSP00000297435	20	DEFA4
ENSP00000288071	20	DDX19B
ENSP00000215773	20	DDT
ENSP00000286648	20	DCK
ENSP00000360968	20	CYP4X1
ENSP00000359507	20	CUTC
ENSP00000217423	20	CST4
ENSP00000307540	20	CST2
ENSP00000227348	20	CRTAM
ENSP00000315203	20	CREG2
ENSP00000339353	20	CPSF1
ENSP00000309762	20	COL6A5
ENSP00000381022	20	COL26A1
ENSP00000365538	20	CLYBL
ENSP00000263309	20	CLNS1A
ENSP00000290332	20	CLIC6
ENSP00000291495	20	CILP2
ENSP00000250699	20	CHRNA10
ENSP00000324205	20	CHMP4A
ENSP00000198939	20	CHERP
ENSP00000381397	20	CES4A

ENSP00000341159	20	CERKL
ENSP00000353259	20	CD300A
ENSP00000367605	20	CCL3L3
ENSP00000363773	20	C1QA
ENSP00000289361	20	BTN3A1
ENSP00000369739	20	BPHL
ENSP00000230053	20	B3GAT2
ENSP00000284727	20	ATP5G3
ENSP00000357060	20	ATP1A4
ENSP00000384259	20	ATG4B
ENSP00000275699	20	ASB15
ENSP00000263245	20	ARFGAP3
ENSP00000261558	20	AP5M1
ENSP00000342295	20	ANKRD55
ENSP00000274181	20	ADAMTS16
ENSP00000351717	20	ABCB8
ENSP00000273067	20	4-Mar
ENSP00000238576	20	-
ENSP00000290575	20	-
ENSP00000293569	20	-
ENSP00000328729	20	-
ENSP00000329426	20	-
ENSP00000330211	20	-
ENSP00000332877	20	-
ENSP00000344402	20	-
ENSP00000353243	20	-
ENSP00000362934	20	-
ENSP00000367255	20	-
ENSP00000368809	20	-
ENSP00000369775	20	-
ENSP00000375675	20	-
ENSP00000388356	20	-
ENSP00000398601	20	-
ENSP00000409207	20	-
ENSP00000413759	20	-
ENSP00000416357	20	-
ENSP00000359837	19	ZZZ3
ENSP00000382513	19	ZNF594
ENSP00000301310	19	ZNF582
ENSP00000321132	19	ZNF554
ENSP00000359740	19	ZNF451
ENSP00000378165	19	ZNF207
ENSP00000296490	19	WDR82
ENSP00000308179	19	WDR3
ENSP00000326534	19	VPS39
ENSP00000309262	19	USP48

ENSP00000362513	19	UGT1A5
ENSP00000379500	19	UEVLD
ENSP00000292211	19	UBE2Q1
ENSP00000039989	19	TTC17
ENSP00000284995	19	TSEN2
ENSP00000251607	19	TRNT1
ENSP00000282369	19	TRIM36
ENSP00000362219	19	TREML1
ENSP00000351896	19	TRAPPC4
ENSP00000284885	19	TMPRSS15
ENSP00000359305	19	TMED5
ENSP00000260867	19	TIMM23
ENSP00000386186	19	TIGD1
ENSP00000357231	19	THEMIS
ENSP00000215742	19	THAP7
ENSP00000355338	19	TBCK
ENSP00000329256	19	TBC1D3F
ENSP00000318262	19	TBC1D15
ENSP00000340271	19	TAF3
ENSP00000386439	19	SYNC
ENSP00000342554	19	STX2
ENSP00000215095	19	STX1B
ENSP00000348886	19	STOML2
ENSP00000313752	19	SSNA1
ENSP00000347646	19	SOX12
ENSP00000363349	19	SNX30
ENSP00000372390	19	SLC7A4
ENSP00000350475	19	SLC4A5
ENSP00000264451	19	SLC30A9
ENSP00000262462	19	SLC27A6
ENSP00000262352	19	SLC1A1
ENSP00000338358	19	SERPINB6
ENSP00000283752	19	SERPINB3
ENSP00000342850	19	SERPINA6
ENSP00000361855	19	SEMG2
ENSP00000280551	19	SEC24D
ENSP00000341117	19	SDSL
ENSP00000255390	19	SCO1
ENSP00000321594	19	SCNN1D
ENSP00000369292	19	SAMD9
ENSP00000371729	19	SACS
ENSP00000362744	19	RPS4X
ENSP00000318646	19	RPS15A
ENSP00000389103	19	RPL23A
ENSP00000269439	19	RNF165
ENSP00000316543	19	RAPH1

ENSP00000386385	19	PXMP4
ENSP00000290294	19	PRAC1
ENSP00000387046	19	PPM1M
ENSP00000313070	19	PPIP5K2
ENSP00000380129	19	PPIP5K1
ENSP00000330190	19	POU6F1
ENSP00000264231	19	POPDC2
ENSP00000228347	19	POLR3B
ENSP00000301286	19	PLIN4
ENSP00000347933	19	PLGLB1
ENSP00000263265	19	PLEKHA4
ENSP00000234453	19	PLEKHA3
ENSP00000289672	19	PKD1L1
ENSP00000348772	19	PITPNM1
ENSP00000218432	19	PIN4
ENSP00000415203	19	PIGG
ENSP00000362987	19	PHYHIPL
ENSP00000417038	19	PHACTR2
ENSP00000288774	19	PEX10
ENSP00000353868	19	PCED1A
ENSP00000367363	19	PCDHA8
ENSP00000289272	19	PCDHA13
ENSP00000308466	19	PBLD
ENSP00000328992	19	PAXBP1
ENSP00000278360	19	PAMR1
ENSP00000364620	19	PADI1
ENSP00000263314	19	P2RX3
ENSP00000371786	19	ONECUT3
ENSP00000254998	19	NXT1
ENSP00000285968	19	NUP205
ENSP00000357402	19	NKAIN2
ENSP00000355955	19	NENF
ENSP00000360483	19	NDC1
ENSP00000293404	19	NAGS
ENSP00000352272	19	MYOZ1
ENSP00000383023	19	MYL5
ENSP00000394619	19	MSH5
ENSP00000290130	19	MIS18A
ENSP00000265052	19	MGLL
ENSP00000381928	19	MGARP
ENSP00000293777	19	MED11
ENSP00000395473	19	MAN2B1
ENSP00000274711	19	LRRTM2
ENSP00000348043	19	LRRC20
ENSP00000325929	19	LNX2
ENSP00000261292	19	LIPG

ENSP00000310126	19	LINGO2
ENSP00000329932	19	LGALS9C
ENSP00000388841	19	LGALS9B
ENSP00000371595	19	KRTAP5-6
ENSP00000371328	19	KLRC3
ENSP00000327468	19	KLHDC8B
ENSP00000361952	19	KCNK15
ENSP00000325448	19	KARS
ENSP00000415436	19	KANSL2
ENSP00000271843	19	JTB
ENSP00000295321	19	IWS1
ENSP00000369456	19	ITPA
ENSP00000323424	19	ISG20L2
ENSP00000290552	19	IRX6
ENSP00000256079	19	IPO8
ENSP00000387262	19	IMMT
ENSP00000217901	19	IDH3G
ENSP00000263043	19	ICK
ENSP00000335511	19	HTR3E
ENSP00000405409	19	HTR3D
ENSP00000322617	19	HTR3C
ENSP00000265866	19	HNRNPH3
ENSP00000295470	19	HNRNPDL
ENSP00000414196	19	HLA-DPB1
ENSP00000302276	19	HEXIM2
ENSP00000299192	19	HEATR3
ENSP00000357342	19	HCN3
ENSP00000323811	19	HACL1
ENSP00000312584	19	GSDMB
ENSP00000313432	19	GRHPR
ENSP00000284311	19	GPR15
ENSP00000347206	19	GPAA1
ENSP00000403576	19	GNL1
ENSP00000300406	19	GNGT2
ENSP00000266068	19	GMEB2
ENSP00000328570	19	GLRX5
ENSP00000296882	19	GJB7
ENSP00000381970	19	GIN1
ENSP00000265012	19	GCNT2
ENSP00000300648	19	GCN1L1
ENSP00000318821	19	GCC1
ENSP00000386029	19	GABRR2
ENSP00000302599	19	FUT9
ENSP00000286955	19	FUT6
ENSP00000252771	19	FCHO1
ENSP00000355827	19	FBXO28

ENSP00000241071	19	FBXO24
ENSP00000307833	19	FBXO22
ENSP00000287468	19	FBRS
ENSP00000270509	19	FBN3
ENSP00000221801	19	FBL
ENSP00000279259	19	FAU
ENSP00000386895	19	FAM124B
ENSP00000417970	19	FAM120B
ENSP00000246100	19	FAM110A
ENSP00000323046	19	EXOSC3
ENSP00000351520	19	ENTPD4
ENSP00000269466	19	ELAC1
ENSP00000337675	19	EBAG9
ENSP00000269445	19	DYM
ENSP00000296161	19	DTX3L
ENSP00000242776	19	DDX39A
ENSP00000260803	19	DBR1
ENSP00000321821	19	CYP4F12
ENSP00000359976	19	CTH
ENSP00000298875	19	CPSF2
ENSP00000296046	19	CPA3
ENSP00000258424	19	COX5B
ENSP00000332723	19	COLEC10
ENSP00000288040	19	CLEC18A
ENSP00000299565	19	CHRNA5
ENSP00000300113	19	CHP2
ENSP00000319255	19	CHORDC1
ENSP00000324491	19	CHMP7
ENSP00000256785	19	CFHR5
ENSP00000319052	19	CETN1
ENSP00000264993	19	CDV3
ENSP00000265334	19	CDKL3
ENSP00000326432	19	CCR8
ENSP00000332716	19	CCDC28A
ENSP00000379310	19	CASC1
ENSP00000222125	19	CAPS
ENSP00000419081	19	CALML4
ENSP00000324960	19	CABP4
ENSP00000332809	19	C2orf71
ENSP00000344566	19	C21orf2
ENSP00000286031	19	C1orf112
ENSP00000316898	19	C12orf54
ENSP00000369965	19	BTF3
ENSP00000322991	19	BCAT2
ENSP00000348799	19	ATF7IP2
ENSP00000265138	19	ARRDC3

ENSP00000355026	19	ARHGEF15
ENSP00000156471	19	AQR
ENSP00000216366	19	AP4S1
ENSP00000360149	19	ALG6
ENSP00000387123	19	ALDH7A1
ENSP00000228850	19	AKAP3
ENSP00000370521	19	AIPL1
ENSP00000362273	19	ADPRHL2
ENSP00000286621	19	ADK
ENSP00000294016	19	ADCY9
ENSP00000297323	19	ADCY1
ENSP00000256412	19	ADAMDEC1
ENSP00000255082	19	ACY3
ENSP00000356015	19	ACAT2
ENSP00000389813	19	ACAD10
ENSP00000377040	19	AASS
ENSP00000386456	19	AAK1
ENSP00000236900	19	-
ENSP00000258531	19	-
ENSP00000330937	19	-
ENSP00000339175	19	-
ENSP00000339580	19	-
ENSP00000342405	19	-
ENSP00000343488	19	-
ENSP00000358559	19	-
ENSP00000379736	19	-
ENSP00000380991	19	-
ENSP00000405037	19	-
ENSP00000409630	19	-
ENSP00000378326	18	ZP3
ENSP00000328614	18	ZNRF3
ENSP00000243639	18	ZNF468
ENSP00000381304	18	ZFP90
ENSP00000358413	18	ZDHHC6
ENSP00000364677	18	ZBTB12
ENSP00000398106	18	ZBED5
ENSP00000263795	18	WDR76
ENSP00000339449	18	WDR16
ENSP00000322276	18	VNN2
ENSP00000344144	18	VCX
ENSP00000308332	18	UTP23
ENSP00000369681	18	USP3
ENSP00000246337	18	UROD
ENSP00000257632	18	UPK3B
ENSP00000301831	18	ULK4
ENSP00000338703	18	UGP2

ENSP00000301281	18	UBXN6
ENSP00000287913	18	TSSK4
ENSP00000362261	18	TRAPPC3
ENSP00000367595	18	TPRG1L
ENSP00000317595	18	TPPP2
ENSP00000324775	18	TMIE
ENSP00000369699	18	TMEM27
ENSP00000300128	18	TMEM194A
ENSP00000259751	18	TJAP1
ENSP00000274532	18	TIMD4
ENSP00000385276	18	TENM3
ENSP00000221855	18	TBCB
ENSP00000346265	18	SYT5
ENSP00000283141	18	SYCP2L
ENSP00000339221	18	SULT1A4
ENSP00000355156	18	SUCNR1
ENSP00000295641	18	STK11IP
ENSP00000366156	18	SRM
ENSP00000365019	18	SPIN1
ENSP00000345964	18	SORCS1
ENSP00000339769	18	SMEK2
ENSP00000383143	18	SLITRK6
ENSP00000330320	18	SLC9A7
ENSP00000330199	18	SLC6A17
ENSP00000243896	18	SLC35C2
ENSP00000348019	18	SLC17A5
ENSP00000266771	18	SLC15A4
ENSP00000225519	18	SHPK
ENSP00000383212	18	SGSM1
ENSP00000350789	18	SFMBT1
ENSP00000345044	18	SBK2
ENSP00000373420	18	RUFY2
ENSP00000350976	18	RTP2
ENSP00000270625	18	RPS11
ENSP00000375730	18	RPL13A
ENSP00000413254	18	RPH3A
ENSP00000005386	18	RPAP3
ENSP00000374552	18	RNF216
ENSP00000303276	18	RNASE2
ENSP00000342529	18	RIBC2
ENSP00000258302	18	RGS8
ENSP00000392936	18	RFPL4A
ENSP00000295802	18	RETSAT
ENSP00000362803	18	PPIL1
ENSP00000329697	18	PPAP2C
ENSP00000296223	18	POLR2H

ENSP00000262753	18	POF1B
ENSP00000295030	18	PEX13
ENSP00000222968	18	PDAP1
ENSP00000268896	18	PCTP
ENSP00000262890	18	PAFAH1B3
ENSP00000367930	18	OXER1
ENSP00000326869	18	ORMDL1
ENSP00000355512	18	OPN3
ENSP00000367721	18	NUP160
ENSP00000358783	18	NPBWR2
ENSP00000267422	18	NOVA1
ENSP00000373810	18	NOP16
ENSP00000262510	18	NLRC5
ENSP00000402231	18	NKX2-6
ENSP00000386394	18	NIF3L1
ENSP00000341737	18	NECAP1
ENSP00000315774	18	NDUFS8
ENSP00000259037	18	NDUFB5
ENSP00000369014	18	NDNF
ENSP00000415090	18	MYO7B
ENSP00000347324	18	MSRB3
ENSP00000278865	18	MS4A3
ENSP00000258169	18	MPHOSPH6
ENSP00000295065	18	MEMO1
ENSP00000310593	18	MAP9
ENSP00000290536	18	M1AP
ENSP00000407535	18	LY6G6F
ENSP00000240304	18	LUC7L3
ENSP00000379651	18	LILRA6
ENSP00000357736	18	LELP1
ENSP00000226299	18	LAP3
ENSP00000366886	18	KLHL21
ENSP00000375810	18	KLC3
ENSP00000382815	18	KIAA2026
ENSP00000334181	18	KDM4D
ENSP00000408405	18	KCTD1
ENSP00000262916	18	KCNQ4
ENSP00000358786	18	KCNA10
ENSP00000261247	18	JKAMP
ENSP00000363360	18	INIP
ENSP00000342520	18	IL17REL
ENSP00000359787	18	IFI44L
ENSP00000413950	18	HSD17B8
ENSP00000363976	18	HLA-DMA
ENSP00000368066	18	HAO1
ENSP00000357863	18	GTF3C6

ENSP00000401596	18	GPBP1
ENSP00000344260	18	GALNT15
ENSP00000269195	18	GALNT1
ENSP00000295452	18	GABRG1
ENSP00000357914	18	GABPB2
ENSP00000370057	18	FRMPD4
ENSP00000238667	18	FLVCR2
ENSP00000344572	18	FIBP
ENSP00000332777	18	FEZF1
ENSP00000281828	18	FARSB
ENSP00000352955	18	FAM3A
ENSP00000284486	18	FAM167A
ENSP00000252338	18	FAM155B
ENSP00000219368	18	FA2H
ENSP00000260585	18	EPT1
ENSP00000359520	18	ENTPD7
ENSP00000311545	18	EMR1
ENSP00000245925	18	EML2
ENSP00000220853	18	EMC2
ENSP00000358831	18	ELOVL4
ENSP00000351769	18	DUS2
ENSP00000319170	18	DHFRL1
ENSP00000328359	18	DEFA3
ENSP00000350401	18	DDHD1
ENSP00000254337	18	DCAF15
ENSP00000311117	18	DBI
ENSP00000364691	18	CROCC
ENSP00000238112	18	CPSF3
ENSP00000315775	18	COG4
ENSP00000354673	18	CNOT4
ENSP00000310003	18	CNIH2
ENSP00000268595	18	CMTM2
ENSP00000221804	18	CLC
ENSP00000264935	18	CEP72
ENSP00000269503	18	CBLN2
ENSP00000366221	18	CASZ1
ENSP00000386959	18	CAPS2
ENSP00000373215	18	CADPS
ENSP00000285273	18	CA10
ENSP00000223122	18	C1GALT1
ENSP00000230340	18	BYSL
ENSP00000362372	18	BRWD3
ENSP00000318128	18	BLOC1S4
ENSP00000222547	18	BET1
ENSP00000321874	18	B3GNT3
ENSP00000334216	18	ATP4B

ENSP00000347408	18	AP3M1
ENSP00000361047	18	ALG13
ENSP00000216479	18	AHSA1
ENSP00000274487	18	ADAMTS19
ENSP00000229243	18	ACRBP
ENSP00000216327	18	ABHD4
ENSP00000296577	18	ABCE1
ENSP00000280560	18	ABCB9
ENSP00000272895	18	ABCA12
ENSP00000261772	18	AARS
ENSP00000248450	18	AAMP
ENSP00000324842	18	AACS
ENSP00000380824	18	10-Sep
ENSP00000244799	18	-
ENSP00000263717	18	-
ENSP00000322376	18	-
ENSP00000331108	18	-
ENSP00000331291	18	-
ENSP00000358575	18	-
ENSP00000370191	18	-
ENSP00000375415	18	-
ENSP00000376937	18	-
ENSP00000382134	18	-
ENSP00000384595	18	-
ENSP00000391638	18	-
ENSP00000408830	18	-
ENSP00000413719	18	-
ENSP00000414221	18	-
ENSP00000354518	17	ZNF830
ENSP00000393835	17	ZNF506
ENSP00000266529	17	ZCRB1
ENSP00000404853	17	XPO7
ENSP00000367879	17	XK
ENSP00000330381	17	WDR5B
ENSP00000382717	17	WDR19
ENSP00000340526	17	UTS2B
ENSP00000274030	17	USP53
ENSP00000387230	17	UPP2
ENSP00000276201	17	UPF3B
ENSP00000384850	17	UNKL
ENSP00000367952	17	UCMA
ENSP00000357292	17	UBQLN4
ENSP00000338533	17	TTC33
ENSP00000375081	17	TSSK1B
ENSP00000262209	17	TRPA1
ENSP00000321810	17	TRIT1

ENSP00000332288	17	TRIM61
ENSP00000256255	17	TMEM66
ENSP00000376553	17	TMEM119
ENSP00000214869	17	TMED1
ENSP00000411099	17	TLK1
ENSP00000343995	17	TEKT3
ENSP00000357812	17	TDRKH
ENSP00000308708	17	TCP11
ENSP00000395574	17	TCEB3
ENSP00000354777	17	TBKBP1
ENSP00000263635	17	TANC1
ENSP00000356848	17	TADA1
ENSP00000005279	17	SYNRG
ENSP00000361042	17	SURF1
ENSP00000369045	17	SPINK4
ENSP00000309189	17	SPERT
ENSP00000237201	17	SPACA1
ENSP00000360272	17	SORBS1
ENSP00000406485	17	SMTNL1
ENSP00000295269	17	SLC9A4
ENSP00000305302	17	SLC6A19
ENSP00000354689	17	SLC39A13
ENSP00000350278	17	SLC30A7
ENSP00000228318	17	SLC25A3
ENSP00000230640	17	SKIV2L2
ENSP00000315182	17	SHQ1
ENSP00000346892	17	SERF1A
ENSP00000257549	17	SDS
ENSP00000331019	17	RPS27L
ENSP00000341885	17	RPS2
ENSP00000322419	17	RPLP2
ENSP00000296277	17	RPL39L
ENSP00000278833	17	ROM1
ENSP00000256257	17	RNF122
ENSP00000352427	17	RGR
ENSP00000304139	17	RBMXL2
ENSP00000368086	17	RAB3IP
ENSP00000359042	17	PRPF38B
ENSP00000398342	17	PPP1R14D
ENSP00000370222	17	POMP
ENSP00000217446	17	PIGU
ENSP00000360125	17	PGM1
ENSP00000290722	17	PGLYRP3
ENSP00000334188	17	PFDN5
ENSP00000360473	17	PFDN4
ENSP00000358312	17	PEX11B

ENSP00000298919	17	PDZRN4
ENSP00000368730	17	PDE7A
ENSP00000387654	17	PCYOX1
ENSP00000289269	17	PCDHAC2
ENSP00000313377	17	PANK2
ENSP00000320291	17	OSBPL1A
ENSP00000381960	17	OOSP1
ENSP00000216879	17	NSFL1C
ENSP00000332766	17	NPB
ENSP00000287713	17	NMNAT2
ENSP00000337618	17	NIPA2
ENSP00000344672	17	NHSL1
ENSP00000280700	17	NGLY1
ENSP00000298310	17	NEMF
ENSP00000372335	17	NELFA
ENSP00000264363	17	NDST4
ENSP00000285039	17	MYO5B
ENSP00000220058	17	MTFMT
ENSP00000354982	17	MT-CO3
ENSP00000355265	17	MT-ATP8
ENSP00000380318	17	MPST
ENSP00000364946	17	MKX
ENSP00000268711	17	MED9
ENSP00000288087	17	MDP1
ENSP00000249016	17	MCHR1
ENSP00000286307	17	LSM11
ENSP00000250173	17	LRRC6
ENSP00000360597	17	LRRC26
ENSP00000298288	17	LRR1
ENSP00000246529	17	LRFN3
ENSP00000345985	17	LRFN2
ENSP00000261596	17	LPIN2
ENSP00000318423	17	LINS
ENSP00000357810	17	LINGO4
ENSP00000270452	17	LILRB4
ENSP00000333952	17	LCE5A
ENSP00000357773	17	LCE2D
ENSP00000316737	17	LCE1D
ENSP00000265537	17	LARS2
ENSP00000329165	17	KRT36
ENSP00000377558	17	KRT35
ENSP00000357762	17	KPRP
ENSP00000331682	17	KLHL22
ENSP00000378033	17	KCNK4
ENSP00000233826	17	KCNJ13
ENSP00000315654	17	KCNG2

ENSP00000280684	17	KCNA6
ENSP00000259205	17	IL36G
ENSP00000344976	17	IL1RAPL2
ENSP00000407353	17	IFLTD1
ENSP00000299518	17	IDH3A
ENSP00000218364	17	HTATSF1
ENSP00000365370	17	HNRNPCL1
ENSP00000337854	17	HEXDC
ENSP00000257626	17	GSAP
ENSP00000271883	17	GON4L
ENSP00000282570	17	GMCL1
ENSP00000361700	17	GLRA4
ENSP00000344659	17	GLB1L2
ENSP00000419038	17	GFM1
ENSP00000226798	17	FRG1
ENSP00000330098	17	FBXL6
ENSP00000247977	17	FBXL12
ENSP00000359375	17	FATE1
ENSP00000377225	17	FAM50A
ENSP00000355045	17	FAM179B
ENSP00000298492	17	FAM175B
ENSP00000257013	17	FAM127A
ENSP00000358518	17	F8A2
ENSP00000326514	17	EXOC1
ENSP00000303552	17	ETFDH
ENSP00000331044	17	ENKUR
ENSP00000253673	17	EMR3
ENSP00000254528	17	EMILIN2
ENSP00000355028	17	DZIP3
ENSP00000378635	17	DRP2
ENSP00000353701	17	DPP3
ENSP00000366179	17	DNAJC1
ENSP00000249356	17	DNAJB9
ENSP00000297436	17	DEFA6
ENSP00000329890	17	DEFA5
ENSP00000387426	17	CYHR1
ENSP00000271277	17	CTTNBP2NL
ENSP00000321732	17	CTDNEP1
ENSP00000297405	17	CSMD3
ENSP00000241312	17	CSMD2
ENSP00000413493	17	CPSF3L
ENSP00000347549	17	CPA5
ENSP00000216361	17	COCH
ENSP00000298912	17	CLMN
ENSP00000243776	17	CHPF
ENSP00000337289	17	CENPQ

ENSP00000260967	17	CDK15
ENSP00000381795	17	CCDC33
ENSP00000309649	17	CA5A
ENSP00000298546	17	C9orf9
ENSP00000354458	17	C8A
ENSP00000406598	17	C4orf27
ENSP00000243611	17	C4BPB
ENSP00000342512	17	C3orf33
ENSP00000352447	17	C1orf116
ENSP00000269221	17	C18orf8
ENSP00000348751	17	BTN3A2
ENSP00000292475	17	ATP5J2
ENSP00000215375	17	ATP5D
ENSP00000355090	17	ARHGAP11A
ENSP00000307004	17	APLF
ENSP00000297562	17	AP5Z1
ENSP00000303518	17	ANKRD49
ENSP00000253003	17	ADRM1
ENSP00000362304	17	ADAMTS14
ENSP00000360997	17	ADAMTS13
ENSP00000252669	17	ACSBG2
ENSP00000270593	17	ACPT
ENSP00000230048	17	ACOT13
ENSP00000282641	17	A1CF
ENSP00000266643	17	9-Mar
ENSP00000351813	17	5-Mar
ENSP00000278816	17	-
ENSP00000307781	17	-
ENSP00000324129	17	-
ENSP00000327691	17	-
ENSP00000330146	17	-
ENSP00000350541	17	-
ENSP00000352989	17	-
ENSP00000355033	17	-
ENSP00000364745	17	-
ENSP00000367784	17	-
ENSP00000368241	17	-
ENSP00000371742	17	-
ENSP00000372607	17	-
ENSP00000382790	17	-
ENSP00000386159	17	-
ENSP00000392001	17	-
ENSP00000414381	17	-
ENSP00000418813	17	-
ENSP00000382218	16	ZSWIM7
ENSP00000359958	16	ZRANB2

ENSP00000409514	16	ZNF717
ENSP00000318373	16	ZNF513
ENSP00000329141	16	ZNF37A
ENSP00000352444	16	ZNF33B
ENSP00000363556	16	ZNF32
ENSP00000302222	16	ZNF25
ENSP00000379847	16	ZNF143
ENSP00000343443	16	ZNF107
ENSP00000287218	16	ZFAND3
ENSP00000340590	16	ZCCHC16
ENSP00000329614	16	YBEY
ENSP00000328004	16	YAF2
ENSP00000384222	16	WWC2
ENSP00000328800	16	WBP2NL
ENSP00000381282	16	VIMP
ENSP00000345656	16	VAPA
ENSP00000254803	16	UTP3
ENSP00000296792	16	UTP15
ENSP00000297229	16	USP49
ENSP00000407818	16	USP46
ENSP00000381863	16	USP19
ENSP00000350409	16	UNC50
ENSP00000309198	16	UBE3C
ENSP00000369081	16	TXNDC5
ENSP00000362711	16	TXLNA
ENSP00000357597	16	TSPYL1
ENSP00000290846	16	TRMU
ENSP00000327994	16	TRIM50
ENSP00000256649	16	TRIM45
ENSP00000230099	16	TRIM38
ENSP00000278422	16	TMX2
ENSP00000371318	16	TMEM74B
ENSP00000281924	16	TMEM163
ENSP00000381204	16	TMEM123
ENSP00000261789	16	TM9SF1
ENSP00000318115	16	TIMM50
ENSP00000344411	16	TAS1R3
ENSP00000361057	16	SURF4
ENSP00000377176	16	SPATA7
ENSP00000320634	16	SLCO3A1
ENSP00000339260	16	SLC6A13
ENSP00000270570	16	SLC47A1
ENSP00000301305	16	SLC39A4
ENSP00000234256	16	SLC1A4
ENSP00000377112	16	SLC12A8
ENSP00000279477	16	SIRPB1

ENSP00000323328	16	SIGLEC7
ENSP00000365503	16	SHISA7
ENSP00000382327	16	SDR39U1
ENSP00000376421	16	SDK2
ENSP00000244930	16	SCGB2A1
ENSP00000334668	16	SAMD7
ENSP00000358902	16	SAMD10
ENSP00000337732	16	RUNDC3B
ENSP00000330631	16	RTN4RL1
ENSP00000417240	16	RPSAP58
ENSP00000339095	16	RPS7
ENSP00000379339	16	RPS29
ENSP00000367439	16	RPP38
ENSP00000404375	16	RPL36A
ENSP00000346015	16	RPL27A
ENSP00000184183	16	ROPN1
ENSP00000298690	16	RNASE7
ENSP00000332208	16	RFX6
ENSP00000249007	16	RFPL3
ENSP00000248980	16	RFPL2
ENSP00000240651	16	PYROXD1
ENSP00000376758	16	PTRH2
ENSP00000373153	16	PTPLB
ENSP00000381504	16	PRPS2
ENSP00000369634	16	PRPF40B
ENSP00000393845	16	PPIAL4G
ENSP00000221859	16	POLR2I
ENSP00000271715	16	POGZ
ENSP00000303191	16	PLRG1
ENSP00000259883	16	PGBD1
ENSP00000419975	16	PEX5L
ENSP00000327107	16	PDZD3
ENSP00000292823	16	PCYT1A
ENSP00000357448	16	PBXIP1
ENSP00000263549	16	PARP12
ENSP00000345395	16	PAPLN
ENSP00000364635	16	PADI2
ENSP00000373150	16	OR2W1
ENSP00000331086	16	ODF4
ENSP00000263032	16	NXF5
ENSP00000401177	16	NOL8
ENSP00000354387	16	NMRK1
ENSP00000263693	16	NKAIN1
ENSP00000307552	16	NINJ2
ENSP00000328464	16	NAT8L
ENSP00000261839	16	MYO5C

ENSP00000354849	16	MYBPC1
ENSP00000396774	16	MUC20
ENSP00000296003	16	MTMR14
ENSP00000264995	16	MRPL3
ENSP00000373261	16	MOBP
ENSP00000262430	16	MLYCD
ENSP00000296468	16	MFSD8
ENSP00000385610	16	MEX3C
ENSP00000267984	16	MESDC1
ENSP00000386908	16	MEGF11
ENSP00000359640	16	MCOLN2
ENSP00000285599	16	MAN2B2
ENSP00000366387	16	MAMDC2
ENSP00000358778	16	LRIF1
ENSP00000312535	16	LRFN4
ENSP00000362354	16	LPIN3
ENSP00000283415	16	LPCAT1
ENSP00000411932	16	LMOD2
ENSP00000376333	16	LLGL2
ENSP00000335223	16	LCE4A
ENSP00000335358	16	LCE3B
ENSP00000335006	16	LCE3A
ENSP00000357772	16	LCE2C
ENSP00000357768	16	LCE2A
ENSP00000334187	16	LCE1F
ENSP00000357759	16	LCE1E
ENSP00000357757	16	LCE1C
ENSP00000353203	16	LCE1B
ENSP00000175091	16	LAPTM4A
ENSP00000257951	16	KRT84
ENSP00000310216	16	KLRC4
ENSP00000370671	16	KLHL42
ENSP00000251691	16	KIAA1244
ENSP00000085068	16	ISOC2
ENSP00000402153	16	IQCJ
ENSP00000375033	16	IGHV3-48
ENSP00000370223	16	IDH3B
ENSP00000305721	16	IBTK
ENSP00000357791	16	HRNR
ENSP00000368965	16	HGSNAT
ENSP00000382269	16	HECTD1
ENSP00000369871	16	HAUS6
ENSP00000253458	16	GSE1
ENSP00000363433	16	GPRIN2
ENSP00000267015	16	GPR84
ENSP00000359675	16	GNG5

ENSP00000322716	16	GLYR1
ENSP00000260447	16	GCHFR
ENSP00000356729	16	FMO3
ENSP00000326022	16	FHL5
ENSP00000265029	16	FETUB
ENSP00000393776	16	FCHO2
ENSP00000373565	16	FAM83H
ENSP00000359652	16	EYS
ENSP00000360248	16	ENTPD1
ENSP00000243878	16	ENKD1
ENSP00000302289	16	EMB
ENSP00000232905	16	EIF1B
ENSP00000360107	16	EFHC1
ENSP00000236957	16	EEF1B2
ENSP00000338050	16	DTX3
ENSP00000295937	16	DNAH12
ENSP00000319651	16	DMRTA1
ENSP00000326219	16	DHRS4
ENSP00000349576	16	DCTD
ENSP00000410772	16	DAXX
ENSP00000264161	16	DARS
ENSP00000334246	16	CYP4Z1
ENSP00000269703	16	CYP4F22
ENSP00000413575	16	CROT
ENSP00000262424	16	CRISPLD2
ENSP00000262428	16	COTL1
ENSP00000261037	16	COL8A1
ENSP00000417628	16	CNTNAP4
ENSP00000305449	16	CNNM3
ENSP00000294053	16	CLPB
ENSP00000249806	16	CLN6
ENSP00000371505	16	CLEC6A
ENSP00000229332	16	CLEC4A
ENSP00000261340	16	CLEC2D
ENSP00000357683	16	CHTOP
ENSP00000333947	16	CHST15
ENSP00000355511	16	CHML
ENSP00000219172	16	CENPT
ENSP00000360381	16	CDCP2
ENSP00000275603	16	CCT6A
ENSP00000354416	16	CCL28
ENSP00000306776	16	CCDC110
ENSP00000267406	16	CBLN3
ENSP00000260743	16	CALHM2
ENSP00000385019	16	CACNA1I
ENSP00000369755	16	C2CD4B

ENSP00000253362	16	BPIFA2
ENSP00000361776	16	BEX4
ENSP00000219794	16	BCKDK
ENSP00000308334	16	ATP5S
ENSP00000349142	16	ATP5C1
ENSP00000370546	16	ARSD
ENSP00000276569	16	ARMC1
ENSP00000313506	16	ARHGAP28
ENSP00000357218	16	APOA1BP
ENSP00000325369	16	AP3S1
ENSP00000274000	16	AP1AR
ENSP00000331516	16	ANKRD44
ENSP00000249883	16	AMOTL2
ENSP00000177648	16	ALPK1
ENSP00000346577	16	AK5
ENSP00000238855	16	AFTPH
ENSP00000238618	16	ACYP1
ENSP00000362799	16	ACRC
ENSP00000333664	16	ACAA1
ENSP00000355637	16	ABCB10
ENSP00000242630	16	-
ENSP00000276530	16	-
ENSP00000328115	16	-
ENSP00000350649	16	-
ENSP00000351650	16	-
ENSP00000358387	16	-
ENSP00000371113	16	-
ENSP00000374000	16	-
ENSP00000382600	16	-
ENSP00000389075	16	-
ENSP00000404580	16	-
ENSP00000321963	15	ZSCAN4
ENSP00000337008	15	ZNF644
ENSP00000304769	15	ZNF467
ENSP00000338860	15	ZNF444
ENSP00000332750	15	ZKSCAN8
ENSP00000345793	15	ZC3H7B
ENSP00000161863	15	YTHDC2
ENSP00000362135	15	YRDC
ENSP00000310405	15	XCR1
ENSP00000295121	15	WDR92
ENSP00000261167	15	WBP11
ENSP00000362481	15	UPRT
ENSP00000334276	15	UGT2B28
ENSP00000286604	15	UGT2A1
ENSP00000302801	15	TRMT61B

ENSP00000374866	15	TRGV8
ENSP00000374871	15	TRGV1
ENSP00000307519	15	TMPRSS11E
ENSP00000299596	15	TMEM41B
ENSP00000366593	15	TMEM201
ENSP00000222284	15	TMEM147
ENSP00000360184	15	TM9SF3
ENSP00000365766	15	TIMM17B
ENSP00000355471	15	TFB2M
ENSP00000296096	15	TCF23
ENSP00000321259	15	TALDO1
ENSP00000381030	15	STK32A
ENSP00000338030	15	STARD7
ENSP00000340703	15	SPRR2B
ENSP00000295872	15	SPICE1
ENSP00000332592	15	SPAG16
ENSP00000264694	15	SNX25
ENSP00000311837	15	SNTG2
ENSP00000244227	15	SNRNP27
ENSP00000328940	15	SLX1B
ENSP00000251303	15	SLX1A
ENSP00000366283	15	SLITRK5
ENSP00000202831	15	SLC8B1
ENSP00000367770	15	SLC35B4
ENSP00000297282	15	SLC13A4
ENSP00000412361	15	SIGLEC11
ENSP00000264382	15	SI
ENSP00000371512	15	SGCZ
ENSP00000269491	15	SERPINB12
ENSP00000239944	15	SERP1
ENSP00000341538	15	SEC61G
ENSP00000345262	15	SCOC
ENSP00000326924	15	SCAPER
ENSP00000333358	15	RNASE10
ENSP00000347839	15	RAB11FIP2
ENSP00000357882	15	PSTK
ENSP00000300557	15	PRR15L
ENSP00000358224	15	PPIAL4C
ENSP00000361338	15	PPAPDC3
ENSP00000350098	15	POP5
ENSP00000334564	15	POLR3C
ENSP00000372316	15	POLN
ENSP00000311368	15	POLD4
ENSP00000354532	15	PNP
ENSP00000358223	15	PNLIP
ENSP00000326706	15	PLEKHO2

ENSP00000291539	15	PDE9A
ENSP00000377527	15	PC
ENSP00000307164	15	PATE1
ENSP00000315693	15	PAM16
ENSP00000363654	15	PALM2-AKAP2
ENSP00000244221	15	PAIP2B
ENSP00000289619	15	PAGE5
ENSP00000358131	15	OTUD7B
ENSP00000305926	15	OTUD7A
ENSP00000203664	15	OTUB2
ENSP00000039007	15	OTC
ENSP00000264151	15	OSGEPL1
ENSP00000324534	15	OR14C36
ENSP00000385636	15	OBSL1
ENSP00000342262	15	NUP43
ENSP00000293973	15	NTN3
ENSP00000267017	15	NPFF
ENSP00000370589	15	NOP56
ENSP00000360412	15	NOC3L
ENSP00000368389	15	NGRN
ENSP00000324792	15	NFATC2IP
ENSP00000346196	15	NDUFV3
ENSP00000210444	15	NANS
ENSP00000395253	15	MUC19
ENSP00000308845	15	MRPS12
ENSP00000370930	15	MRPL23
ENSP00000318154	15	MRFAP1L1
ENSP00000377252	15	MON2
ENSP00000297347	15	MED30
ENSP00000302537	15	MDM1
ENSP00000362136	15	MANEAL
ENSP00000225972	15	LRRC59
ENSP00000318374	15	LENG8
ENSP00000278671	15	LAMTOR1
ENSP00000293525	15	KRT86
ENSP00000007735	15	KRT33A
ENSP00000225899	15	KRT32
ENSP00000264651	15	KRT24
ENSP00000260598	15	KHK
ENSP00000347409	15	KEL
ENSP00000357067	15	KCNJ9
ENSP00000420040	15	KCNIP2
ENSP00000322730	15	ITM2C
ENSP00000299732	15	IQCD
ENSP00000303977	15	INO80E
ENSP00000259211	15	IL36A

ENSP00000264257	15	IL1RL2
ENSP00000412897	15	IFNA4
ENSP00000394494	15	IFNA13
ENSP00000265395	15	HIBADH
ENSP00000261170	15	GUCY2C
ENSP00000351644	15	GTPBP3
ENSP00000355551	15	GPR137B
ENSP00000396441	15	GPATCH4
ENSP00000299314	15	GNPTAB
ENSP00000301671	15	GHDC
ENSP00000365920	15	GCNT1
ENSP00000359490	15	GBP4
ENSP00000305603	15	FUT3
ENSP00000334665	15	FSCN2
ENSP00000417601	15	FBXL2
ENSP00000343279	15	FAM83G
ENSP00000383933	15	FAM19A5
ENSP00000354270	15	FAM107A
ENSP00000362187	15	FAM102A
ENSP00000265564	15	EXOSC7
ENSP00000216554	15	EIF5
ENSP00000362688	15	EIF3I
ENSP00000329715	15	DRG1
ENSP00000262960	15	DPP9
ENSP00000371835	15	DEFB126
ENSP00000268676	15	DEF8
ENSP00000357920	15	DDO
ENSP00000367715	15	DCDC2
ENSP00000371870	15	DAZ2
ENSP00000355086	15	DARS2
ENSP00000358953	15	CUEDC2
ENSP00000303963	15	CTRB2
ENSP00000282957	15	CPB1
ENSP00000222481	15	CPA2
ENSP00000251166	15	CORO7
ENSP00000322316	15	COQ7
ENSP00000258654	15	COG3
ENSP00000355470	15	CNST
ENSP00000402460	15	CLSTN2
ENSP00000336994	15	CLPTM1
ENSP00000327599	15	CLEC4G
ENSP00000308870	15	CLDN15
ENSP00000302629	15	CHSY3
ENSP00000317468	15	CHMP6
ENSP00000371787	15	CEMP1
ENSP00000310966	15	CD3EAP

ENSP00000256151	15	CCDC59
ENSP00000348933	15	CCDC25
ENSP00000005284	15	CACNG3
ENSP00000223642	15	C5
ENSP00000364321	15	C4B
ENSP00000291577	15	C21orf33
ENSP00000375889	15	C19orf47
ENSP00000261700	15	C14orf166
ENSP00000260723	15	BTBD16
ENSP00000318852	15	BPNT1
ENSP00000328030	15	BEX5
ENSP00000243578	15	B9D2
ENSP00000320965	15	B4GALT3
ENSP00000343318	15	B3GALT5
ENSP00000286371	15	ATP1B3
ENSP00000283290	15	ATG3
ENSP00000261191	15	ASUN
ENSP00000268042	15	ARRDC4
ENSP00000366522	15	ARHGAP39
ENSP00000365081	15	APOM
ENSP00000267116	15	ANKRD52
ENSP00000346987	15	ANAPC13
ENSP00000252505	15	ALLC
ENSP00000242375	15	AKR1D1
ENSP00000380184	15	AGPAT6
ENSP00000264595	15	AGA
ENSP00000355493	15	ADSS
ENSP00000358407	15	ADAM30
ENSP00000243903	15	ACTR5
ENSP00000289416	15	ACSM3
ENSP00000261406	15	-
ENSP00000262414	15	-
ENSP00000279839	15	-
ENSP00000282606	15	-
ENSP00000342595	15	-
ENSP00000350047	15	-
ENSP00000351760	15	-
ENSP00000356298	15	-
ENSP00000359368	15	-
ENSP00000361154	15	-
ENSP00000366492	15	-
ENSP00000397716	15	-
ENSP00000399765	15	-
ENSP00000403377	15	-
ENSP00000406585	15	-
ENSP00000406867	15	-

ENSP00000408784	15	-
ENSP00000420822	15	-
ENSP00000225410	14	ZNHIT3
ENSP00000309812	14	ZNF80
ENSP00000276123	14	ZNF711
ENSP00000303696	14	ZNF57
ENSP00000378503	14	ZBTB9
ENSP00000327716	14	WRB
ENSP00000380675	14	WEE2
ENSP00000314193	14	WDR75
ENSP00000417185	14	VWA1
ENSP00000366565	14	VPS28
ENSP00000316092	14	VARS2
ENSP00000192788	14	UHRF1BP1
ENSP00000365938	14	UGGT2
ENSP00000377381	14	UBTD2
ENSP00000297661	14	UBAP1
ENSP00000305654	14	TVP23B
ENSP00000278279	14	TUT1
ENSP00000258796	14	TTYH3
ENSP00000371559	14	TSSC1
ENSP00000267970	14	TSPAN3
ENSP00000284320	14	TOMM70A
ENSP00000338165	14	TMEM39B
ENSP00000315635	14	TMEM25
ENSP00000360715	14	TMEM189
ENSP00000358430	14	TECTB
ENSP00000313886	14	TCEANC
ENSP00000299290	14	TBATA
ENSP00000287652	14	TATDN2
ENSP00000346576	14	SYTL2
ENSP00000369500	14	SYAP1
ENSP00000345477	14	ST3GAL2
ENSP00000364665	14	SSX2B
ENSP00000364041	14	SPIN2A
ENSP00000375909	14	SPHKAP
ENSP00000308893	14	SNED1
ENSP00000340766	14	SMG7
ENSP00000245680	14	SLC35F5
ENSP00000358565	14	SLC35A1
ENSP00000290075	14	SLC25A37
ENSP00000362159	14	SLC25A25
ENSP00000345580	14	SLC25A10
ENSP00000263512	14	SLC10A3
ENSP00000319417	14	SKA3
ENSP00000333433	14	SKA2

ENSP00000313079	14	SHISA2
ENSP00000337133	14	SERPINA9
ENSP00000349929	14	SDCCAG3
ENSP00000281142	14	SCLT1
ENSP00000345445	14	SAMM50
ENSP00000339795	14	RPL7
ENSP00000420821	14	RPL41
ENSP00000380156	14	RPL32
ENSP00000283646	14	RPIA
ENSP00000340369	14	RINL
ENSP00000419786	14	RGAG1
ENSP00000330269	14	RESP18
ENSP00000302088	14	RER1
ENSP00000331342	14	RAB11FIP1
ENSP00000298999	14	R3HCC1L
ENSP00000281273	14	QTRTD1
ENSP00000261875	14	PTPLAD1
ENSP00000363373	14	PTBP3
ENSP00000275605	14	PSPH
ENSP00000329915	14	PSMG1
ENSP00000367164	14	PRR20A
ENSP00000315379	14	PRPF3
ENSP00000260648	14	PREPL
ENSP00000339764	14	PRDM8
ENSP00000286175	14	PPIL3
ENSP00000335614	14	PPFIA3
ENSP00000264220	14	PPAT
ENSP00000371307	14	PPAPDC2
ENSP00000225573	14	PNPO
ENSP00000318075	14	PLEKHJ1
ENSP00000219345	14	PLA2G15
ENSP00000297373	14	PHKG1
ENSP00000313504	14	PHKB
ENSP00000322807	14	PDXDC1
ENSP00000323313	14	PDIA5
ENSP00000295266	14	PDHA2
ENSP00000307854	14	PCMTD2
ENSP00000298281	14	PCF11
ENSP00000171757	14	P2RY10
ENSP00000305631	14	NXNL1
ENSP00000371155	14	NUPL1
ENSP00000310668	14	NUP93
ENSP00000352911	14	NUDT16
ENSP00000235628	14	NT5C1A
ENSP00000355944	14	NSL1
ENSP00000184266	14	NDUFB4

ENSP00000323076	14	NDUFAF3
ENSP00000252102	14	NDUFA2
ENSP00000351484	14	NAALADL1
ENSP00000297130	14	MYOZ3
ENSP00000386213	14	MYO3B
ENSP00000358136	14	MTMR11
ENSP00000356290	14	MTHFD1L
ENSP00000218721	14	MLNR
ENSP00000329918	14	MEX3B
ENSP00000324944	14	MBOAT1
ENSP00000261483	14	MAN2A1
ENSP00000298974	14	MAGEA9
ENSP00000261267	14	LYZ
ENSP00000345917	14	LYAR
ENSP00000344242	14	LRRC17
ENSP00000251377	14	LILRA2
ENSP00000312273	14	LGI4
ENSP00000339251	14	KRTDAP
ENSP00000379607	14	KNCN
ENSP00000371327	14	KLRC2
ENSP00000298307	14	KLHDC2
ENSP00000347532	14	KIAA1598
ENSP00000379654	14	KIAA0430
ENSP00000386918	14	KDELR3
ENSP00000228495	14	KCTD10
ENSP00000307694	14	KCNS1
ENSP00000302166	14	KCNK9
ENSP00000344820	14	KCNK7
ENSP00000307265	14	IRF2BP1
ENSP00000306523	14	INSM2
ENSP00000347129	14	INCA1
ENSP00000342557	14	IL4I1
ENSP00000263326	14	IL37
ENSP00000295980	14	IL17RE
ENSP00000377370	14	IGSF11
ENSP00000414456	14	IGLV9-49
ENSP00000420020	14	IGKV1-33
ENSP00000242591	14	IFT81
ENSP00000289753	14	HTR6
ENSP00000341267	14	HMGN3
ENSP00000363614	14	HMGCL
ENSP00000352706	14	HIBCH
ENSP00000228827	14	GPN3
ENSP00000204679	14	GNPTG
ENSP00000222286	14	GAPDHS
ENSP00000339067	14	FGFBP3

ENSP00000357682	14	FANK1
ENSP00000259963	14	FAM8A1
ENSP00000304642	14	FAM134B
ENSP00000362409	14	FAM129B
ENSP00000319883	14	EMR2
ENSP00000359778	14	ELTD1
ENSP00000361536	14	ELOVL1
ENSP00000253108	14	EIF3G
ENSP00000364687	14	EHMT2
ENSP00000374490	14	DNAH17
ENSP00000251636	14	DHX29
ENSP00000288490	14	DGKI
ENSP00000377344	14	DDX60
ENSP00000323680	14	DCAF11
ENSP00000366208	14	CST11
ENSP00000365243	14	COX4I2
ENSP00000354762	14	COA3
ENSP00000322280	14	CLRN1
ENSP00000312663	14	CHRNA9
ENSP00000262127	14	CEP76
ENSP00000404464	14	CCBE1
ENSP00000374183	14	CAMSAP1
ENSP00000262138	14	CACNG4
ENSP00000322061	14	C7
ENSP00000406706	14	C6orf25
ENSP00000412786	14	C4B_2
ENSP00000407942	14	C4B
ENSP00000364444	14	C4A
ENSP00000388662	14	C4A
ENSP00000396688	14	C4A
ENSP00000264434	14	C2orf42
ENSP00000360320	14	C1orf177
ENSP00000254336	14	C19orf57
ENSP00000338990	14	C12orf44
ENSP00000406541	14	C11orf21
ENSP00000330200	14	BTNL9
ENSP00000244519	14	BTN3A3
ENSP00000304151	14	BOP1
ENSP00000321507	14	AZIN1
ENSP00000262919	14	ATRN
ENSP00000300688	14	ATP5L
ENSP00000260952	14	ASNSD1
ENSP00000264914	14	ARSB
ENSP00000344577	14	APOL3
ENSP00000335287	14	ANKRD46
ENSP00000307199	14	AHSP

ENSP00000338512	14	AGTPBP1
ENSP00000264167	14	AGPS
ENSP00000299164	14	ADAMTS15
ENSP00000358241	14	ACP6
ENSP00000411471	14	ABCF3
ENSP00000253577	14	ABCB7
ENSP00000268251	14	ABAT
ENSP00000264432	14	-
ENSP00000302005	14	-
ENSP00000321876	14	-
ENSP00000346128	14	-
ENSP00000350017	14	-
ENSP00000358371	14	-
ENSP00000364762	14	-
ENSP00000368252	14	-
ENSP00000383155	14	-
ENSP00000395044	14	-
ENSP00000397762	14	-
ENSP00000412888	14	-
ENSP00000371051	13	ZZEF1
ENSP00000254323	13	ZSWIM4
ENSP00000292450	13	ZSCAN21
ENSP00000331462	13	ZNF704
ENSP00000386845	13	ZNF385B
ENSP00000379368	13	ZNF35
ENSP00000406147	13	ZNF257
ENSP00000307774	13	ZNF239
ENSP00000340514	13	ZNF14
ENSP00000399605	13	ZFHX4
ENSP00000359891	13	ZDHHC16
ENSP00000417677	13	ZBTB8OS
ENSP00000366396	13	XRN2
ENSP00000327821	13	XPOT
ENSP00000378857	13	WDR6
ENSP00000251289	13	WDR18
ENSP00000218056	13	WDR13
ENSP00000318684	13	VSIG2
ENSP00000318629	13	VPS37A
ENSP00000368544	13	VIT
ENSP00000263966	13	USP13
ENSP00000274278	13	UGT3A1
ENSP00000356853	13	UCK2
ENSP00000396068	13	UBR3
ENSP00000323687	13	UBE2O
ENSP00000360821	13	UBAC1
ENSP00000250101	13	TXNDC17

ENSP00000289407	13	TSPAN33
ENSP00000354543	13	TSC22D2
ENSP00000305161	13	TRIM56
ENSP00000323913	13	TRIM55
ENSP00000006275	13	TRAPPC6A
ENSP00000356599	13	TOR3A
ENSP00000299882	13	TMPRSS5
ENSP00000297632	13	TMEM65
ENSP00000386292	13	TMEM194B
ENSP00000350718	13	TMCC2
ENSP00000405455	13	TKT
ENSP00000251864	13	TDRD1
ENSP00000378546	13	TAPBP
ENSP00000290607	13	STARD9
ENSP00000304032	13	STARD5
ENSP00000276480	13	ST18
ENSP00000267884	13	SRP14
ENSP00000288680	13	SPPL3
ENSP00000387266	13	SPIRE1
ENSP00000359549	13	SPANXA2
ENSP00000269053	13	SPACA3
ENSP00000304429	13	SNX7
ENSP00000263694	13	SNRNP40
ENSP00000357674	13	SNAPIN
ENSP00000355261	13	SMG5
ENSP00000359729	13	SLC9A6
ENSP00000333638	13	SLC52A2
ENSP00000296327	13	SLC51A
ENSP00000336888	13	SLC44A2
ENSP00000322020	13	SLC25A22
ENSP00000408509	13	SH2D5
ENSP00000360076	13	SGIP1
ENSP00000249786	13	SERF2
ENSP00000350215	13	SEMA5B
ENSP00000381823	13	SEC24A
ENSP00000357901	13	SCNM1
ENSP00000390783	13	SCFD1
ENSP00000311712	13	RTP1
ENSP00000225430	13	RPL19
ENSP00000354739	13	RPL12
ENSP00000315859	13	RNPS1
ENSP00000391432	13	RNPC3
ENSP00000347548	13	RNF220
ENSP00000344489	13	RNF133
ENSP00000322242	13	RNF10
ENSP00000320623	13	RMND5B

ENSP00000386228	13	RIMS2
ENSP00000334134	13	RGS9BP
ENSP00000345487	13	QRFP
ENSP00000276616	13	PSKH2
ENSP00000319140	13	PSG11
ENSP00000245796	13	PSD4
ENSP00000299275	13	PLEKHA5
ENSP00000333751	13	PLCXD3
ENSP00000371073	13	PLCXD1
ENSP00000394848	13	PLCXD1
ENSP00000337397	13	PKD1L2
ENSP00000352185	13	PHF2
ENSP00000352672	13	PGLYRP4
ENSP00000313490	13	PFAS
ENSP00000370224	13	PARM1
ENSP00000406197	13	PAQR8
ENSP00000311665	13	PAAF1
ENSP00000313384	13	OR1E1
ENSP00000332511	13	OLFML1
ENSP00000224950	13	OBFC1
ENSP00000339479	13	NT5C2
ENSP00000330070	13	NPW
ENSP00000330284	13	NPBWR1
ENSP00000416658	13	NME6
ENSP00000309767	13	NLRP6
ENSP00000358815	13	NEURL1B
ENSP00000385149	13	NEU4
ENSP00000246117	13	NCLN
ENSP00000244204	13	NAGK
ENSP00000354972	13	NAA35
ENSP00000357360	13	MTX1
ENSP00000262146	13	MTFR1
ENSP00000365693	13	MSRB2
ENSP00000245539	13	MRPS7
ENSP00000362036	13	MRPS16
ENSP00000279242	13	MRPL49
ENSP00000359518	13	MRGBP
ENSP00000244051	13	MOCS3
ENSP00000245551	13	MIF4GD
ENSP00000281416	13	MFSD6
ENSP00000299610	13	MFAP4
ENSP00000408058	13	METTL11B
ENSP00000230588	13	MEP1A
ENSP00000342343	13	MED22
ENSP00000327070	13	MDH2
ENSP00000347896	13	MBD6

ENSP00000343164	13	MATN4
ENSP00000378851	13	MAPK1IP1L
ENSP00000349785	13	MAP1LC3C
ENSP00000226578	13	MANBA
ENSP00000360525	13	MAGOH
ENSP00000364777	13	MAGED4
ENSP00000368273	13	MAGEB2
ENSP00000293872	13	LUC7L
ENSP00000343331	13	LIPI
ENSP00000375629	13	LILRB2
ENSP00000310551	13	LCLAT1
ENSP00000257867	13	LACRT
ENSP00000225550	13	KRT37
ENSP00000340083	13	KRCC1
ENSP00000320821	13	KIF1C
ENSP00000357304	13	KIAA0907
ENSP00000362328	13	KCNK17
ENSP00000368797	13	KBTBD7
ENSP00000265239	13	IQCG
ENSP00000296980	13	IL22RA2
ENSP00000396301	13	IK
ENSP00000419139	13	IGHD5-24
ENSP00000359783	13	IFI44
ENSP00000367623	13	HSPA14
ENSP00000230236	13	HSD17B8
ENSP00000387753	13	HSD17B8
ENSP00000403538	13	HSD17B8
ENSP00000406488	13	HSD17B8
ENSP00000168216	13	HSD17B10
ENSP00000256906	13	HRH4
ENSP00000366506	13	HRH2
ENSP00000243103	13	HOXC12
ENSP00000303997	13	GTPBP2
ENSP00000361219	13	GTF3C4
ENSP00000276218	13	GPR119
ENSP00000170564	13	GPATCH1
ENSP00000311876	13	GNPDA1
ENSP00000373918	13	GARS
ENSP00000315835	13	GALNT11
ENSP00000270310	13	FXYD7
ENSP00000242257	13	FTSJ2
ENSP00000319726	13	FRMD8
ENSP00000300784	13	FN3K
ENSP00000313159	13	FBXO34
ENSP00000369618	13	FASTKD5
ENSP00000361254	13	FAM213A

ENSP00000278829	13	FADS3
ENSP00000253496	13	F12
ENSP00000353270	13	ERGIC2
ENSP00000295571	13	EOGT
ENSP00000317431	13	ENTHD1
ENSP00000408583	13	EHMT2
ENSP00000220325	13	EHD4
ENSP00000260605	13	DYNC2LI1
ENSP00000225729	13	DRG2
ENSP00000389630	13	DPY19L4
ENSP00000367001	13	DPP6
ENSP00000322181	13	DPM2
ENSP00000273075	13	DNPEP
ENSP00000265028	13	DNAJB11
ENSP00000322191	13	DEFB104B
ENSP00000380495	13	DDX51
ENSP00000346236	13	DDX46
ENSP00000322524	13	DCTPP1
ENSP00000414906	13	DCTN4
ENSP00000341504	13	DCAKD
ENSP00000297056	13	DAGLB
ENSP00000332444	13	CSTF2T
ENSP00000383938	13	CRELD2
ENSP00000290776	13	CPNE2
ENSP00000382356	13	COL28A1
ENSP00000366275	13	CNNM4
ENSP00000251102	13	CNGB1
ENSP00000364831	13	CLCNKB
ENSP00000363041	13	CISD1
ENSP00000310440	13	CHMP2A
ENSP00000365782	13	CEP78
ENSP00000372319	13	CDY2B
ENSP00000311656	13	CCPG1
ENSP00000351727	13	CCER1
ENSP00000311695	13	CCDC85B
ENSP00000404220	13	CCDC155
ENSP00000381553	13	CCDC11
ENSP00000302227	13	CCBL1
ENSP00000309052	13	CATSPER1
ENSP00000293255	13	CABP5
ENSP00000224181	13	C8G
ENSP00000345107	13	C2orf88
ENSP00000258457	13	C2orf49
ENSP00000328677	13	C2CD4C
ENSP00000380959	13	C17orf67
ENSP00000261721	13	BTBD1

ENSP00000276416	13	BIN3
ENSP00000365131	13	BAG6
ENSP00000352522	13	ATP6V1H
ENSP00000276390	13	ATP6V1B2
ENSP00000389649	13	ATP5J
ENSP00000243997	13	ATP5E
ENSP00000371372	13	ATP12A
ENSP00000301776	13	ASRGL1
ENSP00000256578	13	AMPD2
ENSP00000286190	13	ALS2CR12
ENSP00000258888	13	ALPK3
ENSP00000299626	13	ALG8
ENSP00000312126	13	ADCY4
ENSP00000267499	13	ADAM21
ENSP00000348459	13	ACMSD
ENSP00000244571	13	AARS2
ENSP00000243152	13	-
ENSP00000265306	13	-
ENSP00000267857	13	-
ENSP00000299373	13	-
ENSP00000312350	13	-
ENSP00000316590	13	-
ENSP00000318869	13	-
ENSP00000329463	13	-
ENSP00000336673	13	-
ENSP00000348664	13	-
ENSP00000366856	13	-
ENSP00000370766	13	-
ENSP00000373087	13	-
ENSP00000373973	13	-
ENSP00000378411	13	-
ENSP00000389835	13	-
ENSP00000390155	13	-
ENSP00000398251	13	-
ENSP00000411368	13	-
ENSP00000414669	13	-
ENSP00000420828	13	-
ENSP00000364023	12	ZXDB
ENSP00000412999	12	ZNF805
ENSP00000340841	12	ZNF621
ENSP00000270451	12	ZNF581
ENSP00000350773	12	ZNF329
ENSP00000005082	12	ZNF195
ENSP00000324274	12	ZCCHC10
ENSP00000351052	12	ZC3HC1
ENSP00000338788	12	ZC3H15

ENSP00000263849	12	ZC2HC1A
ENSP00000270708	12	WRAP73
ENSP00000352820	12	WDSUB1
ENSP00000263150	12	WDR37
ENSP00000301765	12	VPS37C
ENSP00000333255	12	VMA21
ENSP00000309031	12	VCPIP1
ENSP00000252597	12	USHBP1
ENSP00000227471	12	UNC93B1
ENSP00000313953	12	TTC27
ENSP00000314073	12	TRPT1
ENSP00000359160	12	TRMT13
ENSP00000350352	12	TRMT1
ENSP00000264029	12	TREH
ENSP00000361900	12	TOMM34
ENSP00000338371	12	TNRC6B
ENSP00000262225	12	TMED2
ENSP00000295899	12	THOC7
ENSP00000353093	12	TCEAL8
ENSP00000264866	12	TBC1D19
ENSP00000215790	12	TBC1D10A
ENSP00000364520	12	TAS1R2
ENSP00000297875	12	SYTL5
ENSP00000225777	12	SYNGR2
ENSP00000361092	12	SURF6
ENSP00000324302	12	STXBP6
ENSP00000249344	12	STRIP2
ENSP00000320431	12	ST8SIA3
ENSP00000336733	12	ST6GALNAC4
ENSP00000329214	12	ST6GALNAC3
ENSP00000270061	12	SSBP4
ENSP00000365830	12	SOWAHC
ENSP00000272348	12	SNRPG
ENSP00000215829	12	SNRPD3
ENSP00000371297	12	SNPH
ENSP00000297151	12	SLU7
ENSP00000360967	12	SLC6A14
ENSP00000352655	12	SLC39A10
ENSP00000253270	12	SLC35D2
ENSP00000365413	12	SLC28A3
ENSP00000344801	12	SLC24A2
ENSP00000316905	12	SFXN1
ENSP00000383145	12	SFI1
ENSP00000362110	12	SF3A3
ENSP00000368477	12	SERP2
ENSP00000376268	12	SEC14L1

ENSP00000355467	12	SCCPDH
ENSP00000395259	12	SCAND3
ENSP00000301995	12	SCAND1
ENSP00000328708	12	RXFP3
ENSP00000259030	12	RTP4
ENSP00000417464	12	RRP1
ENSP00000262584	12	RPL8
ENSP00000338293	12	ROMO1
ENSP00000295640	12	RNPEP
ENSP00000289248	12	RHBDL2
ENSP00000297164	12	RELL2
ENSP00000313385	12	RELL1
ENSP00000171214	12	RDH8
ENSP00000254695	12	RAP1GAP2
ENSP00000251507	12	RABGAP1L
ENSP00000355385	12	R3HDM4
ENSP00000351536	12	QSOX2
ENSP00000378782	12	QPRT
ENSP00000307567	12	QARS
ENSP00000305005	12	PSG8
ENSP00000292125	12	PSG6
ENSP00000274710	12	PSD2
ENSP00000365175	12	PRRC2A
ENSP00000348010	12	PRPF39
ENSP00000349124	12	PPP4R2
ENSP00000343885	12	PPA2
ENSP00000367029	12	POLR1E
ENSP00000345359	12	PODXL2
ENSP00000323302	12	POC1B
ENSP00000370430	12	PNPLA4
ENSP00000356104	12	PM20D1
ENSP00000330442	12	PLB1
ENSP00000362778	12	PI16
ENSP00000329968	12	PHKG2
ENSP00000331106	12	PEX26
ENSP00000241041	12	PEX16
ENSP00000359382	12	PASD1
ENSP00000264883	12	NUP54
ENSP00000264670	12	NSUN2
ENSP00000327268	12	NDUFV2
ENSP00000296684	12	NDUFS4
ENSP00000263774	12	NDUFS3
ENSP00000356972	12	NDUFS2
ENSP00000347988	12	NDUFA5
ENSP00000339720	12	NDUFA4
ENSP00000205194	12	NAT14

ENSP00000251127	12	NALCN
ENSP00000335636	12	NAA20
ENSP00000379108	12	MTHFD2L
ENSP00000356001	12	MRPL18
ENSP00000262794	12	MOV10L1
ENSP00000359819	12	MOSPD1
ENSP00000232603	12	MORC1
ENSP00000310189	12	MOB1B
ENSP00000321455	12	MICU3
ENSP00000256186	12	MICALCL
ENSP00000264266	12	MFSD1
ENSP00000233114	12	MDH1
ENSP00000307093	12	MAP6
ENSP00000279068	12	LSM14B
ENSP00000299194	12	LRTM2
ENSP00000386357	12	LRRTM4
ENSP00000355963	12	LPGAT1
ENSP00000359691	12	LGSN
ENSP00000280704	12	LDHC
ENSP00000377954	12	LARS
ENSP00000251646	12	KRT33B
ENSP00000343930	12	KLHL17
ENSP00000301332	12	KIFC2
ENSP00000268919	12	KIF2B
ENSP00000263468	12	KIAA1377
ENSP00000259335	12	KIAA0368
ENSP00000303928	12	KIAA0232
ENSP00000258739	12	KDELR2
ENSP00000352527	12	KCNK5
ENSP00000295568	12	KBTBD8
ENSP00000300961	12	JSRP1
ENSP00000278071	12	ITPRIP
ENSP00000273283	12	ITIH1
ENSP00000358966	12	IMPG1
ENSP00000259213	12	IL36B
ENSP00000299157	12	IKBIP
ENSP00000375016	12	IGHV3-21
ENSP00000239347	12	IFNA7
ENSP00000316772	12	HMHA1
ENSP00000407431	12	HLA-C
ENSP00000413992	12	HLA-C
ENSP00000014930	12	HEBP1
ENSP00000304668	12	HARS
ENSP00000253237	12	GRWD1
ENSP00000348161	12	GPR160
ENSP00000338573	12	GNL3L

ENSP00000259727	12	GMPR
ENSP00000367172	12	GKN1
ENSP00000368119	12	GALT
ENSP00000357158	12	FCRL1
ENSP00000367319	12	FBXO47
ENSP00000320309	12	FARSA
ENSP00000359292	12	FAM178A
ENSP00000331915	12	FAM101B
ENSP00000265843	12	EXPH5
ENSP00000384900	12	ERLIN1
ENSP00000185150	12	ERLEC1
ENSP00000330375	12	EPGN
ENSP00000271764	12	EIF2D
ENSP00000394869	12	EIF2B4
ENSP00000258198	12	DYNC1LI2
ENSP00000319705	12	DUOXA2
ENSP00000322885	12	DTX2
ENSP00000339208	12	DPP8
ENSP00000316053	12	DNAJB8
ENSP00000413684	12	DNAJB5
ENSP00000401514	12	DNAH1
ENSP00000270223	12	DMWD
ENSP00000210060	12	DHPS
ENSP00000335538	12	DEFB131
ENSP00000365486	12	DEFB123
ENSP00000253381	12	DEFB118
ENSP00000323858	12	DDX54
ENSP00000302805	12	DDI1
ENSP00000371802	12	DAZ3
ENSP00000310149	12	CYP2W1
ENSP00000416598	12	CYP21A2
ENSP00000371294	12	CTNS
ENSP00000361650	12	CRIP3
ENSP00000265085	12	CPEB4
ENSP00000288532	12	COQ5
ENSP00000369862	12	COL4A3BP
ENSP00000303153	12	COL22A1
ENSP00000244728	12	COL21A1
ENSP00000351767	12	COL20A1
ENSP00000368814	12	CMTM1
ENSP00000255427	12	CHIT1
ENSP00000251424	12	CFHR4
ENSP00000336524	12	CEP63
ENSP00000317156	12	CEP192
ENSP00000382271	12	CEP152
ENSP00000364737	12	CENPP

ENSP00000348527	12	CENPL
ENSP00000304370	12	CDC40
ENSP00000301458	12	CD320
ENSP00000304903	12	CD2BP2
ENSP00000402239	12	CCDC159
ENSP00000321813	12	CAMTA2
ENSP00000270458	12	CACNG8
ENSP00000345659	12	CA7
ENSP00000393004	12	C6orf136
ENSP00000274258	12	C5orf42
ENSP00000243189	12	C1orf63
ENSP00000322609	12	C1orf173
ENSP00000306477	12	BTD
ENSP00000042931	12	BEST2
ENSP00000323479	12	B3GALNT1
ENSP00000348205	12	ATP5G1
ENSP00000355173	12	ATG9A
ENSP00000370718	12	ASMTL
ENSP00000386532	12	ASB18
ENSP00000352992	12	ART5
ENSP00000383059	12	ARGLU1
ENSP00000381250	12	APOF
ENSP00000261842	12	AP4E1
ENSP00000256658	12	AP4B1
ENSP00000377148	12	AP1G1
ENSP00000299381	12	ANAPC16
ENSP00000265605	12	ALDH8A1
ENSP00000312250	12	ADPGK
ENSP00000390849	12	ABHD5
ENSP00000209873	12	AAAS
ENSP00000274140	12	6-Mar
ENSP00000009606	12	-
ENSP00000222124	12	-
ENSP00000228958	12	-
ENSP00000235799	12	-
ENSP00000301037	12	-
ENSP00000303282	12	-
ENSP00000338397	12	-
ENSP00000388197	12	-
ENSP00000393085	12	-
ENSP00000393549	12	-
ENSP00000396814	12	-
ENSP00000397329	12	-
ENSP00000400255	12	-
ENSP00000406480	12	-
ENSP00000408936	12	-

ENSP00000411680	12	-
ENSP00000420825	12	-
ENSP00000402343	11	ZNF469
ENSP00000337363	11	ZNF394
ENSP00000362527	11	ZNF362
ENSP00000326921	11	ZFYVE1
ENSP00000220669	11	ZFAND1
ENSP00000274496	11	YIPF5
ENSP00000286760	11	WHAMM
ENSP00000348129	11	WDR88
ENSP00000335522	11	WDR86
ENSP00000376139	11	WDR45B
ENSP00000216456	11	VTI1B
ENSP00000255304	11	USPL1
ENSP00000385700	11	USP24
ENSP00000219281	11	USB1
ENSP00000386126	11	UMODL1
ENSP00000356982	11	UFC1
ENSP00000269346	11	TTYH2
ENSP00000354168	11	TSSK6
ENSP00000361229	11	TSPAN14
ENSP00000331620	11	TSPAN10
ENSP00000252826	11	TRPM4
ENSP00000285805	11	TRIM74
ENSP00000318615	11	TRIM73
ENSP00000211076	11	TPSD1
ENSP00000364396	11	TNXB
ENSP00000257663	11	TMEM60
ENSP00000297477	11	TMEM184A
ENSP00000261296	11	TGDS
ENSP00000364037	11	TEX10
ENSP00000347444	11	TDRD7
ENSP00000406293	11	TCEA3
ENSP00000266556	11	TAPBPL
ENSP00000340914	11	SYT3
ENSP00000225504	11	SUPT4H1
ENSP00000247001	11	SUGP1
ENSP00000242770	11	STX10
ENSP00000377717	11	ST3GAL6
ENSP00000216774	11	SRP54
ENSP00000316012	11	SPAG11A
ENSP00000311427	11	SNX33
ENSP00000342374	11	SNRPD2
ENSP00000354574	11	SLC9B2
ENSP00000254488	11	SLC6A11
ENSP00000327943	11	SLC5A2

ENSP00000254853	11	SLC52A1
ENSP00000356105	11	SLC41A1
ENSP00000269740	11	SLC39A3
ENSP00000235345	11	SLC35D1
ENSP00000239451	11	SLC25A2
ENSP00000291842	11	SHKBP1
ENSP00000345295	11	SH2D4B
ENSP00000305790	11	SF3B3
ENSP00000327436	11	SETD3
ENSP00000382779	11	SEH1L
ENSP00000254663	11	SCLY
ENSP00000347592	11	SAP30BP
ENSP00000326247	11	SAMD9L
ENSP00000282003	11	RNF219
ENSP00000373752	11	RNF180
ENSP00000334851	11	RGS7BP
ENSP00000354109	11	RGS22
ENSP00000306637	11	RGPD8
ENSP00000386810	11	RGPD4
ENSP00000270645	11	RCN3
ENSP00000199814	11	RBM22
ENSP00000259351	11	RALGPS1
ENSP00000349709	11	RAET1E
ENSP00000351832	11	RAB3GAP2
ENSP00000012049	11	QPCTL
ENSP00000326003	11	PUS10
ENSP00000311572	11	PTGR1
ENSP00000230582	11	PRSS16
ENSP00000415363	11	PRRC2A
ENSP00000333551	11	PROSC
ENSP00000264808	11	PRDM5
ENSP00000331065	11	PPP1R27
ENSP00000407310	11	PPP1R10
ENSP00000228705	11	PPM1H
ENSP00000389292	11	PPIL6
ENSP00000265627	11	PON3
ENSP00000295588	11	POGLUT1
ENSP00000393953	11	PNPT1
ENSP00000257694	11	PNPLA8
ENSP00000273077	11	PNKD
ENSP00000347038	11	PLSCR4
ENSP00000223864	11	PLGRKT
ENSP00000344961	11	PLEKHG7
ENSP00000272203	11	PLEKHA6
ENSP00000355503	11	PLD5
ENSP00000406909	11	PHOSPHO1

ENSP00000362807	11	PEF1
ENSP00000310661	11	PDE7B
ENSP00000360502	11	PDE6C
ENSP00000258229	11	PCNXL2
ENSP00000265260	11	PCNP
ENSP00000383168	11	PCBP3
ENSP00000367609	11	PABPN1L
ENSP00000386160	11	OR52B4
ENSP00000313922	11	OAZ3
ENSP00000332613	11	OAF
ENSP00000333593	11	NXPH4
ENSP00000258742	11	NUPL2
ENSP00000363431	11	NPY4R
ENSP00000261592	11	NOL4
ENSP00000264663	11	NNT
ENSP00000311687	11	NIPAL4
ENSP00000399309	11	NEU1
ENSP00000233627	11	NDUFS7
ENSP00000367346	11	NDUFAF5
ENSP00000296597	11	NDUFAF2
ENSP00000281513	11	NBAS
ENSP00000303584	11	N6AMT1
ENSP00000367049	11	MZT1
ENSP00000349145	11	MYO16
ENSP00000274643	11	MYLK4
ENSP00000418668	11	MURC
ENSP00000355084	11	MSRB1
ENSP00000308897	11	MRPL11
ENSP00000393251	11	MRGPRE
ENSP00000275053	11	MMS22L
ENSP00000309790	11	MIS18BP1
ENSP00000338706	11	MID1IP1
ENSP00000331664	11	MGAT4C
ENSP00000308546	11	MEPCE
ENSP00000348982	11	MEGF6
ENSP00000265245	11	LSG1
ENSP00000292616	11	LRWD1
ENSP00000340983	11	LRRC25
ENSP00000285737	11	LONP2
ENSP00000309463	11	LIPT2
ENSP00000383900	11	LIPK
ENSP00000302393	11	LDHAL6B
ENSP00000415464	11	LARP4
ENSP00000246646	11	KRT38
ENSP00000255992	11	KRBA1
ENSP00000273963	11	KLHL8

ENSP00000343273	11	KLHL7
ENSP00000296564	11	KIAA0947
ENSP00000374558	11	KIAA0226L
ENSP00000294725	11	KCNT2
ENSP00000376966	11	KCNC2
ENSP00000265272	11	JAKMIP2
ENSP00000362395	11	ITM2A
ENSP00000365351	11	ITGBL1
ENSP00000287996	11	IPPK
ENSP00000233331	11	INO80B
ENSP00000419781	11	IGLJ3
ENSP00000420436	11	IGKV1-5
ENSP00000417427	11	IGKV1-37
ENSP00000284110	11	HS3ST3A1
ENSP00000397867	11	HLA-C
ENSP00000295488	11	HELQ
ENSP00000311502	11	HEG1
ENSP00000317743	11	HARBI1
ENSP00000381666	11	GXYLT1
ENSP00000343428	11	GPR18
ENSP00000392859	11	GMPR2
ENSP00000301329	11	GLOD4
ENSP00000323075	11	GAPT
ENSP00000259056	11	GALNT5
ENSP00000336729	11	GALNT16
ENSP00000288988	11	GALNT14
ENSP00000291670	11	FTCD
ENSP00000360488	11	FRA10AC1
ENSP00000344393	11	FCF1
ENSP00000224862	11	FBXL15
ENSP00000331411	11	FBLN7
ENSP00000217429	11	FAM83D
ENSP00000353650	11	FABP12
ENSP00000364035	11	FAAH2
ENSP00000354125	11	EIF3B
ENSP00000382886	11	EHMT2
ENSP00000404232	11	EFHC2
ENSP00000318147	11	EDEM3
ENSP00000340609	11	DUPD1
ENSP00000195654	11	DOPEY1
ENSP00000383909	11	DMRTA2
ENSP00000255189	11	DMGDH
ENSP00000371813	11	DEFB132
ENSP00000371825	11	DEFB127
ENSP00000351147	11	DCAF4
ENSP00000339511	11	CXorf27

ENSP00000408204	11	CT47A9
ENSP00000392283	11	CT47A7
ENSP00000366178	11	CST9L
ENSP00000310891	11	CSGALNACT1
ENSP00000366557	11	CRNKL1
ENSP00000265394	11	CPVL
ENSP00000329748	11	CPNE8
ENSP00000247655	11	COX7C
ENSP00000263401	11	COMMD9
ENSP00000316605	11	CNGB3
ENSP00000328478	11	CNGA2
ENSP00000256722	11	CMPK2
ENSP00000229266	11	CHPT1
ENSP00000319851	11	CHDH
ENSP00000295767	11	CHCHD4
ENSP00000290567	11	CES5A
ENSP00000260662	11	CENPO
ENSP00000221554	11	CCDC130
ENSP00000346931	11	C6orf120
ENSP00000348302	11	C3orf27
ENSP00000295148	11	C2orf44
ENSP00000347298	11	C2orf27A
ENSP00000347712	11	C2CD4A
ENSP00000304364	11	C1GALT1C1
ENSP00000253110	11	C19orf66
ENSP00000358293	11	C10orf118
ENSP00000386121	11	BTBD9
ENSP00000280758	11	BTBD11
ENSP00000356432	11	BRINP3
ENSP00000369372	11	BEND2
ENSP00000343674	11	ASTL
ENSP00000384894	11	AQP12B
ENSP00000337144	11	AQP12A
ENSP00000261722	11	AP3B2
ENSP00000379891	11	AP1S3
ENSP00000328789	11	AP1S2
ENSP00000363395	11	ANXA8L2
ENSP00000352101	11	ANXA8L1
ENSP00000321617	11	ANKZF1
ENSP00000396747	11	ANKRD60
ENSP00000347802	11	ANKRD35
ENSP00000313513	11	ANKAR
ENSP00000281471	11	AMN1
ENSP00000299155	11	AMN
ENSP00000333813	11	ALG12
ENSP00000366927	11	ALDH1B1

ENSP00000293350	11	ALDH16A1
ENSP00000314036	11	AGPAT4
ENSP00000341662	11	AGMO
ENSP00000296721	11	AFAP1L1
ENSP00000310015	11	ADAT1
ENSP00000008311	11	-
ENSP00000058691	11	-
ENSP00000286603	11	-
ENSP00000306296	11	-
ENSP00000340625	11	-
ENSP00000351106	11	-
ENSP00000352838	11	-
ENSP00000363709	11	-
ENSP00000367361	11	-
ENSP00000368771	11	-
ENSP00000371882	11	-
ENSP00000381306	11	-
ENSP00000385120	11	-
ENSP00000397500	11	-
ENSP00000402909	11	-
ENSP00000408153	11	-
ENSP00000413404	11	-
ENSP00000413735	11	-
ENSP00000345140	10	ZNF746
ENSP00000292069	10	ZNF667
ENSP00000337320	10	ZNF664
ENSP00000300850	10	ZNF646
ENSP00000323348	10	ZNF645
ENSP00000301042	10	ZNF641
ENSP00000351939	10	ZNF574
ENSP00000361602	10	ZNF503
ENSP00000337473	10	ZNF324B
ENSP00000337475	10	ZDHHC4
ENSP00000361411	10	ZCCHC24
ENSP00000363972	10	ZC4H2
ENSP00000242351	10	ZC3HAV1
ENSP00000360147	10	XPNPEP2
ENSP00000356793	10	XCL2
ENSP00000386156	10	WIBG
ENSP00000362677	10	WDR38
ENSP00000361800	10	WDR34
ENSP00000325377	10	WDR33
ENSP00000261517	10	VPS13C
ENSP00000301962	10	USP42
ENSP00000219689	10	USP31
ENSP00000260323	10	UNC13C

ENSP00000354883	10	UBL7
ENSP00000351492	10	UBL5
ENSP00000340596	10	UBE3B
ENSP00000396152	10	UBD
ENSP00000331298	10	UBALD2
ENSP00000356903	10	UAP1
ENSP00000399155	10	TUBB
ENSP00000301364	10	TSR1
ENSP00000299550	10	TRIM66
ENSP00000320869	10	TRIM41
ENSP00000418697	10	TRGJP2
ENSP00000362381	10	TOR2A
ENSP00000330264	10	TMPRSS9
ENSP00000349087	10	TMEM259
ENSP00000340668	10	TMEM248
ENSP00000400311	10	TMEM240
ENSP00000333697	10	TMEM179B
ENSP00000211314	10	TMEM14A
ENSP00000256686	10	TMEM106C
ENSP00000288014	10	THTPA
ENSP00000327323	10	THNSL2
ENSP00000366036	10	TBC1D8
ENSP00000331867	10	TAS1R1
ENSP00000258975	10	TACO1
ENSP00000275200	10	TAAR8
ENSP00000324419	10	SYT9
ENSP00000363441	10	SYT15
ENSP00000418994	10	SYNPR
ENSP00000273666	10	STXBP5L
ENSP00000259400	10	STX17
ENSP00000364482	10	STK19
ENSP00000265304	10	SSBP1
ENSP00000342181	10	SRP72
ENSP00000274008	10	SPATA5
ENSP00000317123	10	SNRNP200
ENSP00000304707	10	SLN
ENSP00000348206	10	SLMO2
ENSP00000260649	10	SLC3A1
ENSP00000417654	10	SLC35A5
ENSP00000379836	10	SLC30A5
ENSP00000355893	10	SLC30A10
ENSP00000359526	10	SLC25A28
ENSP00000360549	10	SLC1A7
ENSP00000367137	10	SLC17A4
ENSP00000380250	10	SLC17A3
ENSP00000353677	10	SLC17A2

ENSP00000227880	10	SLC15A3
ENSP00000412310	10	SKIV2L
ENSP00000250360	10	SIGLEC9
ENSP00000315137	10	SGPP2
ENSP00000221494	10	SF3A2
ENSP00000210633	10	SEMA4G
ENSP00000268220	10	SEC11A
ENSP00000290216	10	SCRN2
ENSP00000420357	10	RWDD1
ENSP00000377942	10	RSPRY1
ENSP00000260443	10	RSL24D1
ENSP00000251453	10	RPS16
ENSP00000361076	10	RPL7A
ENSP00000307971	10	RFNG
ENSP00000326170	10	RFFL
ENSP00000265732	10	RBM48
ENSP00000384160	10	RBM33
ENSP00000265271	10	RBM27
ENSP00000313890	10	RBM15B
ENSP00000250237	10	QTRT1
ENSP00000363459	10	PTPN20A
ENSP00000392353	10	PSMG4
ENSP00000405932	10	PROX2
ENSP00000253329	10	PPIL4
ENSP00000388373	10	PPFIA2
ENSP00000221770	10	POP4
ENSP00000382058	10	POLR3G
ENSP00000263857	10	POLR1A
ENSP00000317177	10	PLD6
ENSP00000324403	10	PHF21B
ENSP00000360054	10	PHACTR3
ENSP00000239666	10	PDZD11
ENSP00000272227	10	PDIA6
ENSP00000311453	10	PDE8A
ENSP00000355082	10	PCDH18
ENSP00000264833	10	OLFM2
ENSP00000295119	10	NUP35
ENSP00000262302	10	NUBP2
ENSP00000355587	10	NTPCR
ENSP00000367752	10	NRSN1
ENSP00000353078	10	NRAP
ENSP00000410674	10	NOTCH4
ENSP00000283429	10	NMRAL1
ENSP00000356785	10	NME7
ENSP00000363894	10	NIPSNAP3B
ENSP00000260361	10	NDUFAF1

ENSP00000007516	10	NDUFAB1
ENSP00000261520	10	NARG2
ENSP00000362616	10	NAP1L2
ENSP00000273353	10	MYH15
ENSP00000263629	10	MTIF2
ENSP00000287025	10	MTERFD1
ENSP00000281047	10	MSGN1
ENSP00000016913	10	MS4A12
ENSP00000081029	10	MRPS35
ENSP00000260102	10	MRPL15
ENSP00000263268	10	MREG
ENSP00000266839	10	MMAB
ENSP00000350874	10	METTL9
ENSP00000384369	10	METTL15
ENSP00000376127	10	MAU2
ENSP00000305059	10	MAP1LC3B2
ENSP00000357453	10	MAN1A1
ENSP00000335385	10	MAGED4B
ENSP00000414006	10	LSM2
ENSP00000361007	10	LIPJ
ENSP00000293760	10	LEMD2
ENSP00000280706	10	LDHAL6A
ENSP00000328358	10	KRT79
ENSP00000337850	10	KPTN
ENSP00000156476	10	KLK13
ENSP00000314608	10	KLHL11
ENSP00000259708	10	KLC4
ENSP00000351207	10	KIAA1671
ENSP00000265068	10	KIAA1257
ENSP00000245121	10	KATNAL2
ENSP00000343763	10	IYD
ENSP00000298818	10	ISCA2
ENSP00000391457	10	INO80C
ENSP00000369574	10	IFNA21
ENSP00000277517	10	IDI2
ENSP00000291560	10	HSF2BP
ENSP00000216027	10	HSCB
ENSP00000298068	10	HECTD2
ENSP00000243706	10	HAUS3
ENSP00000230771	10	HARS2
ENSP00000344874	10	GUCY1A2
ENSP00000365504	10	GTF2H4
ENSP00000264952	10	GRK7
ENSP00000354581	10	GPX6
ENSP00000376384	10	GPR123
ENSP00000359686	10	GPR112

ENSP00000229314	10	GOLT1B
ENSP00000309653	10	GLIS1
ENSP00000367196	10	GLIPR2
ENSP00000396615	10	GLB1L3
ENSP00000295759	10	GLB1L
ENSP00000365550	10	GKAP1
ENSP00000326227	10	GANC
ENSP00000326948	10	FTSJ1
ENSP00000287474	10	FRRS1
ENSP00000399938	10	FLYWCH1
ENSP00000388861	10	FLOT1
ENSP00000413152	10	FKBP7
ENSP00000333836	10	FIGN
ENSP00000369666	10	FBXL19
ENSP00000329040	10	FAM174B
ENSP00000420174	10	ERVFRD-1
ENSP00000318982	10	EPSTI1
ENSP00000365840	10	ENTPD6
ENSP00000278505	10	ENDOD1
ENSP00000378254	10	EML3
ENSP00000248378	10	EMC6
ENSP00000359022	10	ELOVL3
ENSP00000252445	10	ELOF1
ENSP00000314810	10	EDDM3B
ENSP00000365838	10	DPRX
ENSP00000251076	10	DMXL2
ENSP00000328524	10	DENND5A
ENSP00000268854	10	DDX52
ENSP00000334625	10	DCUN1D4
ENSP00000355114	10	DCAF12
ENSP00000388320	10	CTU2
ENSP00000313759	10	CTC1
ENSP00000366170	10	CST9
ENSP00000228515	10	CSRNP2
ENSP00000220763	10	CPQ
ENSP00000319464	10	CPN2
ENSP00000359446	10	CPN1
ENSP00000378464	10	CPLX3
ENSP00000261070	10	COX17
ENSP00000016171	10	COX15
ENSP00000274458	10	COMMD10
ENSP00000384264	10	CNGA1
ENSP00000377747	10	CHD2
ENSP00000223208	10	CEP41
ENSP00000327191	10	CCT6B
ENSP00000334767	10	CCDC84

ENSP00000249064	10	CCDC117
ENSP00000364219	10	CAMK2N1
ENSP00000252729	10	CACNG6
ENSP00000290039	10	CACHD1
ENSP00000261669	10	CAB39L
ENSP00000365522	10	C9orf64
ENSP00000338885	10	C2CD2L
ENSP00000309561	10	C19orf70
ENSP00000302918	10	C11orf40
ENSP00000040738	10	BOD1L1
ENSP00000257899	10	BLOC1S1
ENSP00000369518	10	ATRAID
ENSP00000272238	10	ATP6V1C2
ENSP00000236067	10	ATP6V0B
ENSP00000306003	10	ATP5I
ENSP00000332756	10	ATP11C
ENSP00000283558	10	ATP11A
ENSP00000350786	10	ASIC4
ENSP00000360475	10	ARRDC1
ENSP00000368189	10	ARID3C
ENSP00000315915	10	ANXA2R
ENSP00000341013	10	AMTN
ENSP00000285518	10	AGPAT5
ENSP00000317578	10	ADRBK2
ENSP00000369921	10	ADAMTSL1
ENSP00000238651	10	ACOT2
ENSP00000360366	10	ACOT11
ENSP00000379568	10	ACBD5
ENSP00000373082	10	ABCF1
ENSP00000414373	10	ABCF1
ENSP00000260246	10	-
ENSP00000345153	10	-
ENSP00000354203	10	-
ENSP00000354217	10	-
ENSP00000364022	10	-
ENSP00000367228	10	-
ENSP00000367414	10	-
ENSP00000369215	10	-
ENSP00000380537	10	-
ENSP00000384049	10	-
ENSP00000394198	10	-
ENSP00000399924	10	-
ENSP00000402658	10	-
ENSP00000405398	10	-
ENSP00000412508	10	-
ENSP00000302502	9	ZSCAN32

ENSP00000350295	9	ZNF816
ENSP00000311768	9	ZNF706
ENSP00000333223	9	ZNF470
ENSP00000324064	9	ZNF354C
ENSP00000327143	9	ZNF354B
ENSP00000391067	9	ZNF302
ENSP00000400113	9	ZDHHC13
ENSP00000316794	9	ZCCHC5
ENSP00000237275	9	ZC2HC1B
ENSP00000216268	9	ZBED4
ENSP00000363746	9	WDR46
ENSP00000348848	9	WDR45
ENSP00000414721	9	WBP1L
ENSP00000225298	9	UTP18
ENSP00000282344	9	USP12
ENSP00000290868	9	UROC1
ENSP00000379217	9	UBFD1
ENSP00000292879	9	U2AF1L4
ENSP00000296015	9	TTC14
ENSP00000262067	9	TSPAN13
ENSP00000343398	9	TMPPE
ENSP00000406885	9	TMEM41A
ENSP00000388431	9	TMEM251
ENSP00000253047	9	TMEM160
ENSP00000368858	9	TMEM14B
ENSP00000380025	9	TMBIM1
ENSP00000361158	9	TESK2
ENSP00000287380	9	TBC1D31
ENSP00000310193	9	TBC1D10C
ENSP00000249284	9	TAS2R16
ENSP00000418379	9	TAF1L
ENSP00000228567	9	SYT10
ENSP00000340594	9	SYDE2
ENSP00000276646	9	SYBU
ENSP00000221283	9	STXBP2
ENSP00000401513	9	STRC
ENSP00000310969	9	STON1
ENSP00000296632	9	STARD4
ENSP00000312066	9	SRP68
ENSP00000333292	9	SPNS2
ENSP00000366690	9	SPATC1
ENSP00000305494	9	SPATA5L1
ENSP00000355900	9	SPATA17
ENSP00000357251	9	SOGA3
ENSP00000357836	9	SNX27
ENSP00000261369	9	SNX24

ENSP00000254193	9	SNRPA1
ENSP00000327467	9	SMDT1
ENSP00000350036	9	SMC1B
ENSP00000299977	9	SLFN5
ENSP00000356687	9	SLC9C2
ENSP00000331938	9	SLC9B1P1
ENSP00000296422	9	SLC9B1
ENSP00000301335	9	SLC43A2
ENSP00000313318	9	SLC35C1
ENSP00000348211	9	SLC25A46
ENSP00000289707	9	SLAMF8
ENSP00000298428	9	SEC61A2
ENSP00000308339	9	SEC16B
ENSP00000274938	9	SCUBE3
ENSP00000354080	9	SCUBE1
ENSP00000357301	9	RXFP4
ENSP00000023939	9	RTFDC1
ENSP00000216038	9	RTCB
ENSP00000360031	9	RRP12
ENSP00000323288	9	RPUSD2
ENSP00000196551	9	RPS5
ENSP00000359688	9	RPF1
ENSP00000352401	9	RPE
ENSP00000274134	9	ROPN1L
ENSP00000244360	9	RNF39
ENSP00000306906	9	RNF181
ENSP00000337623	9	RNASEH2B
ENSP00000302046	9	RNASE6
ENSP00000344904	9	RIC3
ENSP00000294413	9	RHCE
ENSP00000227474	9	PUS3
ENSP00000345168	9	PURG
ENSP00000333003	9	PRSS55
ENSP00000349856	9	PRRC2B
ENSP00000363313	9	PRPF4
ENSP00000367835	9	PRPF18
ENSP00000296682	9	PRDM9
ENSP00000416062	9	PPT2
ENSP00000292539	9	PPP1R16A
ENSP00000343943	9	PPOX
ENSP00000361465	9	POLR1C
ENSP00000263331	9	POLR1B
ENSP00000414352	9	PLEKHM1
ENSP00000361337	9	PLAC9
ENSP00000198536	9	PILRA
ENSP00000352782	9	PHOSPHO2

ENSP00000338868	9	PHF8
ENSP00000262764	9	PGS1
ENSP00000412189	9	PGAM4
ENSP00000263985	9	PET112
ENSP00000295992	9	PCOLCE2
ENSP00000358862	9	PCGF6
ENSP00000251654	9	PCCB
ENSP00000364190	9	PBX2
ENSP00000397177	9	PBX2
ENSP00000409279	9	OTUD4
ENSP00000215061	9	OCEL1
ENSP00000218004	9	NXT2
ENSP00000365015	9	NXNL2
ENSP00000371101	9	NOL10
ENSP00000413572	9	NLE1
ENSP00000387219	9	NFU1
ENSP00000229725	9	NEU1
ENSP00000364782	9	NEU1
ENSP00000401067	9	NEU1
ENSP00000403720	9	NEU1
ENSP00000408207	9	NEU1
ENSP00000409489	9	NEU1
ENSP00000233190	9	NDUFS1
ENSP00000410088	9	NCAPH2
ENSP00000281038	9	NARS2
ENSP00000281871	9	MZT2B
ENSP00000337222	9	MYADM
ENSP00000248643	9	MTERF
ENSP00000369682	9	MRPS26
ENSP00000417602	9	MRPL47
ENSP00000300151	9	MRPL16
ENSP00000306548	9	MRPL13
ENSP00000379669	9	MPV17L
ENSP00000250124	9	MPDU1
ENSP00000332646	9	MFSD10
ENSP00000359483	9	LRRC8C
ENSP00000261407	9	LPCAT3
ENSP00000383923	9	LIPN
ENSP00000383901	9	LIPM
ENSP00000301219	9	LILRA5
ENSP00000378116	9	LGALS12
ENSP00000301202	9	LAIR2
ENSP00000360676	9	KTI12
ENSP00000257974	9	KRT82
ENSP00000301656	9	KRT27
ENSP00000339356	9	KLRG2

ENSP00000325525	9	KIR3DL2
ENSP00000297591	9	KIAA1429
ENSP00000216039	9	JOSD1
ENSP00000173527	9	ISOC1
ENSP00000244314	9	IRGC
ENSP00000284202	9	IMPACT
ENSP00000419211	9	IGKV1-8
ENSP00000256433	9	IER3IP1
ENSP00000384474	9	HYPK
ENSP00000254878	9	HRSP12
ENSP00000386207	9	HMSD
ENSP00000372955	9	HMCES
ENSP00000393646	9	HLA-DMB
ENSP00000292432	9	HK3
ENSP00000230012	9	HDGFL1
ENSP00000260372	9	HAUS2
ENSP00000316339	9	HAO2
ENSP00000374268	9	GXYLT2
ENSP00000382670	9	GRXCR1
ENSP00000339057	9	GPRASP2
ENSP00000310255	9	GPR152
ENSP00000273352	9	GPR128
ENSP00000360299	9	GPR110
ENSP00000417354	9	GOLIM4
ENSP00000366233	9	GABBR1
ENSP00000406066	9	GABBR1
ENSP00000416731	9	GABBR1
ENSP00000252675	9	FUT5
ENSP00000269373	9	FN3KRP
ENSP00000376945	9	FBXW9
ENSP00000296438	9	FBXW12
ENSP00000342023	9	FBXO38
ENSP00000274680	9	FARS2
ENSP00000182377	9	FAR2
ENSP00000328426	9	FAM208B
ENSP00000289921	9	FAM160B2
ENSP00000296380	9	EXO5
ENSP00000316395	9	EPHA10
ENSP00000388000	9	EGFL8
ENSP00000355126	9	EGFL6
ENSP00000268206	9	EFTUD1
ENSP00000344758	9	ECM2
ENSP00000256039	9	DYDC2
ENSP00000303515	9	DUS1L
ENSP00000357384	9	DPM3
ENSP00000250937	9	DOHH

ENSP00000343665	9	DOC2B
ENSP00000374045	9	DNAH6
ENSP00000386770	9	DNAH10
ENSP00000360500	9	DMRTB1
ENSP00000284061	9	DGKE
ENSP00000362687	9	DDX50
ENSP00000352928	9	DDA1
ENSP00000259632	9	DCTN3
ENSP00000382281	9	DAXX
ENSP00000343074	9	CYP3A5
ENSP00000317159	9	CYC1
ENSP00000356218	9	CYB5R1
ENSP00000368758	9	CTXN3
ENSP00000362063	9	CSTF2
ENSP00000234301	9	COX7A2L
ENSP00000287490	9	COX6A2
ENSP00000342015	9	COX19
ENSP00000333946	9	COQ6
ENSP00000370984	9	COMMD8
ENSP00000267935	9	COMMD4
ENSP00000399013	9	CNTNAP5
ENSP00000331766	9	CLECL1
ENSP00000234488	9	CLCN6
ENSP00000284382	9	CERS3
ENSP00000353048	9	CCT8L2
ENSP00000346800	9	CCDC81
ENSP00000333374	9	CCDC60
ENSP00000240079	9	CCDC53
ENSP00000295989	9	CAND2
ENSP00000351684	9	CAMSAP2
ENSP00000169565	9	CACNG5
ENSP00000294288	9	CABP2
ENSP00000366030	9	C9orf41
ENSP00000362085	9	C9orf16
ENSP00000354553	9	C1orf85
ENSP00000287859	9	C1orf27
ENSP00000242784	9	C19orf43
ENSP00000326846	9	C14orf28
ENSP00000333041	9	C14orf180
ENSP00000377369	9	C14orf166B
ENSP00000359660	9	AVPI1
ENSP00000273398	9	ATP6V1A
ENSP00000285393	9	ATP6V0D2
ENSP00000262429	9	ATP2C2
ENSP00000310622	9	APOBEC4
ENSP00000360485	9	AKAP14

ENSP00000333019	9	ADSSL1
ENSP00000381036	9	ADGB
ENSP00000378161	9	ACYP2
ENSP00000346733	9	ACAP3
ENSP00000298992	9	ABTB2
ENSP00000295962	9	ABHD6
ENSP00000226840	9	AADAT
ENSP00000345676	9	1-Mar
ENSP00000021776	9	-
ENSP00000268795	9	-
ENSP00000306710	9	-
ENSP00000310075	9	-
ENSP00000348819	9	-
ENSP00000370288	9	-
ENSP00000373773	9	-
ENSP00000378477	9	-
ENSP00000383038	9	-
ENSP00000384147	9	-
ENSP00000389612	9	-
ENSP00000399380	9	-
ENSP00000410293	9	-
ENSP00000411131	9	-
ENSP00000411338	9	-
ENSP00000411971	9	-
ENSP00000294353	8	ZYG11B
ENSP00000252463	8	ZSCAN10
ENSP00000352836	8	ZNF675
ENSP00000293771	8	ZNF653
ENSP00000294753	8	ZNF496
ENSP00000313443	8	ZNF491
ENSP00000331577	8	ZNF397
ENSP00000302310	8	ZNF396
ENSP00000262990	8	ZNF330
ENSP00000415772	8	ZNF311
ENSP00000270014	8	ZNF155
ENSP00000352770	8	ZNF138
ENSP00000253048	8	ZC3H4
ENSP00000346466	8	WDR91
ENSP00000353699	8	WDR72
ENSP00000265758	8	WBSCR22
ENSP00000279281	8	VPS51
ENSP00000378426	8	VKORC1
ENSP00000414780	8	VCX3B
ENSP00000300896	8	USP32
ENSP00000333490	8	URAD
ENSP00000324343	8	UPB1

ENSP00000244565	8	UNC5CL
ENSP00000237186	8	UBE3D
ENSP00000398539	8	UBD
ENSP00000410416	8	UBD
ENSP00000354039	8	UBAP2
ENSP00000365714	8	TTYH1
ENSP00000347920	8	TTC39B
ENSP00000319486	8	TSPAN16
ENSP00000291416	8	TRIM62
ENSP00000391685	8	TRIM31
ENSP00000261180	8	TRHDE
ENSP00000339813	8	TPST2
ENSP00000341031	8	TPRG1
ENSP00000356966	8	TOMM40L
ENSP00000365290	8	TNF
ENSP00000372988	8	TNF
ENSP00000389490	8	TNF
ENSP00000389492	8	TNF
ENSP00000361311	8	TMEM53
ENSP00000363478	8	TMEM50A
ENSP00000341364	8	TMEM230
ENSP00000291934	8	TMEM190
ENSP00000330945	8	TMED9
ENSP00000404042	8	TMED4
ENSP00000315906	8	TIGD5
ENSP00000260619	8	THUMPD2
ENSP00000293970	8	TBC1D24
ENSP00000221543	8	TBC1D17
ENSP00000386538	8	TBC1D10B
ENSP00000040877	8	TARBP1
ENSP00000356908	8	TAAR2
ENSP00000371394	8	SYT8
ENSP00000319323	8	STOML1
ENSP00000391798	8	STK19
ENSP00000414302	8	SRSF12
ENSP00000261854	8	SPPL2A
ENSP00000358715	8	SORCS3
ENSP00000313121	8	SNX14
ENSP00000306627	8	SLC9C1
ENSP00000335292	8	SLC51B
ENSP00000405812	8	SLC4A8
ENSP00000326671	8	SLC47A2
ENSP00000395653	8	SLC46A1
ENSP00000327133	8	SLC35A4
ENSP00000359174	8	SLC35A3
ENSP00000354721	8	SLC26A1

ENSP00000321077	8	SIGLEC8
ENSP00000324245	8	SHROOM1
ENSP00000358742	8	SFR1
ENSP00000335024	8	SERPINA11
ENSP00000305956	8	SEPSECS
ENSP00000269389	8	SECTM1
ENSP00000307607	8	SDR16C5
ENSP00000272091	8	SDE2
ENSP00000370394	8	RUFY3
ENSP00000258955	8	RSAD1
ENSP00000232888	8	RRP9
ENSP00000228140	8	RPS13
ENSP00000259469	8	RPL35
ENSP00000287038	8	RPL30
ENSP00000402338	8	RPF2
ENSP00000371923	8	RP1L1
ENSP00000325794	8	RNF151
ENSP00000369162	8	RIOK1
ENSP00000390630	8	RIMBP3C
ENSP00000407925	8	RIMBP3B
ENSP00000391564	8	RIMBP3
ENSP00000362863	8	REEP3
ENSP00000370648	8	RDH14
ENSP00000413929	8	RCOR3
ENSP00000349783	8	RC3H2
ENSP00000339090	8	RBMX2
ENSP00000333001	8	RBM8A
ENSP00000342502	8	PYCR2
ENSP00000337331	8	PTDSS1
ENSP00000254630	8	PTCD3
ENSP00000268835	8	PRPSAP2
ENSP00000318520	8	PROM2
ENSP00000301039	8	PROCA1
ENSP00000351686	8	PRDM10
ENSP00000381302	8	PPAPDC1A
ENSP00000347345	8	POLR3H
ENSP00000330031	8	PIWIL3
ENSP00000262265	8	PIH1D1
ENSP00000332313	8	PIGW
ENSP00000252813	8	PGPEP1
ENSP00000288050	8	PDPR
ENSP00000246535	8	PDCD2L
ENSP00000362456	8	PBDC1
ENSP00000265174	8	PAPSS1
ENSP00000322486	8	OTOS
ENSP00000334418	8	OR4C12

ENSP00000358967	8	OPN1LW
ENSP00000322273	8	OLFML3
ENSP00000374237	8	NXPE2
ENSP00000348831	8	NUDT10
ENSP00000352904	8	NT5C1B
ENSP00000337501	8	NRIP2
ENSP00000264279	8	NOP58
ENSP00000307525	8	NMD3
ENSP00000307449	8	NECAB2
ENSP00000257829	8	NAT10
ENSP00000401508	8	NAPRT1
ENSP00000326424	8	NADSYN1
ENSP00000274605	8	N4BP3
ENSP00000249442	8	MTX2
ENSP00000263063	8	MTPAP
ENSP00000359859	8	MTG2
ENSP00000380531	8	MRPS34
ENSP00000312395	8	MRPS21
ENSP00000397790	8	MRPS18B
ENSP00000229238	8	MRPL51
ENSP00000377486	8	MRPL19
ENSP00000333837	8	MRPL12
ENSP00000271373	8	MPC2
ENSP00000377796	8	METTL7B
ENSP00000371345	8	METTL22
ENSP00000296411	8	METAP1
ENSP00000362457	8	MAGEE2
ENSP00000364992	8	LY6G6F
ENSP00000406832	8	LY6G6D
ENSP00000359990	8	LRRC40
ENSP00000344364	8	LRRC29
ENSP00000355166	8	LRRC10
ENSP00000332681	8	LRP2BP
ENSP00000287585	8	LHFPL4
ENSP00000412803	8	LEPROTL1
ENSP00000307214	8	LCMT2
ENSP00000326128	8	LARP4B
ENSP00000264170	8	KYNU
ENSP00000330101	8	KRT76
ENSP00000377616	8	KRT222
ENSP00000279544	8	KLRF1
ENSP00000356990	8	KLHDC9
ENSP00000352282	8	KLHDC1
ENSP00000335388	8	KIR3DX1
ENSP00000363232	8	KIF12
ENSP00000220244	8	KIAA1199

ENSP00000251993	8	KIAA0930
ENSP00000318406	8	KIAA0319L
ENSP00000318016	8	KIAA0196
ENSP00000263372	8	KCNK6
ENSP00000282146	8	KCNK13
ENSP00000310568	8	KCNK10
ENSP00000395323	8	KCNIP1
ENSP00000281830	8	KCNE4
ENSP00000361173	8	KCNE1L
ENSP00000357008	8	ITLN2
ENSP00000357881	8	IKZF5
ENSP00000301585	8	ICT1
ENSP00000219439	8	HSDL1
ENSP00000399168	8	HLA-B
ENSP00000370555	8	GYG2
ENSP00000329558	8	GRPEL2
ENSP00000355799	8	GPR31
ENSP00000362153	8	GNL2
ENSP00000389175	8	GLYCTK
ENSP00000361110	8	GBGT1
ENSP00000287957	8	GATAD1
ENSP00000280236	8	FSIP1
ENSP00000335651	8	FSD2
ENSP00000303508	8	FRMD3
ENSP00000237172	8	FILIP1
ENSP00000321962	8	FIBIN
ENSP00000381982	8	FER1L6
ENSP00000399259	8	FCHSD1
ENSP00000418111	8	FBXL17
ENSP00000265978	8	FAM160A2
ENSP00000364814	8	FAM131C
ENSP00000419235	8	FAM115A
ENSP00000372112	8	FAHD1
ENSP00000368984	8	EXOSC9
ENSP00000361433	8	EXOSC2
ENSP00000346173	8	ETFB
ENSP00000262455	8	ERP44
ENSP00000361331	8	ERI3
ENSP00000339861	8	ENY2
ENSP00000256545	8	EMC7
ENSP00000216190	8	EIF3D
ENSP00000312671	8	EHBP1L1
ENSP00000380054	8	EAF1
ENSP00000356130	8	DSTYK
ENSP00000295066	8	DPY30
ENSP00000382104	8	DOPEY2

ENSP00000216068	8	DNAL4
ENSP00000320548	8	DNAJC4
ENSP00000345575	8	DNAJB12
ENSP00000301180	8	DIP2B
ENSP00000311135	8	DHX37
ENSP00000248879	8	DGCR6L
ENSP00000334364	8	DEFB106B
ENSP00000332340	8	DDX28
ENSP00000318227	8	DCAF8
ENSP00000364404	8	DCAF17
ENSP00000334128	8	CYP27C1
ENSP00000363624	8	CUTA
ENSP00000332018	8	CTAG1A
ENSP00000291440	8	CPAMD8
ENSP00000261643	8	COX10
ENSP00000254759	8	COQ3
ENSP00000325002	8	COPG1
ENSP00000278980	8	COMMD7
ENSP00000305459	8	COG8
ENSP00000361926	8	CNPY3
ENSP00000317439	8	CNPY1
ENSP00000410396	8	CMSS1
ENSP00000344392	8	CLUAP1
ENSP00000299665	8	CLEC4D
ENSP00000388457	8	CLDND1
ENSP00000318113	8	CLDN22
ENSP00000356133	8	CLDN20
ENSP00000332771	8	CLCNKA
ENSP00000309270	8	CHST1
ENSP00000354730	8	CHAMP1
ENSP00000342510	8	CEP97
ENSP00000404151	8	CEP170B
ENSP00000373023	8	CDSN
ENSP00000339390	8	CDH26
ENSP00000224756	8	CCSER2
ENSP00000260054	8	CCDC90B
ENSP00000347586	8	CCDC69
ENSP00000341684	8	CCDC15
ENSP00000259229	8	CCDC115
ENSP00000326238	8	CAPZA3
ENSP00000222212	8	CACNG7
ENSP00000369431	8	CAAP1
ENSP00000352704	8	C20orf112
ENSP00000354874	8	C17orf47
ENSP00000261250	8	C12orf4
ENSP00000328698	8	C10orf107

ENSP00000258761	8	BZW2
ENSP00000358146	8	BOLA1
ENSP00000266383	8	B4GALNT3
ENSP00000319778	8	AURKAIP1
ENSP00000366748	8	AUP1
ENSP00000249289	8	ATP6V1F
ENSP00000379203	8	ATP6V1C1
ENSP00000358737	8	ATP5F1
ENSP00000273859	8	ATP10D
ENSP00000313600	8	ATP10B
ENSP00000259477	8	ARPC5L
ENSP00000219204	8	ARL2BP
ENSP00000414757	8	ARL13A
ENSP00000318355	8	AQP10
ENSP00000372930	8	APOM
ENSP00000389591	8	APOM
ENSP00000394610	8	APOM
ENSP00000398944	8	APOM
ENSP00000401684	8	APOM
ENSP00000405730	8	APOM
ENSP00000265224	8	ANKRD7
ENSP00000386502	8	ANKRD61
ENSP00000350686	8	ANKLE2
ENSP00000352976	8	AMZ2
ENSP00000231420	8	AGXT2
ENSP00000412805	8	AGPAT1
ENSP00000364986	8	AGMAT
ENSP00000364217	8	AGER
ENSP00000265707	8	ADAM18
ENSP00000336842	8	ACTR8
ENSP00000379282	8	ABHD16A
ENSP00000326491	8	ABHD1
ENSP00000215591	8	-
ENSP00000292369	8	-
ENSP00000296111	8	-
ENSP00000298715	8	-
ENSP00000316222	8	-
ENSP00000326763	8	-
ENSP00000330714	8	-
ENSP00000330965	8	-
ENSP00000343880	8	-
ENSP00000346324	8	-
ENSP00000362200	8	-
ENSP00000364429	8	-
ENSP00000368839	8	-
ENSP00000373156	8	-

ENSP00000374954	8	-
ENSP00000382113	8	-
ENSP00000388779	8	-
ENSP00000390826	8	-
ENSP00000391307	8	-
ENSP00000393642	8	-
ENSP00000395419	8	-
ENSP00000397637	8	-
ENSP00000401283	8	-
ENSP00000402325	8	-
ENSP00000408234	8	-
ENSP00000411441	8	-
ENSP00000352614	7	ZSWIM5
ENSP00000329738	7	ZSCAN30
ENSP00000282326	7	ZSCAN1
ENSP00000341151	7	ZNF81
ENSP00000299927	7	ZNF592
ENSP00000313258	7	ZNF541
ENSP00000336565	7	ZNF446
ENSP00000367300	7	ZNF425
ENSP00000262085	7	ZNF282
ENSP00000291182	7	ZNF235
ENSP00000219091	7	ZNF205
ENSP00000347755	7	ZNF140
ENSP00000385939	7	ZNF12
ENSP00000363545	7	ZDBF2
ENSP00000346603	7	ZBTB45
ENSP00000288828	7	WIPI2
ENSP00000278856	7	WDR74
ENSP00000265107	7	WDR70
ENSP00000334148	7	WDR25
ENSP00000280190	7	WDR17
ENSP00000329654	7	WBSCR17
ENSP00000363869	7	VSIG4
ENSP00000285199	7	USP43
ENSP00000258243	7	URB2
ENSP00000207549	7	UNC13D
ENSP00000373413	7	UFSP1
ENSP00000281741	7	TXNDC16
ENSP00000283033	7	TXNDC11
ENSP00000351206	7	TXLNB
ENSP00000256367	7	TTC9
ENSP00000323584	7	TSHZ1
ENSP00000347161	7	TSGA10
ENSP00000298746	7	TRUB1
ENSP00000312356	7	TRMT10C

ENSP00000273962	7	TRMT10A
ENSP00000355437	7	TRIM58
ENSP00000290942	7	TPPP3
ENSP00000259339	7	TOR1B
ENSP00000261556	7	TMEM260
ENSP00000414899	7	TMEM241
ENSP00000356211	7	TMEM183A
ENSP00000233047	7	TMEM159
ENSP00000334708	7	TMEM150A
ENSP00000351380	7	TMA16
ENSP00000400500	7	THAP5
ENSP00000385006	7	THAP4
ENSP00000389466	7	TGM7
ENSP00000341346	7	TEKT1
ENSP00000331302	7	TCEB3B
ENSP00000407195	7	TAPBP
ENSP00000325564	7	SYCN
ENSP00000310814	7	STARD6
ENSP00000355273	7	ST6GAL2
ENSP00000362900	7	SRSF4
ENSP00000367139	7	SPINK9
ENSP00000348314	7	SPEF2
ENSP00000359561	7	SPANXB1
ENSP00000367965	7	SOWAHA
ENSP00000404438	7	SOHLH1
ENSP00000374152	7	SMYD5
ENSP00000336627	7	SLITRK4
ENSP00000309741	7	SLCO4C1
ENSP00000320378	7	SLC7A8
ENSP00000285850	7	SLC7A7
ENSP00000231706	7	SLC7A14
ENSP00000323549	7	SLC6A18
ENSP00000338627	7	SLC6A16
ENSP00000398852	7	SLC44A4
ENSP00000255559	7	SLC39A11
ENSP00000334223	7	SLC36A2
ENSP00000264128	7	SLC25A24
ENSP00000329452	7	SLC25A21
ENSP00000406546	7	SLC23A3
ENSP00000343022	7	SLC16A12
ENSP00000355428	7	SH3BP5L
ENSP00000316007	7	SCAMP4
ENSP00000386348	7	SAPCD2
ENSP00000362574	7	S100PBP
ENSP00000254605	7	RRP8
ENSP00000007264	7	RPUSD1

ENSP00000379888	7	RPS8
ENSP00000414321	7	RPS24
ENSP00000309830	7	RPL38
ENSP00000418082	7	RPL37A
ENSP00000346001	7	RPL3
ENSP00000306080	7	RNMTL1
ENSP00000367173	7	RNF207
ENSP00000320508	7	RNF185
ENSP00000308193	7	RNASEH2C
ENSP00000307096	7	RNASE4
ENSP00000283109	7	RIOK2
ENSP00000360112	7	RAVER2
ENSP00000259569	7	RANBP6
ENSP00000376440	7	RAD9B
ENSP00000359607	7	PYROXD2
ENSP00000348722	7	PUS7
ENSP00000389875	7	PSORS1C1
ENSP00000327386	7	PRSS57
ENSP00000331845	7	PRR14L
ENSP00000361512	7	PRPS1
ENSP00000343190	7	PPCDC
ENSP00000253107	7	PPAN
ENSP00000351695	7	PNLIPRP1
ENSP00000341412	7	PLET1
ENSP00000351318	7	PLEKHG1
ENSP00000385892	7	PLEKHB2
ENSP00000409637	7	PLCL2
ENSP00000322218	7	PITPNM2
ENSP00000420037	7	PIGP
ENSP00000258324	7	PIGC
ENSP00000358617	7	PHTF1
ENSP00000216252	7	PHF5A
ENSP00000225873	7	PEX12
ENSP00000295645	7	PDCL2
ENSP00000217073	7	PABPC1L
ENSP00000296358	7	OTOP1
ENSP00000332576	7	OSBP2
ENSP00000243045	7	ORMDL2
ENSP00000327585	7	OR1D2
ENSP00000311477	7	OR10AG1
ENSP00000369157	7	OR10A4
ENSP00000284719	7	OLA1
ENSP00000379401	7	ODAM
ENSP00000416829	7	OARD1
ENSP00000338352	7	NUDT4
ENSP00000376615	7	NT5DC3

ENSP00000355077	7	NSMCE1
ENSP00000296802	7	NSA2
ENSP00000348395	7	NPEPL1
ENSP00000333735	7	NPAP1
ENSP00000264230	7	NOA1
ENSP00000299481	7	NLRP14
ENSP00000285814	7	NIFK
ENSP00000274606	7	NHP2
ENSP00000246190	7	NECAB3
ENSP00000268668	7	NDUFB10
ENSP00000266544	7	NDUFA9
ENSP00000403683	7	NABP1
ENSP00000303920	7	MZB1
ENSP00000325402	7	MYPOP
ENSP00000258787	7	MYO1G
ENSP00000354728	7	MT-ND4L
ENSP00000315878	7	MS4A6A
ENSP00000285298	7	MRPS17
ENSP00000351506	7	MRPL42
ENSP00000244230	7	MPHOSPH10
ENSP00000347486	7	MORN3
ENSP00000215862	7	MORC2
ENSP00000361097	7	MMRN2
ENSP00000338487	7	MGAT4B
ENSP00000373004	7	MCCD1
ENSP00000343657	7	MCCC2
ENSP00000265594	7	MCCC1
ENSP00000353655	7	MAN2A2
ENSP00000360645	7	MAN1B1
ENSP00000419370	7	MALSU1
ENSP00000350592	7	MAGEA12
ENSP00000293274	7	LYZL6
ENSP00000328737	7	LYPD4
ENSP00000355187	7	LRRTM3
ENSP00000296144	7	LRRC2
ENSP00000334375	7	LRCH3
ENSP00000342071	7	LIPT1
ENSP00000276590	7	LACTB2
ENSP00000267436	7	L2HGDH
ENSP00000222307	7	KXD1
ENSP00000378292	7	KRT80
ENSP00000249776	7	KNSTRN
ENSP00000328494	7	KIF21B
ENSP00000374376	7	KIAA1549L
ENSP00000218176	7	KCND1
ENSP00000388723	7	KBTBD13

ENSP00000333350	7	JRKL
ENSP00000378451	7	JADE2
ENSP00000310623	7	ISCU
ENSP00000365159	7	ISCA1
ENSP00000259239	7	IMP4
ENSP00000268389	7	IGSF6
ENSP00000277124	7	IDNK
ENSP00000373640	7	HYKK
ENSP00000223026	7	HYAL4
ENSP00000305919	7	HTRA4
ENSP00000336984	7	HORMAD2
ENSP00000387088	7	HNRNPLL
ENSP00000410857	7	HLA-DRB4
ENSP00000409159	7	HLA-DQB2
ENSP00000382018	7	HLA-DQB1
ENSP00000229633	7	HINT3
ENSP00000291481	7	HAPLN4
ENSP00000243297	7	H2BFM
ENSP00000338290	7	GZF1
ENSP00000324570	7	GYLTL1B
ENSP00000296734	7	GPX8
ENSP00000332900	7	GPR97
ENSP00000366628	7	GPR157
ENSP00000335156	7	GPR144
ENSP00000283303	7	GPR115
ENSP00000381727	7	GPR111
ENSP00000156109	7	GPKOW
ENSP00000266014	7	GLT8D1
ENSP00000337972	7	GDPD5
ENSP00000284116	7	GDPD1
ENSP00000394008	7	GABARAPL3
ENSP00000344579	7	FSCB
ENSP00000366995	7	FRMPD1
ENSP00000365569	7	FLOT1
ENSP00000354421	7	FICD
ENSP00000379026	7	FBXW10
ENSP00000264669	7	FASTKD3
ENSP00000400513	7	FASTKD1
ENSP00000238823	7	FAM98A
ENSP00000347744	7	FAM49A
ENSP00000295569	7	FAM19A4
ENSP00000335808	7	FAM192A
ENSP00000385893	7	FAM161A
ENSP00000395249	7	FAM134A
ENSP00000272610	7	FAHD2B
ENSP00000356382	7	F13B

ENSP00000301825	7	ENTPD3
ENSP00000323714	7	EIF4E1B
ENSP00000414377	7	EHMT2
ENSP00000301729	7	ECI1
ENSP00000361276	7	DYDC1
ENSP00000265720	7	DUS4L
ENSP00000227451	7	DTX4
ENSP00000268793	7	DPEP3
ENSP00000310360	7	DNAL1
ENSP00000320303	7	DNAJC28
ENSP00000311273	7	DNAH7
ENSP00000373825	7	DNAH2
ENSP00000334801	7	DHRS4L2
ENSP00000354597	7	DENND4B
ENSP00000400326	7	DDX39B
ENSP00000417706	7	DCUN1D2
ENSP00000349822	7	DBNDD2
ENSP00000345317	7	CXCL17
ENSP00000345412	7	CPSF7
ENSP00000297770	7	CPA6
ENSP00000272995	7	COPS7B
ENSP00000390661	7	CLEC2L
ENSP00000380581	7	CLEC17A
ENSP00000286808	7	CLDN17
ENSP00000327179	7	CIRH1A
ENSP00000281882	7	CFC1B
ENSP00000355089	7	CELF4
ENSP00000338369	7	CELA3B
ENSP00000262659	7	CCM2L
ENSP00000225726	7	CCDC47
ENSP00000330240	7	CCDC34
ENSP00000255784	7	CCDC134
ENSP00000260508	7	CCBL2
ENSP00000341006	7	CATSPER4
ENSP00000318912	7	CA13
ENSP00000330361	7	C8orf33
ENSP00000373024	7	C6orf15
ENSP00000335041	7	C2orf76
ENSP00000284881	7	C21orf91
ENSP00000299367	7	C2
ENSP00000419417	7	C1orf52
ENSP00000272139	7	C1orf35
ENSP00000294360	7	C1orf123
ENSP00000351422	7	C19orf60
ENSP00000282059	7	C18orf25
ENSP00000382157	7	C14orf182

ENSP00000329361	7	BTBD6
ENSP00000395461	7	BTBD19
ENSP00000343742	7	BROX
ENSP00000363642	7	BMS1
ENSP00000358886	7	ATXN7L2
ENSP00000406372	7	ATP6V1G2
ENSP00000363162	7	ATP6V1G1
ENSP00000326340	7	ATG16L2
ENSP00000269197	7	ASXL3
ENSP00000284142	7	ASB17
ENSP00000401369	7	ASB10
ENSP00000269932	7	ARMC6
ENSP00000298694	7	ARHGEF40
ENSP00000362442	7	ARHGAP40
ENSP00000384753	7	ANKLE1
ENSP00000369579	7	ANKDD1A
ENSP00000262844	7	AMMECR1
ENSP00000266581	7	AMIGO2
ENSP00000291572	7	AGPAT3
ENSP00000337463	7	AGPAT1
ENSP00000382723	7	AGPAT1
ENSP00000300176	7	AGFG2
ENSP00000365725	7	ABHD12
ENSP00000342216	7	ABCA9
ENSP00000263094	7	ABCA7
ENSP00000299698	7	A2ML1
ENSP00000251473	7	-
ENSP00000252206	7	-
ENSP00000257034	7	-
ENSP00000264597	7	-
ENSP00000270637	7	-
ENSP00000310493	7	-
ENSP00000318090	7	-
ENSP00000337629	7	-
ENSP00000349402	7	-
ENSP00000352962	7	-
ENSP00000353780	7	-
ENSP00000365382	7	-
ENSP00000373694	7	-
ENSP00000375181	7	-
ENSP00000376118	7	-
ENSP00000387653	7	-
ENSP00000388441	7	-
ENSP00000393672	7	-
ENSP00000394529	7	-
ENSP00000395393	7	-

ENSP00000398748	7	-
ENSP00000403878	7	-
ENSP00000404970	7	-
ENSP00000406309	7	-
ENSP00000406696	7	-
ENSP00000407089	7	-
ENSP00000410815	7	-
ENSP00000252797	6	ZNF764
ENSP00000316527	6	ZNF609
ENSP00000345479	6	ZNF587
ENSP00000292530	6	ZNF333
ENSP00000397178	6	ZNF300
ENSP00000404403	6	ZBTB22
ENSP00000359364	6	YTHDF1
ENSP00000328326	6	XKR4
ENSP00000286049	6	XAGE2B
ENSP00000363308	6	WDR31
ENSP00000333799	6	WBSCR16
ENSP00000217957	6	VSIG1
ENSP00000369810	6	VPS16
ENSP00000328397	6	VMO1
ENSP00000250823	6	VCY1B
ENSP00000415316	6	VARS
ENSP00000261637	6	UTP20
ENSP00000414922	6	USP41
ENSP00000303434	6	USP38
ENSP00000333329	6	USP17L2
ENSP00000412922	6	URM1
ENSP00000367934	6	UQCRQ
ENSP00000287022	6	UQCRB
ENSP00000391088	6	UNC80
ENSP00000359698	6	UBTD1
ENSP00000296786	6	UBLCP1
ENSP00000272930	6	UBE2F
ENSP00000373066	6	TXNRD3NB
ENSP00000363634	6	TXNDC8
ENSP00000301819	6	TTC21A
ENSP00000364293	6	TSR2
ENSP00000303437	6	TSNARE1
ENSP00000362936	6	TRNAU1AP
ENSP00000374399	6	TRMT61A
ENSP00000309433	6	TRMT112
ENSP00000343990	6	TRIM14
ENSP00000313704	6	TPRN
ENSP00000347748	6	TPRA1
ENSP00000356584	6	TOR1AIP2

ENSP00000351214	6	TOMM7
ENSP00000299608	6	TMX3
ENSP00000335261	6	TMLHE
ENSP00000374484	6	TMEM87A
ENSP00000362021	6	TMEM35
ENSP00000293261	6	TMEM143
ENSP00000306887	6	TMEM126A
ENSP00000352413	6	TMC3
ENSP00000258412	6	TMBIM1
ENSP00000305852	6	TM4SF4
ENSP00000366854	6	THAP3
ENSP00000272438	6	TEX261
ENSP00000256246	6	TEX15
ENSP00000387303	6	TDRD9
ENSP00000294848	6	TDRD5
ENSP00000196169	6	TDRD3
ENSP00000328232	6	TCEB3C
ENSP00000284259	6	TBCEL
ENSP00000341652	6	TBCCD1
ENSP00000402935	6	TBC1D5
ENSP00000364207	6	TBC1D2
ENSP00000355941	6	TATDN3
ENSP00000347538	6	SYT17
ENSP00000418901	6	SYT14
ENSP00000312318	6	SSSCA1
ENSP00000265044	6	SSR3
ENSP00000338034	6	SPRYD4
ENSP00000332163	6	SPRR4
ENSP00000324870	6	SPINK6
ENSP00000372482	6	SNRNP25
ENSP00000355721	6	SNAP47
ENSP00000298966	6	SMCO4
ENSP00000241274	6	SLITRK3
ENSP00000238688	6	SLIRP
ENSP00000337561	6	SLC43A3
ENSP00000273173	6	SLC22A14
ENSP00000316202	6	SLC13A2
ENSP00000348596	6	SDAD1
ENSP00000244496	6	RRP36
ENSP00000355899	6	RRP15
ENSP00000296674	6	RPS23
ENSP00000346045	6	RPS17
ENSP00000311430	6	RPL4
ENSP00000268661	6	RPL3L
ENSP00000307889	6	RPL13
ENSP00000270357	6	RNPEPL1

ENSP00000326095	6	RNF166
ENSP00000280571	6	RILPL2
ENSP00000016946	6	RGPD5
ENSP00000254901	6	REEP2
ENSP00000295971	6	RBM47
ENSP00000223073	6	RBM28
ENSP00000368341	6	RBM24
ENSP00000362639	6	RABEPK
ENSP00000252329	6	PSMG3
ENSP00000363993	6	PSMB9
ENSP00000406878	6	PSMB8
ENSP00000372067	6	PRMT8
ENSP00000305924	6	PPP4R4
ENSP00000274853	6	PPP1R18
ENSP00000340610	6	POTEH
ENSP00000386971	6	POTEG
ENSP00000304353	6	POP7
ENSP00000331258	6	POMK
ENSP00000342889	6	POLR2K
ENSP00000364956	6	PLEKHM2
ENSP00000330278	6	PLEKHH1
ENSP00000225609	6	PIGL
ENSP00000368199	6	PGPEP1L
ENSP00000356989	6	PFDN2
ENSP00000359234	6	PDZD7
ENSP00000309548	6	PDP2
ENSP00000361486	6	PCIF1
ENSP00000337500	6	PCGF5
ENSP00000231134	6	PCDHB5
ENSP00000261888	6	PARP16
ENSP00000384700	6	PAPOLB
ENSP00000373610	6	OTOA
ENSP00000315410	6	OSBPL3
ENSP00000380153	6	OR51E2
ENSP00000313803	6	OR3A1
ENSP00000376633	6	OR2F1
ENSP00000335575	6	OR1J2
ENSP00000279028	6	OCSTAMP
ENSP00000264312	6	OCIAD1
ENSP00000345895	6	NUP50
ENSP00000349874	6	NUDT13
ENSP00000304854	6	NUDCD2
ENSP00000361558	6	NTMT1
ENSP00000409285	6	NRM
ENSP00000405348	6	NPIPB7
ENSP00000332198	6	NOP10

ENSP00000370883	6	NOMO2
ENSP00000328854	6	NOC4L
ENSP00000380109	6	NFKBID
ENSP00000276689	6	NDUFB9
ENSP00000247866	6	NDUFB2
ENSP00000358272	6	NDUFAF4
ENSP00000356202	6	MTRF1L
ENSP00000368790	6	MTRF1
ENSP00000364320	6	MRTO4
ENSP00000258455	6	MRPS9
ENSP00000261413	6	MRPS27
ENSP00000320184	6	MRPS23
ENSP00000241600	6	MRPS2
ENSP00000339844	6	MRPL43
ENSP00000199706	6	MRPL28
ENSP00000354525	6	MRPL24
ENSP00000411177	6	MRPL22
ENSP00000366095	6	MOG
ENSP00000390785	6	MOG
ENSP00000395005	6	MOG
ENSP00000398412	6	MICB
ENSP00000331787	6	METTL7A
ENSP00000373474	6	METTL14
ENSP00000315152	6	METAP1D
ENSP00000382178	6	MDGA2
ENSP00000354649	6	MAGT1
ENSP00000368264	6	MAGEB1
ENSP00000287748	6	LYZL4
ENSP00000364467	6	LYZL2
ENSP00000302160	6	LSM3
ENSP00000361177	6	LRIT1
ENSP00000317300	6	LPCAT4
ENSP00000216080	6	LMF2
ENSP00000371548	6	LGI2
ENSP00000297720	6	LETM2
ENSP00000277526	6	LCN9
ENSP00000307240	6	KRT74
ENSP00000293303	6	KLHL10
ENSP00000334140	6	KLHDC10
ENSP00000291860	6	KIR3DL3
ENSP00000368464	6	KIF24
ENSP00000305702	6	KCTD19
ENSP00000302719	6	KCNAB3
ENSP00000386711	6	JAKMIP1
ENSP00000384198	6	INO80D
ENSP00000263383	6	ILVBL

ENSP00000357073	6	IGSF9
ENSP00000332773	6	IGDCC3
ENSP00000369566	6	IFNA10
ENSP00000370293	6	HUS1B
ENSP00000354213	6	HS3ST3B1
ENSP00000002596	6	HS3ST1
ENSP00000361935	6	HPCAL4
ENSP00000353472	6	HLA-G
ENSP00000302517	6	HLA-DRB3
ENSP00000279392	6	HIRIP3
ENSP00000274787	6	HIGD2A
ENSP00000359978	6	HHLA3
ENSP00000248089	6	HCFC1R1
ENSP00000206474	6	HAUS4
ENSP00000373176	6	GTPBP8
ENSP00000369344	6	GPR150
ENSP00000307831	6	GPR113
ENSP00000271732	6	GOLPH3L
ENSP00000248572	6	GNGT1
ENSP00000309092	6	GMPPB
ENSP00000406164	6	GMNC
ENSP00000270257	6	GEMIN7
ENSP00000363503	6	GDPD2
ENSP00000226796	6	GAR1
ENSP00000272252	6	GALM
ENSP00000379766	6	GALK2
ENSP00000361932	6	FUT11
ENSP00000288078	6	FUK
ENSP00000265825	6	FSCN3
ENSP00000263578	6	FOXRED1
ENSP00000346560	6	FILIP1L
ENSP00000349356	6	FIGNL1
ENSP00000259989	6	FGFBP2
ENSP00000360871	6	FCN1
ENSP00000371805	6	FBXL18
ENSP00000322926	6	FARP1
ENSP00000335082	6	FAM91A1
ENSP00000239906	6	FAM53C
ENSP00000369625	6	FAM50B
ENSP00000237642	6	FAM47E-STBD1
ENSP00000354688	6	FAM45A
ENSP00000372290	6	FAM193A
ENSP00000373808	6	FAM169A
ENSP00000373726	6	FAM159B
ENSP00000221233	6	EXOSC5
ENSP00000252482	6	EXOC3L2

ENSP00000266397	6	ERP27
ENSP00000350651	6	ERI2
ENSP00000349970	6	ERGIC3
ENSP00000318066	6	ENPP4
ENSP00000382675	6	ENHO
ENSP00000303779	6	EME2
ENSP00000393324	6	EIF4E3
ENSP00000363616	6	EDEM2
ENSP00000262547	6	DZANK1
ENSP00000311977	6	DUS3L
ENSP00000359127	6	DPH5
ENSP00000419599	6	DPH3
ENSP00000379279	6	DNAJC24
ENSP00000366997	6	DIS3
ENSP00000311401	6	DENND6A
ENSP00000362727	6	DENND1A
ENSP00000335307	6	DEFB106A
ENSP00000306117	6	DDX19A
ENSP00000346483	6	DDRGK1
ENSP00000297579	6	DCAF13
ENSP00000360167	6	DCAF12L1
ENSP00000311899	6	DAW1
ENSP00000308430	6	CYB5B
ENSP00000391509	6	CUTA
ENSP00000382984	6	CSNK2B
ENSP00000413092	6	CSNK2B
ENSP00000241305	6	CPXM2
ENSP00000259997	6	CPEB2
ENSP00000262061	6	COPZ1
ENSP00000348054	6	COMMD6
ENSP00000419475	6	COMMD2
ENSP00000397441	6	COG6
ENSP00000194871	6	COBLL1
ENSP00000412217	6	CLIC1
ENSP00000281129	6	CEP128
ENSP00000419395	6	CDNF
ENSP00000334996	6	CCIN
ENSP00000383205	6	CCHCR1
ENSP00000320649	6	CCDC89
ENSP00000344749	6	CCDC37
ENSP00000295117	6	CCDC104
ENSP00000273936	6	CABS1
ENSP00000364964	6	C6orf25
ENSP00000231512	6	C5orf15
ENSP00000364660	6	C2
ENSP00000334848	6	C11orf74

ENSP00000278483	6	C11orf73
ENSP00000376799	6	C11orf65
ENSP00000419126	6	C10orf88
ENSP00000286067	6	C10orf12
ENSP00000222969	6	BUD31
ENSP00000338862	6	BRIX1
ENSP00000323557	6	BRD9
ENSP00000246090	6	BANF2
ENSP00000287590	6	B3GNT7
ENSP00000303740	6	B3GALT1
ENSP00000315568	6	AVL9
ENSP00000342481	6	ATP9A
ENSP00000411672	6	ATP6V0E2
ENSP00000315383	6	ASPRV1
ENSP00000355195	6	ASB12
ENSP00000323847	6	ARL14
ENSP00000338777	6	AP3S2
ENSP00000323387	6	ANKRD29
ENSP00000303570	6	ANKMY2
ENSP00000395863	6	AGPAT1
ENSP00000327453	6	ACSM2B
ENSP00000303246	6	ACOT12
ENSP00000278544	6	ACER3
ENSP00000356567	6	ACBD6
ENSP00000416404	6	ABHD16A
ENSP00000239730	6	-
ENSP00000269201	6	-
ENSP00000270457	6	-
ENSP00000273406	6	-
ENSP00000296394	6	-
ENSP00000297761	6	-
ENSP00000301171	6	-
ENSP00000307206	6	-
ENSP00000311153	6	-
ENSP00000314065	6	-
ENSP00000314827	6	-
ENSP00000332601	6	-
ENSP00000335486	6	-
ENSP00000340826	6	-
ENSP00000340868	6	-
ENSP00000342918	6	-
ENSP00000346793	6	-
ENSP00000355092	6	-
ENSP00000371358	6	-
ENSP00000372135	6	-
ENSP00000382022	6	-

ENSP00000382875	6	-
ENSP00000383628	6	-
ENSP00000385165	6	-
ENSP00000387795	6	-
ENSP00000388949	6	-
ENSP00000390835	6	-
ENSP00000392091	6	-
ENSP00000396289	6	-
ENSP00000396457	6	-
ENSP00000397971	6	-
ENSP00000398069	6	-
ENSP00000401262	6	-
ENSP00000401876	6	-
ENSP00000404539	6	-
ENSP00000410437	6	-
ENSP00000411492	6	-
ENSP00000416599	5	ZNRD1
ENSP00000332595	5	ZNF92
ENSP00000369777	5	ZNF768
ENSP00000252799	5	ZNF747
ENSP00000312141	5	ZNF654
ENSP00000338217	5	ZNF532
ENSP00000311521	5	ZNF490
ENSP00000308578	5	ZNF431
ENSP00000255129	5	ZNF334
ENSP00000344162	5	ZNF136
ENSP00000298585	5	ZMYND19
ENSP00000283441	5	ZDHHC11
ENSP00000262577	5	ZC3H3
ENSP00000362609	5	ZBTB8A
ENSP00000328079	5	WDR53
ENSP00000361740	5	WBP5
ENSP00000326379	5	VWA9
ENSP00000343366	5	VSTM1
ENSP00000272322	5	VPS54
ENSP00000320416	5	VPS37D
ENSP00000303129	5	VAT1L
ENSP00000401121	5	VARS
ENSP00000305647	5	VAMP5
ENSP00000363348	5	UQCC2
ENSP00000346155	5	UCKL1
ENSP00000382507	5	UBXN2B
ENSP00000288561	5	UBN2
ENSP00000368591	5	TTLL10
ENSP00000266254	5	TTLL1
ENSP00000327487	5	TSEN54

ENSP00000261249	5	TRMT5
ENSP00000356476	5	TRMT1L
ENSP00000369440	5	TRIM6
ENSP00000272395	5	TRIM43
ENSP00000389607	5	TRIM40
ENSP00000374891	5	TRBV6-5
ENSP00000374883	5	TRBV6-4
ENSP00000374876	5	TRBV6-1
ENSP00000374880	5	TRBV4-1
ENSP00000374916	5	TRBV19
ENSP00000302783	5	TRAPPC1
ENSP00000305664	5	TRABD
ENSP00000297071	5	TRA2A
ENSP00000381476	5	TMPRSS12
ENSP00000348639	5	TMPRSS11F
ENSP00000264728	5	TMEM40
ENSP00000301939	5	TMEM256
ENSP00000370736	5	TMEM165
ENSP00000275767	5	TMEM140
ENSP00000378166	5	TMCO6
ENSP00000294543	5	TMCO4
ENSP00000397843	5	TMA7
ENSP00000342322	5	TM7SF3
ENSP00000365534	5	THNSL1
ENSP00000258991	5	TEX2
ENSP00000290871	5	TEPP
ENSP00000370607	5	TECRL
ENSP00000304941	5	TCTN2
ENSP00000399388	5	TCF19
ENSP00000361859	5	TCEAL6
ENSP00000361765	5	TCEAL5
ENSP00000243286	5	TCEAL3
ENSP00000332359	5	TCEAL2
ENSP00000340693	5	TBC1D23
ENSP00000276692	5	TATDN1
ENSP00000240687	5	TAS2R7
ENSP00000358060	5	TARS2
ENSP00000394700	5	SYT16
ENSP00000316983	5	SV2C
ENSP00000366081	5	SSX4B
ENSP00000263736	5	SRBD1
ENSP00000295050	5	SPRTN
ENSP00000364054	5	SPIN3
ENSP00000312284	5	SPESP1
ENSP00000263672	5	SPCS2
ENSP00000359546	5	SPANXD

ENSP00000351884	5	SPANXC
ENSP00000359550	5	SPANXA1
ENSP00000266735	5	SNRPF
ENSP00000267488	5	SLC38A6
ENSP00000344648	5	SLC37A1
ENSP00000243389	5	SLC36A1
ENSP00000301891	5	SLC22A11
ENSP00000359376	5	SLC17A9
ENSP00000309751	5	SLC16A13
ENSP00000394400	5	SKIV2L
ENSP00000314023	5	SIDT2
ENSP00000332513	5	SH3BGR
ENSP00000261674	5	SFSWAP
ENSP00000310082	5	SETD6
ENSP00000382767	5	SEL1L3
ENSP00000369380	5	SCML1
ENSP00000291536	5	RSPH1
ENSP00000348849	5	RPS26
ENSP00000362532	5	RPRD1B
ENSP00000253788	5	RPL27
ENSP00000267291	5	RNF113B
ENSP00000350136	5	RIMKLB
ENSP00000330842	5	RGPD6
ENSP00000371169	5	RCL1
ENSP00000303712	5	RBMY1A1
ENSP00000355565	5	RBM34
ENSP00000341905	5	RASAL3
ENSP00000356231	5	RABIF
ENSP00000382241	5	QSER1
ENSP00000259845	5	PSORS1C2
ENSP00000403456	5	PSORS1C2
ENSP00000268281	5	PRSS36
ENSP00000409444	5	PRRC2A
ENSP00000342709	5	PRR19
ENSP00000314396	5	PRMT10
ENSP00000381010	5	PRKRIP1
ENSP00000335185	5	PRICKLE4
ENSP00000253008	5	PRDM12
ENSP00000346255	5	PPTC7
ENSP00000373107	5	PPP1R11
ENSP00000296484	5	POC1A
ENSP00000384610	5	PNPLA7
ENSP00000263657	5	PNO1
ENSP00000318131	5	PNMAL1
ENSP00000376372	5	PLD4
ENSP00000357069	5	PIGM

ENSP00000359848	5	PIGK
ENSP00000310978	5	PHYKPL
ENSP00000204549	5	PDCD7
ENSP00000367727	5	PANK4
ENSP00000363400	5	PAFAH2
ENSP00000285083	5	OXNAD1
ENSP00000365678	5	OTUD1
ENSP00000328090	5	OTOP3
ENSP00000332528	5	OTOP2
ENSP00000324557	5	OR2M7
ENSP00000410227	5	OR2H2
ENSP00000360270	5	OMA1
ENSP00000378504	5	NXF3
ENSP00000380251	5	NUDT19
ENSP00000388464	5	NSUN5
ENSP00000408335	5	NOTCH4
ENSP00000315674	5	NOP14
ENSP00000321929	5	NME9
ENSP00000254940	5	NIP7
ENSP00000215956	5	NHP2L1
ENSP00000330787	5	NDUFB1
ENSP00000416944	5	NCR3
ENSP00000358228	5	NBPF24
ENSP00000385903	5	NAA60
ENSP00000307887	5	MXRA8
ENSP00000402038	5	MTO1
ENSP00000355206	5	MT-ND3
ENSP00000245564	5	MSTO1
ENSP00000420714	5	MRPS14
ENSP00000347821	5	MORC4
ENSP00000308351	5	MLKL
ENSP00000289359	5	MITD1
ENSP00000325562	5	MINOS1
ENSP00000260953	5	METTL5
ENSP00000307077	5	METTL18
ENSP00000308957	5	MCTP1
ENSP00000317271	5	MCFD2
ENSP00000282276	5	MARS2
ENSP00000368266	5	MAGEB4
ENSP00000355198	5	MAGEB3
ENSP00000292430	5	LY6K
ENSP00000342711	5	LY6H
ENSP00000252622	5	LSM7
ENSP00000259324	5	LRRC8A
ENSP00000367498	5	LRRC47
ENSP00000239367	5	LRP11

ENSP00000377086	5	LONRF2
ENSP00000296877	5	LEAP2
ENSP00000357973	5	LACE1
ENSP00000257901	5	KRT85
ENSP00000352314	5	KLHL14
ENSP00000287152	5	KIF6
ENSP00000413445	5	KIAA1324L
ENSP00000385215	5	KIAA1009
ENSP00000258180	5	KIAA0513
ENSP00000258111	5	KCNMB4
ENSP00000319370	5	KCNMB3
ENSP00000218343	5	JADE3
ENSP00000420517	5	IGHJ3
ENSP00000397956	5	IER3
ENSP00000365895	5	HS6ST3
ENSP00000392347	5	HLA-G
ENSP00000259667	5	HINT2
ENSP00000381473	5	HIGD1C
ENSP00000413520	5	HID1
ENSP00000364371	5	HIATL2
ENSP00000300605	5	HDHD2
ENSP00000381220	5	HDDC2
ENSP00000352606	5	HAPLN3
ENSP00000403156	5	GTF2H4
ENSP00000367214	5	GRXCR2
ENSP00000305839	5	GPRIN1
ENSP00000358233	5	GPR89B
ENSP00000303149	5	GPR27
ENSP00000301917	5	GPR25
ENSP00000276077	5	GPR174
ENSP00000365529	5	GPR158
ENSP00000384516	5	GGTLC3
ENSP00000365426	5	GGACT
ENSP00000266754	5	GAS2L3
ENSP00000380488	5	GALNT9
ENSP00000364150	5	GALNT12
ENSP00000411286	5	GABBR1
ENSP00000305334	5	FSTL5
ENSP00000350032	5	FRMD4A
ENSP00000332886	5	FREM3
ENSP00000380037	5	FITM2
ENSP00000267426	5	FITM1
ENSP00000410007	5	FBXO46
ENSP00000341416	5	FBXO16
ENSP00000346874	5	FAR1
ENSP00000348852	5	FAM168A

ENSP00000329137	5	FAM132A
ENSP00000284274	5	FAM105B
ENSP00000300255	5	EVA1C
ENSP00000346635	5	ERO1LB
ENSP00000360561	5	ENTPD8
ENSP00000397426	5	EGFL8
ENSP00000378312	5	EFCAB5
ENSP00000382819	5	DXO
ENSP00000256538	5	DPH6
ENSP00000381791	5	DPF3
ENSP00000340017	5	DOC2A
ENSP00000365007	5	DNAJC16
ENSP00000268482	5	DHX38
ENSP00000327975	5	DHRS7C
ENSP00000364271	5	DFNB59
ENSP00000263256	5	DESI1
ENSP00000307126	5	DEGS2
ENSP00000371644	5	DEFB136
ENSP00000330460	5	DDX59
ENSP00000360828	5	DDX27
ENSP00000215770	5	DDTL
ENSP00000315351	5	D2HGDH
ENSP00000216775	5	CPNE6
ENSP00000230459	5	COX7A2
ENSP00000406327	5	COX20
ENSP00000354960	5	COLGALT2
ENSP00000252599	5	COLGALT1
ENSP00000263655	5	CNRIP1
ENSP00000351682	5	CNDP1
ENSP00000258711	5	CHST12
ENSP00000035307	5	CHPF2
ENSP00000287202	5	CELF6
ENSP00000290122	5	CELA3A
ENSP00000337358	5	CECR5
ENSP00000269967	5	CCDC97
ENSP00000328487	5	CCDC87
ENSP00000280245	5	CCDC83
ENSP00000278520	5	CCDC82
ENSP00000386866	5	CCDC14
ENSP00000325355	5	CASKIN2
ENSP00000378690	5	CALN1
ENSP00000358147	5	CACUL1
ENSP00000364784	5	C6orf48
ENSP00000326879	5	C5orf22
ENSP00000343172	5	C4orf40
ENSP00000320081	5	C3orf58

ENSP00000264848	5	C3orf52
ENSP00000290155	5	C21orf59
ENSP00000291691	5	C21orf58
ENSP00000335285	5	C1QL4
ENSP00000363768	5	C1QC
ENSP00000350704	5	C1orf109
ENSP00000327950	5	C19orf71
ENSP00000376103	5	C19orf12
ENSP00000380933	5	C12orf74
ENSP00000229281	5	C12orf57
ENSP00000288757	5	C12orf43
ENSP00000340296	5	C10orf129
ENSP00000403393	5	BRD2
ENSP00000300399	5	BPIFC
ENSP00000361281	5	BEST4
ENSP00000301587	5	ATP5H
ENSP00000293709	5	ATF6B
ENSP00000404814	5	ATF6B
ENSP00000367638	5	ARHGEF39
ENSP00000174653	5	AP3M2
ENSP00000258749	5	AOAH
ENSP00000369434	5	ANKRD16
ENSP00000343362	5	ANKFY1
ENSP00000272647	5	AMMECR1L
ENSP00000323096	5	AMIGO3
ENSP00000382091	5	ALKBH5
ENSP00000392678	5	AKIRIN1
ENSP00000393114	5	AIF1
ENSP00000353114	5	AHNAK2
ENSP00000389516	5	AGPAT1
ENSP00000405864	5	AGPAT1
ENSP00000332448	5	ADAT3
ENSP00000368605	5	ACOT9
ENSP00000356632	5	ABRACL
ENSP00000302657	5	ABHD15
ENSP00000354841	5	ABHD14B
ENSP00000412553	5	ABCF1
ENSP00000313674	5	AAR2
ENSP00000193422	5	-
ENSP00000222301	5	-
ENSP00000255289	5	-
ENSP00000258459	5	-
ENSP00000262801	5	-
ENSP00000262946	5	-
ENSP00000265616	5	-
ENSP00000296596	5	-

ENSP00000299989	5	-
ENSP00000300147	5	-
ENSP00000317400	5	-
ENSP00000323063	5	-
ENSP00000323252	5	-
ENSP00000337518	5	-
ENSP00000338524	5	-
ENSP00000344587	5	-
ENSP00000346085	5	-
ENSP00000349380	5	-
ENSP00000356182	5	-
ENSP00000368677	5	-
ENSP00000371763	5	-
ENSP00000372767	5	-
ENSP00000372860	5	-
ENSP00000372980	5	-
ENSP00000375309	5	-
ENSP00000375370	5	-
ENSP00000380055	5	-
ENSP00000389125	5	-
ENSP00000389657	5	-
ENSP00000392535	5	-
ENSP00000397119	5	-
ENSP00000400610	5	-
ENSP00000402962	5	-
ENSP00000405143	5	-
ENSP00000405382	5	-
ENSP00000407619	5	-
ENSP00000407737	5	-
ENSP00000409376	5	-
ENSP00000410782	5	-
ENSP00000411702	5	-
ENSP00000413171	5	-
ENSP00000415539	5	-
ENSP00000416693	5	-
ENSP00000381693	4	ZSWIM8
ENSP00000345339	4	ZSCAN31
ENSP00000223210	4	ZNF862
ENSP00000359802	4	ZNF75D
ENSP00000353957	4	ZNF567
ENSP00000296600	4	ZNF474
ENSP00000313582	4	ZNF436
ENSP00000247956	4	ZNF317
ENSP00000354501	4	ZNF277
ENSP00000276816	4	ZNF16
ENSP00000349708	4	ZMYM6

ENSP00000347498	4	ZFYVE19
ENSP00000363257	4	ZDHHC18
ENSP00000365130	4	ZCCHC6
ENSP00000345633	4	ZCCHC13
ENSP00000362556	4	ZBTB43
ENSP00000238831	4	YIPF4
ENSP00000292778	4	YDJC
ENSP00000223273	4	YAE1D1
ENSP00000349658	4	XPNPEP3
ENSP00000302938	4	WFDC13
ENSP00000351100	4	WDR55
ENSP00000309457	4	VPS41
ENSP00000321309	4	VCX2
ENSP00000356433	4	UST
ENSP00000309565	4	UQCRH
ENSP00000332887	4	UQCR10
ENSP00000334044	4	UBL4B
ENSP00000304908	4	TXNDC2
ENSP00000400663	4	TUBB
ENSP00000262605	4	TTPAL
ENSP00000266182	4	TTLL8
ENSP00000260505	4	TTLL7
ENSP00000258398	4	TTLL4
ENSP00000336127	4	TTC7B
ENSP00000360323	4	TTC22
ENSP00000261647	4	TTC19
ENSP00000203001	4	TRMT6
ENSP00000362026	4	TRMT2B
ENSP00000343765	4	TRIM60
ENSP00000348216	4	TRIM4
ENSP00000344724	4	TOX2
ENSP00000381856	4	TOMM6
ENSP00000301599	4	TMEM88
ENSP00000238788	4	TMEM214
ENSP00000380747	4	TMEM209
ENSP00000305069	4	TMEM192
ENSP00000254742	4	TMEM128
ENSP00000416050	4	TM2D2
ENSP00000280605	4	TKTL2
ENSP00000373167	4	TIGIT
ENSP00000338607	4	THEM6
ENSP00000340088	4	THEG
ENSP00000357811	4	TEX36
ENSP00000355323	4	TEX28
ENSP00000340969	4	TEX264
ENSP00000392879	4	TCP11X2

ENSP00000335595	4	TCP11L1
ENSP00000265112	4	TARS
ENSP00000395701	4	TAPBP
ENSP00000261778	4	TANGO6
ENSP00000353631	4	SYTL3
ENSP00000316609	4	SWI5
ENSP00000346130	4	SVIP
ENSP00000003583	4	STPG1
ENSP00000395864	4	STK19
ENSP00000262915	4	ST3GAL3
ENSP00000359119	4	SRPK3
ENSP00000357739	4	SPRR2E
ENSP00000367494	4	SPIRE2
ENSP00000258704	4	SPDYE1
ENSP00000366390	4	SPDYC
ENSP00000341765	4	SPATA16
ENSP00000359529	4	SPANXN2
ENSP00000332062	4	SNX20
ENSP00000339834	4	SNRNP48
ENSP00000353557	4	SLC35F1
ENSP00000240333	4	SLC35B1
ENSP00000309504	4	SLC26A7
ENSP00000217909	4	SLC25A43
ENSP00000326693	4	SLC25A42
ENSP00000297578	4	SLC25A32
ENSP00000206544	4	SLC22A17
ENSP00000314606	4	SGSH
ENSP00000351981	4	SGMS2
ENSP00000383939	4	SETD5
ENSP00000415332	4	SESTD1
ENSP00000223641	4	SEC61B
ENSP00000299714	4	SEC11C
ENSP00000272732	4	SCRN3
ENSP00000366645	4	SCP2D1
ENSP00000351395	4	SCGB1D4
ENSP00000388920	4	RWDD4
ENSP00000298317	4	RPUSD4
ENSP00000391247	4	RPP21
ENSP00000346012	4	RPL36AL
ENSP00000294189	4	RPL29
ENSP00000265100	4	RPL26L1
ENSP00000222247	4	RPL18A
ENSP00000388550	4	RNF24
ENSP00000006777	4	RHBDD2
ENSP00000343081	4	PUS7L
ENSP00000386621	4	PTGES3L-AARSD1

ENSP00000311121	4	PSMA8
ENSP00000373937	4	PRTG
ENSP00000414624	4	PRPSAP1
ENSP00000257181	4	PRPF38A
ENSP00000413596	4	PPP1R18
ENSP00000338510	4	PPHLN1
ENSP00000410855	4	POM121L7
ENSP00000366828	4	POLR3F
ENSP00000361446	4	POLR3A
ENSP00000275072	4	PM20D2
ENSP00000354332	4	PLEKHS1
ENSP00000247226	4	PLEKHG3
ENSP00000273371	4	PLA1A
ENSP00000335618	4	PITPNC1
ENSP00000164305	4	PIGB
ENSP00000403042	4	PHTF2
ENSP00000298198	4	PGM2L1
ENSP00000321691	4	PDDC1
ENSP00000385713	4	PCNXL4
ENSP00000354724	4	PCGF3
ENSP00000372757	4	PBX2
ENSP00000361914	4	OXCT2
ENSP00000368989	4	OR6C3
ENSP00000369279	4	OR52E6
ENSP00000329982	4	OR4F16
ENSP00000324687	4	OR2T33
ENSP00000363216	4	OGDHL
ENSP00000419718	4	OFCC1
ENSP00000340159	4	NWD1
ENSP00000258662	4	NUDT15
ENSP00000358019	4	NSMCE4A
ENSP00000342535	4	NLGN4Y
ENSP00000401739	4	NKPD1
ENSP00000237889	4	NDUFB3
ENSP00000362873	4	NDUFA8
ENSP00000330737	4	NDUFA12
ENSP00000358372	4	NBPF8
ENSP00000253719	4	NAPSA
ENSP00000302441	4	NANP
ENSP00000249299	4	NAA38
ENSP00000368716	4	NAA16
ENSP00000286794	4	NAA11
ENSP00000221086	4	MTMR9
ENSP00000370508	4	MTIF3
ENSP00000320567	4	MRPS33
ENSP00000357823	4	MRPL9

ENSP00000347277	4	MRPL52
ENSP00000312311	4	MRPL46
ENSP00000223324	4	MRPL32
ENSP00000396622	4	MROH7
ENSP00000040663	4	MRI1
ENSP00000372445	4	METTL17
ENSP00000315731	4	METRNL
ENSP00000359890	4	MBNL3
ENSP00000410818	4	MAD2L1BP
ENSP00000395699	4	LY6G5B
ENSP00000336817	4	LUZP2
ENSP00000356548	4	LTV1
ENSP00000410758	4	LSM5
ENSP00000304923	4	LRRC28
ENSP00000366280	4	LMAN2L
ENSP00000310431	4	LMAN1L
ENSP00000259006	4	LIMD2
ENSP00000251372	4	LILRA1
ENSP00000356247	4	LGR6
ENSP00000295682	4	KRTCAP2
ENSP00000366984	4	KRT40
ENSP00000250351	4	KLK12
ENSP00000365172	4	KDELC1
ENSP00000297404	4	KCNV1
ENSP00000368799	4	KBTBD6
ENSP00000362071	4	JPH2
ENSP00000372292	4	IQSEC3
ENSP00000374826	4	IGLV1-50
ENSP00000327344	4	IGIP
ENSP00000415823	4	IGFL1
ENSP00000264020	4	IFT46
ENSP00000349364	4	IFFO1
ENSP00000303599	4	HSFY1
ENSP00000263278	4	HSD17B14
ENSP00000305193	4	HS1BP3
ENSP00000312625	4	HP1BP3
ENSP00000232854	4	HEMK1
ENSP00000222511	4	GTPBP10
ENSP00000401105	4	GTF2H4
ENSP00000328672	4	GPRIN3
ENSP00000378195	4	GPR75
ENSP00000229955	4	GPR63
ENSP00000319250	4	GPR62
ENSP00000342981	4	GPR114
ENSP00000352547	4	GPAT2
ENSP00000388687	4	GPANK1

ENSP00000412884	4	GPANK1
ENSP00000352111	4	GOLGA8A
ENSP00000300515	4	GOLGA6L9
ENSP00000266069	4	GID8
ENSP00000227756	4	GALNT18
ENSP00000297107	4	GALNT10
ENSP00000417492	4	FSD1L
ENSP00000344307	4	FOCAD
ENSP00000362570	4	FNDC5
ENSP00000321386	4	FBXO39
ENSP00000283946	4	FBXO36
ENSP00000258200	4	FBXL8
ENSP00000354814	4	FAM63A
ENSP00000309786	4	FAM21D
ENSP00000354891	4	FAM163A
ENSP00000286544	4	FAM161B
ENSP00000306888	4	FAM151A
ENSP00000233379	4	FAHD2A
ENSP00000272427	4	EXOC6B
ENSP00000359410	4	EPHX4
ENSP00000229003	4	ENDOU
ENSP00000309175	4	EIF1AD
ENSP00000010132	4	DYRK4
ENSP00000271385	4	DUSP27
ENSP00000344937	4	DPY19L3
ENSP00000305948	4	DPCR1
ENSP00000263697	4	DNAJC8
ENSP00000264711	4	DNAJC27
ENSP00000302843	4	DNAJC18
ENSP00000344431	4	DNAJB13
ENSP00000199320	4	DIMT1
ENSP00000330509	4	DEXI
ENSP00000246105	4	DEFB129
ENSP00000258772	4	DDX56
ENSP00000247003	4	DDX49
ENSP00000379475	4	DDX39B
ENSP00000361232	4	DDX31
ENSP00000359788	4	DDX26B
ENSP00000371682	4	DCAF16
ENSP00000366953	4	DCAF10
ENSP00000344989	4	DALRD3
ENSP00000294072	4	CYB561A3
ENSP00000410942	4	CSNK2B
ENSP00000299721	4	CPLX4
ENSP00000340568	4	COX8C
ENSP00000262507	4	COQ9

ENSP00000300452	4	COQ4
ENSP00000366032	4	COMMD3
ENSP00000305442	4	COG7
ENSP00000330730	4	COA5
ENSP00000347919	4	COA4
ENSP00000353698	4	CNPPD1
ENSP00000289382	4	CNOT11
ENSP00000420443	4	CNIH4
ENSP00000369268	4	CNGA4
ENSP00000219400	4	CMC2
ENSP00000299642	4	CLEC3A
ENSP00000359594	4	CLCA4
ENSP00000356385	4	CFHR2
ENSP00000385739	4	CEACAM21
ENSP00000295887	4	CDS1
ENSP00000336587	4	CDR2L
ENSP00000314544	4	CCZ1B
ENSP00000374260	4	CCDC149
ENSP00000344627	4	CCDC141
ENSP00000329360	4	CCDC137
ENSP00000365577	4	CCDC120
ENSP00000378145	4	CCDC109B
ENSP00000366608	4	CBWD7
ENSP00000380523	4	CAPSL
ENSP00000367755	4	CAMKMT
ENSP00000361524	4	C9orf78
ENSP00000316262	4	C8orf82
ENSP00000315614	4	C8orf47
ENSP00000304071	4	C7orf33
ENSP00000386139	4	C5orf55
ENSP00000295079	4	C2orf47
ENSP00000389111	4	C1orf233
ENSP00000313500	4	C17orf53
ENSP00000331720	4	C16orf72
ENSP00000355022	4	C16orf59
ENSP00000325144	4	C15orf53
ENSP00000367189	4	C11orf83
ENSP00000398350	4	C11orf68
ENSP00000307264	4	C11orf24
ENSP00000359674	4	C10orf62
ENSP00000403151	4	C10orf105
ENSP00000371724	4	BPY2C
ENSP00000371829	4	BPY2B
ENSP00000332413	4	BEST3
ENSP00000352144	4	B4GALT4
ENSP00000349293	4	B4GALT2

ENSP00000368496	4	B3GALT6
ENSP00000304891	4	ATP6V1E2
ENSP00000265093	4	ATP6V0E1
ENSP00000377878	4	ATP5G2
ENSP00000368062	4	ATAD3C
ENSP00000238789	4	ATAD2B
ENSP00000333395	4	ARSI
ENSP00000267339	4	ANKRD10
ENSP00000307481	4	AMDHD2
ENSP00000266736	4	AMDHD1
ENSP00000369099	4	ADPRM
ENSP00000262198	4	ADNP2
ENSP00000296513	4	ADAD1
ENSP00000261206	4	ACSS3
ENSP00000414066	4	ACN9
ENSP00000406965	4	ABHD16A
ENSP00000222800	4	ABHD11
ENSP00000236709	4	A4GNT
ENSP00000249005	4	A4GALT
ENSP00000355877	4	1-Mar
ENSP00000216463	4	-
ENSP00000233468	4	-
ENSP00000300983	4	-
ENSP00000312355	4	-
ENSP00000323846	4	-
ENSP00000325668	4	-
ENSP00000330732	4	-
ENSP00000332123	4	-
ENSP00000332975	4	-
ENSP00000337455	4	-
ENSP00000345065	4	-
ENSP00000345980	4	-
ENSP00000351384	4	-
ENSP00000364654	4	-
ENSP00000380255	4	-
ENSP00000380341	4	-
ENSP00000382606	4	-
ENSP00000383418	4	-
ENSP00000383587	4	-
ENSP00000384740	4	-
ENSP00000387389	4	-
ENSP00000388081	4	-
ENSP00000388274	4	-
ENSP00000388414	4	-
ENSP00000388803	4	-
ENSP00000393383	4	-

ENSP00000393751	4	-
ENSP00000397758	4	-
ENSP00000397974	4	-
ENSP00000398103	4	-
ENSP00000398223	4	-
ENSP00000398359	4	-
ENSP00000398671	4	-
ENSP00000404013	4	-
ENSP00000405110	4	-
ENSP00000410634	4	-
ENSP00000411115	4	-
ENSP00000413009	4	-
ENSP00000413443	4	-
ENSP00000413677	4	-
ENSP00000414069	4	-
ENSP00000415763	4	-
ENSP00000415880	4	-
ENSP00000415912	4	-
ENSP00000416095	4	-
ENSP00000360583	3	ZYG11A
ENSP00000307801	3	ZPLD1
ENSP00000331111	3	ZNRD1
ENSP00000340683	3	ZNF823
ENSP00000351482	3	ZNF707
ENSP00000391200	3	ZNF692
ENSP00000255746	3	ZNF679
ENSP00000355459	3	ZNF670
ENSP00000347338	3	ZNF607
ENSP00000363384	3	ZNF593
ENSP00000292841	3	ZNF585A
ENSP00000282282	3	ZNF547
ENSP00000311679	3	ZNF483
ENSP00000379451	3	ZNF418
ENSP00000311319	3	ZNF417
ENSP00000340796	3	ZNF326
ENSP00000219069	3	ZNF263
ENSP00000250076	3	ZNF232
ENSP00000335437	3	ZNF20
ENSP00000274712	3	ZMAT2
ENSP00000254037	3	ZCCHC9
ENSP00000301011	3	ZC3H18
ENSP00000323678	3	ZADH2
ENSP00000326813	3	YOD1
ENSP00000333775	3	XAGE2
ENSP00000370242	3	WWC3
ENSP00000361871	3	WFDC12

ENSP00000294664	3	WDR63
ENSP00000402869	3	WDR46
ENSP00000384302	3	WDR43
ENSP00000368612	3	VWA8
ENSP00000267202	3	VPS37B
ENSP00000415434	3	USP40
ENSP00000258399	3	USP37
ENSP00000347997	3	UBQLN3
ENSP00000359525	3	UBE2NL
ENSP00000373039	3	UBD
ENSP00000386935	3	UAP1L1
ENSP00000352645	3	TYW1
ENSP00000410829	3	TUBB
ENSP00000216129	3	TTLL12
ENSP00000321346	3	TTLL11
ENSP00000333018	3	TTC32
ENSP00000419279	3	TTC26
ENSP00000348996	3	TSGA13
ENSP00000346916	3	TRIM6-TRIM34
ENSP00000365924	3	TRIM31
ENSP00000408233	3	TRIM26
ENSP00000374917	3	TRBV20-1
ENSP00000417300	3	TRBC2
ENSP00000323455	3	TPRX1
ENSP00000384411	3	TOMM5
ENSP00000336783	3	TNRC6C
ENSP00000330475	3	TMPRSS11B
ENSP00000311842	3	TMEM9B
ENSP00000401338	3	TMEM8A
ENSP00000283206	3	TMEM87B
ENSP00000264452	3	TMEM33
ENSP00000402698	3	TMEM30C
ENSP00000372720	3	TMEM241
ENSP00000162044	3	TMEM161A
ENSP00000206380	3	TMEM101
ENSP00000356856	3	TMCO1
ENSP00000350630	3	TLL2
ENSP00000169551	3	TIMM21
ENSP00000215570	3	TIMM13
ENSP00000292894	3	THAP8
ENSP00000330877	3	TEX40
ENSP00000404923	3	TECPR1
ENSP00000362590	3	TBC1D22B
ENSP00000401149	3	TAP1
ENSP00000258034	3	TAAR5
ENSP00000383866	3	SZRD1

ENSP00000358888	3	SYPL2
ENSP00000293695	3	SYCE2
ENSP00000341282	3	SYCE1
ENSP00000308727	3	SUSD5
ENSP00000297325	3	SUN3
ENSP00000272452	3	SULT1C4
ENSP00000329200	3	STAC3
ENSP00000291839	3	ST6GALNAC6
ENSP00000233025	3	SPCS1
ENSP00000404277	3	SPATA6L
ENSP00000289805	3	SPATA2L
ENSP00000352927	3	SOGA2
ENSP00000343709	3	SNX10
ENSP00000319597	3	SNAPC5
ENSP00000363345	3	SLC46A2
ENSP00000321498	3	SLC37A3
ENSP00000297307	3	SLC35G3
ENSP00000400932	3	SLC35G1
ENSP00000345528	3	SLC35F6
ENSP00000234800	3	SLC35E2B
ENSP00000352849	3	SIRPB2
ENSP00000358909	3	SFXN2
ENSP00000354590	3	SFT2D1
ENSP00000353440	3	SEZ6
ENSP00000265965	3	SERGEF
ENSP00000243253	3	SEC61A1
ENSP00000406547	3	SCAMP5
ENSP00000387942	3	SAPCD1
ENSP00000300013	3	SAAL1
ENSP00000418693	3	RWDD2B
ENSP00000341963	3	RSC1A1
ENSP00000321449	3	RRP7A
ENSP00000230050	3	RPS12
ENSP00000358064	3	RPRD2
ENSP00000369391	3	RPP40
ENSP00000389565	3	RPP21
ENSP00000403833	3	RPP21
ENSP00000295959	3	RPP14
ENSP00000339027	3	RPLP0
ENSP00000346022	3	RPL9
ENSP00000084795	3	RPL18
ENSP00000309334	3	RPL15
ENSP00000345156	3	RPL14
ENSP00000306123	3	RPAP1
ENSP00000304670	3	RNFT1
ENSP00000373106	3	RNF39

ENSP00000340162	3	RNASE9
ENSP00000311398	3	RNASE8
ENSP00000414330	3	RIMKLA
ENSP00000322775	3	RHBDF2
ENSP00000381253	3	RGPD1
ENSP00000409315	3	RBM18
ENSP00000226105	3	RANGRF
ENSP00000317903	3	R3HDM2
ENSP00000350094	3	QRICH1
ENSP00000316675	3	PVRIG
ENSP00000368318	3	PUSL1
ENSP00000370013	3	PTCD2
ENSP00000372595	3	PSMB9
ENSP00000401328	3	PRSS48
ENSP00000401932	3	PRSS45
ENSP00000401701	3	PRSS42
ENSP00000297767	3	PRSS37
ENSP00000327168	3	PRR7
ENSP00000262293	3	PRR11
ENSP00000372789	3	PPT2
ENSP00000416505	3	PPT2
ENSP00000393832	3	PPP1R3G
ENSP00000055335	3	PPP1R3F
ENSP00000373067	3	PPP1R18
ENSP00000404066	3	PPP1R18
ENSP00000405234	3	PPP1R18
ENSP00000365963	3	PPP1R11
ENSP00000365694	3	PPP1R10
ENSP00000295908	3	PPM1K
ENSP00000216452	3	PIGH
ENSP00000358753	3	PIFO
ENSP00000285141	3	PIEZO2
ENSP00000378788	3	PHF20L1
ENSP00000305465	3	PDILT
ENSP00000309142	3	PDE12
ENSP00000287196	3	PARP6
ENSP00000298440	3	OXGR1
ENSP00000230223	3	OR5V1
ENSP00000351466	3	OR5AC2
ENSP00000330049	3	OR4Q3
ENSP00000340748	3	OR13H1
ENSP00000359384	3	OOEP
ENSP00000294794	3	OLFML2B
ENSP00000371994	3	NYNRIN
ENSP00000307852	3	NUDT18
ENSP00000365160	3	NUDT11

ENSP00000347626	3	NUDCD3
ENSP00000406933	3	NT5DC2
ENSP00000366519	3	NSUN6
ENSP00000363899	3	NIPSNAP3A
ENSP00000368920	3	NHLRC3
ENSP00000382390	3	NEURL4
ENSP00000274137	3	NDUFS6
ENSP00000215565	3	NDUFB7
ENSP00000398290	3	NDUFA3
ENSP00000320083	3	NAALAD2
ENSP00000372202	3	N6AMT2
ENSP00000350332	3	MYBPC2
ENSP00000354772	3	MVB12B
ENSP00000300226	3	MS4A8
ENSP00000276585	3	MRPS28
ENSP00000253686	3	MRPS25
ENSP00000362208	3	MRPS15
ENSP00000331849	3	MRPL54
ENSP00000308717	3	MRPL48
ENSP00000253099	3	MRPL4
ENSP00000308275	3	MRPL38
ENSP00000372093	3	MRPL36
ENSP00000252602	3	MRPL34
ENSP00000361084	3	MRPL14
ENSP00000250156	3	MRM1
ENSP00000305766	3	MRGPRX1
ENSP00000278949	3	MPZL3
ENSP00000406674	3	MOGAT1
ENSP00000271139	3	MOB3C
ENSP00000327124	3	MIEF1
ENSP00000258436	3	MFSD9
ENSP00000332624	3	MFSD5
ENSP00000356115	3	MFSD4
ENSP00000322956	3	MFAP3
ENSP00000366766	3	METTL3
ENSP00000262432	3	METTL2B
ENSP00000263092	3	METTL16
ENSP00000357829	3	METTL10
ENSP00000318086	3	MAP7D3
ENSP00000363452	3	MAN1C1
ENSP00000353246	3	MAK16
ENSP00000319240	3	MAGOHB
ENSP00000333487	3	MAGEA2B
ENSP00000363638	3	LYPLA2
ENSP00000364978	3	LY6G6C
ENSP00000360988	3	LUZP4

ENSP00000402413	3	LTA
ENSP00000406280	3	LSM2
ENSP00000293406	3	LSM12
ENSP00000409403	3	LRTOMT
ENSP00000298124	3	LRRC18
ENSP00000300591	3	LOXHD1
ENSP00000360690	3	LONRF3
ENSP00000262301	3	LMF1
ENSP00000357835	3	LHPP
ENSP00000238875	3	LGALSL
ENSP00000300051	3	LDHD
ENSP00000349923	3	LAGE3
ENSP00000354526	3	L3MBTL3
ENSP00000355302	3	KRTAP1-5
ENSP00000366976	3	KRTAP1-4
ENSP00000344420	3	KRTAP1-3
ENSP00000347823	3	KRT39
ENSP00000330878	3	KPNA7
ENSP00000341549	3	KLHL33
ENSP00000356123	3	KLHDC8A
ENSP00000316681	3	KIAA1731
ENSP00000381198	3	KIAA1468
ENSP00000380692	3	KDM7A
ENSP00000312814	3	KCTD2
ENSP00000384391	3	KCTD17
ENSP00000391498	3	KCNK16
ENSP00000228799	3	ITFG2
ENSP00000324882	3	IQUB
ENSP00000385597	3	IQCE
ENSP00000387347	3	IQCA1
ENSP00000271417	3	ILDR2
ENSP00000374828	3	IGLV5-48
ENSP00000413304	3	IGKV6D-21
ENSP00000238609	3	IFI27L2
ENSP00000417580	3	IAH1
ENSP00000359444	3	HSFX1
ENSP00000381785	3	HSDL2
ENSP00000297679	3	HSD3B7
ENSP00000407154	3	HSD11B1L
ENSP00000330606	3	HS3ST4
ENSP00000341285	3	HNRNPA1L2
ENSP00000350090	3	HNRNPA1L2
ENSP00000348316	3	HN1
ENSP00000381654	3	HMGCLL1
ENSP00000387624	3	HLA-G
ENSP00000365817	3	HLA-E

ENSP00000378786	3	HLA-DRA
ENSP00000372718	3	HLA-DMB
ENSP00000349478	3	HCAR1
ENSP00000329662	3	H1FX
ENSP00000339750	3	GTDC1
ENSP00000228887	3	GPRC5D
ENSP00000303549	3	GPR82
ENSP00000362746	3	GPR21
ENSP00000308479	3	GPR171
ENSP00000367125	3	GPR153
ENSP00000297468	3	GPR146
ENSP00000386772	3	GPATCH11
ENSP00000216410	3	GNPNAT1
ENSP00000373090	3	GNL1
ENSP00000278765	3	GGTLC1
ENSP00000359485	3	GBP6
ENSP00000359512	3	GBP3
ENSP00000376570	3	GALNT13
ENSP00000296137	3	FYCO1
ENSP00000366630	3	FOXD4L4
ENSP00000355938	3	FLVCR1
ENSP00000353794	3	FBXL22
ENSP00000380734	3	FAM98B
ENSP00000387471	3	FAM96B
ENSP00000298784	3	FAM35A
ENSP00000253490	3	FAM153B
ENSP00000353887	3	FAM153A
ENSP00000282226	3	FAM151B
ENSP00000375267	3	FAM127B
ENSP00000351740	3	FAM114A1
ENSP00000181796	3	FAM107B
ENSP00000288985	3	ERCC6L2
ENSP00000254928	3	ERAL1
ENSP00000282041	3	EPG5
ENSP00000345974	3	ENOSF1
ENSP00000216799	3	EMC9
ENSP00000416892	3	EIF3L
ENSP00000370258	3	EIF3CL
ENSP00000351125	3	ECHDC2
ENSP00000242827	3	EBPL
ENSP00000337759	3	DXO
ENSP00000391123	3	DXO
ENSP00000302892	3	DTWD2
ENSP00000296097	3	DNAJC5G
ENSP00000276570	3	DNAJC5B
ENSP00000321711	3	DIS3L

ENSP00000408956	3	DHX16
ENSP00000263035	3	DHTKD1
ENSP00000378887	3	DHRS7B
ENSP00000251312	3	DHRS11
ENSP00000262585	3	DENND3
ENSP00000238146	3	DDX55
ENSP00000306407	3	DBNDD1
ENSP00000331556	3	CYLC1
ENSP00000282251	3	CWF19L2
ENSP00000311300	3	CTSW
ENSP00000352222	3	CTPS2
ENSP00000372459	3	CTIF
ENSP00000261660	3	CPPED1
ENSP00000404078	3	COX6A1P2
ENSP00000295890	3	COX18
ENSP00000326052	3	COX14
ENSP00000006101	3	COPZ2
ENSP00000396304	3	CLDN25
ENSP00000349456	3	CLCC1
ENSP00000391402	3	CISD3
ENSP00000357477	3	CEP85L
ENSP00000352841	3	CEP57L1
ENSP00000312706	3	CCDC65
ENSP00000216144	3	CABP7
ENSP00000364613	3	C9orf89
ENSP00000346345	3	C9orf163
ENSP00000297324	3	C8orf48
ENSP00000335500	3	C7orf57
ENSP00000340220	3	C7orf26
ENSP00000371061	3	C5orf51
ENSP00000326110	3	C5orf30
ENSP00000322469	3	C3orf38
ENSP00000359115	3	C20orf195
ENSP00000408078	3	C1orf222
ENSP00000359401	3	C1orf146
ENSP00000316465	3	C18orf56
ENSP00000285697	3	C16orf87
ENSP00000219139	3	C16orf70
ENSP00000317579	3	C16orf58
ENSP00000170150	3	BPIFB2
ENSP00000331369	3	BOLA3
ENSP00000296424	3	BDH2
ENSP00000335201	3	BCDIN3D
ENSP00000400157	3	B3GNT9
ENSP00000397259	3	ATXN7L3
ENSP00000335203	3	ATPIF1

ENSP00000372995	3	ATP6V1G2
ENSP00000256031	3	ATP13A3
ENSP00000387185	3	ATHL1
ENSP00000394914	3	ATAT1
ENSP00000306410	3	ARMC4
ENSP00000246041	3	AP5S1
ENSP00000286918	3	ANKRD9
ENSP00000377170	3	ANKRD39
ENSP00000265140	3	ANKRD32
ENSP00000351875	3	ANKRD30B
ENSP00000227618	3	ANAPC15
ENSP00000368152	3	ALKBH6
ENSP00000239891	3	ALG5
ENSP00000310120	3	ALG10B
ENSP00000266483	3	ALG10
ENSP00000349525	3	AHSA2
ENSP00000238561	3	ADCK1
ENSP00000352177	3	ADAM29
ENSP00000256389	3	ADAM20
ENSP00000300441	3	ACSF2
ENSP00000323071	3	ACOT4
ENSP00000274849	3	ABT1
ENSP00000269081	3	ABCA10
ENSP00000233609	3	-
ENSP00000257962	3	-
ENSP00000261683	3	-
ENSP00000269064	3	-
ENSP00000279716	3	-
ENSP00000293431	3	-
ENSP00000298129	3	-
ENSP00000299564	3	-
ENSP00000300233	3	-
ENSP00000301284	3	-
ENSP00000313223	3	-
ENSP00000328271	3	-
ENSP00000335197	3	-
ENSP00000342291	3	-
ENSP00000351143	3	-
ENSP00000354582	3	-
ENSP00000366629	3	-
ENSP00000371232	3	-
ENSP00000371390	3	-
ENSP00000373507	3	-
ENSP00000373941	3	-
ENSP00000382491	3	-
ENSP00000382849	3	-

ENSP00000389053	3	-
ENSP00000389465	3	-
ENSP00000390134	3	-
ENSP00000391044	3	-
ENSP00000392778	3	-
ENSP00000396169	3	-
ENSP00000396377	3	-
ENSP00000397334	3	-
ENSP00000397624	3	-
ENSP00000397872	3	-
ENSP00000401212	3	-
ENSP00000401489	3	-
ENSP00000402148	3	-
ENSP00000403669	3	-
ENSP00000404192	3	-
ENSP00000405369	3	-
ENSP00000407388	3	-
ENSP00000407508	3	-
ENSP00000408494	3	-
ENSP00000411620	3	-
ENSP00000412031	3	-
ENSP00000412074	3	-
ENSP00000412521	3	-
ENSP00000412809	3	-
ENSP00000413616	3	-
ENSP00000413654	3	-
ENSP00000414664	3	-
ENSP00000415470	3	-
ENSP00000412253	2	ZSCAN18
ENSP00000397636	2	ZNRD1
ENSP00000405636	2	ZNRD1
ENSP00000410530	2	ZNRD1
ENSP00000410954	2	ZNRD1
ENSP00000414110	2	ZNRD1
ENSP00000329793	2	ZNF85
ENSP00000331465	2	ZNF84
ENSP00000380840	2	ZNF709
ENSP00000361584	2	ZNF691
ENSP00000287461	2	ZNF689
ENSP00000310042	2	ZNF622
ENSP00000322427	2	ZNF611
ENSP00000307746	2	ZNF608
ENSP00000347730	2	ZNF536
ENSP00000223428	2	ZNF510
ENSP00000339585	2	ZNF449
ENSP00000282286	2	ZNF304

ENSP00000403441	2	ZNF30
ENSP00000267807	2	ZNF280D
ENSP00000397693	2	ZNF28
ENSP00000418719	2	ZNF273
ENSP00000263095	2	ZNF264
ENSP00000349494	2	ZNF254
ENSP00000292562	2	ZNF251
ENSP00000253159	2	ZNF236
ENSP00000288177	2	ZNF19
ENSP00000269834	2	ZIM3
ENSP00000361790	2	ZFP69
ENSP00000368017	2	ZFC3H1
ENSP00000269499	2	ZCCHC2
ENSP00000362758	2	ZBTB26
ENSP00000388191	2	ZBTB22
ENSP00000331933	2	WSCD2
ENSP00000222190	2	WDR83OS
ENSP00000372695	2	WDR46
ENSP00000308976	2	VWC2L
ENSP00000362560	2	VSTM2L
ENSP00000322339	2	VN1R1
ENSP00000353998	2	VKORC1L1
ENSP00000345216	2	USP54
ENSP00000400880	2	USP17L11
ENSP00000372199	2	URB1
ENSP00000256339	2	UNC79
ENSP00000282507	2	UGT3A2
ENSP00000312107	2	UBXN2A
ENSP00000364240	2	UBXN10
ENSP00000351157	2	TXNDC15
ENSP00000419204	2	TTC6
ENSP00000007390	2	TSR3
ENSP00000398163	2	TSPY3
ENSP00000305524	2	TSEN34
ENSP00000327738	2	TRIML1
ENSP00000327604	2	TRIM49
ENSP00000365844	2	TRIM39
ENSP00000394371	2	TRIM26
ENSP00000374898	2	TRBV5-6
ENSP00000357591	2	TRAPPC3L
ENSP00000301021	2	TRAPPC2L
ENSP00000309402	2	TRAM1L1
ENSP00000352265	2	TPGS1
ENSP00000331827	2	TNFAIP8L1
ENSP00000375859	2	TMEM91
ENSP00000335416	2	TMEM68

ENSP00000260403	2	TMEM62
ENSP00000261234	2	TMEM5
ENSP00000363824	2	TMEM38B
ENSP00000303987	2	TMEM223
ENSP00000305892	2	TMEM208
ENSP00000358999	2	TMEM187
ENSP00000004103	2	TMEM176A
ENSP00000296595	2	TMEM161B
ENSP00000352284	2	TMEM139
ENSP00000299705	2	TMED3
ENSP00000389399	2	TMCO3
ENSP00000343635	2	TLDC1
ENSP00000257245	2	TIMM10
ENSP00000339532	2	THUMPD3
ENSP00000305533	2	THAP9
ENSP00000307142	2	TEX37
ENSP00000331500	2	TEX19
ENSP00000265993	2	TCTN3
ENSP00000240619	2	TAS2R10
ENSP00000372722	2	TAP1
ENSP00000402316	2	TAP1
ENSP00000405356	2	TAP1
ENSP00000412933	2	TAP1
ENSP00000413080	2	TAP1
ENSP00000415660	2	TAP1
ENSP00000332721	2	TANGO2
ENSP00000236273	2	SYF2
ENSP00000299134	2	SVOP
ENSP00000363388	2	SUSD1
ENSP00000320679	2	STX19
ENSP00000311257	2	STOX2
ENSP00000380858	2	ST7-OT4
ENSP00000011691	2	SS18L2
ENSP00000342628	2	SPDYE2
ENSP00000414920	2	SPATA24
ENSP00000312774	2	SPACA4
ENSP00000279034	2	SOGA1
ENSP00000323435	2	SNX22
ENSP00000417806	2	SMIM4
ENSP00000295958	2	SMIM14
ENSP00000271227	2	SLC44A3
ENSP00000266980	2	SLC39A5
ENSP00000316596	2	SLC38A9
ENSP00000265836	2	SLC35F2
ENSP00000368981	2	SLC35B3
ENSP00000242275	2	SLC25A51

ENSP00000354886	2	SLC25A47
ENSP00000311856	2	SLC25A30
ENSP00000364543	2	SKIV2L
ENSP00000262861	2	SIPA1L2
ENSP00000342075	2	SIMC1
ENSP00000272433	2	SFXN5
ENSP00000356541	2	SF3B5
ENSP00000359370	2	SEC31B
ENSP00000310521	2	SEC22A
ENSP00000355177	2	RUSC2
ENSP00000364749	2	RSG1
ENSP00000250784	2	RPS4Y1
ENSP00000211372	2	RPS18
ENSP00000393241	2	RPS18
ENSP00000412583	2	RPS18
ENSP00000416110	2	RPS18
ENSP00000355315	2	RPL39
ENSP00000322832	2	ROGDI
ENSP00000389709	2	RNF212
ENSP00000306396	2	RNF187
ENSP00000364263	2	RNF186
ENSP00000420740	2	RNF183
ENSP00000360455	2	RHOXF2B
ENSP00000310335	2	REP15
ENSP00000318415	2	RBMXL1
ENSP00000267229	2	RBM26
ENSP00000358549	2	RARS2
ENSP00000333456	2	RAI2
ENSP00000264160	2	R3HDM1
ENSP00000308258	2	PTDSS2
ENSP00000412027	2	PSMB9
ENSP00000364016	2	PSMB8
ENSP00000372723	2	PSMB8
ENSP00000394155	2	PSMB8
ENSP00000402406	2	PSMB8
ENSP00000404585	2	PSMB8
ENSP00000414770	2	PSMB8
ENSP00000161006	2	PRSS22
ENSP00000351608	2	PRRT2
ENSP00000228811	2	PRR4
ENSP00000412456	2	PPP1R18
ENSP00000407981	2	PPP1R11
ENSP00000254765	2	POPDC3
ENSP00000280800	2	PLBD2
ENSP00000290431	2	PKD2L2
ENSP00000078527	2	PIGV

ENSP00000309430	2	PIGS
ENSP00000309230	2	PEAK1
ENSP00000266395	2	PDE6H
ENSP00000353739	2	PCMTD1
ENSP00000354293	2	PCDHB16
ENSP00000228820	2	PARP11
ENSP00000330808	2	OTOL1
ENSP00000323606	2	OR6C2
ENSP00000359464	2	OGFRL1
ENSP00000230792	2	NUDT12
ENSP00000401321	2	NOTCH4
ENSP00000370949	2	NFXL1
ENSP00000002125	2	NDUFAF7
ENSP00000379430	2	NDUFAF6
ENSP00000301457	2	NDUFA7
ENSP00000252711	2	NDUFA10
ENSP00000361454	2	NCBP2L
ENSP00000399903	2	NBEAL1
ENSP00000362171	2	NAP1L3
ENSP00000311500	2	MZT2A
ENSP00000348050	2	MXRA7
ENSP00000241527	2	MTERFD2
ENSP00000239614	2	MSANTD2
ENSP00000338648	2	MS4A4A
ENSP00000318158	2	MRPS24
ENSP00000295491	2	MRPS18C
ENSP00000355692	2	MRPL55
ENSP00000363999	2	MRPL50
ENSP00000258383	2	MRPL44
ENSP00000333401	2	MRPL40
ENSP00000354086	2	MRPL37
ENSP00000225969	2	MRPL27
ENSP00000341082	2	MRPL20
ENSP00000288937	2	MRPL17
ENSP00000324100	2	MRPL10
ENSP00000262966	2	MPND
ENSP00000344551	2	MORN2
ENSP00000223114	2	MOGAT3
ENSP00000306220	2	MMGT1
ENSP00000264968	2	MGAT4A
ENSP00000330051	2	MFSD6L
ENSP00000362707	2	MANBAL
ENSP00000348959	2	MAN1A2
ENSP00000314518	2	LYSMD3
ENSP00000397708	2	LY6G6D
ENSP00000372921	2	LY6G5B

ENSP00000398952	2	LY6G5B
ENSP00000403495	2	LTA
ENSP00000332103	2	LSMD1
ENSP00000296581	2	LSM6
ENSP00000364813	2	LSM2
ENSP00000319341	2	LSM10
ENSP00000342188	2	LRRIQ4
ENSP00000411568	2	LRRD1
ENSP00000339047	2	LRRC61
ENSP00000295628	2	LRRC58
ENSP00000260382	2	LRRC49
ENSP00000344470	2	LRRC39
ENSP00000372959	2	LRRC30
ENSP00000007969	2	LRRC23
ENSP00000285928	2	LRGUK
ENSP00000406478	2	LILRB5
ENSP00000331647	2	LENG9
ENSP00000366955	2	KRTAP4-2
ENSP00000381489	2	KRTAP4-1
ENSP00000375429	2	KRTAP3-2
ENSP00000381494	2	KRTAP2-2
ENSP00000334985	2	KRTAP19-5
ENSP00000386376	2	KRTAP19-3
ENSP00000375478	2	KRTAP10-3
ENSP00000306261	2	KRT78
ENSP00000312397	2	KLHL3
ENSP00000270583	2	KLHDC4
ENSP00000346401	2	KIF25
ENSP00000055682	2	KIAA2022
ENSP00000356560	2	KIAA1614
ENSP00000409964	2	KIAA1430
ENSP00000261588	2	KIAA0556
ENSP00000251343	2	KHNYN
ENSP00000362836	2	KCTD20
ENSP00000321544	2	KBTBD11
ENSP00000369989	2	KATNAL1
ENSP00000274766	2	KAAG1
ENSP00000348648	2	JPH4
ENSP00000298622	2	JAKMIP3
ENSP00000306106	2	JAGN1
ENSP00000256861	2	ITIH5
ENSP00000336861	2	IQCH
ENSP00000374842	2	IGLV3-22
ENSP00000374847	2	IGLV2-14
ENSP00000374799	2	IGKV2-40
ENSP00000418903	2	IGKV1-27

ENSP00000395656	2	IGHV4-31
ENSP00000419583	2	IGHD3-9
ENSP00000418018	2	IFNE
ENSP00000361507	2	HYI
ENSP00000338387	2	HLCS
ENSP00000414360	2	HLA-DQA1
ENSP00000395780	2	HLA-DOB
ENSP00000405108	2	HLA-DOB
ENSP00000378723	2	HLA-DMB
ENSP00000333277	2	HIST2H3D
ENSP00000359171	2	HIAT1
ENSP00000388322	2	HHLA1
ENSP00000297440	2	HEATR2
ENSP00000238379	2	HDHD3
ENSP00000320838	2	GSG1
ENSP00000364580	2	GRTP1
ENSP00000367378	2	GPR34
ENSP00000302676	2	GPR22
ENSP00000366970	2	GPR20
ENSP00000324553	2	GPR156
ENSP00000390746	2	GPANK1
ENSP00000411162	2	GNL1
ENSP00000315925	2	GMPPA
ENSP00000343890	2	GLTPD1
ENSP00000296805	2	GFM2
ENSP00000369895	2	GEMIN8
ENSP00000317027	2	GCNT4
ENSP00000294671	2	GBP7
ENSP00000308591	2	GAL3ST3
ENSP00000313309	2	FUZ
ENSP00000367284	2	FUNDC1
ENSP00000366814	2	FOXD4L2
ENSP00000410658	2	FLOT1
ENSP00000398812	2	FKBPL
ENSP00000238256	2	FKBP15
ENSP00000363529	2	FAM83C
ENSP00000420140	2	FAM71F2
ENSP00000263384	2	FAM32A
ENSP00000337541	2	FAM21C
ENSP00000351259	2	FAM21B
ENSP00000366901	2	FAM216A
ENSP00000319897	2	FAM214B
ENSP00000342604	2	FAM184A
ENSP00000333553	2	FAM178B
ENSP00000219535	2	FAM173A
ENSP00000379294	2	FAM172A

ENSP00000357545	2	FAM162B
ENSP00000194672	2	FAM135A
ENSP00000307821	2	FADS6
ENSP00000325674	2	EXOC3L1
ENSP00000370039	2	EML5
ENSP00000253457	2	EMC8
ENSP00000267750	2	EMC4
ENSP00000420608	2	EMC1
ENSP00000354722	2	EIF1AY
ENSP00000361001	2	EFCAB14
ENSP00000326267	2	EFCAB11
ENSP00000255108	2	DPH2
ENSP00000361625	2	DOLPP1
ENSP00000363412	2	DNAJC25-GNG10
ENSP00000320650	2	DNAJC25
ENSP00000342012	2	DMKN
ENSP00000315569	2	DIS3L2
ENSP00000364943	2	DDAH2
ENSP00000403154	2	DDAH2
ENSP00000409075	2	CYB5RL
ENSP00000299498	2	CYB5R2
ENSP00000360652	2	CXorf56
ENSP00000360961	2	CT83
ENSP00000246020	2	CSTL1
ENSP00000374562	2	COX16
ENSP00000373419	2	CORO6
ENSP00000339168	2	COLEC11
ENSP00000355572	2	COA6
ENSP00000364934	2	CLIC1
ENSP00000372896	2	CLIC1
ENSP00000404589	2	CLIC1
ENSP00000406335	2	CLIC1
ENSP00000409247	2	CLIC1
ENSP00000355319	2	CIPC
ENSP00000262570	2	CHCHD3
ENSP00000361923	2	CHCHD1
ENSP00000389427	2	CEP44
ENSP00000365075	2	CELA2B
ENSP00000383895	2	CEBPZ-AS1
ENSP00000384887	2	CEACAM19
ENSP00000303178	2	CDY1B
ENSP00000359403	2	CD99L2
ENSP00000321005	2	CD300LG
ENSP00000329942	2	CD300E
ENSP00000363139	2	CD164L2
ENSP00000377978	2	CCL4L2

ENSP00000383263	2	CCHCR1
ENSP00000262962	2	CCDC94
ENSP00000303158	2	CCDC8
ENSP00000242819	2	CCDC70
ENSP00000291458	2	CCDC58
ENSP00000344655	2	CCDC41
ENSP00000380679	2	CCDC40
ENSP00000361392	2	CCDC24
ENSP00000356779	2	CCDC181
ENSP00000373566	2	CCDC158
ENSP00000290418	2	CCDC142
ENSP00000316237	2	CCDC102B
ENSP00000360642	2	CC2D1B
ENSP00000348915	2	CBWD1
ENSP00000369339	2	C9orf72
ENSP00000354812	2	C9orf114
ENSP00000297534	2	C7orf55
ENSP00000386450	2	C7orf34
ENSP00000365076	2	C6orf47
ENSP00000310951	2	C6orf203
ENSP00000398496	2	C6orf15
ENSP00000368503	2	C4orf46
ENSP00000320251	2	C3orf17
ENSP00000217195	2	C20orf27
ENSP00000341213	2	C20orf24
ENSP00000392835	2	C2
ENSP00000330426	2	C1QTNF8
ENSP00000253407	2	C1QL1
ENSP00000367808	2	C1orf86
ENSP00000355840	2	C1orf65
ENSP00000358965	2	C1orf194
ENSP00000355306	2	C1orf174
ENSP00000221576	2	C19orf53
ENSP00000270502	2	C19orf52
ENSP00000323199	2	C18orf32
ENSP00000396936	2	C17orf72
ENSP00000370770	2	C17orf107
ENSP00000386218	2	C16orf3
ENSP00000307071	2	C15orf40
ENSP00000280749	2	C12orf45
ENSP00000333845	2	C11orf88
ENSP00000331209	2	C11orf54
ENSP00000346600	2	C11orf48
ENSP00000354481	2	BRINP2
ENSP00000306752	2	BOLA2B
ENSP00000345773	2	BEND7

ENSP00000371085	2	BAIAP2L2
ENSP00000316173	2	B3GNT5
ENSP00000309096	2	B3GNT1
ENSP00000363416	2	AUNIP
ENSP00000417190	2	ATPAF2
ENSP00000361005	2	ATPAF1
ENSP00000281087	2	ATP6V1G3
ENSP00000339182	2	ATP13A4
ENSP00000328327	2	ASB7
ENSP00000245543	2	ARMC7
ENSP00000266594	2	ANP32D
ENSP00000292246	2	ANO10
ENSP00000286195	2	ALS2CR11
ENSP00000245812	2	ALKBH7
ENSP00000361083	2	ADIRF
ENSP00000315118	2	ADCK4
ENSP00000407761	2	ACOXL
ENSP00000289119	2	ABHD3
ENSP00000413319	2	ABCF1
ENSP00000311030	2	ABCC12
ENSP00000367528	2	AARD
ENSP00000203166	2	-
ENSP00000217256	2	-
ENSP00000222956	2	-
ENSP00000240349	2	-
ENSP00000246000	2	-
ENSP00000252816	2	-
ENSP00000285176	2	-
ENSP00000295220	2	-
ENSP00000296441	2	-
ENSP00000299377	2	-
ENSP00000307908	2	-
ENSP00000308540	2	-
ENSP00000314978	2	-
ENSP00000316547	2	-
ENSP00000320445	2	-
ENSP00000330785	2	-
ENSP00000332724	2	-
ENSP00000333727	2	-
ENSP00000343452	2	-
ENSP00000349197	2	-
ENSP00000350479	2	-
ENSP00000351543	2	-
ENSP00000354548	2	-
ENSP00000354717	2	-
ENSP00000355345	2	-

ENSP00000360658	2	-
ENSP00000364192	2	-
ENSP00000369893	2	-
ENSP00000370107	2	-
ENSP00000375160	2	-
ENSP00000381576	2	-
ENSP00000381835	2	-
ENSP00000382756	2	-
ENSP00000383069	2	-
ENSP00000383298	2	-
ENSP00000386061	2	-
ENSP00000387338	2	-
ENSP00000387412	2	-
ENSP00000387431	2	-
ENSP00000390130	2	-
ENSP00000392723	2	-
ENSP00000392797	2	-
ENSP00000393111	2	-
ENSP00000393427	2	-
ENSP00000395015	2	-
ENSP00000395470	2	-
ENSP00000395511	2	-
ENSP00000395543	2	-
ENSP00000395886	2	-
ENSP00000397100	2	-
ENSP00000397447	2	-
ENSP00000397801	2	-
ENSP00000397823	2	-
ENSP00000398427	2	-
ENSP00000399049	2	-
ENSP00000399321	2	-
ENSP00000399350	2	-
ENSP00000399885	2	-
ENSP00000399932	2	-
ENSP00000401087	2	-
ENSP00000401574	2	-
ENSP00000402757	2	-
ENSP00000403246	2	-
ENSP00000403626	2	-
ENSP00000403936	2	-
ENSP00000404000	2	-
ENSP00000404663	2	-
ENSP00000405856	2	-
ENSP00000406635	2	-
ENSP00000407452	2	-
ENSP00000407765	2	-

ENSP00000409189	2	-
ENSP00000409319	2	-
ENSP00000410207	2	-
ENSP00000410885	2	-
ENSP00000413020	2	-
ENSP00000413440	2	-
ENSP00000414346	2	-
ENSP00000415385	2	-
ENSP00000415985	2	-
ENSP00000416022	2	-
ENSP00000252207	1	ZSCAN9
ENSP00000325123	1	ZSCAN2
ENSP00000366527	1	ZSCAN16
ENSP00000399863	1	ZNF839
ENSP00000329638	1	ZNF804B
ENSP00000270459	1	ZNF787
ENSP00000348673	1	ZNF770
ENSP00000269394	1	ZNF750
ENSP00000328245	1	ZNF71
ENSP00000301093	1	ZNF701
ENSP00000339314	1	ZNF70
ENSP00000345333	1	ZNF69
ENSP00000342818	1	ZNF669
ENSP00000379702	1	ZNF665
ENSP00000322265	1	ZNF620
ENSP00000411409	1	ZNF598
ENSP00000301744	1	ZNF597
ENSP00000410734	1	ZNF562
ENSP00000302603	1	ZNF556
ENSP00000296091	1	ZNF502
ENSP00000388421	1	ZNF487
ENSP00000261560	1	ZNF430
ENSP00000347045	1	ZNF43
ENSP00000255616	1	ZNF414
ENSP00000299687	1	ZNF407
ENSP00000340132	1	ZNF383
ENSP00000364405	1	ZNF367
ENSP00000341528	1	ZNF34
ENSP00000379208	1	ZNF248
ENSP00000211936	1	ZNF184
ENSP00000302455	1	ZNF17
ENSP00000337081	1	ZNF112
ENSP00000383563	1	ZG16
ENSP00000301318	1	ZFP28
ENSP00000413418	1	ZFHX2
ENSP00000341681	1	ZDHHC7

ENSP00000362465	1	ZDHHC15
ENSP00000381109	1	ZCWPW1
ENSP00000278590	1	ZC3H12C
ENSP00000408077	1	ZC3H12B
ENSP00000238686	1	ZC2HC1C
ENSP00000411491	1	ZBTB47
ENSP00000300022	1	YPEL4
ENSP00000312272	1	YPEL2
ENSP00000412696	1	YIPF7
ENSP00000289953	1	WFDC8
ENSP00000243938	1	WFDC3
ENSP00000262144	1	WDR59
ENSP00000379201	1	VCPKMT
ENSP00000362105	1	UTP11L
ENSP00000301712	1	UNKL
ENSP00000346627	1	TYW5
ENSP00000359904	1	TYW3
ENSP00000287078	1	TYSND1
ENSP00000299866	1	TVP23A
ENSP00000365105	1	TTLL9
ENSP00000325266	1	TTC9C
ENSP00000307640	1	TTC36
ENSP00000386181	1	TTC30B
ENSP00000347915	1	TTC30A
ENSP00000316740	1	TTC29
ENSP00000362255	1	TSPO2
ENSP00000329858	1	TRMT12
ENSP00000414258	1	TRIM40
ENSP00000391917	1	TRIM39
ENSP00000409605	1	TRIM15
ENSP00000397073	1	TRIM10
ENSP00000374869	1	TRGV3
ENSP00000374896	1	TRBV6-7
ENSP00000420669	1	TRBJ2-1
ENSP00000330289	1	TRAPPC6B
ENSP00000316990	1	TRAPPC5
ENSP00000409231	1	TRAPPC13
ENSP00000324318	1	TRAPPC12
ENSP00000341677	1	TPD52L3
ENSP00000354204	1	TOMM20L
ENSP00000328016	1	TNFAIP8L3
ENSP00000297533	1	TMUB1
ENSP00000321038	1	TMEM86B
ENSP00000297459	1	TMEM74
ENSP00000374234	1	TMEM72
ENSP00000234831	1	TMEM59

ENSP00000187762	1	TMEM38A
ENSP00000350961	1	TMEM261
ENSP00000386149	1	TMEM257
ENSP00000266673	1	TMEM19
ENSP00000323068	1	TMEM168
ENSP00000229563	1	TMEM14C
ENSP00000306344	1	TMEM135
ENSP00000312615	1	TMEM134
ENSP00000345152	1	TMEM120B
ENSP00000365776	1	TMC4
ENSP00000418803	1	TIMMDC1
ENSP00000317170	1	TIGD2
ENSP00000249861	1	THAP10
ENSP00000318502	1	TGIF2LY
ENSP00000355119	1	TGIF2LX
ENSP00000358957	1	TEX28P2
ENSP00000399753	1	TEX13A
ENSP00000283025	1	TEKT5
ENSP00000352510	1	TECPR2
ENSP00000357796	1	TCHHL1
ENSP00000414980	1	TCF19
ENSP00000345014	1	TBC1D3G
ENSP00000381781	1	TBC1D3B
ENSP00000229088	1	TBC1D30
ENSP00000336724	1	TBC1D22A
ENSP00000346139	1	TBC1D20
ENSP00000361731	1	TBC1D13
ENSP00000338093	1	TARSL2
ENSP00000372599	1	TAP2
ENSP00000372726	1	TAP2
ENSP00000316130	1	SYNE4
ENSP00000344219	1	SUSD4
ENSP00000337926	1	SUGP2
ENSP00000327509	1	STAC2
ENSP00000321343	1	ST8SIA5
ENSP00000267260	1	SRRM4
ENSP00000347721	1	SPNS3
ENSP00000369080	1	SPEF1
ENSP00000351503	1	SPATS2L
ENSP00000331532	1	SPATA32
ENSP00000347153	1	SPATA31A7
ENSP00000299140	1	SPATA19
ENSP00000418593	1	SNX21
ENSP00000221573	1	SNAPC2
ENSP00000390750	1	SMIM19
ENSP00000339324	1	SMIM15

ENSP00000399161	1	SLC44A4
ENSP00000219320	1	SLC38A7
ENSP00000363891	1	SLC38A10
ENSP00000311833	1	SLC37A2
ENSP00000366942	1	SLC36A3
ENSP00000342518	1	SLC35F4
ENSP00000355577	1	SLC35F3
ENSP00000333591	1	SLC35D3
ENSP00000273158	1	SLC25A38
ENSP00000306328	1	SLC25A33
ENSP00000334594	1	SLC10A7
ENSP00000321936	1	SH3D21
ENSP00000347924	1	SFXN4
ENSP00000264454	1	SEC22C
ENSP00000255858	1	SEC14L4
ENSP00000215812	1	SEC14L3
ENSP00000332407	1	SDR42E1
ENSP00000248958	1	SDF2L1
ENSP00000403346	1	SAPCD1
ENSP00000356444	1	SAMD5
ENSP00000335397	1	RTN4RL2
ENSP00000362894	1	RTKN2
ENSP00000288666	1	RPS4Y2
ENSP00000317691	1	RPP25
ENSP00000412619	1	RPP21
ENSP00000415046	1	RPP21
ENSP00000346063	1	RPL7L1
ENSP00000252543	1	RPL36
ENSP00000386717	1	RPL31
ENSP00000240159	1	RNF170
ENSP00000269391	1	RNF157
ENSP00000331734	1	RIPPLY3
ENSP00000386588	1	RGPD3
ENSP00000332727	1	RGPD2
ENSP00000409466	1	RGP1
ENSP00000295049	1	RFTN2
ENSP00000293273	1	RDM1
ENSP00000321179	1	RBM44
ENSP00000354884	1	RASSF9
ENSP00000356329	1	RAET1G
ENSP00000382030	1	RAD51AP2
ENSP00000217043	1	R3HDML
ENSP00000365837	1	PUS1
ENSP00000372721	1	PSMB9
ENSP00000393744	1	PSMB9
ENSP00000407233	1	PSMB9

ENSP00000407810	1	PSMB9
ENSP00000386212	1	PSMB11
ENSP00000358217	1	PRDM13
ENSP00000365343	1	PRAMEF3
ENSP00000398462	1	PPT2
ENSP00000411038	1	PPP1R11
ENSP00000359198	1	PPDPF
ENSP00000216177	1	PNPLA5
ENSP00000321116	1	PNPLA1
ENSP00000405718	1	PNMA6A
ENSP00000367655	1	PKHD1L1
ENSP00000413405	1	PIGZ
ENSP00000317301	1	PIGX
ENSP00000331643	1	PGBD2
ENSP00000372694	1	PFDN6
ENSP00000412319	1	PFDN6
ENSP00000415678	1	PFDN6
ENSP00000261813	1	PFDN1
ENSP00000274569	1	PCYOX1L
ENSP00000304192	1	PCNX
ENSP00000306918	1	PCDHGC4
ENSP00000411439	1	PATE4
ENSP00000395505	1	PATE3
ENSP00000351325	1	PATE2
ENSP00000281631	1	PARP8
ENSP00000238714	1	PAPOLG
ENSP00000239231	1	PANK3
ENSP00000308012	1	PABPC5
ENSP00000362621	1	PABPC1L2B
ENSP00000362618	1	PABPC1L2A
ENSP00000363873	1	OXLD1
ENSP00000285420	1	OTUD6B
ENSP00000339389	1	OTUD6A
ENSP00000382323	1	OTOG
ENSP00000349052	1	OSCP1
ENSP00000323928	1	OR8H3
ENSP00000323982	1	OR8H2
ENSP00000322435	1	OR6B3
ENSP00000412591	1	OR5V1
ENSP00000367650	1	OR5L2
ENSP00000335529	1	OR5L1
ENSP00000393889	1	OR5K2
ENSP00000373193	1	OR5K1
ENSP00000330338	1	OR52L1
ENSP00000318956	1	OR52K2
ENSP00000302422	1	OR52K1

ENSP00000308764	1	OR52I2
ENSP00000369725	1	OR52A1
ENSP00000369731	1	OR51A4
ENSP00000369729	1	OR51A2
ENSP00000329467	1	OR4M2
ENSP00000319654	1	OR4M1
ENSP00000334393	1	OR4F5
ENSP00000317482	1	OR4F4
ENSP00000409316	1	OR4F29
ENSP00000315047	1	OR4F17
ENSP00000412752	1	OR4A47
ENSP00000325065	1	OR4A15
ENSP00000291231	1	OR3A3
ENSP00000386180	1	OR3A2
ENSP00000412537	1	OR2W1
ENSP00000326225	1	OR2T8
ENSP00000355429	1	OR2T5
ENSP00000355431	1	OR2T4
ENSP00000330904	1	OR2T34
ENSP00000352604	1	OR2T3
ENSP00000343062	1	OR2T2
ENSP00000324583	1	OR2T12
ENSP00000355432	1	OR2M5
ENSP00000389625	1	OR2M3
ENSP00000352710	1	OR2M2
ENSP00000349719	1	OR2L8
ENSP00000353044	1	OR2L3
ENSP00000355435	1	OR2L2
ENSP00000408783	1	OR2J3
ENSP00000416299	1	OR2J2
ENSP00000414688	1	OR2H2
ENSP00000383549	1	OR2H1
ENSP00000407739	1	OR2H1
ENSP00000386222	1	OR2F2
ENSP00000403571	1	OR2B3
ENSP00000420502	1	OR2A7
ENSP00000319546	1	OR2A4
ENSP00000386175	1	OR2A1
ENSP00000305469	1	OR1S2
ENSP00000311688	1	OR1S1
ENSP00000362788	1	OR1L6
ENSP00000259466	1	OR1L4
ENSP00000248384	1	OR1E2
ENSP00000408647	1	OR14J1
ENSP00000367219	1	OR13J1
ENSP00000334452	1	OR13F1

ENSP00000317357	1	OR13D1
ENSP00000259362	1	OR13C9
ENSP00000334068	1	OR13C8
ENSP00000363911	1	OR13C5
ENSP00000277216	1	OR13C4
ENSP00000363913	1	OR13C3
ENSP00000415065	1	OR12D2
ENSP00000348033	1	OR11L1
ENSP00000319071	1	OR11H6
ENSP00000318997	1	OR11H4
ENSP00000383532	1	OR11A1
ENSP00000412097	1	OR11A1
ENSP00000354707	1	OR10Z1
ENSP00000357132	1	OR10X1
ENSP00000310704	1	OR10H5
ENSP00000335596	1	OR10H1
ENSP00000364164	1	OR10G9
ENSP00000329689	1	OR10G7
ENSP00000419119	1	OR10C1
ENSP00000299454	1	OR10A5
ENSP00000303862	1	OR10A2
ENSP00000257627	1	OCM2
ENSP00000370873	1	OCIAD2
ENSP00000361835	1	NXF2B
ENSP00000301490	1	NUDT8
ENSP00000287706	1	NTAN1
ENSP00000331843	1	NPIPA1
ENSP00000382274	1	NOMO3
ENSP00000291971	1	NLRP8
ENSP00000362781	1	NHSL2
ENSP00000362058	1	NDUFS5
ENSP00000265500	1	NDUFC1
ENSP00000377770	1	NDUFC1
ENSP00000377411	1	NDUFA4L2
ENSP00000311740	1	NDUFA11
ENSP00000298406	1	NAA30
ENSP00000304147	1	MYEOV2
ENSP00000349678	1	MYBPHL
ENSP00000363418	1	MTFR1L
ENSP00000293892	1	MSLNL
ENSP00000304713	1	MSANTD4
ENSP00000300184	1	MS4A7
ENSP00000300190	1	MS4A5
ENSP00000382250	1	MRPS6
ENSP00000315397	1	MRPS31
ENSP00000258105	1	MRPL53

ENSP00000308901	1	MRPL45
ENSP00000296102	1	MRPL33
ENSP00000373404	1	MRPL2
ENSP00000310726	1	MRP63
ENSP00000307636	1	MORN4
ENSP00000259891	1	MOG
ENSP00000390632	1	MOG
ENSP00000397101	1	MOG
ENSP00000215582	1	MISP
ENSP00000379057	1	MIEF2
ENSP00000416015	1	MICAL3
ENSP00000350353	1	METTL20
ENSP00000409167	1	MDC1
ENSP00000368468	1	MCUR1
ENSP00000314776	1	MBLAC2
ENSP00000299952	1	MARVELD3
ENSP00000368320	1	MAGEB6
ENSP00000368315	1	MAGEB5
ENSP00000364650	1	LYZL1
ENSP00000370761	1	LYRM5
ENSP00000387077	1	LYPD6B
ENSP00000334463	1	LYPD6
ENSP00000382956	1	LY6G6D
ENSP00000410321	1	LY6G5C
ENSP00000361048	1	LURAP1
ENSP00000410481	1	LTB
ENSP00000416509	1	LTA
ENSP00000368135	1	LRRN4
ENSP00000400803	1	LRRC69
ENSP00000326817	1	LRRC57
ENSP00000291592	1	LRRC3
ENSP00000292524	1	LRRC14
ENSP00000325091	1	LRCH2
ENSP00000262134	1	LPCAT2
ENSP00000266604	1	LLPH
ENSP00000222224	1	LENG1
ENSP00000356122	1	LEMD1
ENSP00000340434	1	LDOC1L
ENSP00000317619	1	LACC1
ENSP00000332805	1	KRTAP8-1
ENSP00000332690	1	KRTAP6-1
ENSP00000382584	1	KRTAP5-5
ENSP00000382590	1	KRTAP5-4
ENSP00000371606	1	KRTAP5-1
ENSP00000375151	1	KRTAP4-3
ENSP00000339238	1	KRTAP24-1

ENSP00000377583	1	KRTAP2-4
ENSP00000375238	1	KRTAP2-1
ENSP00000375147	1	KRTAP16-1
ENSP00000347635	1	KRTAP13-1
ENSP00000375476	1	KRTAP12-4
ENSP00000381005	1	KRTAP12-3
ENSP00000375475	1	KRTAP12-1
ENSP00000305975	1	KRTAP1-1
ENSP00000334197	1	KRTAP10-11
ENSP00000369438	1	KRTAP10-10
ENSP00000383226	1	KRTAP10-1
ENSP00000307014	1	KRT73
ENSP00000305263	1	KRT28
ENSP00000219837	1	KNOP1
ENSP00000358265	1	KLHL32
ENSP00000300976	1	KLHL26
ENSP00000336800	1	KLHL25
ENSP00000232766	1	KLHL18
ENSP00000332791	1	KLHL15
ENSP00000265529	1	KIF9
ENSP00000298569	1	KIAA1191
ENSP00000359392	1	KHDC3L
ENSP00000359415	1	KHDC1L
ENSP00000415106	1	KBTBD4
ENSP00000399475	1	JMJD8
ENSP00000380467	1	JMJD7
ENSP00000273691	1	ILDR1
ENSP00000410629	1	IGSF23
ENSP00000374825	1	IGLV1-51
ENSP00000420118	1	IGKV3-11
ENSP00000419058	1	IGKV1-39
ENSP00000375048	1	IGHV7-81
ENSP00000375036	1	IGHV3-53
ENSP00000375022	1	IGHV3-30
ENSP00000375018	1	IGHV3-23
ENSP00000334714	1	IGFN1
ENSP00000398139	1	IER3
ENSP00000355672	1	IBA57
ENSP00000372881	1	HSPA1L
ENSP00000301785	1	HNRNPUL2
ENSP00000248098	1	HN1L
ENSP00000287701	1	HMBOX1
ENSP00000383521	1	HLA-G
ENSP00000259951	1	HLA-F
ENSP00000372608	1	HLA-DRA
ENSP00000402951	1	HLA-DRA

ENSP00000410443	1	HLA-DRA
ENSP00000339398	1	HLA-DQA1
ENSP00000389288	1	HLA-DPB1
ENSP00000407674	1	HLA-DPB1
ENSP00000397139	1	HLA-DPA1
ENSP00000406250	1	HLA-DPA1
ENSP00000410390	1	HLA-DOB
ENSP00000401504	1	HLA-DOA
ENSP00000416448	1	HLA-DOA
ENSP00000408453	1	HLA-DMB
ENSP00000413471	1	HLA-DMB
ENSP00000414817	1	HLA-DMB
ENSP00000416233	1	HLA-A
ENSP00000358153	1	HIST2H4B
ENSP00000366618	1	HIST1H2BL
ENSP00000396452	1	HDHD1
ENSP00000354723	1	H2BFWT
ENSP00000261047	1	GUCA1C
ENSP00000285689	1	GRAMD3
ENSP00000372752	1	GPSM3
ENSP00000414024	1	GPSM3
ENSP00000356658	1	GPR52
ENSP00000270590	1	GPR32
ENSP00000264080	1	GPR108
ENSP00000363250	1	GPN2
ENSP00000401272	1	GPANK1
ENSP00000308535	1	GOLT1A
ENSP00000399637	1	GOLGA8I
ENSP00000359634	1	GOLGA7B
ENSP00000418001	1	GK5
ENSP00000358451	1	GDAP2
ENSP00000341782	1	GDAP1L1
ENSP00000353142	1	GAL3ST4
ENSP00000206595	1	G2E3
ENSP00000332757	1	FUT10
ENSP00000401306	1	FSIP2
ENSP00000216187	1	FOXRED2
ENSP00000416935	1	FOLR4
ENSP00000264703	1	FNDC4
ENSP00000359498	1	FMR1NB
ENSP00000364298	1	FKBPL
ENSP00000412439	1	FKBPL
ENSP00000374328	1	FAXC
ENSP00000395848	1	FAM90A26
ENSP00000307798	1	FAM90A1
ENSP00000356096	1	FAM72A

ENSP00000344331	1	FAM69C
ENSP00000042381	1	FAM65A
ENSP00000289166	1	FAM46B
ENSP00000343115	1	FAM222B
ENSP00000351783	1	FAM222A
ENSP00000291634	1	FAM207A
ENSP00000366623	1	FAM203B
ENSP00000321320	1	FAM203A
ENSP00000377396	1	FAM198B
ENSP00000410098	1	FAM193B
ENSP00000267594	1	FAM181A
ENSP00000367356	1	FAM171A1
ENSP00000349336	1	FAM163B
ENSP00000309432	1	FAM134C
ENSP00000335040	1	FAM129C
ENSP00000341597	1	FAM114A2
ENSP00000307181	1	FAM103A1
ENSP00000340474	1	EXD3
ENSP00000233712	1	EVA1A
ENSP00000272342	1	ETAA1
ENSP00000339115	1	ESPNL
ENSP00000355735	1	ERMARD
ENSP00000300714	1	ENTHD2
ENSP00000230565	1	ENPP5
ENSP00000345555	1	ENO4
ENSP00000368517	1	ECHDC3
ENSP00000370022	1	EBF4
ENSP00000396593	1	DYTN
ENSP00000312224	1	DTD2
ENSP00000377523	1	DQX1
ENSP00000315988	1	DPY19L2
ENSP00000360803	1	DNLZ
ENSP00000378605	1	DNAJC30
ENSP00000372005	1	DNAJC19
ENSP00000269945	1	DMRTC2
ENSP00000349893	1	DLK2
ENSP00000280056	1	DHRS12
ENSP00000370889	1	DENND1C
ENSP00000312702	1	DEFB114
ENSP00000333234	1	DEFB108B
ENSP00000372136	1	DEFA1B
ENSP00000399542	1	DDX39B
ENSP00000417748	1	DDI2
ENSP00000299340	1	CYYR1
ENSP00000381086	1	CXorf36
ENSP00000326411	1	CWF19L1

ENSP00000260327	1	CTDSPL2
ENSP00000276241	1	CT55
ENSP00000396772	1	CSNK2B
ENSP00000182096	1	CRYBG3
ENSP00000262207	1	CRISPLD1
ENSP00000296953	1	CREBRF
ENSP00000343900	1	CPSF4L
ENSP00000263960	1	COQ10B
ENSP00000312587	1	COQ10A
ENSP00000299886	1	COG1
ENSP00000273308	1	CNPY2
ENSP00000389881	1	CNGA1
ENSP00000335605	1	CMTM7
ENSP00000205636	1	CMTM6
ENSP00000385898	1	CLPSL2
ENSP00000272367	1	CLEC4F
ENSP00000353013	1	CLEC14A
ENSP00000305204	1	CKAP2L
ENSP00000269878	1	CIB3
ENSP00000317404	1	CHST13
ENSP00000299295	1	CHODL
ENSP00000338838	1	CHID1
ENSP00000290913	1	CHCHD6
ENSP00000418428	1	CHCHD10
ENSP00000295304	1	CHAC2
ENSP00000387209	1	CEP19
ENSP00000367476	1	CEP104
ENSP00000393854	1	CENPBD1
ENSP00000329318	1	CECR6
ENSP00000259726	1	CDSN
ENSP00000388386	1	CDSN
ENSP00000379242	1	CDRT1
ENSP00000394183	1	CDKN2AIPNL
ENSP00000364501	1	CD300LD
ENSP00000408012	1	CCHCR1
ENSP00000323782	1	CCDC43
ENSP00000373304	1	CCDC174
ENSP00000292779	1	CCDC116
ENSP00000366612	1	CBWD5
ENSP00000259199	1	CBWD2
ENSP00000308268	1	CARNS1
ENSP00000311984	1	CARKD
ENSP00000416290	1	C7orf25
ENSP00000347322	1	C6orf89
ENSP00000328069	1	C6orf58
ENSP00000359505	1	C6orf57

ENSP00000390018	1	C6orf10
ENSP00000410028	1	C6orf10
ENSP00000195455	1	C4orf6
ENSP00000294947	1	C2orf61
ENSP00000369853	1	C2CD2
ENSP00000356363	1	C1orf53
ENSP00000361603	1	C1orf50
ENSP00000420716	1	C1orf228
ENSP00000366537	1	C1orf200
ENSP00000355623	1	C1orf198
ENSP00000345972	1	C1orf168
ENSP00000360045	1	C1orf141
ENSP00000329920	1	C19orf59
ENSP00000329492	1	C18orf21
ENSP00000401362	1	C15orf41
ENSP00000401770	1	C14orf2
ENSP00000376767	1	C11orf57
ENSP00000359050	1	C10orf76
ENSP00000298298	1	C10orf25
ENSP00000260210	1	BUD13
ENSP00000364632	1	BPIFB4
ENSP00000364643	1	BPIFB3
ENSP00000331127	1	BOLA2
ENSP00000309644	1	BOD1
ENSP00000319979	1	B3GNTL1
ENSP00000319636	1	B3GNT4
ENSP00000372696	1	B3GALT4
ENSP00000390784	1	B3GALT4
ENSP00000394876	1	B3GALT4
ENSP00000398660	1	B3GALT4
ENSP00000356404	1	B3GALT2
ENSP00000363645	1	AWAT1
ENSP00000304500	1	ATP9B
ENSP00000406389	1	ATP6V1G2
ENSP00000413222	1	ATP6V1G2
ENSP00000218008	1	ATP1B4
ENSP00000353010	1	ATG2B
ENSP00000366475	1	ATG2A
ENSP00000345420	1	ASCL4
ENSP00000312458	1	ARV1
ENSP00000369346	1	ARSK
ENSP00000320219	1	ARSJ
ENSP00000352319	1	ARSF
ENSP00000315357	1	ARL6IP6
ENSP00000335578	1	ARGFX
ENSP00000368528	1	APOO

ENSP00000159087	1	ANO8
ENSP00000308772	1	ANKS4B
ENSP00000339802	1	ANKRD34B
ENSP00000386398	1	ANKRD30BL
ENSP00000378328	1	ANKRD13B
ENSP00000265742	1	ANKIB1
ENSP00000314914	1	AMER3
ENSP00000350469	1	AMER2
ENSP00000295453	1	ALPPL2
ENSP00000292566	1	ALKBH4
ENSP00000298375	1	AKR1E2
ENSP00000257787	1	AKIRIN2
ENSP00000350509	1	AIG1
ENSP00000247087	1	AHDC1
ENSP00000363207	1	AGAP5
ENSP00000393631	1	ADM5
ENSP00000310547	1	ADCK5
ENSP00000369238	1	ADAM32
ENSP00000320646	1	ACSF3
ENSP00000286353	1	ACPL2
ENSP00000367453	1	ACBD7
ENSP00000264990	1	ACAD11
ENSP00000273596	1	ABHD14A
ENSP00000273359	1	ABHD10
ENSP00000355880	1	2-Mar
ENSP00000031146	1	-
ENSP00000226094	1	-
ENSP00000226258	1	-
ENSP00000237694	1	-
ENSP00000246039	1	-
ENSP00000263301	1	-
ENSP00000273411	1	-
ENSP00000293201	1	-
ENSP00000293995	1	-
ENSP00000298215	1	-
ENSP00000298635	1	-
ENSP00000298901	1	-
ENSP00000299992	1	-
ENSP00000306472	1	-
ENSP00000311379	1	-
ENSP00000312767	1	-
ENSP00000313973	1	-
ENSP00000317447	1	-
ENSP00000317556	1	-
ENSP00000318340	1	-
ENSP00000324438	1	-

ENSP00000324450	1	-
ENSP00000328013	1	-
ENSP00000330342	1	-
ENSP00000331866	1	-
ENSP00000332124	1	-
ENSP00000332155	1	-
ENSP00000334201	1	-
ENSP00000334711	1	-
ENSP00000335650	1	-
ENSP00000339557	1	-
ENSP00000339737	1	-
ENSP00000343430	1	-
ENSP00000344077	1	-
ENSP00000344426	1	-
ENSP00000345431	1	-
ENSP00000347310	1	-
ENSP00000348580	1	-
ENSP00000350204	1	-
ENSP00000352682	1	-
ENSP00000353324	1	-
ENSP00000353928	1	-
ENSP00000354703	1	-
ENSP00000354769	1	-
ENSP00000356177	1	-
ENSP00000358670	1	-
ENSP00000359239	1	-
ENSP00000360177	1	-
ENSP00000360235	1	-
ENSP00000362594	1	-
ENSP00000363573	1	-
ENSP00000364722	1	-
ENSP00000365415	1	-
ENSP00000366948	1	-
ENSP00000367166	1	-
ENSP00000368353	1	-
ENSP00000369560	1	-
ENSP00000371322	1	-
ENSP00000372914	1	-
ENSP00000374979	1	-
ENSP00000375503	1	-
ENSP00000375558	1	-
ENSP00000380784	1	-
ENSP00000380937	1	-
ENSP00000381019	1	-
ENSP00000381059	1	-
ENSP00000381121	1	-

ENSP00000381143	1	-
ENSP00000381900	1	-
ENSP00000381903	1	-
ENSP00000382149	1	-
ENSP00000382296	1	-
ENSP00000382346	1	-
ENSP00000382780	1	-
ENSP00000383017	1	-
ENSP00000383870	1	-
ENSP00000384640	1	-
ENSP00000384718	1	-
ENSP00000385074	1	-
ENSP00000387466	1	-
ENSP00000387558	1	-
ENSP00000388672	1	-
ENSP00000388916	1	-
ENSP00000388987	1	-
ENSP00000389156	1	-
ENSP00000390147	1	-
ENSP00000390520	1	-
ENSP00000391533	1	-
ENSP00000392010	1	-
ENSP00000392249	1	-
ENSP00000392940	1	-
ENSP00000392982	1	-
ENSP00000393556	1	-
ENSP00000393936	1	-
ENSP00000394523	1	-
ENSP00000395270	1	-
ENSP00000395735	1	-
ENSP00000395763	1	-
ENSP00000395798	1	-
ENSP00000395900	1	-
ENSP00000396627	1	-
ENSP00000396960	1	-
ENSP00000397439	1	-
ENSP00000397549	1	-
ENSP00000398461	1	-
ENSP00000398762	1	-
ENSP00000399699	1	-
ENSP00000401268	1	-
ENSP00000401279	1	-
ENSP00000401628	1	-
ENSP00000401786	1	-
ENSP00000402497	1	-
ENSP00000403037	1	-

ENSP00000403780	1	-
ENSP00000403931	1	-
ENSP00000404034	1	-
ENSP00000404254	1	-
ENSP00000404946	1	-
ENSP00000405654	1	-
ENSP00000406271	1	-
ENSP00000406362	1	-
ENSP00000406378	1	-
ENSP00000407415	1	-
ENSP00000407488	1	-
ENSP00000407532	1	-
ENSP00000408050	1	-
ENSP00000408337	1	-
ENSP00000408398	1	-
ENSP00000410122	1	-
ENSP00000410420	1	-
ENSP00000410573	1	-
ENSP00000410906	1	-
ENSP00000410949	1	-
ENSP00000411236	1	-
ENSP00000411513	1	-
ENSP00000411605	1	-
ENSP00000412251	1	-
ENSP00000413564	1	-
ENSP00000413650	1	-
ENSP00000413706	1	-
ENSP00000415110	1	-
ENSP00000415596	1	-
ENSP00000416141	1	-
ENSP00000416297	1	-
ENSP00000416821	1	-
ENSP00000416909	1	-
ENSP00000418124	1	-
ENSP00000295131	0	ZSWIM2
ENSP00000303015	0	ZRSR2
ENSP00000375133	0	ZRSR1
ENSP00000313822	0	ZNF821
ENSP00000409463	0	ZNF740
ENSP00000291770	0	ZNF714
ENSP00000347043	0	ZNF649
ENSP00000375598	0	ZNF628
ENSP00000343617	0	ZNF606
ENSP00000344791	0	ZNF600
ENSP00000301475	0	ZNF558
ENSP00000252840	0	ZNF557

ENSP00000375582	0	ZNF552
ENSP00000339823	0	ZNF546
ENSP00000353652	0	ZNF528
ENSP00000219478	0	ZNF500
ENSP00000402815	0	ZNF497
ENSP00000364645	0	ZNF484
ENSP00000270617	0	ZNF473
ENSP00000309161	0	ZNF471
ENSP00000353491	0	ZNF460
ENSP00000305373	0	ZNF440
ENSP00000305077	0	ZNF439
ENSP00000253115	0	ZNF426
ENSP00000344308	0	ZNF341
ENSP00000252979	0	ZNF337
ENSP00000353586	0	ZNF280B
ENSP00000336719	0	ZNF226
ENSP00000380315	0	ZNF208
ENSP00000379464	0	ZNF134
ENSP00000319716	0	ZNF101
ENSP00000351042	0	ZNF100
ENSP00000391742	0	ZMYND15
ENSP00000361646	0	ZMYND12
ENSP00000362435	0	ZMYM6NB
ENSP00000352920	0	ZMYM1
ENSP00000262961	0	ZFR2
ENSP00000309429	0	ZDHHC24
ENSP00000303468	0	ZCCHC4
ENSP00000268616	0	ZCCHC14
ENSP00000275766	0	ZC3HAV1L
ENSP00000282007	0	ZC3H13
ENSP00000413557	0	ZBTB9
ENSP00000300101	0	ZBTB39
ENSP00000356674	0	ZBTB37
ENSP00000362551	0	ZBTB34
ENSP00000414777	0	ZBTB12
ENSP00000376519	0	ZBBX
ENSP00000343435	0	YIF1B
ENSP00000327570	0	XKRX
ENSP00000331704	0	XKR3
ENSP00000303061	0	XAGE3
ENSP00000267522	0	WDR89
ENSP00000360065	0	WDR78
ENSP00000288912	0	WDR66
ENSP00000361570	0	WDR65
ENSP00000006526	0	WDR54
ENSP00000399454	0	WDR46

ENSP00000405614	0	WDR46
ENSP00000293883	0	WDR24
ENSP00000316775	0	WBSCR28
ENSP00000297873	0	WBSCR27
ENSP00000411804	0	VWA7
ENSP00000374049	0	VWA3A
ENSP00000357080	0	VSIG8
ENSP00000256362	0	VRTN
ENSP00000374037	0	VPS9D1
ENSP00000397879	0	VPS8
ENSP00000281187	0	VPS26B
ENSP00000394802	0	VARS2
ENSP00000211402	0	VARS
ENSP00000364815	0	VARS
ENSP00000390673	0	VARS
ENSP00000392396	0	VARS
ENSP00000403359	0	VARS
ENSP00000311245	0	USMG5
ENSP00000230256	0	UNC93A
ENSP00000344942	0	UNC119B
ENSP00000382713	0	UBE2QL1
ENSP00000383911	0	UBAC2
ENSP00000406144	0	TTC39A
ENSP00000370419	0	TTC38
ENSP00000383873	0	TTC34
ENSP00000233623	0	TTC31
ENSP00000381003	0	TTC28
ENSP00000357220	0	TTC24
ENSP00000384479	0	TTC18
ENSP00000355621	0	TTC13
ENSP00000342499	0	TSTD2
ENSP00000373485	0	TSNAXIP1
ENSP00000357234	0	TSACC
ENSP00000361982	0	TRUB2
ENSP00000297994	0	TRMT10B
ENSP00000317498	0	TRIML2
ENSP00000269383	0	TRIM65
ENSP00000403221	0	TRIM15
ENSP00000413961	0	TRIM15
ENSP00000374867	0	TRGV5
ENSP00000374868	0	TRGV4
ENSP00000404928	0	TRGV2
ENSP00000374864	0	TRGV10
ENSP00000417149	0	TRGJ2
ENSP00000342570	0	TREML4
ENSP00000374884	0	TRBV7-3

ENSP00000413966	0	TRBV5-4
ENSP00000374904	0	TRBV5-1
ENSP00000374910	0	TRBV3-1
ENSP00000397118	0	TRBV30
ENSP00000374922	0	TRBV27
ENSP00000374920	0	TRBV24-1
ENSP00000374919	0	TRBV23-1
ENSP00000374887	0	TRBV10-1
ENSP00000417114	0	TRBJ2-6
ENSP00000420233	0	TRBJ2-5
ENSP00000400448	0	TPRXL
ENSP00000246024	0	TMX4
ENSP00000364938	0	TMEM82
ENSP00000380646	0	TMEM80
ENSP00000361095	0	TMEM69
ENSP00000414786	0	TMEM64
ENSP00000262817	0	TMEM59L
ENSP00000326063	0	TMEM39A
ENSP00000257262	0	TMEM258
ENSP00000309792	0	TMEM234
ENSP00000403130	0	TMEM233
ENSP00000394680	0	TMEM232
ENSP00000350050	0	TMEM229B
ENSP00000375053	0	TMEM203
ENSP00000296978	0	TMEM200A
ENSP00000292114	0	TMEM199
ENSP00000296582	0	TMEM184C
ENSP00000342148	0	TMEM167B
ENSP00000290079	0	TMEM141
ENSP00000303999	0	TMEM133
ENSP00000372394	0	TMEM129
ENSP00000227525	0	TMEM109
ENSP00000334849	0	TMEM104
ENSP00000216468	0	TMED8
ENSP00000381104	0	TM9SF4
ENSP00000374014	0	TM6SF2
ENSP00000317000	0	TM6SF1
ENSP00000303028	0	TM4SF20
ENSP00000254616	0	TIMM10B
ENSP00000355162	0	TIGD4
ENSP00000357807	0	THEM5
ENSP00000342169	0	TEX9
ENSP00000323795	0	TEX35
ENSP00000315111	0	TDRP
ENSP00000341539	0	TCAIM
ENSP00000383522	0	TBC1D3H

ENSP00000309794	0	TBC1D16
ENSP00000240691	0	TAS2R9
ENSP00000240615	0	TAS2R8
ENSP00000327724	0	TAS2R60
ENSP00000247883	0	TAS2R5
ENSP00000334050	0	TAS2R42
ENSP00000386201	0	TAS2R41
ENSP00000386210	0	TAS2R40
ENSP00000247881	0	TAS2R4
ENSP00000405095	0	TAS2R39
ENSP00000247879	0	TAS2R3
ENSP00000375091	0	TAS2R19
ENSP00000375095	0	TAS2R13
ENSP00000385347	0	TAPT1
ENSP00000372684	0	TAPBP
ENSP00000364034	0	TAP2
ENSP00000361519	0	SZT2
ENSP00000385122	0	SYCE3
ENSP00000367911	0	SYCE1L
ENSP00000405482	0	SVOPL
ENSP00000333310	0	SULT1C3
ENSP00000324551	0	SSUH2
ENSP00000414512	0	SRRM5
ENSP00000215917	0	SRRD
ENSP00000279058	0	SPINT4
ENSP00000329522	0	SPDYE4
ENSP00000291672	0	SPATC1L
ENSP00000280191	0	SPATA4
ENSP00000268981	0	SPATA22
ENSP00000335392	0	SPATA12
ENSP00000405210	0	SPANXN4
ENSP00000405202	0	SPANXB2
ENSP00000306940	0	SNX29
ENSP00000226460	0	SMR3A
ENSP00000229570	0	SMIM8
ENSP00000417147	0	SMIM7
ENSP00000382231	0	SMIM11
ENSP00000338988	0	SLMO1
ENSP00000312402	0	SLFN11
ENSP00000263997	0	SLC7A6OS
ENSP00000297524	0	SLC7A13
ENSP00000236495	0	SLC5A9
ENSP00000389244	0	SLC44A4
ENSP00000326070	0	SLC41A3
ENSP00000363807	0	SLC39A7
ENSP00000410656	0	SLC39A7

ENSP00000414145	0	SLC39A7
ENSP00000416439	0	SLC39A7
ENSP00000366586	0	SLC39A12
ENSP00000299709	0	SLC38A8
ENSP00000355384	0	SLC26A11
ENSP00000381782	0	SLC25A45
ENSP00000322649	0	SLC25A41
ENSP00000294454	0	SLC25A34
ENSP00000301454	0	SLC25A23
ENSP00000307443	0	SLC22A25
ENSP00000396586	0	SLC22A24
ENSP00000385028	0	SLC22A23
ENSP00000275227	0	SLC18B1
ENSP00000330141	0	SLC16A5
ENSP00000358794	0	SLC16A4
ENSP00000295190	0	SLC16A14
ENSP00000340402	0	SLC15A5
ENSP00000293800	0	SLC13A5
ENSP00000357072	0	SLAMF9
ENSP00000264313	0	SLAIN2
ENSP00000267219	0	SLAIN1
ENSP00000372827	0	SKIV2L
ENSP00000399530	0	SKIV2L
ENSP00000400626	0	SKIV2L
ENSP00000305529	0	SIRPG
ENSP00000222345	0	SIPA1L3
ENSP00000374125	0	SIGLEC15
ENSP00000358853	0	SH3BGRL2
ENSP00000373031	0	SFTA2
ENSP00000285947	0	SETD9
ENSP00000329189	0	SETD4
ENSP00000331376	0	SERHL2
ENSP00000284951	0	SEL1L2
ENSP00000261847	0	SECISBP2L
ENSP00000368165	0	SDHAF1
ENSP00000344545	0	SCGB1C1
ENSP00000384182	0	SCFD2
ENSP00000297354	0	SBSPON
ENSP00000229903	0	SAYSD1
ENSP00000359707	0	SAMD13
ENSP00000357199	0	RRNAD1
ENSP00000373331	0	RPUSD3
ENSP00000346046	0	RPS17L
ENSP00000389182	0	RPP30
ENSP00000297613	0	RPP25L
ENSP00000346080	0	RPL22L1

ENSP00000298283	0	RPL10L
ENSP00000251776	0	ROPN1B
ENSP00000257575	0	RNFT2
ENSP00000274811	0	RNF44
ENSP00000398512	0	RNF39
ENSP00000300650	0	RNF214
ENSP00000375397	0	RNF208
ENSP00000385440	0	RNASEK
ENSP00000269051	0	RHBDL3
ENSP00000397644	0	RFX7
ENSP00000170168	0	REXO1
ENSP00000233596	0	REEP6
ENSP00000291892	0	RDH13
ENSP00000281722	0	RBM46
ENSP00000361557	0	RBM41
ENSP00000375562	0	RBM12B-AS1
ENSP00000383421	0	RBM11
ENSP00000286380	0	RAET1L
ENSP00000265806	0	R3HCC1
ENSP00000348645	0	PXDNL
ENSP00000369636	0	PXDC1
ENSP00000293922	0	PTX4
ENSP00000330389	0	PTRHD1
ENSP00000292478	0	PTCD1
ENSP00000280606	0	PRSS53
ENSP00000293851	0	PRSS33
ENSP00000359378	0	PRRG3
ENSP00000386166	0	PRR21
ENSP00000394510	0	PRR12
ENSP00000332034	0	PROSER1
ENSP00000416033	0	PROB1
ENSP00000313816	0	PRIMPOL
ENSP00000335675	0	PRELID2
ENSP00000263765	0	PRDM11
ENSP00000350358	0	PRAMEF12
ENSP00000332134	0	PRAMEF1
ENSP00000364295	0	PQLC2
ENSP00000406219	0	PPT2
ENSP00000315035	0	PPP1R42
ENSP00000292330	0	PPP1R35
ENSP00000403557	0	PPP1R11
ENSP00000414808	0	PPP1R11
ENSP00000377385	0	PPAN-P2RY11
ENSP00000344125	0	POMGNT2
ENSP00000254320	0	PODNL1
ENSP00000358232	0	PNLIPRP3

ENSP00000337757	0	PIH1D3
ENSP00000339382	0	PIGO
ENSP00000309515	0	PHYHD1
ENSP00000322530	0	PGBD5
ENSP00000392303	0	PET100
ENSP00000347931	0	PCNXL3
ENSP00000300146	0	PATL1
ENSP00000364089	0	PAGE3
ENSP00000364110	0	PAGE2B
ENSP00000364107	0	PAGE2
ENSP00000364261	0	OTUD3
ENSP00000308714	0	OR9Q2
ENSP00000334934	0	OR9Q1
ENSP00000307598	0	OR9K2
ENSP00000302606	0	OR9I1
ENSP00000307515	0	OR9G4
ENSP00000309012	0	OR9G1
ENSP00000316518	0	OR9A2
ENSP00000304188	0	OR8U1
ENSP00000310632	0	OR8S1
ENSP00000323853	0	OR8K5
ENSP00000323555	0	OR8K3
ENSP00000279783	0	OR8K1
ENSP00000301529	0	OR8J3
ENSP00000304060	0	OR8J1
ENSP00000303864	0	OR8I2
ENSP00000323595	0	OR8H1
ENSP00000325381	0	OR8D4
ENSP00000350022	0	OR8D2
ENSP00000350474	0	OR8D1
ENSP00000330280	0	OR8B8
ENSP00000348449	0	OR8B4
ENSP00000307159	0	OR8B12
ENSP00000284287	0	OR8A1
ENSP00000302867	0	OR7G3
ENSP00000303822	0	OR7G2
ENSP00000293614	0	OR7G1
ENSP00000387523	0	OR7E24
ENSP00000310488	0	OR7D4
ENSP00000345563	0	OR7D2
ENSP00000248072	0	OR7C2
ENSP00000248073	0	OR7C1
ENSP00000316955	0	OR7A5
ENSP00000328144	0	OR7A17
ENSP00000248058	0	OR7A10
ENSP00000304807	0	OR6Y1

ENSP00000333724	0	OR6X1
ENSP00000396085	0	OR6V1
ENSP00000325203	0	OR6T1
ENSP00000313110	0	OR6S1
ENSP00000307734	0	OR6Q1
ENSP00000334721	0	OR6P1
ENSP00000344101	0	OR6N2
ENSP00000335535	0	OR6N1
ENSP00000311038	0	OR6M1
ENSP00000357126	0	OR6K6
ENSP00000357128	0	OR6K3
ENSP00000352626	0	OR6K2
ENSP00000305640	0	OR6F1
ENSP00000328402	0	OR6C76
ENSP00000368987	0	OR6C75
ENSP00000342836	0	OR6C74
ENSP00000329153	0	OR6C70
ENSP00000368983	0	OR6C68
ENSP00000368986	0	OR6C65
ENSP00000351211	0	OR6C6
ENSP00000377799	0	OR6C4
ENSP00000368990	0	OR6C1
ENSP00000386151	0	OR6B1
ENSP00000330384	0	OR6A2
ENSP00000342448	0	OR5W2
ENSP00000305403	0	OR5T3
ENSP00000323688	0	OR5T2
ENSP00000323612	0	OR5T1
ENSP00000308595	0	OR5R1
ENSP00000332068	0	OR5P3
ENSP00000331823	0	OR5P2
ENSP00000279791	0	OR5M9
ENSP00000323354	0	OR5M8
ENSP00000347003	0	OR5K4
ENSP00000373194	0	OR5K3
ENSP00000310788	0	OR5J2
ENSP00000301532	0	OR5I1
ENSP00000347418	0	OR5H2
ENSP00000401706	0	OR5H14
ENSP00000278409	0	OR5F1
ENSP00000335025	0	OR5D18
ENSP00000367649	0	OR5D16
ENSP00000334456	0	OR5D14
ENSP00000354800	0	OR5D13
ENSP00000362784	0	OR5C1
ENSP00000308270	0	OR5B3

ENSP00000353537	0	OR5B21
ENSP00000303076	0	OR5B2
ENSP00000349945	0	OR5B17
ENSP00000306657	0	OR5B12
ENSP00000302057	0	OR5AU1
ENSP00000324111	0	OR5AS1
ENSP00000302639	0	OR5AR1
ENSP00000303111	0	OR5AP2
ENSP00000320302	0	OR5AN1
ENSP00000322784	0	OR5AK2
ENSP00000303834	0	OR5A2
ENSP00000303096	0	OR5A1
ENSP00000321196	0	OR56B4
ENSP00000322939	0	OR56B1
ENSP00000328215	0	OR56A4
ENSP00000331572	0	OR56A3
ENSP00000321246	0	OR56A1
ENSP00000309673	0	OR52W1
ENSP00000369742	0	OR52R1
ENSP00000322866	0	OR52N5
ENSP00000323224	0	OR52N4
ENSP00000322801	0	OR52N2
ENSP00000322823	0	OR52N1
ENSP00000353343	0	OR52M1
ENSP00000369728	0	OR52J3
ENSP00000326259	0	OR52H1
ENSP00000328878	0	OR52E6
ENSP00000321426	0	OR52E4
ENSP00000322088	0	OR52E2
ENSP00000326232	0	OR52D1
ENSP00000341581	0	OR52B6
ENSP00000303469	0	OR52A5
ENSP00000321729	0	OR51V1
ENSP00000369738	0	OR51T1
ENSP00000322754	0	OR51S1
ENSP00000300778	0	OR51Q1
ENSP00000333196	0	OR51M1
ENSP00000322156	0	OR51L1
ENSP00000332473	0	OR51J1
ENSP00000341987	0	OR51I2
ENSP00000369559	0	OR51I1
ENSP00000322724	0	OR51H1P
ENSP00000322593	0	OR51G2
ENSP00000322546	0	OR51G1
ENSP00000323952	0	OR51F2
ENSP00000345163	0	OR51F1

ENSP00000380155	0	OR51E1
ENSP00000350222	0	OR51D1
ENSP00000369568	0	OR51B6
ENSP00000300773	0	OR51B5
ENSP00000369573	0	OR51B4
ENSP00000327540	0	OR51B2
ENSP00000352305	0	OR51A7
ENSP00000307751	0	OR4X2
ENSP00000321506	0	OR4X1
ENSP00000310337	0	OR4S2
ENSP00000321447	0	OR4S1
ENSP00000324831	0	OR4P4
ENSP00000332110	0	OR4N5
ENSP00000332500	0	OR4N4
ENSP00000319601	0	OR4N2
ENSP00000319217	0	OR4L1
ENSP00000319511	0	OR4K5
ENSP00000298642	0	OR4K2
ENSP00000319197	0	OR4K17
ENSP00000304077	0	OR4K15
ENSP00000305011	0	OR4K14
ENSP00000319322	0	OR4K13
ENSP00000285600	0	OR4K1
ENSP00000327525	0	OR4F6
ENSP00000333184	0	OR4F15
ENSP00000386195	0	OR4E2
ENSP00000328563	0	OR4D9
ENSP00000300127	0	OR4D6
ENSP00000305970	0	OR4D5
ENSP00000320077	0	OR4D11
ENSP00000324769	0	OR4C6
ENSP00000321338	0	OR4C5
ENSP00000329056	0	OR4C46
ENSP00000321419	0	OR4C3
ENSP00000324913	0	OR4C16
ENSP00000324958	0	OR4C15
ENSP00000306651	0	OR4C11
ENSP00000311605	0	OR4B1
ENSP00000367664	0	OR4A5
ENSP00000325128	0	OR4A16
ENSP00000316284	0	OR2Z1
ENSP00000312403	0	OR2Y1
ENSP00000353516	0	OR2W3
ENSP00000332185	0	OR2V2
ENSP00000404102	0	OR2V1
ENSP00000347965	0	OR2T6

ENSP00000342008	0	OR2T27
ENSP00000328934	0	OR2T11
ENSP00000329210	0	OR2T10
ENSP00000355430	0	OR2T1
ENSP00000344040	0	OR2S2
ENSP00000306688	0	OR2M4
ENSP00000350836	0	OR2L13
ENSP00000305055	0	OR2K2
ENSP00000409339	0	OR2H2
ENSP00000341291	0	OR2G6
ENSP00000326301	0	OR2G3
ENSP00000326349	0	OR2G2
ENSP00000320560	0	OR2D3
ENSP00000299459	0	OR2D2
ENSP00000355443	0	OR2C3
ENSP00000307726	0	OR2C1
ENSP00000244623	0	OR2B6
ENSP00000304419	0	OR2B2
ENSP00000325682	0	OR2B11
ENSP00000304846	0	OR2AT4
ENSP00000323423	0	OR2AP1
ENSP00000355436	0	OR2AK2
ENSP00000325078	0	OR2AJ1
ENSP00000342697	0	OR2AG2
ENSP00000313936	0	OR2AE1
ENSP00000386208	0	OR2A5
ENSP00000386167	0	OR2A25
ENSP00000386209	0	OR2A2
ENSP00000386137	0	OR2A14
ENSP00000386174	0	OR2A12
ENSP00000297913	0	OR1Q1
ENSP00000362792	0	OR1N2
ENSP00000306974	0	OR1N1
ENSP00000401966	0	OR1M1
ENSP00000306607	0	OR1L8
ENSP00000302863	0	OR1L3
ENSP00000362790	0	OR1L1
ENSP00000277309	0	OR1K1
ENSP00000343521	0	OR1J4
ENSP00000259357	0	OR1J1
ENSP00000209540	0	OR1I1
ENSP00000331545	0	OR1G1
ENSP00000305424	0	OR1F1
ENSP00000386138	0	OR1C1
ENSP00000303151	0	OR1B1
ENSP00000371377	0	OR1A2

ENSP00000305207	0	OR1A1
ENSP00000283225	0	OR14K1
ENSP00000339726	0	OR14I1
ENSP00000355441	0	OR14A2
ENSP00000350248	0	OR14A16
ENSP00000349930	0	OR11G2
ENSP00000383550	0	OR11A1
ENSP00000378516	0	OR10W1
ENSP00000302199	0	OR10V1
ENSP00000334115	0	OR10T2
ENSP00000357134	0	OR10R2
ENSP00000314324	0	OR10Q1
ENSP00000308082	0	OR10P1
ENSP00000324251	0	OR10K2
ENSP00000289451	0	OR10K1
ENSP00000334441	0	OR10J5
ENSP00000331789	0	OR10J3
ENSP00000399078	0	OR10J1
ENSP00000318834	0	OR10H4
ENSP00000307130	0	OR10H3
ENSP00000306095	0	OR10H2
ENSP00000325076	0	OR10G4
ENSP00000302437	0	OR10G3
ENSP00000308689	0	OR10AD1
ENSP00000326718	0	OR10A7
ENSP00000312470	0	OR10A6
ENSP00000353988	0	OR10A3
ENSP00000278855	0	OOSP2
ENSP00000330075	0	OGFOD3
ENSP00000359598	0	ODF2L
ENSP00000361544	0	NUP62CL
ENSP00000357547	0	NUP210L
ENSP00000330247	0	NT5DC4
ENSP00000326858	0	NT5DC1
ENSP00000269534	0	NT5C3B
ENSP00000419740	0	NSUN4
ENSP00000318986	0	NSUN3
ENSP00000389894	0	NRM
ENSP00000407779	0	NRM
ENSP00000408118	0	NRM
ENSP00000346335	0	NRDE2
ENSP00000399107	0	NPIPB3
ENSP00000343891	0	NLRP13
ENSP00000359340	0	NKAIN4
ENSP00000363520	0	NIPAL3
ENSP00000358307	0	NHLRC2

ENSP00000396656	0	NFKBIL1
ENSP00000341637	0	NCR3LG1
ENSP00000342156	0	NCR3
ENSP00000316782	0	NBPF3
ENSP00000382328	0	N4BP2L2
ENSP00000369473	0	N4BP2L1
ENSP00000240050	0	MTERFD3
ENSP00000300182	0	MS4A6E
ENSP00000415222	0	MS4A4E
ENSP00000272418	0	MRPS5
ENSP00000256441	0	MRPS36
ENSP00000259873	0	MRPS18B
ENSP00000398494	0	MRPS18B
ENSP00000415703	0	MRPS18B
ENSP00000361206	0	MRPS18A
ENSP00000053468	0	MRPS10
ENSP00000338389	0	MRPL35
ENSP00000315017	0	MRPL1
ENSP00000381857	0	MROH6
ENSP00000382476	0	MROH2B
ENSP00000314042	0	MRGPRX4
ENSP00000330612	0	MRGPRG
ENSP00000257776	0	MRAP2
ENSP00000311200	0	MPPE1
ENSP00000362869	0	MORN5
ENSP00000367792	0	MORN1
ENSP00000264819	0	MIER2
ENSP00000252229	0	MICB
ENSP00000373006	0	MICB
ENSP00000393355	0	MICB
ENSP00000402484	0	MICB
ENSP00000414846	0	MICAL3
ENSP00000402134	0	MICA
ENSP00000366939	0	MGME1
ENSP00000301327	0	MFSD3
ENSP00000385527	0	MFSD2B
ENSP00000337240	0	MFSD11
ENSP00000373300	0	METTL6
ENSP00000320349	0	METTL4
ENSP00000341543	0	METTL23
ENSP00000267273	0	METTL21C
ENSP00000300209	0	METTL21B
ENSP00000385481	0	METTL21A
ENSP00000369849	0	MEDAG
ENSP00000363533	0	MDH1B
ENSP00000396484	0	MDC1

ENSP00000416511	0	MDC1
ENSP00000362144	0	MCU
ENSP00000244217	0	MCEE
ENSP00000381150	0	MBLAC1
ENSP00000370800	0	MBD3L2
ENSP00000314560	0	MAP6D1
ENSP00000319388	0	MAMDC4
ENSP00000315064	0	MAGEF1
ENSP00000298296	0	MAGEC3
ENSP00000244096	0	MAGEA10
ENSP00000267838	0	LYSMD2
ENSP00000368396	0	LYRM9
ENSP00000389102	0	LY6G6F
ENSP00000409428	0	LY6G6C
ENSP00000397467	0	LY6G5C
ENSP00000400837	0	LY6G5C
ENSP00000407899	0	LY6G5B
ENSP00000372991	0	LTA
ENSP00000407133	0	LTA
ENSP00000323304	0	LSMEM1
ENSP00000372883	0	LSM2
ENSP00000403345	0	LSM2
ENSP00000289488	0	LRTOMT
ENSP00000338887	0	LRRC8D
ENSP00000332674	0	LRRC8B
ENSP00000325978	0	LRRC31
ENSP00000369395	0	LRRC19
ENSP00000367315	0	LRRC10B
ENSP00000296603	0	LMBRD2
ENSP00000267102	0	LMBR1L
ENSP00000274382	0	LIX1
ENSP00000360411	0	LDLRAD1
ENSP00000360696	0	LCN12
ENSP00000418491	0	LCN10
ENSP00000343118	0	LAMTOR4
ENSP00000246070	0	LAMP5
ENSP00000366950	0	KRTAP9-2
ENSP00000375148	0	KRTAP29-1
ENSP00000383219	0	KRTAP10-6
ENSP00000334798	0	KRT26
ENSP00000310573	0	KRT25
ENSP00000345797	0	KRBOX4
ENSP00000362206	0	KLHL4
ENSP00000379434	0	KLHL28
ENSP00000272797	0	KLHL23
ENSP00000379034	0	KLHDC7B

ENSP00000363647	0	KIFC1
ENSP00000393963	0	KIFC1
ENSP00000320794	0	KIAA2018
ENSP00000376213	0	KIAA1683
ENSP00000309501	0	KIAA1239
ENSP00000386787	0	KIAA0922
ENSP00000242109	0	KIAA0087
ENSP00000398279	0	KDM2B
ENSP00000275532	0	KCTD7
ENSP00000347188	0	KCTD6
ENSP00000368402	0	KCTD4
ENSP00000287042	0	KCNS2
ENSP00000312129	0	KCNG4
ENSP00000256544	0	KATNBL1
ENSP00000396749	0	KANSL3
ENSP00000328923	0	KANK3
ENSP00000293405	0	IZUMO2
ENSP00000355121	0	ITPRIPL1
ENSP00000376973	0	ISY1
ENSP00000291358	0	IQCC
ENSP00000251296	0	IGSF21
ENSP00000374818	0	IGLV8-61
ENSP00000374830	0	IGLV7-46
ENSP00000374833	0	IGLV7-43
ENSP00000374824	0	IGLV5-52
ENSP00000374831	0	IGLV5-45
ENSP00000374835	0	IGLV5-37
ENSP00000374817	0	IGLV4-69
ENSP00000374819	0	IGLV4-60
ENSP00000374853	0	IGLV4-3
ENSP00000374843	0	IGLV3-21
ENSP00000374848	0	IGLV3-12
ENSP00000374850	0	IGLV3-10
ENSP00000374837	0	IGLV2-33
ENSP00000374845	0	IGLV2-18
ENSP00000374849	0	IGLV2-11
ENSP00000374836	0	IGLV1-36
ENSP00000374821	0	IGLV11-55
ENSP00000374822	0	IGLV10-54
ENSP00000419233	0	IGLJ7
ENSP00000419267	0	IGLJ6
ENSP00000418818	0	IGLJ5
ENSP00000418274	0	IGLJ2
ENSP00000374857	0	IGLC3
ENSP00000374791	0	IGKV6-21
ENSP00000374782	0	IGKV3-7

ENSP00000418138	0	IGKV2-30
ENSP00000418292	0	IGKV1D-17
ENSP00000417097	0	IGKJ4
ENSP00000418768	0	IGKJ2
ENSP00000375002	0	IGHV6-1
ENSP00000410711	0	IGHV4-59
ENSP00000394447	0	IGHV3-74
ENSP00000375045	0	IGHV3-73
ENSP00000375007	0	IGHV3-7
ENSP00000375012	0	IGHV3-15
ENSP00000375010	0	IGHV3-11
ENSP00000375008	0	IGHV1-8
ENSP00000375031	0	IGHV1-46
ENSP00000375014	0	IGHV1-18
ENSP00000418164	0	IGHJ5
ENSP00000419074	0	IGHJ4
ENSP00000417751	0	IGHD6-25
ENSP00000419773	0	IGHD3-10
ENSP00000366926	0	IGFL4
ENSP00000344860	0	IGFL3
ENSP00000369558	0	IFNA6
ENSP00000369564	0	IFNA16
ENSP00000372059	0	IFITM5
ENSP00000351047	0	ICA1L
ENSP00000333300	0	HSD17B13
ENSP00000390354	0	HS3ST6
ENSP00000346283	0	HRCT1
ENSP00000350049	0	HOMEZ
ENSP00000383506	0	HLA-G
ENSP00000409132	0	HLA-G
ENSP00000414905	0	HLA-F
ENSP00000409910	0	HLA-E
ENSP00000405295	0	HLA-DRA
ENSP00000387578	0	HLA-DQB2
ENSP00000406872	0	HLA-DQB2
ENSP00000372734	0	HLA-DQB1
ENSP00000241802	0	HLA-DQA2
ENSP00000364076	0	HLA-DQA2
ENSP00000372738	0	HLA-DQA1
ENSP00000387892	0	HLA-DQA1
ENSP00000399298	0	HLA-DPB1
ENSP00000408146	0	HLA-DPB1
ENSP00000363941	0	HLA-DPA1
ENSP00000393566	0	HLA-DPA1
ENSP00000397587	0	HLA-DPA1
ENSP00000390020	0	HLA-DOB

ENSP00000229829	0	HLA-DOA
ENSP00000398890	0	HLA-DMB
ENSP00000411321	0	HLA-DMB
ENSP00000373114	0	HLA-A
ENSP00000388724	0	HLA-A
ENSP00000398188	0	HLA-A
ENSP00000352442	0	HIST1H2BM
ENSP00000350402	0	HHLA2
ENSP00000310621	0	HHATL
ENSP00000377980	0	HEPACAM2
ENSP00000233099	0	HEATR5B
ENSP00000335447	0	HEATR4
ENSP00000253669	0	HAUS8
ENSP00000386076	0	GUCD1
ENSP00000308080	0	GTF2IRD2B
ENSP00000412060	0	GREB1L
ENSP00000319673	0	GPR89A
ENSP00000331600	0	GPR173
ENSP00000308733	0	GPR151
ENSP00000334540	0	GPR141
ENSP00000321698	0	GPR137
ENSP00000298110	0	GPR101
ENSP00000368305	0	GPCPD1
ENSP00000290795	0	GPBP1L1
ENSP00000355902	0	GPATCH2
ENSP00000365057	0	GPANK1
ENSP00000382999	0	GPANK1
ENSP00000406895	0	GPANK1
ENSP00000303077	0	GOT1L1
ENSP00000267731	0	GOLGA8B
ENSP00000310770	0	GLIPR1L1
ENSP00000268797	0	GFOD2
ENSP00000320815	0	GDPD4
ENSP00000384363	0	GDPD3
ENSP00000355444	0	GCSAML
ENSP00000243913	0	GCNT7
ENSP00000229384	0	GATC
ENSP00000384742	0	GAGE2E
ENSP00000355421	0	GAGE2A
ENSP00000409832	0	GAGE12J
ENSP00000282538	0	GADL1
ENSP00000357540	0	FUOM
ENSP00000265342	0	FSTL4
ENSP00000420405	0	FSBP
ENSP00000278882	0	FRG1B
ENSP00000255759	0	FOPNL

ENSP00000399235	0	FOLR3
ENSP00000359034	0	FNDC7
ENSP00000361413	0	FIBCD1
ENSP00000274457	0	FEM1C
ENSP00000377311	0	FDX1L
ENSP00000334430	0	FAM9C
ENSP00000318716	0	FAM9B
ENSP00000370391	0	FAM9A
ENSP00000300030	0	FAM96A
ENSP00000391671	0	FAM92A1
ENSP00000407067	0	FAM86B1
ENSP00000398502	0	FAM86A
ENSP00000295092	0	FAM84A
ENSP00000323034	0	FAM83A
ENSP00000351631	0	FAM76B
ENSP00000351138	0	FAM73B
ENSP00000359827	0	FAM73A
ENSP00000358174	0	FAM72C
ENSP00000326652	0	FAM71F1
ENSP00000398617	0	FAM71E2
ENSP00000315247	0	FAM71C
ENSP00000305596	0	FAM71B
ENSP00000360757	0	FAM69B
ENSP00000359333	0	FAM69A
ENSP00000308575	0	FAM46D
ENSP00000358458	0	FAM46C
ENSP00000366747	0	FAM27E1
ENSP00000299338	0	FAM227B
ENSP00000282633	0	FAM21A
ENSP00000367682	0	FAM213B
ENSP00000323635	0	FAM210A
ENSP00000263733	0	FAM20B
ENSP00000334415	0	FAM183A
ENSP00000280987	0	FAM177A1
ENSP00000307954	0	FAM174A
ENSP00000280330	0	FAM173B
ENSP00000374565	0	FAM168B
ENSP00000318182	0	FAM156A
ENSP00000242505	0	FAM149B1
ENSP00000391993	0	FAM13C
ENSP00000037869	0	FAM136A
ENSP00000276737	0	FAM135B
ENSP00000310135	0	FAM131A
ENSP00000280057	0	FAM124A
ENSP00000298090	0	FAM122B
ENSP00000240364	0	FAM117A

ENSP00000355264	0	FAM111A
ENSP00000355204	0	FAM110B
ENSP00000274217	0	FAM105A
ENSP00000384832	0	FAM104A
ENSP00000315626	0	FAM101A
ENSP00000358454	0	F8A3
ENSP00000270530	0	EVI5L
ENSP00000340427	0	ERMP1
ENSP00000377374	0	ERGIC1
ENSP00000296741	0	ENPP6
ENSP00000332806	0	EMILIN3
ENSP00000335481	0	EMID1
ENSP00000405502	0	EGFL8
ENSP00000411023	0	EGFL8
ENSP00000384081	0	EFR3B
ENSP00000391349	0	DXO
ENSP00000251250	0	DTWD1
ENSP00000364919	0	DIRAS2
ENSP00000280886	0	DIP2C
ENSP00000295373	0	DHX57
ENSP00000252011	0	DHX35
ENSP00000331907	0	DHX34
ENSP00000225296	0	DHX33
ENSP00000365625	0	DHX16
ENSP00000373071	0	DHX16
ENSP00000389862	0	DHX16
ENSP00000390938	0	DHX16
ENSP00000391789	0	DHX16
ENSP00000393958	0	DHX16
ENSP00000396193	0	DHX16
ENSP00000334113	0	DHRSX
ENSP00000368173	0	DHRS13
ENSP00000371546	0	DEPDC5
ENSP00000367490	0	DEPDC4
ENSP00000335382	0	DEFB128
ENSP00000371847	0	DEFB125
ENSP00000365492	0	DEFB119
ENSP00000319126	0	DEFB112
ENSP00000360190	0	DEFB110
ENSP00000347810	0	DEFB107B
ENSP00000334681	0	DEFB107A
ENSP00000335281	0	DEFB105B
ENSP00000334330	0	DEFB105A
ENSP00000353128	0	DCAF12L2
ENSP00000309538	0	DAPL1
ENSP00000359571	0	CXorf66

ENSP00000347339	0	CXorf40B
ENSP00000367922	0	CXorf30
ENSP00000297866	0	CXorf22
ENSP00000368245	0	CXorf21
ENSP00000226432	0	CWH43
ENSP00000370460	0	CWC27
ENSP00000225428	0	CWC25
ENSP00000313226	0	CTXN1
ENSP00000417289	0	CTAGE8
ENSP00000419539	0	CTAGE4
ENSP00000360360	0	CT47B1
ENSP00000400046	0	CT47A8
ENSP00000415974	0	CT47A6
ENSP00000390070	0	CT47A5
ENSP00000389513	0	CT47A4
ENSP00000398559	0	CT47A3
ENSP00000414253	0	CT47A2
ENSP00000411909	0	CT47A10
ENSP00000360328	0	CT47A1
ENSP00000359759	0	CT45A5
ENSP00000405261	0	CT45A4
ENSP00000359769	0	CT45A3
ENSP00000359772	0	CT45A2
ENSP00000359777	0	CT45A1
ENSP00000344042	0	CSRNP3
ENSP00000359310	0	CSAG1
ENSP00000293925	0	CRAMP1L
ENSP00000276127	0	CPXCR1
ENSP00000320672	0	COX6B2
ENSP00000347601	0	COLCA1
ENSP00000360593	0	COA7
ENSP00000223336	0	COA1
ENSP00000262932	0	CNPY4
ENSP00000222032	0	CNFN
ENSP00000405635	0	CNEP1R1
ENSP00000362550	0	CMTR1
ENSP00000333833	0	CMTM4
ENSP00000357660	0	CLRN3
ENSP00000288861	0	CIB4
ENSP00000362601	0	CHIC1
ENSP00000324767	0	CHDC2
ENSP00000416561	0	CFB
ENSP00000306105	0	CEP89
ENSP00000292672	0	CELF5
ENSP00000402203	0	CEACAM18
ENSP00000250838	0	CDY2A

ENSP00000399604	0	CDSN
ENSP00000325301	0	CDPF1
ENSP00000381272	0	CD200R1L
ENSP00000315945	0	CD163L1
ENSP00000377547	0	CCNJL
ENSP00000365477	0	CCDC93
ENSP00000238156	0	CCDC92
ENSP00000239830	0	CCDC77
ENSP00000335325	0	CCDC73
ENSP00000277657	0	CCDC7
ENSP00000298050	0	CCDC67
ENSP00000380690	0	CCDC64
ENSP00000312399	0	CCDC63
ENSP00000293845	0	CCDC42
ENSP00000405708	0	CCDC39
ENSP00000357079	0	CCDC19
ENSP00000377577	0	CCDC176
ENSP00000341451	0	CCDC17
ENSP00000362507	0	CCDC167
ENSP00000283233	0	CCDC148
ENSP00000353942	0	CCDC135
ENSP00000307666	0	CCDC132
ENSP00000296824	0	CCDC127
ENSP00000292314	0	CCDC12
ENSP00000318429	0	CCDC114
ENSP00000414964	0	CCDC107
ENSP00000387252	0	CCDC103
ENSP00000258214	0	CCDC102A
ENSP00000366677	0	CBWD6
ENSP00000386950	0	CATSPERG
ENSP00000371037	0	CATSPERD
ENSP00000282611	0	CATSPER3
ENSP00000299957	0	CASC4
ENSP00000366317	0	CASC10
ENSP00000385247	0	CAPN14
ENSP00000295055	0	CAPN13
ENSP00000358798	0	CALHM3
ENSP00000334289	0	C9orf85
ENSP00000335616	0	C9orf47
ENSP00000395281	0	C9orf116
ENSP00000276704	0	C8orf76
ENSP00000401738	0	C8orf56
ENSP00000297145	0	C7orf60
ENSP00000350011	0	C7orf50
ENSP00000324741	0	C7orf43
ENSP00000382944	0	C6orf25

ENSP00000386146	0	C6orf226
ENSP00000403409	0	C6orf136
ENSP00000411113	0	C6orf136
ENSP00000363135	0	C6orf106
ENSP00000410635	0	C6orf100
ENSP00000378441	0	C6orf1
ENSP00000386184	0	C5orf54
ENSP00000380270	0	C5orf28
ENSP00000337044	0	C5orf24
ENSP00000391404	0	C4orf51
ENSP00000382026	0	C4orf3
ENSP00000413228	0	C3orf55
ENSP00000349732	0	C3orf18
ENSP00000381631	0	C2orf73
ENSP00000315557	0	C2orf57
ENSP00000237822	0	C2orf43
ENSP00000323339	0	C2CD3
ENSP00000248984	0	C22orf24
ENSP00000380941	0	C21orf67
ENSP00000405800	0	C2
ENSP00000407961	0	C2
ENSP00000371572	0	C1QTNF9B
ENSP00000290363	0	C1orf51
ENSP00000357082	0	C1orf204
ENSP00000288048	0	C1orf158
ENSP00000355609	0	C1orf131
ENSP00000356912	0	C1orf111
ENSP00000356311	0	C1orf106
ENSP00000356700	0	C1orf105
ENSP00000397394	0	C19orf25
ENSP00000386557	0	C19orf24
ENSP00000300091	0	C18orf54
ENSP00000317905	0	C17orf96
ENSP00000353028	0	C17orf104
ENSP00000284245	0	C16orf74
ENSP00000353854	0	C15orf39
ENSP00000376307	0	C14orf80
ENSP00000256324	0	C14orf159
ENSP00000322238	0	C14orf119
ENSP00000253233	0	C12orf65
ENSP00000386169	0	C12orf61
ENSP00000280756	0	C12orf23
ENSP00000311479	0	C11orf86
ENSP00000325508	0	C11orf71
ENSP00000280325	0	C11orf53
ENSP00000367878	0	C11orf49

ENSP00000318999	0	C11orf16
ENSP00000260276	0	C11orf1
ENSP00000361199	0	C10orf99
ENSP00000358212	0	C10orf82
ENSP00000391066	0	C10orf40
ENSP00000362376	0	C10orf35
ENSP00000299353	0	C10orf32-ASMT
ENSP00000343686	0	BTBD8
ENSP00000397759	0	BSDC1
ENSP00000413845	0	BRD2
ENSP00000344929	0	BPIFB6
ENSP00000257336	0	BIVM
ENSP00000346256	0	B3GNT6
ENSP00000355559	0	B3GALNT2
ENSP00000356590	0	AXDND1
ENSP00000347152	0	ATRNL1
ENSP00000284509	0	ATP8B4
ENSP00000311336	0	ATP8B3
ENSP00000357475	0	ATP8B2
ENSP00000371084	0	ATP8A1
ENSP00000302194	0	ATP6V1G2
ENSP00000370414	0	ATP5EP2
ENSP00000341942	0	ATP13A5
ENSP00000373079	0	ATAT1
ENSP00000409067	0	ATAT1
ENSP00000411050	0	ATAT1
ENSP00000373860	0	ASPDH
ENSP00000222250	0	ARRDC2
ENSP00000376417	0	ARMC2
ENSP00000380635	0	ARL16
ENSP00000265154	0	ARHGEF38
ENSP00000304586	0	ANKS3
ENSP00000326572	0	ANKRD62
ENSP00000353796	0	ANKRD53
ENSP00000285243	0	ANKRD40
ENSP00000314103	0	ANKRD34A
ENSP00000321731	0	ANKRD24
ENSP00000360998	0	ANKRD22
ENSP00000310874	0	ANKRD13D
ENSP00000359982	0	ANKRD13C
ENSP00000261739	0	ANKRD13A
ENSP00000367631	0	ANKEF1
ENSP00000355929	0	ANGEL2
ENSP00000320848	0	AMIGO2
ENSP00000258494	0	ALDH1L2
ENSP00000415452	0	AGAP8

ENSP00000364567	0	ADPRHL1
ENSP00000358031	0	ADAMTSL4-AS1
ENSP00000327916	0	ACSM5
ENSP00000382349	0	ACSM4
ENSP00000219054	0	ACSM2A
ENSP00000258884	0	ABHD17C
ENSP00000366240	0	ABHD17B
ENSP00000407727	0	ABHD16A
ENSP00000365063	0	ABHD13
ENSP00000343951	0	ABHD12B
ENSP00000364382	0	AAED1
ENSP00000352268	0	AADACL3
ENSP00000348911	0	AADACL2
ENSP00000333181	0	11-Mar
ENSP00000005905	0	-
ENSP00000215872	0	-
ENSP00000244249	0	-
ENSP00000250366	0	-
ENSP00000253320	0	-
ENSP00000253720	0	-
ENSP00000255998	0	-
ENSP00000258775	0	-
ENSP00000262063	0	-
ENSP00000266524	0	-
ENSP00000266813	0	-
ENSP00000268271	0	-
ENSP00000275229	0	-
ENSP00000281131	0	-
ENSP00000283050	0	-
ENSP00000284081	0	-
ENSP00000296662	0	-
ENSP00000297020	0	-
ENSP00000297416	0	-
ENSP00000300992	0	-
ENSP00000303257	0	-
ENSP00000303666	0	-
ENSP00000303817	0	-
ENSP00000305638	0	-
ENSP00000306791	0	-
ENSP00000308220	0	-
ENSP00000308412	0	-
ENSP00000308821	0	-
ENSP00000309619	0	-
ENSP00000310485	0	-
ENSP00000311103	0	-
ENSP00000314362	0	-

ENSP00000314853	0	-
ENSP00000316933	0	-
ENSP00000318279	0	-
ENSP00000318930	0	-
ENSP00000321398	0	-
ENSP00000322424	0	-
ENSP00000322952	0	-
ENSP00000324103	0	-
ENSP00000327564	0	-
ENSP00000329674	0	-
ENSP00000330358	0	-
ENSP00000330634	0	-
ENSP00000331186	0	-
ENSP00000331873	0	-
ENSP00000340476	0	-
ENSP00000341756	0	-
ENSP00000341841	0	-
ENSP00000341933	0	-
ENSP00000342810	0	-
ENSP00000343169	0	-
ENSP00000344387	0	-
ENSP00000352599	0	-
ENSP00000353858	0	-
ENSP00000353947	0	-
ENSP00000358227	0	-
ENSP00000358599	0	-
ENSP00000360679	0	-
ENSP00000362898	0	-
ENSP00000364005	0	-
ENSP00000364083	0	-
ENSP00000364776	0	-
ENSP00000365199	0	-
ENSP00000365346	0	-
ENSP00000365350	0	-
ENSP00000365769	0	-
ENSP00000366342	0	-
ENSP00000367700	0	-
ENSP00000368491	0	-
ENSP00000369441	0	-
ENSP00000369727	0	-
ENSP00000371069	0	-
ENSP00000371611	0	-
ENSP00000372510	0	-
ENSP00000372795	0	-
ENSP00000373036	0	-
ENSP00000374858	0	-

ENSP00000374930	0	-
ENSP00000374939	0	-
ENSP00000374944	0	-
ENSP00000374947	0	-
ENSP00000374955	0	-
ENSP00000374956	0	-
ENSP00000374960	0	-
ENSP00000374962	0	-
ENSP00000374967	0	-
ENSP00000374977	0	-
ENSP00000374988	0	-
ENSP00000375092	0	-
ENSP00000375094	0	-
ENSP00000375387	0	-
ENSP00000377610	0	-
ENSP00000379938	0	-
ENSP00000379974	0	-
ENSP00000380113	0	-
ENSP00000380162	0	-
ENSP00000380660	0	-
ENSP00000381147	0	-
ENSP00000381184	0	-
ENSP00000381447	0	-
ENSP00000381951	0	-
ENSP00000381975	0	-
ENSP00000382055	0	-
ENSP00000382101	0	-
ENSP00000382146	0	-
ENSP00000382339	0	-
ENSP00000383309	0	-
ENSP00000383642	0	-
ENSP00000383732	0	-
ENSP00000383859	0	-
ENSP00000384224	0	-
ENSP00000384233	0	-
ENSP00000384383	0	-
ENSP00000384761	0	-
ENSP00000385052	0	-
ENSP00000385382	0	-
ENSP00000386148	0	-
ENSP00000387841	0	-
ENSP00000387884	0	-
ENSP00000387960	0	-
ENSP00000388305	0	-
ENSP00000388831	0	-
ENSP00000389451	0	-

ENSP00000389531	0	-
ENSP00000389636	0	-
ENSP00000390381	0	-
ENSP00000391874	0	-
ENSP00000391958	0	-
ENSP00000392121	0	-
ENSP00000392127	0	-
ENSP00000392198	0	-
ENSP00000393252	0	-
ENSP00000393308	0	-
ENSP00000393390	0	-
ENSP00000393499	0	-
ENSP00000393656	0	-
ENSP00000393994	0	-
ENSP00000394145	0	-
ENSP00000394236	0	-
ENSP00000394551	0	-
ENSP00000395187	0	-
ENSP00000395225	0	-
ENSP00000395306	0	-
ENSP00000395703	0	-
ENSP00000396718	0	-
ENSP00000397024	0	-
ENSP00000397102	0	-
ENSP00000397134	0	-
ENSP00000397304	0	-
ENSP00000397405	0	-
ENSP00000398036	0	-
ENSP00000398607	0	-
ENSP00000399170	0	-
ENSP00000399186	0	-
ENSP00000399266	0	-
ENSP00000399565	0	-
ENSP00000399849	0	-
ENSP00000400348	0	-
ENSP00000400381	0	-
ENSP00000400635	0	-
ENSP00000400867	0	-
ENSP00000401138	0	-
ENSP00000401233	0	-
ENSP00000401346	0	-
ENSP00000401941	0	-
ENSP00000402181	0	-
ENSP00000402253	0	-
ENSP00000402802	0	-
ENSP00000402914	0	-

ENSP00000403698	0	-
ENSP00000403863	0	-
ENSP00000403930	0	-
ENSP00000404166	0	-
ENSP00000404820	0	-
ENSP00000404826	0	-
ENSP00000405105	0	-
ENSP00000405401	0	-
ENSP00000405426	0	-
ENSP00000405627	0	-
ENSP00000405821	0	-
ENSP00000405962	0	-
ENSP00000406217	0	-
ENSP00000406405	0	-
ENSP00000406586	0	-
ENSP00000406875	0	-
ENSP00000407037	0	-
ENSP00000407125	0	-
ENSP00000407260	0	-
ENSP00000407300	0	-
ENSP00000407311	0	-
ENSP00000407346	0	-
ENSP00000407425	0	-
ENSP00000407515	0	-
ENSP00000407618	0	-
ENSP00000407738	0	-
ENSP00000407965	0	-
ENSP00000407991	0	-
ENSP00000408017	0	-
ENSP00000408225	0	-
ENSP00000408299	0	-
ENSP00000408407	0	-
ENSP00000408451	0	-
ENSP00000408480	0	-
ENSP00000408807	0	-
ENSP00000408943	0	-
ENSP00000409022	0	-
ENSP00000409097	0	-
ENSP00000409166	0	-
ENSP00000409453	0	-
ENSP00000409912	0	-
ENSP00000410574	0	-
ENSP00000411055	0	-
ENSP00000411265	0	-
ENSP00000411379	0	-
ENSP00000411546	0	-

ENSP00000411550	0	-
ENSP00000411694	0	-
ENSP00000411730	0	-
ENSP00000411836	0	-
ENSP00000411847	0	-
ENSP00000411855	0	-
ENSP00000411990	0	-
ENSP00000412264	0	-
ENSP00000412375	0	-
ENSP00000412383	0	-
ENSP00000412423	0	-
ENSP00000412459	0	-
ENSP00000412483	0	-
ENSP00000412998	0	-
ENSP00000413134	0	-
ENSP00000413285	0	-
ENSP00000413414	0	-
ENSP00000413949	0	-
ENSP00000414234	0	-
ENSP00000414550	0	-
ENSP00000414667	0	-
ENSP00000414794	0	-
ENSP00000415010	0	-
ENSP00000415141	0	-
ENSP00000415462	0	-
ENSP00000415647	0	-
ENSP00000415665	0	-
ENSP00000416099	0	-
ENSP00000416324	0	-
ENSP00000416549	0	-
ENSP00000416564	0	-
ENSP00000419512	0	-
ENSP00000420227	0	-
